# Isomerization reactions of metal vinylidene units†

**DOI:** 10.1039/d4sc01993h

**Published:** 2024-05-03

**Authors:** Xuejuan Zheng, Fanping Huang, Xinyuan Li, Kaiyue Zhuo, Dafa Chen, Ming Luo, Haiping Xia

**Affiliations:** a Shenzhen Grubbs Institute, Department of Chemistry, Guangming Advanced Research Institute, Southern University of Science and Technology Shenzhen 518055 China luom3@sustech.edu.cn xiahp@sustech.edu.cn

## Abstract

Isomerization reactions of unsaturated molecules offer an efficient strategy in atom-economical synthesis. Although isomerization reactions of unsaturated organic and organometallic compounds, such as alkenes, alkynes, and metal carbynes, have been achieved, those of metal vinylidene units that contain cumulated double bonds have never been reported. Herein, we inaugurally discovered isomerization reactions of metal vinylidene units *via* protonation and deprotonation reactions of metal carbenes. Experimental and theoretical investigations indicate that the electrical characteristics of substituents on the rings play a crucial role in controlling the formation of metal vinylidene units. The isomerization reactions of metal vinylidene units were driven by thermodynamic forces. Moreover, one of the angles at metal vinylidenes was found as 126.9°, representing the smallest angle in metal vinylidenes and the first cyclic 4d transition metal (Ru) vinylidene complex was successfully isolated. These investigations unveil novel structures and reactivity for metal vinylidenes, offering a fresh perspective on the isomerization reactions of unsaturated molecules containing cumulative unsaturated bonds.

## Abstract 2

Isomerization reactions of unsaturated molecules offer an efficient strategy in atom-economical synthesis.

## Graphical abstract

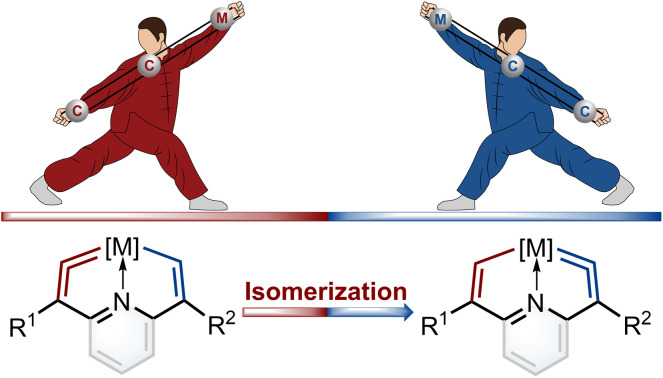

## Introduction

Isomerization reactions, wherein the primary product is isomeric with the principal reactant, stand as indispensable tools in synthetic chemistry.^1^ Isomerization reactions of unsaturated organic molecules such as alkenes and alkynes have been employed as a straightforward strategy to build their valuable corresponding isomers.^2^ For example, the direct synthesis of carvacrol from carvone can be achieved by acid-assisted alkene isomerisation.^3^ Compared with much more common isomerization reactions in unsaturated organic molecules, organometallic unsaturated compounds containing metal–carbon multiple bonds are largely unexplored. To date, acid-induced isomerization reactions of metal–carbyne complexes have been reported and employed to construct novel metallacycles,^4^ however, the isomerization reactions of metal vinylidenes have never been reported.

Metal vinylidenes (M = C_α_ = C_β_R_2_) can be regarded as metal containing heteroallenes and the first mononuclear metal vinylidene complex was isolated in 1972.^5^ Metal vinylidene complexes exhibit rich reactivity and their stoichiometric reactivity is demonstrated through three main types.^6^ (1) C_α_ is electron deficient and typically susceptible to attack by nucleophilic reagents with the exception of some examples attacked by electrophiles.^7^ The addition of nucleophiles to C_α_ results in metal carbene complexes or metal vinyl complexes; (2) C_β_ is electron-rich, making it readily reactive with electrophiles. For example, the protonation of C_β_ transforms metal vinylidene complexes to the corresponding metal carbyne complexes; (3) metal vinylidenes can undergo [2 + 2] cycloaddition reactions with alkenes or alkynes due to the existence of M = C_α_ and C_α_ = C_β_ double bonds. Based on the aforementioned abundant reactivity, metal vinylidene complexes serve as key intermediates in the anti-Markovnikov addition reactions of alkynes and participate in catalytic olefin metathesis.^8^

When the metal vinylidene units are located within rings, their synthesis and isolation become more challenging owing to the ring strain.^9^ The first cyclic metal vinylidene complex was isolated by Esteruelas group in 2004, *i.e.*, iso-metallabenzene.^9*a*^ Subsequently, several other cyclic metal vinylidene complexes have been reported such as iso-metallapyridinium derivatives^9*g*^ and those with cyclic metal vinylidene units in five-membered rings.^9*f*,*i*,*j*^ Due to the unique structure of cyclic metal vinylidenes, they exhibit distinctive reactivity related to metallacycles including the rearrangement of iso-osmabenzenes to metalated cyclopentadienes^9*c*^ and conversion of five-membered cyclic metal vinylidenes to six-membered cyclic metal vinylidenes.^9*f*^ Despite significant progress in metal vinylidenes, isomerization reactions which are achieved in various unsaturated compounds and play a significant role in chemical transformations constitute an intriguing and important topic in metal vinylidenes that demands further investigation.

**Fig. 1:**
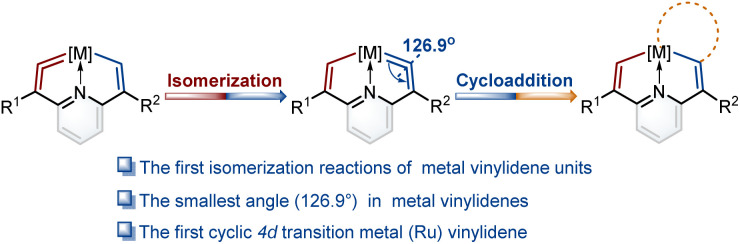
Isomerization reactions of metal vinylidene units and the utilization of cycloaddition reactions to trap isomerization products.

In the present work, we construct a new type of fused ring frameworks that incorporate metal vinylidene units. In these metallacyclic frameworks, the isomerization reactions of metal vinylidene units are discovered for the first time. The electrical properties of substituents play a crucial role in the formation and isomerization of metal vinylidene units. Notably, the angle at one of the cyclic metal vinylidenes is found as 126.9°, the smallest one in metal vinylidenes to date. In addition, the first cyclic 4d transition metal (Ru) vinylidene complex was successfully isolated (Fig. 1). Moreover, these metal vinylidenes can also be regarded as a type of CNC pincer complexes.

## Results and discussion

### Synthesis and protonation reaction of cyclic osmium vinylidene complex 1

**Fig. 2:**
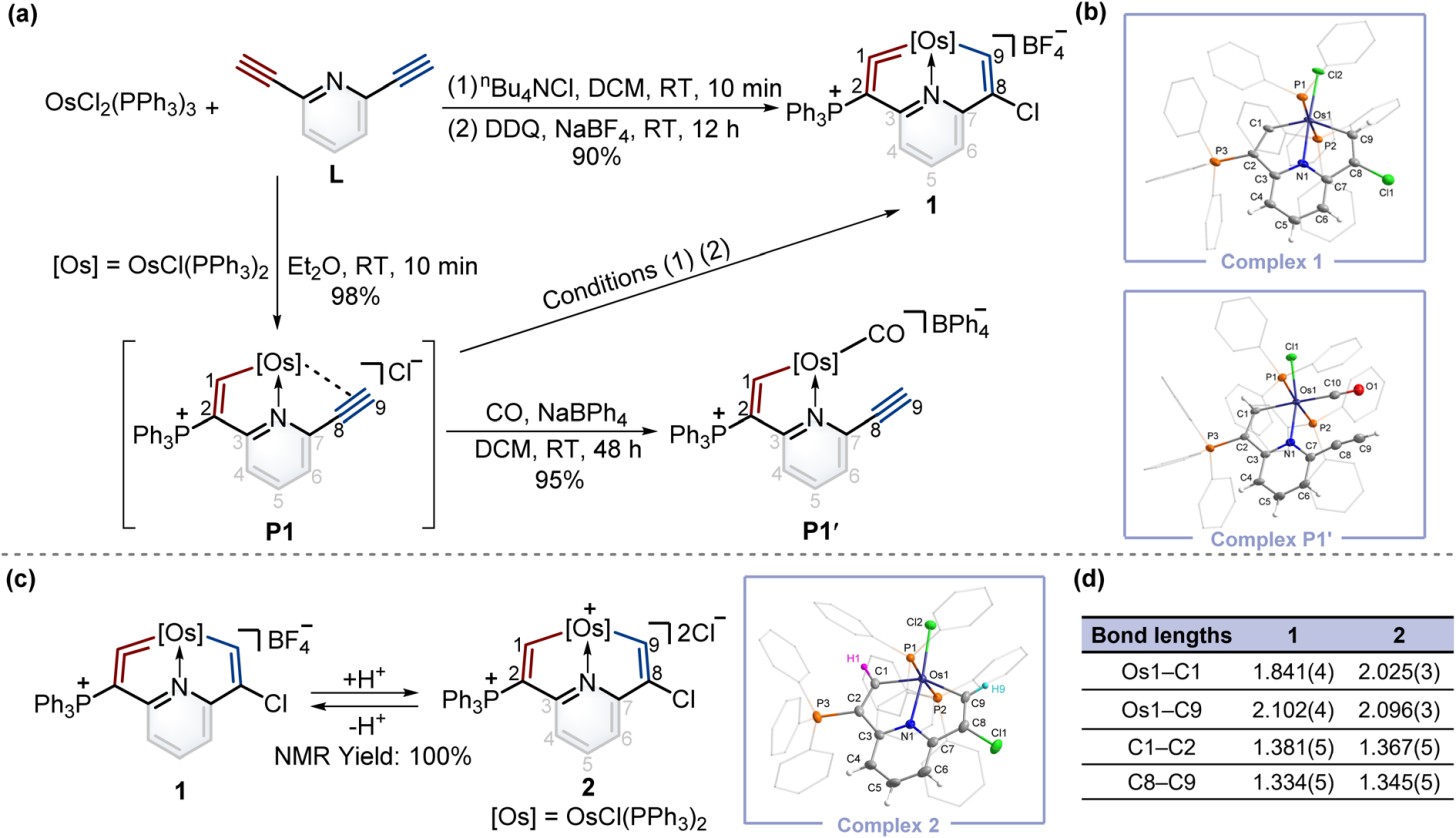
(a) Synthesis of complexes 1, P1, and P1′. (b) X-ray molecular structures for the cations of complexes 1 and P1′ were drawn with a 50% probability level. The hydrogen atoms at Ph groups were omitted for clarity. (c) The protonation reaction of complex 1 with excess AlCl_3_ in wet DCM and deprotonation reaction of complex 2 with excess Lewis bases. X-ray molecular structure for the cation of complex 2 was drawn with a 50% probability level. The hydrogen atoms at Ph groups were omitted for clarity. (d) Selected bond lengths (Å) for complexes 1 and 2.

To construct fused rings containing metal vinylidene moieties, initially we attempted the one-pot reaction of dichloride tris(triphenylphosphine)osmium (OsCl_2_(PPh_3_)_3_) with 2,6-diethynylpyridine (L) using the chloride ion (from ^*n*^Bu_4_NCl) as a nucleophile, followed by addition of 2,3-dichloro-5,6-dicyano-1,4-benzoquinone (DDQ) as an oxidant, yielding cyclic osmium vinylidene complex 1 in an excellent yield (Fig. 2a). The crystal structure of 1 reveals that an osmium vinylidene unit resides on the triphenylphosphonium-substituted ring, as supported by the bond lengths of Os1–C1 (1.841 Å) and C1–C2 (1.381 Å) (Fig. 2b). A proposed mechanism for the formation of complex 1 is shown in the ESI (Scheme S1).† To elucidate the mechanism, the attempt to characterize the key intermediate P1 isolated as a brownish-yellow solid from the reaction of OsCl_2_(PPh_3_)_3_ with L in Et_2_O failed due to its poor solubility and stability in solution. Fortunately, by stirring P1 in an atmosphere of CO, P1′ was isolated and characterized by X-ray diffraction analysis wherein a CO group replaced the alkynyl group to coordinate with the osmium center, indicating the reasonability of the inferred structure of P1 (Fig. 2b).

To realize the isomerization reactions of osmium vinylidenes, we supposed that the addition and then removal of protons at the osmium vinylidenes might be a possible option. As shown in Fig. 2c, upon reaction of complex 1 with excessive AlCl_3_ in wet DCM, the protonation product 2 was obtained exclusively where electrophilic addition of the protons to the osmium vinylidene unit occurred. However, the deprotonation reaction of complex 2 by a large excess of Lewis bases (such as Et_2_O and CH_3_OH) regenerated 1 rather than produced the isomerization product with a cyclic osmium vinylidene unit in the chlorine-substituted ring. Complex 2 was stable only under strong acidic conditions and characterized by NMR spectroscopy and X-ray crystallography. The bond lengths of Os1–C1 (2.025 Å) and Os1–C9 (2.096 Å) are almost identical and indicate Os–C single bonds^10^ as shown in Fig. 2d. The proton signals of H1 (10.10 ppm) and H9 (8.33 ppm), and the carbon signals of C1 (210.79 ppm) and C9 (182.03 ppm) are in accordance with its crystal structure.

### Isomerization reaction of osmium vinylidene units followed by capture of other reagents and DFT theoretical calculations

**Fig. 3:**
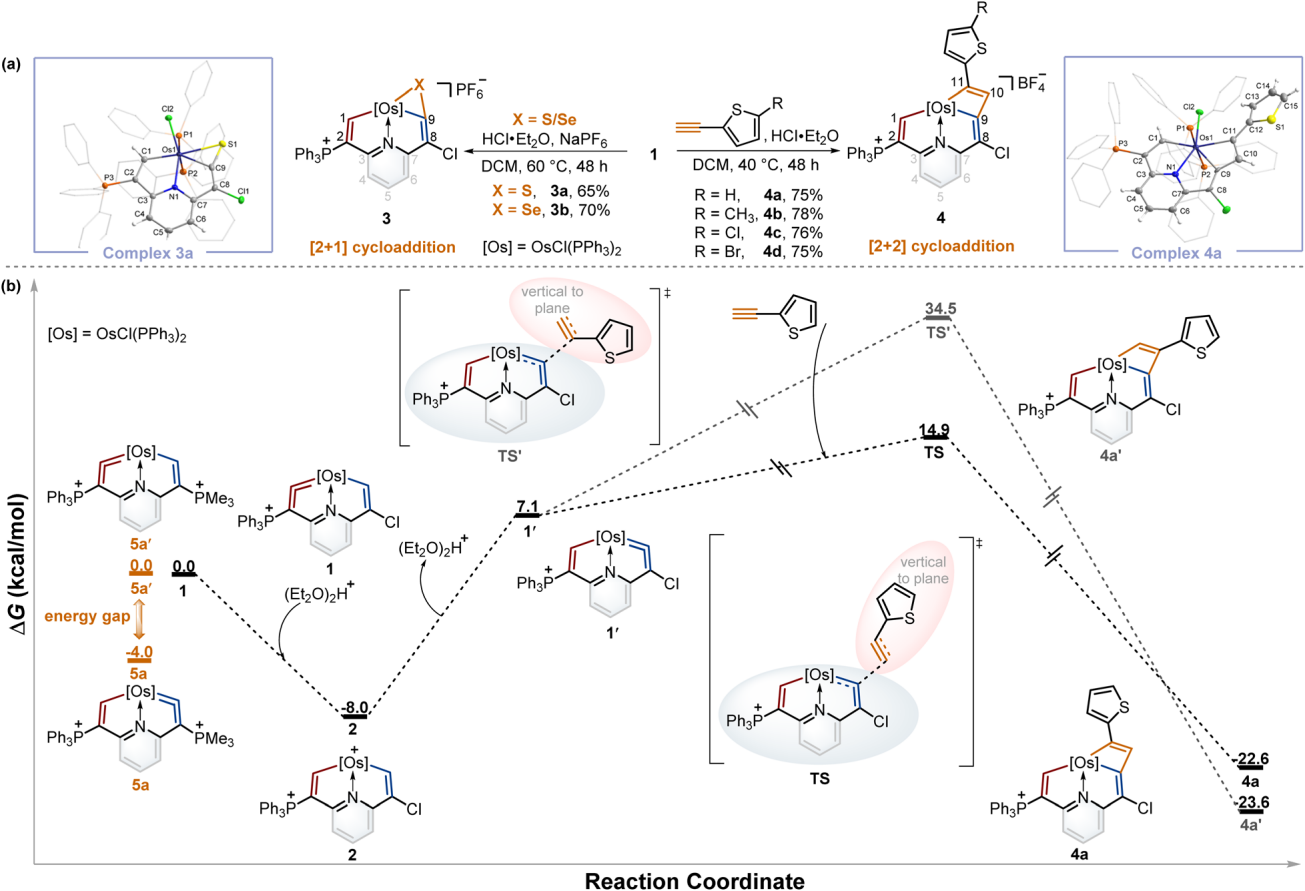
(a) The cycloaddition reactions of complex 1 with sulfur, selenium, and ethynylthiophene derivatives. X-ray molecular structures for the cations of complexes 3a and 4a were drawn with a 50% probability level. The hydrogen atoms at Ph groups were omitted for clarity. (b) DFT-calculated mechanism for the formation of complex 4a (black line) and relative energies between complexes 5a and 5a′ (orange line) at 298 K. Energies are given in kcal mol^−1^.

From the above experiments, the isomerization product might be labile and thus prevented isolation, and we assumed it could be captured by the cycloaddition reactions of metal vinylidene with other reagents. By screening the type and equivalent of acids and temperatures, we found that the reactions of sulfur and selenium with 1 in the presence of 0.5 equivalent of HCl·Et_2_O at 60 °C led to the respective products 3a and 3b*via* the [2 + 1] cycloaddition process in good yields (Table S1†). In a similar fashion, [2 + 2] cycloadditions between the osmium vinylidene and various ethynylthiophene derivatives were also successfully realized, affording complexes 4. Complexes 3a and 4a were successfully crystallographically characterized (Fig. 3a). Taking complex 3a as an example, the bond length of Os1–C1 (2.027 Å) is longer than that of complex 1, and Os1–C9 (2.055 Å) exhibits a similar bond length, both indicating Os–C single bond characters. Obviously, the cycloaddition reactions selectively occurred in the chlorine-substituted ring, as opposed to the triphenylphosphonium-substituted ring containing an osmium vinylidene unit in complex 1, suggesting that the isomerization reaction of osmium vinylidene units took place followed by capture of other reagents.

To gain further insights into the regeneration of complex 1 by complex 2 with Lewis bases and isomerization reactions of metal vinylidene promoted by the cycloaddition reactions, DFT calculations were carried out to help explain the formation of 4a (Fig. 3b, black line). The protonation of complex 1 affords complex 2 with an exergonic effect of 8.0 kcal mol^−1^, aligning with experimental isolation of 2. In comparison, the deprotonation of complex 2 in the chlorine-substituted has a higher energy gap of 15.1 kcal mol^−1^ between 1′ and 2. This indicates 1 is more thermodynamically stable than 1′, consistent with the regeneration of 1. Further calculations on regioselective [2 + 2] cycloadditions between 1′ and ethynylthiophene show that the transition state (TS) of terminal carbon interacting with osmium vinylidene has a much lower barrier compared with that (TS') of non-terminal carbon interacting with osmium vinylidene even though the final cycloaddition products (4a and 4a′) have similar energies. Consequently, the isomerization product 1′ cannot be isolated due to the higher energy. However, it can be successfully captured by the cycloaddition reactions, facilitated by the formation of products with lower energies that promote the isomerization process.

A previous study has shown that the phosphonium substituents play a key role in stabilizing cyclic osmium vinylidene complexes.^9*c*^ To acquire the osmium vinylidene units in the non-triphenylphosphonium-substituted ring, the phosphines rather than chloride ions could be used as nucleophiles in the formation of cyclic osmium vinylidene complexes. We calculated the relative energies of osmium vinylidene units in the double phosphonium-substituted system and found that the relative energy of 5a (osmium vinylidene unit in the trimethylphosphonium-substituted ring) was lower than that of 5a′ (osmium vinylidene unit in the triphenylphosphonium-substituted ring), indicating the possibility of obtaining complex 5a (Fig. 3b, orange line).

### Synthesis of cyclic osmium vinylidene complexes 5 and natural bond orbital (NBO) analysis

**Fig. 4:**
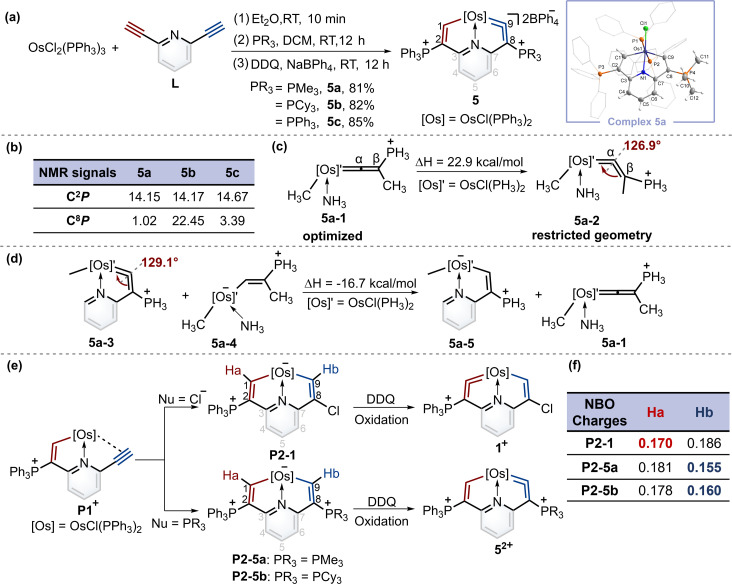
(a) Synthesis of cyclic osmium vinylidene complexes 5. X-ray molecular structure for the cation of complex 5a was drawn with a 50% probability level. The hydrogen atoms at Ph groups were omitted for clarity. (b) Selected NMR chemical shifts (ppm) for 5a–5c. (c) The calculated strain in the cyclic osmium vinylidene structure of 5a was based on the acyclic reference compound. (d) The calculated ring strain energy of 5a based on the isodesmic reactions. (e) A plausible mechanism for the formation of 1^+^ and 5^2+^ from P1^+^. (f) NBO charges of intermediates P2-1, P2-5a and P2-5b.

When PMe_3_ was employed, as expected by the theoretical calculations, complex 5a was successfully isolated in a good yield. Similarly, when the nucleophile was bulky phosphine (*i.e.*, PCy_3_), product 5b was obtained. In particular, triphenylphosphine gave the complex 5c with a symmetrical structure. The osmium vinylidene unit was depicted on the same sides to ensure alignment with complexes 5a and 5b (Fig. 4a). Nevertheless, the expected cycloaddition products were not produced by treatment of 5 with sulfur, selenium and ethynylthiophene derivatives even under heating conditions. The presence of bulky phosphonium substituents is likely to stabilize the osmium vinylidene species. However, their steric and electron-withdrawing properties may concurrently diminish reactivity.

The structure of complex 5a was confirmed by X-ray diffraction analysis as shown in Fig. 4a. It contains an osmium vinylidene unit in the trimethylphosphonium-substituted ring as evidenced by bond lengths of Os1–C9 (1.907 Å) and C9–C8 (1.371 Å). Remarkably, the angle at the osmium vinylidene (Os1–C9–C8) is 126.9°, which is smaller than reported angles in metal vinylidenes (based on a search of the Cambridge Structural Database, CSD version 5.45, in November 2023). NMR spectra data agree with the crystal structure, and the signals of C9 (330.68 ppm) and C8 (101.32 ppm) agree with the osmium vinylidene complex.^11^ In the ^31^P{^1^H} NMR spectrum, the signal of C^2^*P* is observed at 14.15 ppm. Similar chemical shifts of C^2^*P* are also found in complexes 5b and 5c due to their similar chemical environment induced by triphenylphosphonium groups (14.17 ppm for 5b and 14.67 ppm for 5c). In comparison, different chemical shifts of C^8^*P* are found in complexes 5a–5c because of various phosphonium substituents (Fig. 4b). Cyclic osmium vinylidene 5c was also characterized by X-ray diffraction analysis. However, its symmetrical structure diminishes the significance of discussing the structural data. For example, the angle at Os1–C9–C8 (126.3°) in 5c is strongly influenced by Os1–C1–C2 (121.4°) as shown in Fig. S12† for the details on the crystal structure of 5c.

Since the smallest angle (126.9°) in metal vinylidenes was found in 5a. Model DFT computations based on the crystal structure of 5a were carried out to evaluate the ring strain in it wherein PH_3_ ligands replaced the PPh_3_ and PMe_3_. The reference species 5a-1 is strain-free owing to its linear structure, and the right side of the equation of 5a-2 is the fixed angle acquired from the crystal structure of 5a with restricted geometry optimizations^12^ (Fig. 4c). The computed energy difference is 22.9 kcal mol^−1^, similar to those found in the five-membered cyclic metal vinylidene.^9*f*,*j*^ The HOMO orbitals of linear (5a-1) and bent (5a-2) metal vinylidenes have been calculated (Fig. S155†). The HOMO orbital of the linear metal vinylidene clearly occupies the C_β_ atom whereas that of bent metal vinylidene occupies the C_α_ atom. The bent geometry of metal vinylidene alters the electron distributions of HOMO orbitals, thereby leading to selective protonation reactions at the C_α_ atom. Similar changes in electron distributions of HOMO orbitals are also observed in highly bent allenes.^13^ The ring strain in the cyclic metal vinylidene structure was also estimated through the enthalpy change of isodesmic reactions (Fig. 4d). The bond angle optimized at the metal vinylidene unit is 129.1°, which is larger than that observed in 5a. As a result, the computed strain energy (16.7 kcal mol^−1^) is smaller than that between 5a-2 and 5a-1.

Despite that phosphonium substituents can help stabilize cyclic osmium vinylidene complexes, an intriguing question is why the osmium vinylidenes in complexes 1 and 5 are located in different rings. A plausible mechanism for the formation of cyclic osmium vinylidene complexes is shown in Fig. 4e. The P1^+^ was attacked by different nucleophiles to generate the key intermediates P2, followed by the hydride abstraction (Ha or Hb) with the oxidation of DDQ^14^ that determined the site of osmium vinylidene units. However, the expected intermediates P2-1, P2-5a and P2-5b could only be detected by ESI-MS, as their purification failed due to inherent instabilities (Fig. S1–S4†). Natural bond orbital (NBO) analysis^15^ was performed to illustrate the electron density of Ha and Hb in intermediates P2 (Fig. 4f). In intermediate P2-1, the NBO charge of Ha is smaller than that of Hb, indicating that the removal of Ha is easier than Hb by DDQ, and osmium vinylidene is formed in the triphenylphosphonium-substituted ring. In comparison, the NBO charges of Ha in intermediates P2-5a and P2-5b are larger than those of Hb, manifesting that the removal of Hb is easier and the osmium vinylidene is generated in the trialkylphosphonium-substituted ring. The different NBO charges of hydrogens at C1 and C9 carbon atoms are derived from the different substituents at C2 and C8 carbon atoms. The stronger electron-donating capability of phosphonium substituents at C8 leads to smaller NBO charges on OsC*H* in intermediates P2, facilitating easier hydride abstraction by DDQ and the formation of metal vinylidenes. Hence, we speculated that the usage of the weaker electron-donating capability of phosphines compared to PMe_3_ could lead to the products containing osmium vinylidene units in the triphenylphosphonium-substituted ring.

### Synthesis of cyclic osmium vinylidene isomers and isomerization reactions of metal vinylidene units

**Fig. 5:**
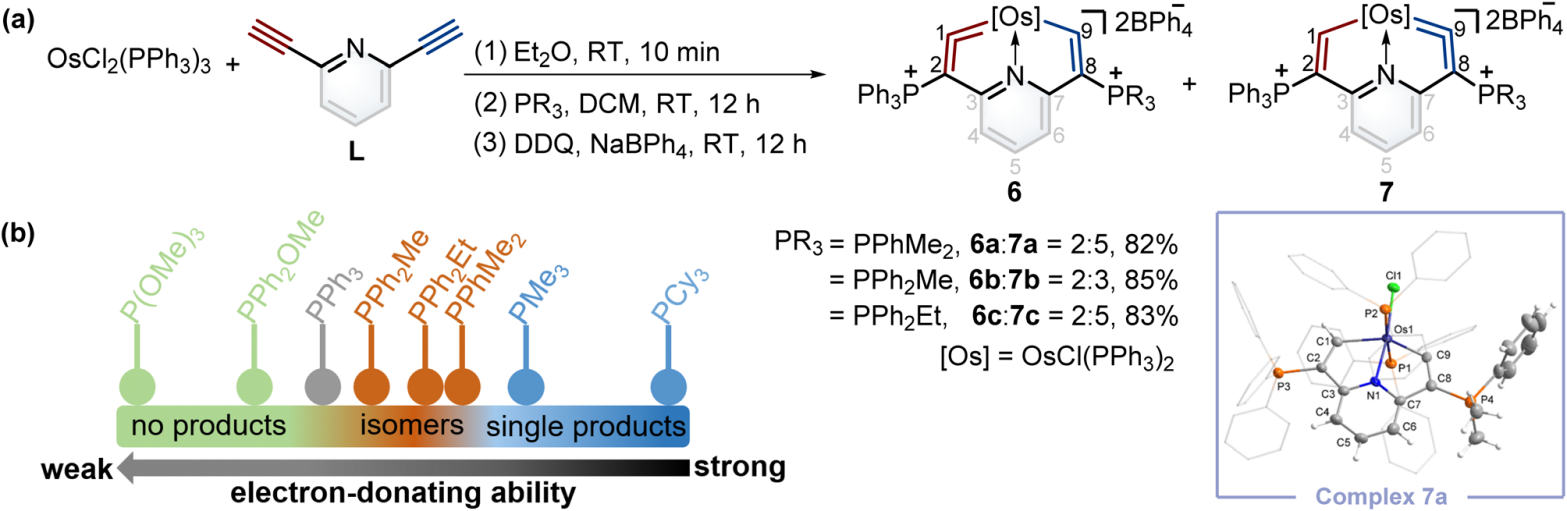
(a) Synthesis of cyclic osmium vinylidene complexes 6 and 7. X-ray molecular structure for the cation of complex 7a was drawn with a 50% probability level. The hydrogen atoms at Ph groups were omitted for clarity. (b) The reaction results from different phosphines depending on their electronic properties.

According to Tolman's map of the electronic properties of phosphines,^16^ the electron-donating capabilities of dimethylphenylphosphine (PPhMe_2_), methyldiphenylphosphine (PPh_2_Me), and ethyldiphenylphosphine (PPh_2_Et) are weaker than that of PMe_3_ yet stronger than that of PPh_3_, and they could be utilized as nucleophiles to construct cyclic metal vinylidenes. To our delight, cyclic osmium vinylidene isomers 6 and 7 bearing osmium vinylidene units in different rings were found (Fig. 5a). In these isomers, the metal vinylidene units are majorly located on the side with slightly stronger electron-donating ligands compared to PPh_3_, supported by NMR spectroscopy. The crystal of 7a was obtained from the DCM solution layered by hexane, allowing for the determination of the solid state structure. The bond lengths of Os1–C9 (1.874 Å) and C9–C8 (1.356 Å) confirm that the Os1–C9–C8 is an osmium vinylidene moiety. The angle at the osmium vinylidene unit is 127.6°. Despite the failure to obtain the crystals of 6 probably due to the higher ratios of 7 in the isomers, they were fully characterized by NMR spectroscopy, elemental analysis, and high-resolution mass spectrometry. For example, the doublet signal of H9 in 6a is observed at 10.98 ppm and similar to that of H1 (11.32 ppm in 7a), and the phosphonium signals in the ^31^P{^1^H} NMR spectrum of 6a (3.18 ppm for C^2^*P* and 10.69 ppm for C^8^*P*) are comparable to those found in 7a (14.20 ppm for C^2^*P* and −0.82 ppm for C^8^*P*). In the osmium vinylidene units, the signals for C1 (333.10 ppm) in 6a and C9 in 7a (332.92 ppm) are almost the same. Similar chemical shifts and coupling constants are also found for C2 (97.04 ppm, *J*_P–C_ = 116.2 Hz) in 6a and C8 (100.94 ppm, *J*_P–C_ = 110.2 Hz) in 7a because they are structural isomers. Other phosphines with electron-donating ability weaker than PPh_3_ (such as PPh_2_OMe and P(OMe)_3_) were investigated under the same conditions. These phosphines (green dots) were found unable to produce the final cyclic metal vinylidenes (Fig. 5b). This might be attributed to their weak nucleophilic ability, preventing the formation of intermediates P2. Therefore, the formation of cyclic metal vinylidene complexes was significantly influenced by the electronic effects of tertiary phosphines.

**Fig. 6:**
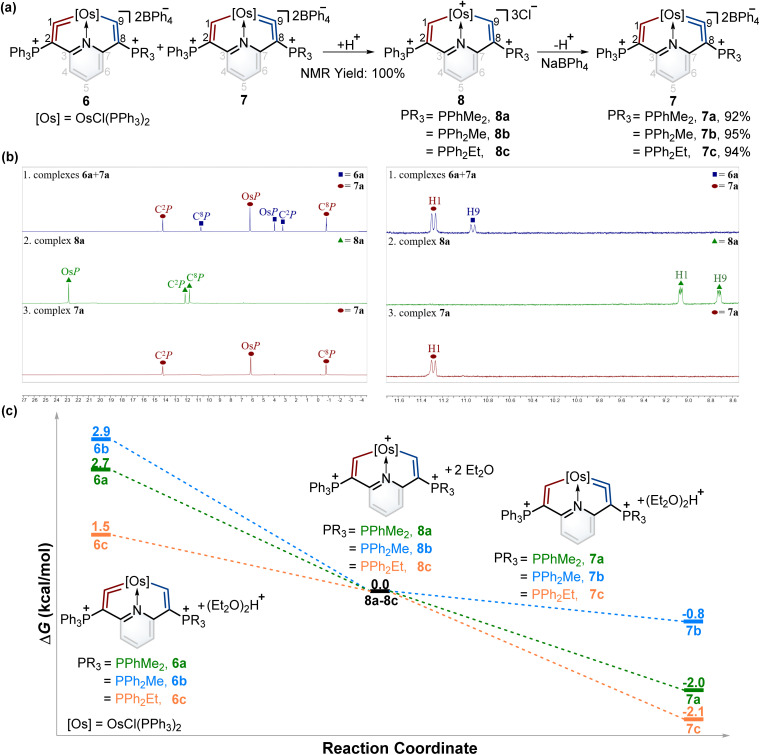
(a) The protonation of complexes 6 and 7 with excess AlCl_3_ in wet DCM and deprotonation of complexes 8 with excess Lewis bases and NaBPh_4_. (b) *In situ*^31^P{^1^H} NMR spectrum for transformation of isomers 6b and 7b to single product 7b. (left) *In situ*^1^H NMR spectrum for transformation of isomers 6b and 7b to single product 7b (right). (c) DFT-calculated the Gibbs energies for the formation of 7 at 298 K. Energies are given in kcal mol^−1^.

The successful isomerization reaction of the osmium vinylidene unit in complex 1 with a single phosphonium group prompted us to investigate the isomerization reactions of the isomers 6 and 7 with double phosphonium groups. Accordingly, the isomers (6 and 7) were treated with acids and resulted in the quantitative production of 8 through the electrophilic addition of protons to osmium vinylidenes, as confirmed by NMR spectra. Then removal of protons in the osmium vinyl units was carried out by Lewis bases, and complexes 8 completely transformed to complexes 7 (Fig. 6a). As a result, isomerization reactions of the osmium vinylidene units in the double phosphonium system were achieved, and complexes 8 could be regarded as intermediates for the transformation of complexes 6 to their isomers 7, as evidenced by *in situ* NMR spectrum (Fig. 6b). Complexes 8 were characterized by NMR spectroscopy. Complexes 8a–8c have the same basic skeleton, and here complex 8a is discussed. The signals of H1 (9.00 ppm) and H9 (8.64 ppm) are similar to those found in complex 2 (10.10 ppm for H1 and 8.33 ppm for H9) which prove the electrophilic addition of protons. The signals of C1 (212.76 ppm) and C9 (209.49 ppm) are similar, consistent with osmium carbon single bond characters. The phosphonium groups in the ^31^P{^1^H} NMR spectrum (12.13 ppm for C^2^*P* and 11.75 ppm for C^8^*P*) in 8a display similarly owing to the disappearance of the osmium vinylidene unit. DFT calculations reveal that the formation of complexes 7 is more feasible than complexes 6, attributed to the Gibbs free energies (Fig. 6c). Based on the observations of formation and isomerization reactions of cyclic osmium vinylidenes, we find that the isomers with the metal vinylidene units located on the side with stronger electron-donating phosphine ligands are more thermodynamically stable (Fig. S156†). Both formation and isomerization reactions prefer to produce more thermodynamically stable products. Once these thermodynamically stable products are isolated, the other isomers cannot be regenerated by the reactions of protonation products (8a3+–8e3+) with a Lewis base, even though the protons (Hb) have smaller NBO charges (Fig. S157†). In comparison, the hydride abstraction process is affected by NBO charges of Hb. We rationalize both thermodynamic energy and NBO charges of protons (kinetic factors) control the hydride abstraction process. However, the kinetic products in the deprotonation reactions are not observed because of the low barrier energies of proposed intermediates (Fig. S158†), and thus only thermodynamically stable products are formed.

### Synthesis of a cyclic ruthenium vinylidene complex

**Fig. 7:**
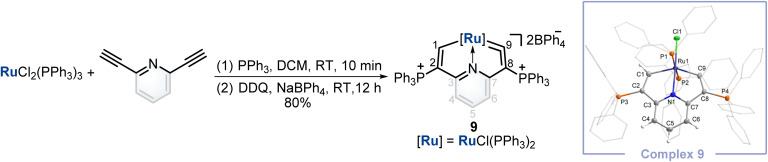
Synthesis of cyclic ruthenium vinylidene complex 9. X-ray molecular structure for the cation of complex 9 was drawn with a 50% probability level. The hydrogen atoms at Ph groups were omitted for clarity.

Finally, inspired by this facile method that could construct cyclic osmium vinylidene with interesting isomerization reactivity, the synthesis of a cyclic ruthenium vinylidene complex was also performed. Under similar conditions, the cyclic ruthenium vinylidene complex 9 with double phosphonium groups was successfully isolated and characterized which represented the first cyclic metal vinylidene complex incorporating a 4d transition metal (Fig. 7). The bond lengths of Ru1–C9 (1.916 Å) and C9–C8 (1.353 Å) convince its ruthenium vinylidene structure.^11^ In comparison, the bond length of Ru1–C1 (1.998 Å) signifies a Ru–C single bond. NMR data are in accordance with its crystal structure, for example, the signals of C9 and C1 are detected at 366.80 and 237.88 ppm, respectively.

## Conclusions

In summary, the isomerization reactions of metal vinylidene units were achieved for the first time *via* protonation and deprotonation reactions of metal carbenes. The electrical properties of substituents on the rings, as revealed by both experimental and theoretical studies, emerge as key factors in the formation of metal vinylidene units. Moreover, DFT calculations indicated that thermodynamic forces facilitated the isomerization reactions of metal vinylidene units. In addition, the smallest angle (126.9°) in metal vinylidenes was found and the bent geometry would facilitate the electrophilic addition at electron deficient C_α_ of metal vinylidenes. Moreover, the first cyclic 4d transition metal vinylidene complex was successfully isolated. These fascinating findings not only provide a new pathway for building molecules that incorporate cyclic metal vinylidenes but also may offer a fresh viewpoint on the isomerization reactions of cumulative unsaturated bonds. Isolation and further distinctive reactivity of metallacyclic vinylidene complexes are the subjects of ongoing research.

## Data availability

The validation experiments, experimental procedures, characterization, and computational details of this manuscript can be found in the ESI.† Crystallographic data have been deposited at the Cambridge Crystallographic Data Centre (CCDC) under accession numbers 2314889 (1), 2314895 (P1′), 2314890 (2), 2314891 (3a), 2314892 (4a), 2314893 (4d), 2314886 (5a), 2314884 (5c), 2314888 (7a)and 2314894 (9).

## Author contributions

X. Z., M. L., and H. X. designed this project; X. Z. performed the experiments. F. H. solved all the X-ray structures. M. L. conducted the computational studies. X. Z., F. H., X. L., K. Z., D. C, M. L. and H. X. analyzed the data and prepared the paper. All of the authors discussed the results and contributed to the preparation of the final manuscript.

## Conflicts of interest

There are no conflicts to declare.

## Supplementary Material

SC-015-D4SC01993H-s001

SC-015-D4SC01993H-s002
