# Supplementary material for: Isomerization reactions of metal vinylidene units
Source: Chem Sci. 2024 May 3;15(22):8443–50. doi: 10.1039/d4sc01993h (PMC11151869; doi:10.1039/d4sc01993h)
Supplement: SC-015-D4SC01993H-s001 [file SC-015-D4SC01993H-s001.pdf]

Electronic Supplementary Material (ESI) for Chemical Science.
This journal is © The Royal Society of Chemistry 2024

Supplementary Information

Table of Contents

1. Proposed Mechanisms and Validation Experiment Spectra S1

2. Experimental Procedures S9

3. X-ray Crystallographic Analysis S26

4. NMR and HRMS Spectra S37

5. Thermal Stability Experiments S107

6. Theoretical Calculations S108

7. Cartesian Coordinates S110

8. References S158

1. Proposed Mechanisms and Validation Experiment Spectra

Scheme S1. A possible mechanism for the formation of 1.

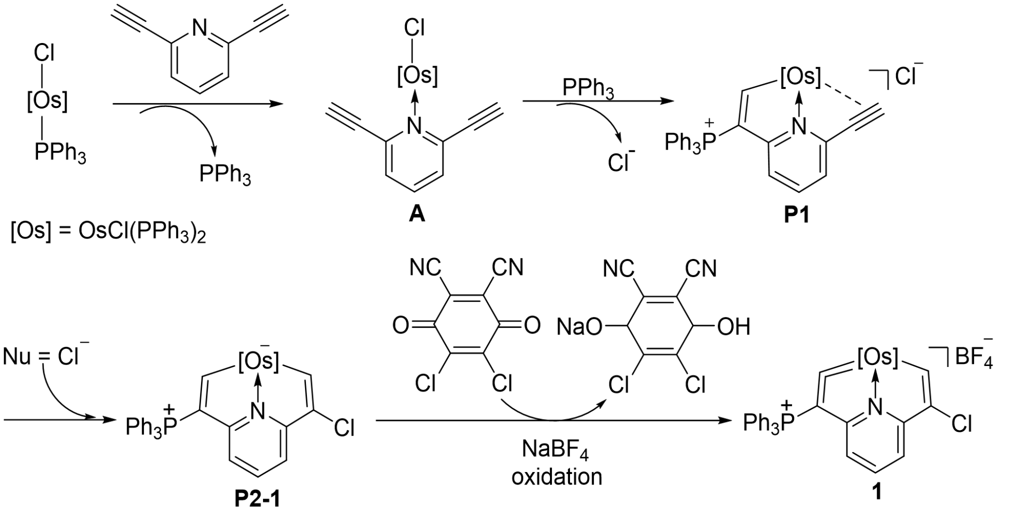

A plausible mechanism for the formation of osmium vinylidene complex **1** is proposed in Scheme S1. The process initiated with the coordination of 2,6-diethynylpridine to the metal center, associated with the dissociation of a PPh_3 ligand, resulting in intermediate **A**. Subsequently, nucleophilic addition of PPh_3 to the C≡C triple bond, followed by the removal of a chloride ion, led to the formation of the osmium vinyl intermediate **P1**. Then, another nucleophile (chloride ion) was added to the alkynyl group generated a metallacycle intermediate **P2-1**. Finally, the oxidative dehydrogenation of **P2-1** by DDQ, followed by the addition of $NaBF_4$, yielded cyclic osmium vinylidene complex **1**. The expected intermediate **P2-1** could be detected by ESI-MS.

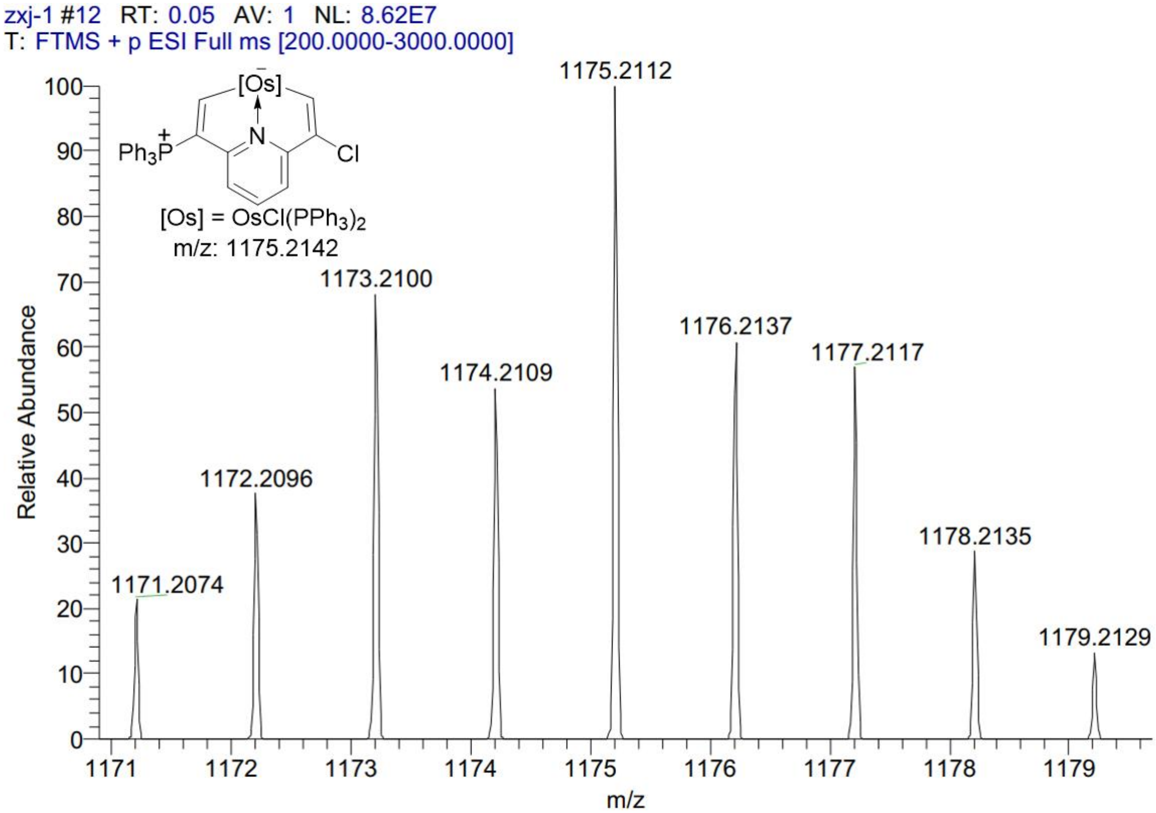

Figure S1. Positive-ion ESI-MS spectrum of [**P2-1**] measured in methanol.

Scheme S2. A possible mechanism for the formation of 5.

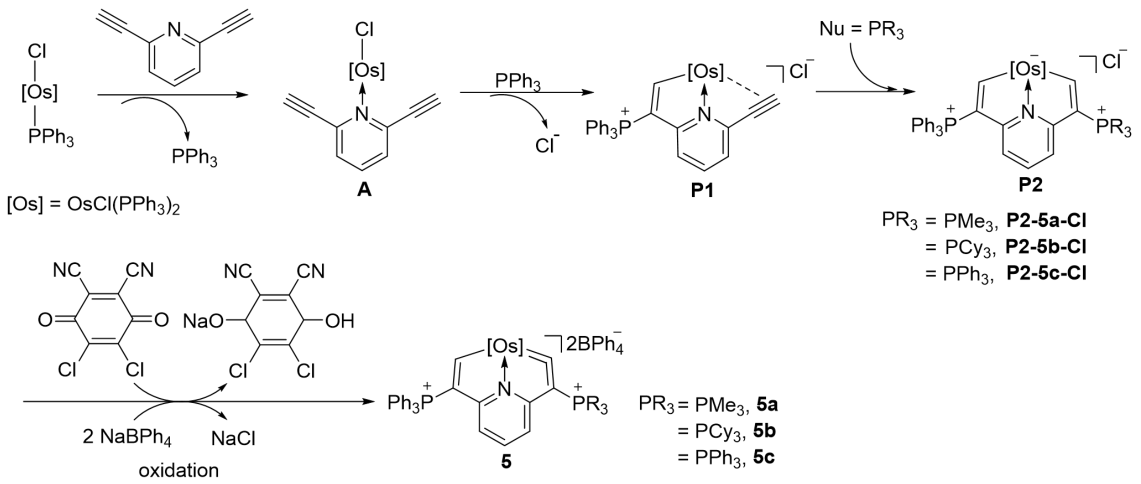

A plausible mechanism for the formation of osmium vinylidene complexes **5** is proposed in Scheme S2. The process before **P1** was the same with the formation of osmium vinylidene complex **1**. Another nucleophiles (tertiary phosphines, PMe_3, PCy_3, PPh_3) were added to the alkynyl group generated metallacycle intermediates **P2,** respectively. Finally, the oxidative dehydrogenation of **P2** with DDQ, followed by the addition of $NaBPh_4$, yielded a series of cyclic osmium vinylidene complexes **5**. The expected intermediates **P2-5a**, **P2-5b** and **P2-5c** could be detected by ESI-MS.

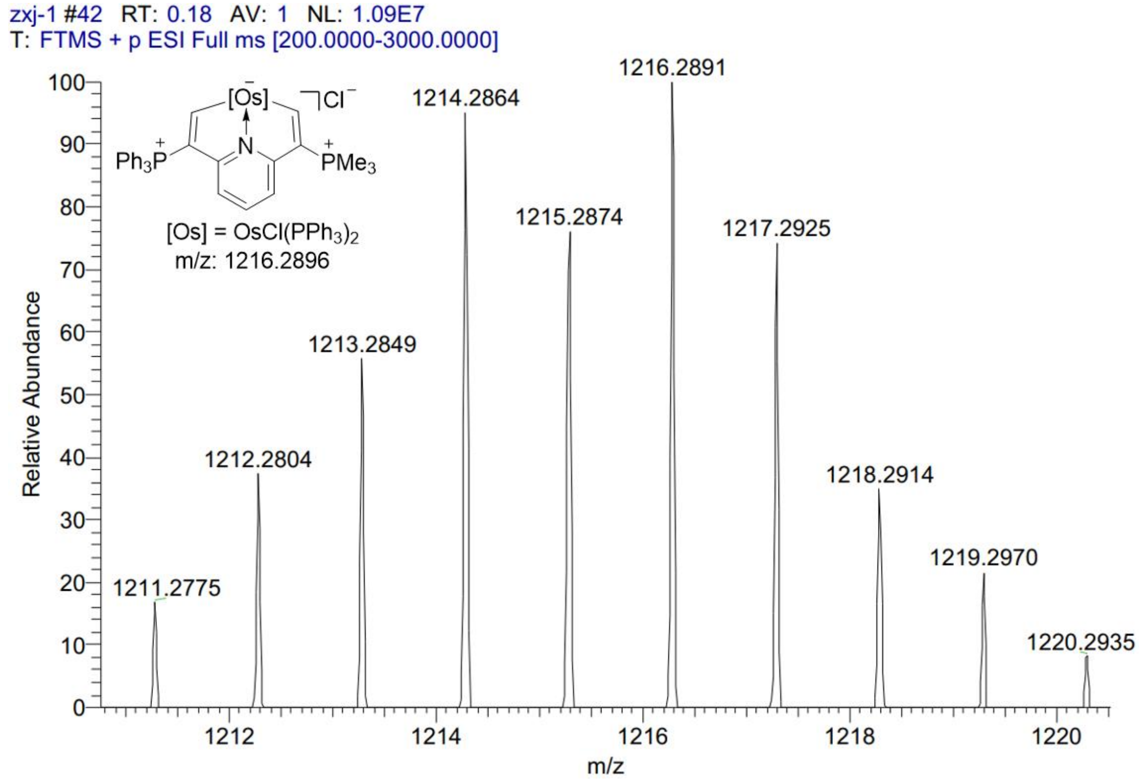

Figure S2. Positive-ion ESI-MS spectrum of [**P2-5a**]$^+$ measured in methanol.

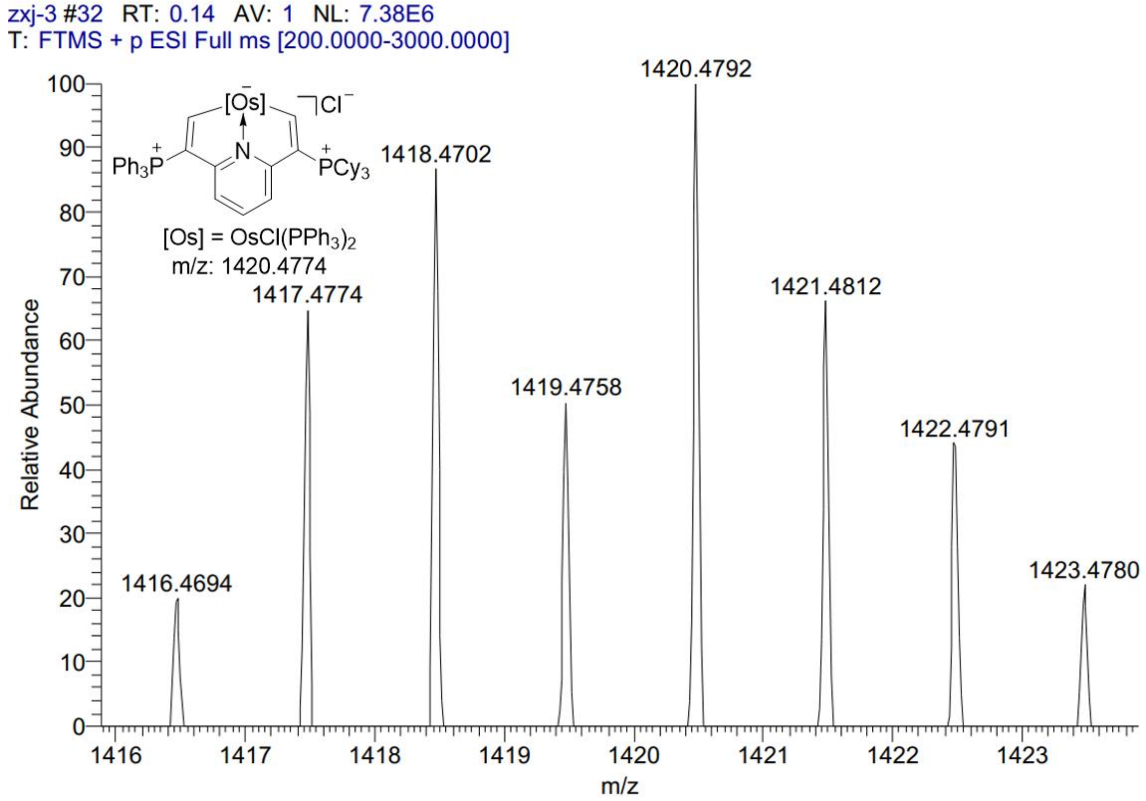

Figure S3. Positive-ion ESI-MS spectrum of [**P2-5b**]$^+$ measured in methanol.

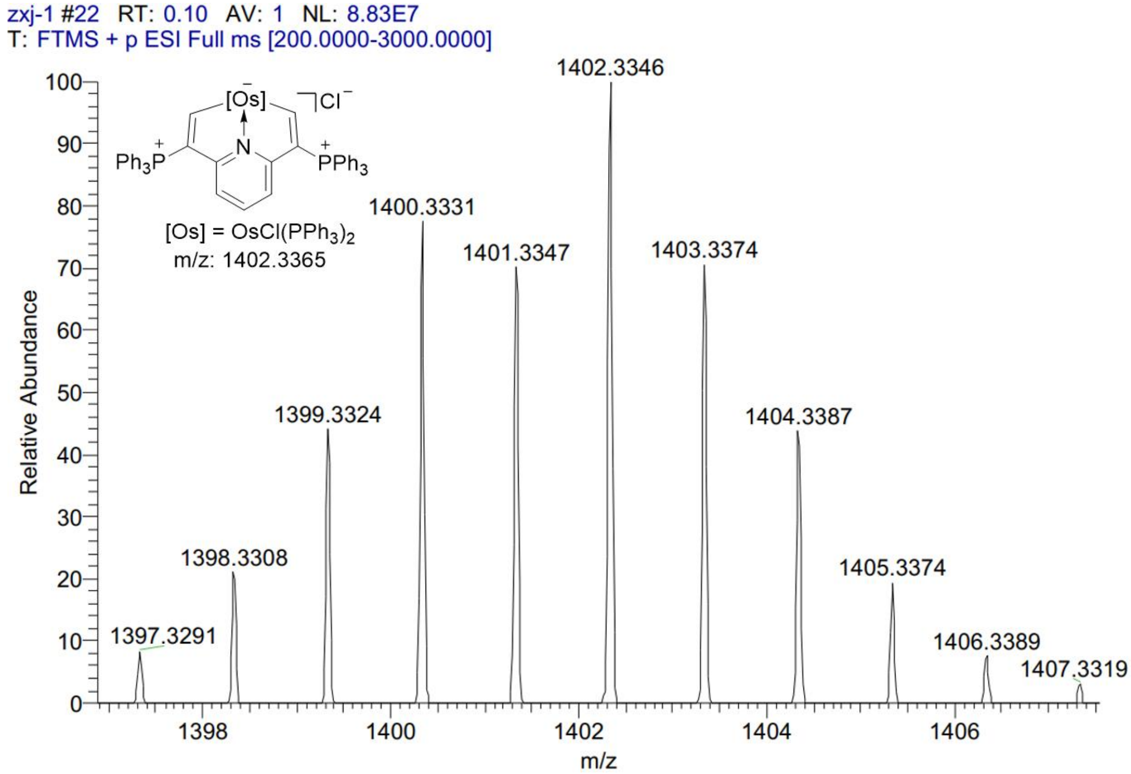

Figure S4. Positive-ion ESI-MS spectrum of [**P2-5c**]$^+$ measured in methanol.

Scheme S3. A possible mechanism for the formation of isomers (6 and 7) and their transformation to single species 7.

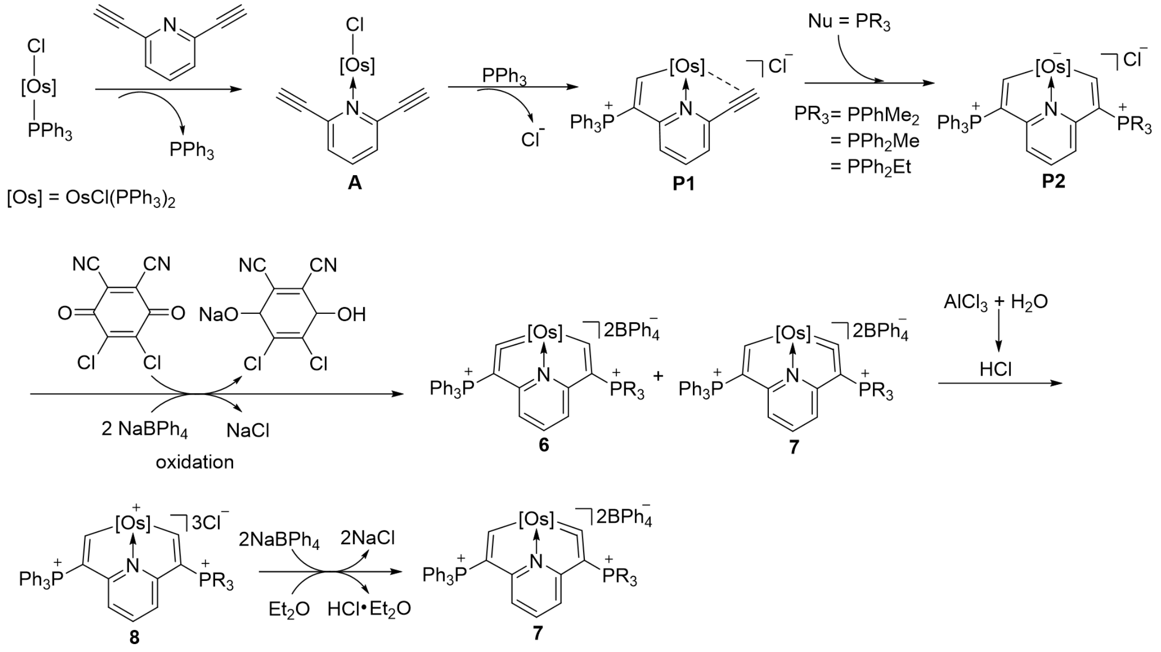

A plausible mechanism for the formation of isomers (**6** and **7**) and their transformation to single species 7 is proposed in Scheme S3. The process before **P2** was the same with the formation of osmium vinylidene complexes **5** by using different tertiary phosphines ($PPhMe_2$, PPh_2Me, PPh_2Et). Then, the oxidative dehydrogenation of **P2** with DDQ, followed by the addition of $NaBPh_4$, yielded a series of cyclic osmium vinylidene isomers (**6** and **7**). Subsequently, The addition of protons with HCl (generated from the hydrolysis of $AlCl_3$) at osmium vinylidene units of isomers (**6** and **7**) gave the complexes **8**. Finally, the removal of protons by Lewis base Et_2O, coupled with exchange of the counter ions by $NaBPh_4$, afforded the final products **7**.

Scheme S4. A possible mechanism for the formation of 3 and 4.

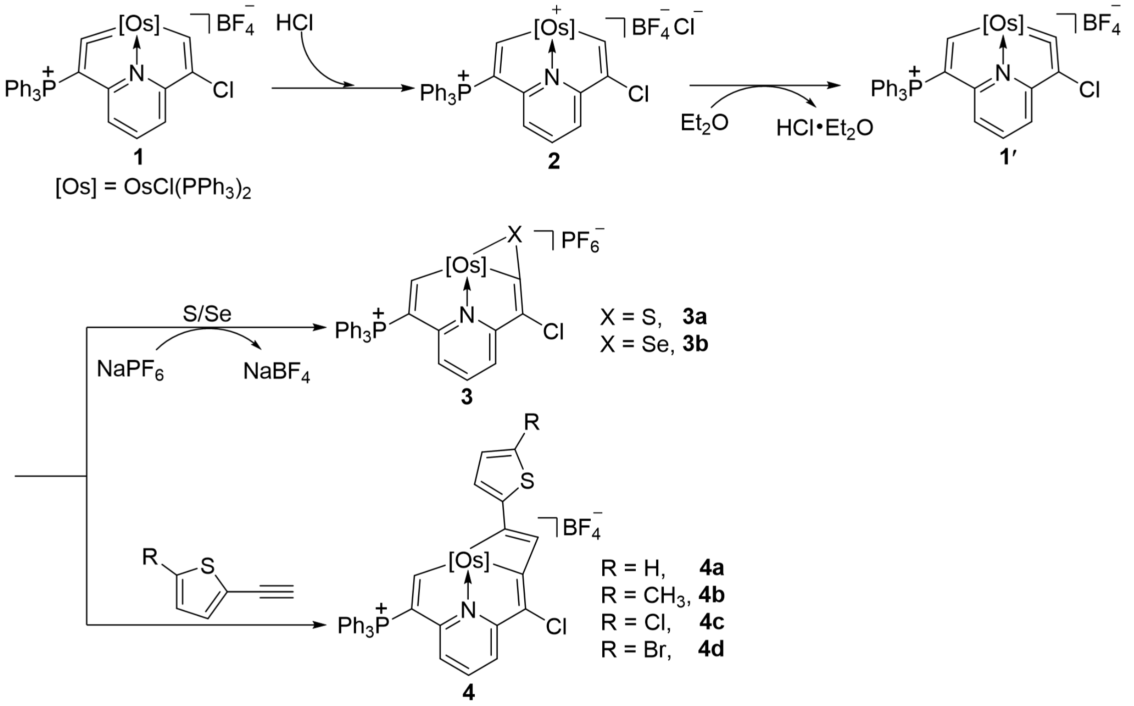

A plausible mechanism for the formation of complexes **3** and **4** is presented in Scheme S4. The addition of protons at osmium vinylidene units of complex **1** gave the complex **2**. Subsequently, the substraction of protons afforded the complex **1'**. Finally, the products **3** were formed via the [2+1] cycloaddition reaction accompanied with the exchange of the counterion by $NaPF_6$ and the products **4** were formed through the [2+2] cycloaddition reaction.

Table S1. The transformation of osmium vinylidene complex 1 to cycloaddition products 3a with different amounts of acid and temperature.

Entry	Equivalent of acids	Temperature (°C)	Yield (%)[a]
1	1 eq. CF_3COOH	40	trace
2	1 eq. $HBF_4{\bullet}Et_2O$	40	16
3	1 eq. $HCl{\bullet}H_2O$	40	10
4	1 eq. $HCl{\bullet}Et_2O$	40	28
5	1 eq. $HCl{\bullet}Et_2O$	50	42
6	1 eq. $HCl{\bullet}Et_2O$	60	53
7	1.5 eq. $HCl{\bullet}Et_2O$	60	45
8	0.5 eq. $HCl{\bullet}Et_2O$	60	65
9	0.3 eq. $HCl{\bullet}Et_2O$	60	50

[a]Reaction conditions: A mixture of compound **1** (15 mg, 0.012 mmol) and S (3.8 mg, 0.12 mmol) were dissolved in CH_2Cl_2 (0.5 mL) by stirring for 48 hours.

2. Experimental Procedures

General Information: All syntheses were carried out under an inert atmosphere (N_2) using standard Schlenk techniques, unless otherwise stated. Solvents were distilled from sodium/benzophenone (diethyl ether) or calcium hydride (dichloromethane) under N_2 prior to use. $OsCl_2(PPh_3)_3$[1] and 2,6-diethynylpyridine (**L**)[2] were prepared according to the previously published procedure. Other reagents were used as received from commercial sources without further purification. Nuclear magnetic resonance (NMR) experiments were performed on a Bruker Ascend 400 spectrometer (^{1}H, 400.1, ^{13}C, 100.6, ^{31}P, 161.9 MHz) and Bruker Ascend III 600 spectrometer (^{1}H, 600.1, ^{13}C, 150.9, ^{31}P, 242.9 MHz) at room temperature. ^{1}H and ^{13}C NMR chemical shifts (δ) are relative to tetramethylsilane, and ^{31}P NMR chemical shifts are relative to 85% H_3PO_4. Two-dimensional is abbreviated as HMBC (heteronuclear multiple bond coherence) and HSQC (heteronuclear single quantum coherence). The absolute values of the coupling constants are given in Hertz (Hz). Multiplicities are abbreviated as singlet (s), doublet (d), triplet (t), quartet (q), multiplet (m), and broad (br). High-resolution mass spectroscopy (HRMS) experiments were conducted on a Thermo Scientific Q Exactive instrument. Elemental analyses were performed on a Vario EL III elemental analyzer.

Synthesis and characterization of 1

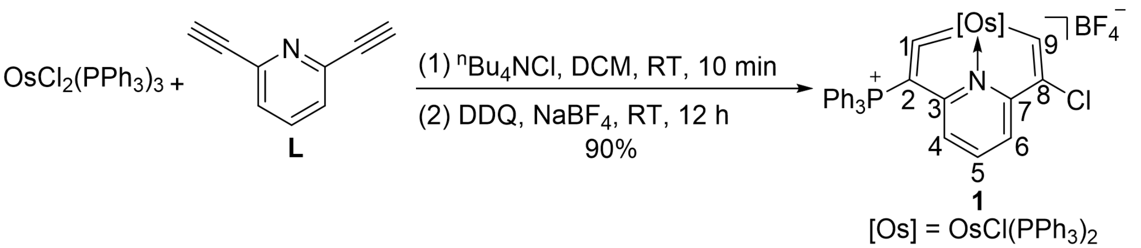

A mixture of $OsCl_2(PPh_3)_3$ (150 mg, 0.14 mmol), ^{n}Bu$_4$NCl (39 mg, 0.14 mmol) and 2,6-diethynylpyridine (18 mg, 0.14 mmol) were dissolved in DCM (5 mL). The reaction mixture was stirred for 10 minutes at room temperature, DDQ (32 mg, 0.14 mmol) and $NaBF_4$ (77 mg, 0.70 mmol) were added subsequently. The reaction mixture was stirred for 12 hours at room temperature to give a brownish-

yellow suspension and the solid suspension was removed through a filter. The filtrate was evaporated to 2 mL recrystallized from CH_2Cl_2/Et_2O 10/1 mixture. The compound **1** was obtained in 90% yield (159 mg) as a yellow solid. ^{1}H-NMR (600.1 MHz, $CDCl_3$, δ ppm): 9.23 (s, 1H, C^9H), 6.93 (t, $J_{H\text{-}H}$ = 7.8 Hz, 1H, C^5H), 6.22 (d, $J_{H\text{-}H}$ = 7.8 Hz, 1H, C^6H), 5.77 (d, $J_{H\text{-}H}$ = 7.8 Hz, 1H, C^4H), 7.83-7.07 (m, 45H, other aromatic protons). ^{31}P-NMR (242.9 MHz, $CDCl_3$, δ ppm): 2.59 (s, C*P*Ph_3), 2.47 (s, Os*P*Ph_3). ^{13}C-NMR (150.9 MHz, $CDCl_3$,δ ppm): 327.96 (td apparent q, $J_{P\text{-}C}$ = 11.5 Hz, $J_{P\text{-}C}$ = 12.1 Hz, C^1), 173.06 (t, $J_{P\text{-}C}$ = 10.5 Hz, C^9), 162.70 (s, C^7), 158.06 (d, $J_{P\text{-}C}$ = 21.1 Hz, C^3), 141.62 (s, C^5), 121.76 (s, C^8), 111.10 (s, C^4), 110.74 (s, C^6), 91.19 (d, $J_{P\text{-}C}$ = 116.2 Hz, C^2), 135.0-118.8 (m, PPh_3). HRMS (ESI): m/z calcd for $[C_{63}H_{49}Cl_2NOsP_3]^+$, 1174.2064; Found: 1174.2045. Anal. Calcd. for $C_{63}H_{49}BCl_2F_4NOsP_3$: C, 60.01; H, 3.92; N, 1.11. Found: C, 59.82; H, 4.22; N, 1.37.

Synthesis and characterization of P1 and P1'

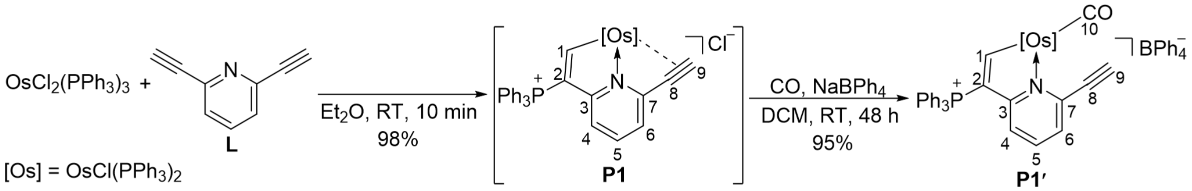

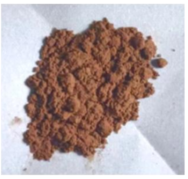

A mixture of $OsCl_2(PPh_3)_3$ (150 mg, 0.14 mmol) and 2,6-diethynylpyridine (22 mg, 0.17 mmol) was dissolved in Et_2O (5 mL). The reaction mixture was stirred for 10 minutes at room temperature to give a brownish-yellow suspension. The solid was collected by filtration, washed with Et_2O (3×2 mL) and then dried under vacuum. The compound **P1** was obtained in 98% yield (161 mg) as a brownish-yellow solid as shown in the picture. A mixture of **P1** (150 mg, 0.13 mmol) and $NaBPh_4$ (133 mg, 0.39 mmol) was dissolved in CH_2Cl_2 (5 mL) and stirred for 48 hours at room temperature in an atmosphere of CO. Then the solid suspension was removed through a filter and the residue was recrystallized from

CH_2Cl_2/Et_2O 1/10 mixture. The compound **P1'** was obtained in 95% yield (184 mg) as a red solid. **P1** exhibits poor solubility in commonly used solvents such as DCM, $CHCl_3$, DCE, MeOH, EtOH, Et_2O, THF, Acetone, CH_3CN, even DMF and DMSO. Furthermore, it demonstrates instability in its solid state after just 3 hours let alone in solution. Consequently, efforts to obtain a comprehensive characterization of **P1**, including NMR experiments and high-resolution mass spectroscopy, have proven unsuccessful. In contrast, **P1'** can be fully characterized without such limitations. ^{1}H-NMR (600.1 MHz, CD_2Cl_2, δ ppm): 11.60 (d, $J_{P\text{-}H}$ = 25.8 Hz, 1H, C^1*H*), 6.65 (t, $J_{H\text{-}H}$ = 7.8 Hz, 1H, C^5*H*), 6.38 (d, $J_{H\text{-}H}$ = 7.8 Hz, 1H, C^4*H*), 6.36 (d, $J_{H\text{-}H}$ = 7.8 Hz, 1H, C^6*H*), 3.32 (s, 1H, C^9*H*), 7.80-6.82 (m, 65H, other aromatic protons). ^{31}P-NMR (242.9 MHz, CD_2Cl_2, δ ppm): 14.92 (s, C*P*Ph$_3$), -7.64 (s, Os*P*Ph$_3$). ^{13}C-NMR (150.9 MHz, CD_2Cl_2, δ ppm): 237.82 (m, C^1), 191.19 (m, C^{10}), 166.81 (d, $J_{P\text{-}C}$ = 33.2 Hz, C^3), 149.75 (s, C^7), 134.26 (s, C^5), 129.96 (s, C^6), 121.02 (s, C^4), 118.24(d, $J_{P\text{-}C}$ = 75.5 Hz, C^2), 89.60 (s, C^9), 83.03 (s, C^8), 136.2-118.4 (m, other aromatic carbons). HRMS (ESI): m/z calcd for $[C_{64}H_{50}ClNOOsP_3]^+$, 1168.2403; Found: 1168.2393. Anal. Calcd. for $C_{88}H_{70}BClNOOsP_3$: C, 71.08; H, 4.75; N, 0.94. Found: C, 70.92; H, 4.92; N, 1.12.

Synthesis and characterization of 2

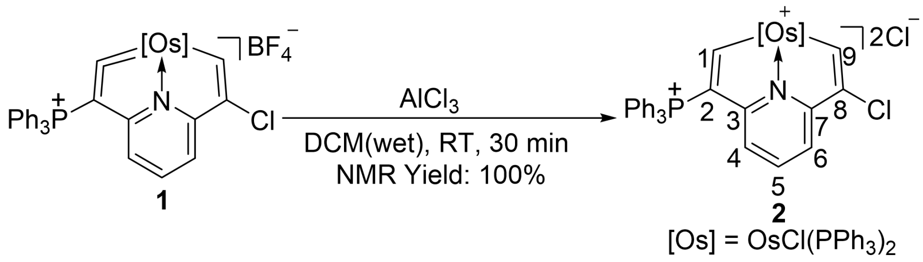

A mixture of **1** (150 mg, 0.12 mmol) and $AlCl_3$ (284 mg, 1.8 mmol) were dissolved in DCM (wet, 2 mL). The reaction mixture was stirred for 30 minutes at room temperature to give an orange suspension, the solid suspension was removed through a filter to give an orange solution of **2** (approximately 100% yield based on ^{1}H and ^{31}P{1H} NMR). ^{1}H-NMR (600.1 MHz, CD_2Cl_2, δ ppm): 10.10 (dd, $J_{P\text{-}H}$ = 11.4 Hz, $J_{H\text{-}H}$ = 4.2 Hz, 1H, C^1*H*), 8.33 (d, $J_{H\text{-}H}$ = 4.0 Hz, 1H, C^9*H*), 7.42 (t, $J_{H\text{-}H}$ = 7.8 Hz, 1H, C^5*H*), 6.10 (d, $J_{H\text{-}H}$ = 7.8 Hz, 1H, C^6*H*), 5.99 (d, $J_{H\text{-}H}$ = 7.8 Hz, 1H, C^4*H*), 8.03-6.95 (m, other aromatic protons plus C^5*H*). ^{31}P-NMR (242.9 MHz, CD_2Cl_2, δ ppm): 16.57 (s, Os*P*Ph$_3$), 10.92 (s, C*P*Ph$_3$). ^{13}C-NMR (150.9 MHz,

CD_2Cl_2, δ ppm): 210.79 (td apparent t, J_{P-C} = 5.3 Hz, C^1), 182.03 (td apparent t, J_{P-C} = 8.3 Hz, C^9), 157.54 (s, C^7), 156.67 (d, J_{P-C} = 19.6 Hz, C^3), 144.51 (s, C^5), 129.72 (s, C^8), 120.27 (d, J_{P-C} = 90.5 Hz, C^2), 119.78 (s, C^6), 119.63 (s, C^4), 137.3-128.7 (m, other aromatic carbons).

Synthesis and characterization of 3a

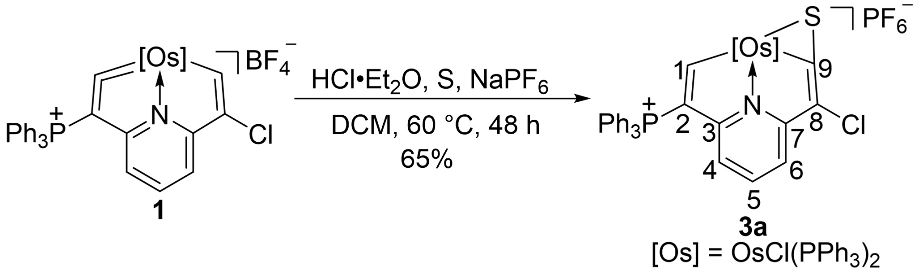

A mixture of compound **1** (150 mg, 0.12 mmol), $HCl·Et_2O$ (30 μL, 2M, 0.06 mmol) and S (38 mg, 1.20 mmol) were dissolved in CH_2Cl_2 (5 mL). After stirring for 48 hours at 60 ℃, The excess S_8 was removed by filtration, and the filtrate was evaporated to 2 mL and then washed with Et_2O (1×20 mL). The compound **3a** was obtained as a brown solid in 65% yield (105 mg). ^{1}H-NMR (600.1 MHz, CD_2Cl_2, δ ppm): 12.18 (d, J_{P-H} = 19.8 Hz, 1H, C^1H), 6.85 (t, J_{H-H} = 7.8 Hz, 1H, C^5H), 6.09 (d, J_{H-H} = 7.8 Hz, 1H, C^4H), 5.99 (d, J_{H-H} = 7.8 Hz, 1H, C^6H), 7.87-6.97 (m, 45H, other aromatic protons). ^{31}P-NMR (242.9 MHz, CD_2Cl_2, δ ppm): 14.22 (s, $CPPh_3$), -20.85 (s, $OsPPh_3$), -144.50 (septet, PF_6). ^{13}C-NMR (150.9 MHz, CD_2Cl_2, δ ppm): 230.23 (t, J_{P-C} = 6.7 Hz,, C^1), 203.52 (br, C^9), 168.66 (s, C^7), 164.74 (d, J_{P-C} = 27.2 Hz, C^3), 135.62 (s, C^5), 113.16 (s, C^6), 109.99 (s, C^4), 109.53 (d, J_{P-C} = 90.5 Hz, C^2), 105.86 (s, C^8), 135.1-117.8 (m, other aromatic carbons). HRMS (ESI): m/z calcd for $[C_{63}H_{49}Cl_2NP_3OsS]^+$, 1206.1785; Found: 1206.1744. Anal. Calcd. for $C_{63}H_{49}F_6Cl_2NP_4OsS$: C, 56.00; H, 3.66; N, 1.04; S, 2.37. Found: C, 56.32; H, 3.85; N, 1.14. ; S, 2.55.

Synthesis and characterization of 3b

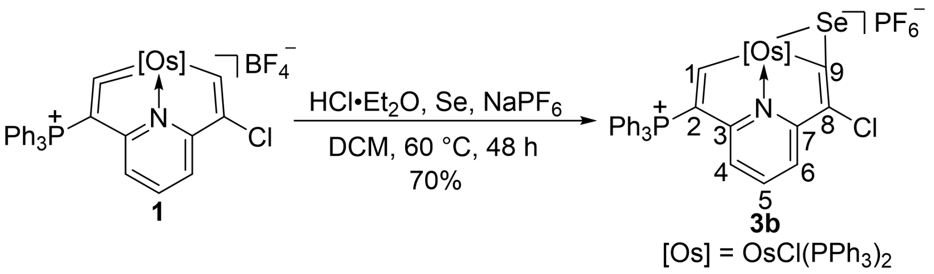

A mixture of compound **1** (150 mg, 0.12 mmol), $HCl{\cdot}Et_2O$ (30 μL, 2M, 0.06 mmol) and Se (95 mg, 1.20 mmol) were dissolved in CH_2Cl_2 (5 mL). After stirring for 48 hours at 60 ℃, The excess Se was removed by filtration, and the filtrate was evaporated to 2 mL and then washed with Et_2O (1×20 mL). The compound **3b** was obtained as a brown solid in 70% yield (117 mg). ^{1}H-NMR (600.1 MHz, CD_2Cl_2, δ ppm): 12.05 (d, $J_{P\text{-}H}$ = 19.8 Hz, 1H, C^1H), 6.82 (t, $J_{H\text{-}H}$ = 7.8 Hz, 1H, C^5H), 6.03 (d, $J_{H\text{-}H}$ = 7.8 Hz, 1H, C^4H), 5.94 (d, $J_{H\text{-}H}$ = 7.8 Hz, 1H, C^6H), 7.88-6.92 (m, 45H, other aromatic protons). ^{31}P-NMR (242.9 MHz, CD_2Cl_2, δ ppm): 13.91 (s, C*P*Ph_3), -25.54 (s, Os*P*Ph_3), -144.49 (septet, *P*F_6). ^{13}C-NMR (150.9 MHz, CD_2Cl_2, δ ppm): 230.31 (t, $J_{P\text{-}C}$ = 6.8 Hz, C^1), 211.98 (t, $J_{P\text{-}C}$ = 3.3 Hz, C^9), 168.57 (s, C^7), 164.96 (d, $J_{P\text{-}C}$ = 27.2 Hz, C^3), 137.31 (s, C^5), 115.92 (s, C^8), 112.69 (s, C^6), 109.57 (d, $J_{P\text{-}C}$ = 90.5 Hz, C^2), 109.69 (s, C^4), 135.5-118.0 (m, other aromatic carbons). HRMS (ESI): m/z calcd for $[C_{63}H_{49}Cl_2NP_3OsSe]^+$, 1254.1229; Found: 1254.1198. Anal. Calcd. for $C_{63}H_{49}F_6Cl_2NP_4OsSe$: C, 54.12; H, 3.53; N, 1.00. Found: C, 53.98; H, 3.61; N, 1.12.

General Procedure for the Synthesis of 4 (Procedure 1)

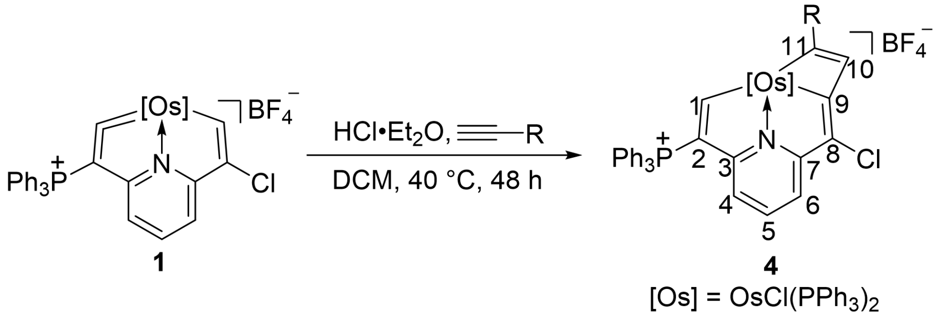

Complexes	**4a**	**4b**	**4c**	**4d**
R	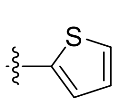	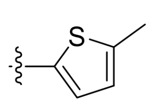	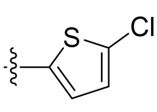	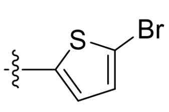
Yield	75%	78%	76%	75%

A mixture of compound **1** (150 mg, 0.12 mmol), HCl·Et_2O (30 μL, 2M, 0.06 mmol) and aromatic terminal alkynes (0.60 mmol) were dissolved in CH_2Cl_2 (5 mL). After stirring for 48 hours at 40 ℃, the solution was evaporated to 2 mL and recrystallized from CH_2Cl_2/Et_2O= 5/1 mixture. The compound **4** was obtained in 75~78% yield as an orange solid.

Synthesis and characterization of 4a

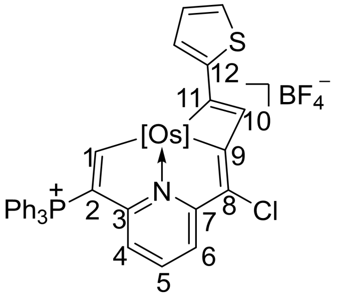

4a
[Os] = $OsCl(PPh_3)_2$

4a was synthesized according to **Procedure 1** and obtained in 75% yield (123 mg). ^{1}H-NMR (600.1 MHz, CD_2Cl_2, δ ppm): 12.88 (d, $J_{P\text{-}H}$ = 21.6 Hz, 1H, C^1H), 7.91 (br, 1H, $C^{10}H$), 6.93 (br, C^5H), 6.44 (d, $J_{H\text{-}H}$ = 7.8 Hz, 1H, C^6H), 6.16 (d, $J_{H\text{-}H}$ = 7.8 Hz, 1H, C^4H), 7.87-6.67 (m, 50H, other aromatic protons plus $C^{10}H$, C^5H). ^{31}P-NMR (242.9 MHz, CD_2Cl_2, δ ppm): 13.45 (s, CPPh$_3$), -25.13 (s, OsPPh$_3$). ^{13}C NMR (150.9 MHz, CD_2Cl_2, δ ppm): 234.10 (m, C^1), 165.70 (d, $J_{P\text{-}C}$ = 30.2 Hz, C^3), 164.56 (s, C^7), 151.14 (t, $J_{P\text{-}C}$ = 5.4 Hz, C^9), 148.83 (t, $J_{P\text{-}C}$ = 8.2 Hz, C^{11}), 147.98 (s, C^{12}), 136.06 (s, C^5), 135.50 (s, C^{10}), 115.74 (s, C^6), 111.75 (d, $J_{P\text{-}C}$ = 87.5 Hz, C^2), 110.92 (s, C^4), 108.95 (s, C^8), 135.5-118.5 (m, PPh_3). HRMS (ESI): m/z calcd for $[C_{69}H_{53}Cl_2NOsP_3S]^+$, 1282.2103; Found: 1282.2085. Anal. Calcd. for $C_{69}H_{53}BCl_2F_4NOsP_3S$: C, 60.53; H, 3.90; N, 1.02; S, 2.34. Found: C, 60.21; H, 4.21; N, 0.92; S, 2.11.

Synthesis and characterization of 4b

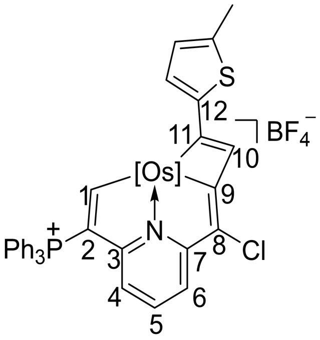

4b
[Os] = $OsCl(PPh_3)_2$

4b was synthesized according to **Procedure 1** and obtained in 78% yield (129 mg) as an yellow solid. ^{1}H-NMR (600.1 MHz, CD_2Cl_2, δ ppm): 12.83 (d, $J_{P\text{-}H}$ = 21.6 Hz, 1H, C^1H), 7.83 (br, $C^{10}H$), 7.06 (br, C^5H), 6.45 (d, $J_{H\text{-}H}$ = 7.2 Hz, 1H, C^6H), 6.16 (d, $J_{H\text{-}H}$ = 7.2 Hz, 1H, C^4H), 0.88 (s, 3H, CH_3), 7.89-6.63 (m, 49H, other

aromatic protons plus $C^{10}H$, $C^{5}H$). ^{31}P-NMR (242.9 MHz, CD_2Cl_2, δ ppm): 13.56 (s, C*P*Ph$_3$), -24.95 (s, Os*P*Ph$_3$). ^{13}C NMR (150.9 MHz, CD_2Cl_2, δ ppm): 233.44 (m, C^1), 165.65 (d, $J_{P\text{-}C}$ = 30.2 Hz, C^3), 164.40 (s, C^7), 150.72 (t, $J_{P\text{-}C}$ = 6.4 Hz, C^9), 149.49 (s, C^{12}), 147.47 (t, $J_{P\text{-}C}$ = 8.5 Hz, C^{11}), 136.29 (s, C^5), 135.61 (s, C^{10}), 115.86 (s, C^6), 111.99 (d, $J_{P\text{-}C}$ = 87.5 Hz, C^2), 111.17 (s, C^4), 109.26 (s, C^8), 135.6-115.7 (m, PPh_3). HRMS (ESI): m/z calcd for $[C_{70}H_{55}Cl_2NOsP_3S]^+$, 1296.2260; Found: 1296.2201. Anal. Calcd. for $C_{70}H_{55}BCl_2F_4NOsP_3S$: C, 60.79; H, 4.01; N, 1.00; S, 2.32. Found: C, 61.02; H, 4.30; N, 1.26; S, 2.65.

Synthesis and characterization of 4c

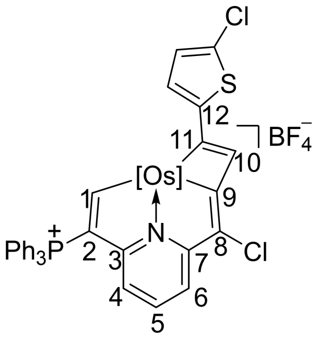

4c

[Os] = $OsCl(PPh_3)_2$

4c was synthesized according to **Procedure 1** and obtained in 76% yield (128 mg) as a yellow solid. ^{1}H-NMR (600.1 MHz, CD_2Cl_2, δ ppm): 12.83 (d, $J_{P\text{-}H}$ = 21.6 Hz, 1H, C^1H), 7.90 (br, $C^{10}H$), 6.95 (br, C^5H), 6.46 (d, $J_{H\text{-}H}$ = 7.2 Hz, 1H, C^6H), 6.17 (d, $J_{H\text{-}H}$ = 7.2 Hz, 1H, C^4H), 7.89-6.48 (m, 49H, other aromatic protons plus $C^{10}H$, C^5H). ^{31}P-NMR (242.9 MHz, CD_2Cl_2, δ ppm): 13.56 (s, C*P*Ph$_3$), -24.94 (s, Os*P*Ph$_3$). ^{13}C NMR (150.9 MHz, CD_2Cl_2, δ ppm): 233.50 (m, C^1), 165.63 (d, $J_{P\text{-}C}$ = 30.2 Hz, C^3), 164.40 (s, C^7), 150.64 (t, $J_{P\text{-}C}$ = 4.5 Hz, C^9), 147.58 (t, $J_{P\text{-}C}$ = 8.6 Hz, C^{11}), 146.62 (s, C^{12}), 136.15 (s, C^5), 135.56 (s, C^{10}), 115.82 (s, C^6), 111.98 (d, $J_{P\text{-}C}$ = 87.5 Hz, C^2), 111.07 (s, C^4), 109.17 (s, C^8), 135.5-118.3 (m, PPh_3). HRMS (ESI): m/z calcd for $[C_{69}H_{52}Cl_3NOsP_3S]^+$, 1316.1714; Found: 1316.1672. Anal. Calcd. for $C_{69}H_{52}BCl_3F_4NOsP_3S$: C, 59.05; H, 3.73; N, 1.00; S, 2.28. Found: C,58.77; H, 3.94; N, 1.17; S, 2.35.

Synthesis and characterization of 4d

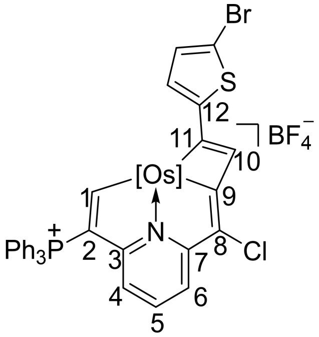

4d

[Os] = $OsCl(PPh_3)_2$

4d was synthesized according to **Procedure 1** and obtained in 75% yield (130 mg) as a yellow solid. ^{1}H-NMR (600.1 MHz, CD_2Cl_2, δ ppm): 12.84 (d, $J_{P\text{-}H}$ = 21.6 Hz, 1H, C^1H), 7.87 (br, $C^{10}H$), 6.93 (br, C^5H), 6.45 (d, $J_{H\text{-}H}$ = 7.8 Hz, 1H, C^6H), 6.16 (d, $J_{H\text{-}H}$ = 7.8 Hz, 1H, C^4H), 7.89-6.62 (m, 49H, other aromatic protons plus $C^{10}H$,

C^5H). ^{31}P-NMR (242.9 MHz, CD_2Cl_2, δ ppm): 13.50 (s, C*P*Ph$_3$), -25.04 (s, Os*P*Ph$_3$). ^{13}C NMR (150.9 MHz, CD_2Cl_2, δ ppm): 233.26 (m, C^1), 165.29 (d, J_{P-C} = 30.2 Hz, C^3), 164.09 (s, C^7), 149.11 (s, C^{12}), 150.43 (t, J_{P-C} = 6.9 Hz, C^9), 147.18 (t, J_{P-C} = 8.2 Hz, C^{11}), 135.67 (s, C^5), 134.90 (s, C^{10}), 115.48 (s, C^6), 111.53 (d, J_{P-C} = 86.9 Hz, C^2), 110.65 (s, C^4), 108.91 (s, C^8), 135.1-111.2 (m, PPh_3). HRMS (ESI): m/z calcd for $[C_{69}H_{52}BrCl_2NOsP_3S]^+$, 1360.1209; Found: 1360.1030. Anal. Calcd. for $C_{69}H_{52}BBrCl_2F_4NOsP_3S$: C, 57.23; H, 3.62; N, 0.97; S, 2.21. Found: C,57.52; H, 3.58; N, 1.22; S, 2.35.

General procedure for the synthesis of 5, isomers (6 and 7) (Procedure 2)

A mixture of $OsCl_2(PPh_3)_3$ (150 mg, 0.14 mmol, 1.0 equiv) and 2,6-diethynylpyridine (22 mg, 0.17 mmol, 1.2 equiv) were dissolved in Et_2O (5 mL). The reaction mixture was stirred for 10 minutes at room temperature, then PR_3 (0.42 mmol, 3.0 equiv) was added and stirred at room temperature for 12 hours. DDQ (1.2~4.0 equiv) and $NaBPh_4$ (342 mg, 0.7 mmol, 5.0 equiv) were added subsequently to give a brown suspension and after 12 hours the solid suspension was removed through a filter. The residue was recrystallized from CH_2Cl_2/MeOH 1/10 mixture. The compounds **5** were obtained in 81~85% yield as yellow solids. The compounds **6** and **7** (isomers) were obtained in 82~85% yield as yellow solids.

(a)

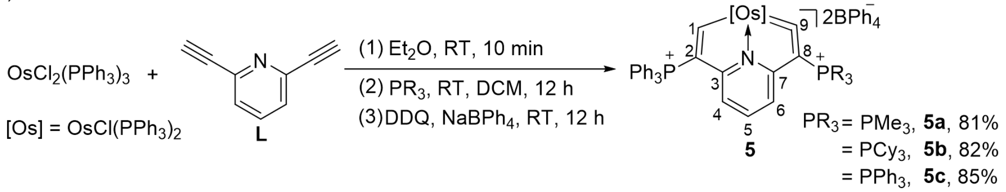

(b)

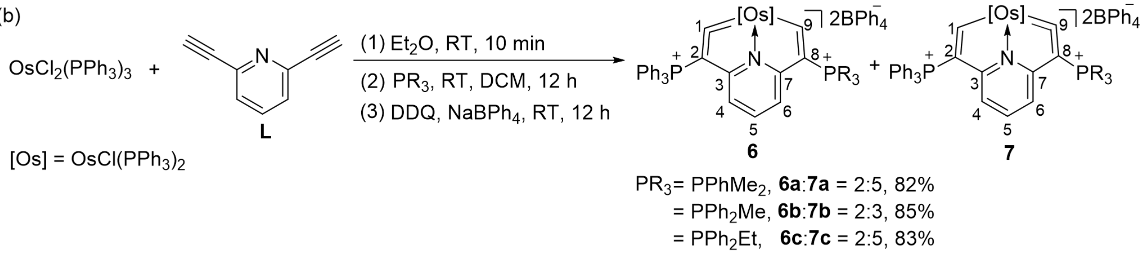

Synthesis and characterization of 5a

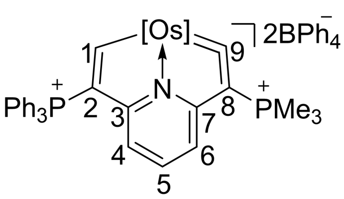

5a
[Os] = $OsCl(PPh_3)_2$

The product was synthesized according to **Procedure 2** with PMe_3 (43 μL, 0.42 mmol) for 12 h and DDQ (38 mg, 0.17 mmol, 1.2 equiv). The compound **5a** was obtained in 81% yield (210 mg). ^{1}H-NMR (600.1 MHz, CD_2Cl_2, δ ppm): 11.35 (d, J_{P-H} = 20.4 Hz, 1H, C^1H), 6.72 (t, J_{H-H} = 7.8 Hz, 1H, C^5H), 6.09 (d, J_{H-H} = 7.8 Hz, 1H, C^4H), 5.47 (d, J_{H-H} = 7.8 Hz, 1H, C^6H), 0.80 (d, J_{P-H} = 13.8 Hz, 9H, CH_3), 7.86-6.30 (m, 86 H, other aromatic protons plus C^5H). ^{31}P-NMR (242.9 MHz, CD_2Cl_2, δ ppm): 14.15 (s, C^2PPh_3), 6.34 (s, $OsPPh_3$), 1.02 (s, C^8PMe_3). ^{13}C-NMR (150.9 MHz, $CD_2Cl_2/(CD_3)_2SO$ = 1/1, δ ppm): 330.68 (br, C^9), 222.52 (br, C^1), 163.60 (br, C^3), 158.62 (d, J_{P-C} = 21.1 Hz, C^7), 141.60 (s, C^5), 115.43 (d, J_{P-C} = 86.0 Hz, C^2), 112.21 (s, C^6), 111.36 (s, C^4), 101.32 (d, J_{P-C} = 105.6 Hz, C^8), 8.50 (d, J_{P-C} = 57.3 Hz, CH_3), 135.3-116.0 (m, other aromatic carbons). HRMS (ESI): m/z calcd for $[C_{66}H_{58}ClNP_4Os]^{2+}$, 607.6406; Found: 607.6407. Anal. Calcd. for $C_{114}H_{98}B_2ClNP_4Os$: C, 73.88; H, 5.33; N, 0.76. Found: C, 74.08; H, 5.48; N, 0.94.

Synthesis and characterization of 5b

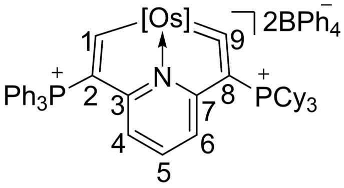

5b
[Os] = $OsCl(PPh_3)_2$

The product was synthesized according to **Procedure 2** with PCy_3 (118 mg, 0.42 mmol) for 12 h and DDQ (128 mg, 0.56 mmol, 4.0 equiv). The compound **5b** was obtained in 82% yield (236 mg). ^{1}H-NMR (600.1 MHz, CD_2Cl_2, δ ppm): 11.20 (d, J_{P-H} = 20.4 Hz, 1H, C^1H), 7.38 (br, C^5H), 6.74 (d, J_{H-H} = 7.8 Hz, 1H, C^6H), 6.31 (d, J_{H-H} = 7.8 Hz, 1H, C^4H), 7.86-6.63 (m, 87H, other aromatic protons plus C^5H, C^6H), 1.75-1.13 (m, 33H, Cy). ^{31}P-NMR (242.9 MHz, CD_2Cl_2, δ ppm): 22.45 (s, C^8PCy_3), 14.17 (s, C^2PPh_3), 1.36 (s, $OsPPh_3$). ^{13}C-NMR (150.9 MHz, CD_2Cl_2, δ ppm): 336.34 (m, C^9), 223.85 (m, C^1), 165.35 (d, J_{P-C} = 27.2 Hz, C^3), 161.87 (d, J_{P-C} = 18.1 Hz, C^7), 143.94 (s, C^5), 119.24 (d, J_{P-C} = 92.0 Hz, C^2), 113.48 (s, C^6), 112.85 (s, C^4), 94.82 (d, J_{P-C} = 90.5 Hz, C^8), 141.4-110.9 (m, other aromatic carbons), 35.6-14.2 (m, Cy). HRMS (ESI): m/z calcd for $[C_{81}H_{82}ClNP_4Os]^{2+}$, 709.7345; Found: 709.7318.

Anal. Calcd. for $C_{129}H_{122}B_2ClNP_4Os$: C, 75.30; H, 5.98; N, 0.68. Found: C, 75.52; H, 6.25; N, 0.74.

Synthesis and characterization of 5c

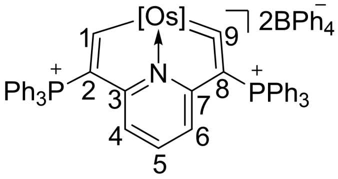

5c

[Os] = $OsCl(PPh_3)_2$

The product was synthesized according to **Procedure 2** with PPh_3 (110 mg, 0.42 mmol) for 12 h and DDQ (96 mg, 0.42 mmol, 3.0 equiv). The compound **5c** was obtained in 85% yield (243 mg). ^{1}H-NMR (600.1 MHz, CD_2Cl_2, δ ppm): 11.29 (d, $J_{P\text{-}H}$ = 20.4 Hz, 1H, C^1H), 6.90 (br, C^5H), 6.23 (d, $J_{H\text{-}H}$ = 7.8 Hz, 1H, C^4H), 5.99 (d, $J_{H\text{-}H}$ = 7.8 Hz, 1H, C^6H), 7.86-6.39 (m, 101H, other aromatic protons plus C^5H). ^{31}P-NMR (242.9 MHz, CD_2Cl_2, δ ppm): 14.67 (s, C^2PPh_3), 3.39 (s, C^8PPh_3), 3.15 (s, $OsPPh_3$). ^{13}C-NMR (150.9 MHz, CD_2Cl_2, δ ppm): 335.08 (m, C^9), 223.39 (m, C^1), 164.80 (d, $J_{P\text{-}C}$ = 27.2 Hz, C^3), 160.84 (d, $J_{P\text{-}C}$ = 21.1 Hz, C^7), 143.02 (s, C^5), 117.50 (d, $J_{P\text{-}C}$ = 84.5 Hz, C^2), 113.02 (s, C^6), 112.98 (s, C^4), 97.87 (d, $J_{P\text{-}C}$ = 113.2 Hz, C^8), 136.3-118.3 (m, other aromatic carbons). HRMS (ESI): m/z calcd for $[C_{81}H_{64}ClNP_4Os]^{2+}$, 700.6641; Found: 700.6639. Anal. Calcd. for $C_{129}H_{104}B_2ClNP_4Os$: C, 75.97; H, 5.14; N, 0.69. Found: C, 76.12; H, 5.25; N, 0.84.

Synthesis and characterization of isomers (6a and 7a)

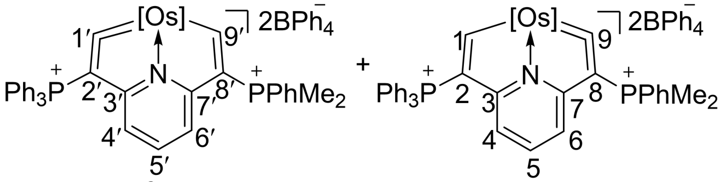

6a **7a**

[Os] = $OsCl(PPh_3)_2$ **6a**:**7a** = 2:5

The product was synthesized according to **Procedure 2** with $PPhMe_2$ (60 μL, 0.42 mmol) for 12 h and DDQ (54 mg, 0.24 mmol, 1.7 equiv). The compound **6a** and **7a** was obtained in 82% yield (220 mg). ^{1}H-NMR (600.1 MHz, CD_2Cl_2, δ ppm): 11.32 (d, $J_{P\text{-}H}$ = 20.4 Hz, 5H, C^1H), 10.98 (d, $J_{P\text{-}H}$ = 20.4 Hz, 2H, $C^{9'}H$), 6.80-6.75 (m, 7H, C^5H plus $C^{5'}H$), 6.12 (d, $J_{H\text{-}H}$ = 7.8 Hz, 5H, C^4H), 5.93 (d, $J_{H\text{-}H}$ = 7.8 Hz, 2H, $C^{6'}H$), 5.81 (d, $J_{H\text{-}H}$ = 7.8 Hz, 2H, $C^{4'}H$), 5.65 (d, $J_{H\text{-}H}$ = 7.8 Hz, 5H, C^6H), 1.20 (d, $J_{P\text{-}H}$ = 12.6 Hz, 12H, $C'H_3$), 1.04 (d, $J_{P\text{-}H}$ = 13.2 Hz, 30H, CH_3), 7.84-6.33 (m, 637H, other aromatic protons plus C^5H, $C^{5'}H$). ^{31}P-NMR (242.9 MHz, CD_2Cl_2, δ ppm): 14.20 (s, C^2PPh_3), 10.69 (s,

$C^{8'}$*P*$PhMe_2$), 6.19 (s, Os*P*Ph_3 of **7a**), 3.94 (s, Os*P*Ph_3 of **6a**), 3.18 (s, $C^{2'}$*P*Ph_3), -0.82 (s, C^8*P*$PhMe_2$). ^{13}C-NMR (150.9 MHz, CD_2Cl_2, δ ppm): 333.10 (m, $C^{1'}$), 332.92 (m, C^9), 224.40 (m, C^1), 215.91 (br, $C^{9'}$), 164.64 (d, $J_{P\text{-}C}$ = 27.2 Hz, C^3), 162.80 (d, $J_{P\text{-}C}$ = 27.2 Hz, $C^{7'}$), 160.63 (d, $J_{P\text{-}C}$ = 21.1 Hz, $C^{3'}$), 159.27 (d, $J_{P\text{-}C}$ = 22.6 Hz, C^7), 142.84 (s, C^5), 142.72 (s, $C^{5'}$), 119.62 (d, $J_{P\text{-}C}$ = 86.0 Hz, $C^{8'}$), 116.40 (d, $J_{P\text{-}C}$ = 86.0 Hz, C^2), 112.72 (s, $C^{4'}$), 112.18 (s, C^6), 112.10 (s, C^4), 111.22 (s, $C^{6'}$), 100.94 (d, $J_{P\text{-}C}$ = 110.2 Hz, C^8), 97.04 (d, $J_{P\text{-}C}$ = 116.2 Hz, $C^{2'}$), 8.71 (d, $J_{P\text{-}C}$ = 57.3 Hz, *C*H_3), 8.41 (d, $J_{P\text{-}C}$ = 57.3 Hz, *C'*H_3), 136.3-116.1 (m, other aromatic carbons). HRMS (ESI): m/z calcd for $[C_{71}H_{60}ClNP_4Os]^{2+}$, 638.6484; Found: 638.6465. Anal. Calcd. for $C_{119}H_{100}B_2ClNP_4Os$: C, 74.63; H, 5.26; N, 0.73. Found: C, 74.48; H, 5.44; N, 0.85.

Synthesis and characterization of isomers (6b and 7b)

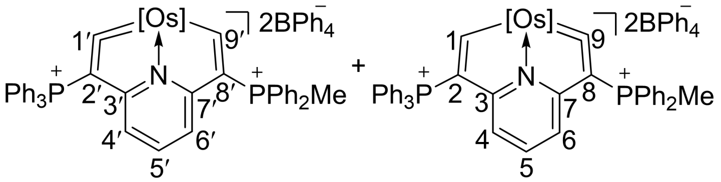

6b **7b**

[Os] = $OsCl(PPh_3)_2$ **6b**:**7b**= 2:3

The product was synthesized according to **Procedure 2** with PPh_2Me (78 μL, 0.42 mmol) for 12 h and DDQ (70 mg, 0.31 mmol, 2.2 equiv). The compound **6b** and **7b** was obtained in 85% yield (235 mg). ^{1}H-NMR (600.1 MHz, CD_2Cl_2, δ ppm): 11.23 (d, $J_{P\text{-}H}$ = 21.6 Hz, 3H, C^1*H*), 10.89 (d, $J_{P\text{-}H}$ = 19.2 Hz, 2H, $C^{9'}$*H*), 6.88-6.82 (m, 5H, C^5*H* plus $C^{5'}$*H*), 6.20 (d, $J_{H\text{-}H}$ = 7.8 Hz, 3H, C^4*H*), 6.09 (d, $J_{H\text{-}H}$ = 7.8 Hz, 2H, $C^{6'}$*H*), 5.89 (d, $J_{H\text{-}H}$ = 7.8 Hz, 2H, $C^{4'}$*H*), 5.76 (d, $J_{H\text{-}H}$ = 7.8 Hz, 3H, C^6*H*), 1.70 (d, $J_{P\text{-}H}$ = 12.6 Hz, 6H, C'*H_3*), 1.33 (d, $J_{P\text{-}H}$ = 13.2 Hz, 9H, C*H_3*), 7.84-6.77 (m, 480H, other aromatic protons plus C^5*H*, $C^{5'}$*H*). ^{31}P-NMR (242.9 MHz, CD_2Cl_2, δ ppm): 14.22 (s, C^2*P*Ph_3), 12.26 (s, $C^{8'}$*P*Ph_2Me), 5.24 (s, Os*P*Ph_3 of **7b**), 3.57 (s, Os*P*Ph_3 of **6b**), 3.37 (s, $C^{2'}$*P*Ph_3), -0.28 (s, C^8*P*Ph_2Me). ^{13}C-NMR (150.9 MHz, CD_2Cl_2, δ ppm): 333.78 (m, C^9, $C^{1'}$), 224.41 (m, C^1), 219.56 (m, $C^{9'}$), 164.86 (d, $J_{P\text{-}C}$ = 27.2 Hz, C^3), 163.30 (d, $J_{P\text{-}C}$ = 27.2 Hz, $C^{7'}$), 160.55 (d, $J_{P\text{-}C}$ = 21.1 Hz, $C^{3'}$), 160.04 (d, $J_{P\text{-}C}$ = 22.6 Hz, C^7), 143.03 (s, C^5), 142.86 (s, $C^{5'}$), 119.09 (d, $J_{P\text{-}C}$ = 86.1 Hz, $C^{8'}$), 116.68 (d, $J_{P\text{-}C}$ = 86.0 Hz, C^2), 112.86 (s, C^6), 112.78 (s, $C^{4'}$), 112.49 (s, C^4), 112.13 (s, $C^{6'}$), 99.16 (d, $J_{P\text{-}C}$ =

113.2 Hz, C^8), 97.21 (d, J_{P-C} = 113.2 Hz, $C^{2'}$), 8.12 (d, J_{P-C} = 58.9 Hz, CH_3), 7.67 (d, J_{P-C} = 58.9 Hz, $C'H_3$), 136.3-114.1 (m, other aromatic carbons). HRMS (ESI): m/z calcd for $[C_{76}H_{62}ClNP_4Os]^{2+}$, 669.6563; Found: 669.6546. Anal. Calcd. for $C_{124}H_{102}B_2ClNP_4Os$: C, 75.32; H, 5.20; N, 0.71. Found: C, 75.64; H, 5.34; N, 0.96.

Synthesis and characterization of isomers (6c and 7c)

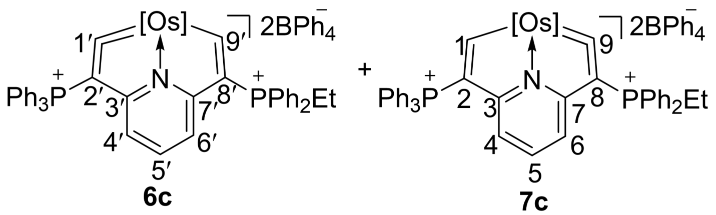

6c **7c**

[Os] = OsCl(PPh3)2 **6c**:**7c** = 2:5

The product was synthesized according to **Procedure 2** with PPh_2Et (86 μL, 0.42 mmol) for 12 h and DDQ (54 mg, 0.24 mmol, 1.7 equiv). The compound **6c** and **7c** was obtained in 83% yield (231 mg). ^{1}H-NMR (600.1 MHz, CD_2Cl_2, δ ppm): 11.33 (d, J_{P-H} = 18.0 Hz, 2H, $C^{9'}H$), 11.27 (d, J_{P-H} = 19.2 Hz, 5H, C^1H), 6.69 (t, J_{H-H} = 7.8 Hz, 2H, $C^{5'}H$), 6.66 (t, J_{H-H} = 7.8 Hz, 5H, C^5H), 6.16 (d, J_{H-H} = 7.8 Hz, 2H, $C^{6'}H$), 6.10 (d, J_{H-H} = 7.8 Hz, 5H, C^4H), 5.87 (d, J_{H-H} = 7.8 Hz, 5H, C^6H), 5.83 (d, J_{H-H} = 7.8 Hz, 2H, $C^{4'}H$), 2.23-2.12 (m, 14H, CH_2), 0.91-0.83 (m, 21H, CH_3), 7.84-6.31 (m, 672H, other aromatic protons plus C^5H, $C^{5'}H$). ^{31}P-NMR (242.9 MHz, CD_2Cl_2, δ ppm): 16.78 (s, $C^{8'}PPh_2Et$), 14.44 (s, C^2PPh_3), 7.38 (s, C^8PPh_2Et), 4.30 (s, $OsPPh_3$ of **7c**), 3.23 (s, $OsPPh_3$ of **6c**+$C^{2'}PPh_3$). ^{13}C-NMR (150.9 MHz, CD_2Cl_2, δ ppm): 334.09 (m, $C^{1'}$+C^9), 223.52 (m, C^1), 219.47 (m, $C^{9'}$), 164.68 (d, J_{P-C} = 24.2 Hz, C^3), 163.80 (d, J_{P-C} = 27.2 Hz, $C^{7'}$), 160.75 (d, J_{P-C} = 19.6 Hz, $C^{3'}$), 160.04 (d, J_{P-C} = 19.6 Hz, C^7), 143.20 (s, $C^{5'}$), 143.09 (s, C^5), 119.31 (d, J_{P-C} = 92.0 Hz, C^2), 118.04 (d, J_{P-C} = 92.3 Hz, $C^{8'}$), 113.22 (s, C^6), 112.98 (s, $C^{4'}$), 112.79 (s, C^4), 112.67 (s, $C^{6'}$), 98.25 (d, J_{P-C} = 108.7 Hz, C^8), 97.46 (d, J_{P-C} = 107.2 Hz, $C^{2'}$), 18.30 (d, J_{P-C} = 57.3 Hz, CH_2), 17.46 (d, J_{P-C} = 54.3 Hz, $C'H_2$), 7.54 (d, J_{P-C} = 4.5 Hz, $C'H_3$), 7.42 (d, J_{P-C} = 4.5 Hz, CH_3), 140.5-115.7 (m, other aromatic carbons). HRMS (ESI): m/z calcd for $[C_{77}H_{64}ClNP_4Os]^{2+}$, 676.6641; Found: 676.6632. Anal. Calcd. for $C_{125}H_{104}B_2ClNP_4Os$: C, 75.39; H, 5.26; N, 0.70. Found: C, 75.58; H, 5.52; N, 0.82.

General procedure for the synthesis of 8 and 7 (Procedure 3)

A mixture of **6** and **7** (150 mg, 1.0 equiv) and $AlCl_3$ (15 equiv) were dissolved in DCM (wet, 2 mL). The reaction mixture was stirred for 30 minutes at room temperature to give an orange suspension, the solid suspension was removed through a filter to give an orange solution of **8** (approximately 100% yield based on ^{1}H and ^{31}P{1H} NMR), then added $NaBPh_4$ (15 equiv) to the orange solution and stirred for 1 hour at room temperature to give a brown suspension, the solid suspension was removed through a filter to take excess sodium off. Et_2O (20 mL) was added into the residue and stirred for 10 minutes. The compound **7** was obtained in 92~95% yield as a yellow solid by filtration.

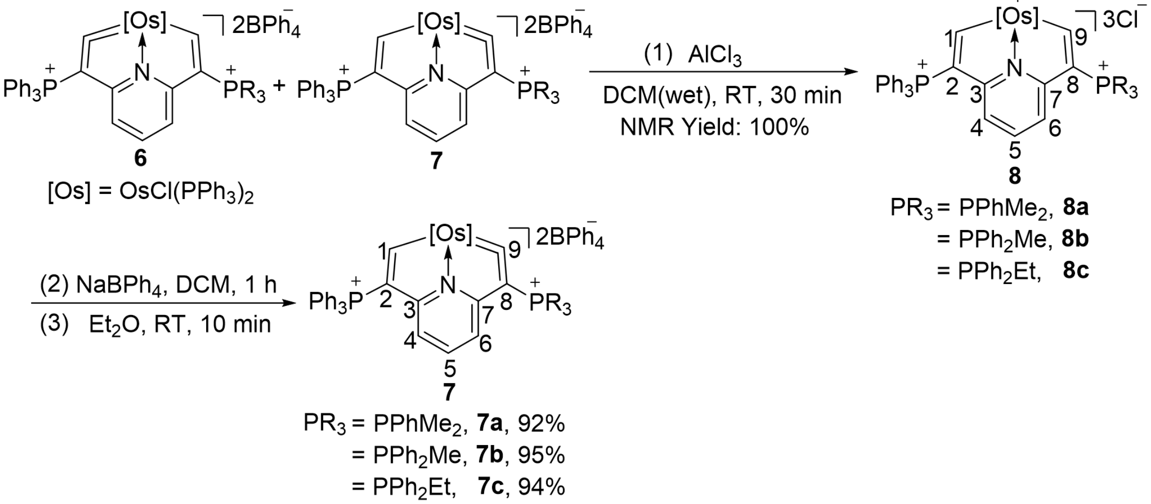

Synthesis and characterization of 8a and 7a

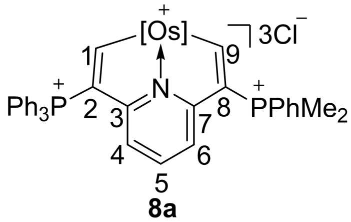

8a

[Os] = $OsCl(PPh_3)_2$

The product **8a** was synthesized according to **Procedure 3** with a mixture of **6a** and **7a** (150 mg, 0.078 mmol) and $AlCl_3$ (156 mg, 1.17 mmol). ^{1}H-NMR (600.1 MHz, CD_2Cl_2, δ ppm): 9.00 (dd, $J_{P\text{-}H}$ = 11.1 Hz, $J_{H\text{-}H}$ = 5.1 Hz, 1H, C^1H), 8.64 (dd, $J_{P\text{-}H}$ = 11.4 Hz, $J_{H\text{-}H}$ = 4.8 Hz, 1H, C^9H), 7.78 (t, $J_{H\text{-}H}$ = 7.8 Hz, 1H, C^5H), 6.56 (d, $J_{H\text{-}H}$ = 7.8 Hz, 1H, C^6H), 6.45 (d, $J_{H\text{-}H}$ = 7.8 Hz, 1H, C^4H), 1.94 (d, $J_{P\text{-}H}$ = 12.6 Hz, 6H, CH_3), 8.11-6.49 (m, other aromatic protons plus C^5H, C^6H, C^4H). ^{31}P-NMR (242.9 MHz, CD_2Cl_2, δ ppm): 22.83 (s, Os*P*Ph_3), 12.13 (s, C^2*P*Ph_3), 11.75 (s, C^8*P*$PhMe_2$). ^{13}C-NMR (150.9 MHz, CD_2Cl_2, δ ppm): 212.76 (td apparent t, $J_{P\text{-}C}$ = 6.1 Hz, C^1),

209.49 (td apparent t, $J_{P\text{-}C}$ = 6.8 Hz, C^9), 159.14 (d, $J_{P\text{-}C}$ = 19.6 Hz, C^3), 157.33 (d, $J_{P\text{-}C}$ = 19.6 Hz, C^7), 146.83 (s, C^5), 124.28 (d, $J_{P\text{-}C}$ = 86.0 Hz, C^8), 121.75 (d, $J_{P\text{-}C}$ = 90.5 Hz, C^2), 118.94 (s, C^4), 117.85 (s, C^6), 8.92 (d, $J_{P\text{-}C}$ = 57.3 Hz, CH_3), 146.5-114.2 (m, other aromatic carbons).

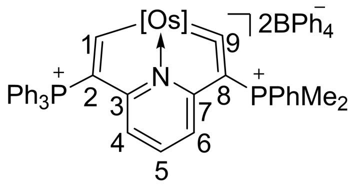

7a

[Os] = $OsCl(PPh_3)_2$

The compound **7a** was obtained in 92% yield (137 mg) according to **Procedure 3**. ^{1}H-NMR (600.1 MHz, CD_2Cl_2, δ ppm): 11.31 (d, $J_{P\text{-}H}$ = 20.4 Hz, 1H, C^1H), 7.08 (t, $J_{H\text{-}H}$ = 7.8 Hz, 1H, C^5H), 6.22 (d, $J_{H\text{-}H}$ = 7.8 Hz, 1H, C^4H), 6.07 (d, $J_{H\text{-}H}$ = 7.8 Hz, 1H, C^6H), 1.45 (d, $J_{P\text{-}H}$ = 13.2 Hz, 6H, CH_3), 7.95-6.38 (m, 91 H, other aromatic protons plus C^5H). ^{31}P-NMR (242.9 MHz, CD_2Cl_2, δ ppm): 14.19 (s, C^2PPh_3), 6.11 (s, $OsPPh_3$), -0.80 (s, C^8PPhMe). ^{13}C-NMR (150.9 MHz, CD_2Cl_2, δ ppm): 332.96 (m, C^9), 224.29 (m, C^1), 164.65 (d, $J_{P\text{-}C}$ = 28.7 Hz, C^3), 159.38 (d, $J_{P\text{-}C}$ = 22.6 Hz, C^7), 143.39 (s, C^5), 116.66 (d, $J_{P\text{-}C}$ = 87.5 Hz, C^2), 112.82 (s, C^6), 112.42 (s, C^4), 101.10 (d, $J_{P\text{-}C}$ = 111.7 Hz, C^8), 9.49 (d, $J_{P\text{-}C}$ = 58.9 Hz, CH_3), 136.3-117.5 (m, other aromatic carbons). HRMS (ESI): m/z calcd for $[C_{71}H_{60}ClNP_4Os]^{2+}$, 638.6484; Found: 638.6477. Anal. Calcd. for $C_{119}H_{100}B_2ClNP_4Os$: C, 74.63; H, 5.26; N, 0.73. Found: C, 74.48; H, 5.30; N, 0.62.

Synthesis and characterization of 8b and 7b

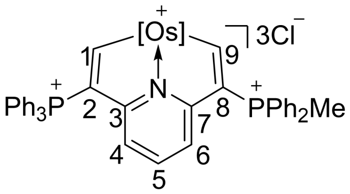

8b

[Os] = $OsCl(PPh_3)_2$

The product **8b** was synthesized according to **Procedure 3** with a mixture of **6b** and **7b** (150 mg, 0.076 mmol) and $AlCl_3$ (152mg, 1.14 mmol). ^{1}H-NMR (400.1 MHz, CD_2Cl_2, δ ppm): 9.08 (dd, $J_{P\text{-}H}$ = 11.2 Hz, $J_{H\text{-}H}$ = 4.8 Hz, 1H, C^1H), 8.67 (dd, $J_{P\text{-}H}$ = 11.2 Hz, $J_{H\text{-}H}$ = 4.8 Hz, 1H, C^9H), 7.88 (t, $J_{H\text{-}H}$ = 8.0 Hz, 1H, C^5H), 6.61 (d, $J_{H\text{-}H}$ = 8.0 Hz, 1H, C^4H), 6.57 (d, $J_{H\text{-}H}$ = 8.0 Hz, 1H, C^6H), 2.25 (d, $J_{P\text{-}H}$ = 12.8 Hz, 3H, CH_3), 8.13-6.56 (m, other aromatic protons plus C^5H, C^6H, C^4H). ^{31}P-NMR (161.9 MHz, CD_2Cl_2, δ ppm): 21.99 (s, $OsPPh_3$), 12.14 (s, C^2PPh_3), 10.75 (s, C^8PPh_2Me). ^{13}C-NMR (100.6 MHz, CD_2Cl_2, δ ppm): 213.34 (td apparent t, $J_{P\text{-}C}$ = 6.4 Hz, C^1), 211.84 (td apparent t, $J_{P\text{-}C}$ =5.6 Hz, C^9), 159.40 (d, $J_{P\text{-}C}$ = 19.1 Hz, C^3), 158.24 (d,

$J_{P\text{-}C}$ = 20.1 Hz, C^7), 147.44 (s, C^5), 123.92 (d, $J_{P\text{-}C}$ = 89.5 Hz, C^8), 122.36 (d, $J_{P\text{-}C}$ = 94.6 Hz, C^2), 119.58 (s, C^4), 119.07 (s, C^6), 8.10 (d, $J_{P\text{-}C}$ = 58.3 Hz, CH_3), 137.7-122.5 (m, other aromatic carbons).

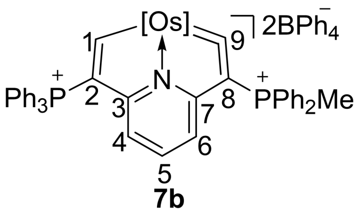

7b

[Os] = $OsCl(PPh_3)_2$

The compound **7b** was obtained in 95% yield (143 mg) according to **Procedure 3**. ^{1}H-NMR (600.1 MHz, CD_2Cl_2, δ ppm): 11.23 (d, $J_{P\text{-}H}$ = 20.4 Hz, 1H, C^1H), 6.71 (t, $J_{H\text{-}H}$ = 7.8 Hz, 1H, C^5H), 6.17 (d, $J_{H\text{-}H}$ = 7.8 Hz, 1H, C^4H), 5.65 (d, $J_{H\text{-}H}$ = 7.8 Hz, 1H, C^6H), 1.18 (d, $J_{P\text{-}H}$ = 12.6 Hz, 3H, CH_3), 7.83-6.37 (m, 96 H, other aromatic protons plus C^5H). ^{31}P-NMR (242.9 MHz, CD_2Cl_2, δ ppm): 14.22 (s, C^2PPh_3), 5.25 (s, $OsPPh_3$), -0.29 (s, C^8PPh_2Me). ^{13}C-NMR (150.9 MHz, CD_2Cl_2, δ ppm): 333.92 (m, C^9), 224.61 (m, C^1), 164.92 (d, $J_{P\text{-}C}$ = 27.2 Hz, C^3), 160.08 (d, $J_{P\text{-}C}$ = 21.1Hz, C^7), 142.93 (s, C^5), 116.66 (d, $J_{P\text{-}C}$ = 86.0 Hz, C^2), 112.79 (s, C^6), 112.46 (s, C^4), 99.12 (d, $J_{P\text{-}C}$ = 113.2 Hz, C^8), 8.02 (d, $J_{P\text{-}C}$ = 58.9 Hz, CH_3), 136.3-117.4 (m, other aromatic carbons). HRMS (ESI): m/z calcd for $[C_{76}H_{62}ClNP_4Os]^{2+}$, 669.6563; Found: 669.6563. Anal. Calcd. for $C_{124}H_{102}B_2ClNP_4Os$: C, 75.32; H, 5.20; N, 0.71. Found: C, 75.45; H, 5.57; N, 0.82.

Synthesis and characterization of 8c and 7c

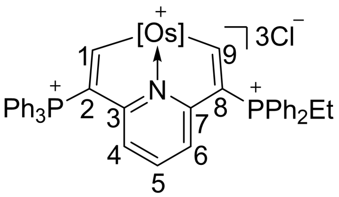

8c

[Os] = $OsCl(PPh_3)_2$

The product **8c** was synthesized according to **Procedure 3** with a mixture of **6c and 7c** (150 mg, 0.075 mmol) and $AlCl_3$ (151 mg, 1.13 mmol). ^{1}H-NMR (600.1 MHz, CD_2Cl_2, δ ppm): 9.03 (dd, $J_{P\text{-}H}$ = 11.1 Hz, $J_{H\text{-}H}$ = 5.4 Hz, 1H, C^1H), 8.89 (dd, $J_{P\text{-}H}$ = 10.8 Hz, $J_{H\text{-}H}$ = 5.4 Hz, 1H, C^9H), 7.82 (t, $J_{H\text{-}H}$ = 7.8 Hz, 1H, C^5H), 6.64 (d, $J_{H\text{-}H}$ = 7.8 Hz, 1H, C^6H), 6.49 (d, $J_{H\text{-}H}$ = 7.8 Hz, 1H, C^4H), 2.52-2.48 (m, 2H, CH_2), 1.20-1.12 (m, 3H, CH_3), 8.06-6.50 (m, other aromatic protons plus C^5H, C^6H, C^4H). ^{31}P-NMR (242.9 MHz, CD_2Cl_2, δ ppm): 20.77 (s, $OsPPh_3$), 16.89 (s, C^8PPh_2Et), 12.04 (s, C^2PPh_3). ^{13}C-NMR (150.9 MHz, CD_2Cl_2, δ ppm): 213.26 (td apparent t, $J_{P\text{-}C}$ = 7.5 Hz, C^1), 211.26 (td apparent t, $J_{P\text{-}C}$ = 7.5 Hz, C^9), 159.47 (d, $J_{P\text{-}C}$ = 18.1 Hz, C^3), 158.70 (d, $J_{P\text{-}C}$ = 19.6 Hz, C^7), 147.40 (s, C^5), 123.93 (d, $J_{P\text{-}C}$ = 84.5 Hz, C^8), 122.35 (d, $J_{P\text{-}C}$

= 89.0 Hz, C^2), 119.62 (s, C^4), 119.54 (s, C^6), 17.34 (d, $J_{P\text{-}C}$ = 52.8 Hz, CH_2), 7.54 (d, $J_{P\text{-}C}$ = 4.5 Hz, CH_3), 137.7-112.9 (m, other aromatic carbons).

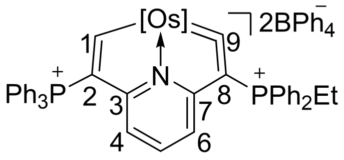

7c

[Os] = $OsCl(PPh_3)_2$

The compound **7c** was obtained in 94% yield (140 mg) according to **Procedure 3**. ^{1}H-NMR (600.1 MHz, CD_2Cl_2, δ ppm): 11.27 (d, $J_{P\text{-}H}$ = 19.2 Hz, 1H, C^1H), 6.63 (t, $J_{H\text{-}H}$ = 7.8 Hz, 1H, C^5H), 6.09 (d, $J_{H\text{-}H}$ = 7.8 Hz, 1H, C^4H), 5.85 (d, $J_{H\text{-}H}$ = 7.8 Hz, 1H, C^6H), 2.20-2.18 (m, 2H, CH_2), 0.88-0.82 (m, 3H, CH_3), 7.84-6.34 (m, 96 H, other aromatic protons plus C^5H). ^{31}P-NMR (242.9 MHz, CD_2Cl_2, δ ppm): 14.44 (s, C^2PPh_3), 7.37 (s, C^8PPh_2Et), 4.29 (s, $OsPPh_3$). ^{13}C-NMR (150.9 MHz, CD_2Cl_2, δ ppm): 334.17 (m, C^9), 223.66 (m, C^1), 164.69 (d, $J_{P\text{-}C}$ = 24.2 Hz, C^3), 160.03 (d, $J_{P\text{-}C}$ = 19.6 Hz, C^7), 142.72 (s, C^5), 119.28 (d, $J_{P\text{-}C}$ = 93.6 Hz, C^2), 112.98 (s, C^6), 112.68 (s, C^4), 98.10 (d, $J_{P\text{-}C}$ = 110.2 Hz, C^8), 18.22 (d, $J_{P\text{-}C}$ = 57.3 Hz, CH_2), 7.27 (d, $J_{P\text{-}C}$ = 6.0 Hz, CH_3), 141.5-115.6 (m, other aromatic carbons). HRMS (ESI): m/z calcd for $[C_{77}H_{64}ClNP_4Os]^{2+}$, 676.6641; Found: 676.6639. Anal. Calcd. for $C_{125}H_{104}B_2ClNP_4Os$: C, 75.39; H, 5.26; N, 0.70. Found: C, 75.62; H, 5.54; N, 0.97.

Synthesis and characterization of 8d

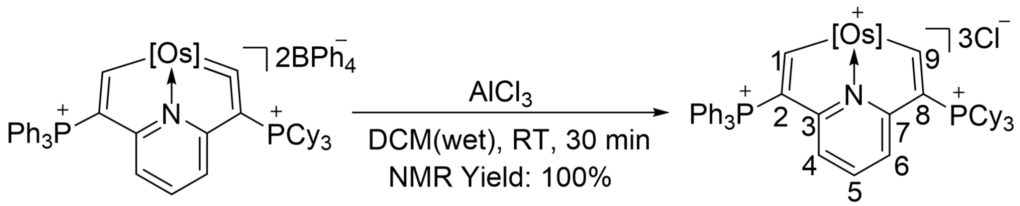

5b **8d**

[Os] = $OsCl(PPh_3)_2$

A mixture of **5b** (150 mg, 1.0 equiv) and $AlCl_3$ (15 equiv) were dissolved in DCM (wet, 2 mL). The reaction mixture was stirred for 30 minutes at room temperature to give an orange suspension, the solid suspension was removed through a filter to give an orange solution of **8d** (approximately 100% yield based on ^{1}H and ^{31}P{1H} NMR). ^{1}H-NMR (600.1 MHz, CD_2Cl_2, δ ppm): 9.25 (dd, $J_{P\text{-}H}$ = 8.4 Hz, $J_{H\text{-}H}$ = 5.1 Hz, 1H, C^9H), 9.14 (dd, $J_{P\text{-}H}$ = 11.4 Hz, $J_{H\text{-}H}$ = 5.1 Hz, 1H, C^1H), 8.11 (t, $J_{H\text{-}H}$ = 7.8 Hz, 1H, C^5H), 7.11 (br, C^6H), 6.61 (br, C^4H), 1.85-1.09 (m, 33H, Cy), 8.10-

6.59 (m, other aromatic protons plus C[5]*H*, C[6]*H*, C[4]*H*). ^{31}P-NMR (242.9 MHz, CD_2Cl_2, δ ppm): 28.90 (s, C[8]*P*Cy_3), 17.77 (s, Os*P*Ph_3), 12.02 (s, C[2]*P*Ph_3). ^{13}C-NMR (150.9 MHz, CD_2Cl_2, δ ppm): 213.22 (td apparent t, $J_{P\text{-}C}$ = 5.8 Hz, C[1]), 209.42 (br, C[9]), 160.55 (d, $J_{P\text{-}C}$ = 18.1 Hz, C[3]), 160.24 (d, $J_{P\text{-}C}$ = 16.6 Hz, C[7]), 147.46 (s, C[5]), 122.11 (d, $J_{P\text{-}C}$ = 90.5 Hz, C[2]), 119.77 (s, C[4]), 119.42 (d, $J_{P\text{-}C}$ = 75.5 Hz, C[8]), 119.02 (s, C[6]), 33.5-25.3 (m, Cy), 147.7-114.8 (m, other aromatic carbons).

Synthesis and characterization of 9

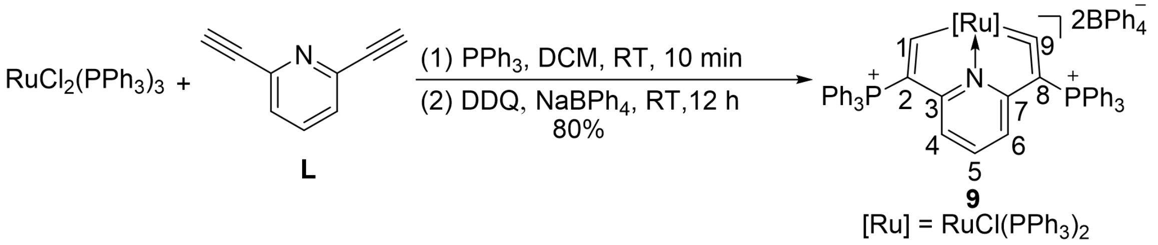

A mixture of $RuCl_2(PPh_3)_3$ (150 mg, 0.16 mmol), PPh_3 (42 mg, 0.16 mmol) and 2,6-diethynylpyridine (20 mg, 0.16 mmol) were dissolved in DCM (5 mL). The reaction mixture was stirred for 10 minutes at room temperature, and DDQ (55 mg, 0.24 mmol) and $NaBPh_4$ (547 mg, 1.6 mmol) were added subsequently. The reaction mixture was stirred for 12 hours at at room temperature to give a brown suspension and the solid suspension was removed through a filter. The filtrate was evaporated to 2 mL recrystallized from CH_2Cl_2/MeOH 1/5 mixture. The compound **9** was obtained in 80% yield (250 mg) as a yellow solid. ^{1}H-NMR (600.1 MHz, CD_2Cl_2, δ ppm): 11.20 (d, $J_{P\text{-}H}$ = 20.4 Hz, 1H, C[1]*H*), 6.88 (br, C[5]*H*), 6.14 (d, $J_{H\text{-}H}$ = 7.8 Hz, 1H, C[4]*H*), 5.94 (d, $J_{H\text{-}H}$ = 7.8 Hz, 1H, C[6]*H*), 7.81-6.79 (m, 101H, other aromatic protons plus C[5]*H*). ^{31}P-NMR (242.9 MHz, CD_2Cl_2, δ ppm): 11.67 (s, C[2]*P*Ph_3), 3.43 (s, C[8]*P*Ph_3), 28.60 (s, Ru*P*Ph_3). ^{13}C-NMR (150.9 MHz, CD_2Cl_2, δ ppm): 366.80 (m, C[9]), 237.88 (m, C[1]), 163.61 (d, $J_{P\text{-}C}$ = 28.7 Hz, C[3]), 160.55 (d, $J_{P\text{-}C}$ = 18.1 Hz, C[7]), 142.34 (s, C[5]), 117.41 (s, C[6]), 116.82 (d, $J_{P\text{-}C}$ = 80.0 Hz, C[2]), 116.22 (s, C[4]), 99.11 (d, $J_{P\text{-}C}$ = 110.2 Hz, C[8]), 136.3-117.2 (m, other aromatic carbons). HRMS (ESI): m/z calcd for $[C_{81}H_{64}ClNP_4Ru]^{2+}$, 655.6355; Found: 655.6357. Anal. Calcd. for $C_{129}H_{104}B_2ClNP_4Ru$: C, 79.45; H, 5.38; N, 0.72. Found: C, 79.21; H, 5.69; N, 0.97.

3. X-ray Crystallographic Analysis

Single-crystal X-ray diffraction data were collected on a Bruker CMOs area detector with monochromated Cu Kα radiation (λ = 1.54184 Å) for **1**, **P1'**, **2**, **3a**, **4d**, **5a**, **5c**, **7a** and **9**, on a Bruker D8-Venture with Ga generator (λ = 1.34139 Å) for **4a**. With Olex2 (Dolomanov *et al.*, 2009), the structure was solved using the SHELXT[3] structure solution program and refined with the SHELXL[4] refinement package using least-squares minimization. Non-hydrogen atoms were refined anisotropically unless otherwise stated. Hydrogen atoms were introduced at their geometric positions and refined as riding atoms unless otherwise stated. Single crystals suitable for X-ray diffraction were grown from a solution of CH_2Cl_2 (**P1'**, **2**, **3a**, **5a**, **5c**, **7a** and **9**), $ClCH_2CH_2Cl$ (**1**, **4a** and **4d**) layered with hexane. X-ray crystal structures have been deposited in the Cambridge Crystallographic Database under the deposition numbers CCDC 2314889 (**1**), CCDC 2314895 (**P1'**), CCDC 2314890 (**2**), CCDC 2314891 (**3a**), CCDC 2314892 (**4a**), CCDC 2314893 (**4d**), CCDC 2314886 (**5a**), CCDC 2314884 (**5c**), CCDC 2314888 (**7a**), CCDC 2314894 (**9**). The data can be obtained free of charge from the CCDC (www.ccdc.cam.ac.uk/data_request/cif).

Crystal data for 1

$[C_{63}H_{49}Cl_2NOsP_3]BF_4 \cdot ClCH_2CH_2Cl$, molecular weight = 1359.80, temperature = 100.0 K, monoclinic, space group $P2_1/n$, a = 22.014(3) Å, b = 21.683(3) Å, c = 12.1814(15) Å, α = 90°, β = 98.337(5)°, γ = 90°, V = 5752.9(12) Å^3, Z = 4, ρ_{calc} = 1.570 g/cm^3, μ = 7.140 mm^{-1}, F(000) = 2720.0, crystal size 0.10×0.10×0.09 mm^3, $\lambda(Cu_{K\alpha})$ = 1.54178, $2\theta_{range}$ = 8.156 to 144.834°, 54624 reflections, 10943 independent reflections (R_{int} = 0.0572, R_σ = 0.0413), data/restraints/parameters : 10943/159/759, GOF = 1.049, R_1/ wR_2 (I ≥ 2σ(I)) : 0.0359/0.0846, R_1/ wR_2 (all data) : 0.0434/0.0896, largest diff. peak/hole : 1.49-1.29 e.Å^{-3}

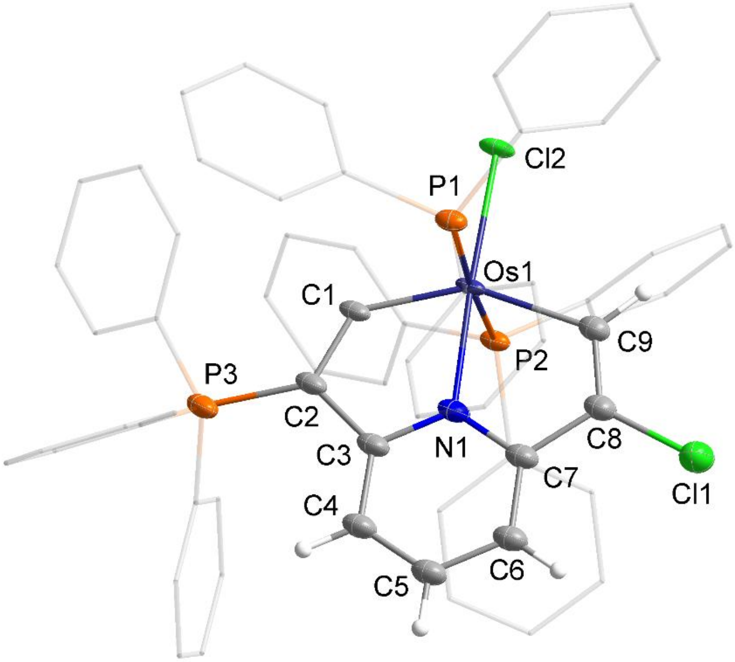

Figure S5. X-ray molecular structure for the cation of complex **1** drawn with 50% probability level. The hydrogen atoms at Ph groups were omitted for clarity. Selected bond lengths (Å) and angles (°): Os1–C1 1.841(4), Os1–N1 2.098(3), Os1–C9 2.102(4), C1–C2 1.381(5), C2–C3 1.461(5), C3–C4 1.405(5), C4–C5 1.383(6), C5–C6 1.380(6), C6–C7 1.389(5), C7–C8 1.445(5), C8–C9 1.334(5), C3–N1 1.354(5), C7–N1 1.359(5); C8–Cl1 1.754(4); Os1–C1–C2 129.2(3), C1–C2–C3 107.7(3), C2–C3–N1 111.1(3), C3–N1–Os1 118.4(2), N1–Os1–C1 73.61(13), Os1–N1–C7 120.2(2), N1–C7–C8 110.8(3), C7–C8–C9 117.3(3), C8–C9–Os1 117.4(3), N1–C3–C4 119.5(3), C3–C4–C5 119.1(4), C4–C5–C6 120.7(4), C5–C6–C7 118.8(4), C6–C7–N1 120.5(3), C3–N1–C7 124.4(3).

Crystal data for P1'

$[C_{64}H_{50}ClNOOsP_3]BPh_4·3CH_2Cl_2$, molecular weight = 1741.59, temperature = 100.0(2) K, triclinic, space group *P-1*, *a* = 14.0545(4) Å, *b* = 17.0441(5) Å, *c* = 17.7828(6) Å, *α* = 78.0980(10)°, *β* = 81.6520(10)°, *γ* = 70.8870(10)°, *V* = 3924.5(2) Å^3, *Z* = 2, ρ_{calc} = 1.474 g/cm^3, *μ* = 6.221 mm^{-1}, *F*(000) = 1764.0, crystal size 0.2×0.18×0.15 mm^3, $\lambda(Cu_{K\alpha})$ = 1.54178, $2\theta_{range}$ = 5.568 to 144.292°, 76359 reflections, 15401 independent reflections (R_{int} = 0.0546, R_σ = 0.0359), data/restraints/parameters : 15401/6/946, GOF = 1.070, R_1/ wR_2 (I ≥ 2σ(I)) : 0.0358/0.0958, R_1/ wR_2 (all data) : 0.0359/0.0958, largest diff. peak/hole : 1.93/-1.63 e.Å^{-3}

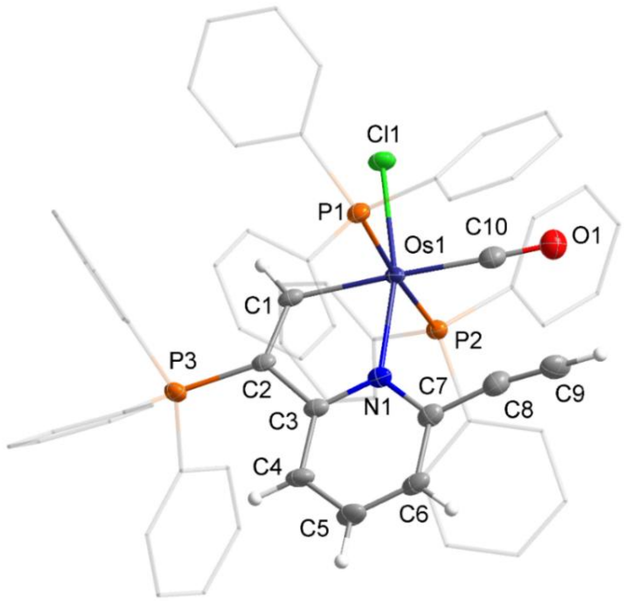

Figure S6. X-ray molecular structure for the cation of complex **P1'** drawn with 50% probability level. The hydrogen atoms at Ph groups were omitted for clarity. Selected bond lengths (Å) and angles (°): Os1–C1 2.061(3), Os1–N1 2.099(2), Os1–C10 1.934(3), C1–C2 1.350(4), C2–C3 1.467(4), C3–C4 1.385(4), C4–C5 1.383(4), C5–C6 1.382(5), C6–C7 1.392(4), C7–C8 1.423(4), C8–C9 1.217(6), C3–N1 1.382(3), C7–N1 1.366(4); Os1–C1–C2 117.6(2), C1–C2–C3 115.7(2), C2–C3–N1 112.9(2), C3–N1–Os1 116.57(18), N1–Os1–C1 76.96(10), Os1–N1–C7 126.57(18), N1–C7–C8 119.9(3), C7–C8–C9 170.1(4), N1–C3–C4 121.7(3), C3–C4–C5 120.8(3), C4–C5–C6 118.1(3), C5–C6–C7 119.9(3), C6–C7–N1 122.6(3), C3–N1–C7 116.9(2).

Crystal data for 2

$[C_{63}H_{50}Cl_2NOsP_3]Cl_2$, molecular weight = 1245.95, temperature = 100.0 K, monoclinic, space group $P2_1/n$, a = 12.5847(5) Å, b = 20.8470(8) Å, c = 21.1394(8) Å, α = 90°, β = 93.8120(10)°, γ = 90°, V = 5533.7(4) $Å^3$, Z = 4, ρ_{calc} = 1.496 g/cm^3, μ = 7.262 mm^{-1}, $F(000)$ = 2496.0, crystal size 0.15×0.13×0.12 mm^3, $\lambda(Cu_{K\alpha})$ = 1.54178, $2\theta_{range}$ = 5.96 to 144.644°, 95746 reflections, 10922 independent reflections (R_{int} = 0.0378, R_σ = 0.0183), data/restraints/parameters : 10922/0/658, GOF = 1.054, R_1/ wR_2 (I ≥ 2σ(I)) : 0.0316/0.0823, R_1/ wR_2 (all data) : 0.0324/0.0830, largest diff. peak/hole : 1.64/-1.74 e.$Å^{-3}$

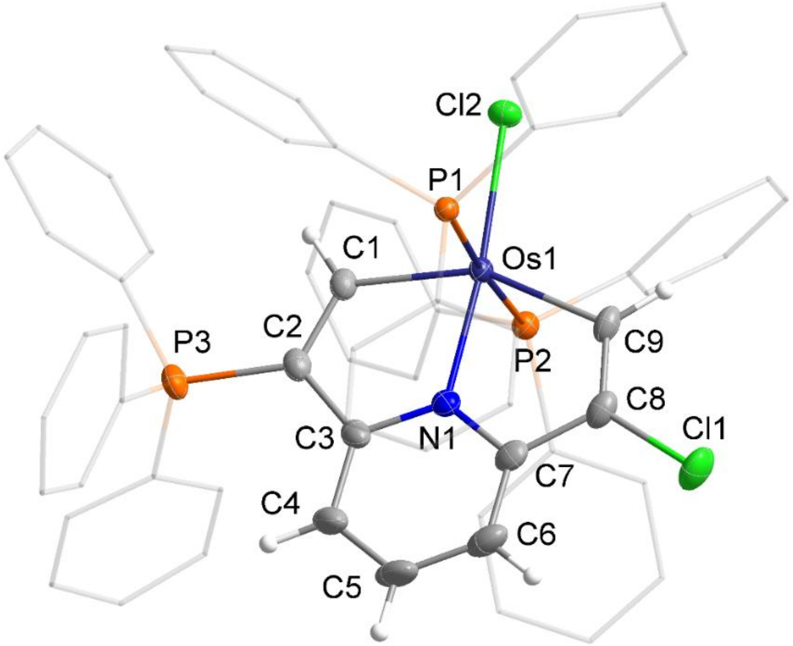

Figure S7. X-ray molecular structure for the cation of complex **2** drawn with 50% probability level. The hydrogen atoms at Ph groups were omitted for clarity. Selected bond lengths (Å) and angles (°): Os1–C1 2.025(3), Os1–N1 2.078(3), Os1–C9 2.096(3), C1–C2 1.367(5), C2–C3 1.458(5), C3–C4 1.384(5), C4–C5 1.396(6), C5–C6 1.382(6), C6–C7 1.382(5), C7–C8 1.449(5), C8–C9 1.345(5), C3–N1 1.365(4), C7–N1 1.359(4); Os1–C1–C2 120.1(2), C1–C2–C3 113.7(3), C2–C3–N1 111.3(3), C3–N1–Os1 119.6(2), N1–Os1–C1 75.21(12), Os1–N1–C7 119.6(2), N1–C7–C8 110.9(3), C7–C8–C9 117.83), C8–C9–Os1 115.7(2), N1–C3–C4 120.4(3), C3–C4–C5 119.0(4), C4–C5–C6 119.8(4), C5–C6–C7 119.6(4), C6–C7–N1 120.5(3), C3–N1–C7 120.6(3).

Crystal data for 3a

$[C_{63}H_{49}Cl_2NOsP_3S]PF_6$, molecular weight = 1351.07, temperature = 100.00 K, triclinic, space group *P-1*, *a* = 14.1630(7) Å, *b* = 14.2184(7) Å, *c* = 15.9648(8) Å, α = 98.109(2)°, β = 106.294(2)°, γ = 101.236(2)°, *V* = 2960.5(3) Å^3, *Z* = 2, ρ_{calc} = 1.516 g/cm^3, μ = 6.744 mm^{-1}, *F*(000) = 1348.0, crystal size 0.11×0.10×0.10 mm^3, $\lambda(Cu_{K\alpha})$ = 1.54178, $2\theta_{range}$ = 5.896 to 144.51°, 79552 reflections, 11652 independent reflections (R_{int} = 0.0526, R_{σ} = 0.0287), data/restraints/parameters : 11652/0/703, GOF = 1.036, R_1/ wR_2 (I ≥ 2σ(I)) : 0.0311/0.0778, R_1/ wR_2 (all data) : 0.0337/0.0792, largest diff. peak/hole : 1.69/-1.61 e.Å^{-3}

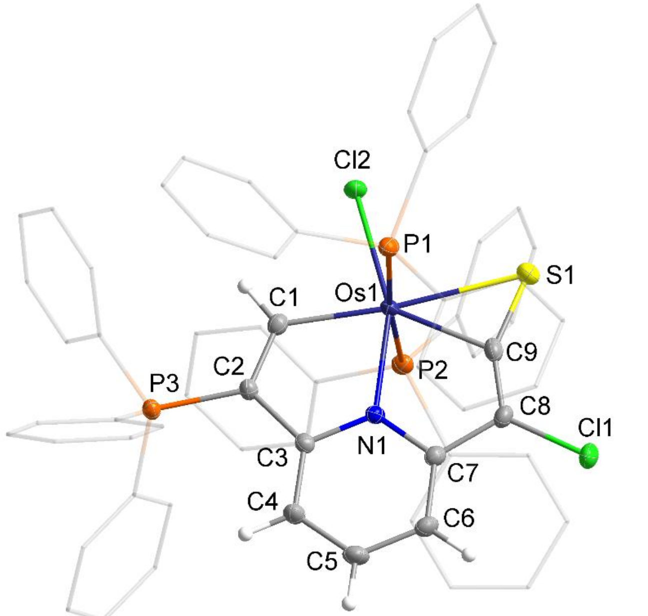

Figure S8. X-ray molecular structure for the cation of complex **3a** drawn with 50% probability level. The hydrogen atoms at Ph groups were omitted for clarity. Selected bond lengths (Å) and angles (°): Os1–C1 2.027(3), Os1–N1 2.103(2), Os1–C9 2.055(3), Os1–S1 2.5588(8), C1–C2 1.363(4), C2–C3 1.456(4), C3–C4 1.390(4), C4–C5 1.392(5), C5–C6 1.375(5), C6–C7 1.396(4), C7–C8 1.425(4), C8–C9 1.335(5), S1–C9 1.624(3), C3–N1 1.367(4), C7–N1 1.365(4); Os1–C1–C2 121.2(2), C1–C2–C3 113.8(3), C2–C3–N1 110.8(3), C3–N1–Os1 119.9(2), N1–Os1–C1 74.20(11), Os1–N1–C7 120.7(2), N1–C7–C8 111.3(3), C7–C8–C9 113.3(3), C8–C9–Os1 122.6(2), S1–Os1–C9 39.35(9), Os1–S1–C9 53.34(11), Os1–C9–S1 87.31(15), N1–C3–C4 120.7(3), C3–C4–C5 119.6(3), C4–C5–C6 119.9(3), C5–C6–C7 119.0(3), C6–C7–N1 121.4(3), C3–N1–C7 119.4(3).

Crystal data for 4a

$[C_{69}H_{53}Cl_2NOsP_3S]BF_4 \cdot 2ClCH_2CH_2Cl$, molecular weight = 1566.90, temperature = 100.00 K, triclinic, space group *P-1*, a = 9.9632(9) Å, b = 11.5217(10) Å, c = 29.202(3) Å, α = 78.917(3)°, β = 82.668(3)°, γ = 88.004(3)°, V = 3262.6(3) Å^3, Z = 2, ρ_{calc} = 1.595 g/cm^3, μ = 4.957 mm^{-1}, F(000) = 1572.0, crystal size 0.1×0.1×0.08 mm^3, $\lambda(Ga_{K\alpha})$ = 1.34139, $2\theta_{range}$ = 6.826 to 111.99°, 60506 reflections, 12772 independent reflections (R_{int} = 0.0686, R_σ = 0.0489), data/restraints/parameters : 12772/99/839, GOF = 1.044, R_1/ wR_2 (I ≥ 2σ(I)) : 0.0486/0.1176, R_1/ wR_2 (all data) : 0.0516/0.1193, largest diff. peak/hole : 1.95/-2.01 e.Å^{-3}

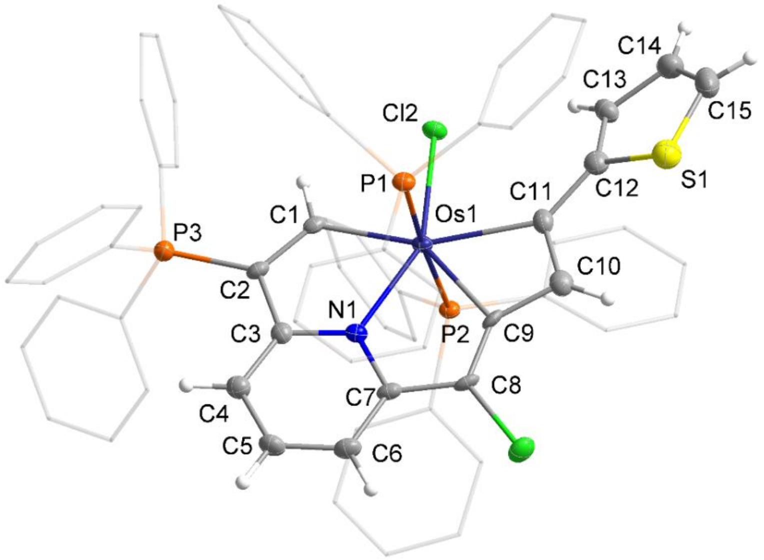

Figure S9. X-ray molecular structure for the cation of complex **4a** drawn with 50% probability level. The hydrogen atoms at Ph groups were omitted for clarity. Selected bond lengths (Å) and angles (°): Os1–C1 2.071(5), Os1–N1 2.135(4), Os1–C9 2.098(4), C1–C2 1.358(7), C2–C3 1.458(6), C3–C4 1.380(7), C4–C5 1.397(7), C5–C6 1.375(7), C6–C7 1.390(7), C7–C8 1.415(7), C8–C9 1.333(7), C3–N1 1.366(6), C7–N1 1.382(6), C9–C10 1.414(7), C10–C11 1.340(7); Os1–C1–C2 122.9(3), C1–C2–C3 122.9(4), C2–C3–N1 110.5(4), C3–N1–Os1 121.6(3), N1–Os1–C1 71.80(16), Os1–N1–C7 119.8(3), N1–C7–C8 111.4(4), C7–C8–C9 115.4(4), C8–C9–Os1 120.9(3), N1–Os1–C9 72.20(17), C9–Os1–C11 58.24(19), Os1–C9–C10 102.9(3), C9–C10–C11 100.6(4), N1–C3–C4 121.5(4), C3–C4–C5 119.4(5), C4–C5–C6 119.7(4), C5–C6–C7 119.3(5), C6–C7–N1 121.2(4), C3–N1–C7 118.6(4).

Crystal data for 4d

$[C_{69}H_{52}BrCl_2NOsP_3S]BF_4\cdot ClCH_2CH_2Cl$, molecular weight = 1546.90, temperature = 100.00 K, triclinic, space group *P-1*, a = 10.0004(13) Å, b = 11.4627(15) Å, c = 27.876(4) Å, α = 101.832(5)°, β= 93.282(5)°, γ = 91.924(5)°, V = 3119.1(7) Å^3, Z = 2, ρ_{calc} = 1.647 g/cm^3, μ = 7.694 mm^{-1}, F(000) = 1540.0, crystal size 0.12×0.10×0.10 mm^3, $\lambda(Cu_{K\alpha})$ = 1.54178, $2\theta_{range}$ = 6.492 to 144.782°, 61603 reflections, 11830 independent reflections (R_{int} = 0.0566, R_σ = 0.0378), data/restraints/parameters : 11830/152/831, GOF = 1.047, R_1/ wR_2 (I ≥ 2σ(I)) : 0.0356/0.0935, R_1/ wR_2 (all data) : 0.0367/0.0943, largest diff. peak/hole : 1.54/-1.72 eÅ^{-3}

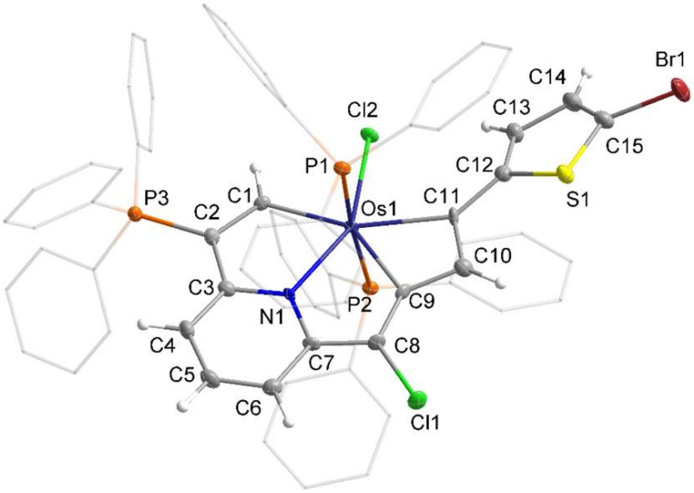

Figure S10. X-ray molecular structure for the cation of complex **4d** drawn with 50% probability level. The hydrogen atoms at Ph groups were omitted for clarity. Selected bond lengths (Å) and angles (°): Os1–C1 2.090(3), Os1–N1 2.147(3), Os1–C9 2.087(3), Os1–C11 2.231(3), C1–C2 1.359(5), C2–C3 1.451(5), C3–C4 1.392(5), C4–C5 1.386(5), C5–C6 1.368(5), C6–C7 1.394(5), C7–C8 1.409(5), C8–C9 1.338(5), C3–N1 1.367(5), C7–N1 1.368(4), C9–C10 1.414(5), C10–C11 1.347(5); Os1–C1–C2 122.0(3), C1–C2–C3 113.6(3), C2–C3–N1 111.3(3), C3–N1–Os1 120.8(2), N1–Os1–C1 72.07(12), Os1–N1–C7 120.5(2), N1–C7–C8 111.0(3), C7–C8–C9 115.3(3), C8–C9–Os1 121.4(3), N1–Os1–C9 71.48(12), C9–Os1–C11 58.85(13), Os1–C9–C10 102.5(2), C9–C10–C11 100.7(3), N1–C3–C4 120.3(3), C3–C4–C5 120.1(3), C4–C5–C6 120.0(3), C5–C6–C7 118.5(3), C6–C7–N1 122.2(3), C3–N1–C7 118.7(3).

Crystal data for 5a

$[C_{66}H_{58}ClNOsP_4]2BPh_4\cdot5CH_2Cl_2$, molecular weight = 2277.71, temperature = 100.0(2) K, triclinic, space group *P-1*, *a* = 15.7974(5) Å, *b* = 17.7933(5) Å, *c* = 20.4463(6) Å, *α* = 75.9630(10)°, *β* = 89.7200(10)°, *γ* = 73.0860(10)°, *V* = 5321.0(3) $Å^3$, *Z* = 2, ρ_{calc} = 1.422 g/cm^3, *μ* = 5.758 mm^{-1}, *F*(000) = 2324.0, crystal size 0.3×0.24×0.15 mm^3, $\lambda(Cu_{K\alpha})$ = 1.54178, $2\theta_{range}$ = 5.860 to 144.30°, 50036 reflections, 19988 independent reflections (R_{int} = 0.0516, R_σ = 0.0507), data/restraints/parameters : 19988/0/1246, GOF = 1.046, R_1/ wR_2 (I ≥ 2σ(I)) : 0.0507/0.1336, R_1/ wR_2 (all data) : 0.0516/0.1345, largest diff. peak/hole : 3.48/-2.52 e.$Å^{-3}$

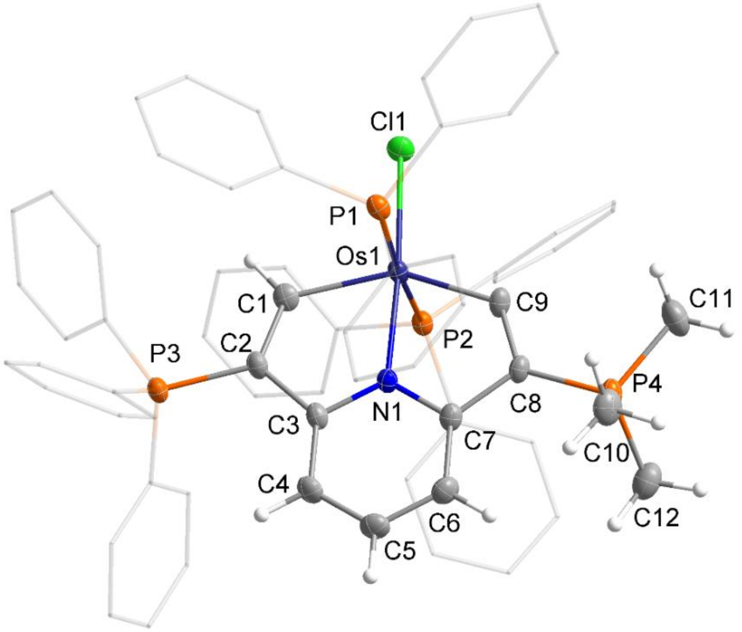

Figure S11. X-ray molecular structure for the cation of complex **5a** drawn with 50% probability level. The hydrogen atoms at Ph groups were omitted for clarity. Selected bond lengths (Å) and angles (°): Os1–C1 2.036(4), Os1–N1 2.083(3), Os1–C9 1.907(4), C1–C2 1.351(5), C2–C3 1.461(5), C3–C4 1.394(5), C4–C5 1.393(6), C5–C6 1.375(5), C6–C7 1.383(5), C7–C8 1.459(5), C8–C9 1.371(5), C3–N1 1.365(5), C7–N1 1.374(5); Os1–C1–C2 121.3(3), C1–C2–C3 113.7(3), C2–C3–N1 110.0(3), C3–N1–Os1 121.1(2), N1–Os1–C1 73.74(13), Os1–N1–C7 119.8(2), N1–C7–C8 110.0(3), C7–C8–C9 109.7(3), C8–C9–Os1 126.9(3), N1–C3–C4 121.0(3), C3–C4–C5 119.3(3), C4–C5–C6 129.4(3), C5–C6–C7 120.1(3), C6–C7–N1 121.0(3), C3–N1–C7 119.1(3).

Crystal data for 5c

$[C_{81}H_{64}ClNOsP_4]2BPh_4$, molecular weight = 2039.28, temperature = 100.0 K, triclinic, space group *P-1*, a = 17.3078(18) Å, b = 17.5817(18) Å, c = 18.5767(19) Å, α = 79.878(4)°, β = 73.879(4)°, γ = 87.864(4)°, V = 5345.7(10) Å^3, Z = 2, ρ_{calc} = 1.267 g/cm^3, μ = 3.430 mm^{-1}, F(000) = 2096.0, crystal size 0.15×0.15×0.12 mm^3, $\lambda(Cu_{K\alpha})$ = 1.54178, $2\theta_{range}$ = 5.316 to 145.386°, 86820 reflections, 20331 independent reflections (R_{int} = 0.0553, R_{σ} = 0.0403), data/restraints/parameters : 20331/0/1243, GOF = 1.088, R_1/ wR_2 (I ≥ 2σ(I)) : 0.0366/0.1086, R_1/ wR_2 (all data) : 0.0404/0.1113, largest diff. peak/hole : 0.91/-1.51 e.Å^{-3}

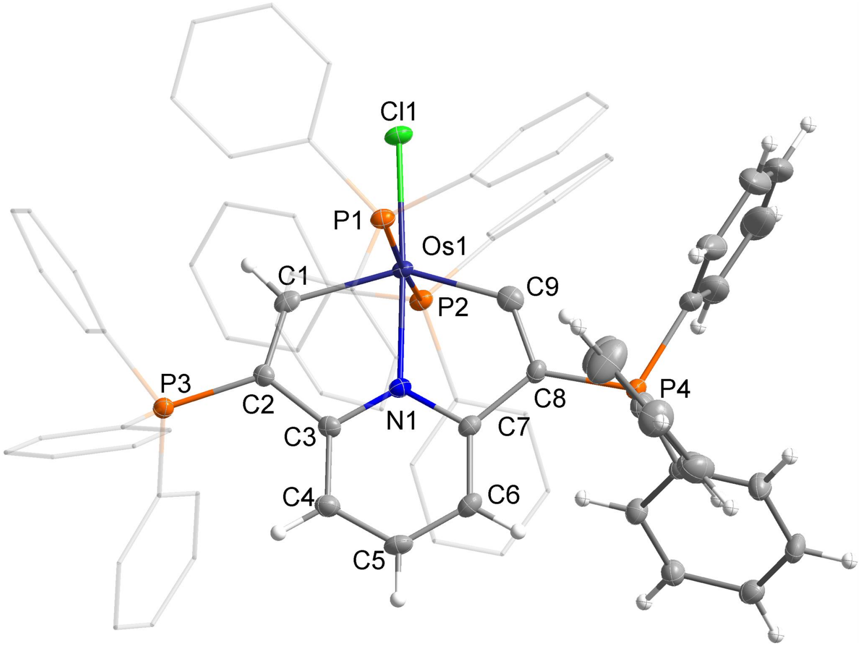

Figure S12. X-ray molecular structure for the cation of complex **5c** drawn with 50% probability level. The hydrogen atoms at Ph groups were omitted for clarity. Selected bond lengths (Å) and angles (°): Os1–C1 2.027(3), Os1–N1 2.093(2), Os1–C9 1.916(3), C1–C2 1.362(4), C2–C3 1.459(4), C3–C4 1.392(4), C4–C5 1.393(4), C5–C6 1.384(5), C6–C7 1.391(4), C7–C8 1.465(4), C8–C9 1.354(4), C3–N1 1.364(4), C7–N1 1.370(4); Os1–C1–C2 121.4(2), C1–C2–C3 113.3(3), C2–C3–N1 110.8(2), C3–N1–Os1 120.36(19), N1–Os1–C1 74.10(11) N1–Os1–C9 73.79(11), Os1–N1–C7 119.15(19), N1–C7–C8 110.1(2), C7–C8–C9 110.5(3), C8–C9–Os1 126.3(2), N1–C3–C4 120.5(3), C3–C4–C5 119.`(3), C4–C5–C6 120.3(3), C5–C6–C7 119.1(3), C6–C7–N1 120.5(3), C3–N1–C7 120.5(2).

Crystal data for 7a

$[C_{71}H_{60}ClNOsP_4]2BPh_4$, molecular weight = 1915.14, temperature = 100.0 K, triclinic, space group *P-1*, a = 16.9627(8) Å, b = 19.3429(9) Å, c = 22.1477(10) Å, α = 110.864(2)°, β = 94.765(2)°, γ = 114.869(2)°, V = 5923.8(5) Å^3, Z = 2, ρ_{calc} = 1.074 g/cm^3, μ = 3.065 mm^{-1}, F(000) = 1968.0, crystal size 0.14×0.12×0.10 mm^3, $\lambda(Cu_{K\alpha})$ = 1.54178, $2\theta_{range}$ = 5.586 to 145.008°, 113199 reflections, 23065 independent reflections (R_{int} = 0.0600, R_σ = 0.0438), data/restraints/parameters : 23065/36/1143, GOF = 1.026, R_1/ wR_2 (I ≥ 2σ(I)) : 0.0553/0.1635, R_1/ wR_2 (all data) : 0.0584/0.1669, largest diff. peak/hole : 2.15/-2.01 e.Å^{-3}

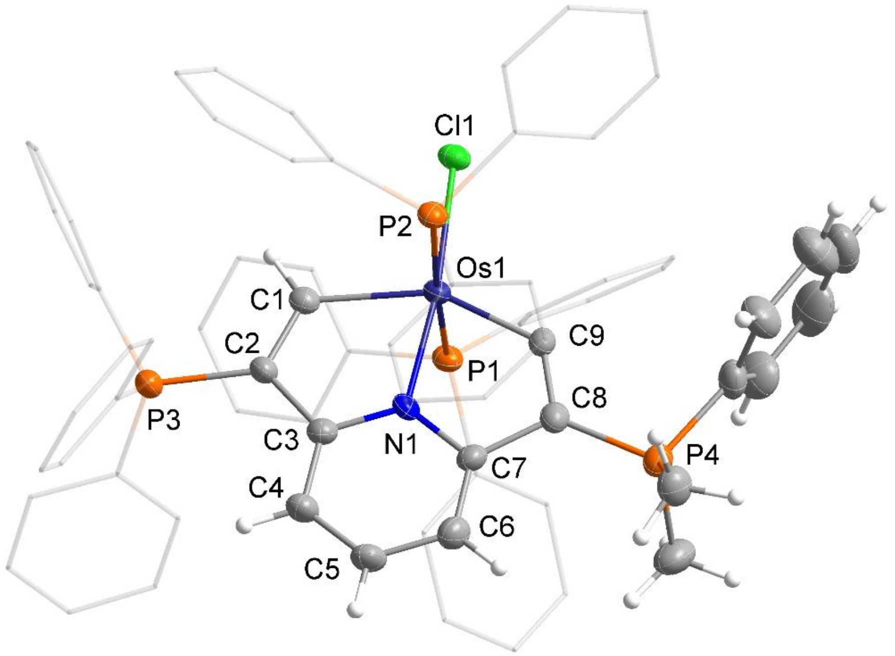

Figure S13. X-ray molecular structure for the cation of complex **7a** drawn with 50% probability level. The hydrogen atoms at Ph groups were omitted for clarity. Selected bond lengths (Å) and angles (°): Os1–C1 2.059(4), Os1–N1 2.085(3), Os1–C9 1.874(4), C1–C2 1.367(6), C2–C3 1.463(6), C3–C4 1.399(6), C4–C5 1.381(6), C5–C6 1.388(7), C6–C7 1.373(6), C7–C8 1.463(6), C8–C9 1.356(6), C3–N1 1.364(5), C7–N1 1.382(5); Os1–C1–C2 119.9(3), C1–C2–C3 113.8(4), C2–C3–N1 111.1(4), C3–N1–Os1 120.7(3), N1–Os1–C1 74.42(15), Os1–N1–C7 119.2(3), N1–C7–C8 109.0(4), C7–C8–C9 110.4(4), C8–C9–Os1 127.6(3), N1–C3–C4 120.1(4), C3–C4–C5 119.4(4), C4–C5–C6 120.3(4), C5–C6–C7 119.4(4), C6–C7–N1 120.7(4), C3–N1–C7 120.1(4).

Crystal data for 9

$[C_{81}H_{64}ClNRuP_4]2BPh_4$, molecular weight = 1950.15, temperature = 100.0 K, triclinic, space group *P-1*, *a* = 17.3296(4) Å, *b* = 17.6406(4) Å, *c* = 18.5079(4) Å, α = 79.8370(10)°, β = 73.9580(10)°, γ = 87.9760(10)°, *V* = 5351.8(2) Å^3, *Z* = 2, ρ_{calc} = 1.210 g/cm^3, μ = 2.361 mm^{-1}, *F*(000) = 2032.0, crystal size 0.13×0.11×0.11 mm^3, $\lambda(Cu_{K\alpha})$ = 1.54178, $2\theta_{range}$ = 5.044 to 144.864°, 56038 reflections, 20270 independent reflections (R_{int} = 0.0360, R_σ = 0.0426), data/restraints/parameters : 20270/0/1243, GOF = 1.038, R_1/ wR_2 (I ≥ 2σ(I)) : 0.0415/0.1064, R_1/ wR_2 (all data) : 0.0448/0.1084, largest diff. peak/hole: 0.94/-1.00 e.Å^{-3}

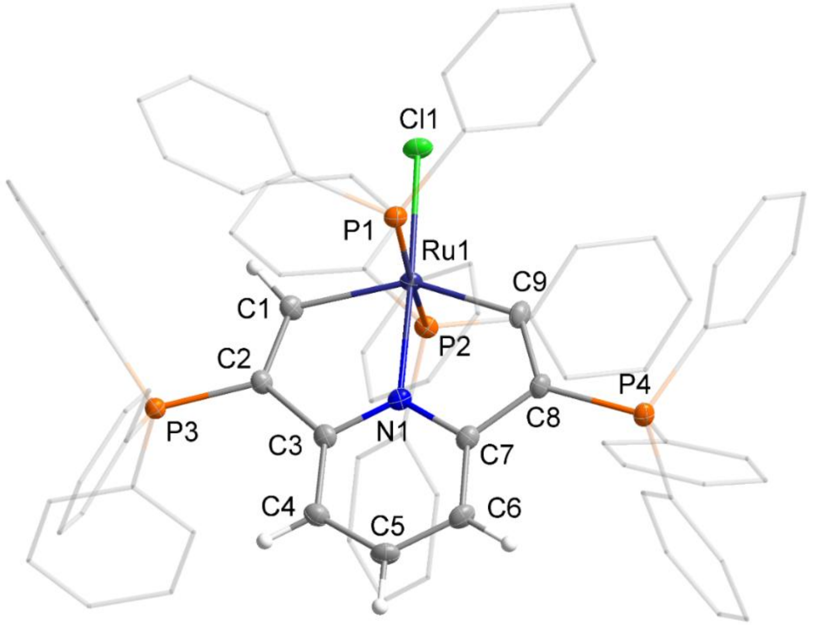

Figure S14. X-ray molecular structure for the cation of complex **9** drawn with 50% probability level. The hydrogen atoms at Ph groups were omitted for clarity. Selected bond lengths (Å) and angles (°): Ru1–C1 1.998(2), Ru1–N1 2.0864(16), Ru1–C9 1.916(2), C1–C2 1.361(3), C2–C3 1.463(3), C3–C4 1.396(3), C4–C5 1.385(3), C5–C6 1.389(3), C6–C7 1.396(3), C7–C8 1.464(3), C8–C9 1.353(3), C3–N1 1.357(3), C7–N1 1.362(3); Ru1–C1–C2 121.89(16), C1–C2–C3 112.74(18), C2–C3–N1 110.89(17), C3–N1–Ru1 119.90(13), N1–Ru1–C1 74.48(8), Ru1–N1–C7 119.26(14), N1–C7–C8 110.00(17), C7–C8–C9 111.04(19), C8–C9–Ru1 125.49(17), N1–C3–C4 120.44(19), C3–C4–C5 119.0(2), C4–C5–C6 120.42(19), C5–C6–C7 118.77(19), C6–C7–N1 120.48(19), C3–N1–C7 120.83(17).

4. NMR and HRMS Spectra

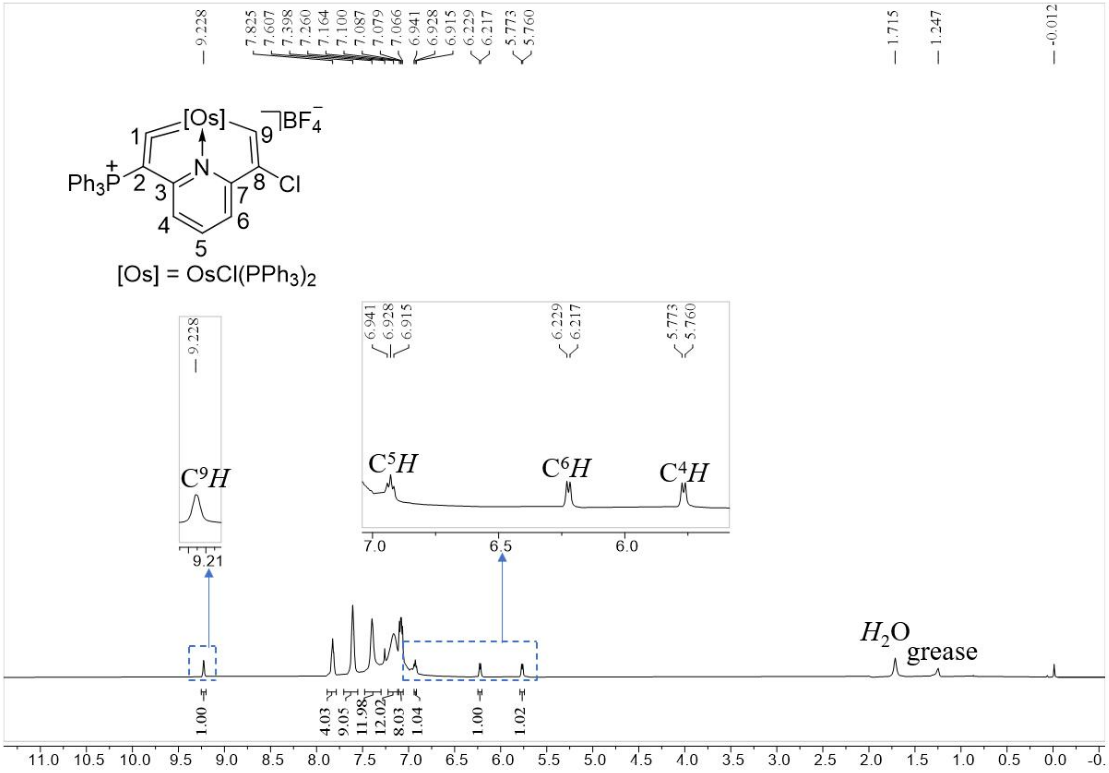

Figure S15. The ^{1}H NMR (600.1 MHz, $CDCl_3$) spectrum for complex **1**.

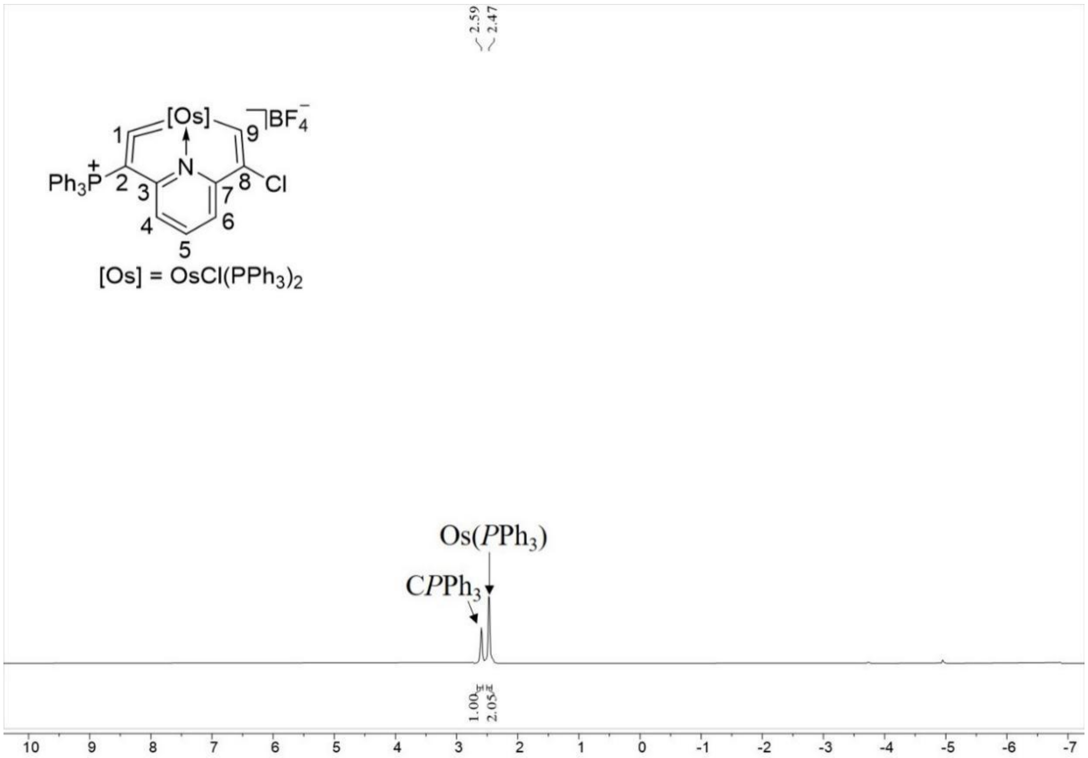

Figure S16. The ^{31}P{^{1}H} NMR (242.9 MHz, $CDCl_3$) spectrum for complex **1**.

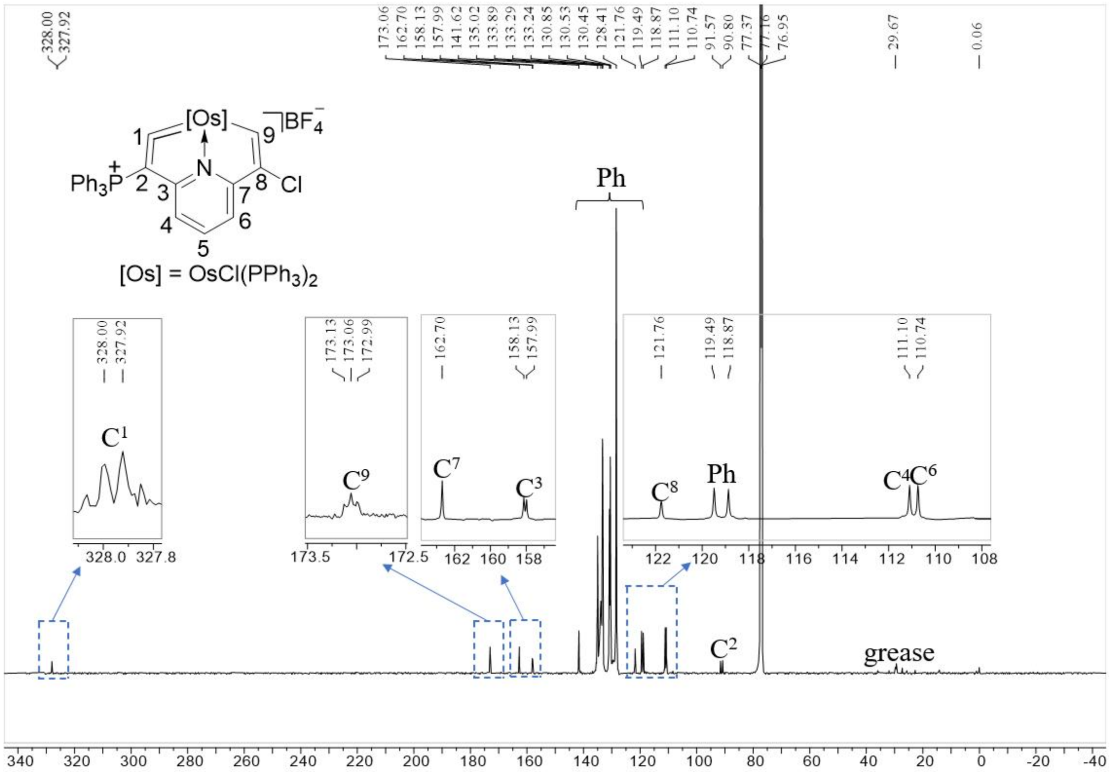

Figure S17. The $^{13}C\{^1H\}$ NMR (150.9 MHz, $CDCl_3$) spectrum for complex **1**.

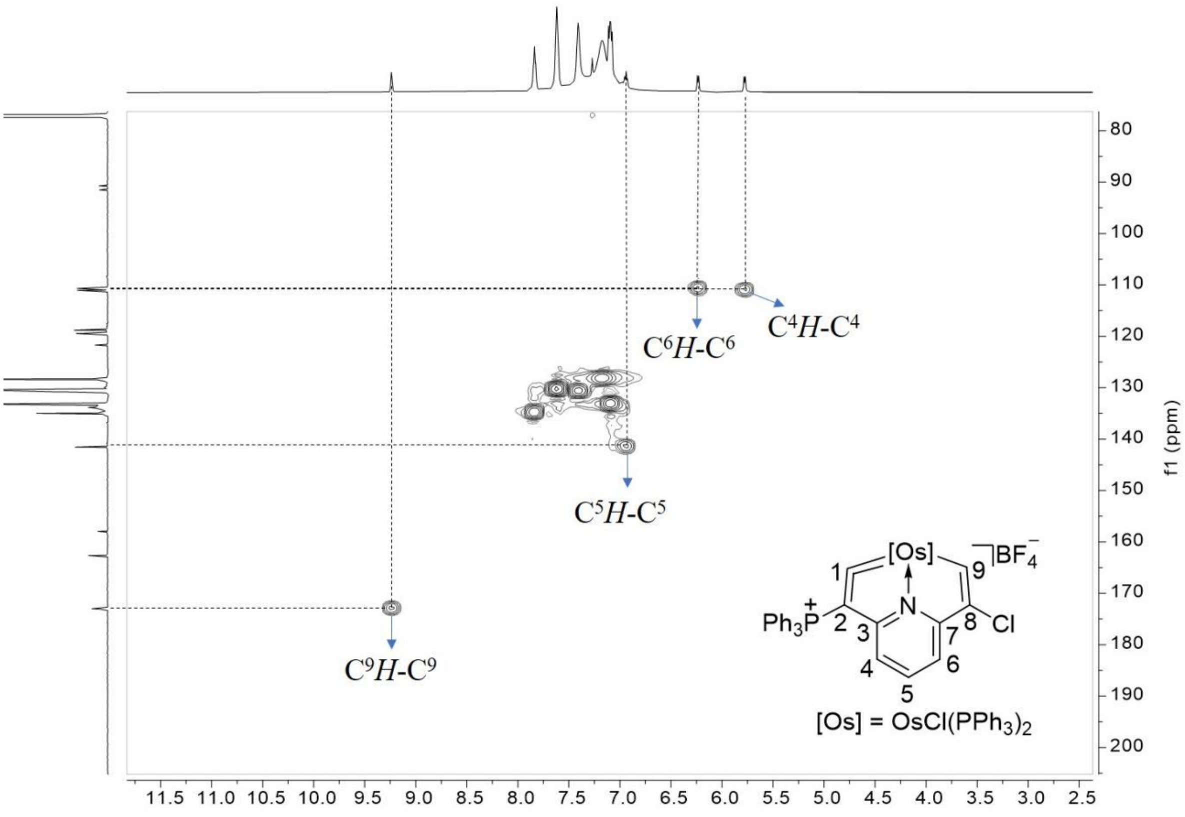

Figure S18. The ^{1}H-^{13}C HSQC (150.9 MHz, CD_2Cl_2) spectrum for complex **1**.

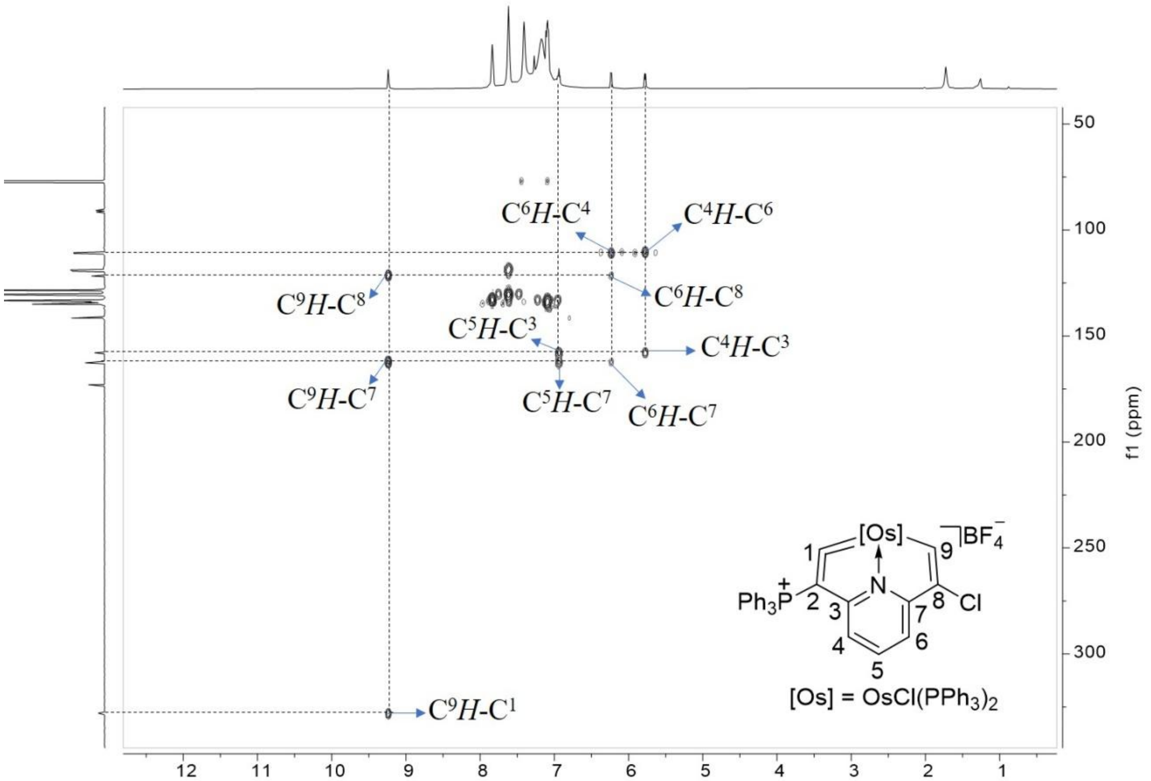

Figure S19. The ^{1}H-^{13}C HMBC (150.9 MHz, CD_2Cl_2) spectrum for complex **1**.

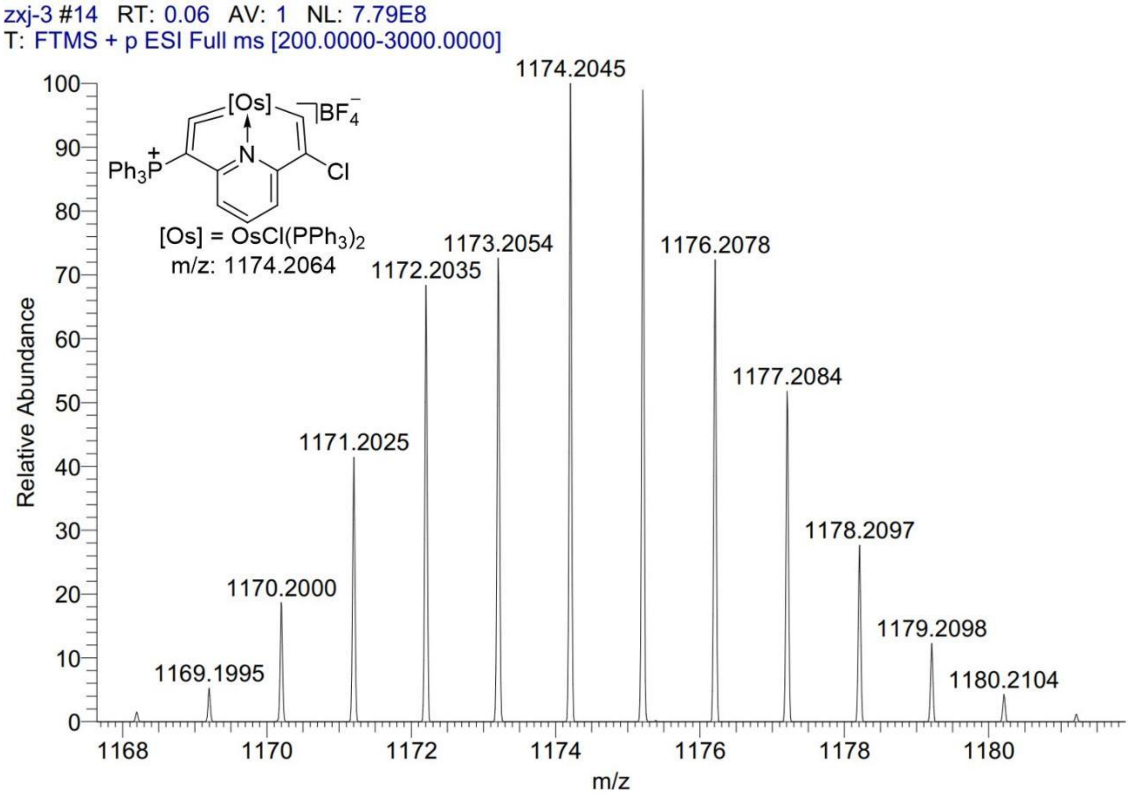

Figure S20. Positive-ion ESI-MS spectrum of [**1**]$^+$ measured in methanol.

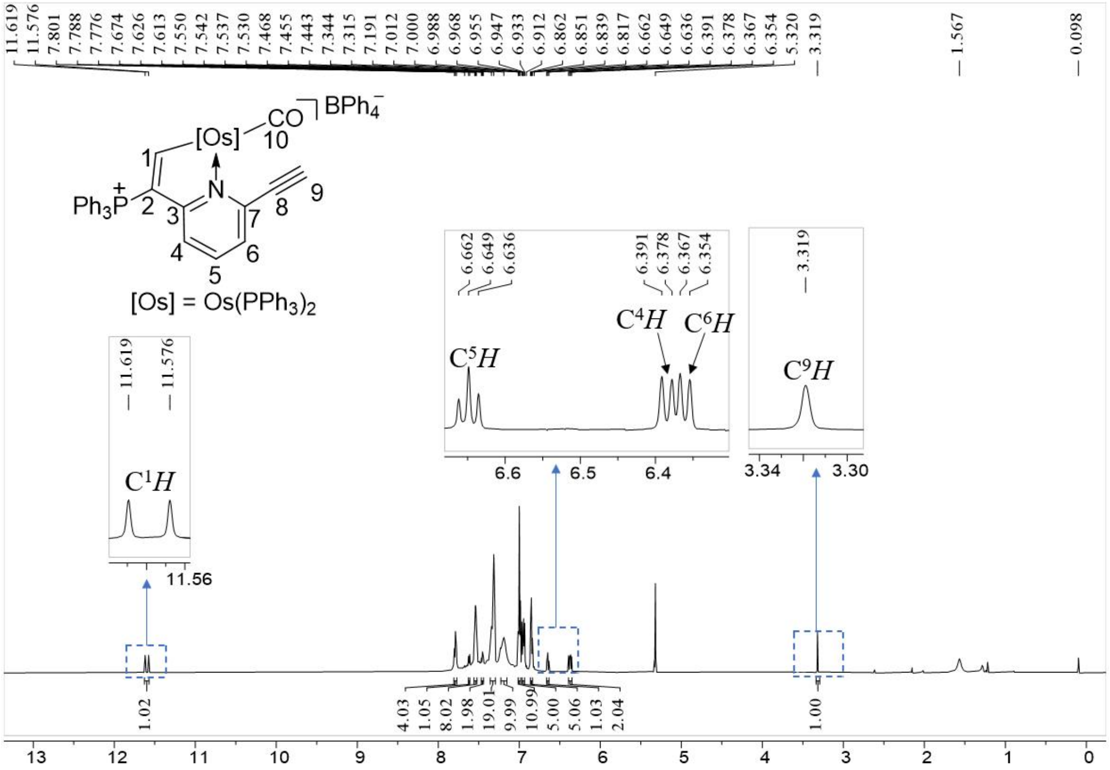

Figure S21. The ^{1}H NMR (600.1 MHz, CD_2Cl_2) spectrum for complex **P1'**.

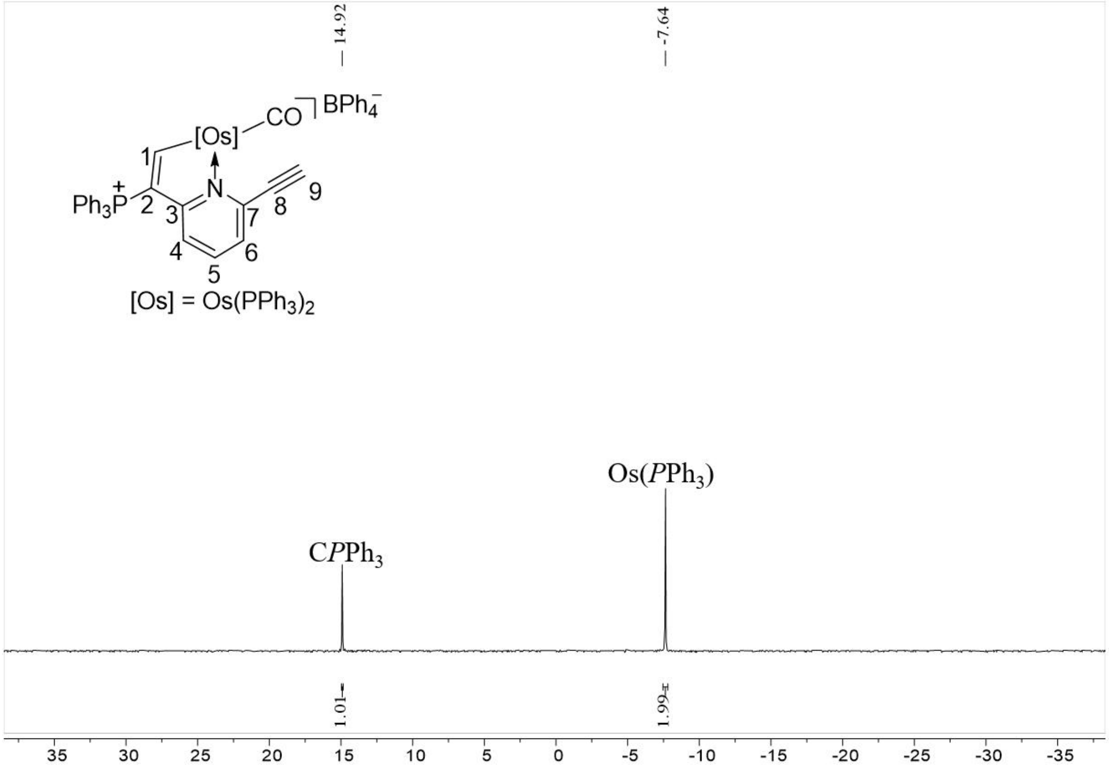

Figure S22. The $^{31}P\{^1H\}$ NMR (242.9 MHz, CD_2Cl_2) spectrum for complex **P1'**.

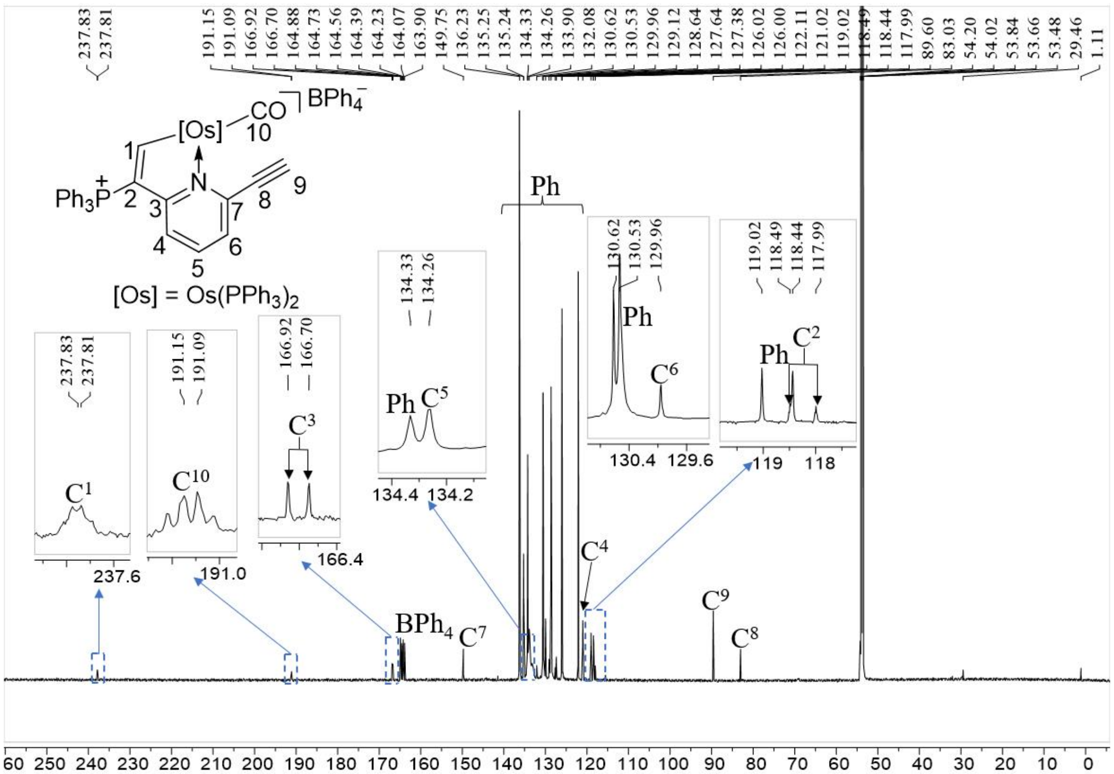

Figure S23. The $^{13}C\{^{1}H\}$ NMR (150.9 MHz, CD_2Cl_2) spectrum for complex **P1'**.

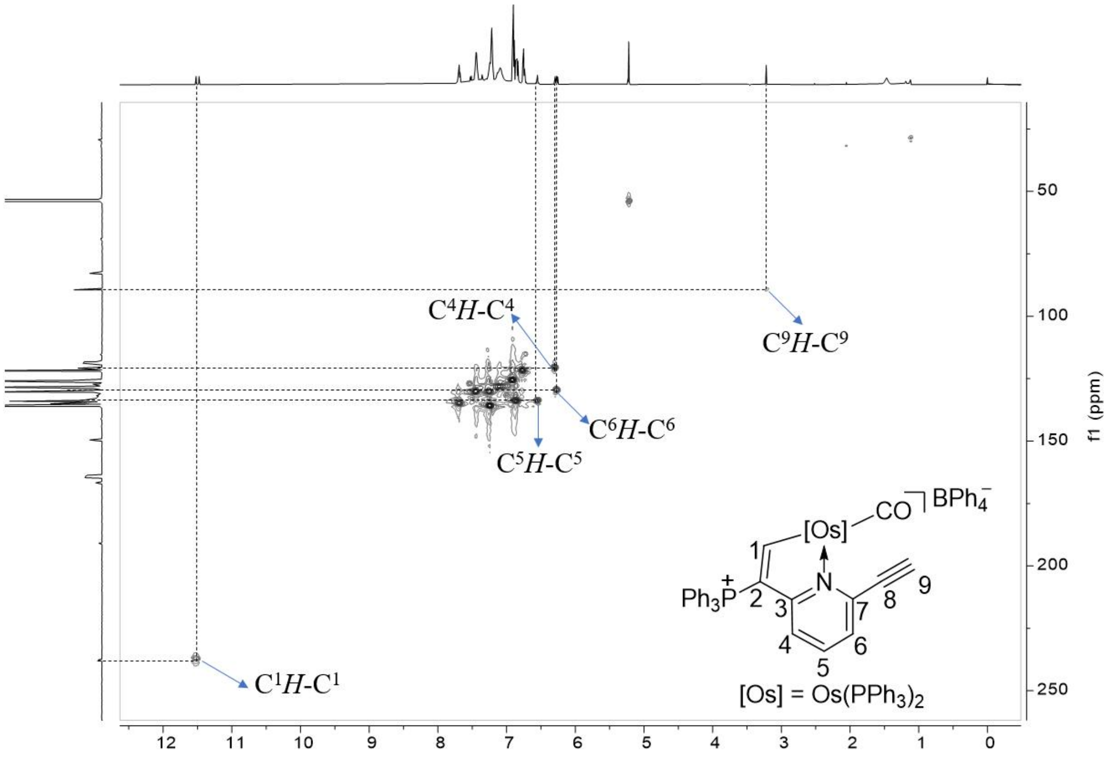

Figure S24. The ^{1}H-^{13}C HSQC (150.9 MHz, CD_2Cl_2) spectrum for complex **P1'**.

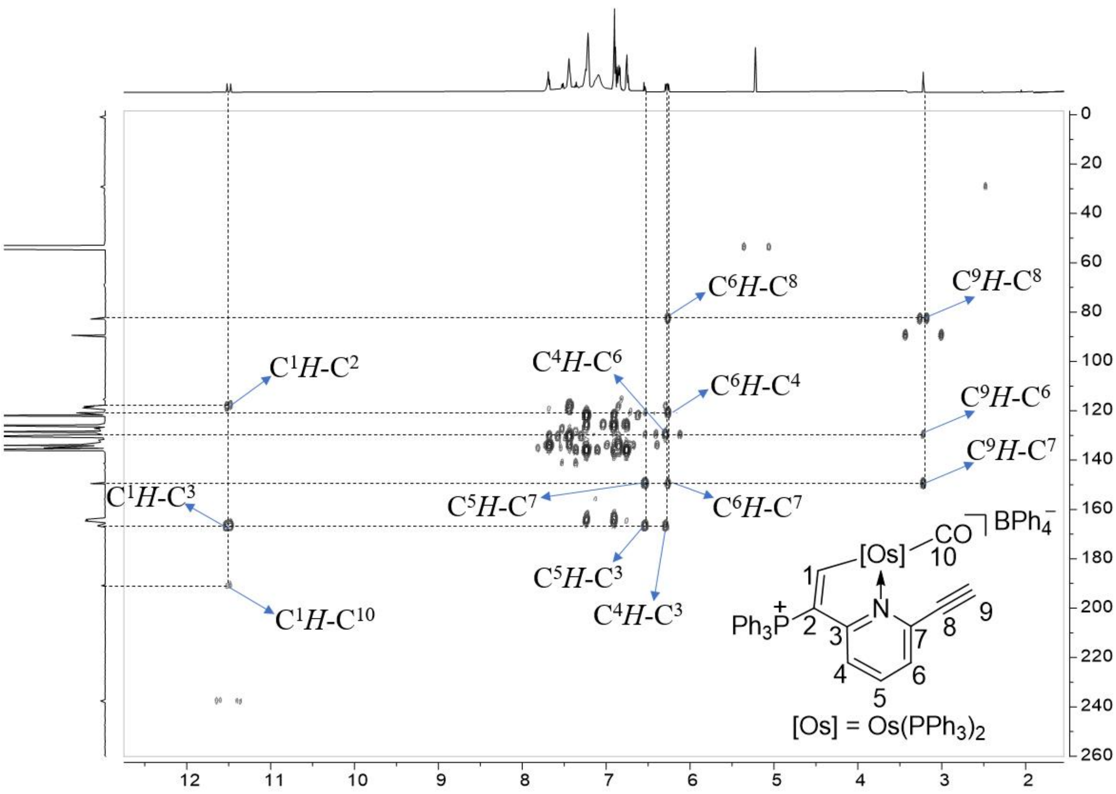

Figure S25. The ^{1}H-^{13}C HMBC (150.9 MHz, CD_2Cl_2) spectrum for complex **P1'**.

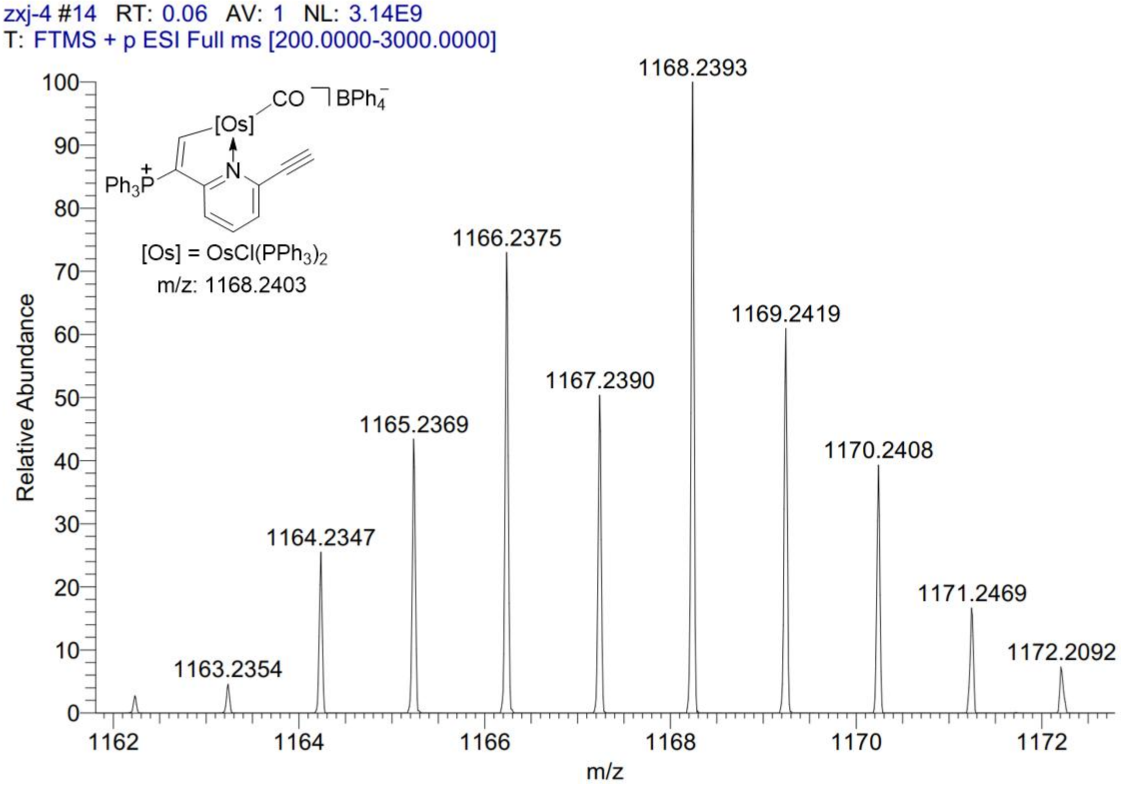

Figure S26. Positive-ion ESI-MS spectrum of [**P1'**]$^{+}$ measured in methanol.

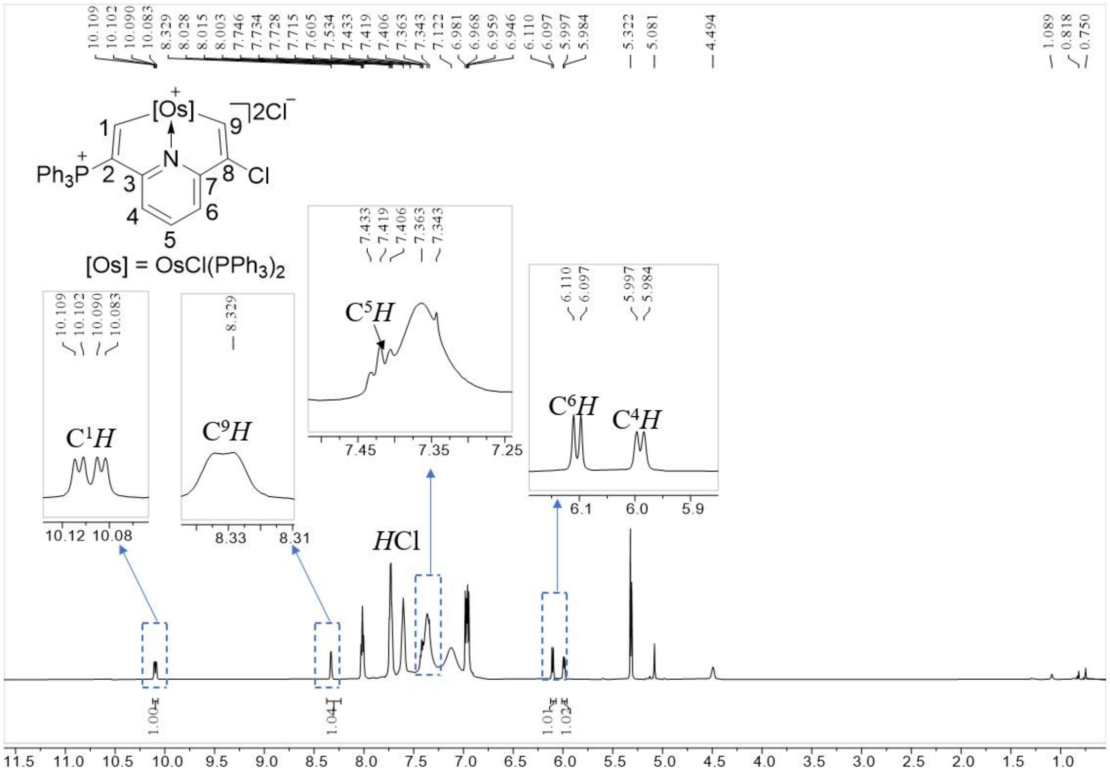

Figure S27. The ^{1}H NMR (600.1 MHz, CD_2Cl_2) spectrum for complex **2**.

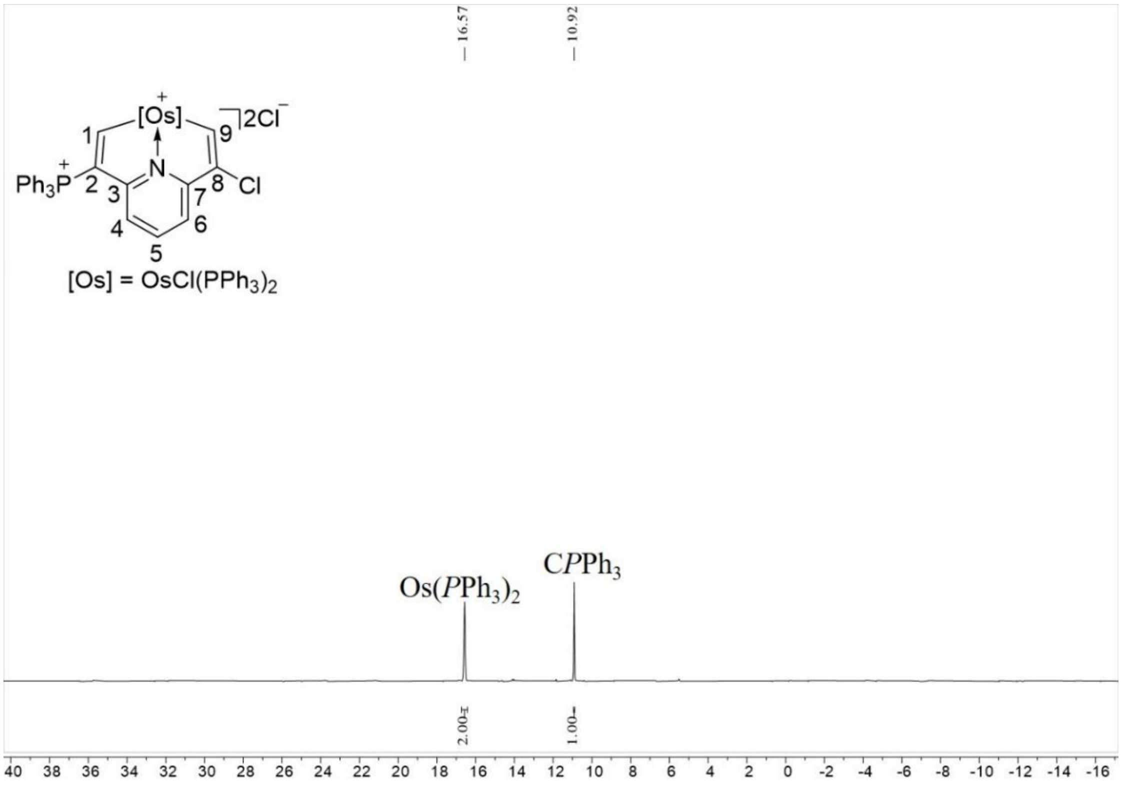

Figure S28. The $^{31}P\{^{1}H\}$ NMR (242.9 MHz, CD_2Cl_2) spectrum for complex **2**.

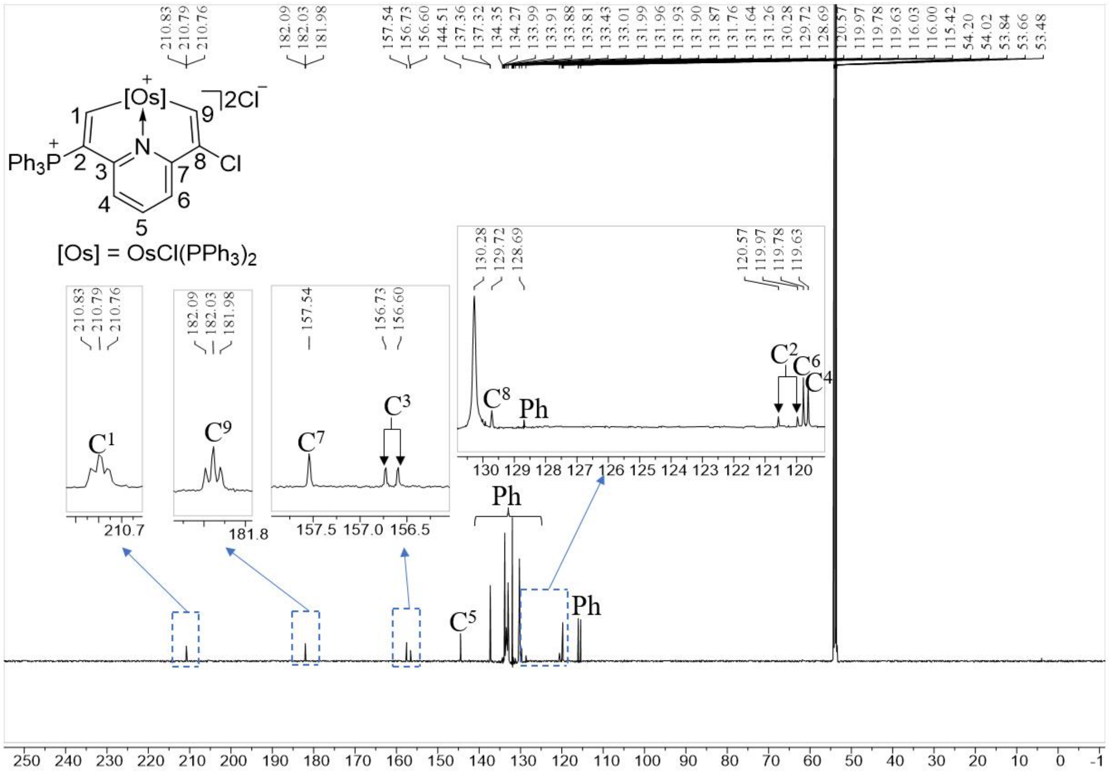

Figure S29. The $^{13}C\{^{1}H\}$ NMR (150.9 MHz, CD_2Cl_2) spectrum for complex **2**.

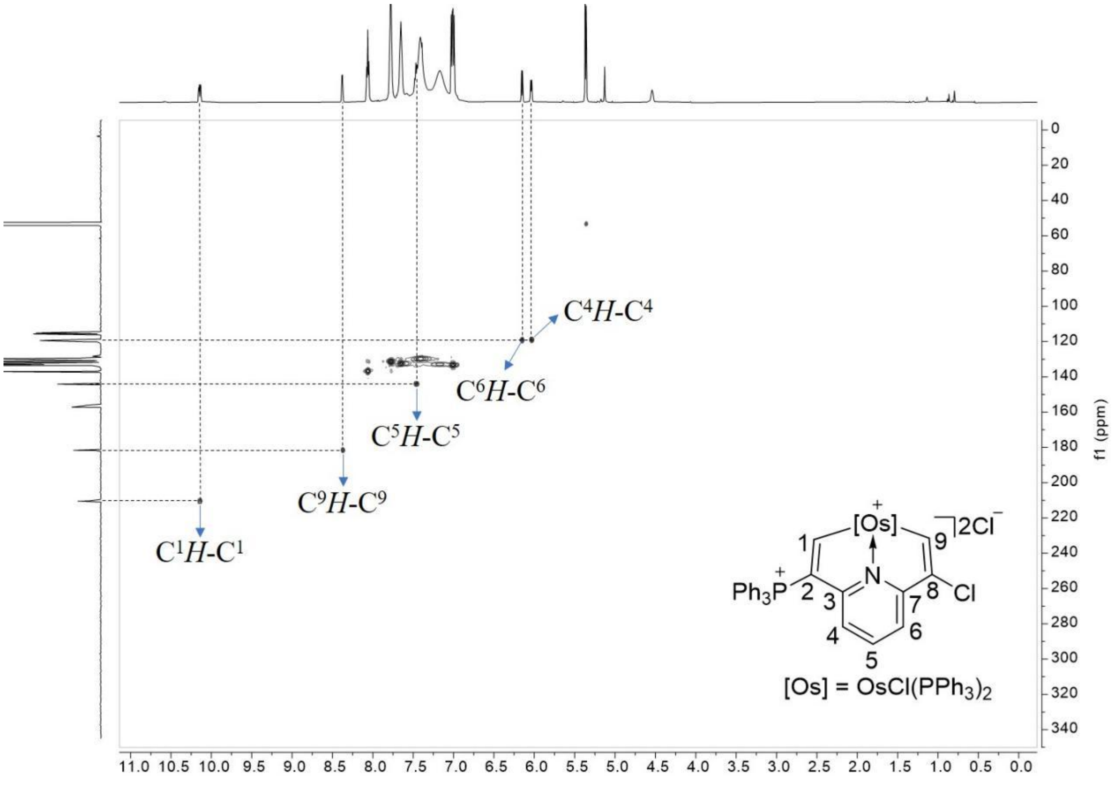

Figure S30. The ^{1}H-^{13}C HSQC (150.9 MHz, CD_2Cl_2) spectrum for complex **2**.

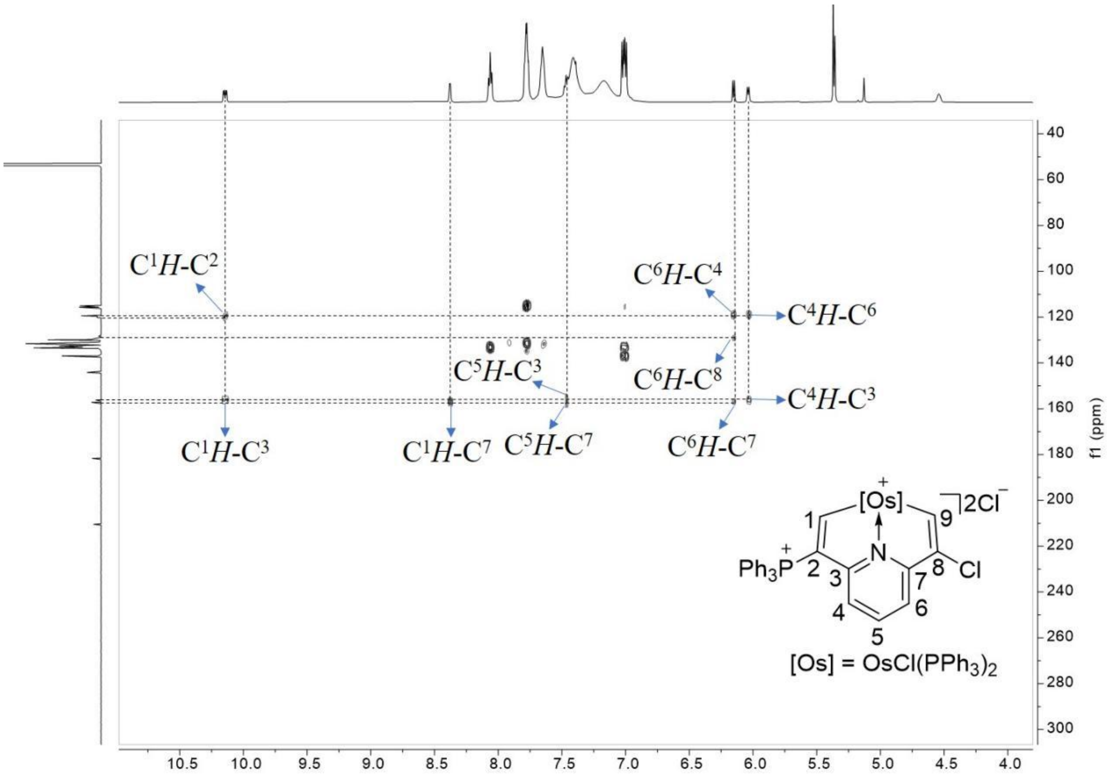

Figure S31. The ^{1}H-^{13}C HMBC (150.9 MHz, CD_2Cl_2) spectrum for complex **2**.

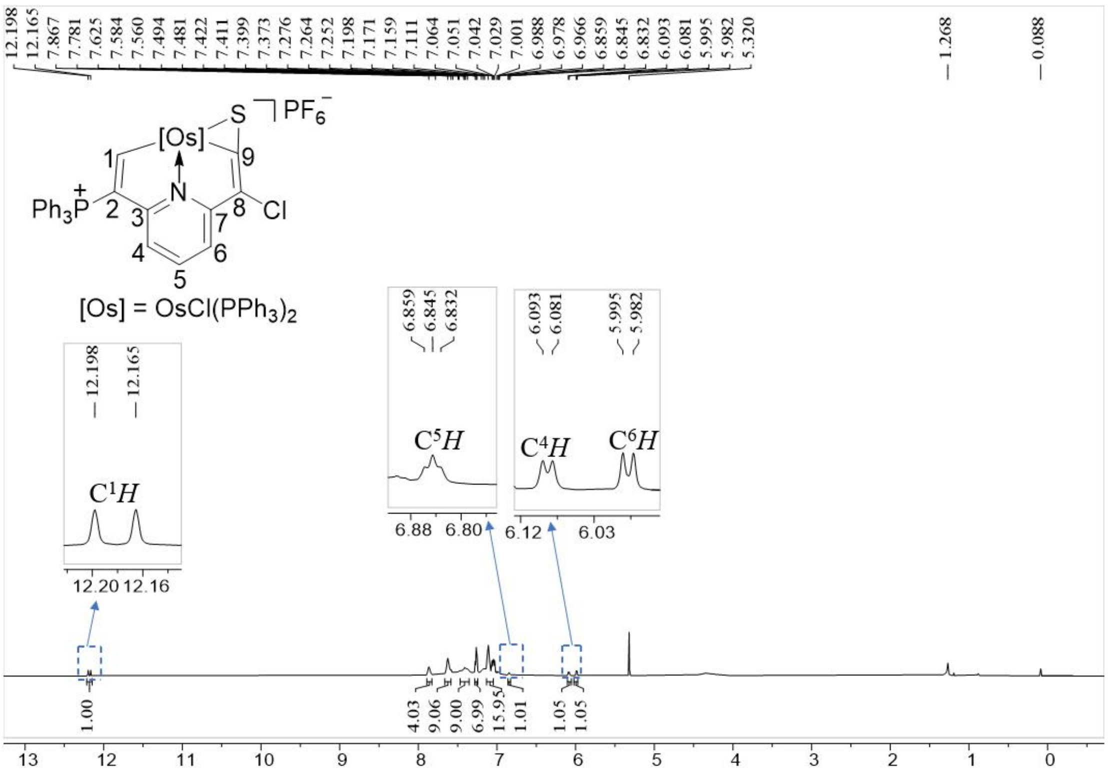

Figure S32. The ^{1}H NMR (600.1 MHz, CD_2Cl_2) spectrum for complex **3a**.

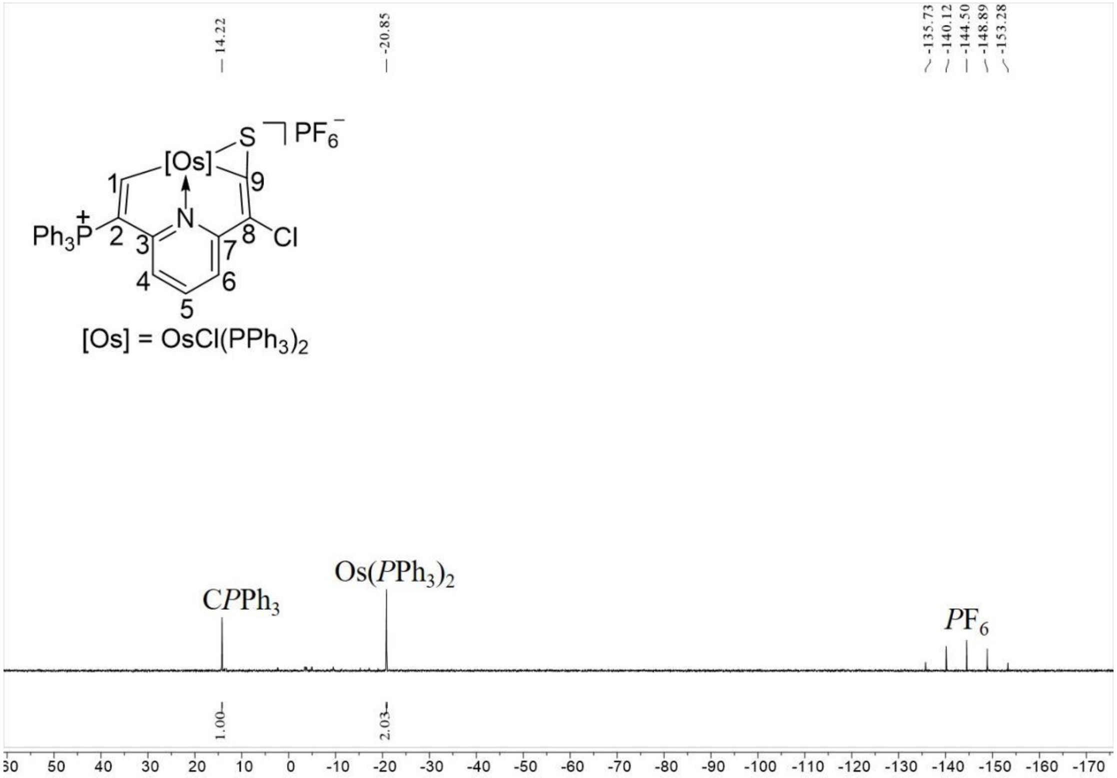

Figure S33. The $^{31}P\{^1H\}$ NMR (242.9 MHz, CD_2Cl_2) spectrum for complex **3a**.

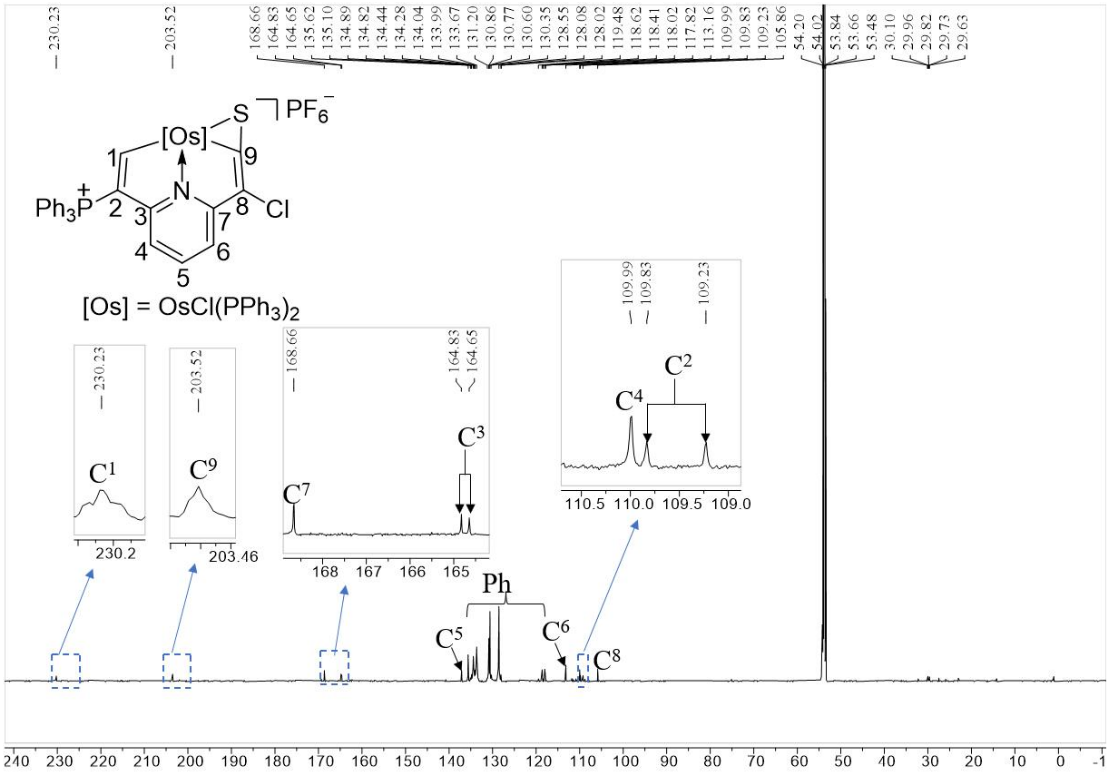

Figure S34. The $^{13}C\{^1H\}$ NMR (150.9 MHz, CD_2Cl_2) spectrum for complex **3a**.

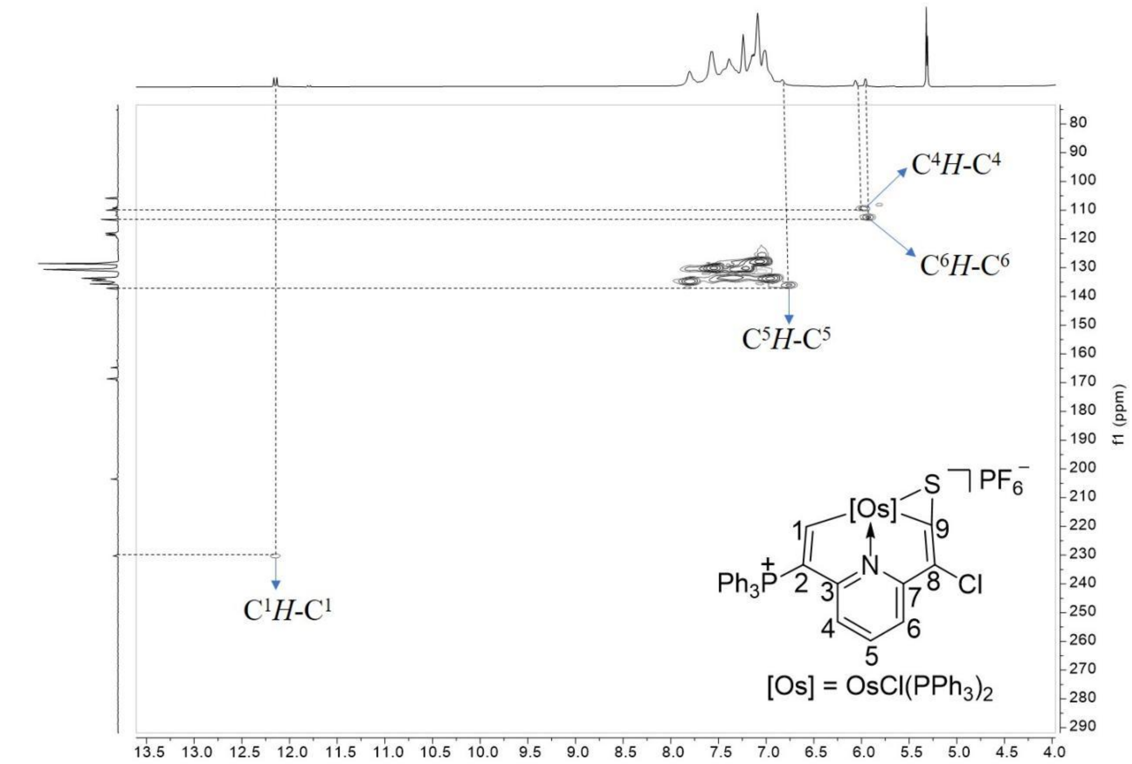

Figure S35. The ^{1}H-^{13}C HSQC (150.9 MHz, CD_2Cl_2) spectrum for complex **3a**.

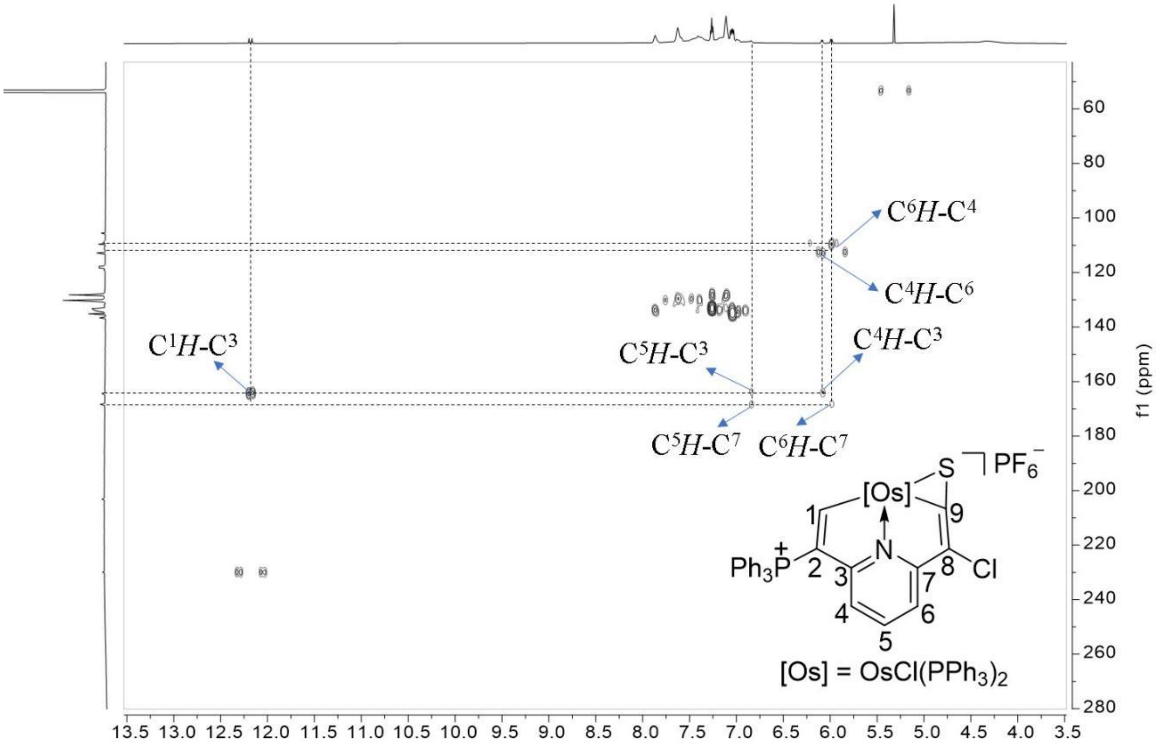

Figure S36. The ^{1}H-^{13}C HMBC (150.9 MHz, CD_2Cl_2) spectrum for complex **3a**.

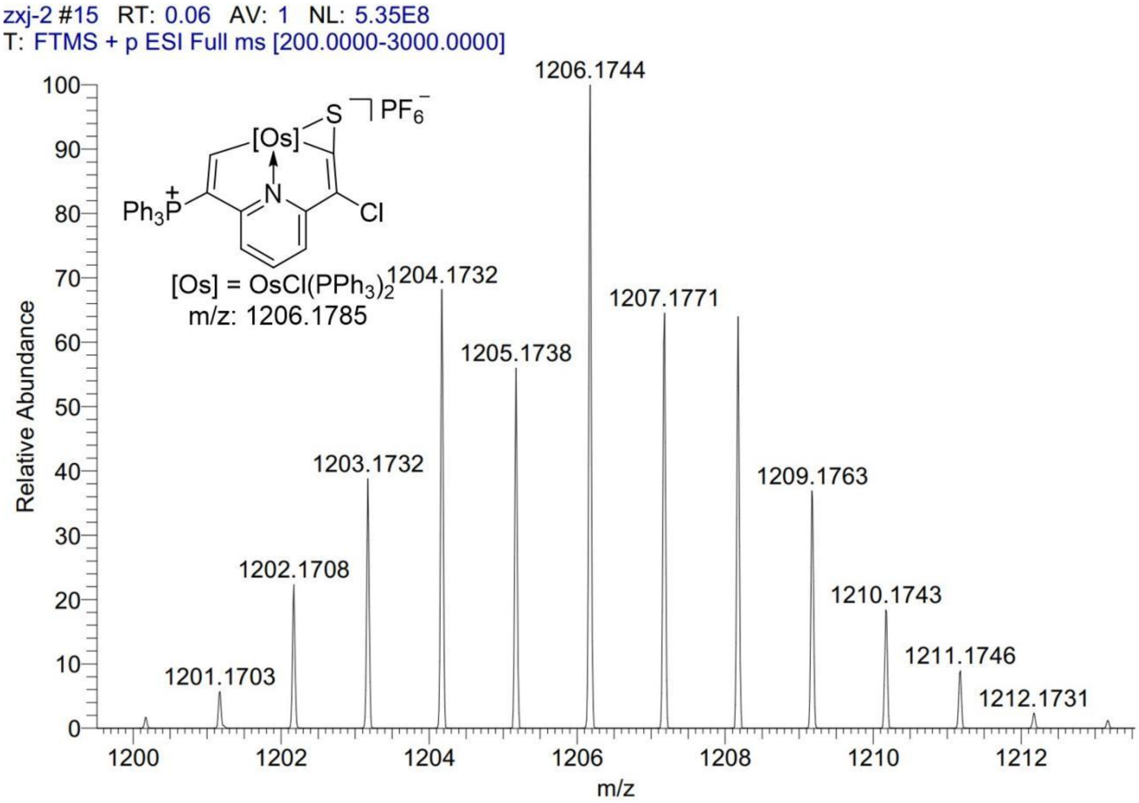

Figure S37. Positive-ion ESI-MS spectrum of [**3a**]$^+$ measured in methanol.

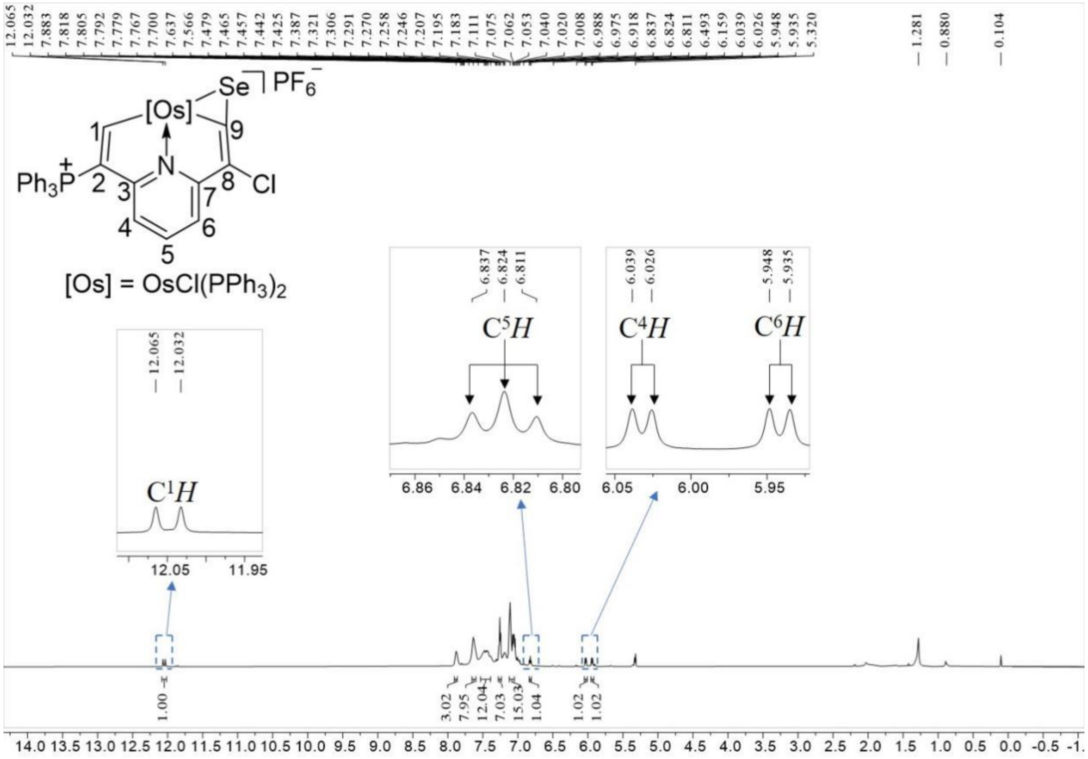

Figure S38. The ^1H NMR (600.1 MHz, CD_2Cl_2) spectrum for complex **3b**.

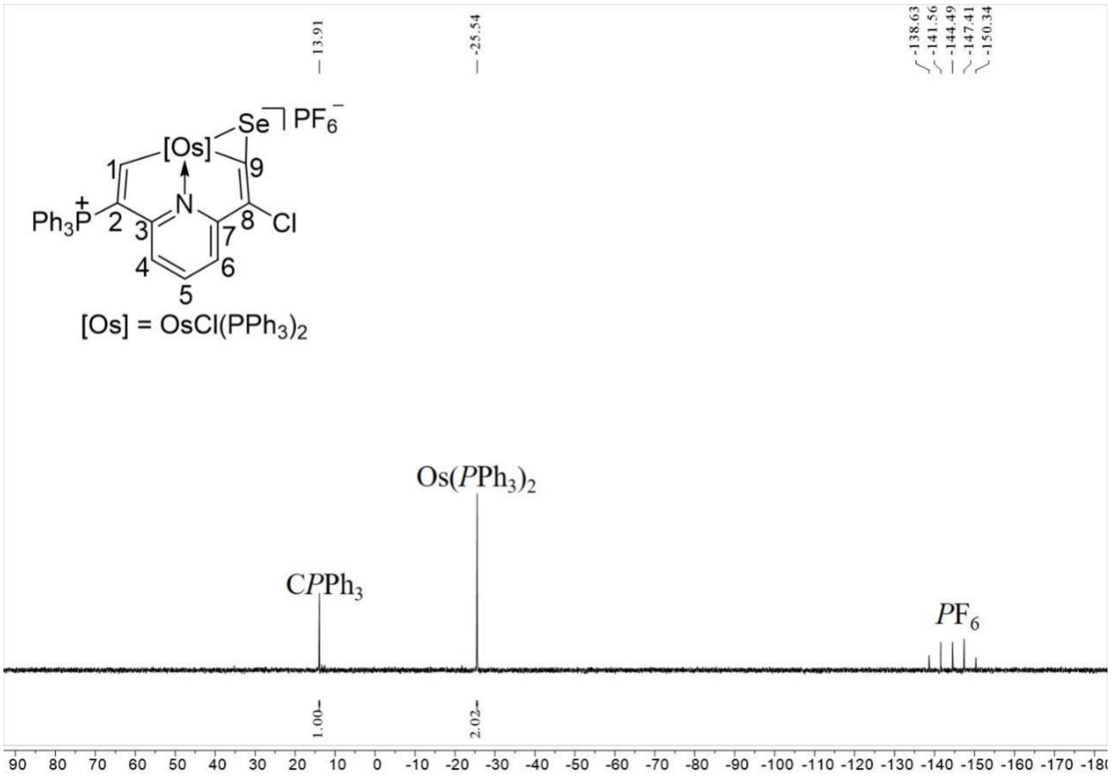

Figure S39. The $^{31}P\{^{1}H\}$ NMR (242.9 MHz, CD_2Cl_2) spectrum for complex **3b**.

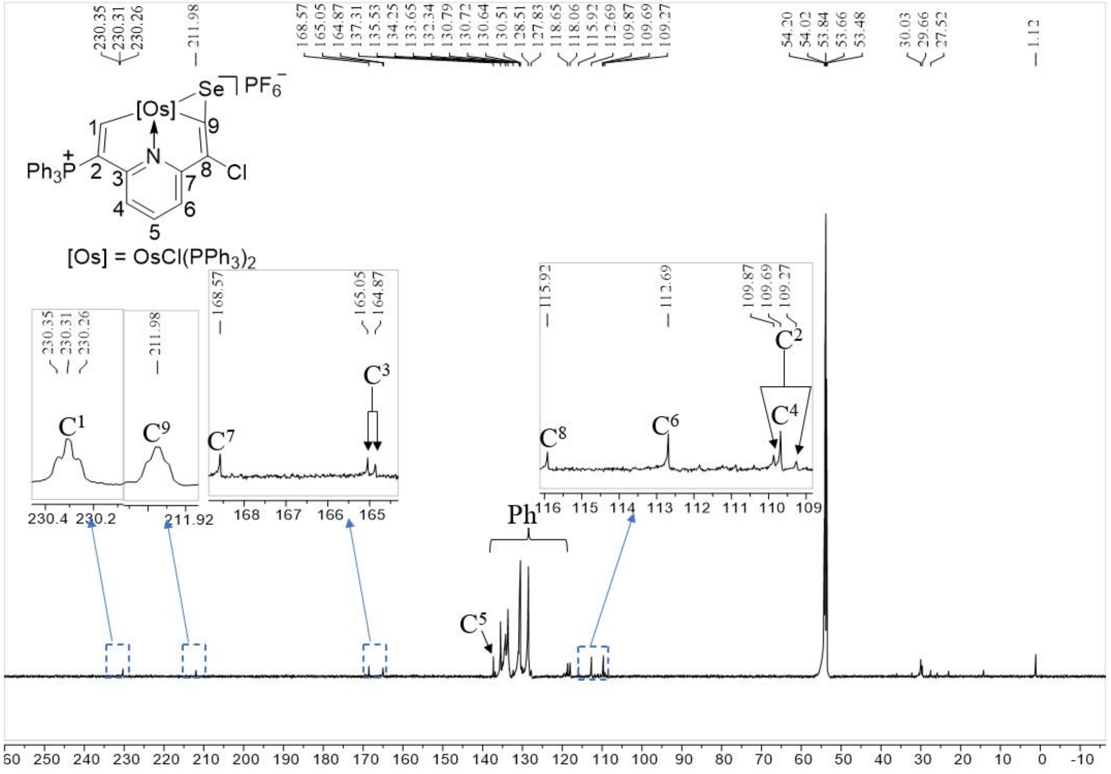

Figure S40. The $^{13}C\{^{1}H\}$ NMR (150.9 MHz, CD_2Cl_2) spectrum for complex **3b**.

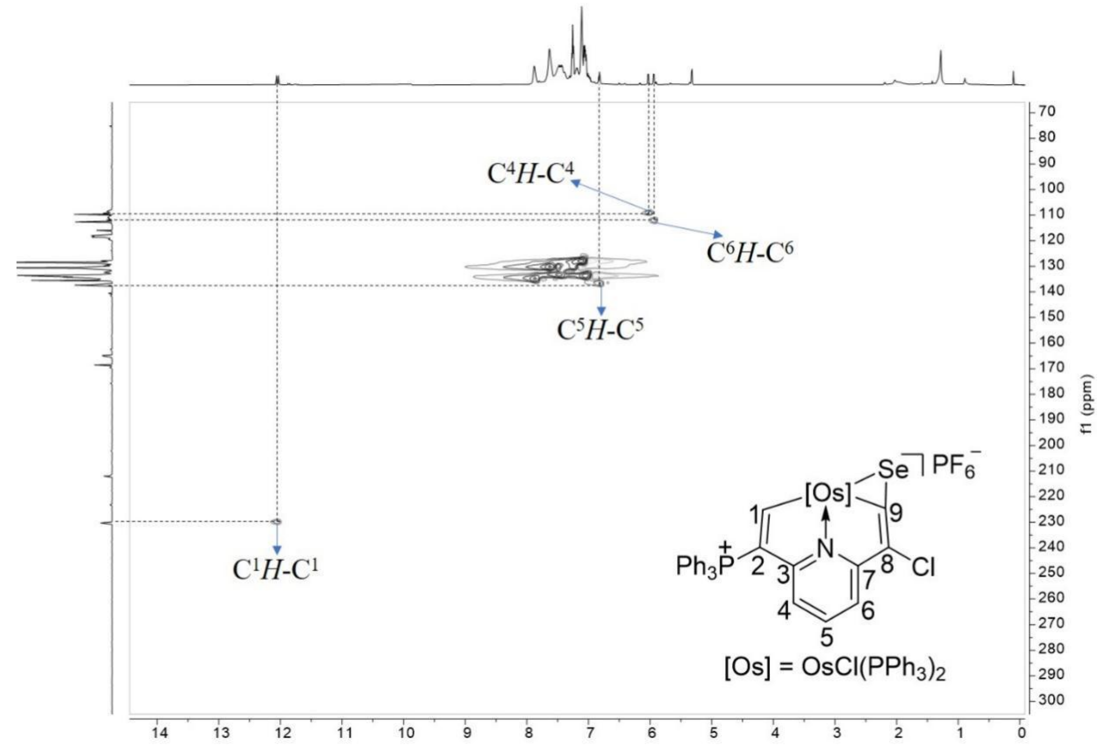

Figure S41. The ^{1}H-^{13}C HSQC (150.9 MHz, CD_2Cl_2) spectrum for complex **3b**.

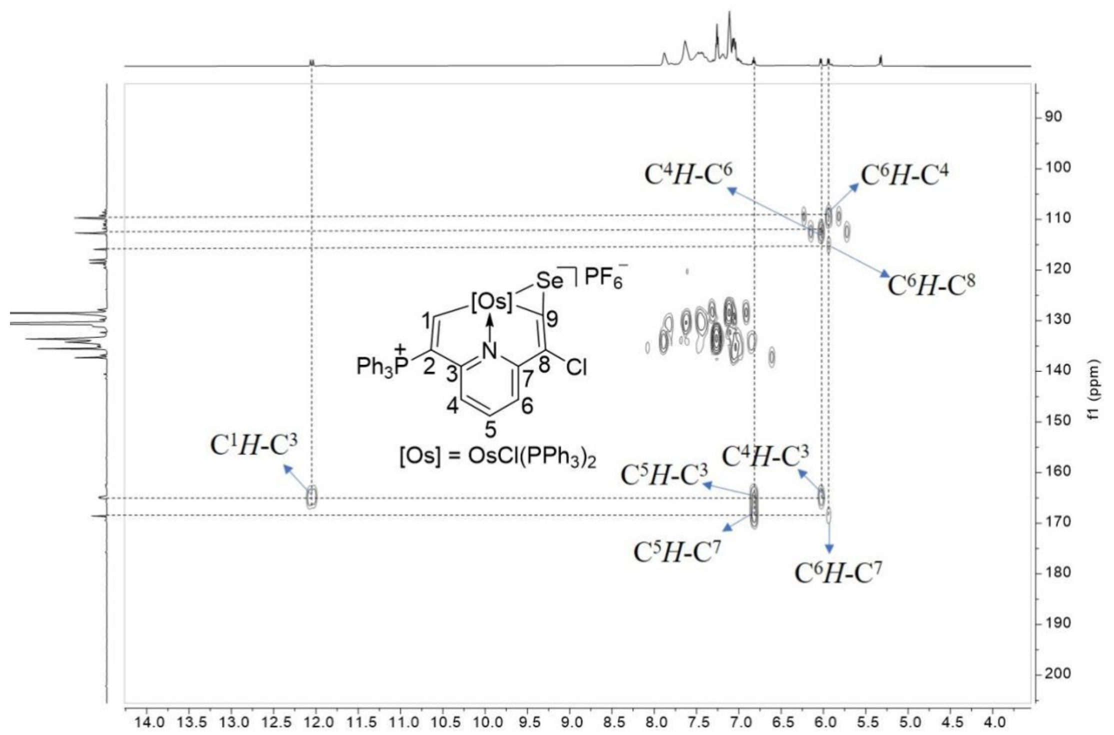

Figure S42. The ^{1}H-^{13}C HMBC (150.9 MHz, CD_2Cl_2) spectrum for complex **3b**.

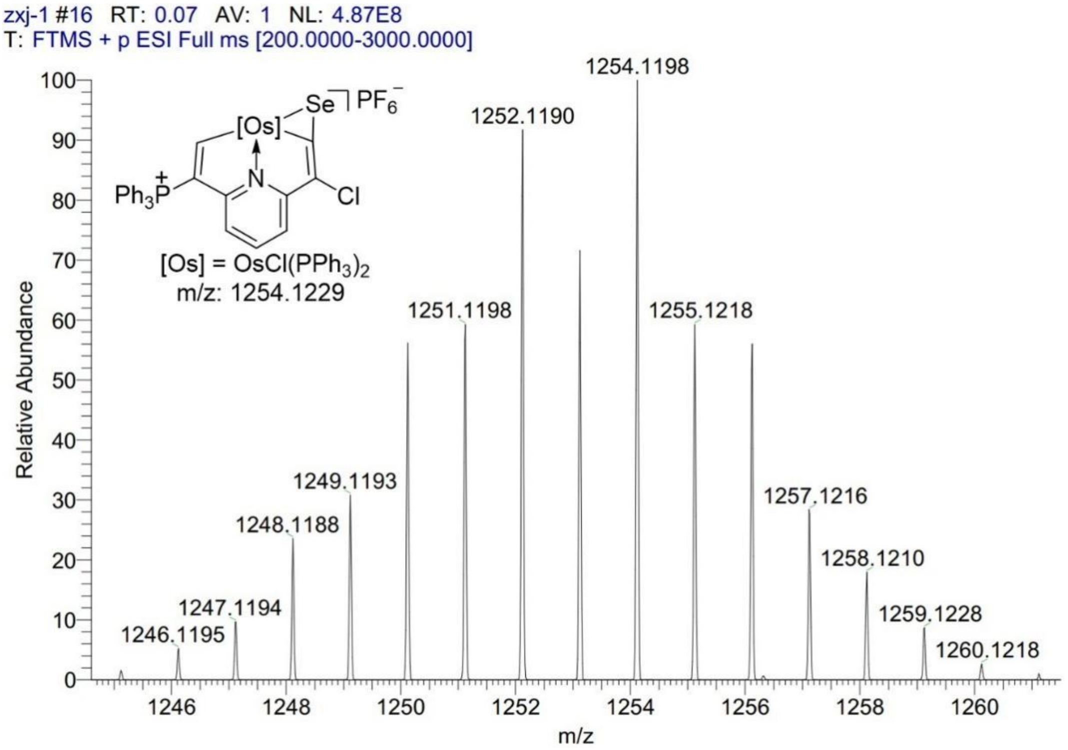

Figure 43. Positive-ion ESI-MS spectrum of [**3b**]$^+$ measured in methanol.

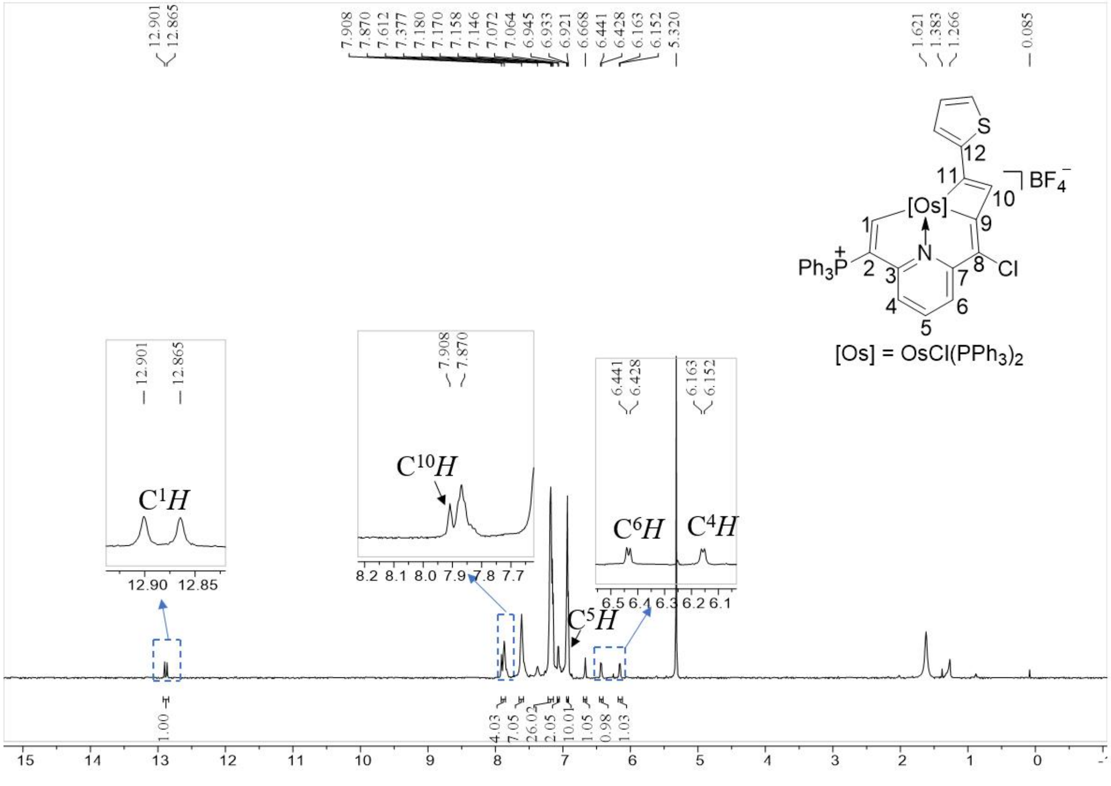

Figure S44. The ^{1}H NMR (600.1 MHz, CD_2Cl_2) spectrum for complex **4a**.

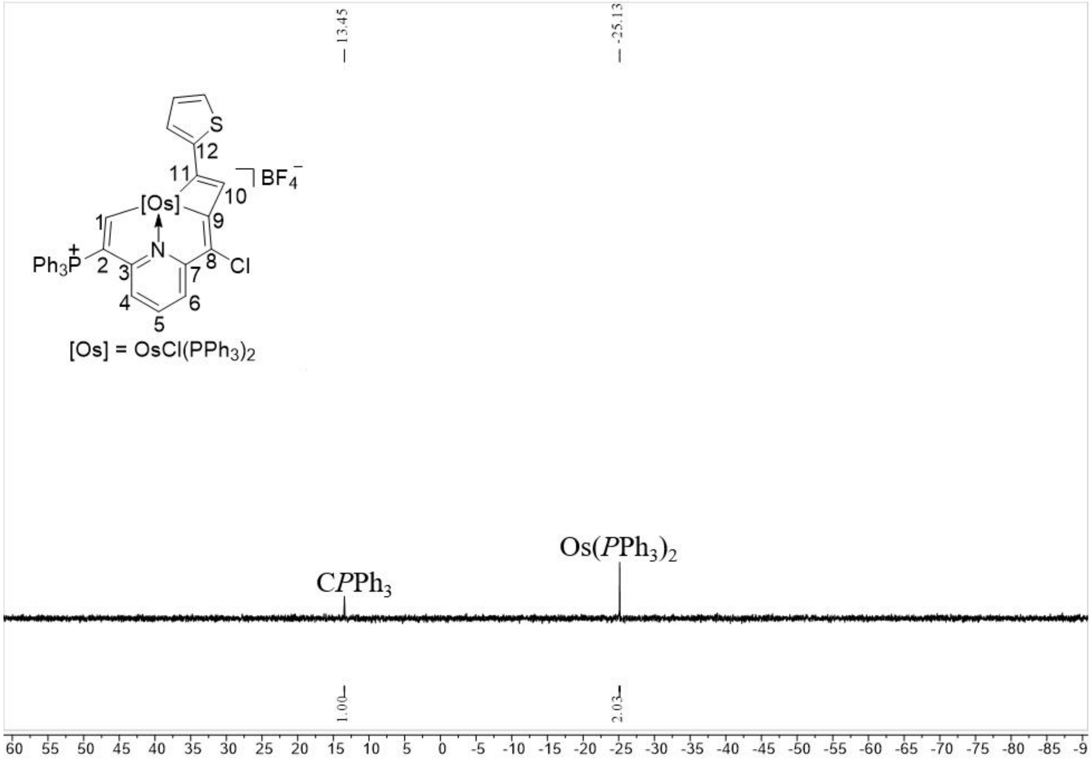

Figure S45. The $^{31}P\{^{1}H\}$ NMR (242.9 MHz, CD_2Cl_2) spectrum for complex **4a**.

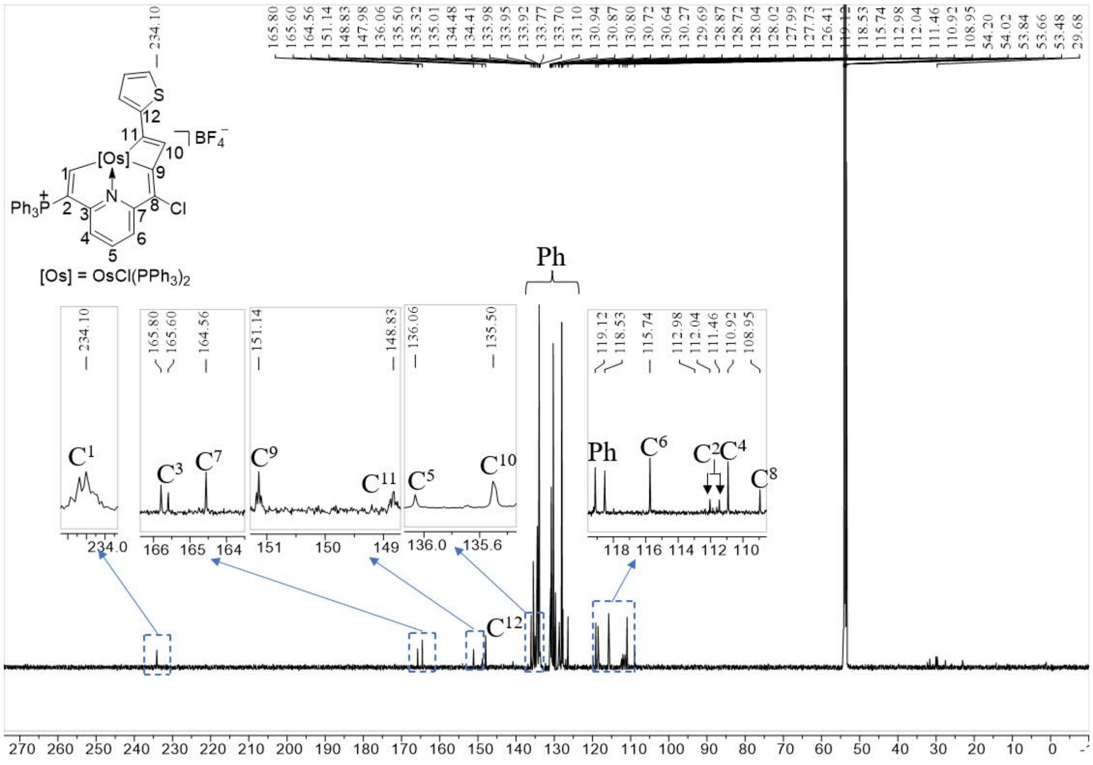

Figure S46. The $^{13}C\{^{1}H\}$ NMR (150.9 MHz, CD_2Cl_2) spectrum for complex **4a**.

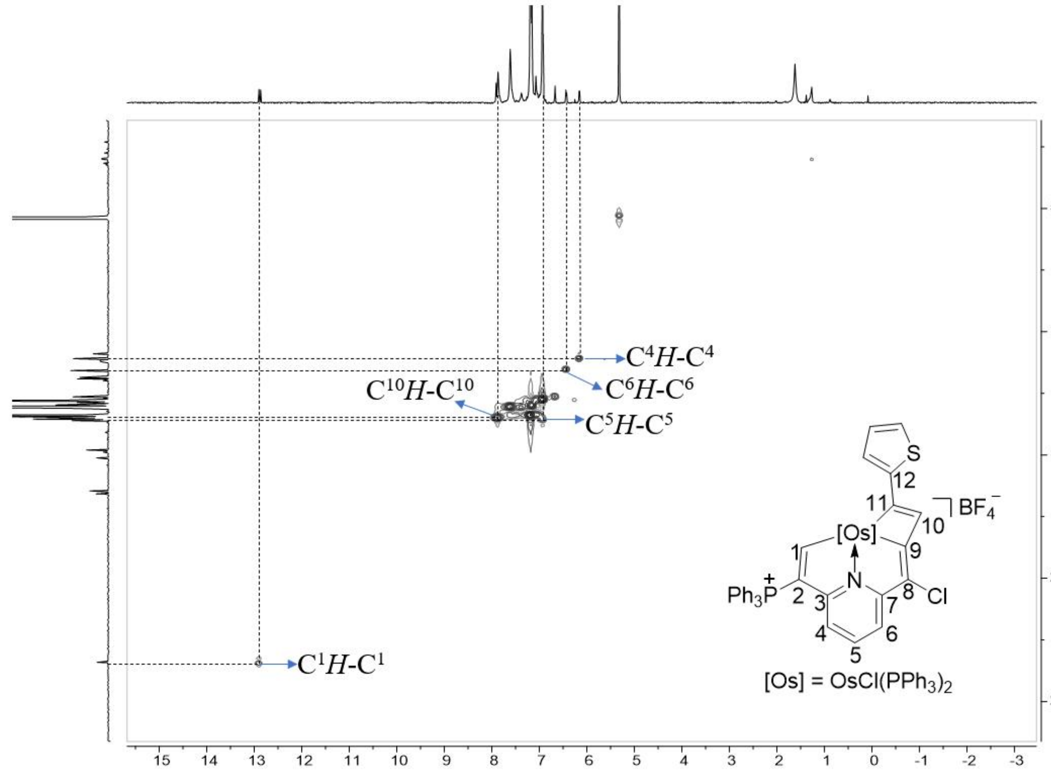

Figure S47. The ^{1}H-^{13}C HSQC (150.9 MHz, CD_2Cl_2) spectrum for complex **4a**.

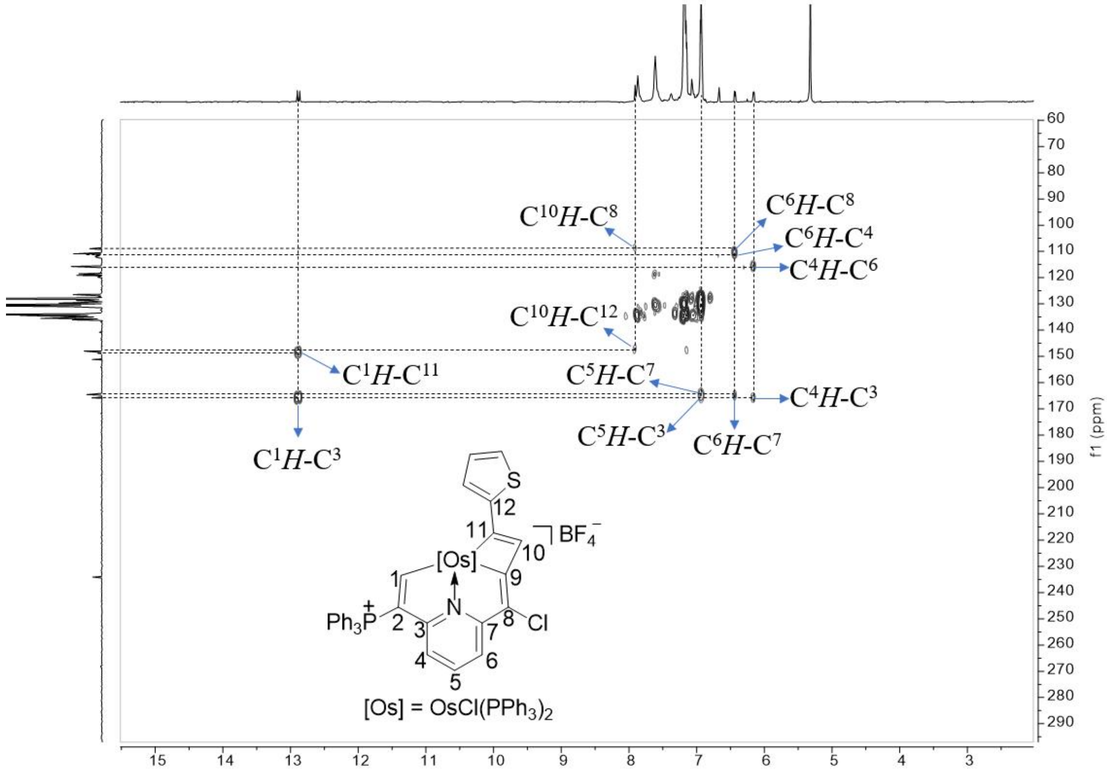

Figure S48. The ^{1}H-^{13}C HMBC (150.9 MHz, CD_2Cl_2) spectrum for complex **4a**.

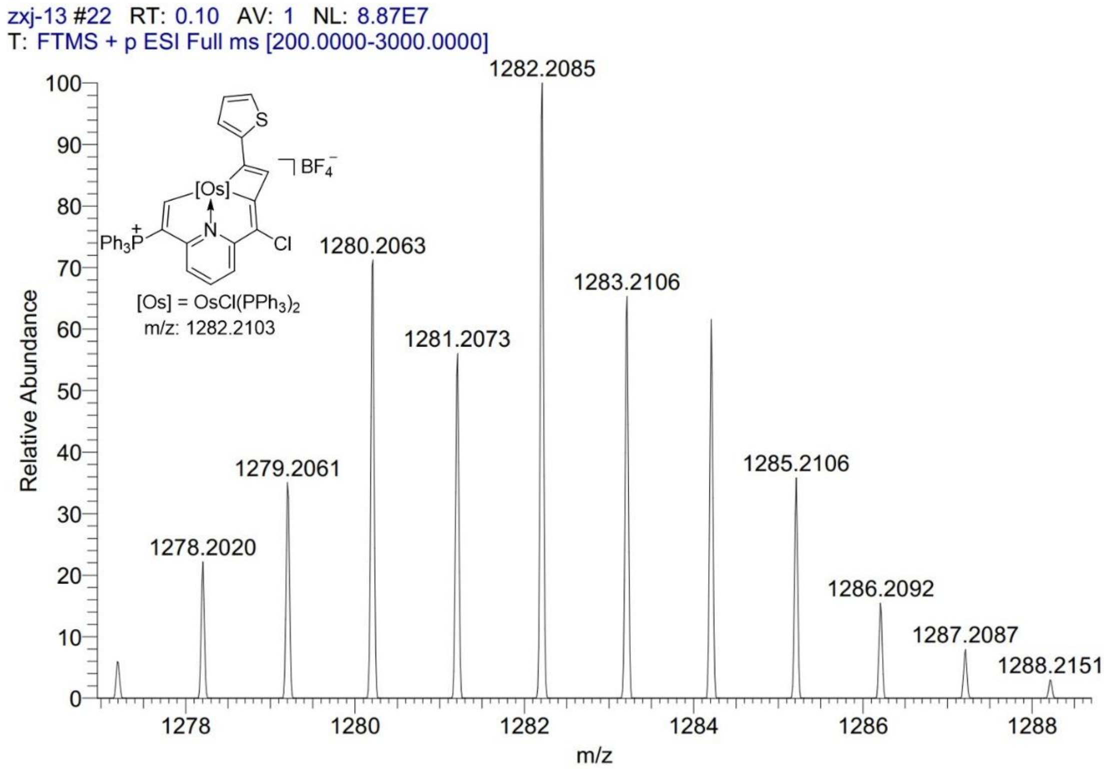

Figure S49. Positive-ion ESI-MS spectrum of [**4a**]$^+$ measured in methanol.

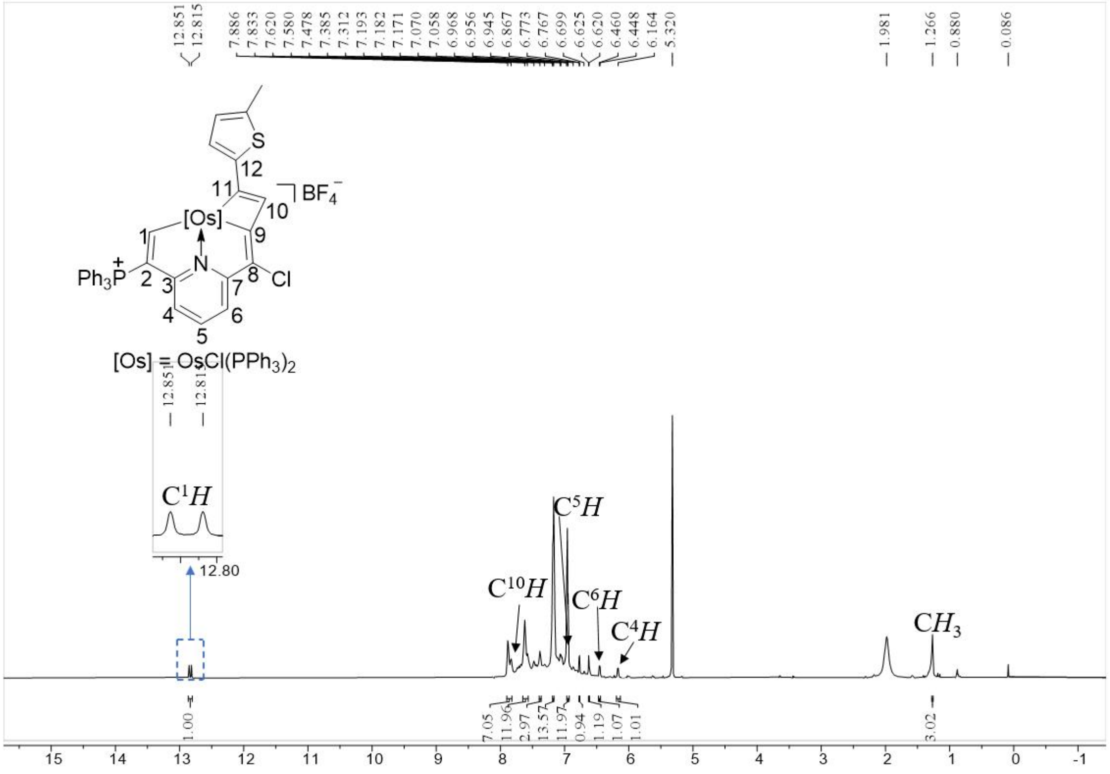

Figure S50. The ^{1}H NMR (600.1 MHz, CD_2Cl_2) spectrum for complex **4b**.

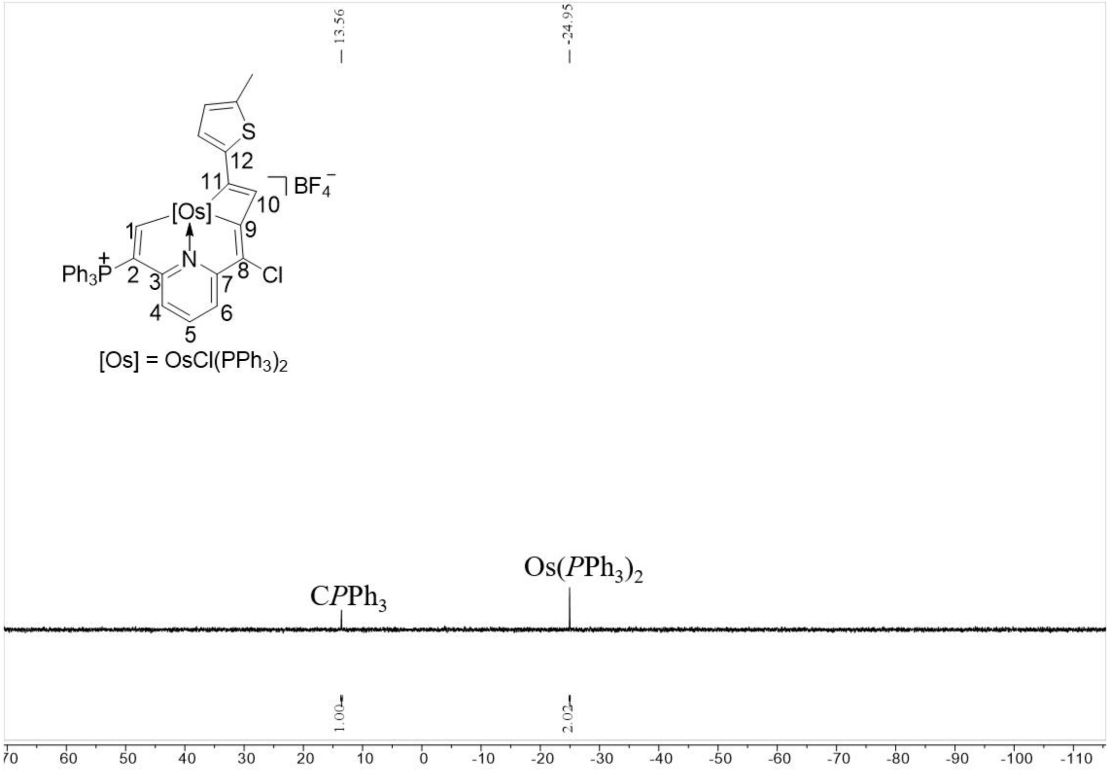

Figure S51. The $^{31}P\{^{1}H\}$ NMR (242.9 MHz, CD_2Cl_2) spectrum for complex **4b**.

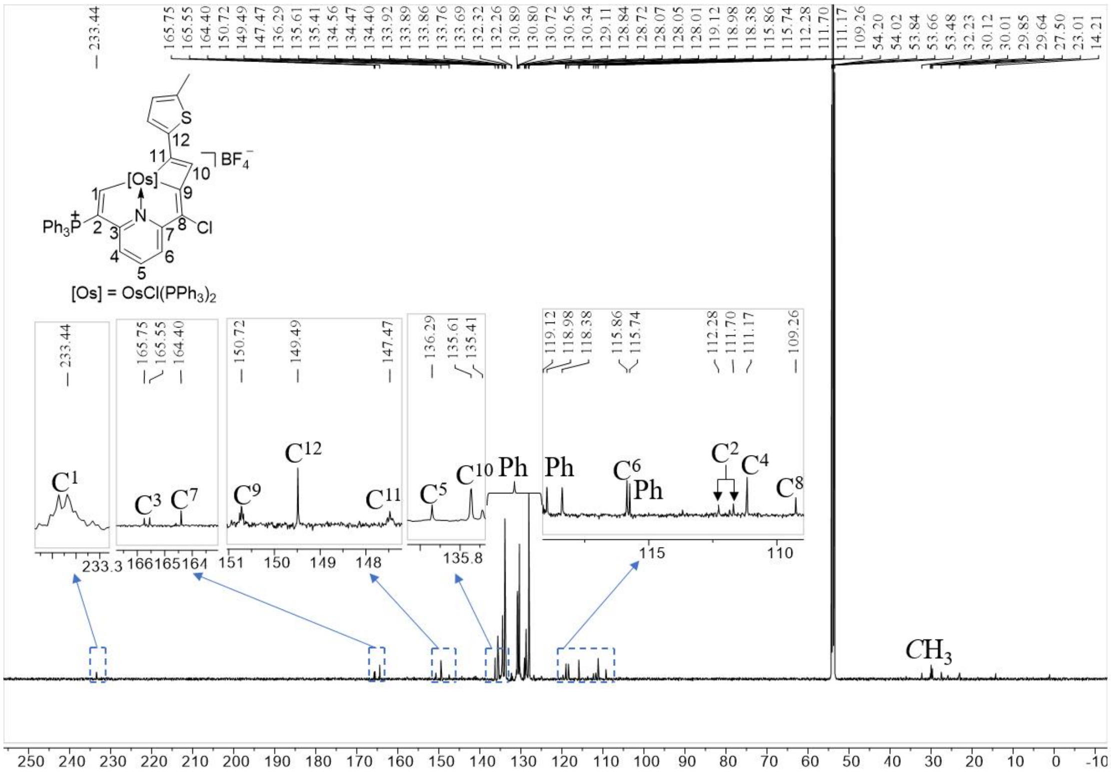

Figure S52. The $^{13}C\{^{1}H\}$ NMR (150.9 MHz, CD_2Cl_2) spectrum for complex **4b**.

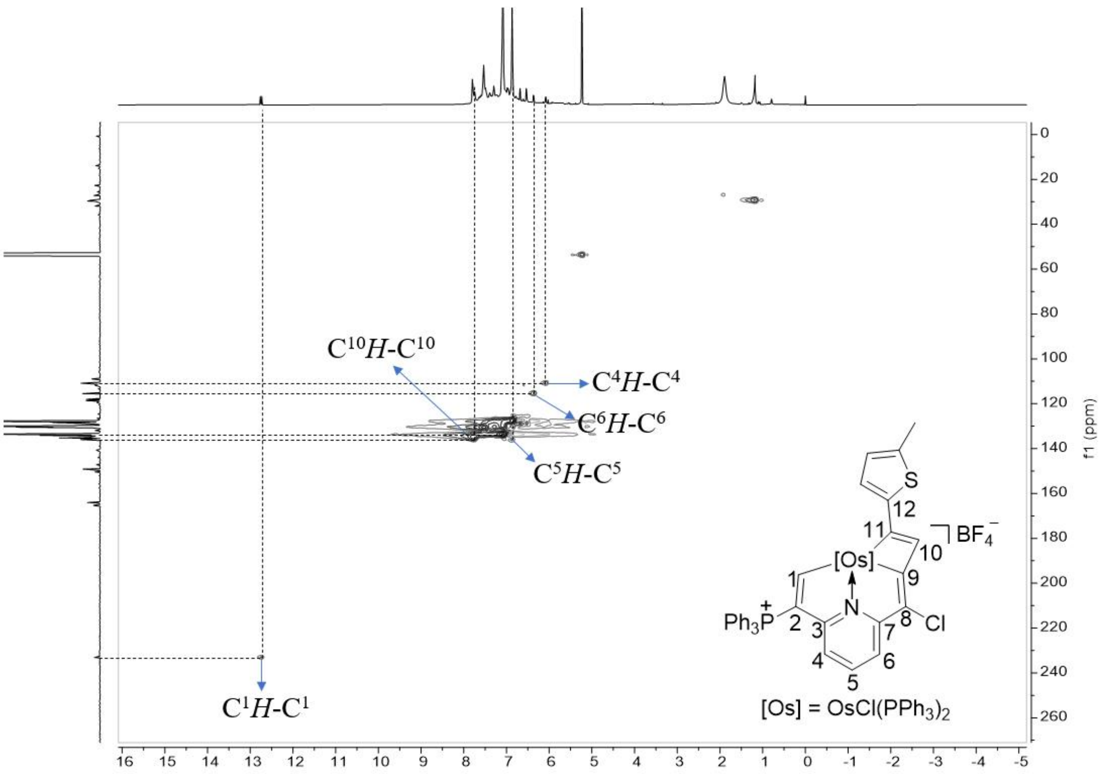

Figure S53. The ^{1}H-^{13}C HSQC (150.9 MHz, CD_2Cl_2) spectrum for complex **4b**.

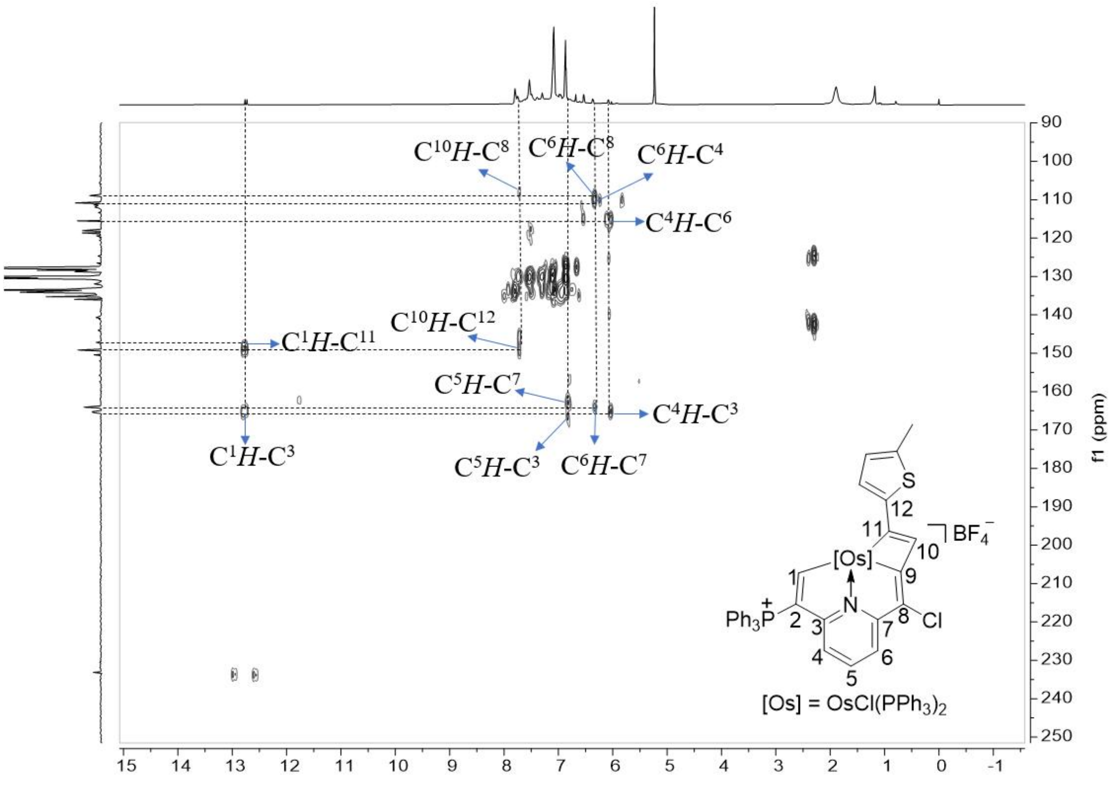

Figure S54. The ^{1}H-^{13}C HMBC (150.9 MHz, CD_2Cl_2) spectrum for complex **4b**.

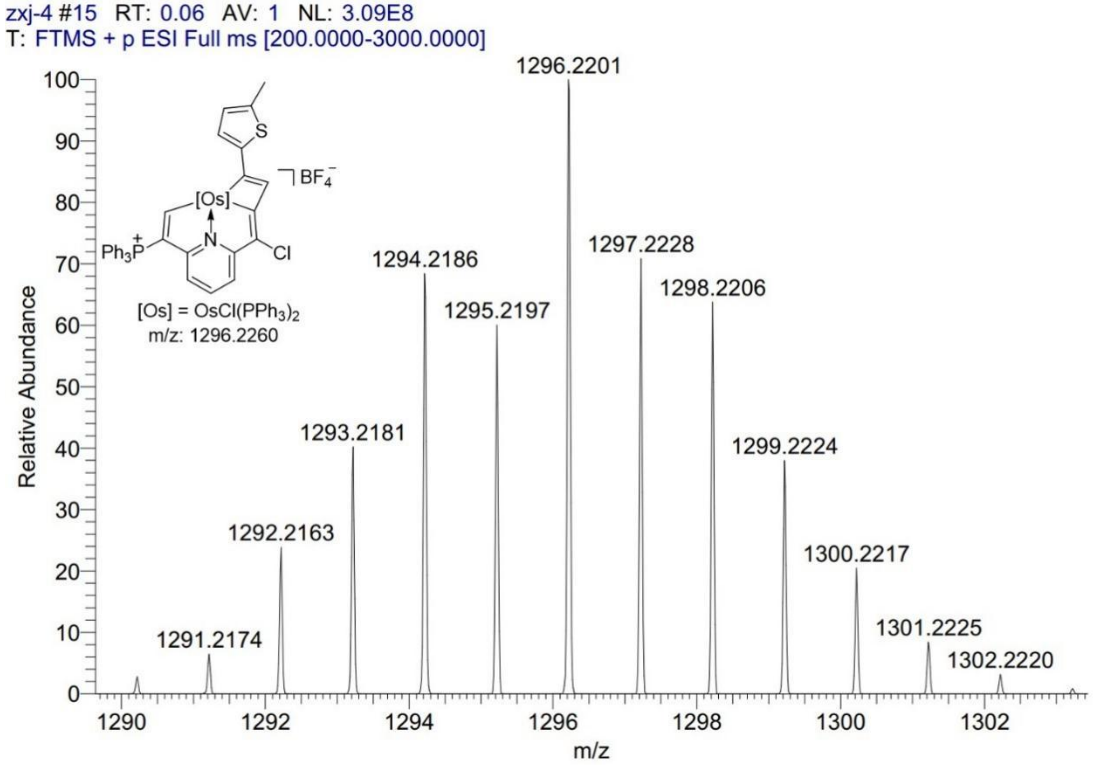

Figure 55. Positive-ion ESI-MS spectrum of [**4b**]$^+$ measured in methanol.

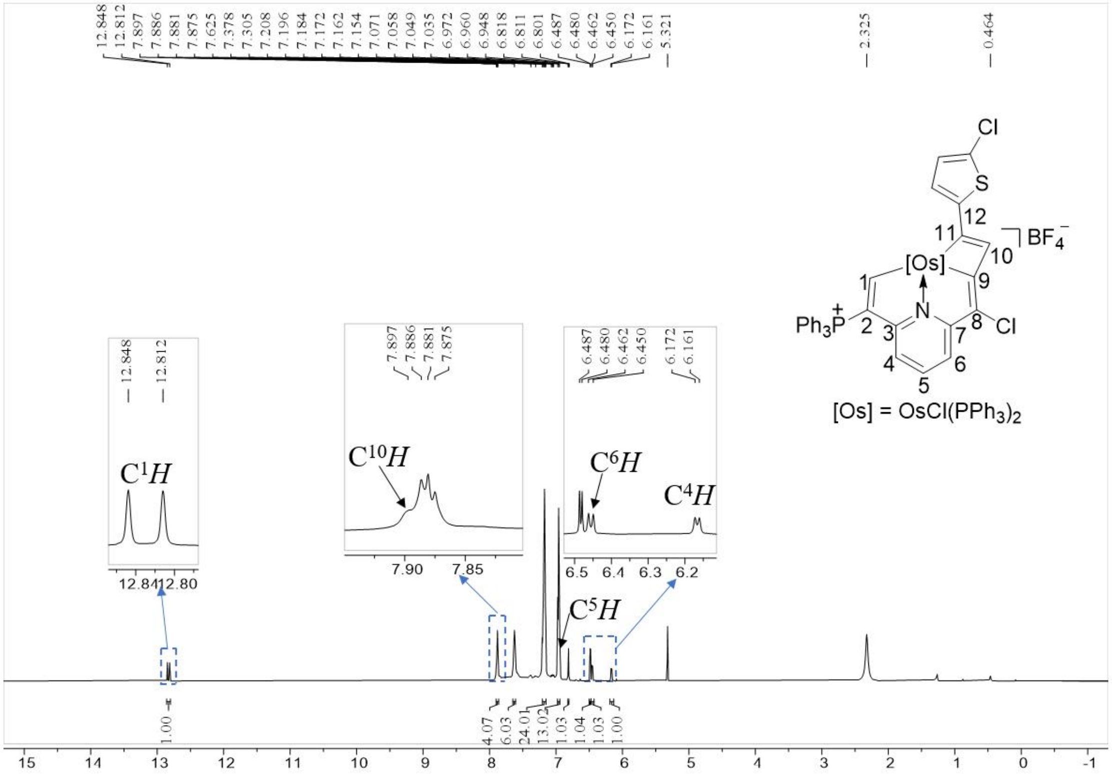

Figure S56. The ^{1}H NMR (600.1 MHz, CD_2Cl_2) spectrum for complex **4c**.

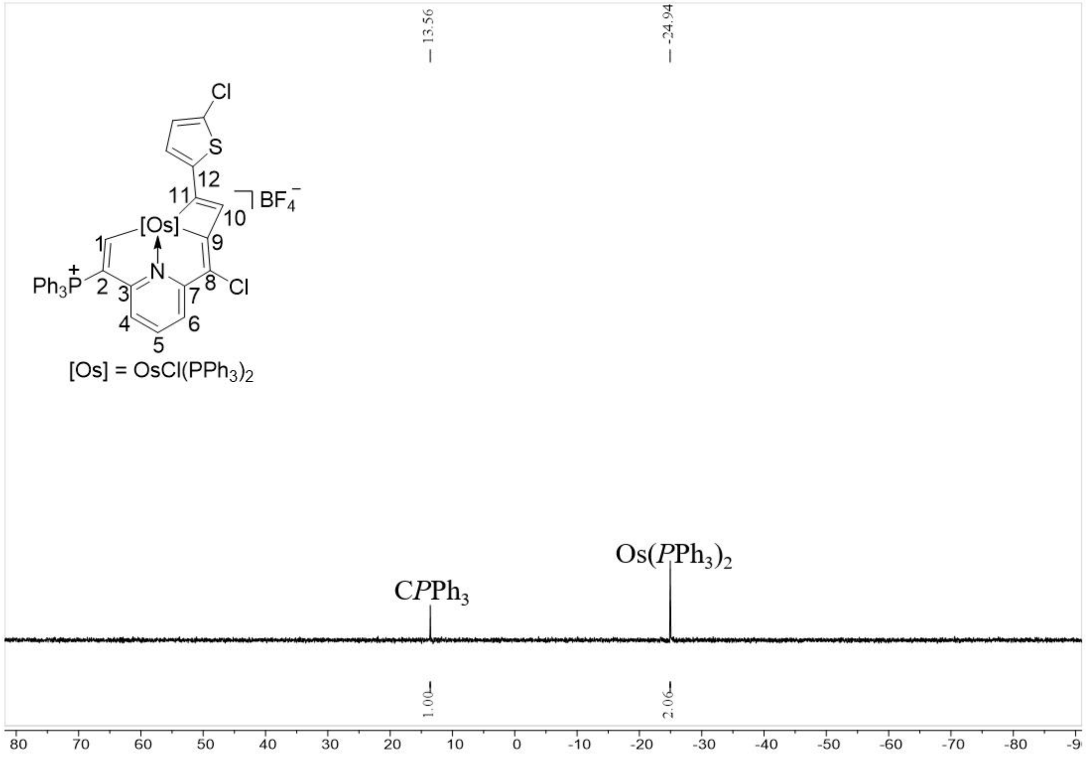

Figure S57. The $^{31}P\{^{1}H\}$ NMR (242.9 MHz, CD_2Cl_2) spectrum for complex **4c**.

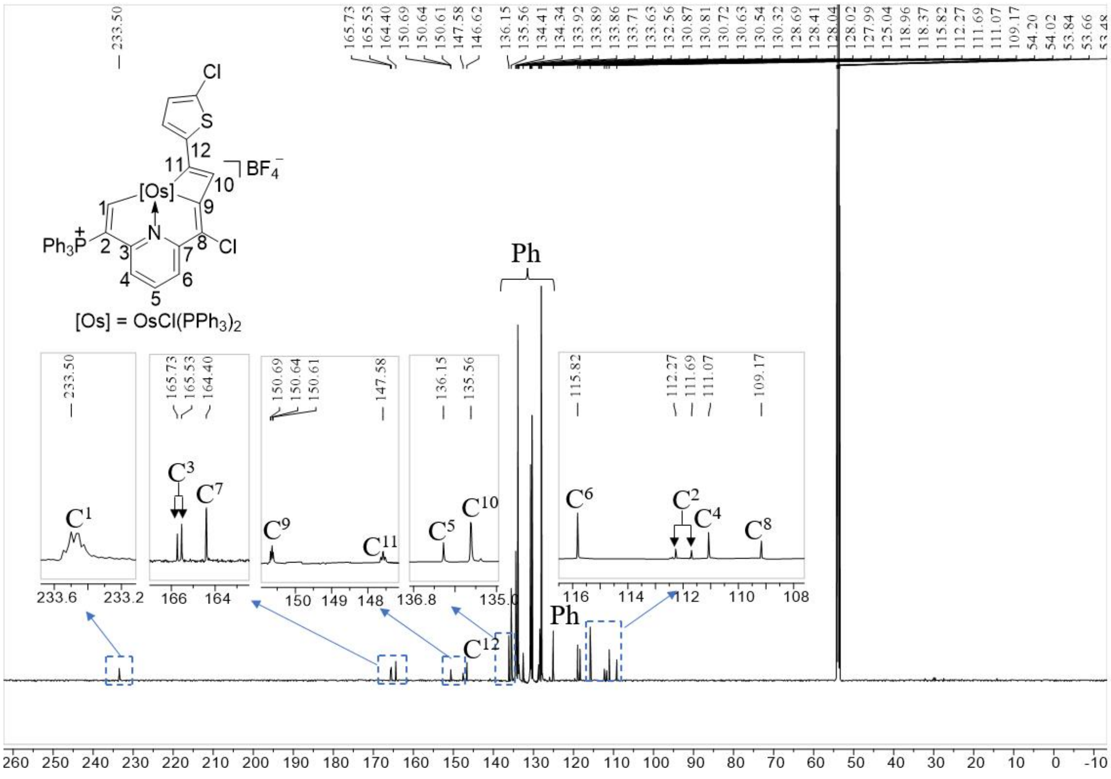

Figure S58. The $^{13}C\{^{1}H\}$ NMR (150.9 MHz, CD_2Cl_2) spectrum for complex **4c**.

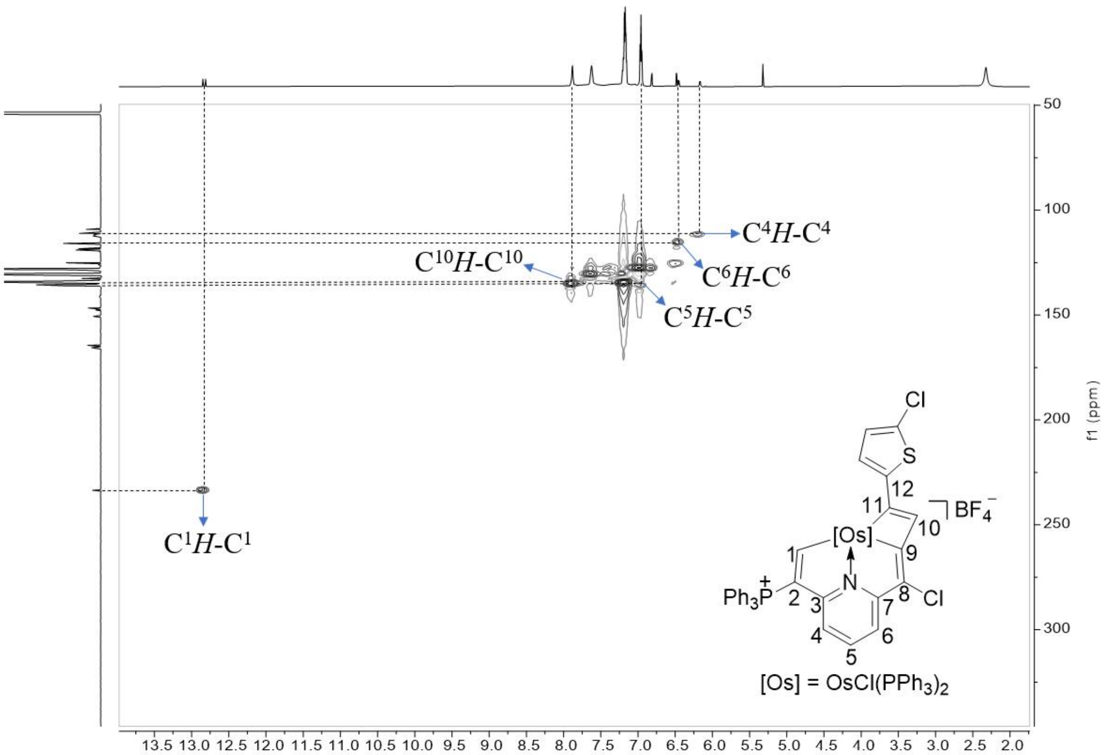

Figure S59. The ^{1}H-^{13}C HSQC (150.9 MHz, CD_2Cl_2) spectrum for complex **4c**.

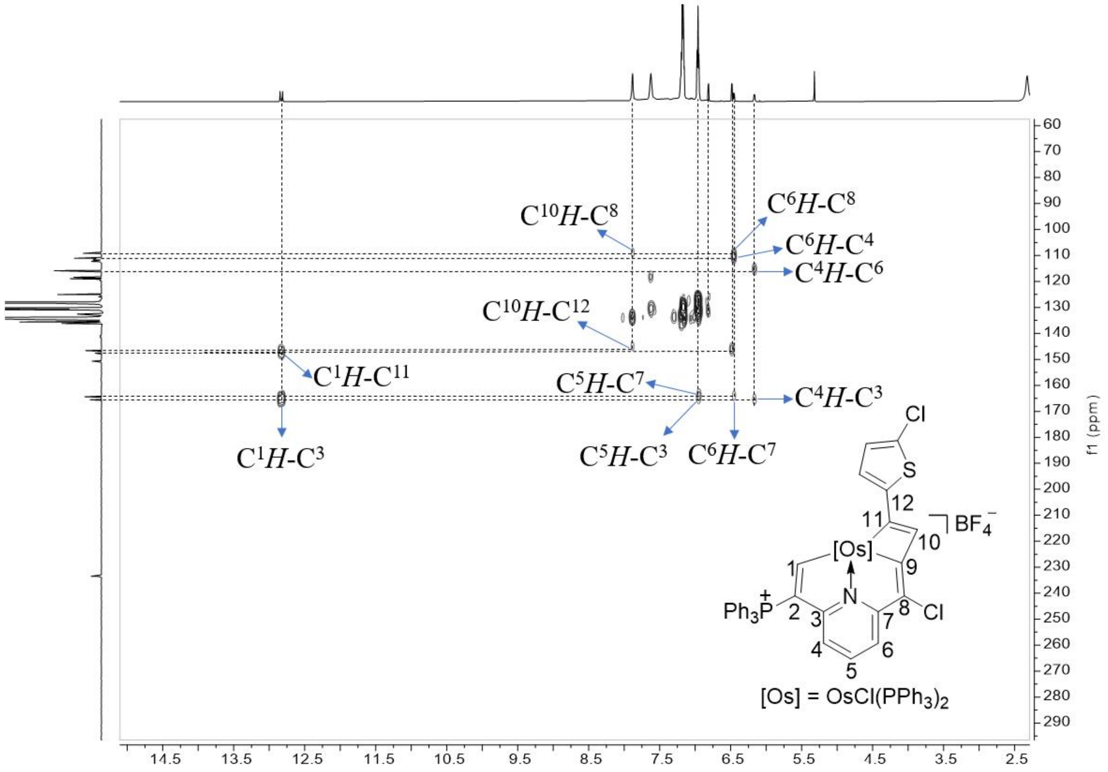

Figure S60. The ^{1}H-^{13}C HMBC (150.9 MHz, CD_2Cl_2) spectrum for complex **4c**.

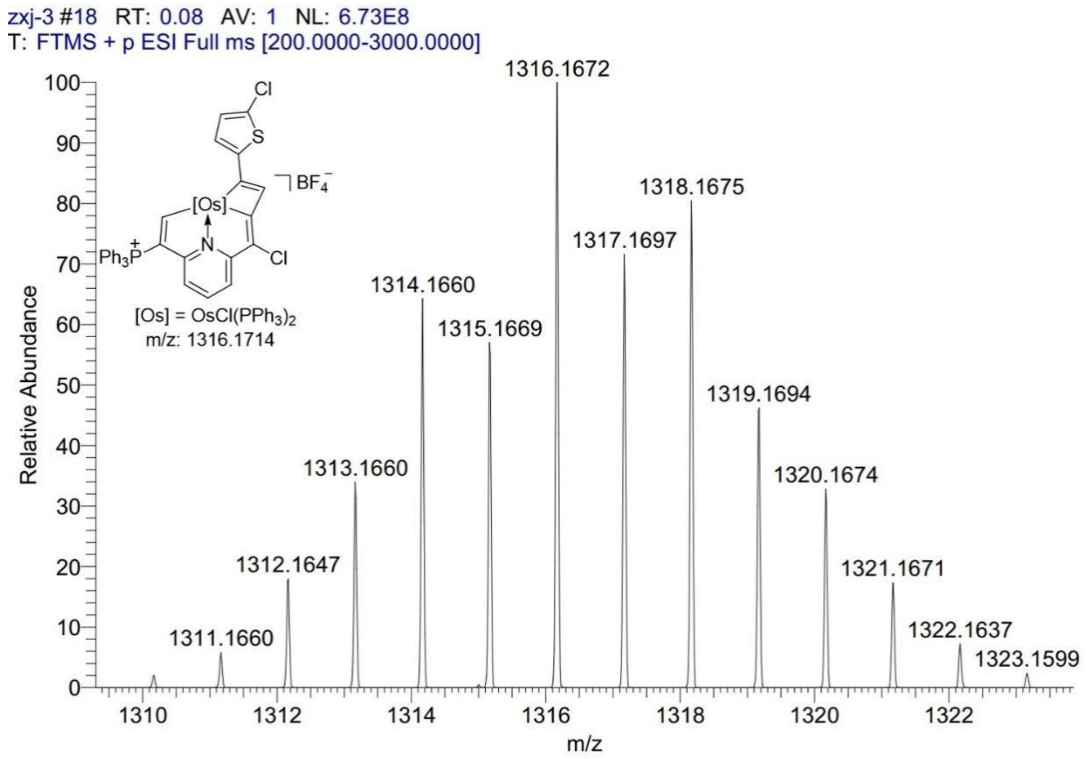

Figure S61. Positive-ion ESI-MS spectrum of [**4c**]$^+$ measured in methanol.

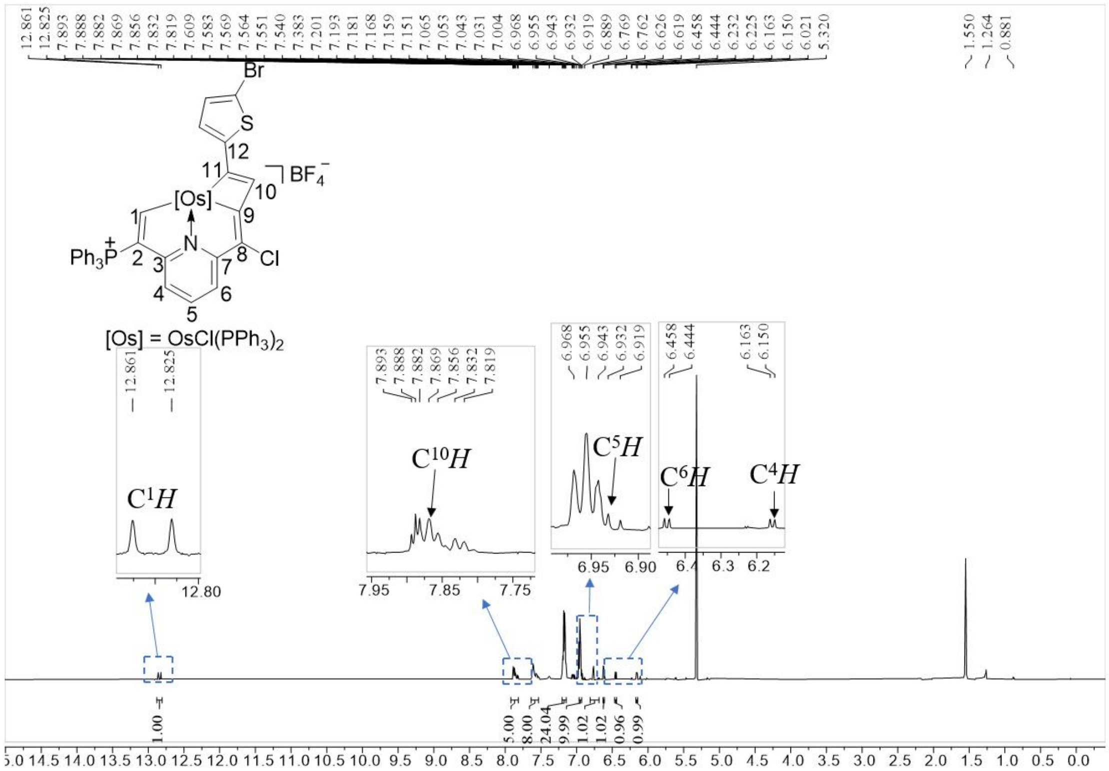

Figure S62. The ^{1}H NMR (600.1 MHz, CD_2Cl_2) spectrum for complex **4d**.

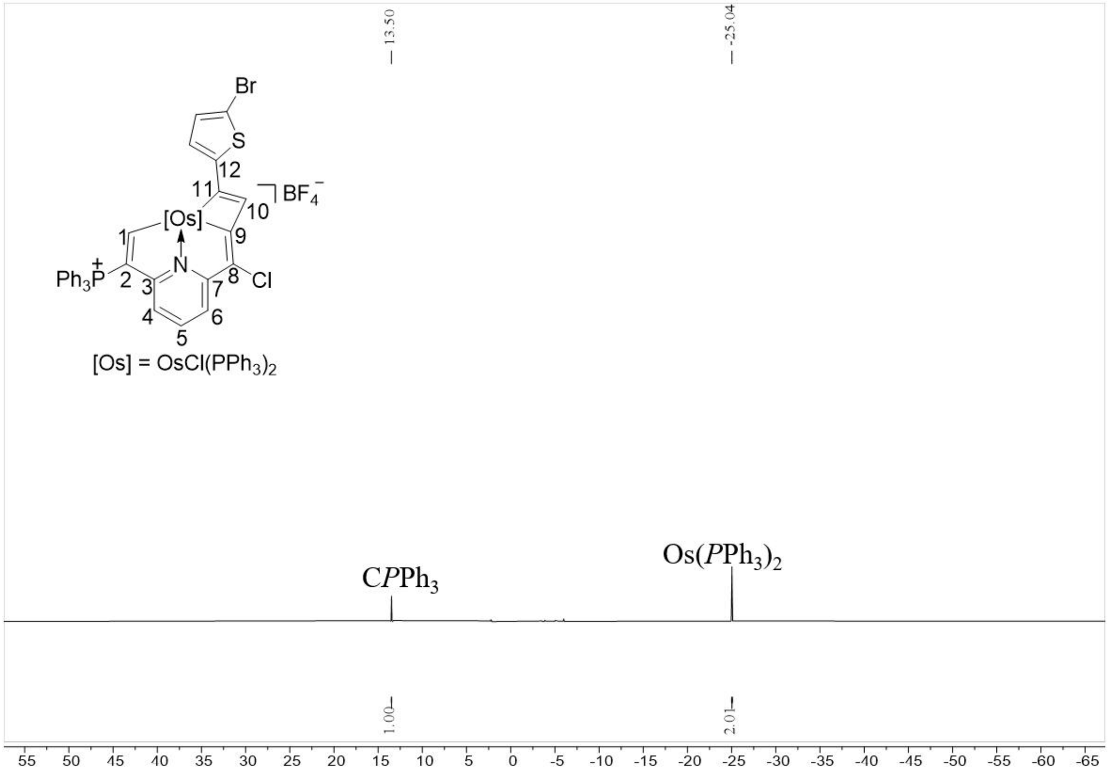

Figure S63. The $^{31}P\{^1H\}$ NMR (242.9 MHz, CD_2Cl_2) spectrum for complex **4d**.

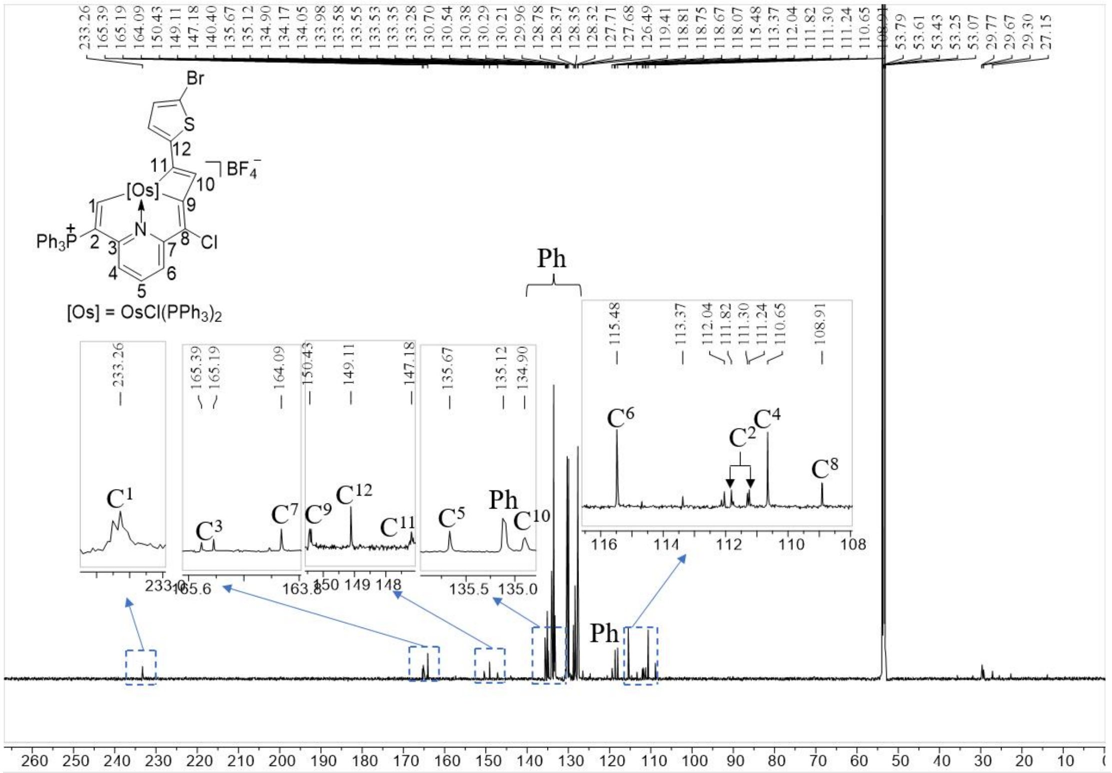

Figure S64. The $^{13}C\{^1H\}$ NMR (150.9 MHz, CD_2Cl_2) spectrum for complex **4d**.

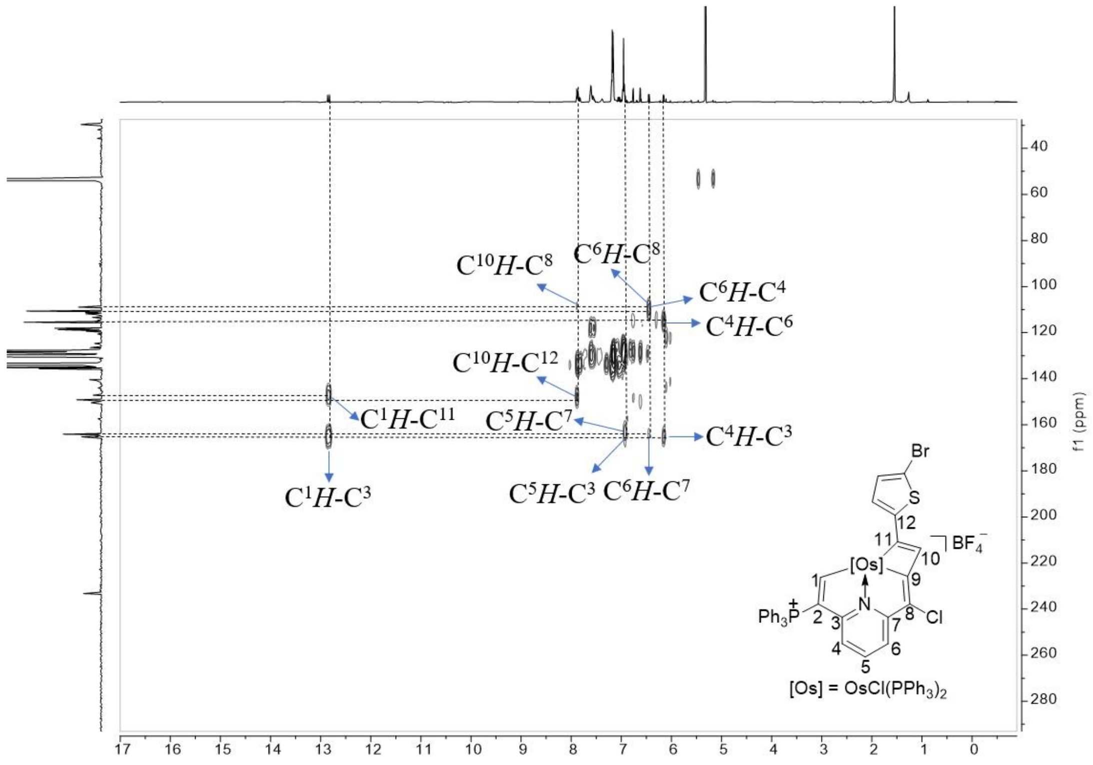

Figure S65. The ^{1}H-^{13}C HMBC (150.9 MHz, CD_2Cl_2) spectrum for complex **4d**.

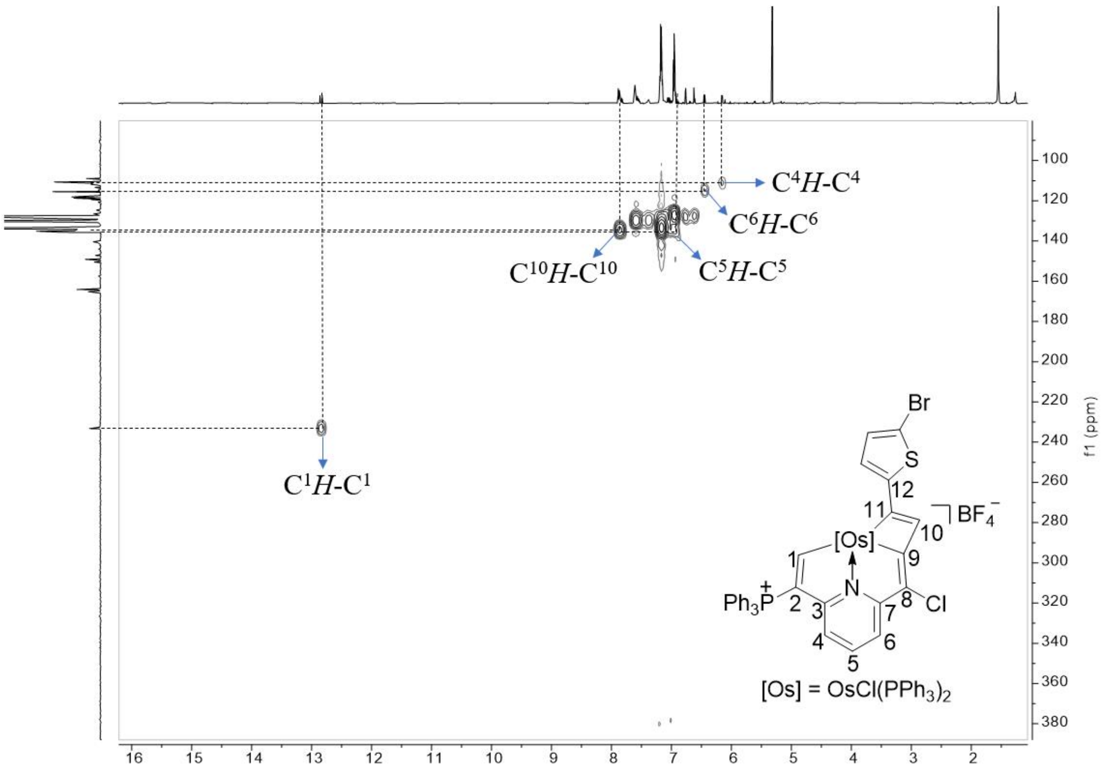

Figure S66. The ^{1}H-^{13}C HSQC (150.9 MHz, CD_2Cl_2) spectrum for complex **4d**.

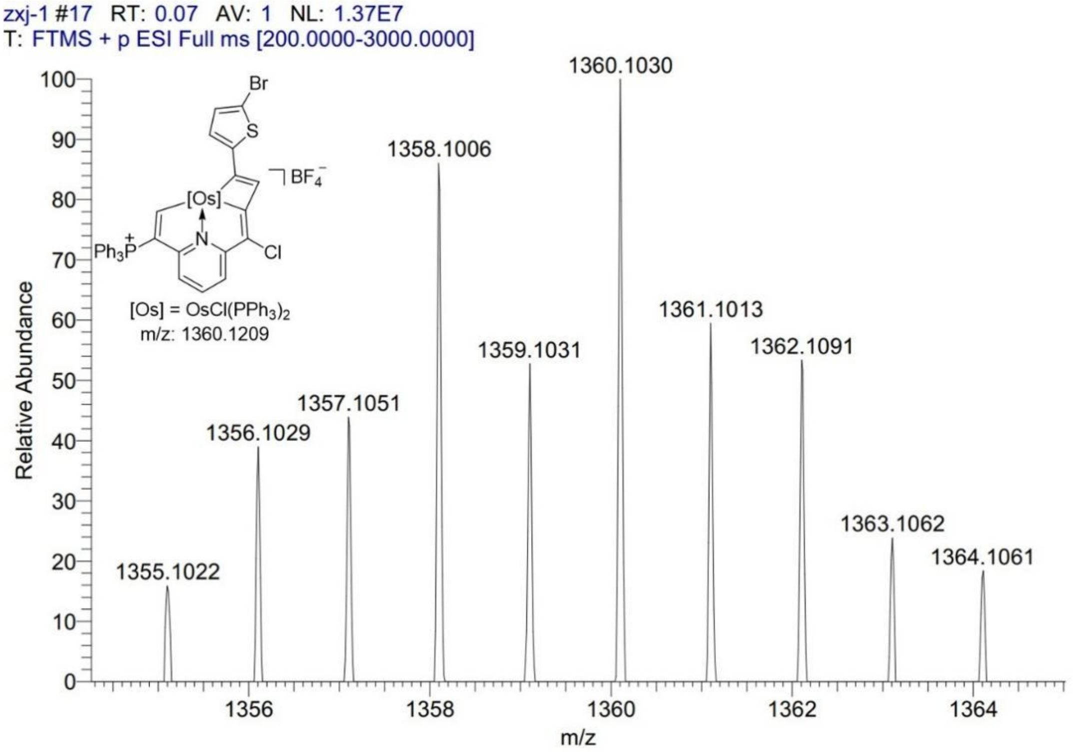

Figure 67. Positive-ion ESI-MS spectrum of [**4d**]$^+$ measured in methanol.

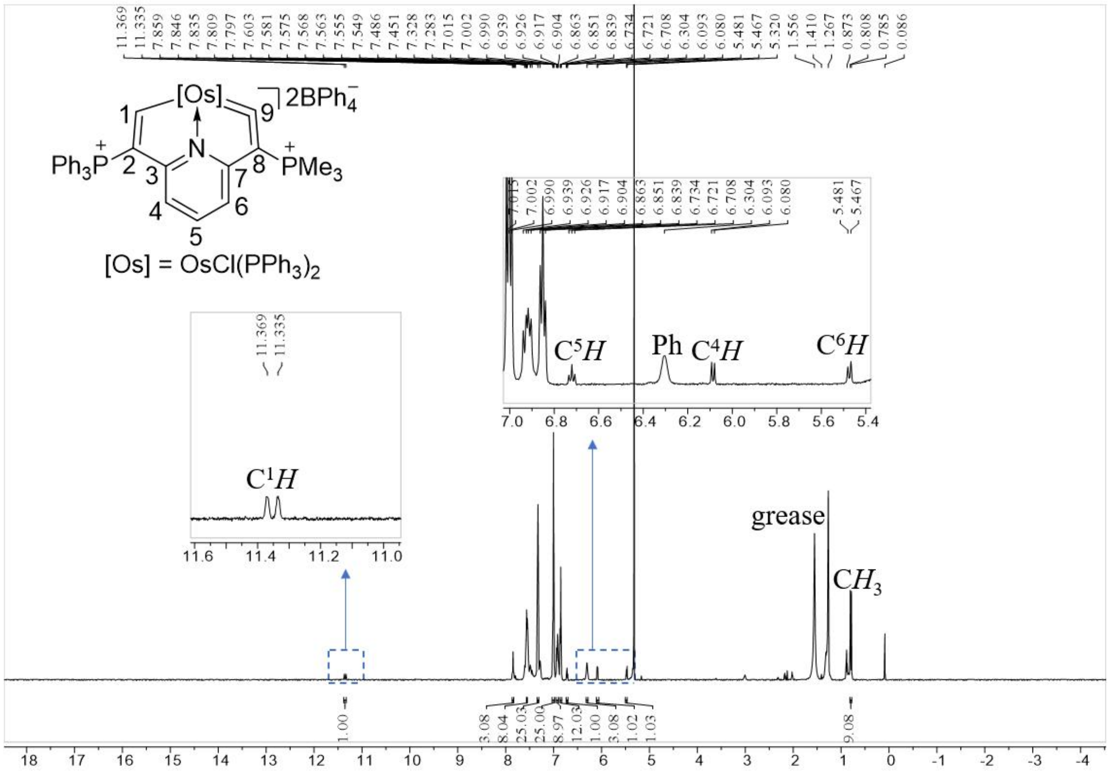

Figure S68. The ^1H NMR (600.1 MHz, CD_2Cl_2) spectrum for complex **5a**.

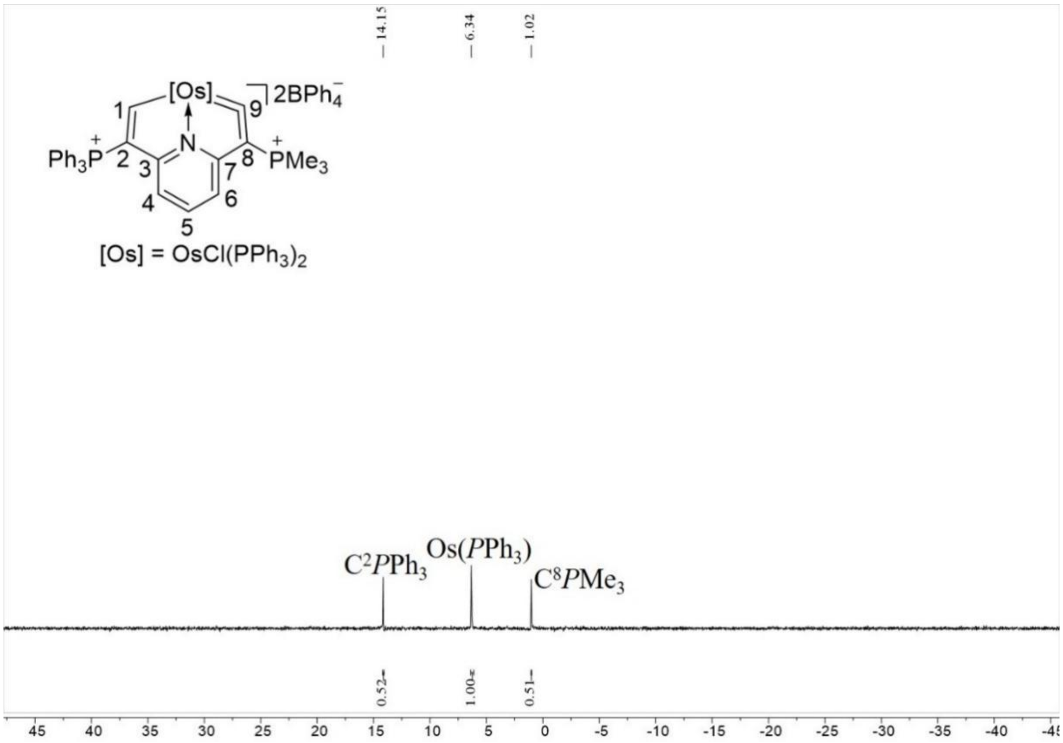

Figure S69. The $^{31}P\{^1H\}$ NMR (242.9 MHz, CD_2Cl_2) spectrum for complex **5a**.

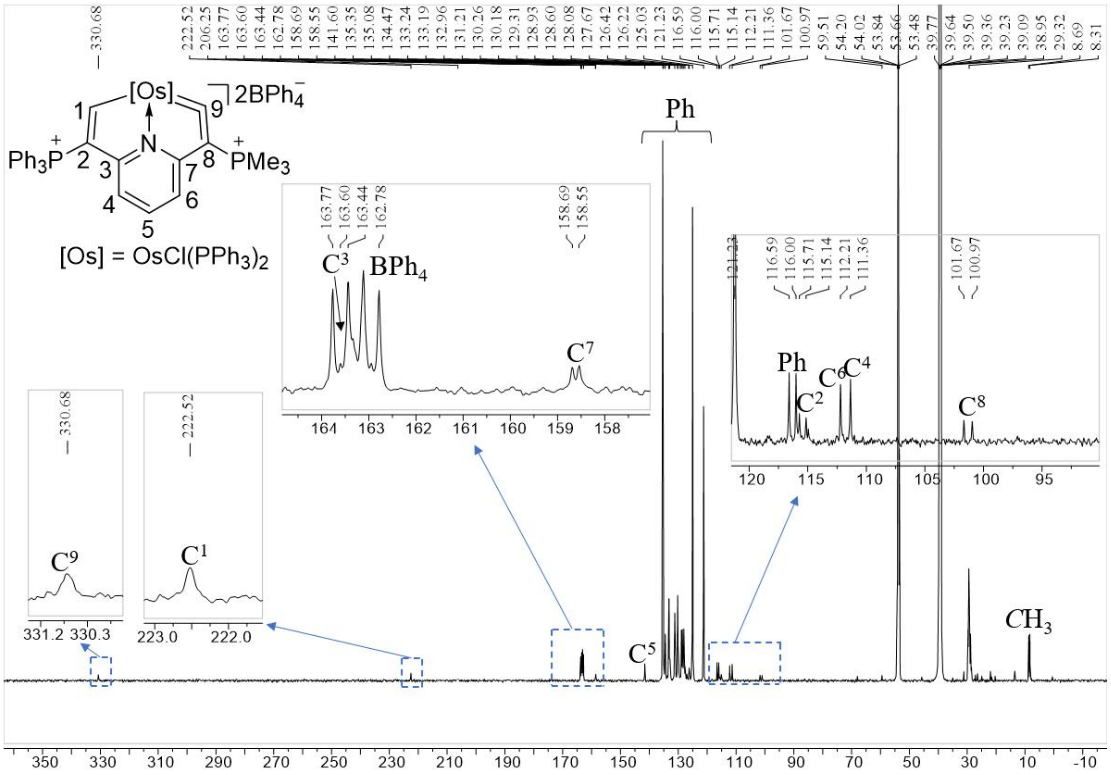

Figure S70. The $^{13}C\{^1H\}$ NMR (150.9 MHz, $CD_2Cl_2/(CD_3)_2SO$ = 1/1) spectrum for complex **5a**.

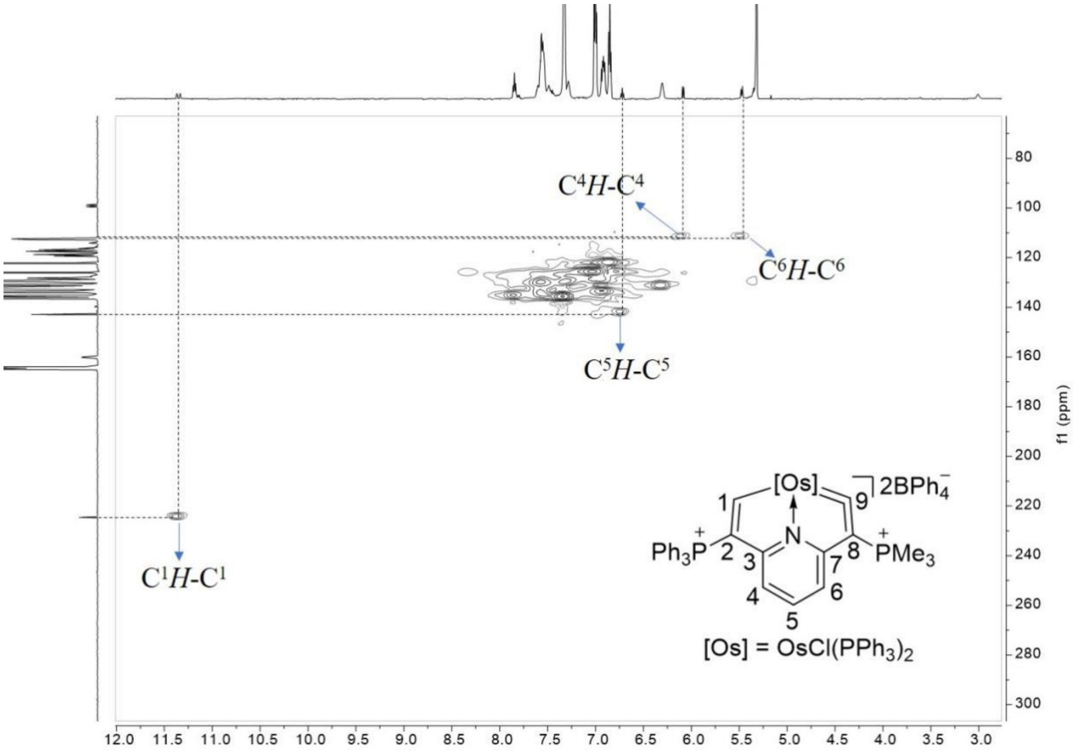

Figure S71. The ^{1}H-^{13}C HSQC (150.9 MHz, CD_2Cl_2) spectrum for complex **5a**.

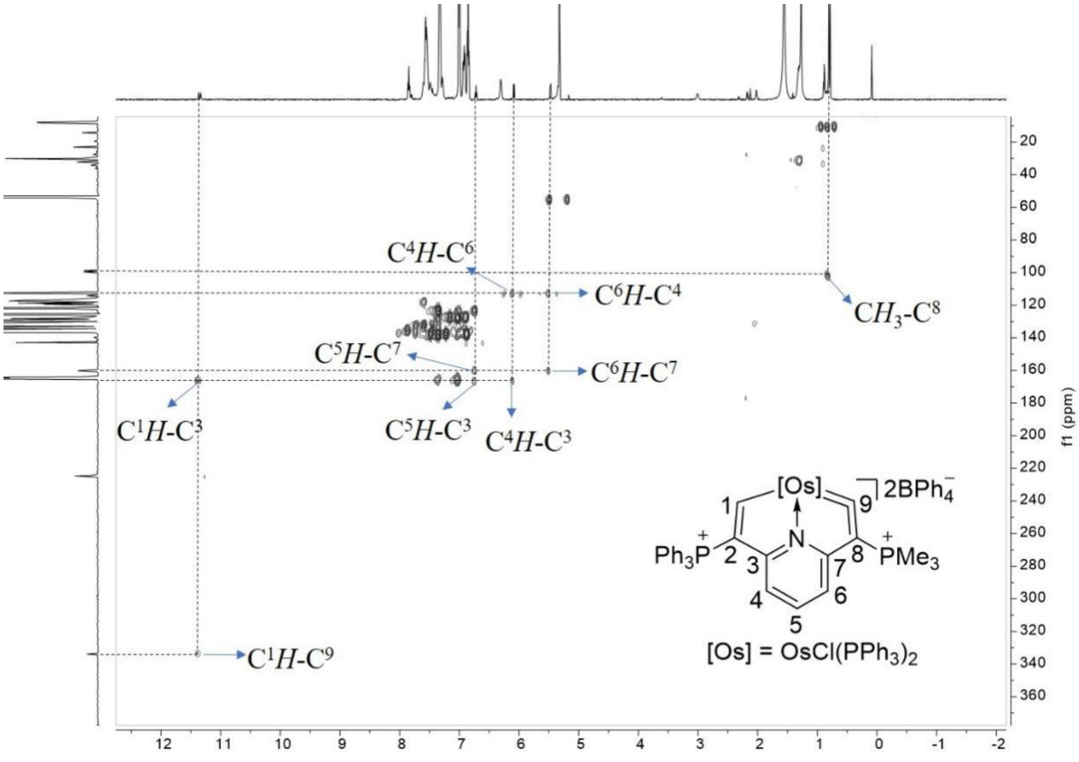

Figure S72. The ^{1}H-^{13}C HMBC (150.9 MHz, CD_2Cl_2) spectrum for complex **5a**.

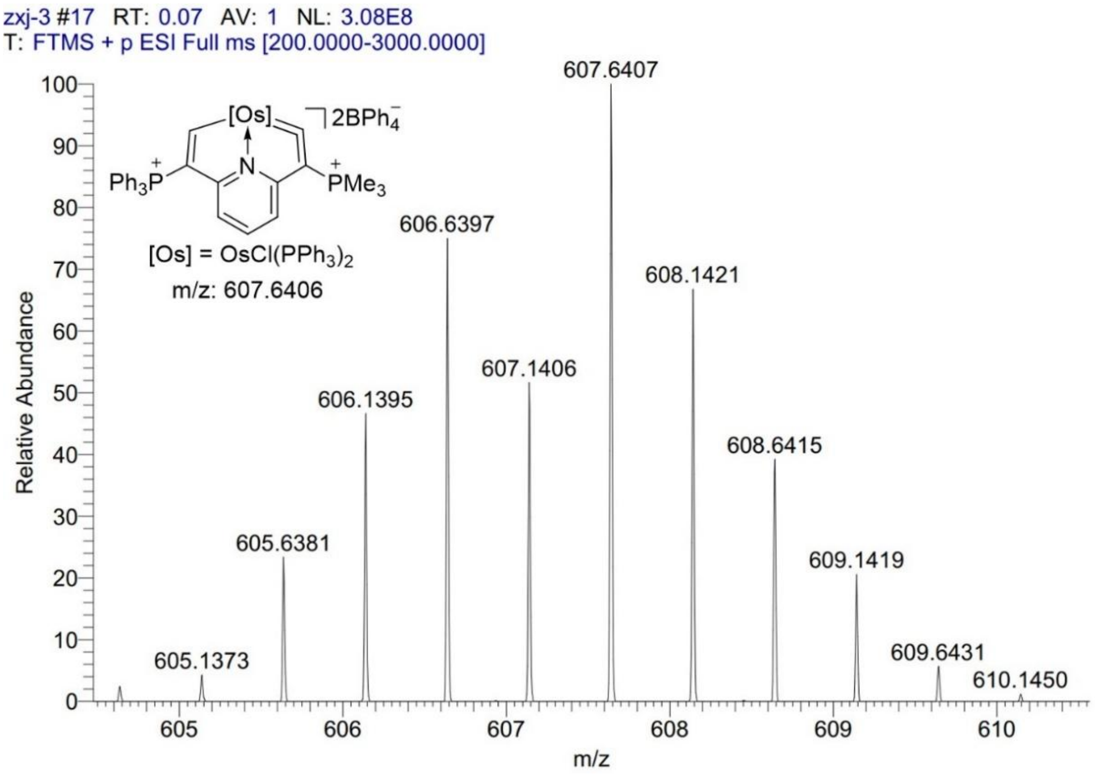

Figure S73. Positive-ion ESI-MS spectrum of [**5a**]$^{+}$ measured in methanol.

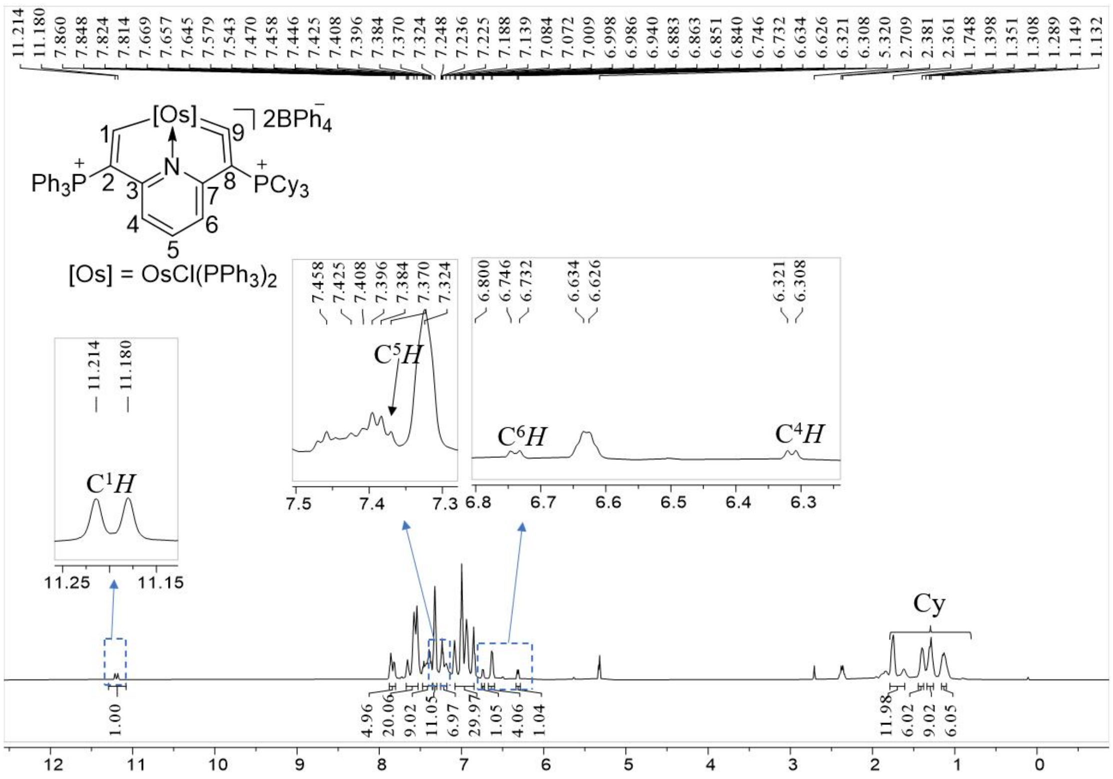

Figure S74. The ^{1}H NMR (600.1 MHz, CD_2Cl_2) spectrum for complex **5b**.

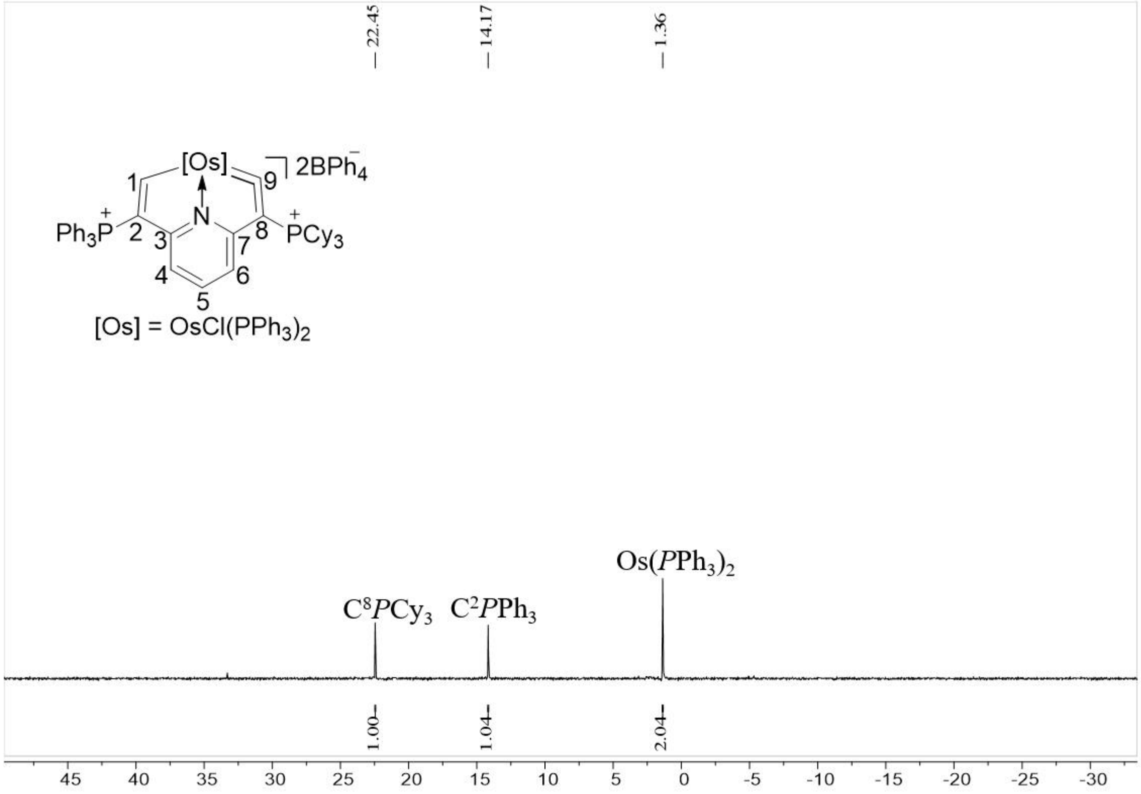

Figure S75. The $^{31}P\{^{1}H\}$ NMR (242.9 MHz, CD_2Cl_2) spectrum for complex **5b**.

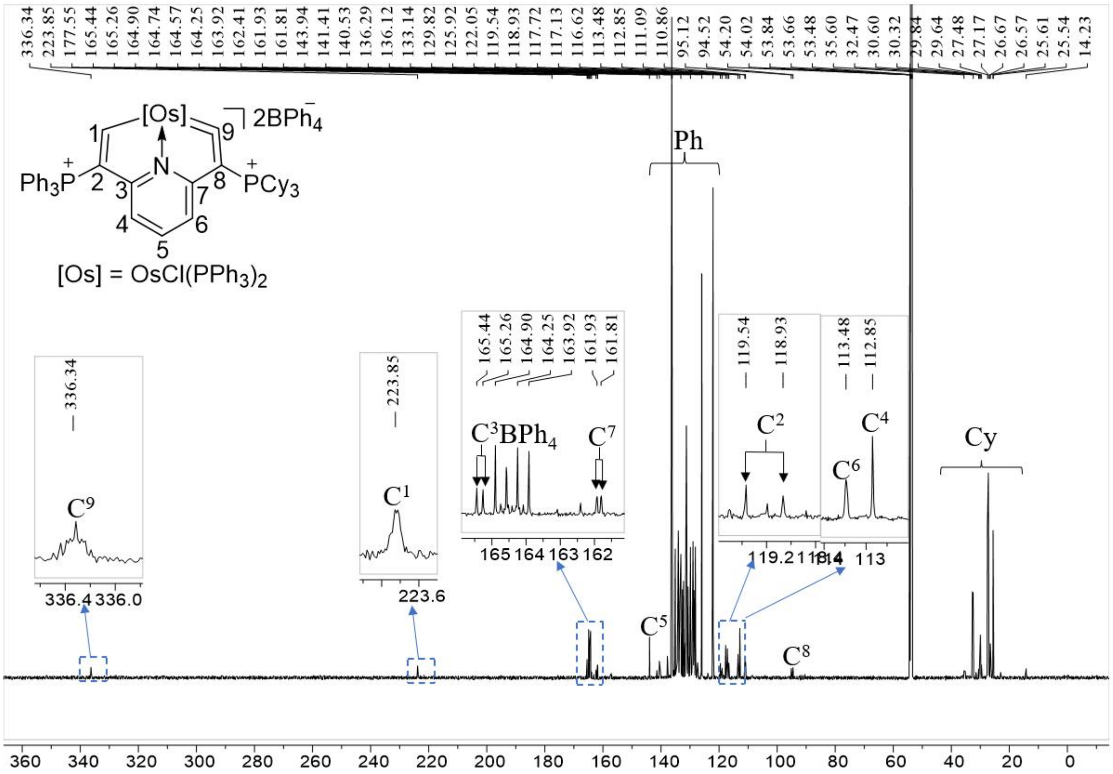

Figure S76. The $^{13}C\{^{1}H\}$ NMR (150.9 MHz, CD_2Cl_2) spectrum for complex **5b**.

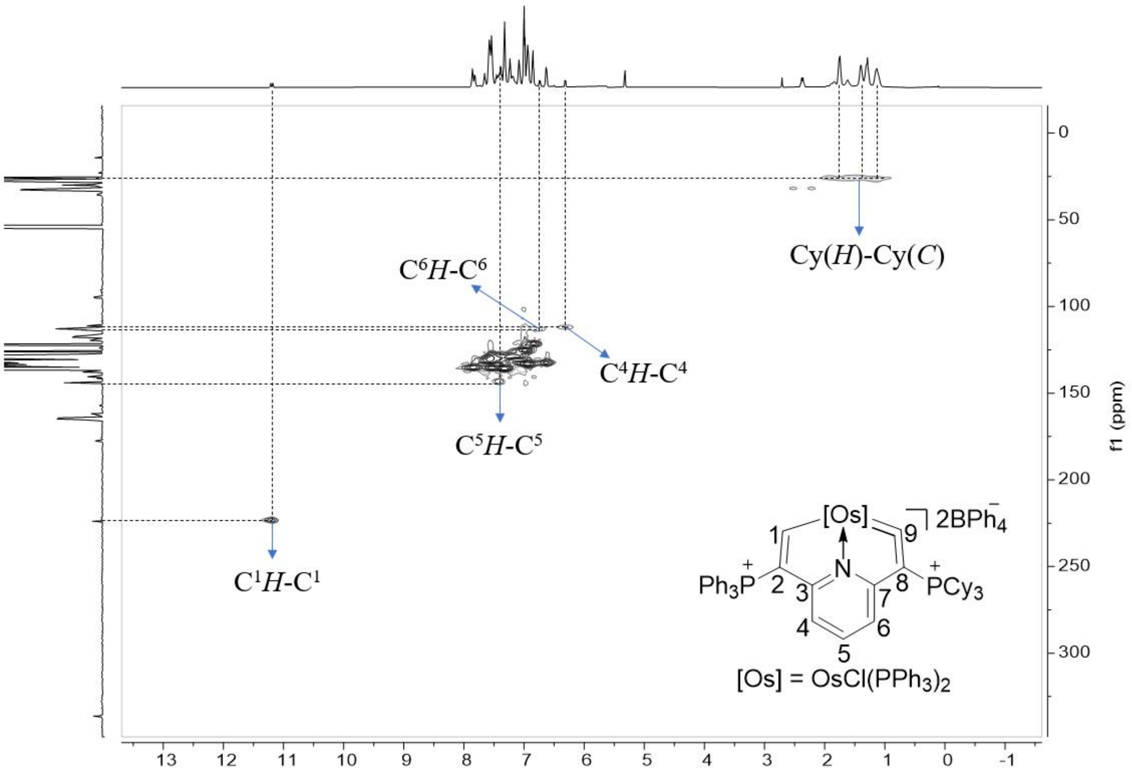

Figure S77. The ^{1}H-^{13}C HSQC (150.9 MHz, CD_2Cl_2) spectrum for complex **5b**.

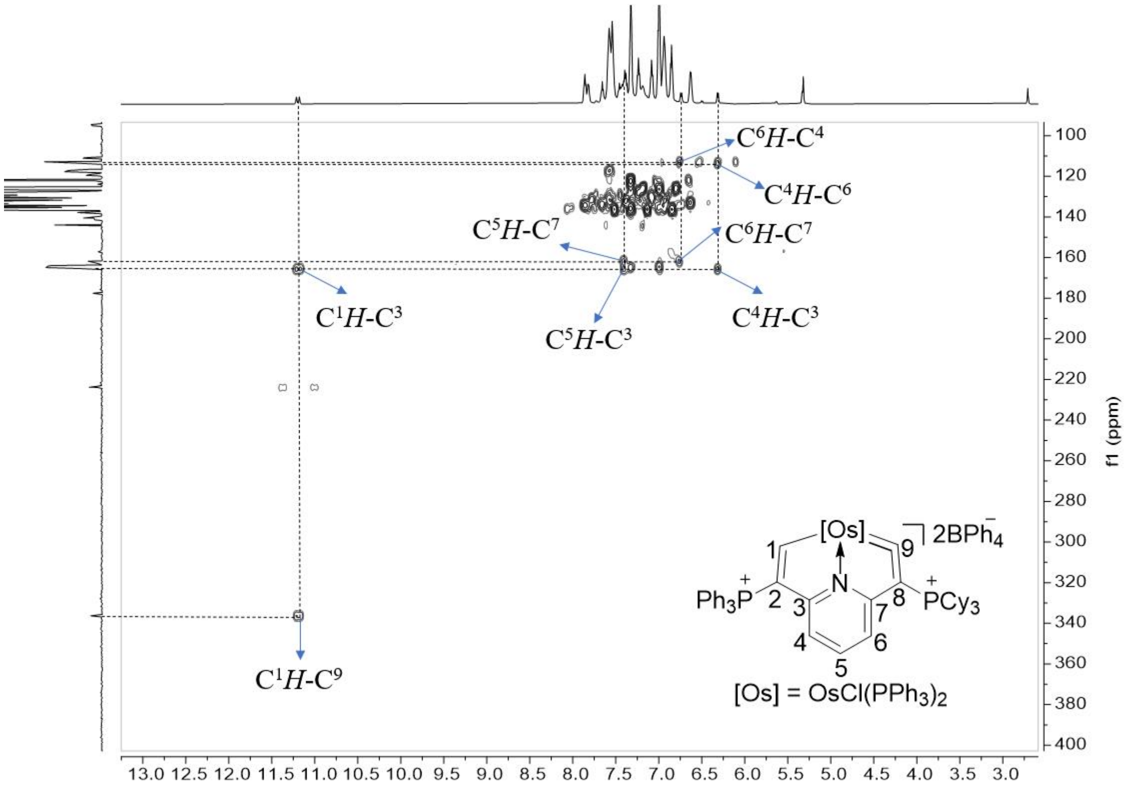

Figure S78. The ^{1}H-^{13}C HMBC (150.9 MHz, CD_2Cl_2) spectrum for complex **5b**.

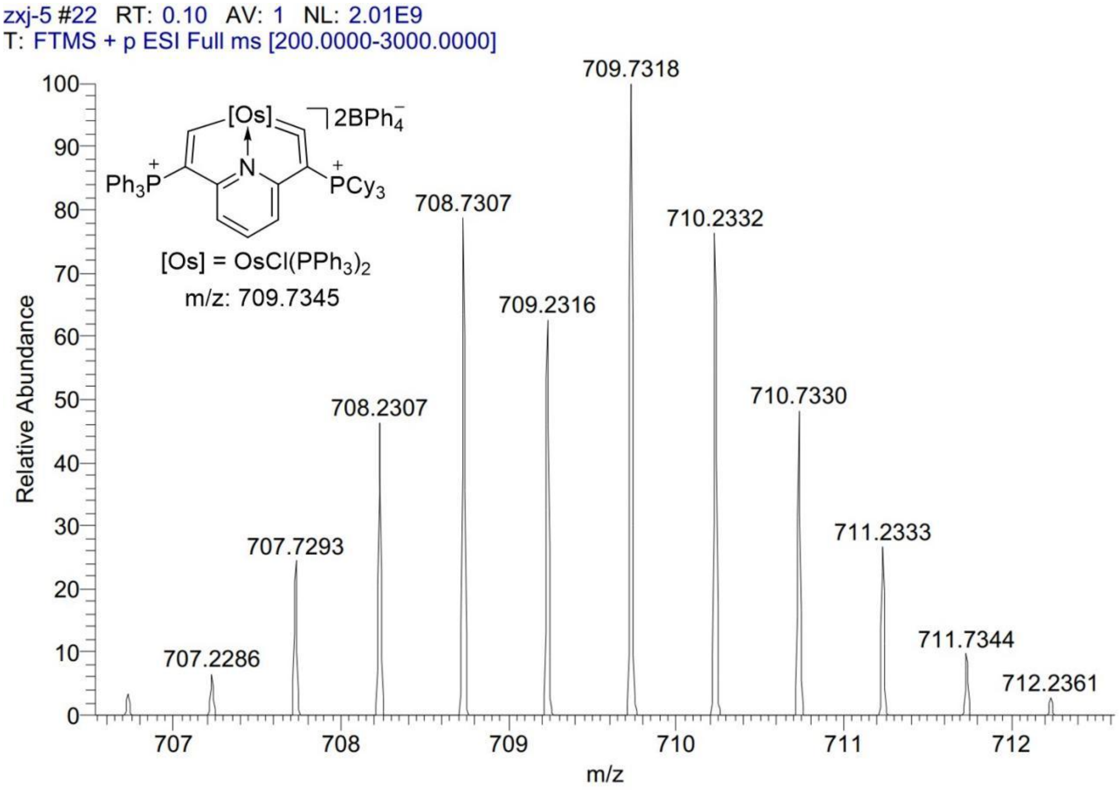

Figure 79. Positive-ion ESI-MS spectrum of [**5b**]$^+$ measured in methanol.

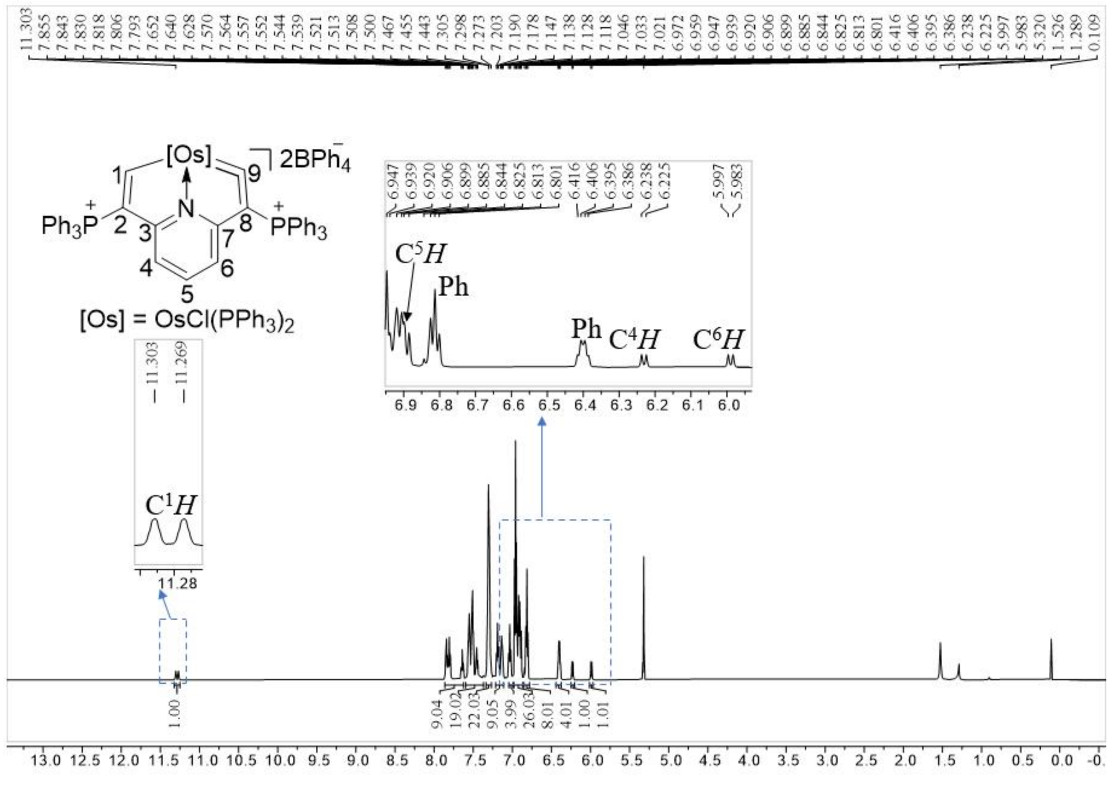

Figure S80. The ^1H NMR (600.1 MHz, CD_2Cl_2) spectrum for complex **5c**.

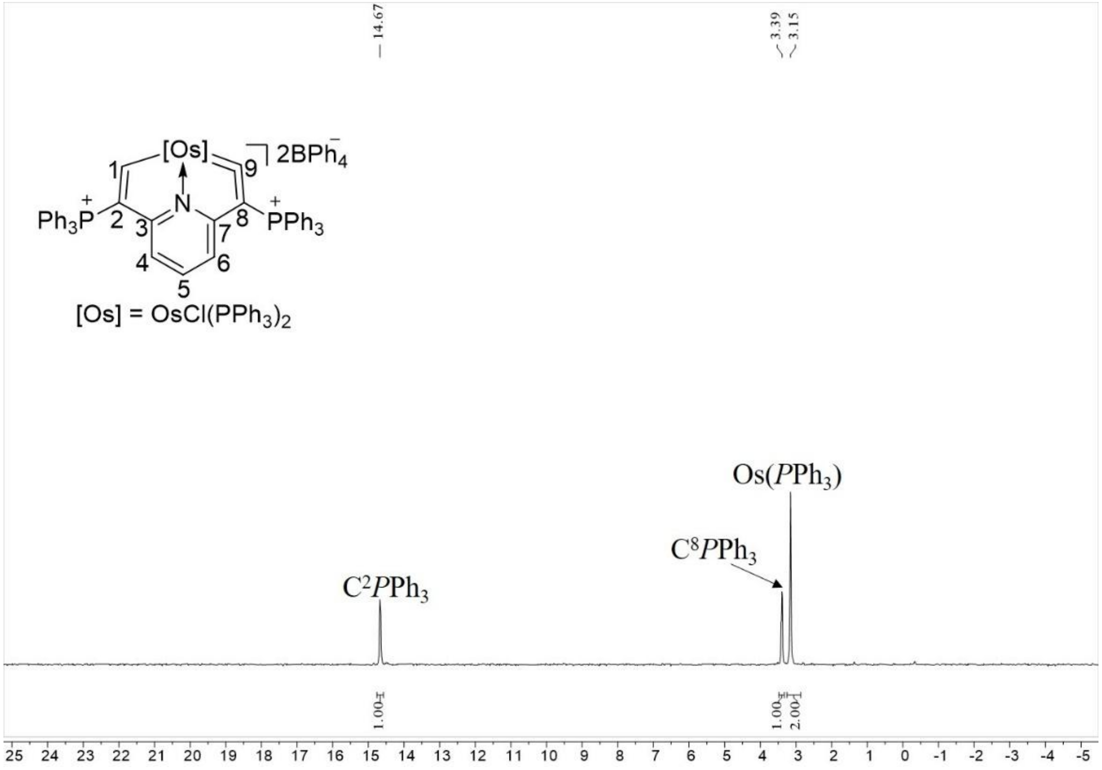

Figure S81. The $^{31}P\{^{1}H\}$ NMR (242.9 MHz, CD_2Cl_2) spectrum for complex **5c**.

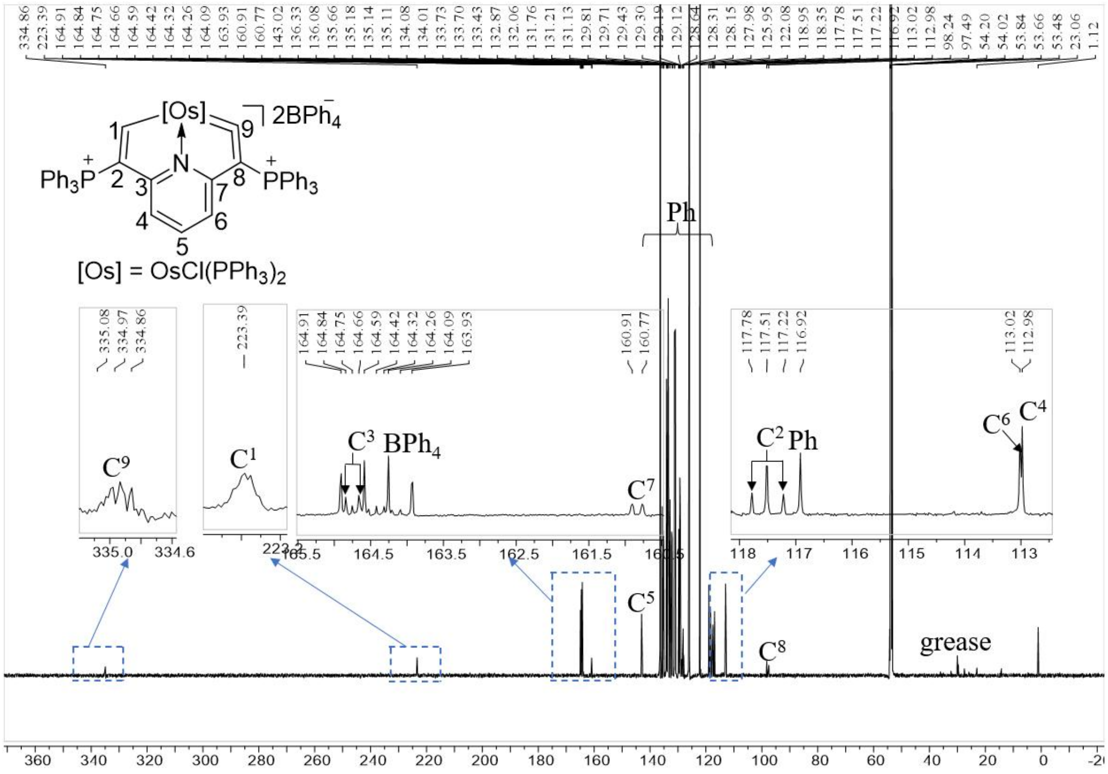

Figure S82. The $^{13}C\{^{1}H\}$ NMR (150.9 MHz, CD_2Cl_2) spectrum for complex **5c**.

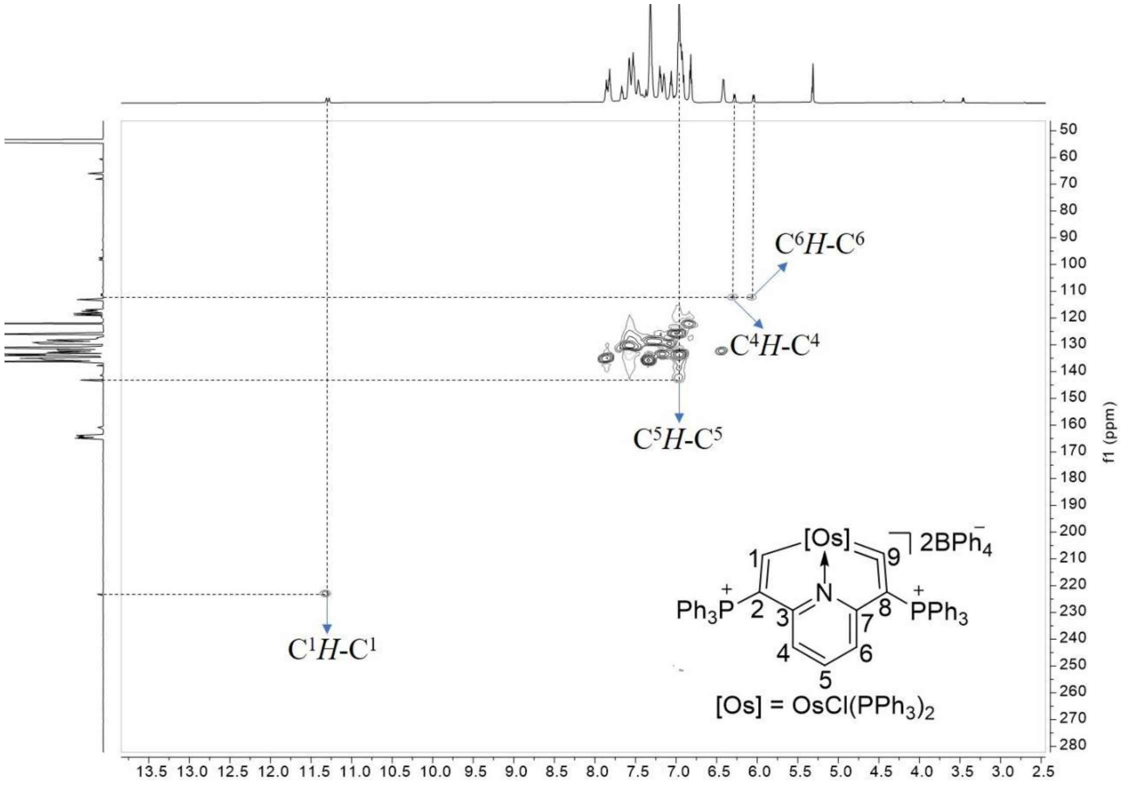

Figure S83. The ^{1}H-^{13}C HSQC (150.9 MHz, CD_2Cl_2) spectrum for complex **5c**.

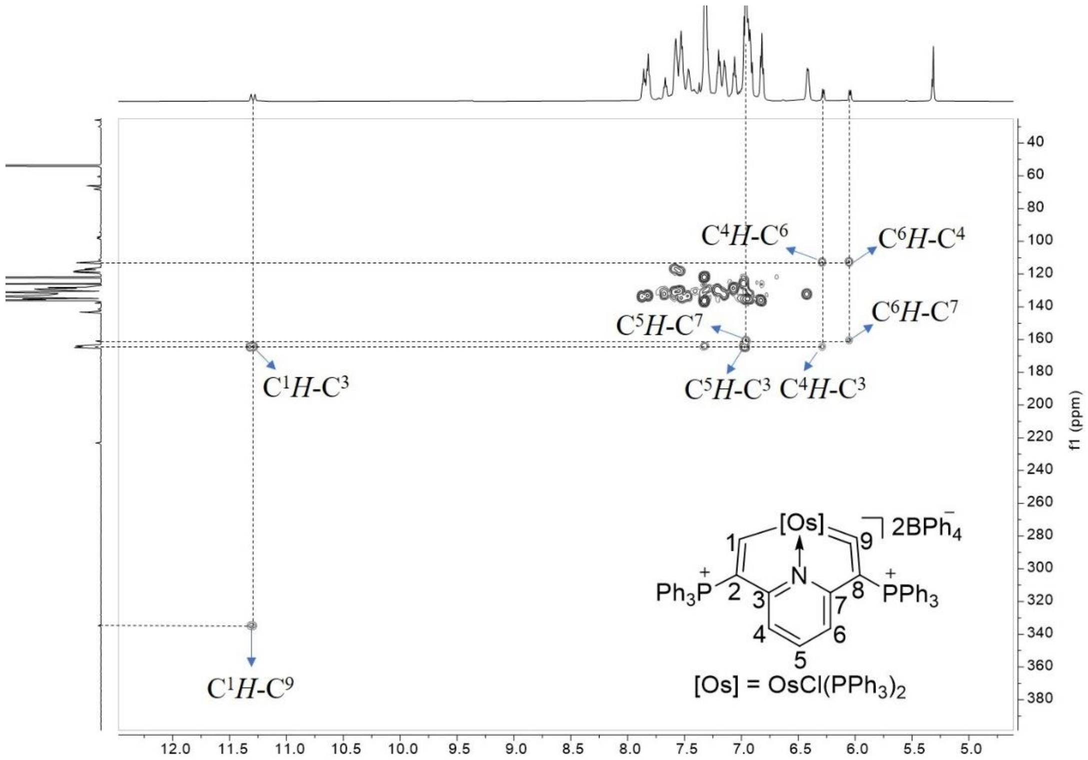

Figure S84. The ^{1}H-^{13}C HMBC (150.9 MHz, CD_2Cl_2) spectrum for complex **5c**.

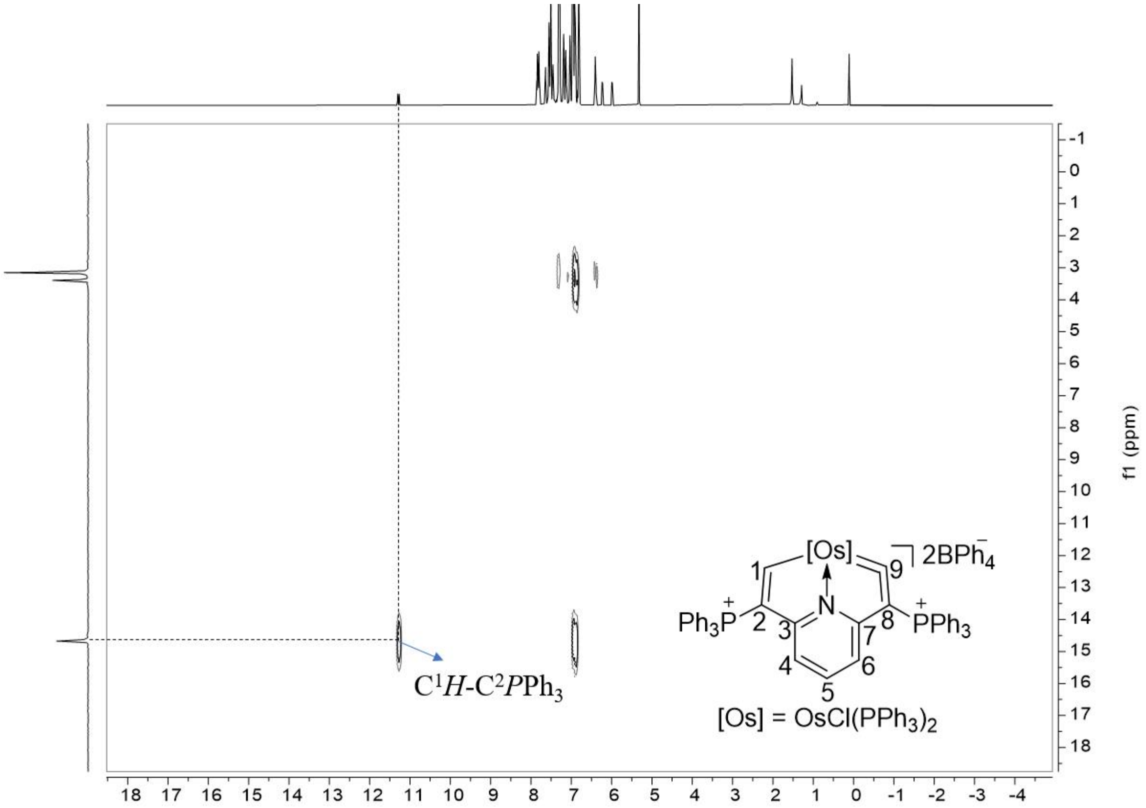

Figure S85. The ^{1}H-^{31}P HMBC (242.9 MHz, CD_2Cl_2) spectrum for complex **5c**.

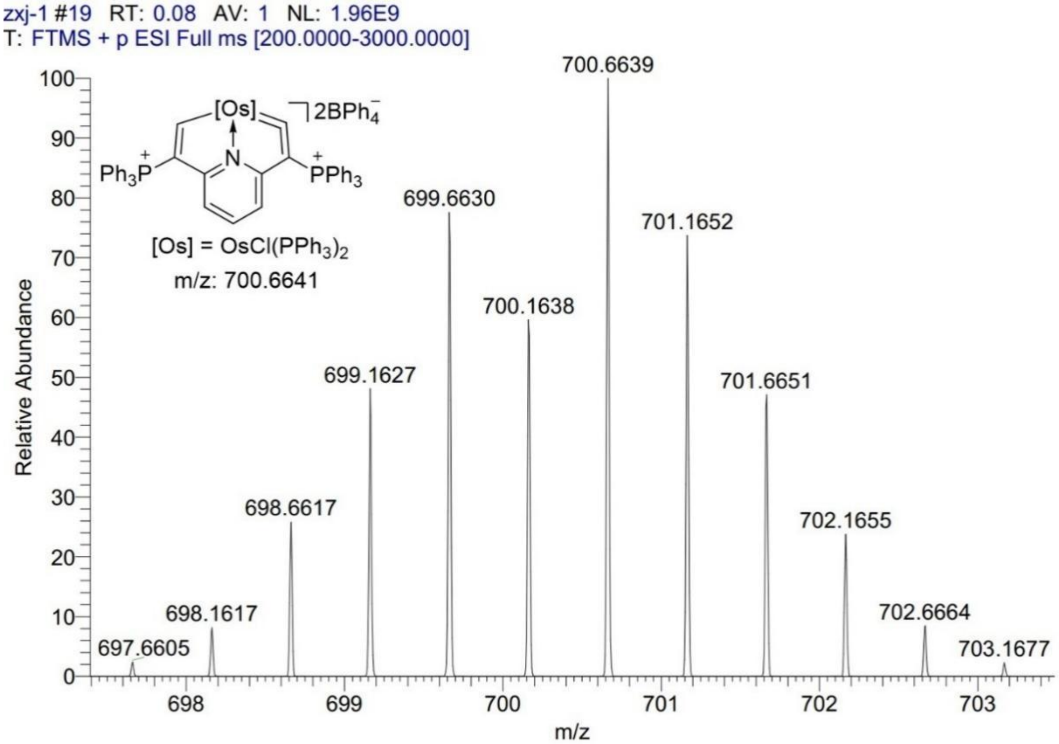

Figure S86. Positive-ion ESI-MS spectrum of [**5c**]$^{+}$ measured in methanol.

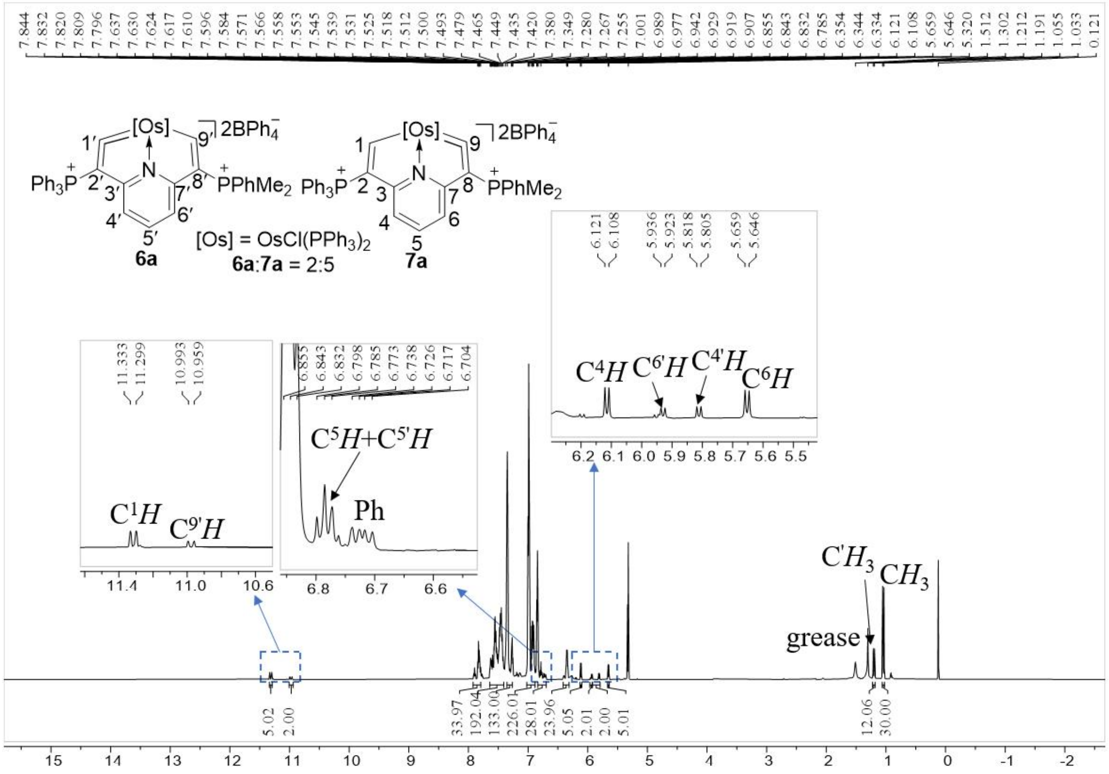

Figure S87. The ^{1}H NMR (600.1 MHz, CD_2Cl_2) spectrum for complexes **6a** and **7a**.

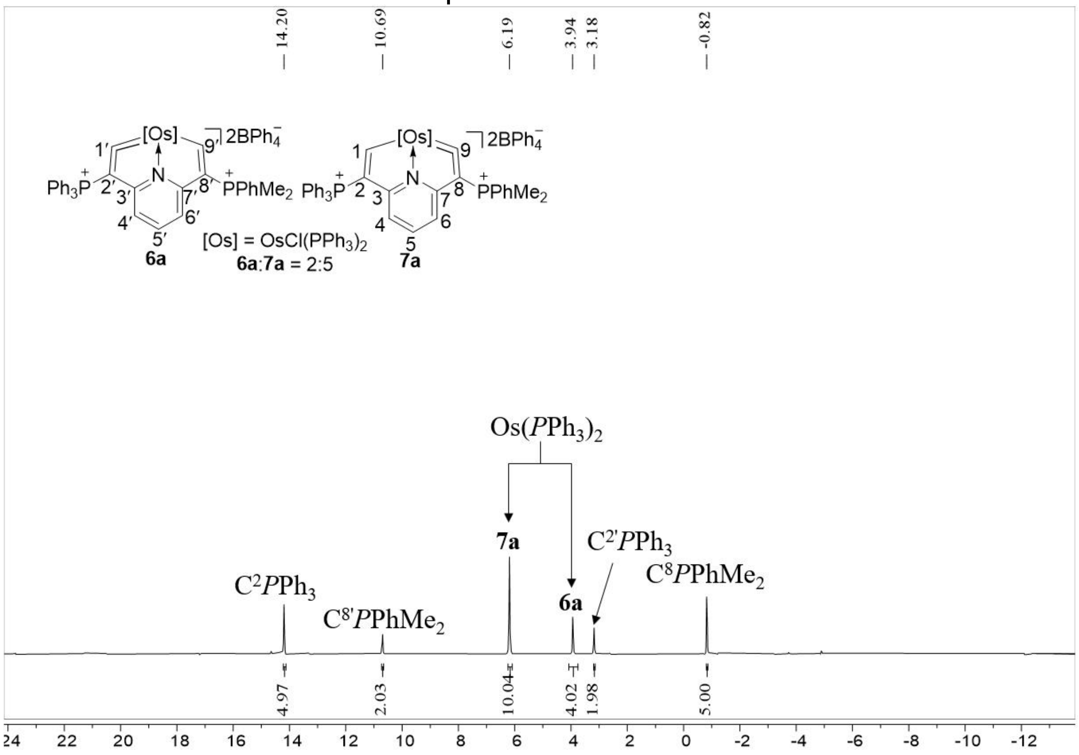

Figure S88. The $^{31}P\{^{1}H\}$ NMR (242.9 MHz, CD_2Cl_2) spectrum for complexes **6a** and **7a**.

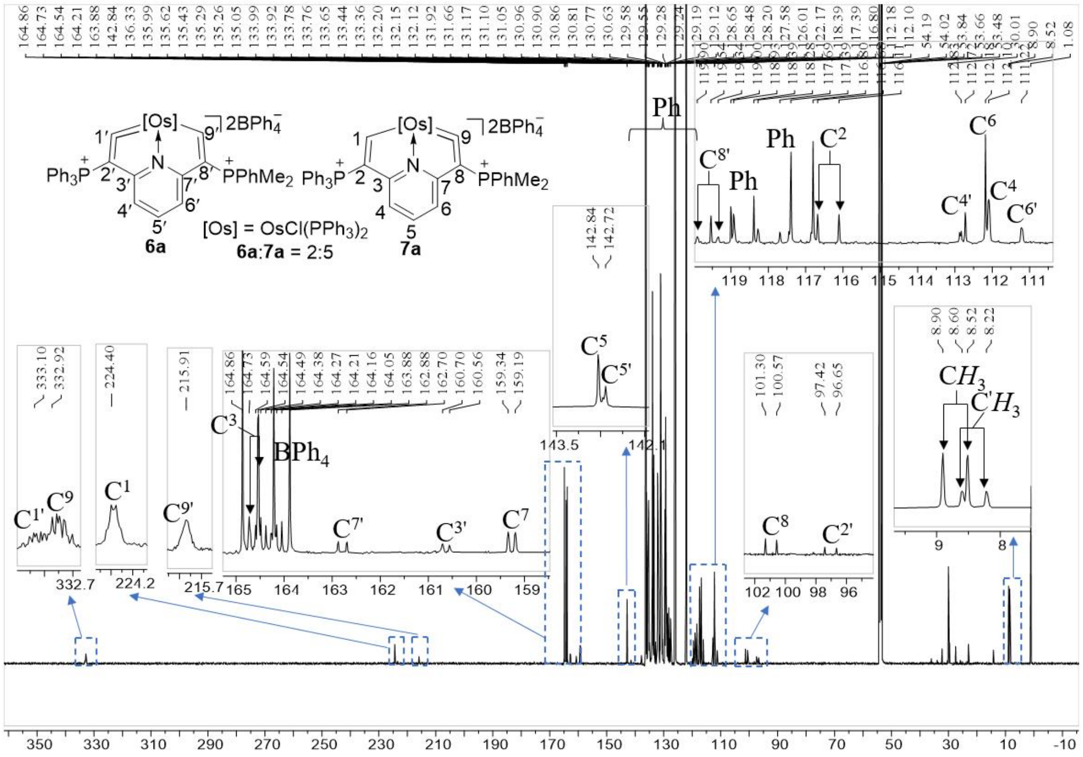

Figure S89. The $^{13}C\{^{1}H\}$ NMR (150.9 MHz, CD_2Cl_2) spectrum for complexes **6a** and **7a**.

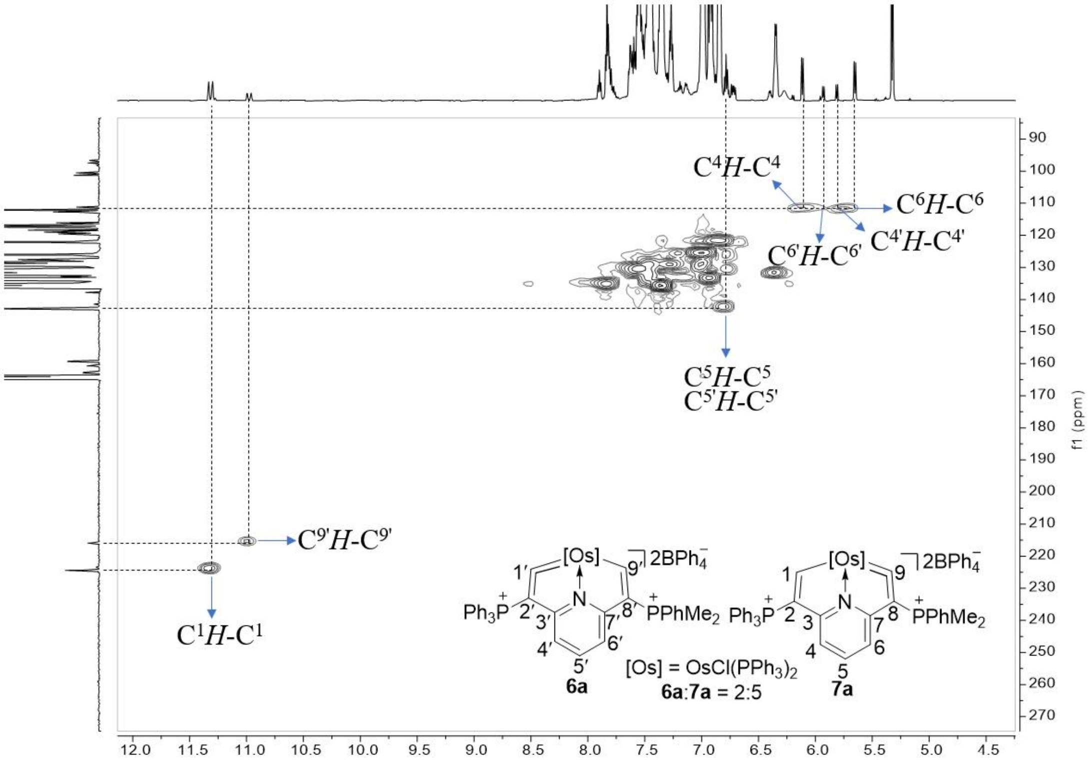

Figure S90. The ^{1}H-^{13}C HSQC (150.9 MHz, CD_2Cl_2) spectrum for complexes **6a** and **7a**.

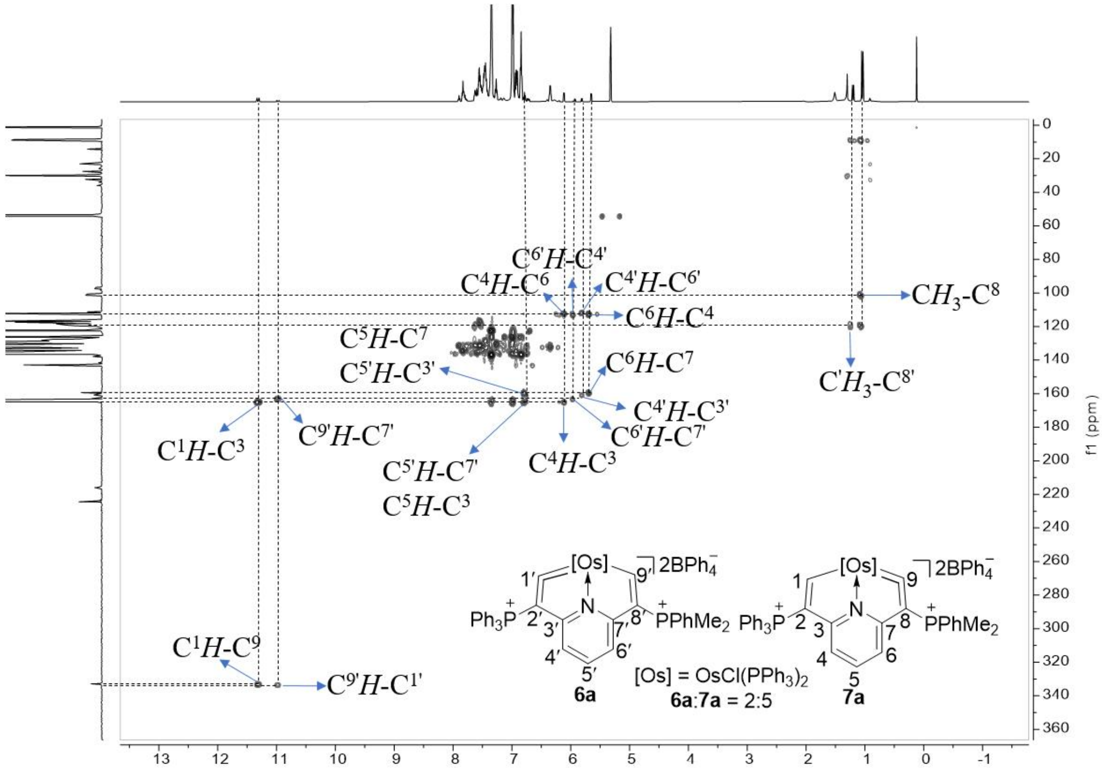

Figure S91. The ^{1}H-^{13}C HMBC (150.9 MHz, CD_2Cl_2) spectrum for complexes **6a** and **7a**.

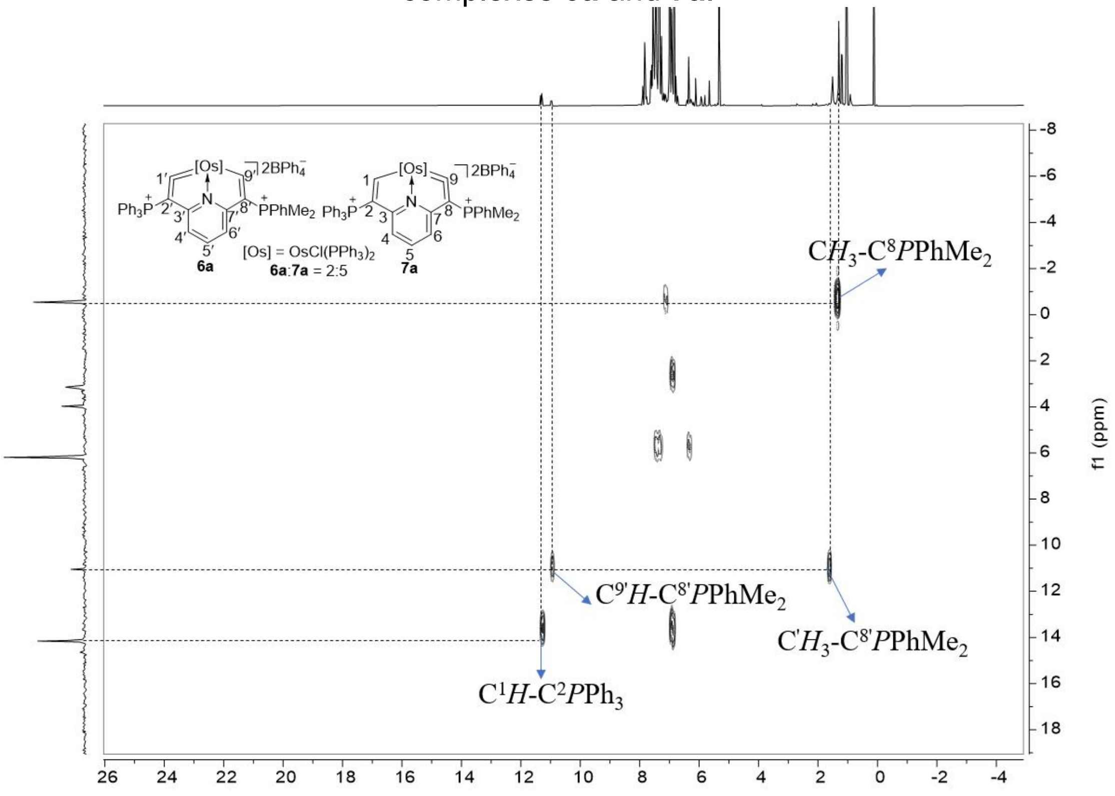

Figure S92. The ^{1}H-^{31}P HMBC (242.9 MHz, CD_2Cl_2) spectrum for complexes **6a** and **7a**.

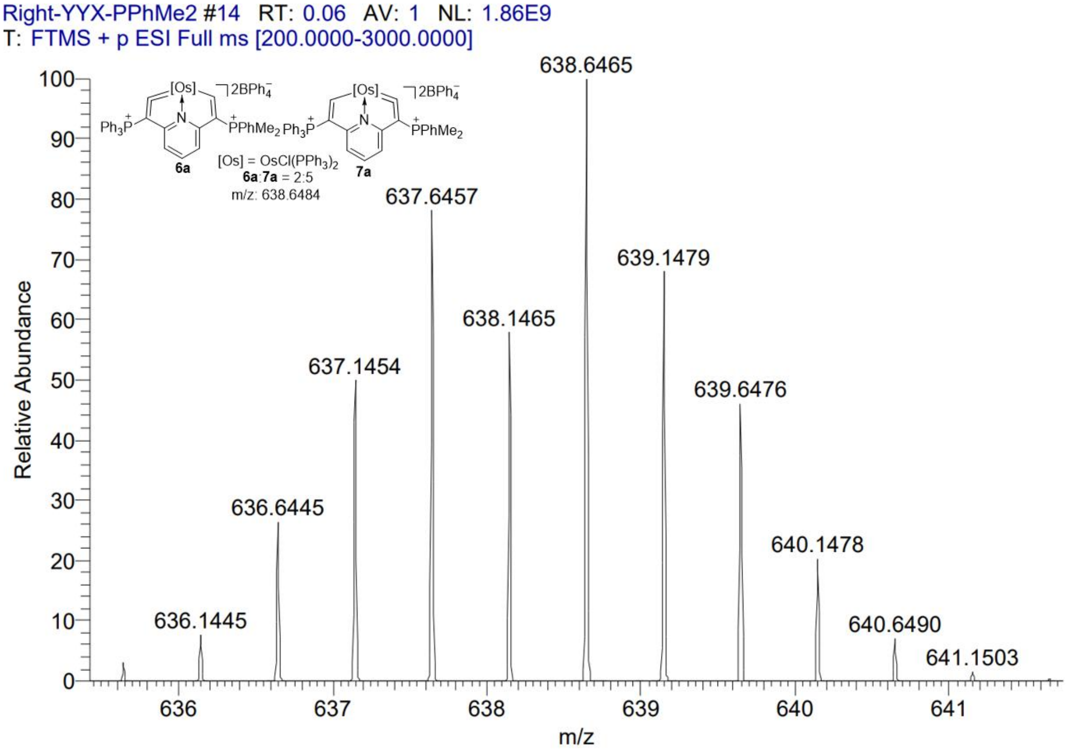

Figure S93. Positive-ion ESI-MS spectrum of [**6a** and **7a**]$^+$ measured in methanol.

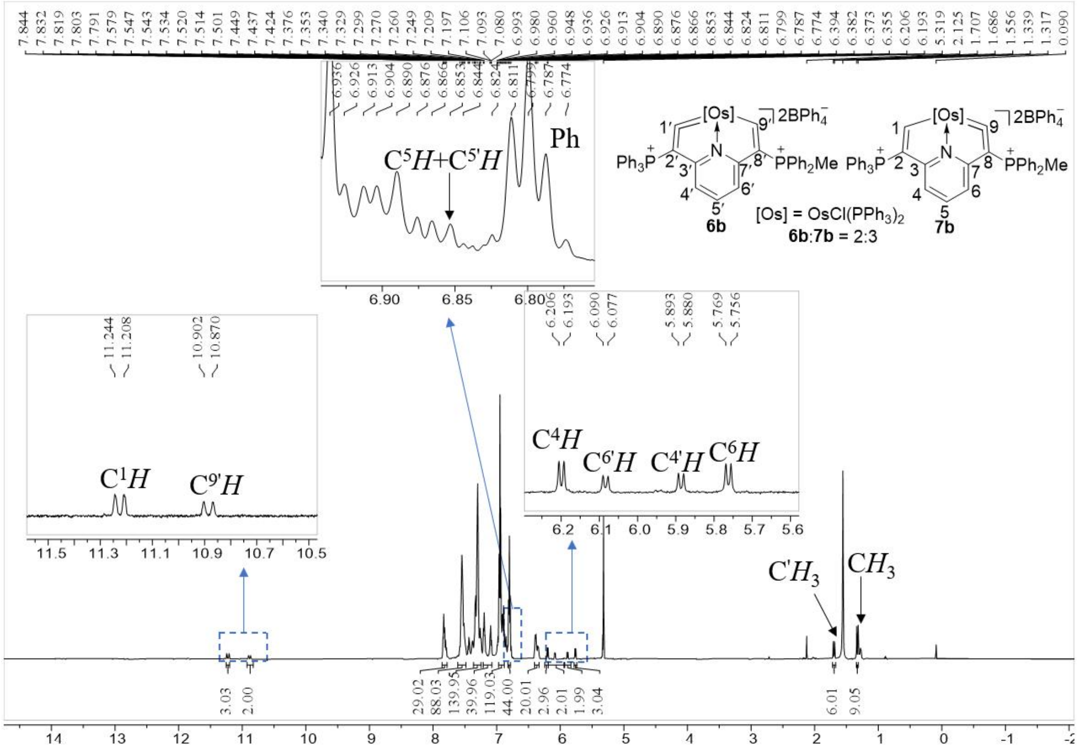

Figure S94. The ^{1}H NMR (600.1 MHz, CD_2Cl_2) spectrum for complexes **6b** and **7b**.

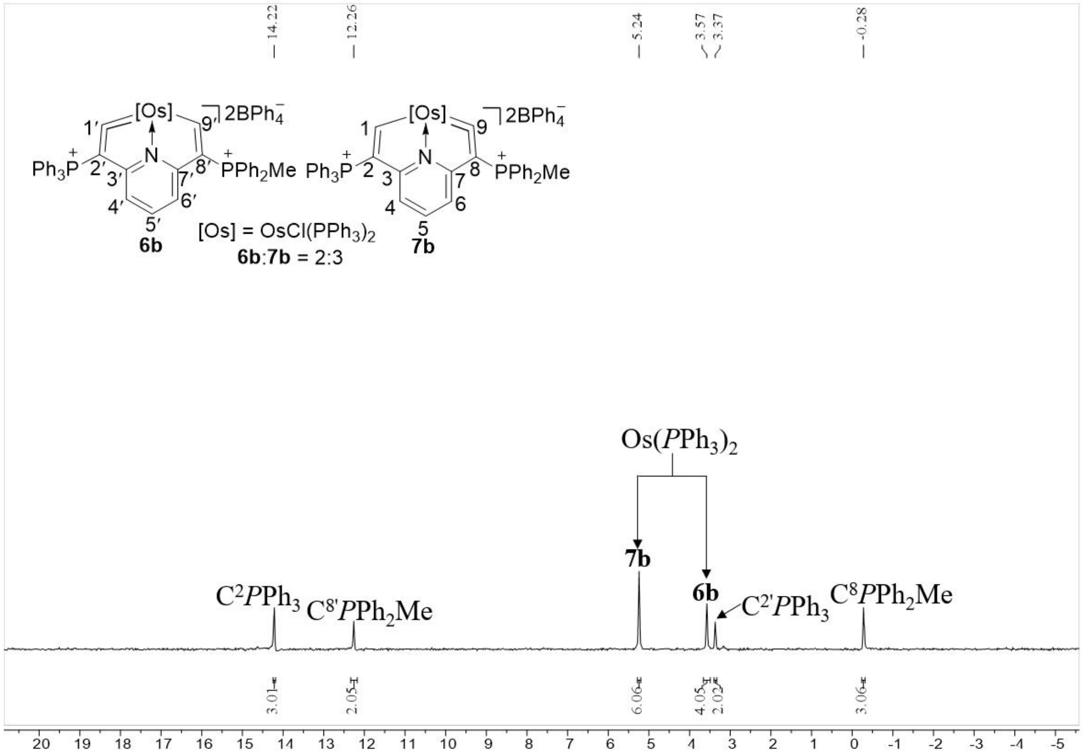

Figure S95. The $^{31}P\{^1H\}$ NMR (242.9 MHz, CD_2Cl_2) spectrum for complexes **6b** and **7b**.

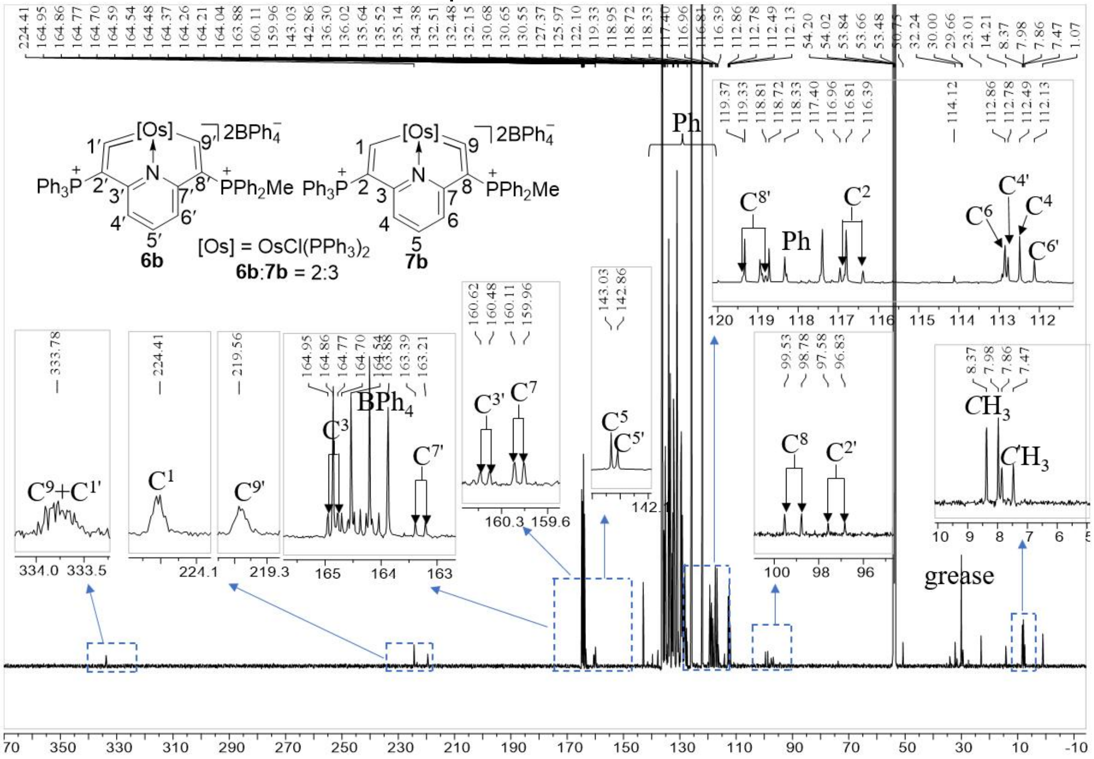

Figure S96. The $^{13}C\{^1H\}$ NMR (150.9 MHz, CD_2Cl_2) spectrum for complexes **6b** and **7b**.

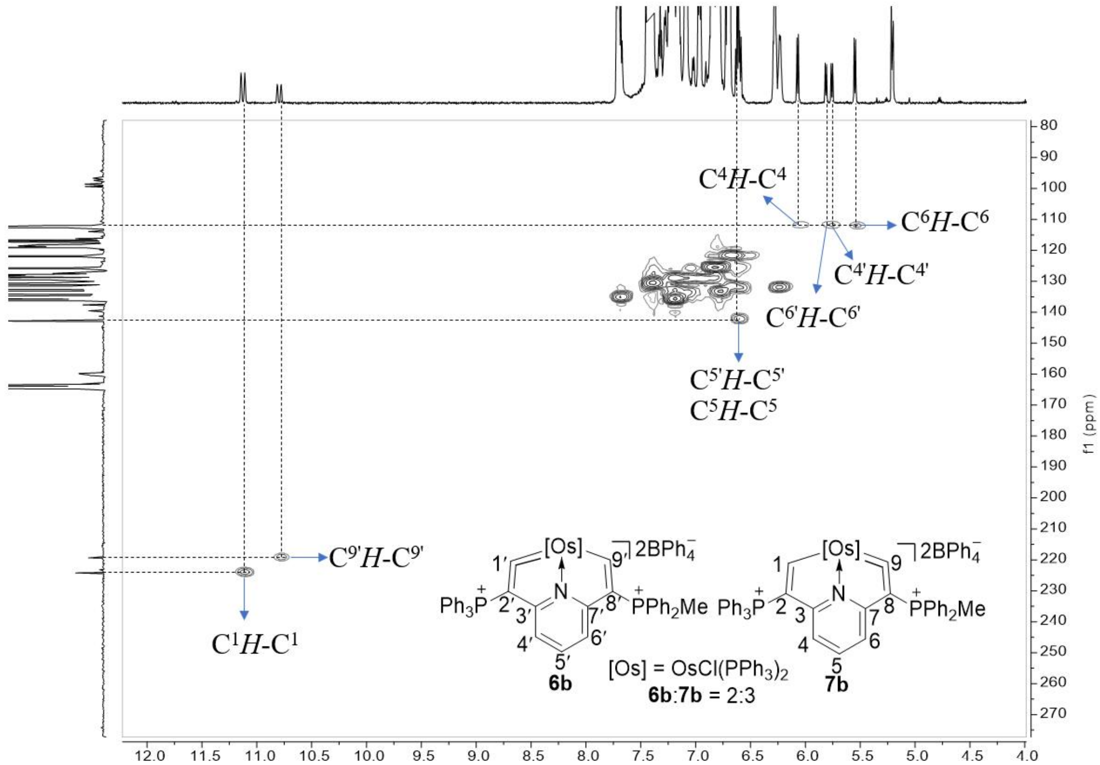

Figure S97. The ^{1}H-^{13}C HSQC (150.9 MHz, CD_2Cl_2) spectrum for complexes **6b** and **7b**.

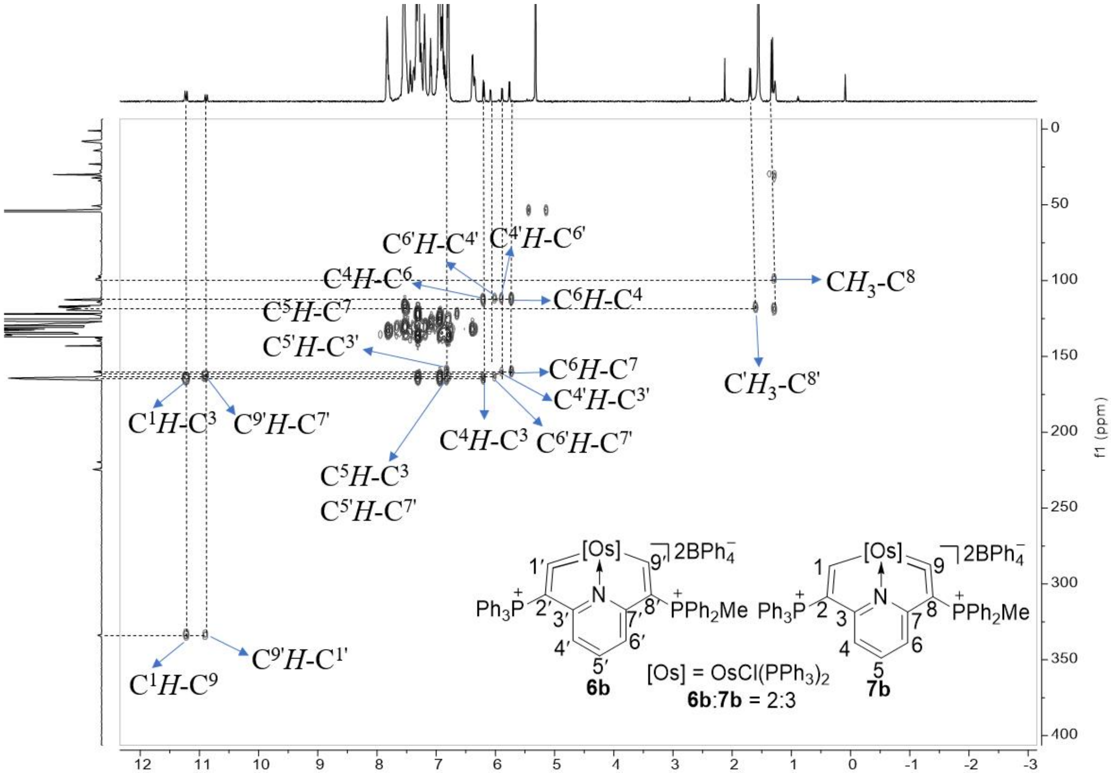

Figure S98. The ^{1}H-^{13}C HMBC (150.9 MHz, CD_2Cl_2) spectrum for complexes **6b** and **7b**.

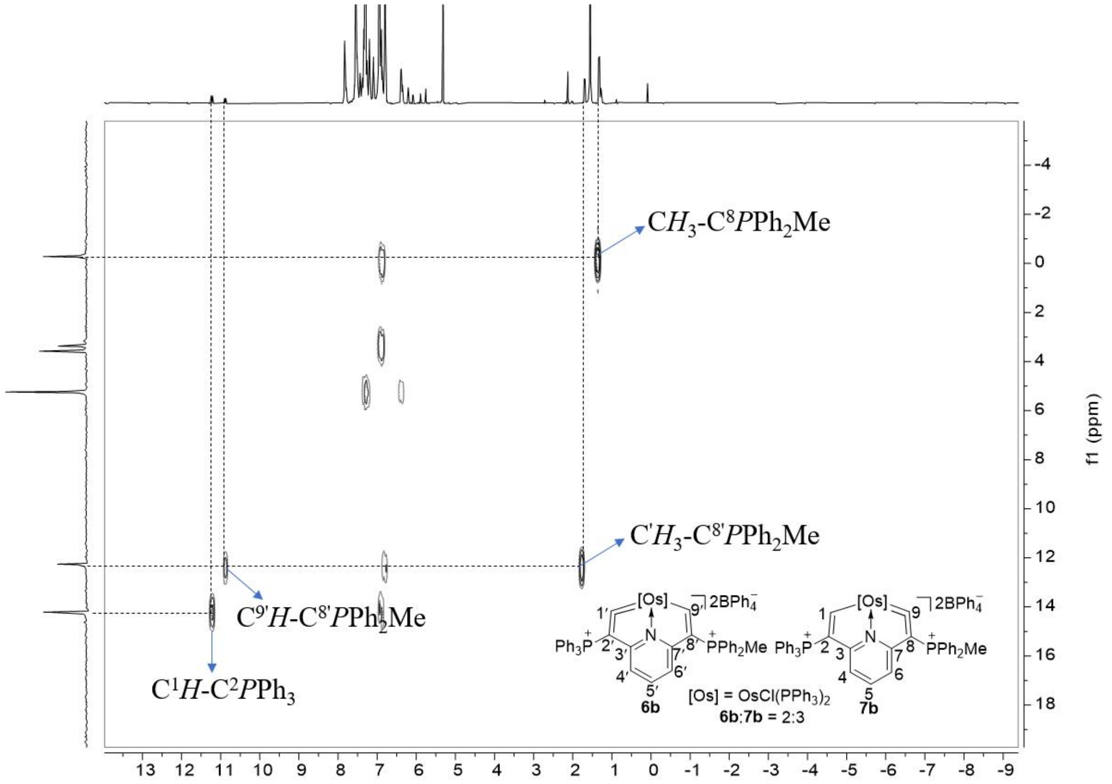

Figure S99. The ^1H-^{31}P HMBC (242.9 MHz, CD_2Cl_2) spectrum for complexes **6b** and **7b**.

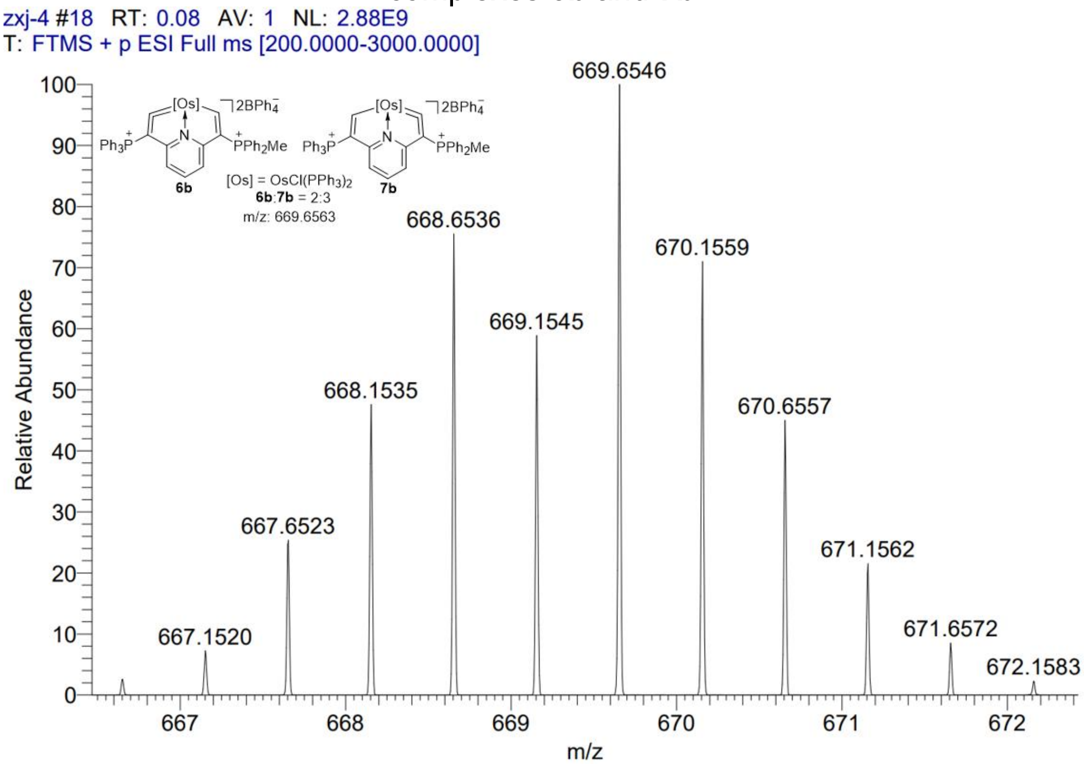

Figure S100. Positive-ion ESI-MS spectrum of [**6b** and **7b**]$^+$ measured in methanol.

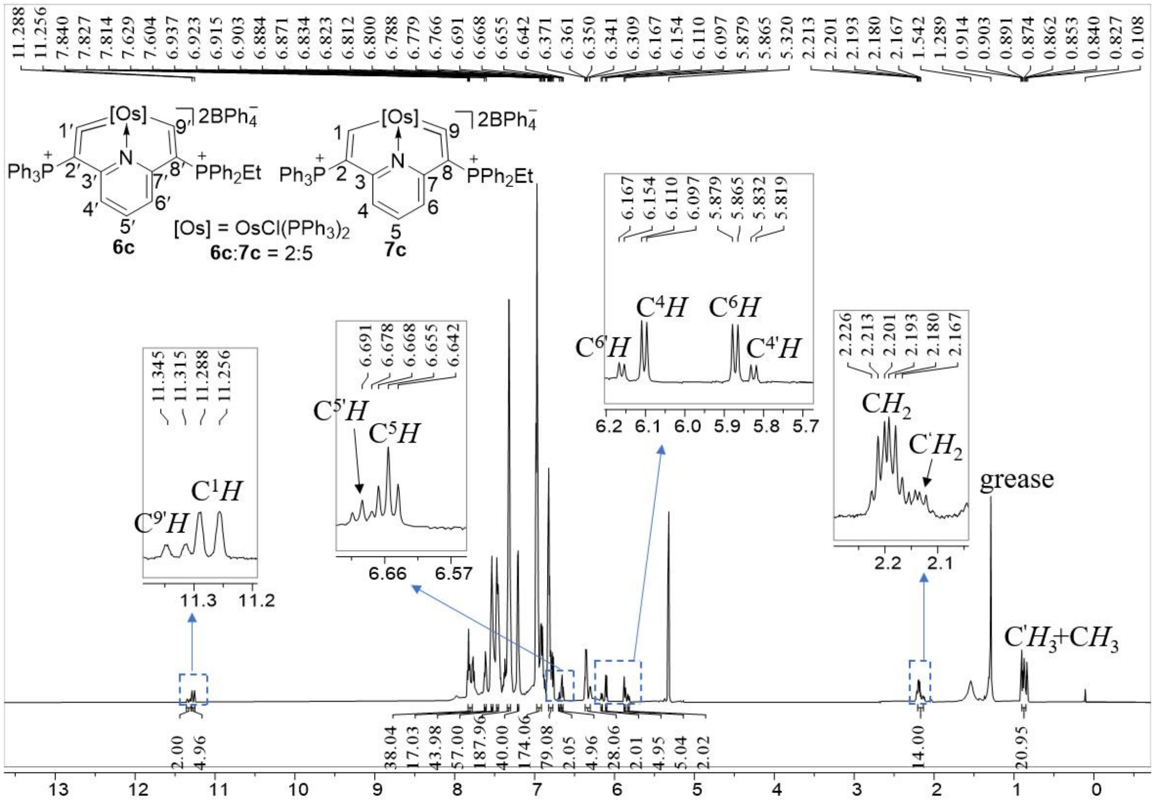

Figure S101. The ^{1}H NMR (600.1 MHz, CD_2Cl_2) spectrum for complexes **6c** and **7c**.

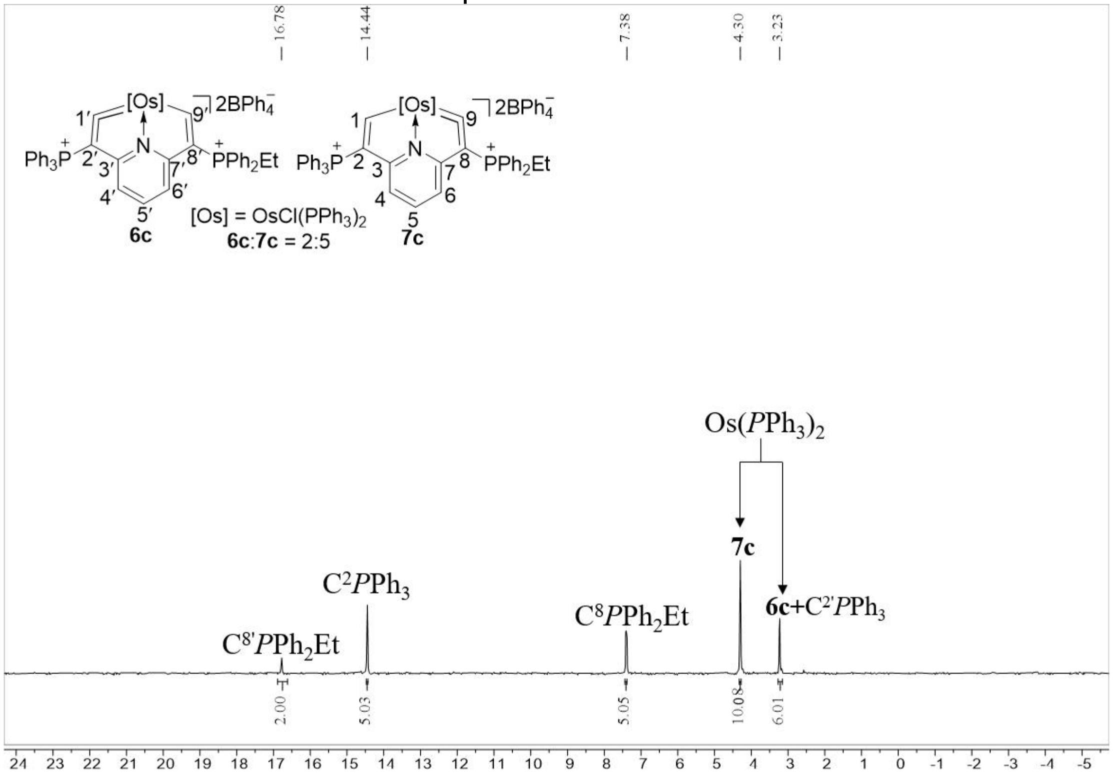

Figure S102. The $^{31}P\{^{1}H\}$ NMR (242.9 MHz, CD_2Cl_2) spectrum for complexes **6c** and **7c**.

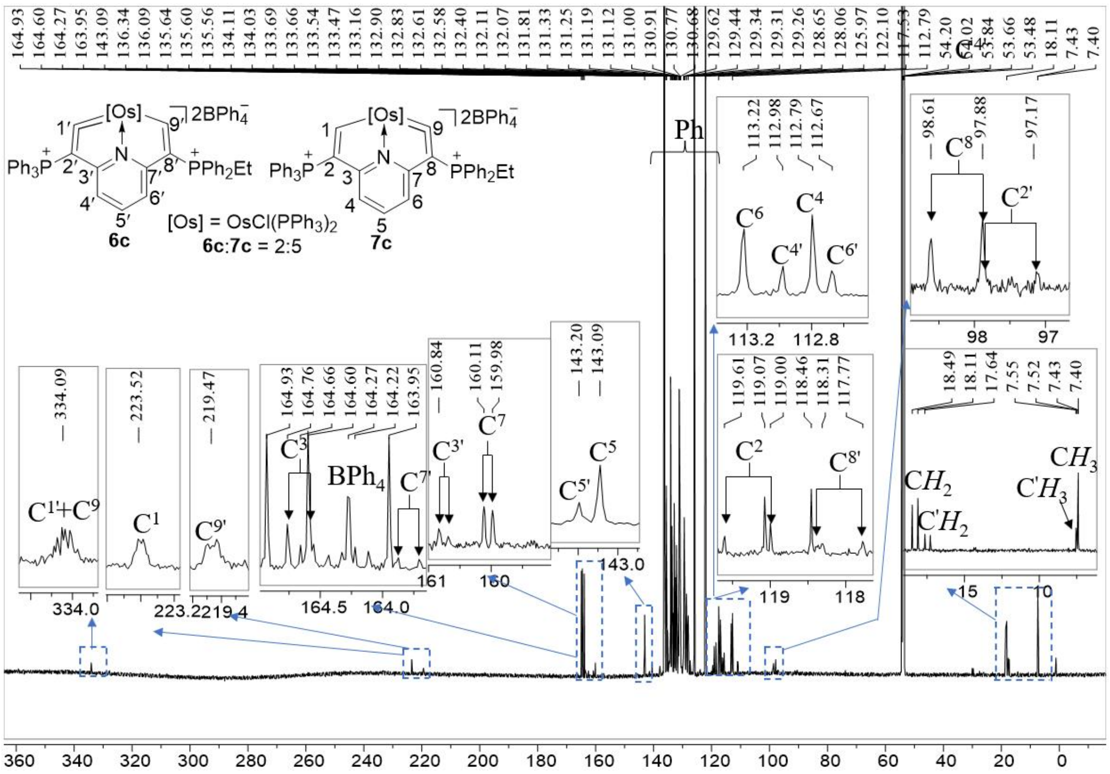

Figure S103. The $^{13}C\{^1H\}$ NMR (150.9 MHz, CD_2Cl_2) spectrum for complexes **6c** and **7c**.

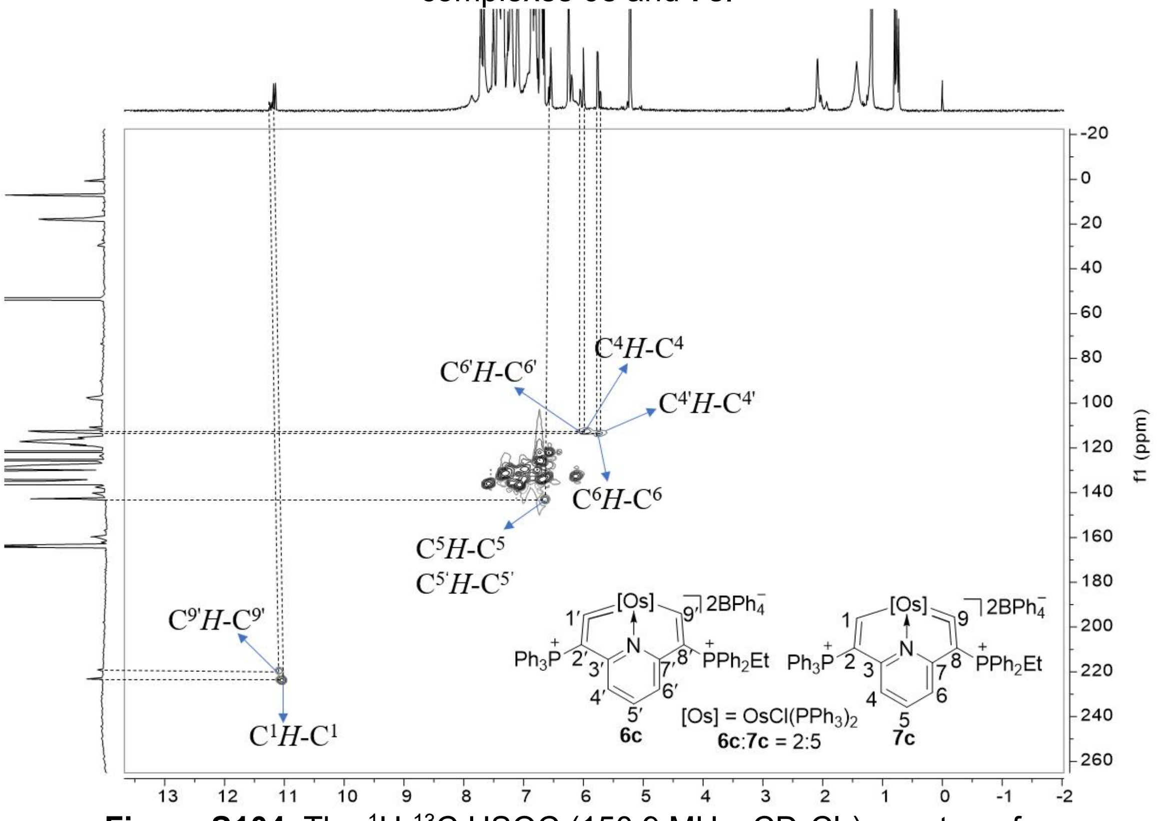

Figure S104. The ^1H-^{13}C HSQC (150.9 MHz, CD_2Cl_2) spectrum for complexes **6c** and **7c**.

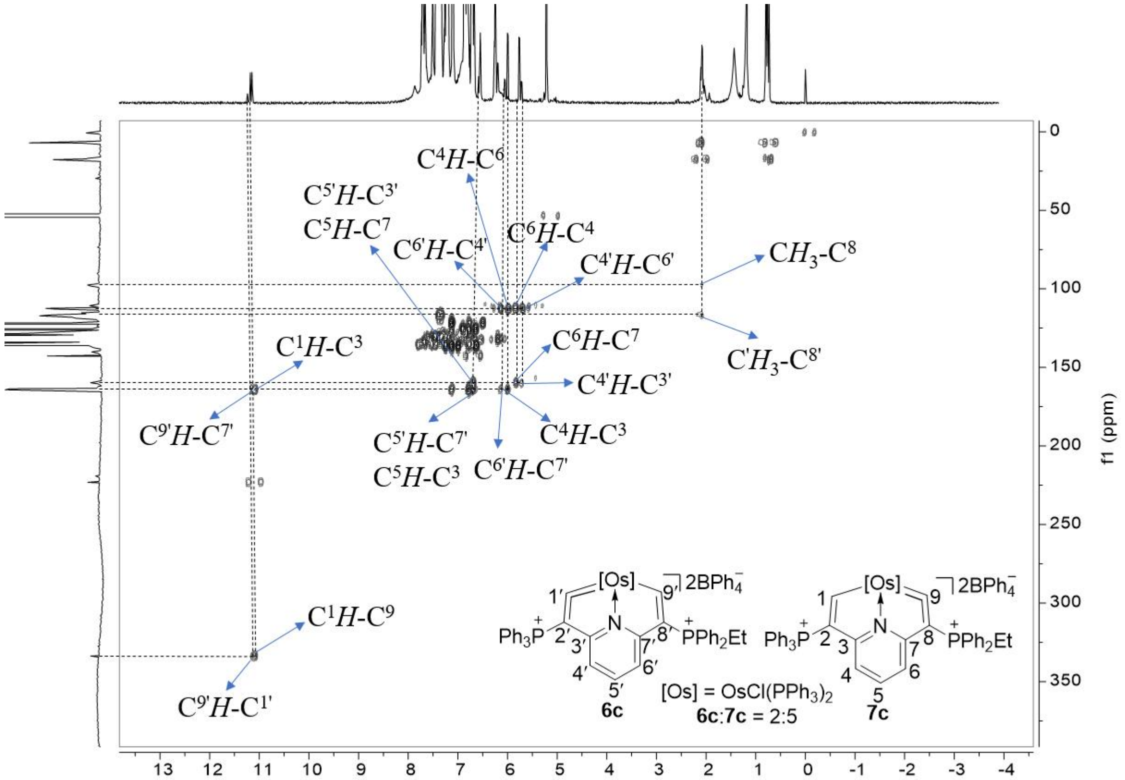

Figure S105. The ^{1}H-^{13}C HMBC (150.9 MHz, CD_2Cl_2) spectrum for complexes **6c** and **7c**.

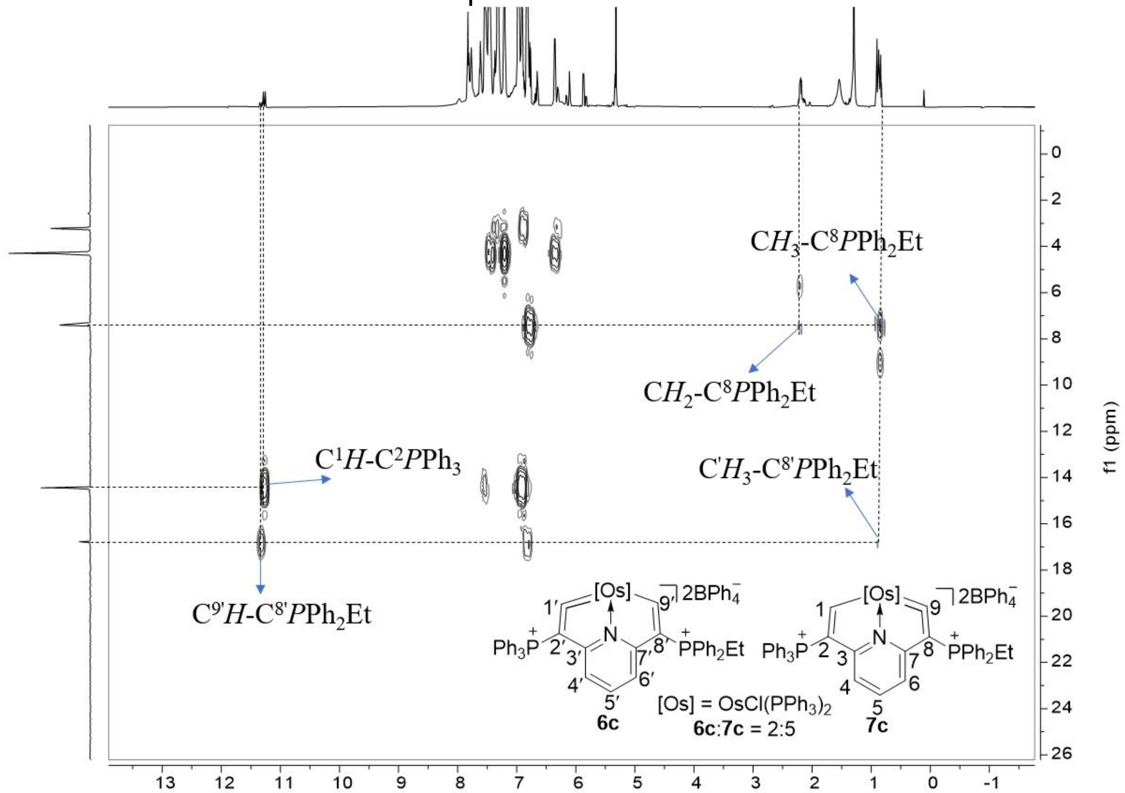

Figure S106. The ^{1}H-^{31}P HMBC (242.9 MHz, CD_2Cl_2) spectrum for complexes **6c** and **7c**.

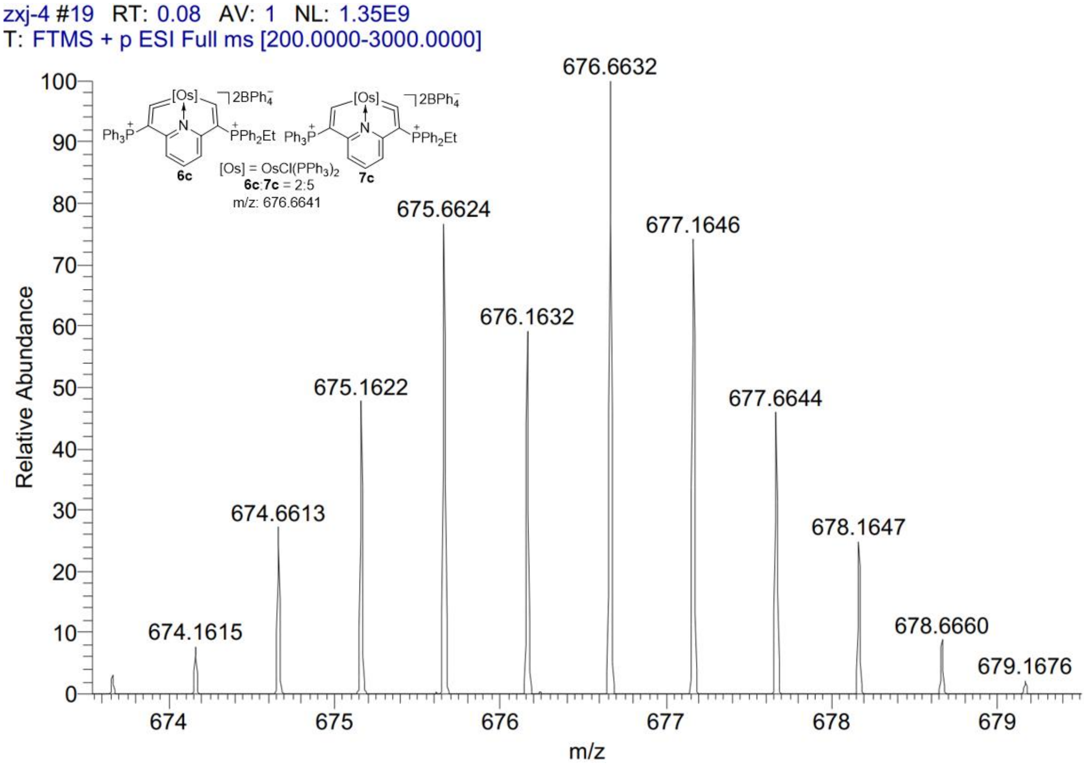

Figure S107. Positive-ion ESI-MS spectrum of [**6c** and **7c**]$^+$ measured in methanol.

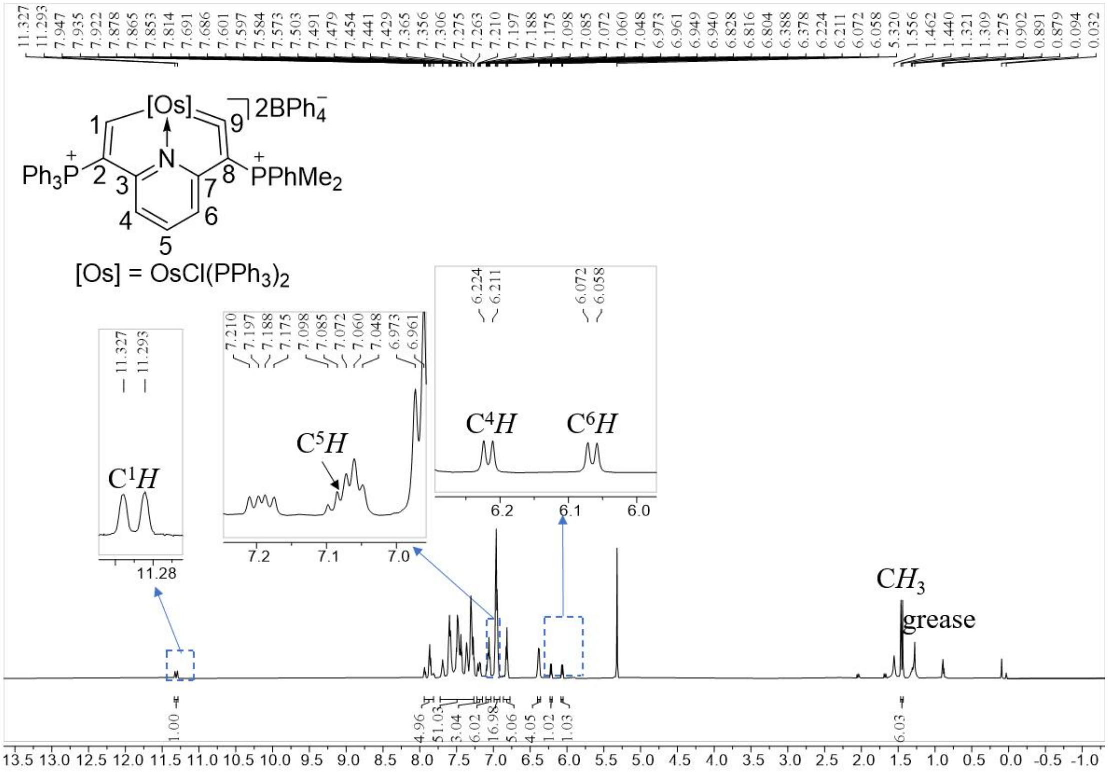

Figure S108. The ^{1}H NMR (600.1 MHz, CD_2Cl_2) spectrum for complex **7a**.

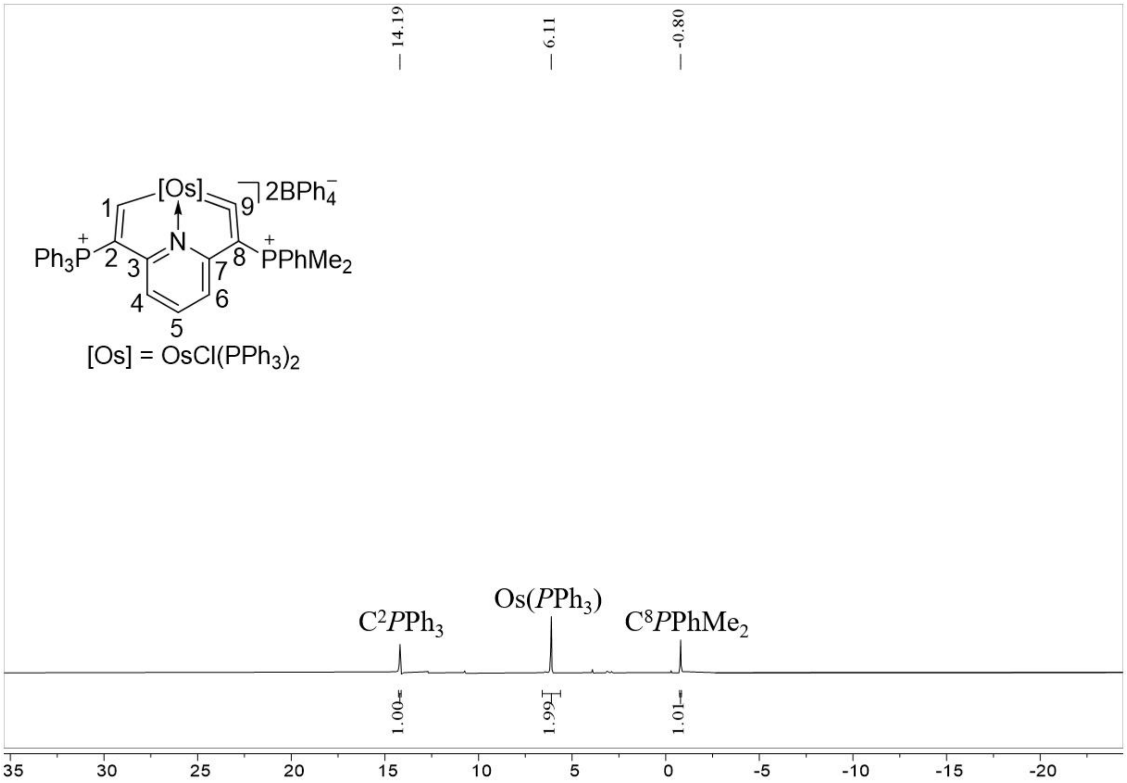

Figure S109. The $^{31}P\{^1H\}$ NMR (242.9 MHz, CD_2Cl_2) spectrum for complex **7a**.

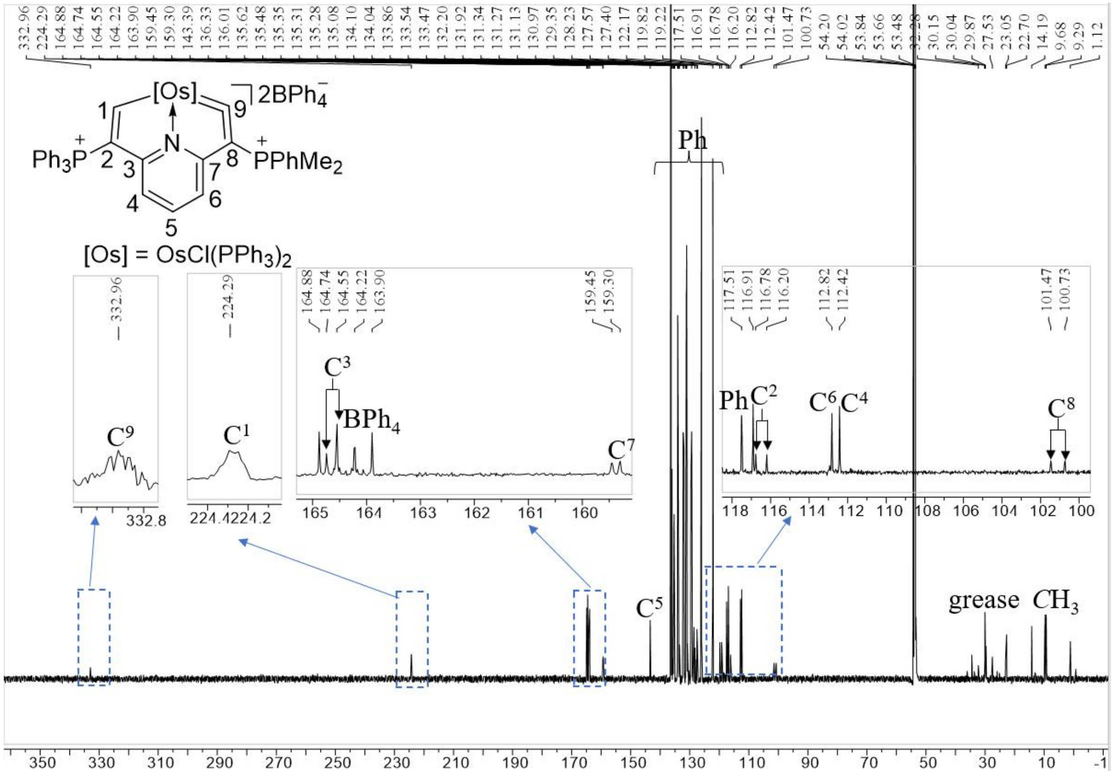

Figure S110. The $^{13}C\{^1H\}$ NMR (150.9 MHz, CD_2Cl_2) spectrum for complex **7a**.

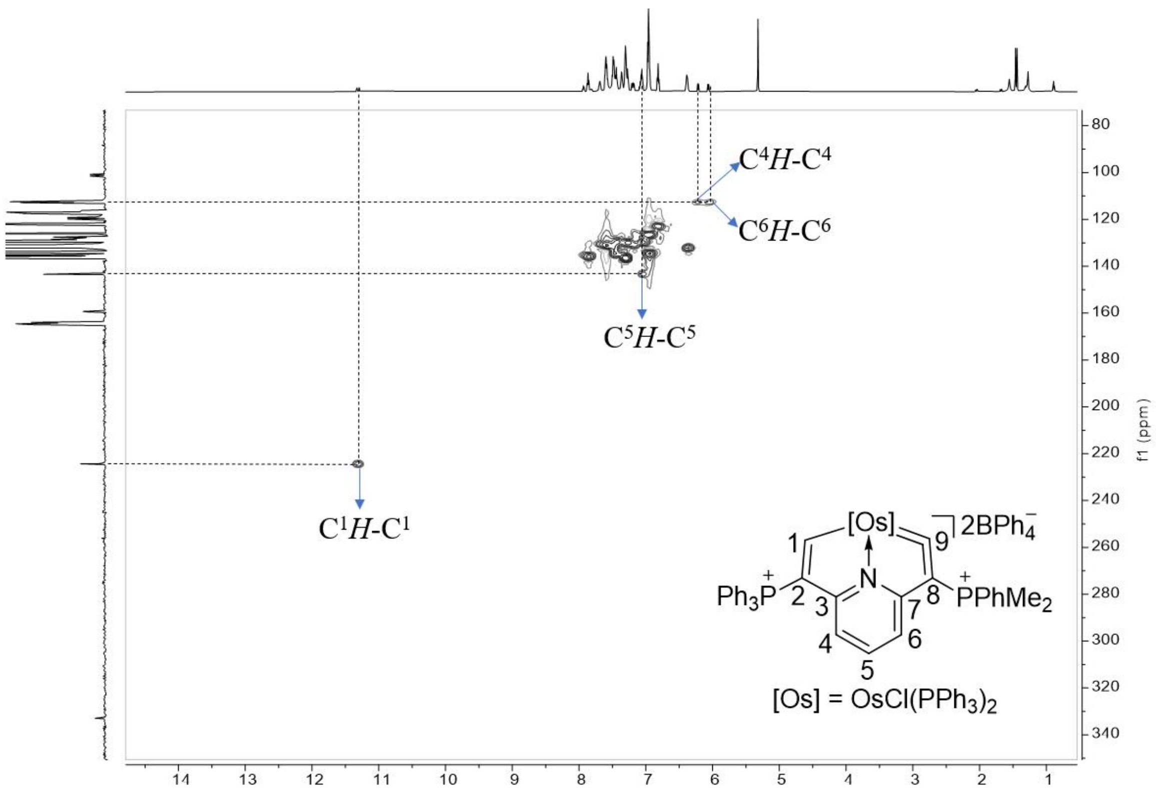

Figure S111. The ^{1}H-^{13}C HSQC (150.9 MHz, CD_2Cl_2) spectrum for complex **7a**.

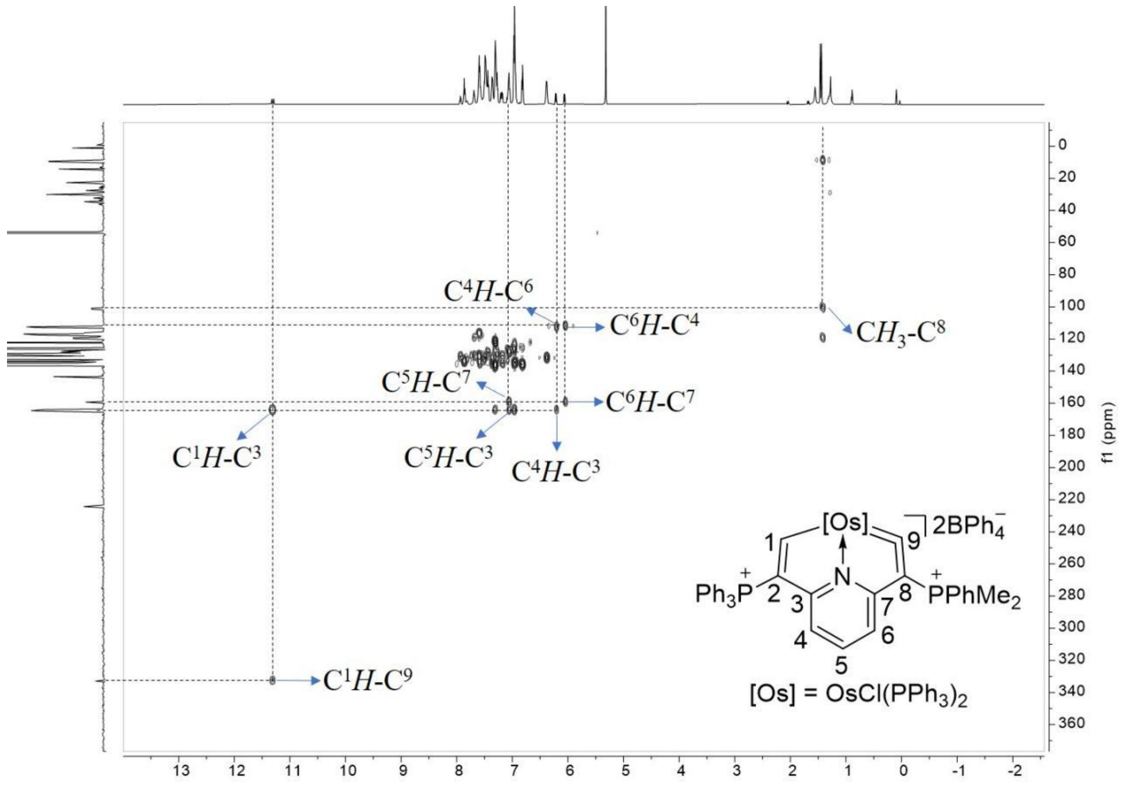

Figure S112. The ^{1}H-^{13}C HMBC (150.9 MHz, CD_2Cl_2) spectrum for complex **7a**.

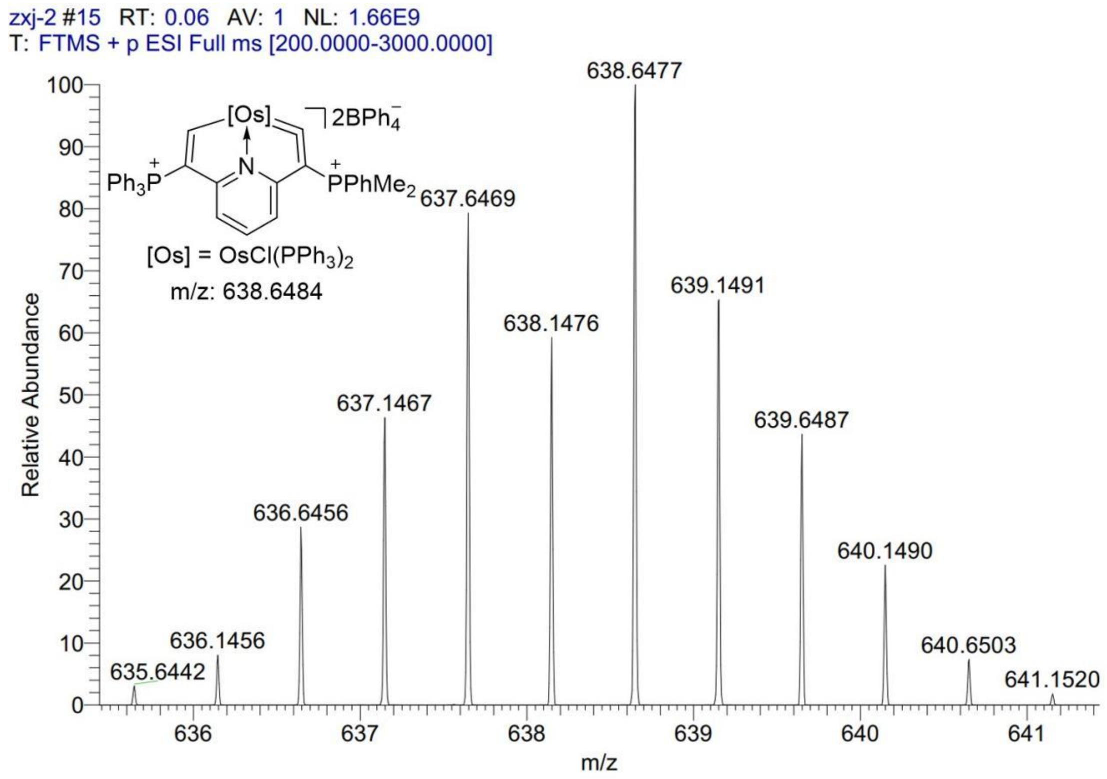

Figure S113. Positive-ion ESI-MS spectrum of [**7a**]$^+$ measured in methanol.

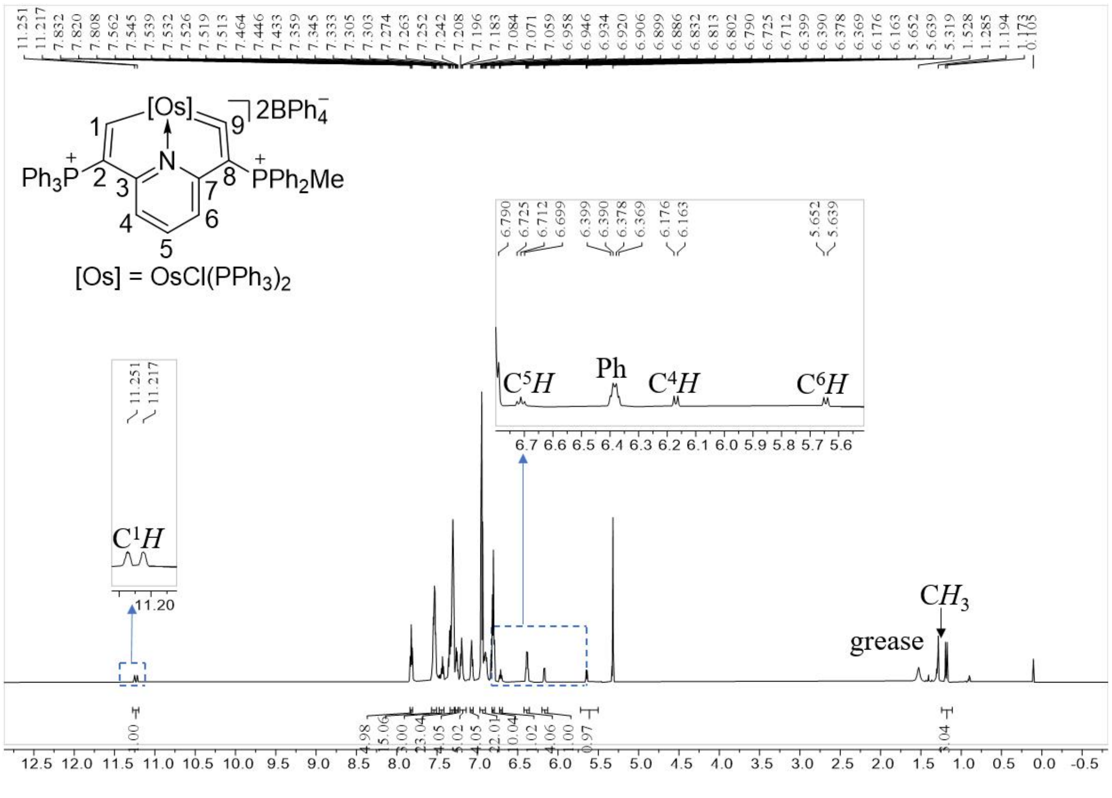

Figure S114. The ^{1}H NMR (600.1 MHz, CD_2Cl_2) spectrum for complex **7b**.

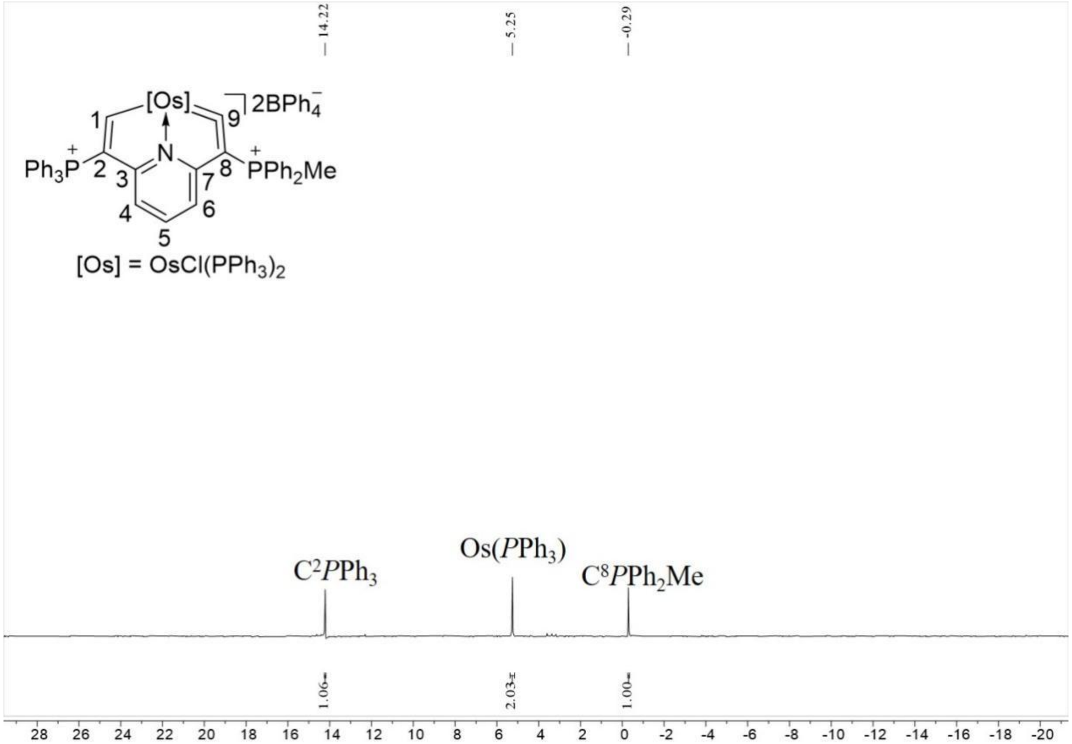

Figure S115. The $^{31}P\{^{1}H\}$ NMR (242.9 MHz, CD_2Cl_2) spectrum for complex **7b**.

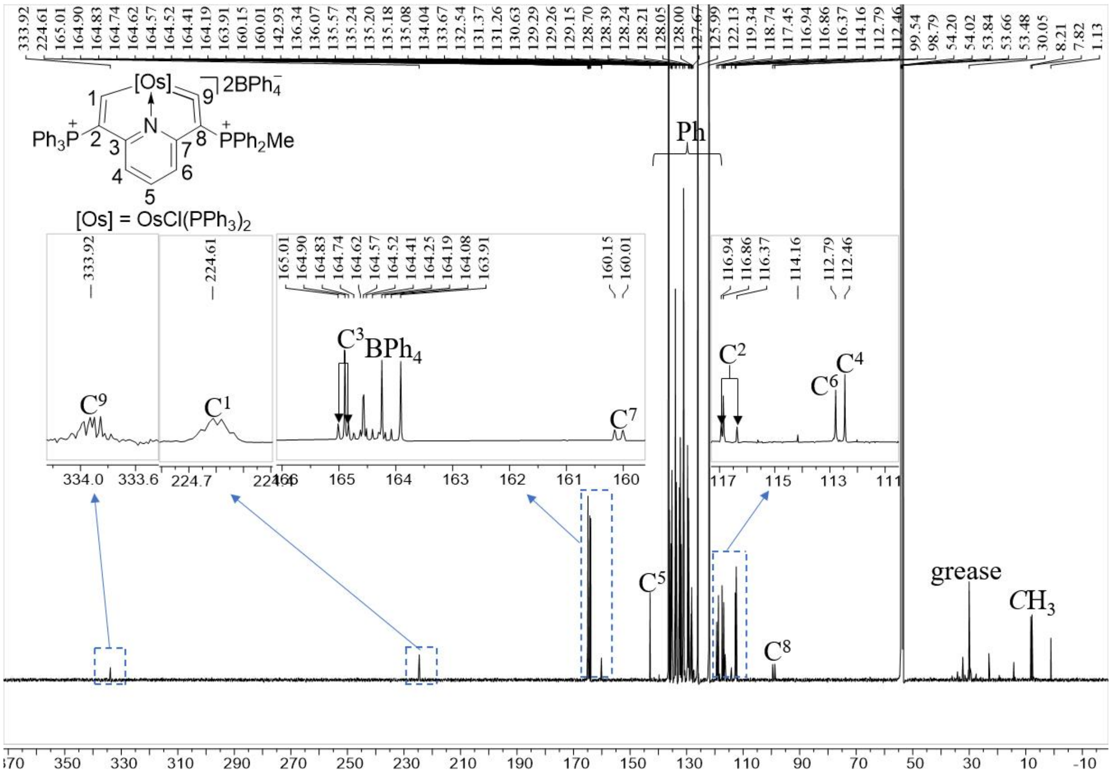

Figure S116. The $^{13}C\{^{1}H\}$ NMR (150.9 MHz, CD_2Cl_2) spectrum for complex **7b**.

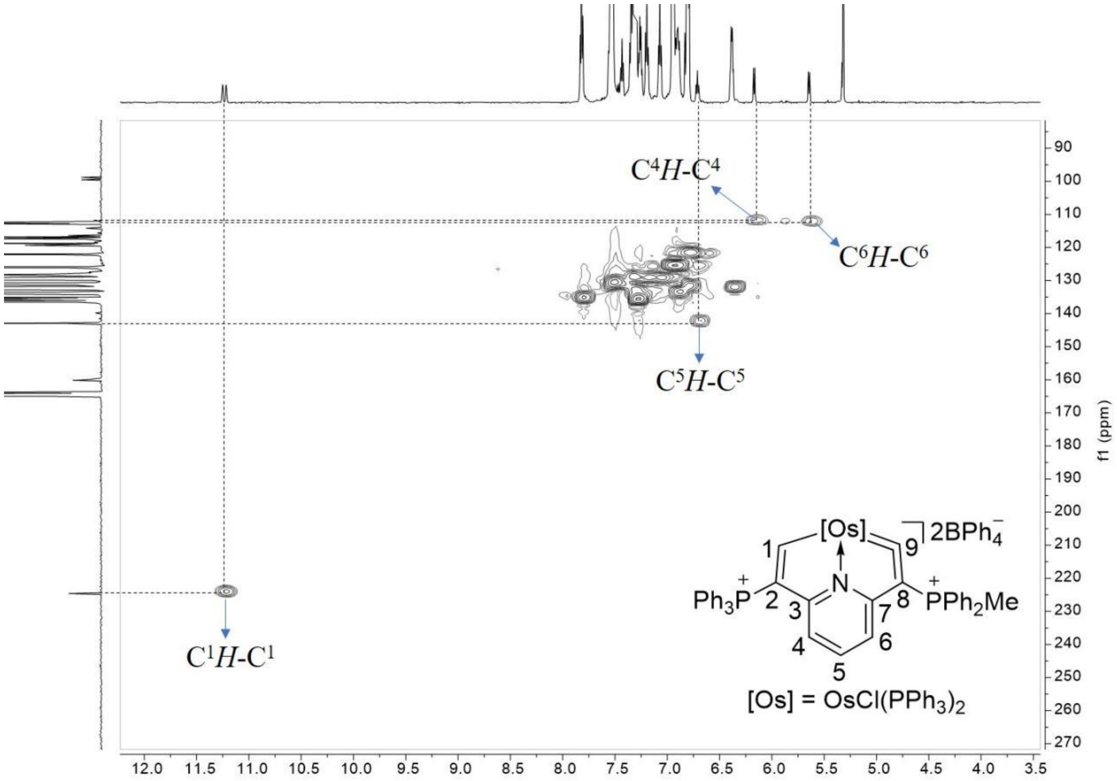

Figure S117. The ^{1}H-^{13}C HSQC (150.9 MHz, CD_2Cl_2) spectrum for complex **7b**.

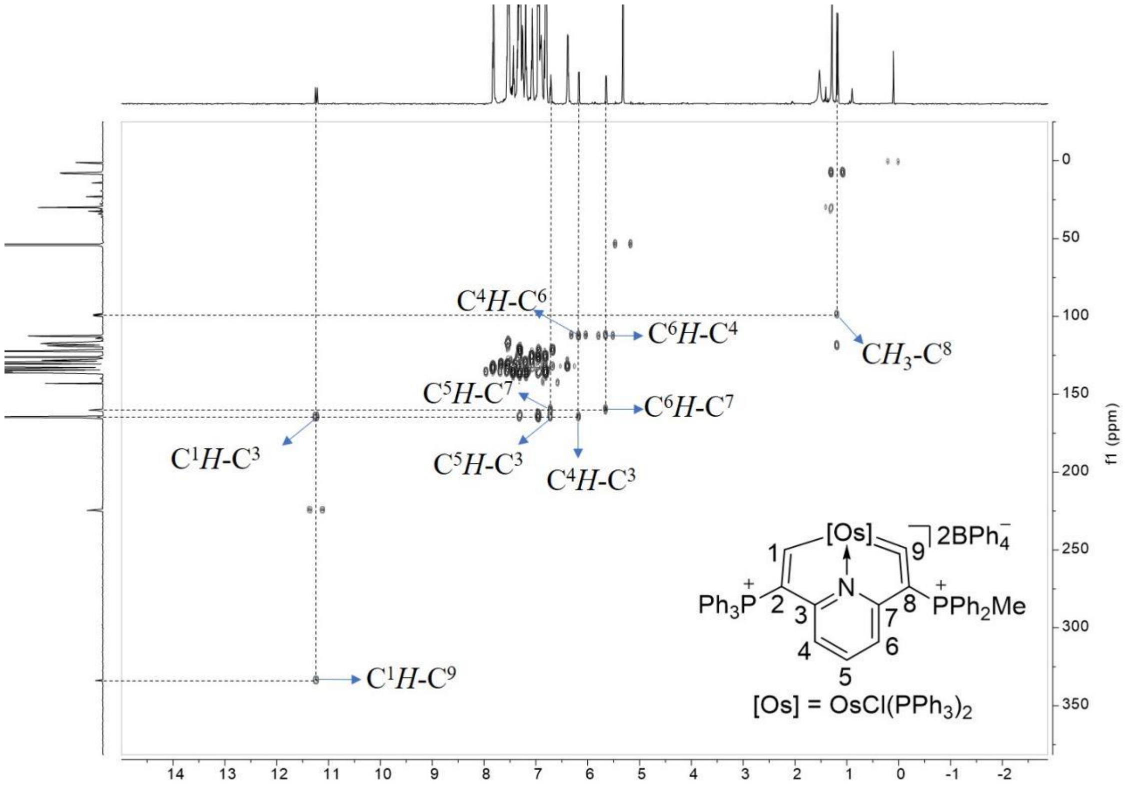

Figure S118. The ^{1}H-^{13}C HMBC (150.9 MHz, CD_2Cl_2) spectrum for complex **7b**.

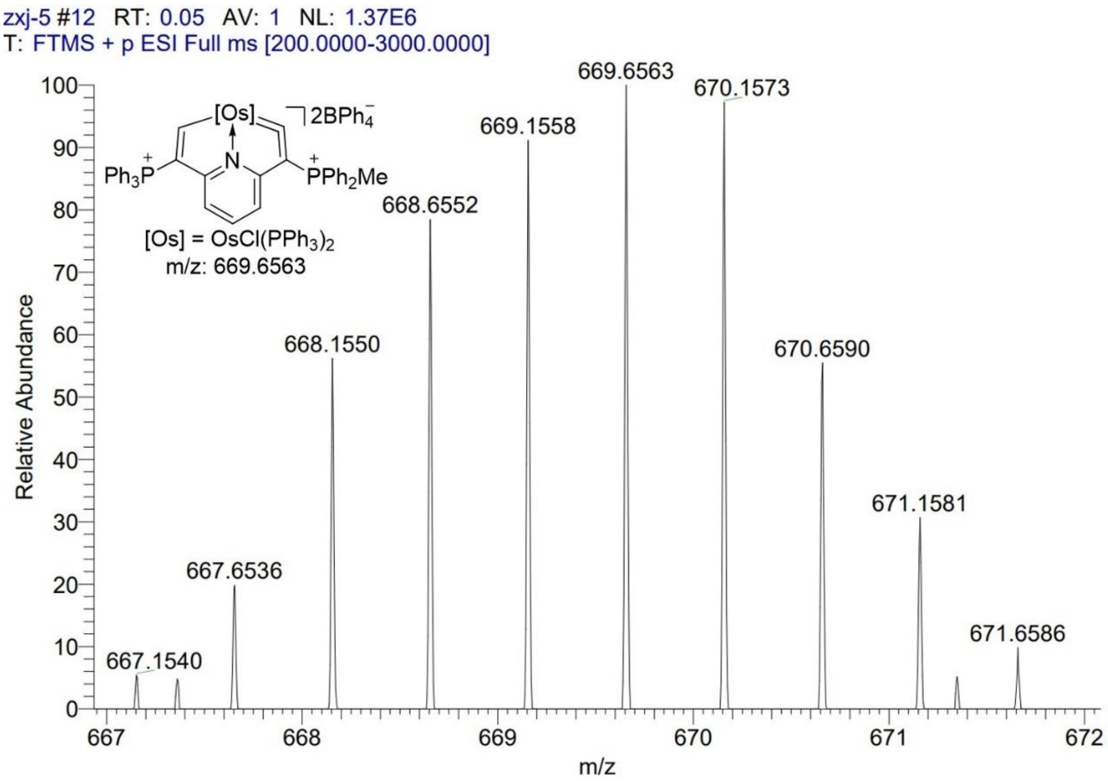

Figure S119. Positive-ion ESI-MS spectrum of [**7b**]$^{+}$ measured in methanol.

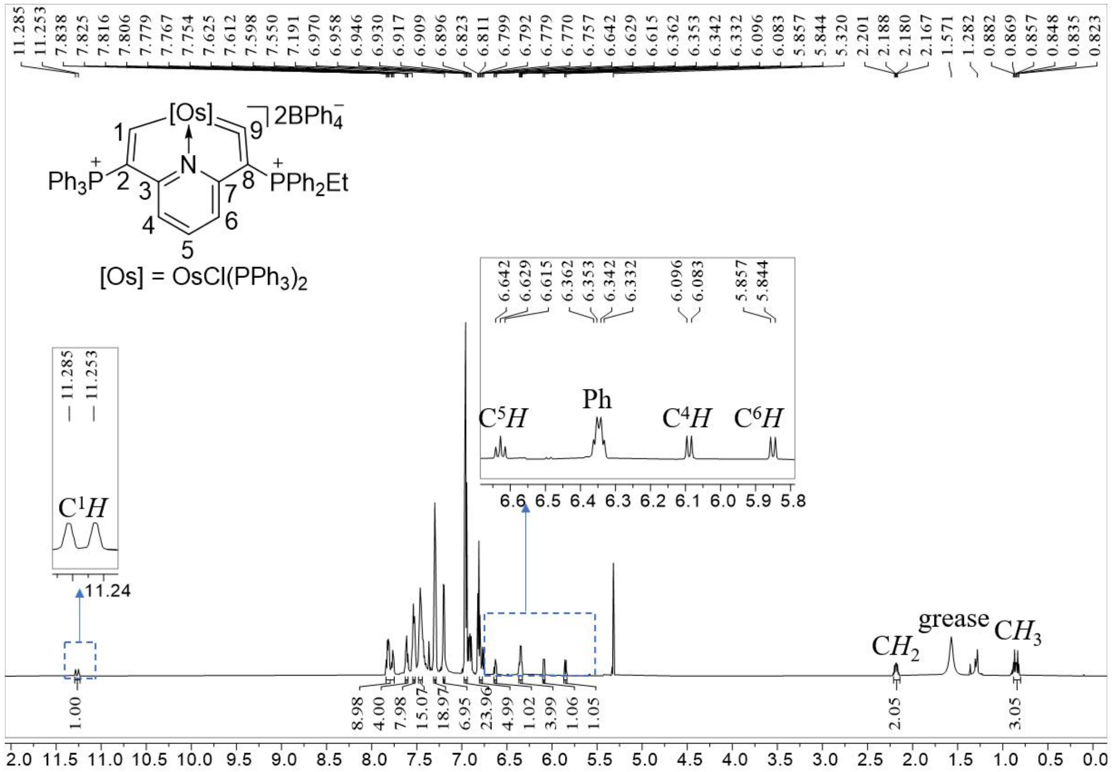

Figure S120. The ^{1}H NMR (600.1 MHz, CD_2Cl_2) spectrum for complex **7c**.

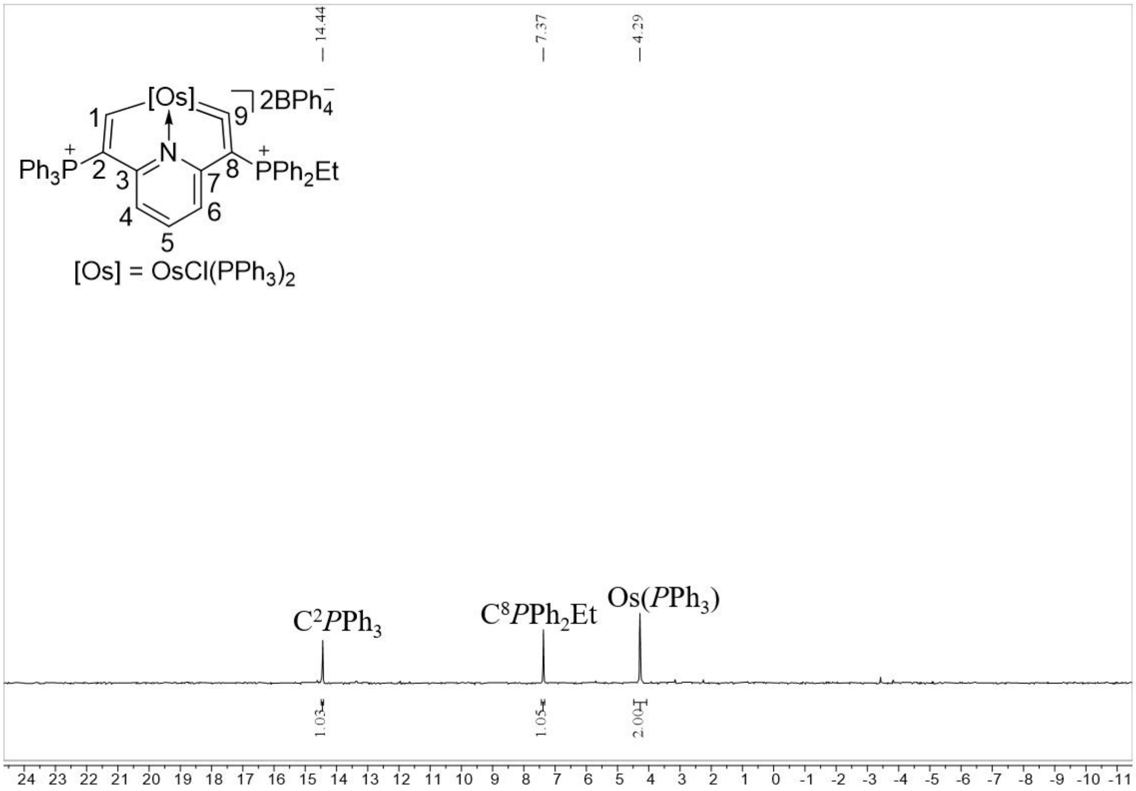

Figure S121. The $^{31}P\{^{1}H\}$ NMR (242.9 MHz, CD_2Cl_2) spectrum for complex **7c**.

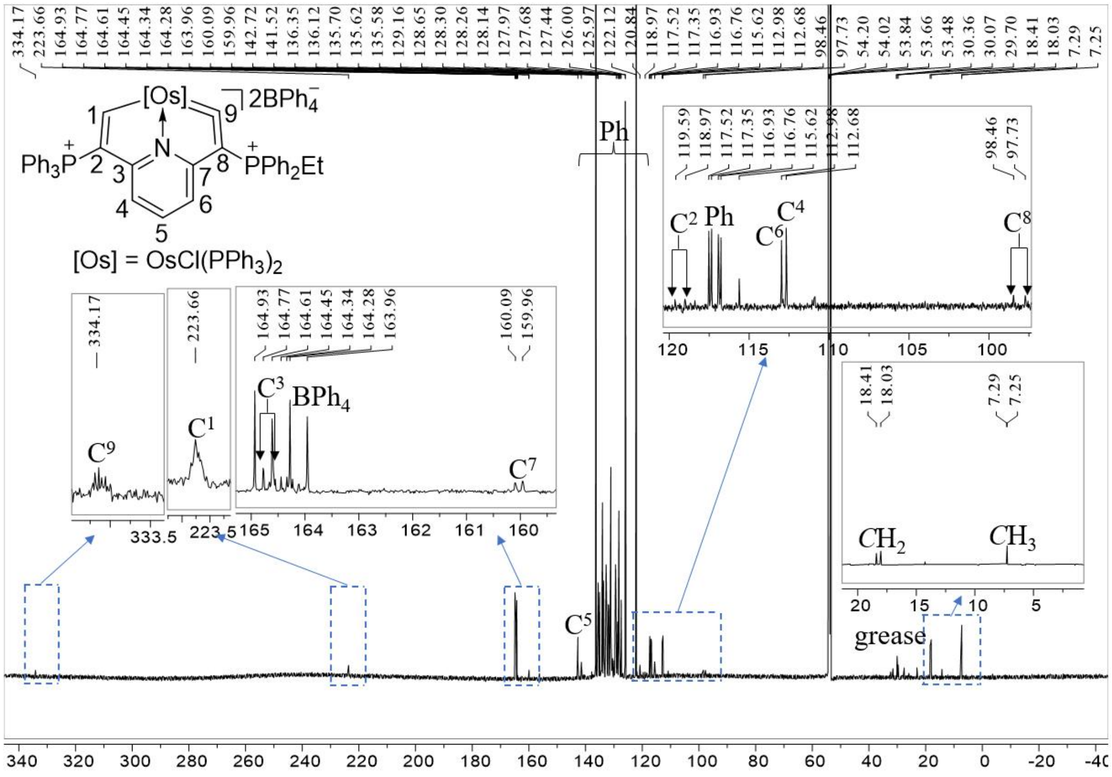

Figure S122. The $^{13}C\{^1H\}$ NMR (150.9 MHz, CD_2Cl_2) spectrum for complex **7c**.

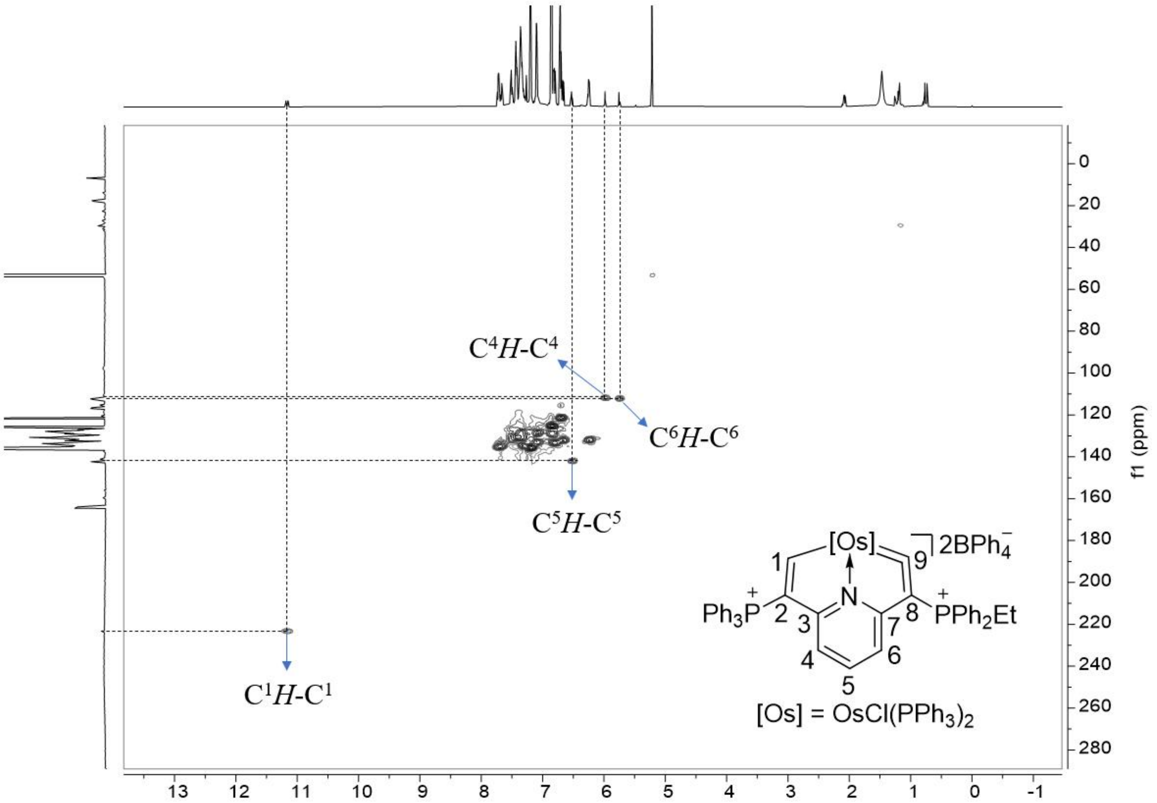

Figure S123. The ^1H-^{13}C HSQC (150.9 MHz, CD_2Cl_2) spectrum for complex **7c**.

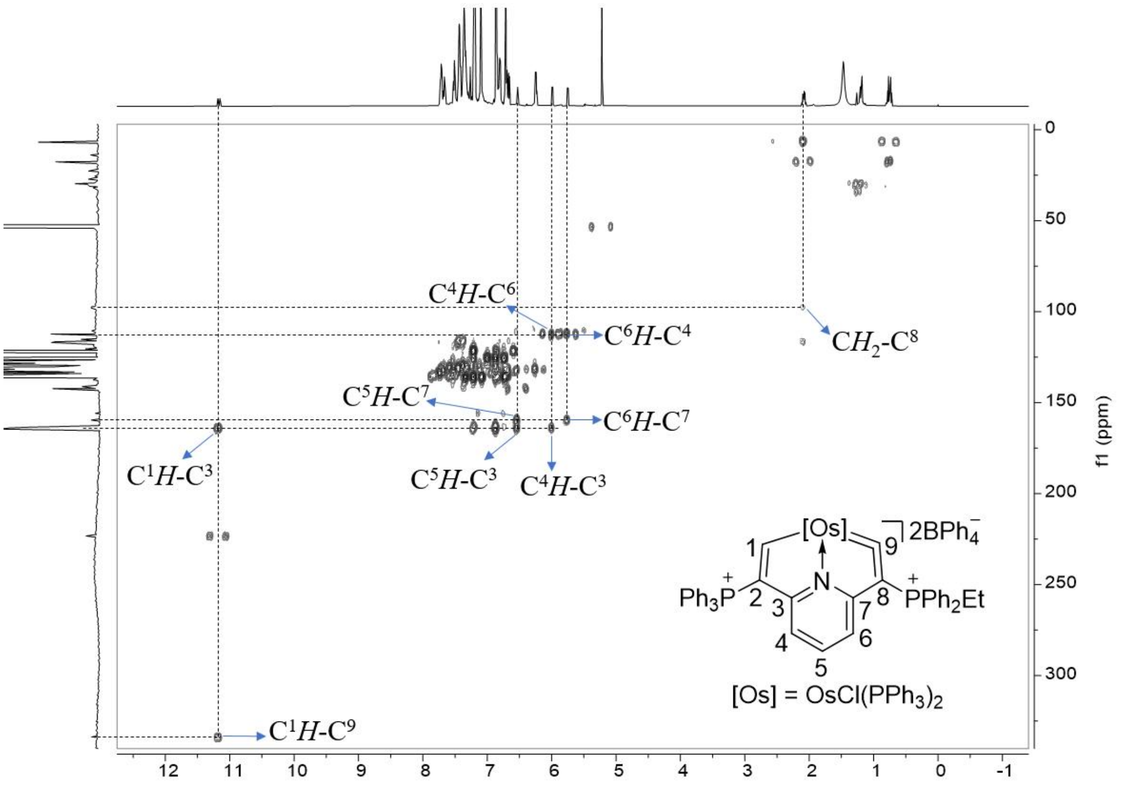

Figure S124. The ^{1}H-^{13}C HMBC (150.9 MHz, CD_2Cl_2) spectrum for complex **7c**.

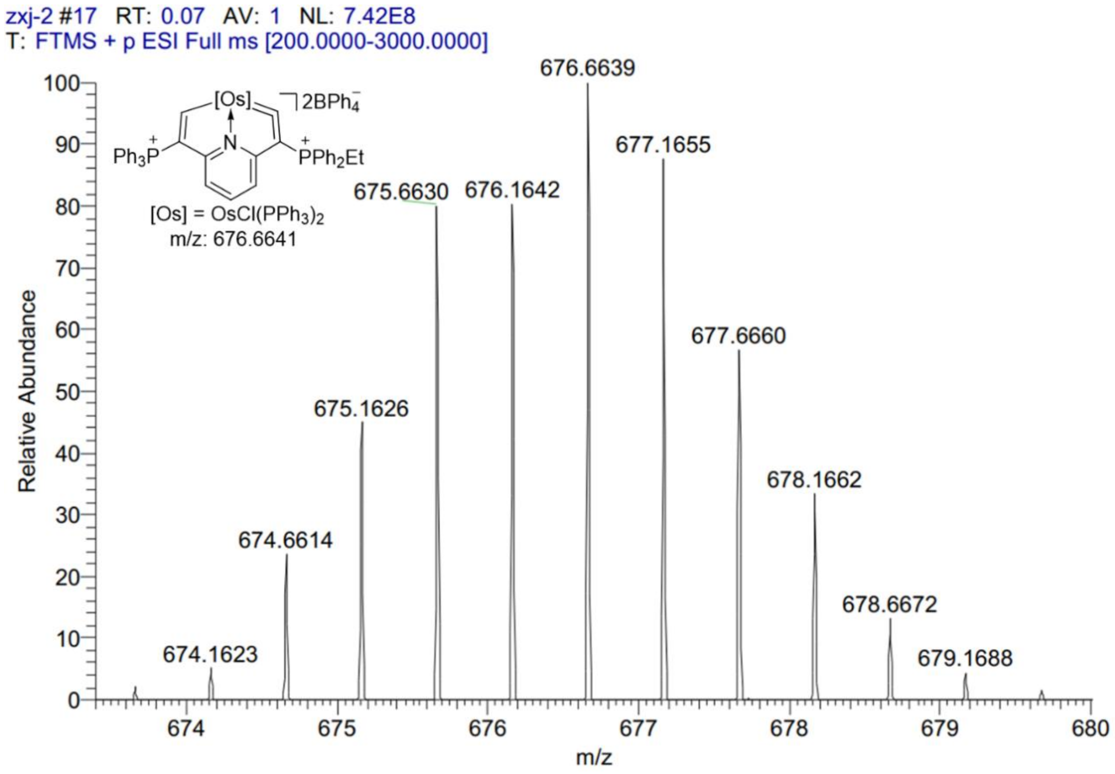

Figure S125. Positive-ion ESI-MS spectrum of [**7c**]$^{+}$ measured in methanol.

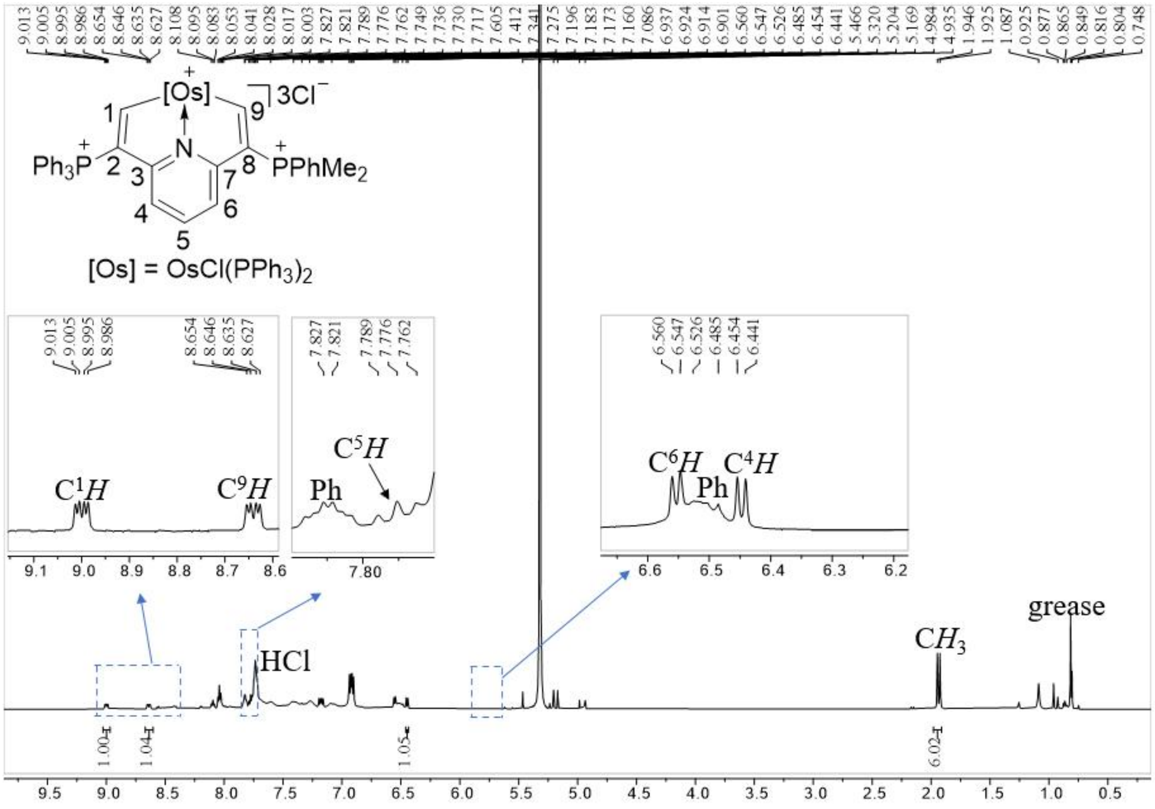

Figure S126. The ^{1}H NMR (600.1 MHz, CD_2Cl_2) spectrum for complex **8a**.

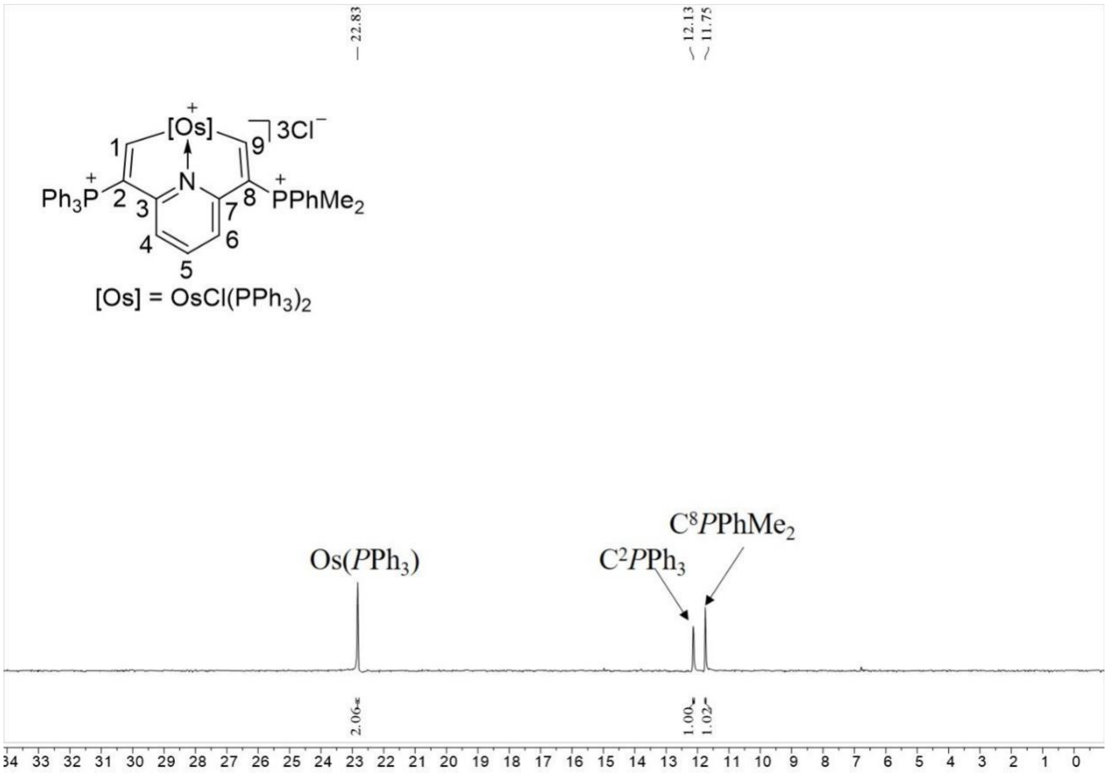

Figure S127. The $^{31}P\{^{1}H\}$ NMR (242.9 MHz, CD_2Cl_2) spectrum for complex **8a**.

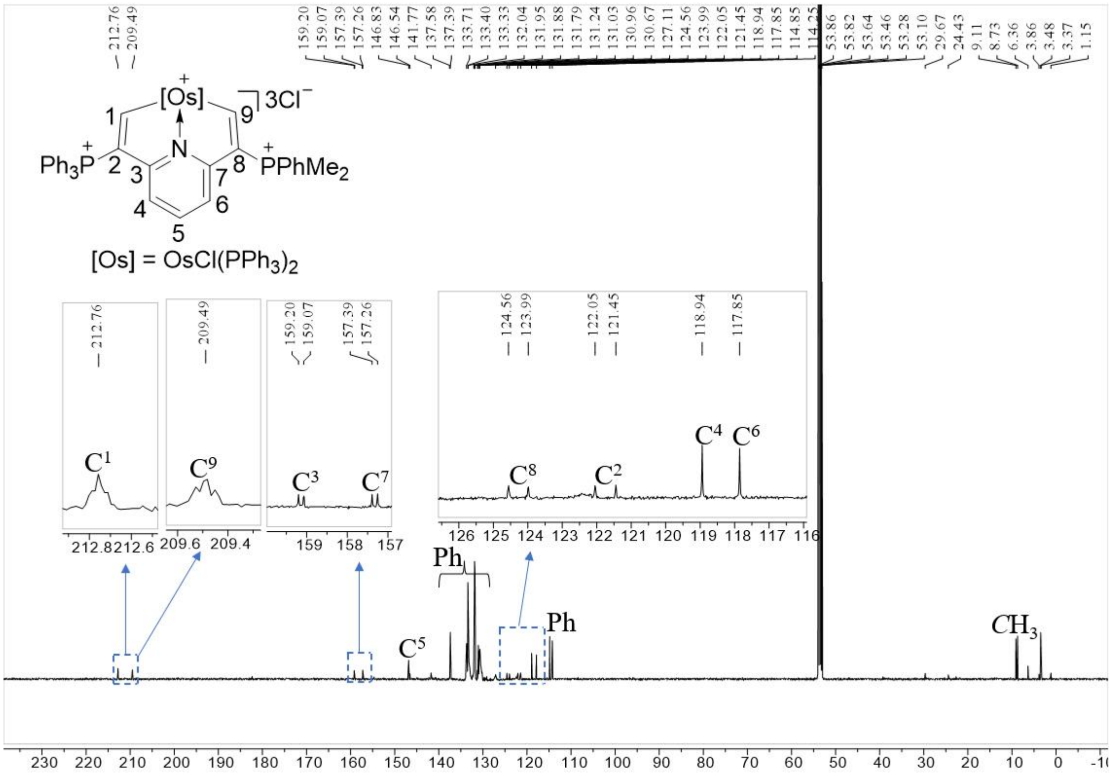

Figure S128. The $^{13}C\{^{1}H\}$ NMR (150.9 MHz, CD_2Cl_2) spectrum for complex **8a**.

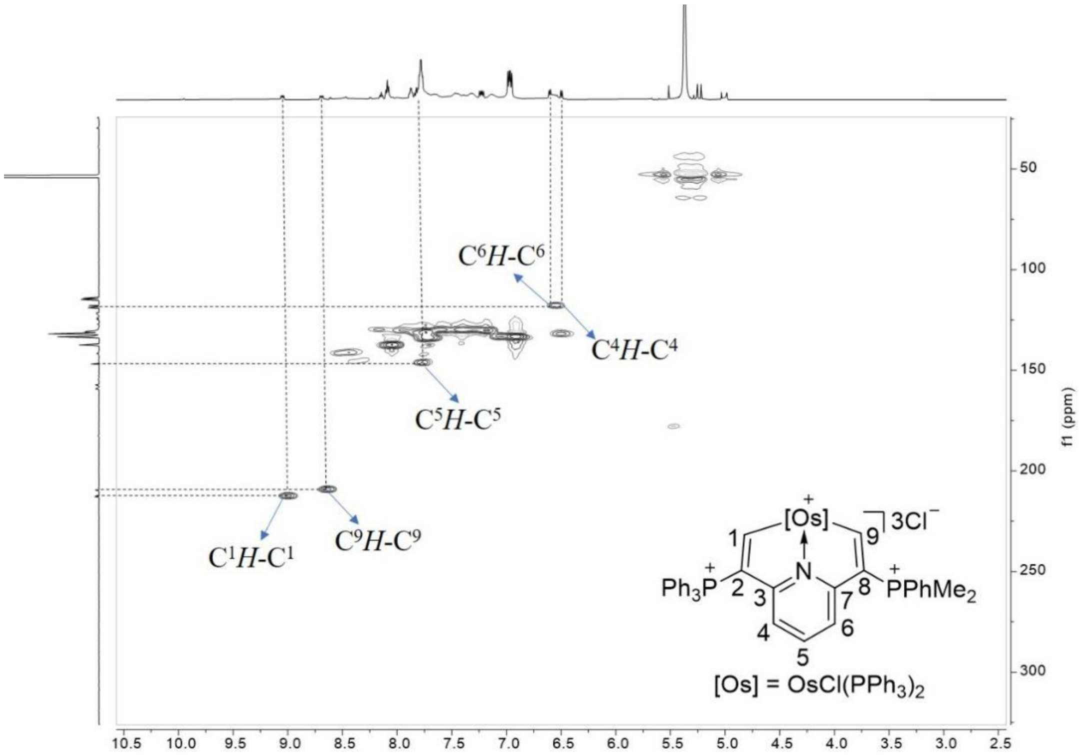

Figure S129. The ^{1}H-^{13}C HSQC (150.9 MHz, CD_2Cl_2) spectrum for complex **8a**.

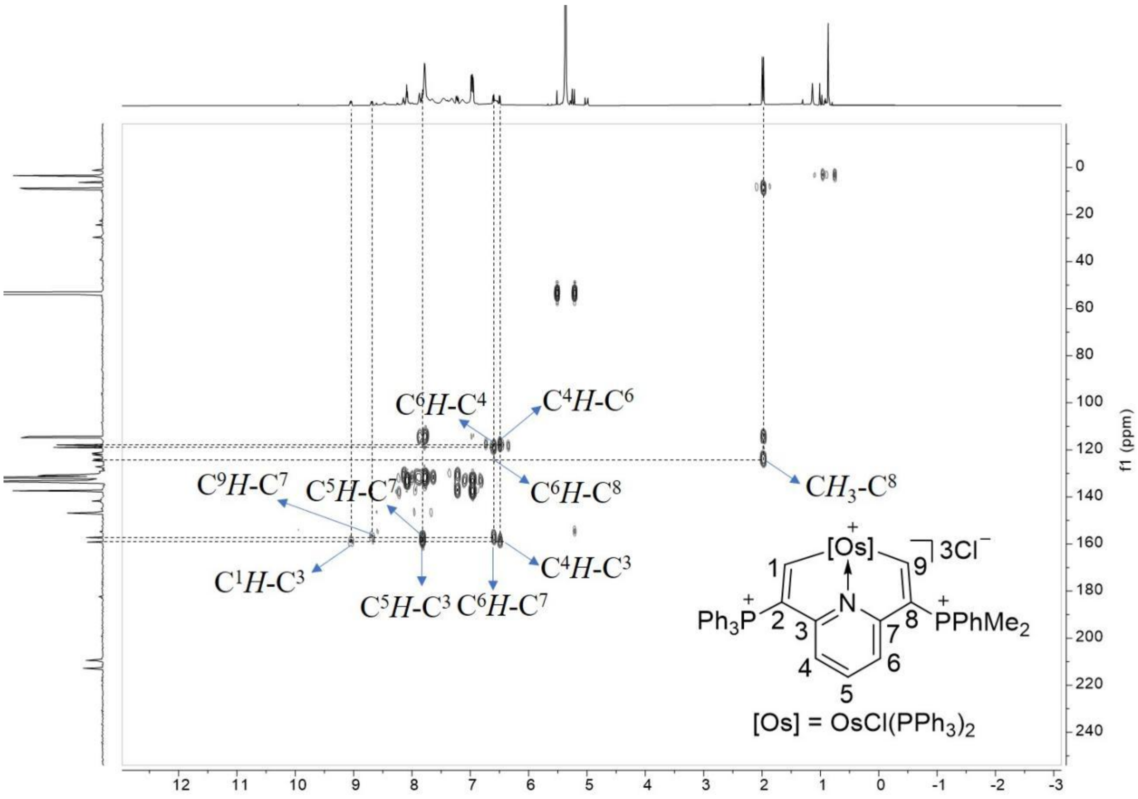

Figure S130. The ^{1}H-^{13}C HMBC (150.9 MHz, CD_2Cl_2) spectrum for complex **8a**.

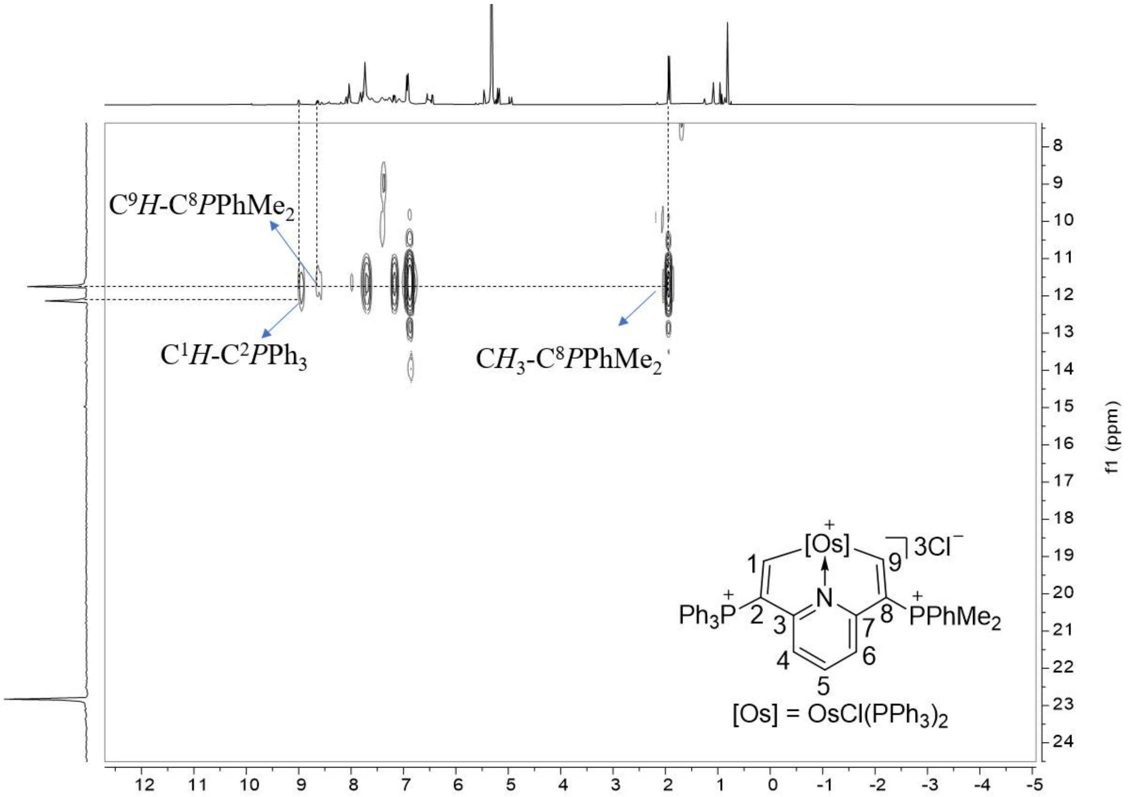

Figure S131. The ^{1}H-^{31}P HMBC (242.9 MHz, CD_2Cl_2) spectrum for complex **8a**.

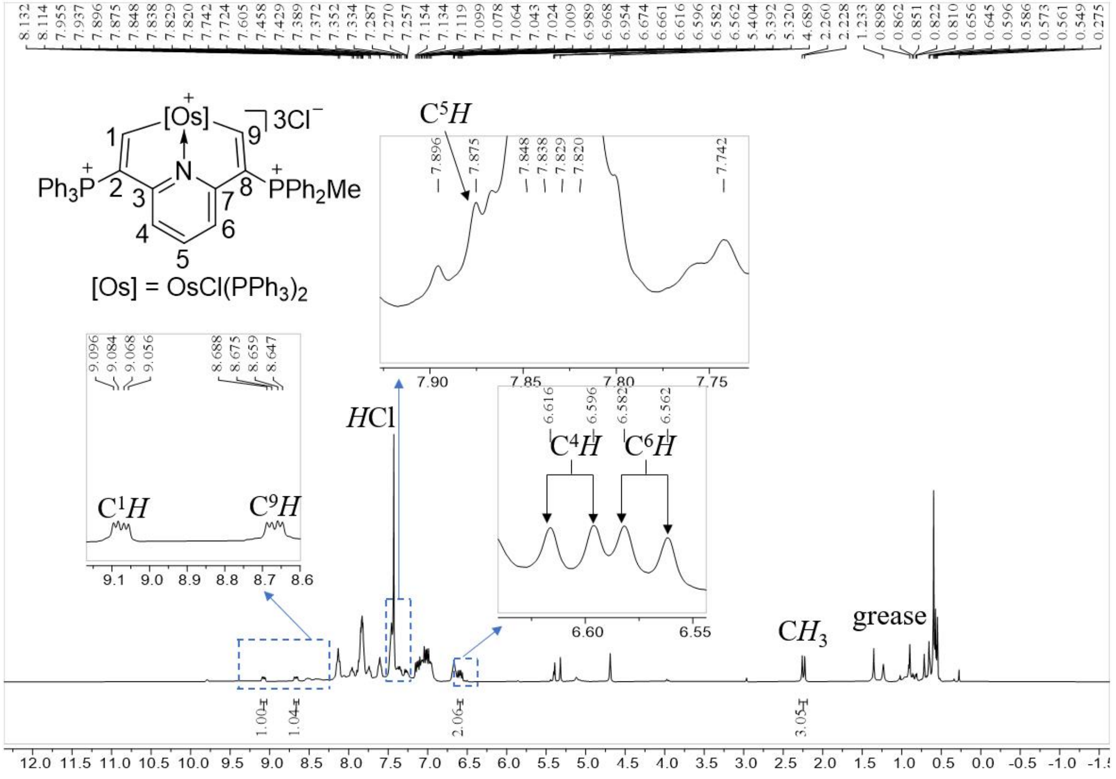

Figure S132. The ^{1}H NMR (400.1 MHz, CD_2Cl_2) spectrum for complex **8b**.

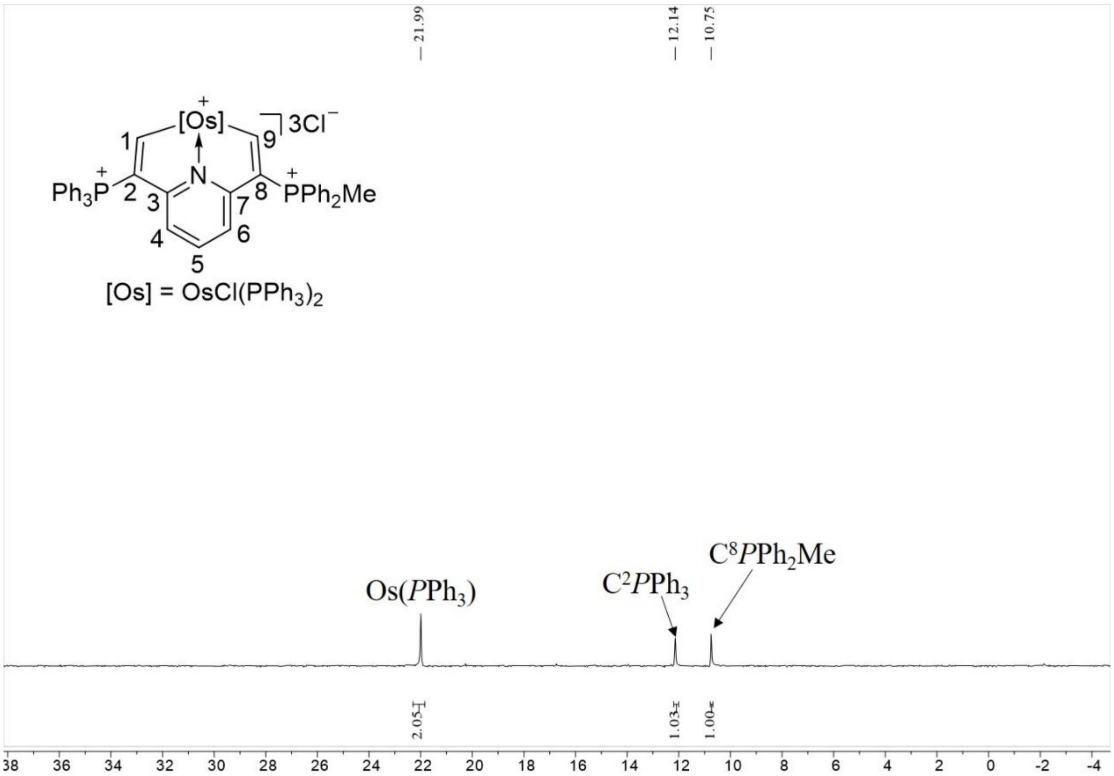

Figure S133. The $^{31}P\{^{1}H\}$ NMR (161.9 MHz, CD_2Cl_2) spectrum for complex **8b**.

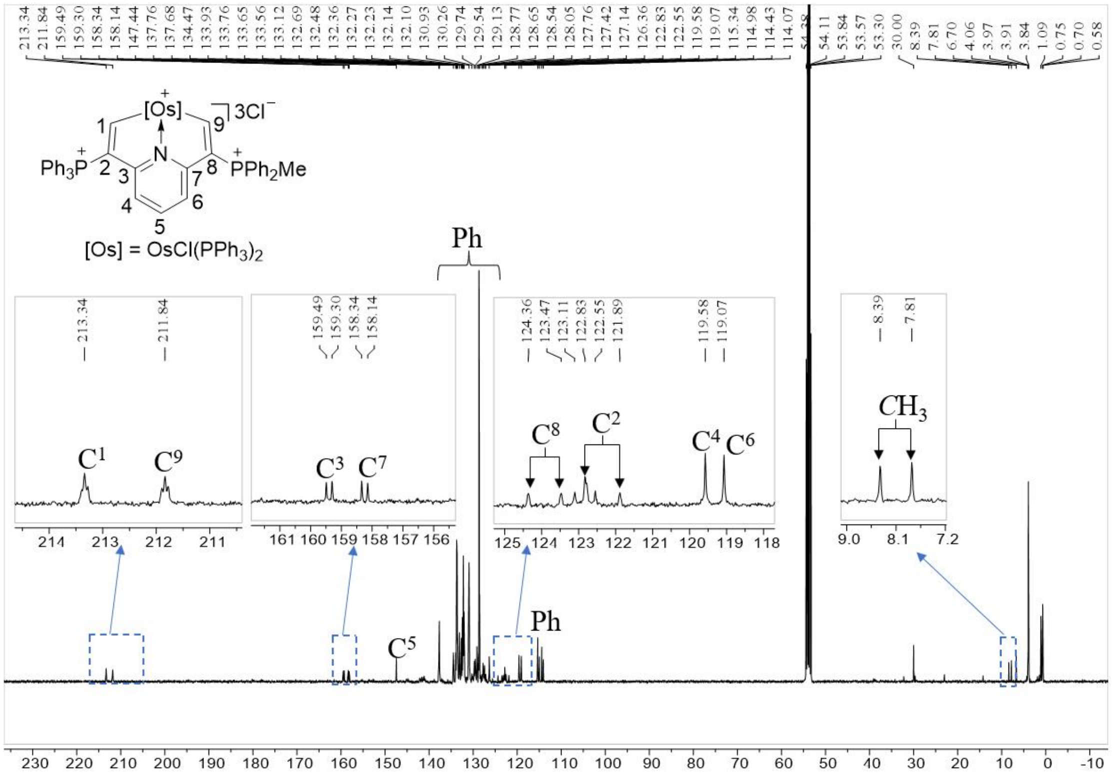

Figure S134. The $^{13}C\{^{1}H\}$ NMR (100.6 MHz, CD_2Cl_2) spectrum for complex **8b**.

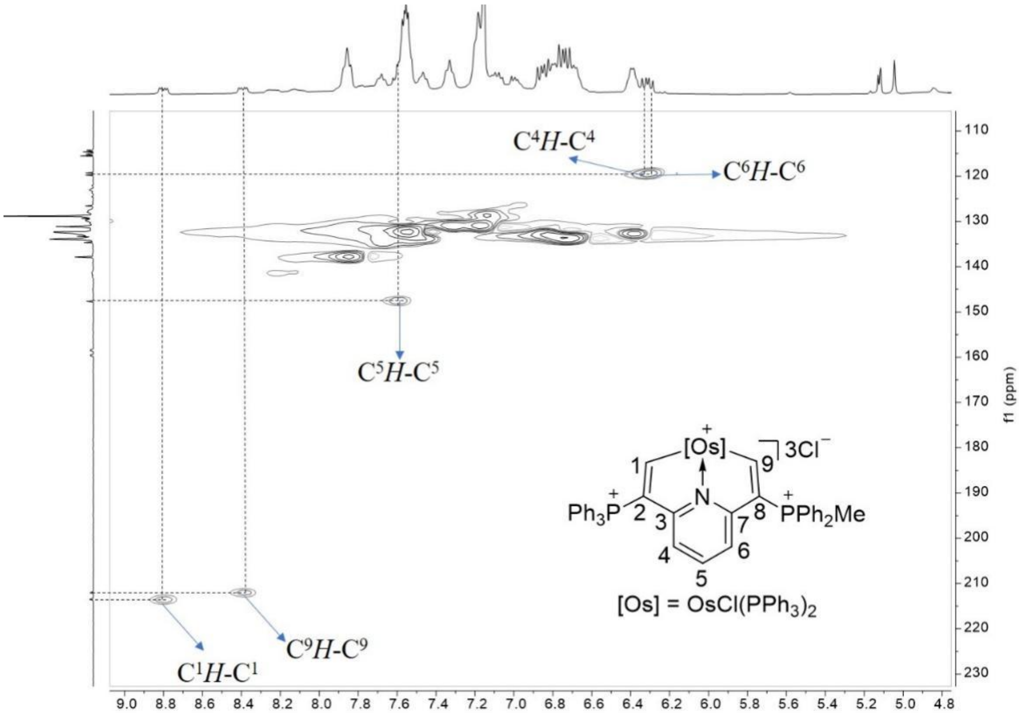

Figure S135. The ^{1}H-^{13}C HSQC (150.9 MHz, CD_2Cl_2) spectrum for complex **8b**.

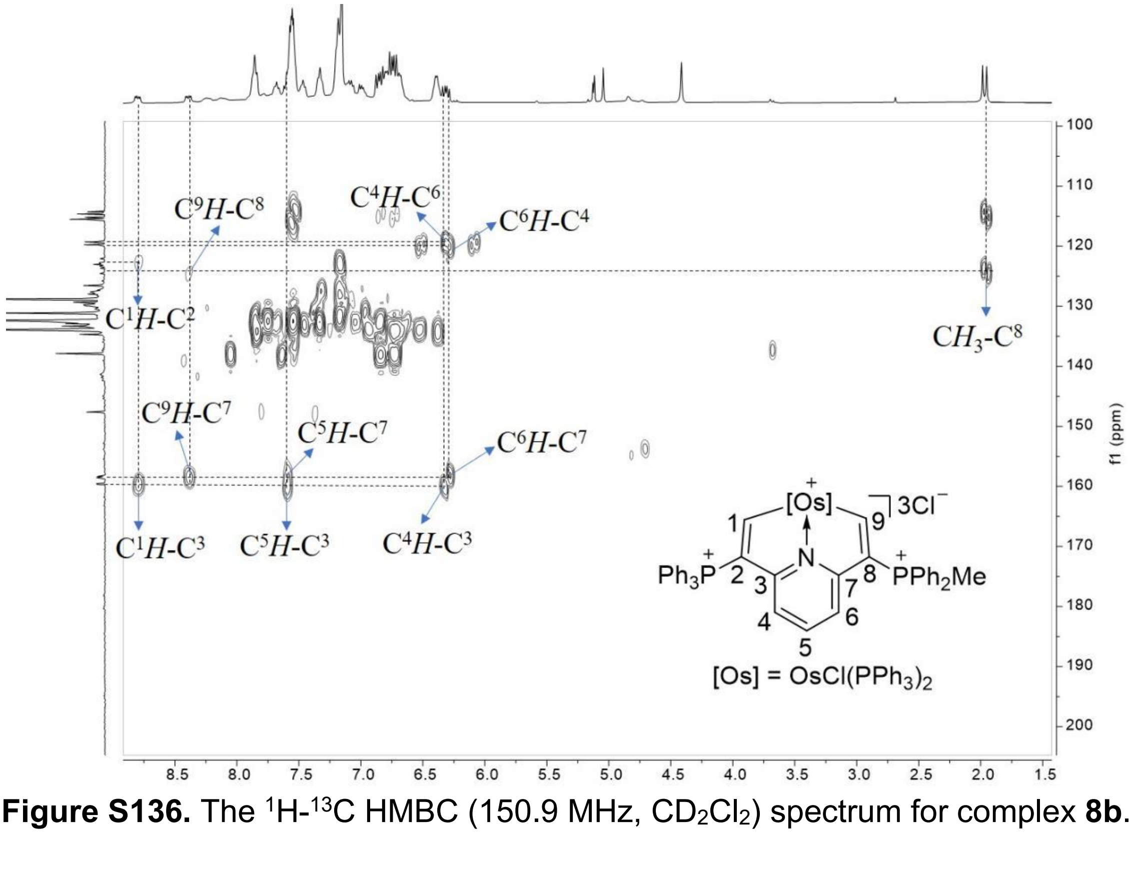

Figure S136. The ^{1}H-^{13}C HMBC (150.9 MHz, CD_2Cl_2) spectrum for complex **8b**.

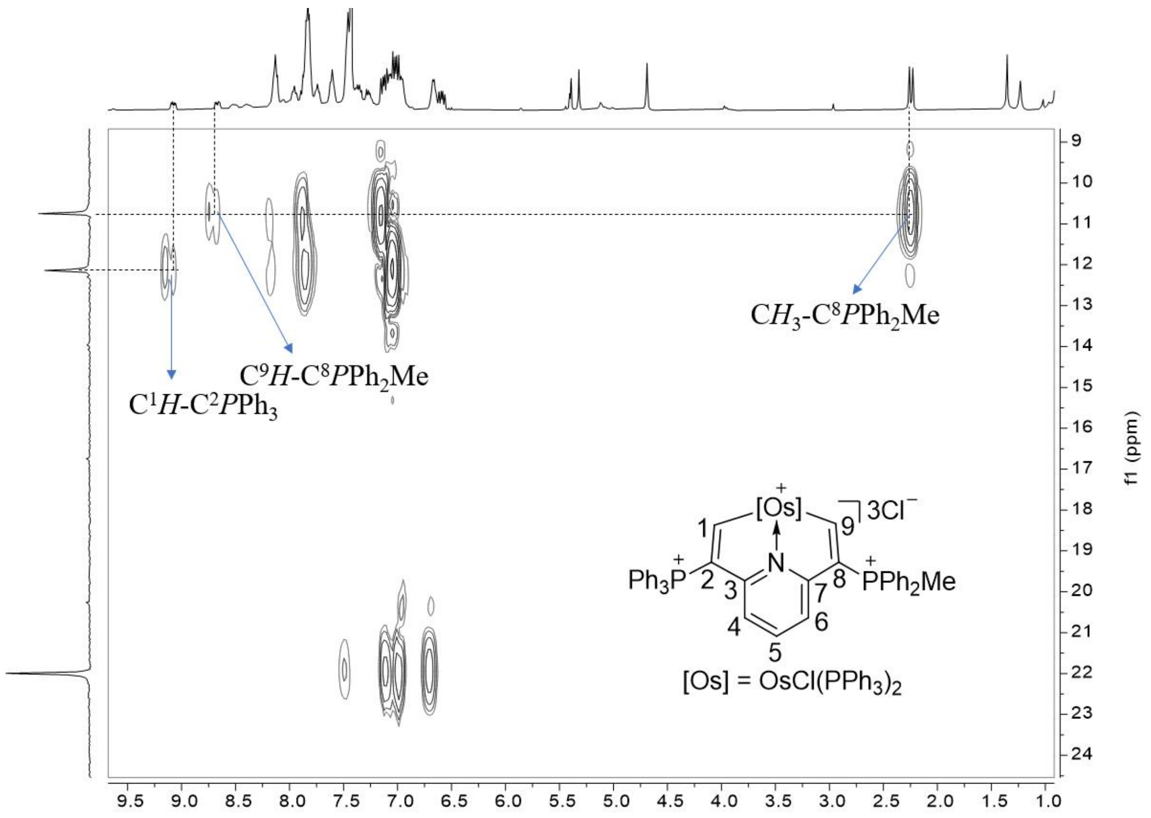

Figure S137. The ^{1}H-^{31}P HMBC (242.9 MHz, CD_2Cl_2) spectrum for complex **8b**.

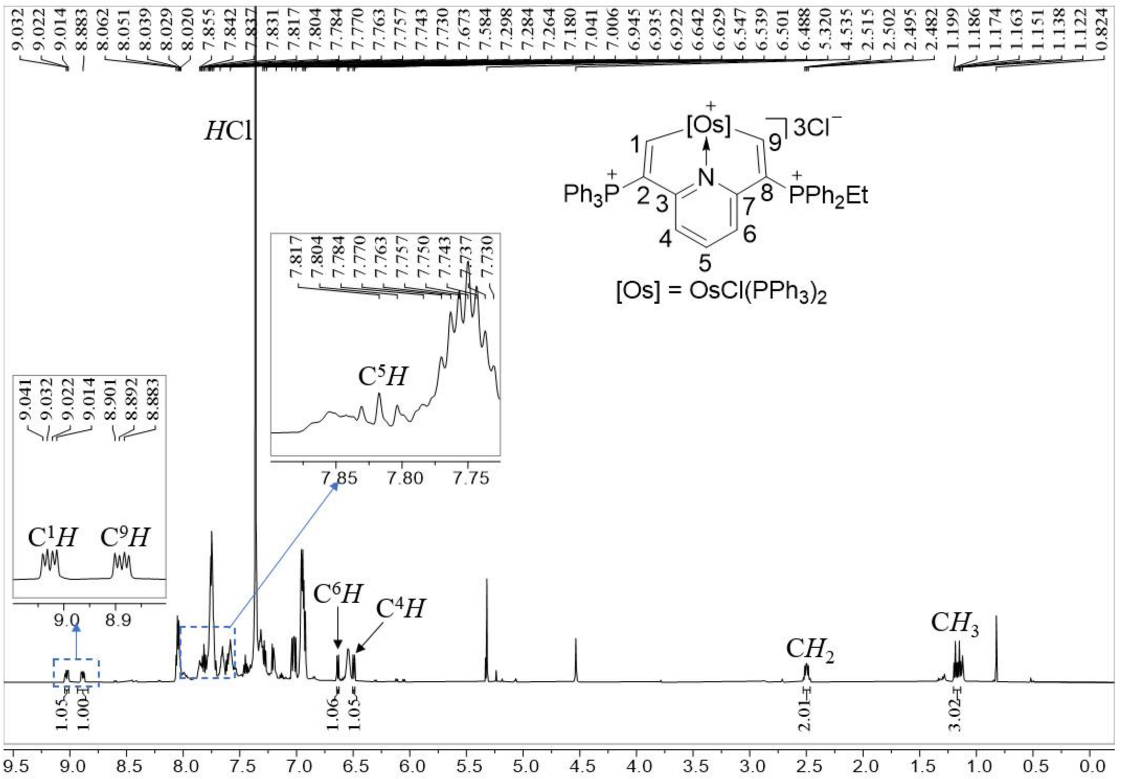

Figure S138. The ^{1}H NMR (600.1 MHz, CD_2Cl_2) spectrum for complex **8c**.

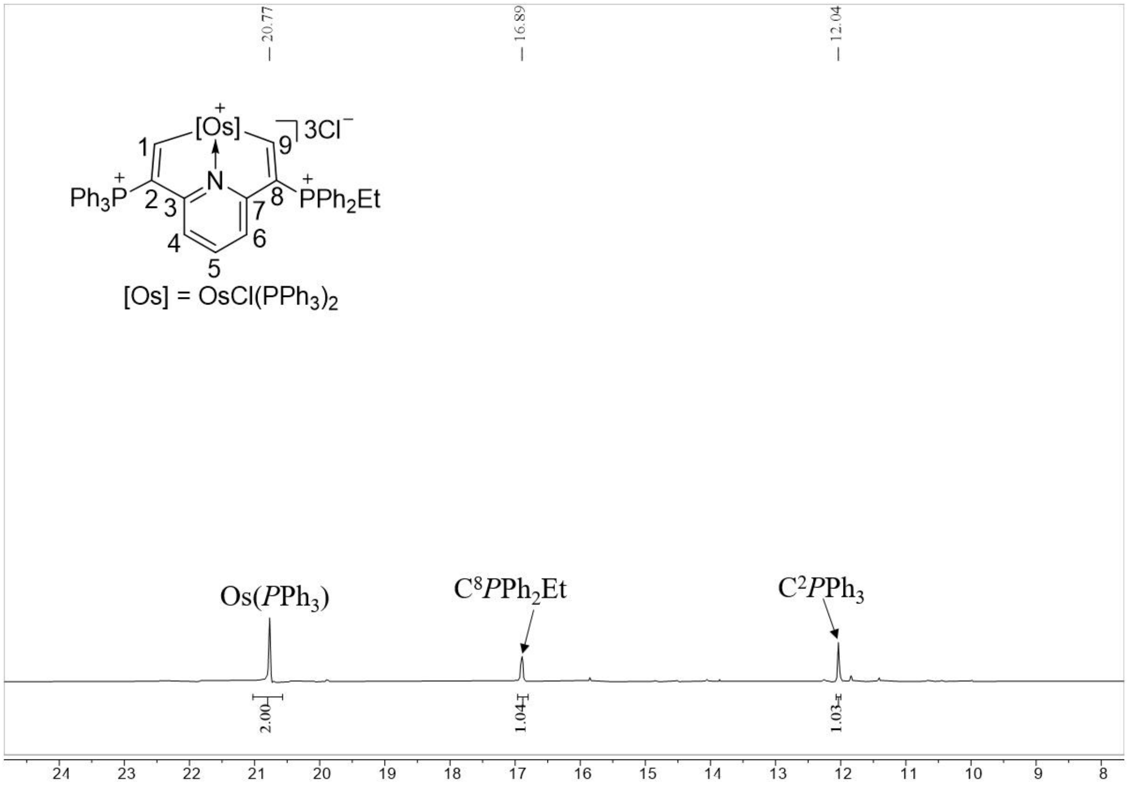

Figure S139. The $^{31}P\{^{1}H\}$ NMR (242.9 MHz, CD_2Cl_2) spectrum for complex **8c**.

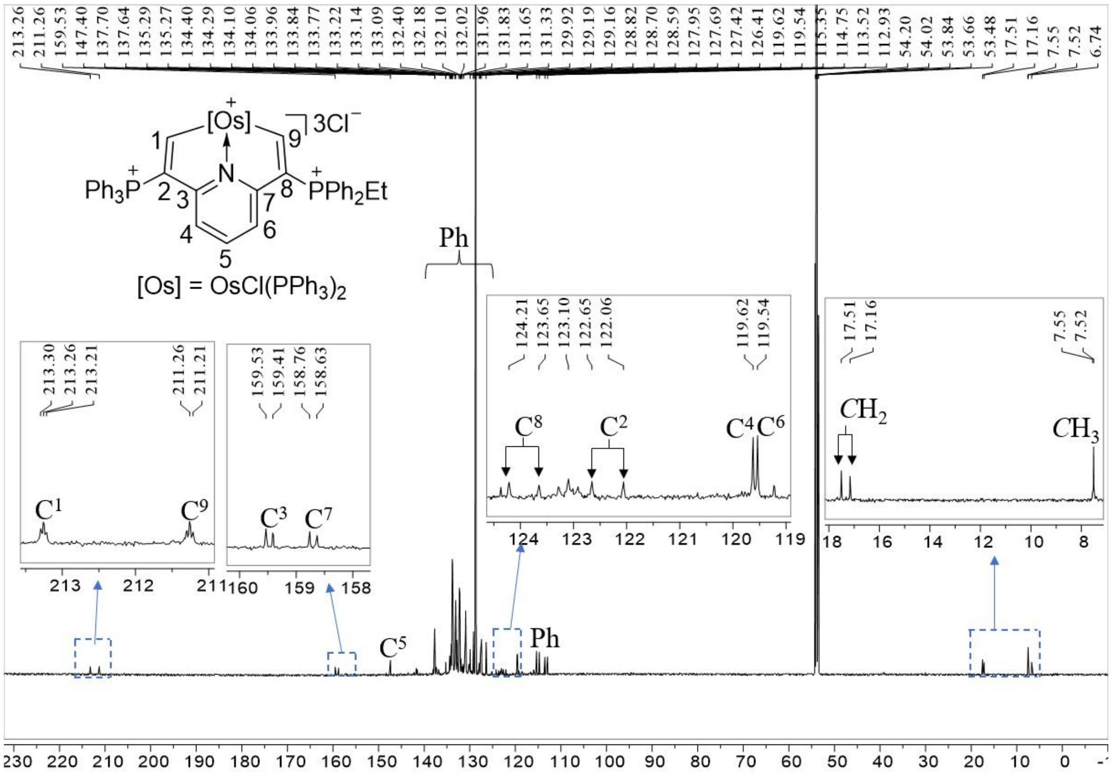

Figure S140. The $^{13}C\{^{1}H\}$ NMR (150.9 MHz, CD_2Cl_2) spectrum for complex **8c**.

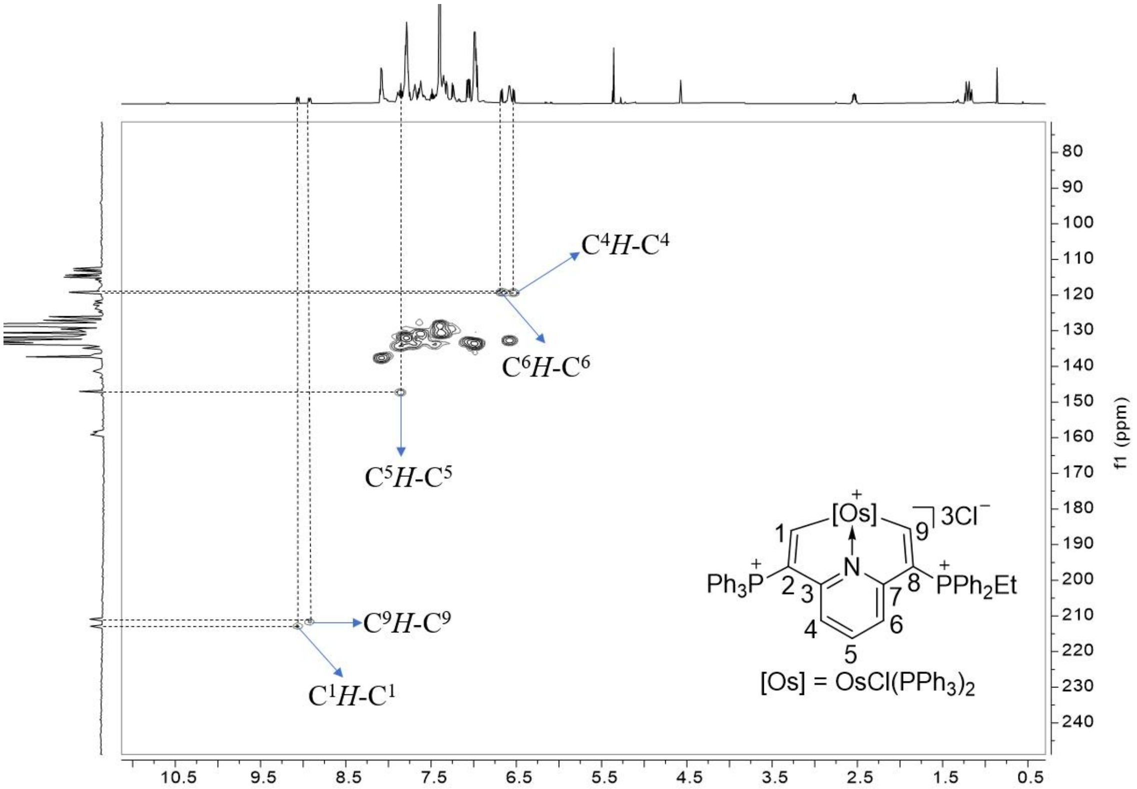

Figure S141. The ^1H-^{13}C HSQC (150.9 MHz, CD_2Cl_2) spectrum for complex **8c**.

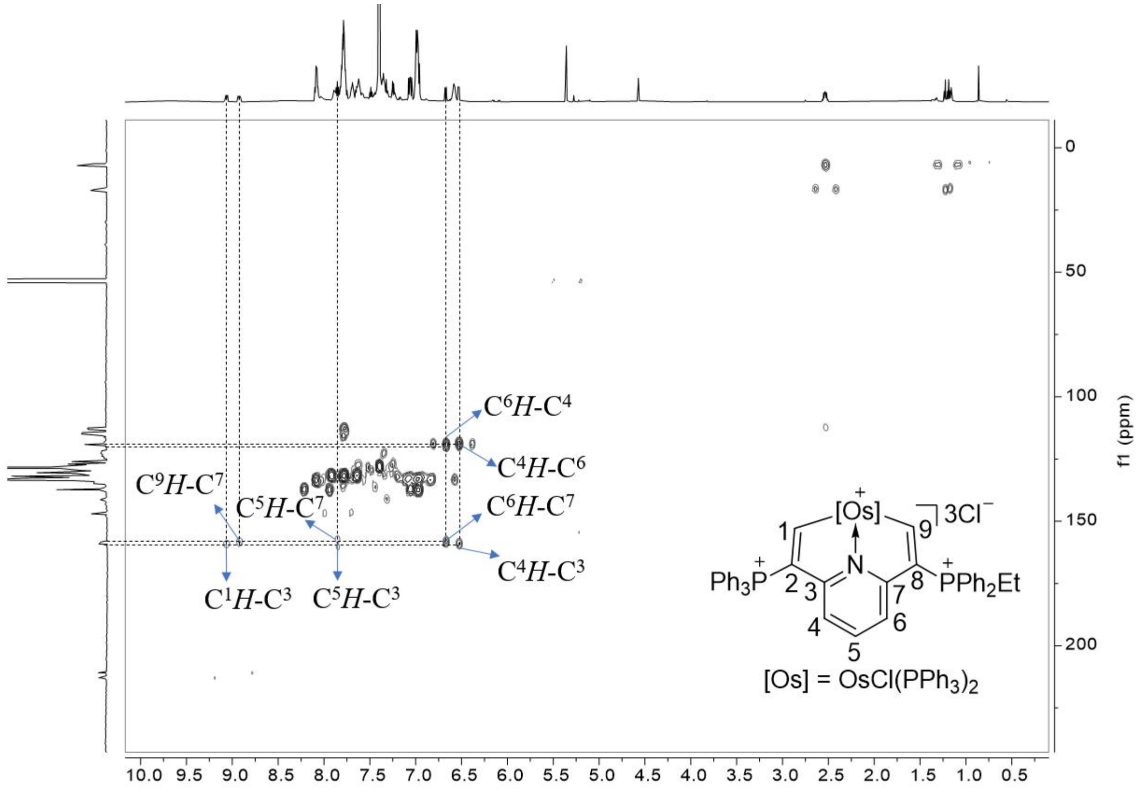

Figure S142. The ^1H-^{13}C HMBC (150.9 MHz, CD_2Cl_2) spectrum for complex **8c**.

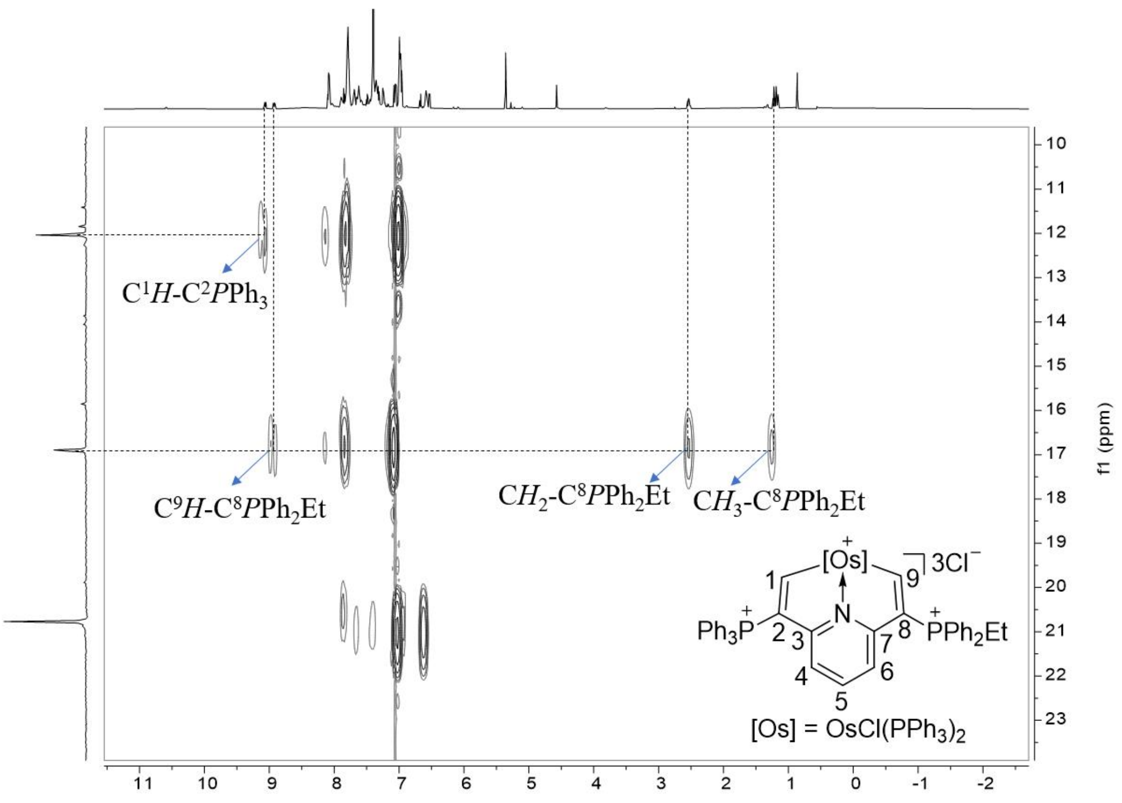

Figure S143. The ^{1}H-^{31}P HMBC (242.9 MHz, CD_2Cl_2) spectrum for complex **8c**.

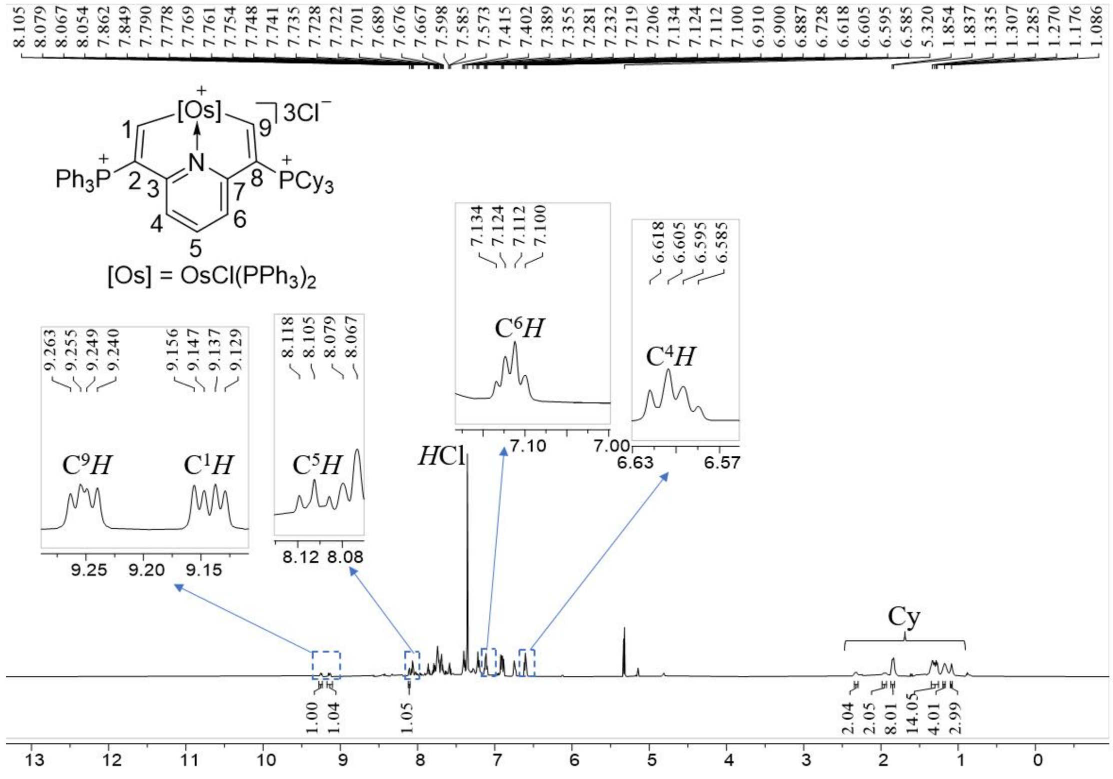

Figure S144. The ^{1}H NMR (600.1 MHz, CD_2Cl_2) spectrum for complex **8d**.

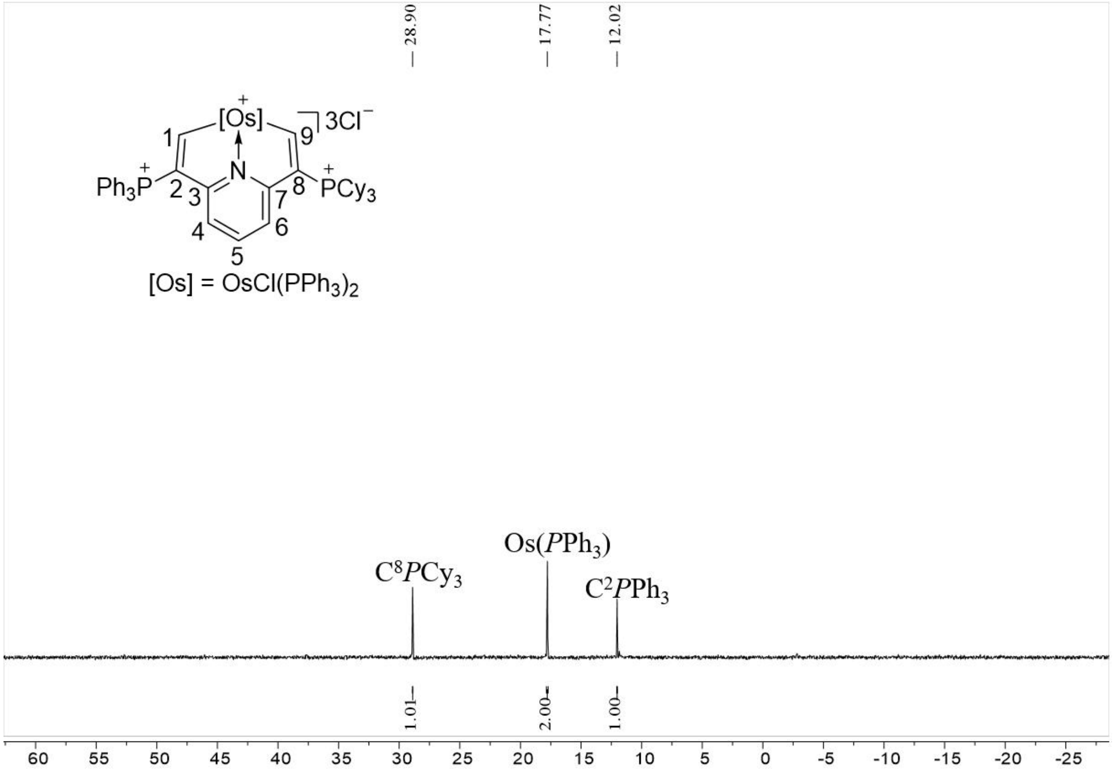

Figure S145. The $^{31}P\{^{1}H\}$ NMR (242.9 MHz, CD_2Cl_2) spectrum for complex **8d**.

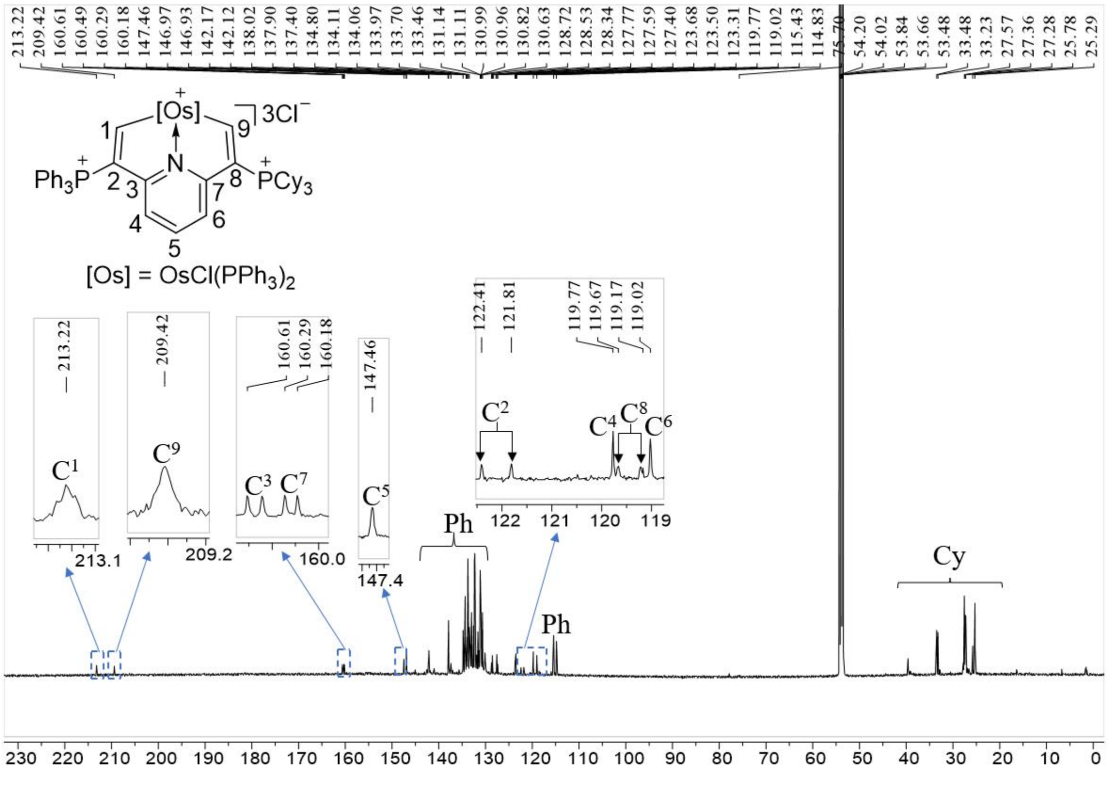

Figure S146. The $^{13}C\{^{1}H\}$ NMR (150.9 MHz, CD_2Cl_2) spectrum for complex **8d**.

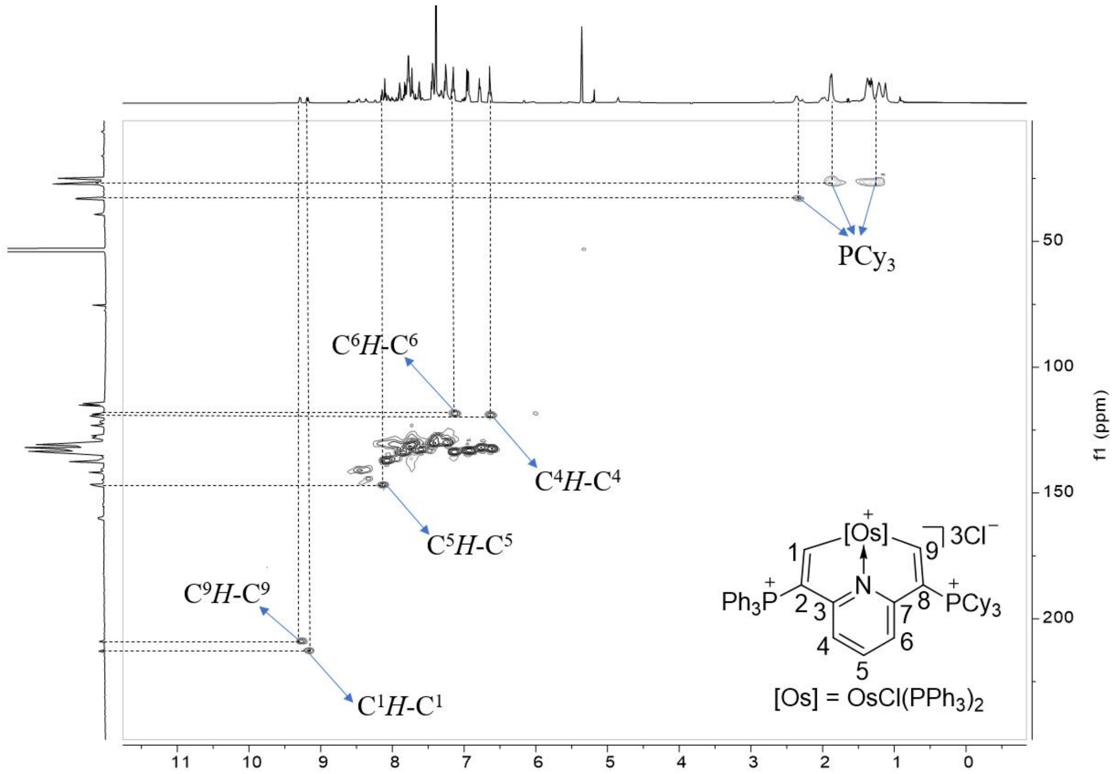

Figure S147. The ^{1}H-^{13}C HSQC (150.9 MHz, CD_2Cl_2) spectrum for complex **8d**.

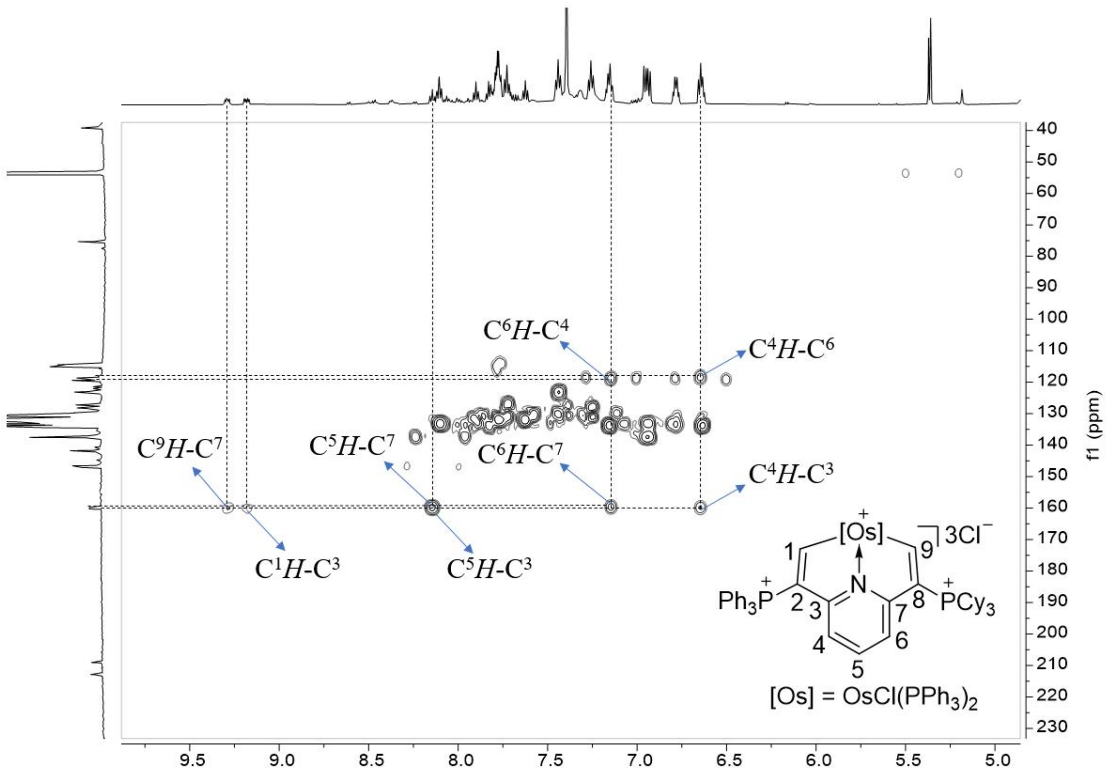

Figure S148. The ^{1}H-^{13}C HMBC (150.9 MHz, CD_2Cl_2) spectrum for complex **8d**.

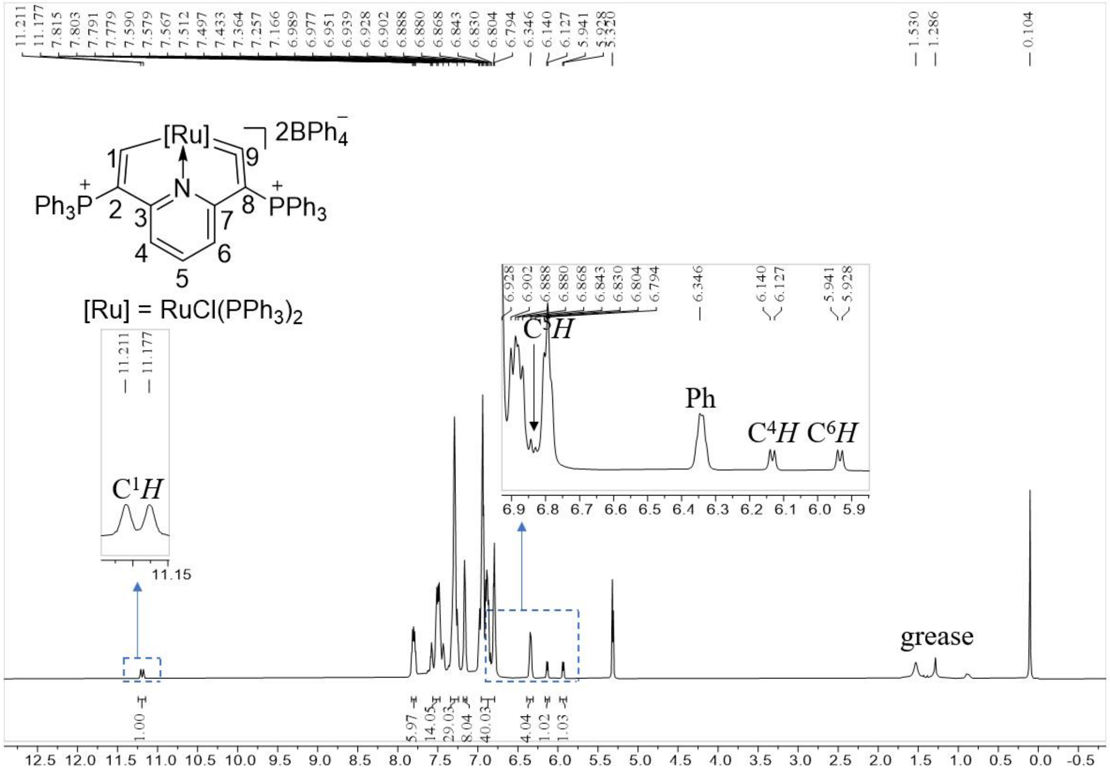

Figure S149. The ^{1}H NMR (600.1 MHz, CD_2Cl_2) spectrum for complex **9**.

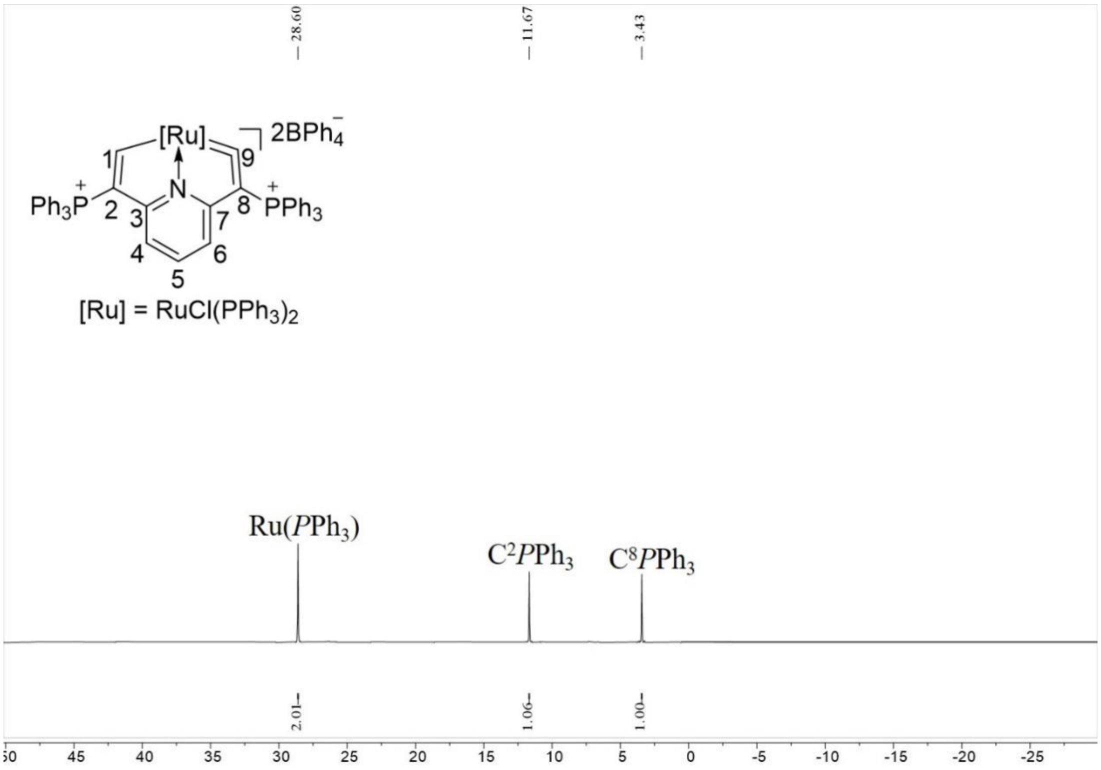

Figure S150. The $^{31}P\{^{1}H\}$ NMR (242.9 MHz, CD_2Cl_2) spectrum for complex **9**.

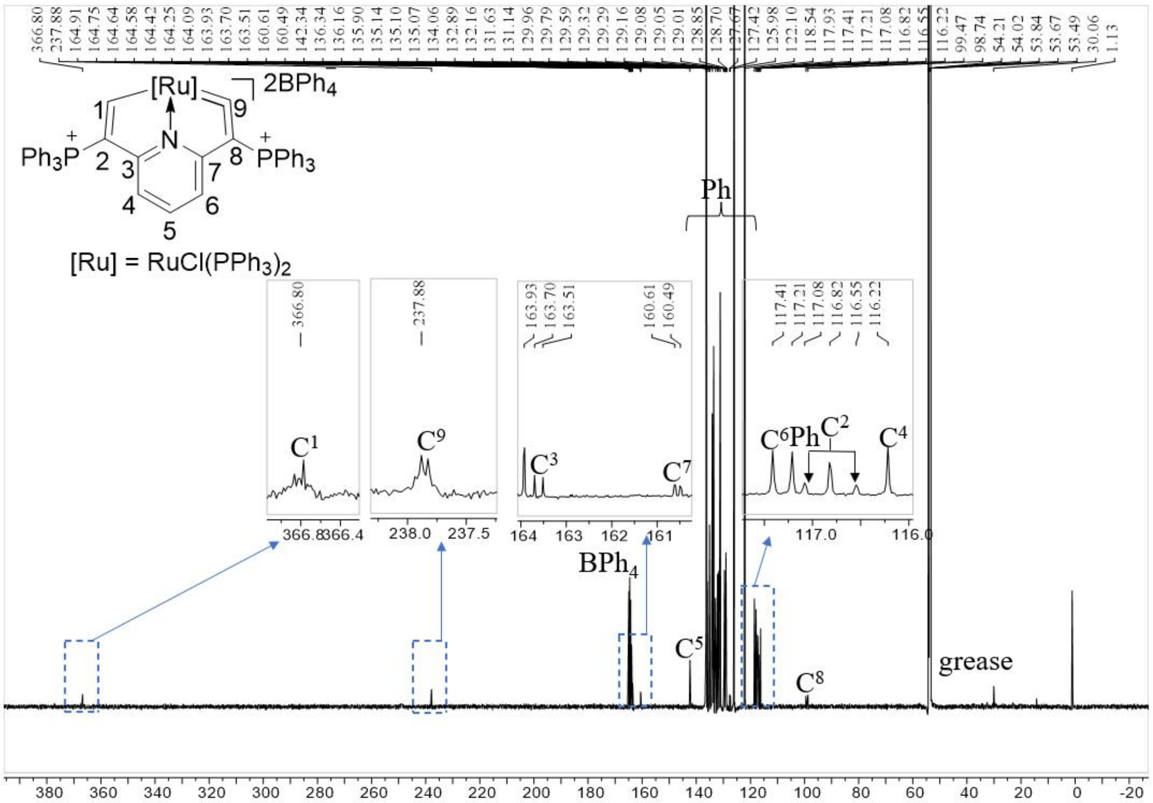

Figure S151. The $^{13}C\{^1H\}$ NMR (150.9 MHz, CD_2Cl_2) spectrum for complex **9**.

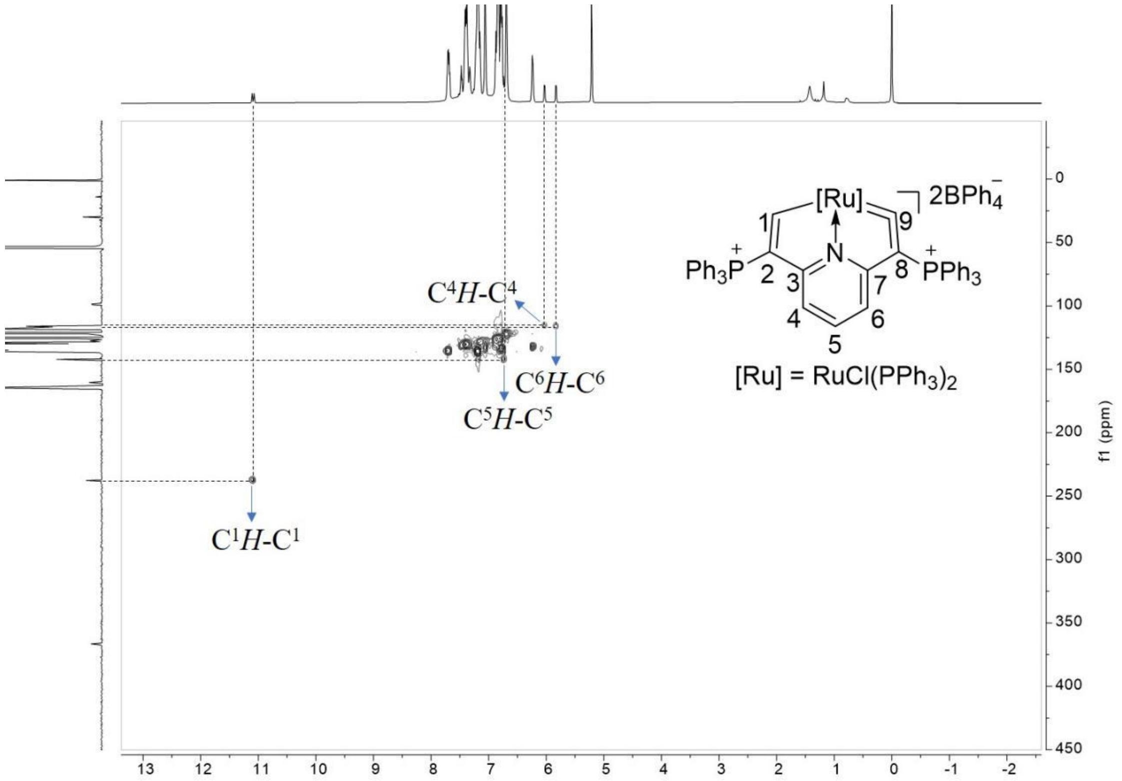

Figure S152. The ^{1}H-^{13}C HSQC (150.9 MHz, CD_2Cl_2) spectrum for complex **9**.

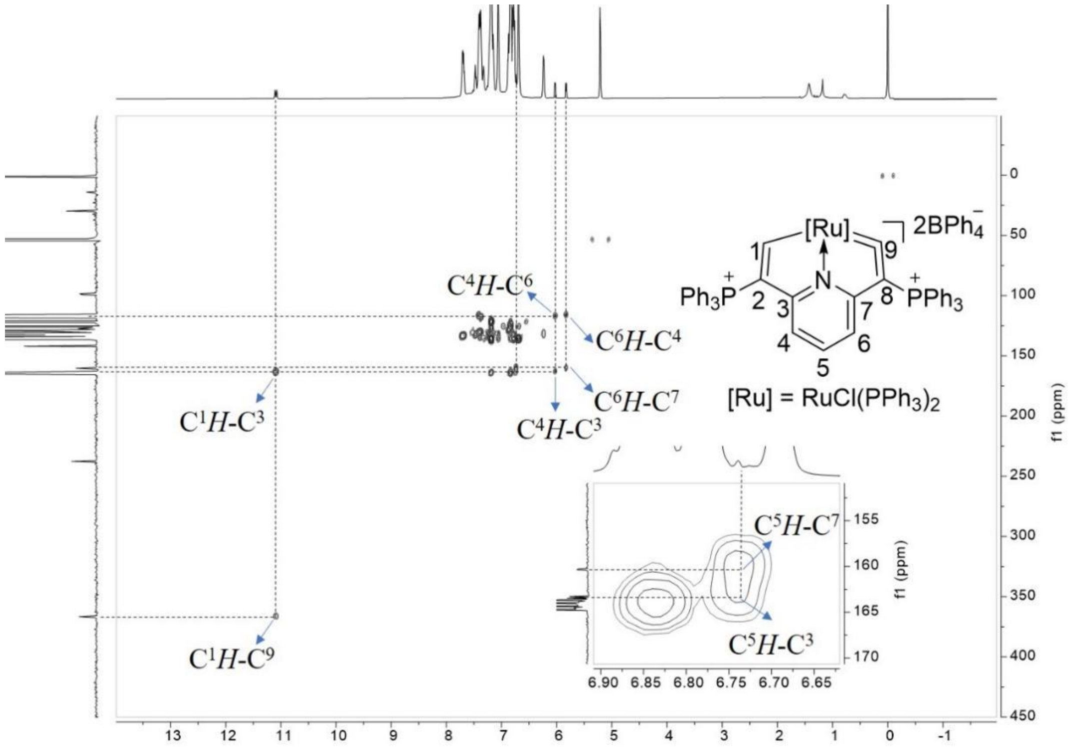

Figure S153. The ^{1}H-^{13}C HMBC (150.9 MHz, CD_2Cl_2) spectrum for complex **9**.

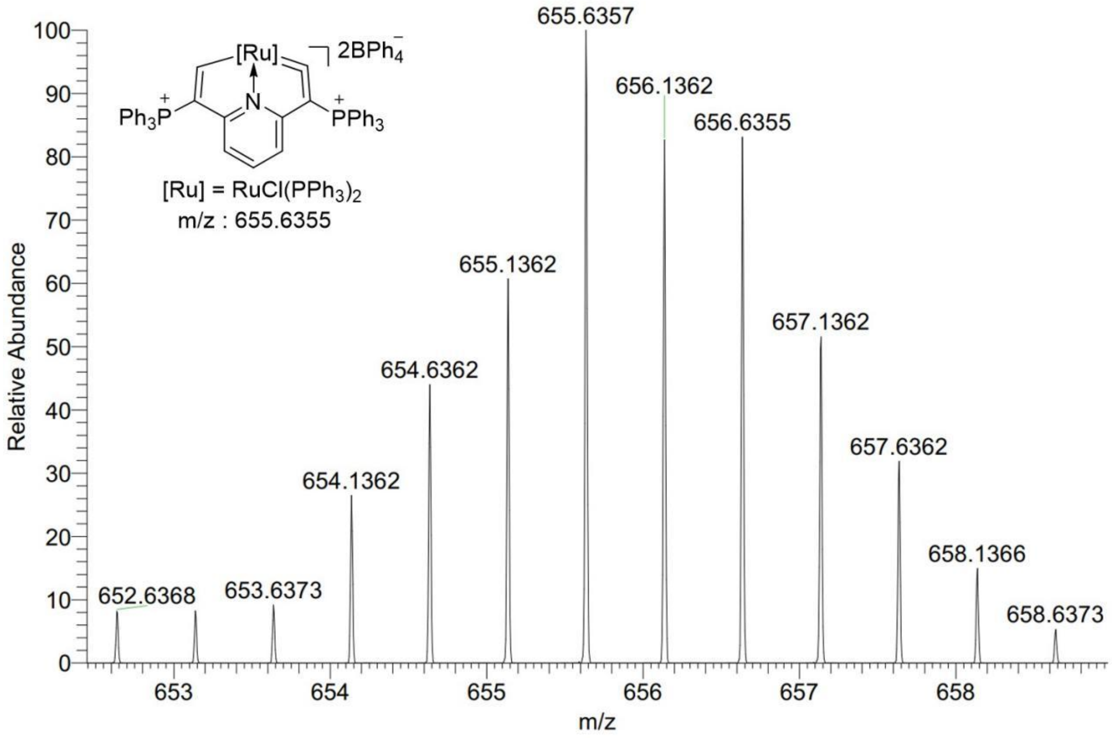

Figure S154. Positive-ion ESI-MS spectrum of [**9**]$^{+}$ measured in methanol

5. Thermal Stability Experiments

A solid sample of **1** could be heated at 120 °C for at least three hours without noticeable decomposition, partly decomposed at 130 °C and completely decomposed at 140 °C. A solid sample of **5a** could be heated at 130 °C for at least three hours without noticeable decomposition, partly decomposed at 140 °C and completely decomposed at 160 °C. A solid sample of **5b** could be heated at 140 °C for at least three hours without noticeable decomposition, partly decomposed at 150 °C and completely decomposed at 170 °C. A solid sample of **5c** could be heated at 150 °C for at least three hours without noticeable decomposition, partly decomposed at 160 °C and completely decomposed at180 °C. A solid sample of **7a** could be heated at 140 °C for at least three hours without noticeable decomposition, partly decomposed at 150 °C and completely decomposed at170 °C. A solid sample of **9** could be heated at 130 °C for at least three hours without noticeable decomposition, partly decomposed at 140 °C and completely decomposed at 150 °C.

Table S2. Thermal decomposition data of complex **1**, **5a**, **5b**, **5c**, **7a**, **9** in solid state.

Temperature (°C)	80	100	120	130	140	150	160	170	180
complex **1**	●	●	●	▲	■	—	—	—	—
complex **5a**	●	●	●	●	▲	▲	■	—	—
complex **5b**	●	●	●	●	●	▲	▲	■	—
complex **5c**	●	●	●	●	●	●	▲	▲	■
complex **7a**	●	●	●	●	●	▲	▲	■	—
complex **9**	●	●	●	●	▲	■	—	—	—

All reactions were performed for 3 h in air. ● = Stable; ▲ = Partly decomposed; ■ = Completely decomposed.

6. Theoretical Calculations

Computational details.

Geometry optimizations were carried out using the Gaussian 16 package[5] with the B3LYP functional[6, 7] augmented with the D3 version of Grimme's empirical dispersion correction.[8-10] the basis set SDD were used to describe the Os atoms[11], whereas the standard 6-31G* basis set was used for the C, N, S, P, Cl and H atoms. Frequency calculations at the same level of theory were performed to identify the number of imaginary frequencies (zero for local minimum and one for transition states) and provide the thermal corrections of Gibbs free energy. Transition states were submitted to intrinsic reaction coordinate calculations to determine two corresponding minima. The single-point energy calculations were performed at the TPSS-D3(BJ)/def2-TZVP level of theory for solution-phase.[12] The gas-phase geometry was used for all the solution phase calculations. The SMD method was used with the dichloromethane solvent[13]. The corrections of Gibbs free energy calculations were added to the single-point energies to obtain the Gibbs free energy in solution. All the energies reported in the paper correspond to the reference state of 1 mol/L, 298K. Natural bond orbital (NBO) calculations were carried out using NBO 7.0 program[14] at the B3LYP-D3/def2-TZVP level of theory.

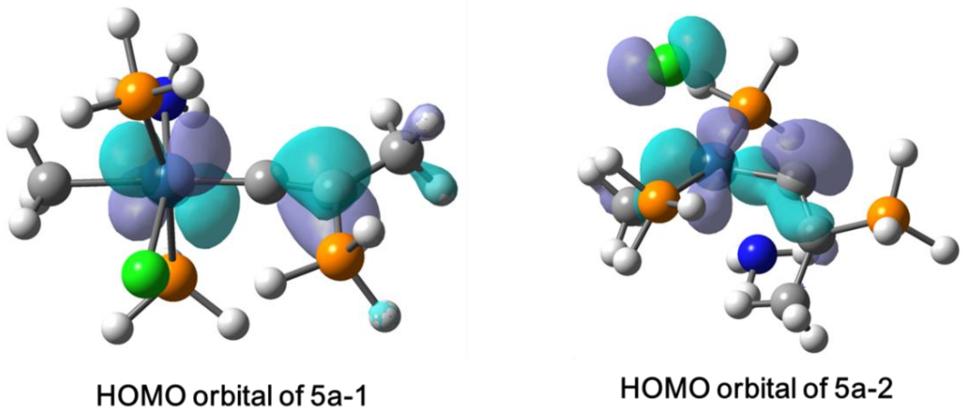

Figure S155. HOMO orbitals of linear (**5a-1**) and bent (**5a-2**) metal vinylidenes.

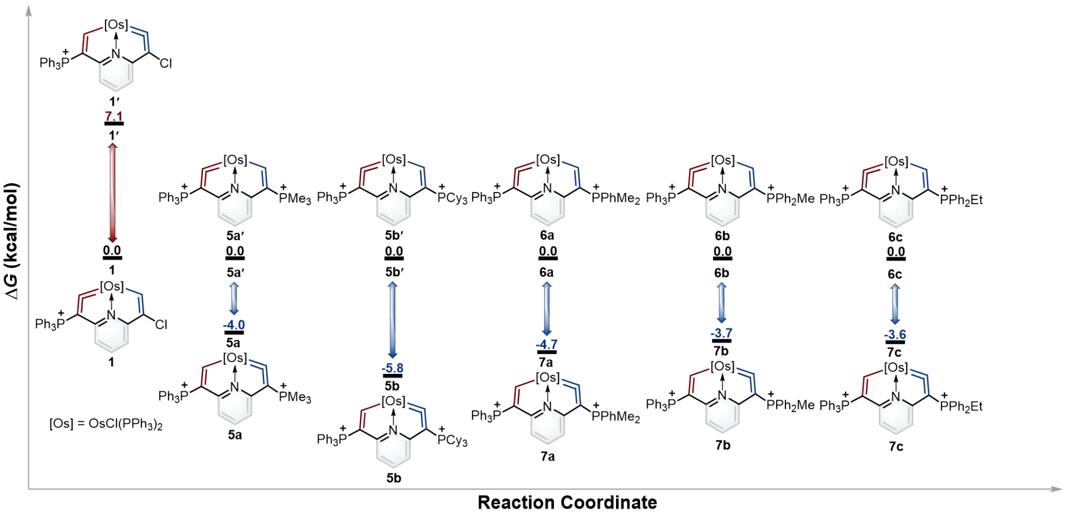

Figure S156. The relative energies between complexes **1'** and **1**, **5a'** and **5a**, **5b'** and **5b**, **6a** and **7a**, **6b** and **7b**, **6c** and **7c** at 298 K. Energies are given in kcal/mol

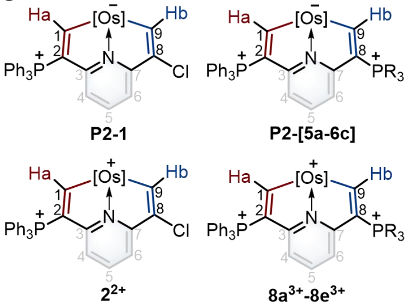

P2-5a: PR_3 = PMe_3
P2-5b: PR_3 = PCy_3
P2-6a: PR_3 = $PPhMe_2$
P2-6b: PR_3 = PPh_2Me
P2-6c: PR_3 = PPh_2Et

$8e^{3+}$: PR_3 = PMe_3
$8d^{3+}$: PR_3 = PCy_3
$8a^{3+}$: PR_3 = $PPhMe_2$
$8b^{3+}$: PR_3 = PPh_2Me
$8c^{3+}$: PR_3 = PPh_2Et

NBO Charges	Ha	Hb	NBO Charges	Ha	Hb
**P2-1**	**0.170**	0.186	**2^{2+}**	**0.246**	0.247
**P2-5a**	0.181	**0.155**	**$8e^{3+}$**	0.279	**0.246**
**P2-5b**	0.178	**0.160**	**$8d^{3+}$**	0.277	**0.259**
**P2-6a**	0.181	**0.165**	**$8a^{3+}$**	0.257	**0.243**
**P2-6b**	0.180	**0.165**	**$8b^{3+}$**	0.258	**0.248**
**P2-6c**	0.181	**0.154**	**$8c^{3+}$**	0.258	**0.226**

Figure S157. The calculated NBO charges of intermediates **P2-1**, **P2-5a-6c**, **2^{2+}**, and **$8a^{3+}$-$8e^{3+}$**.

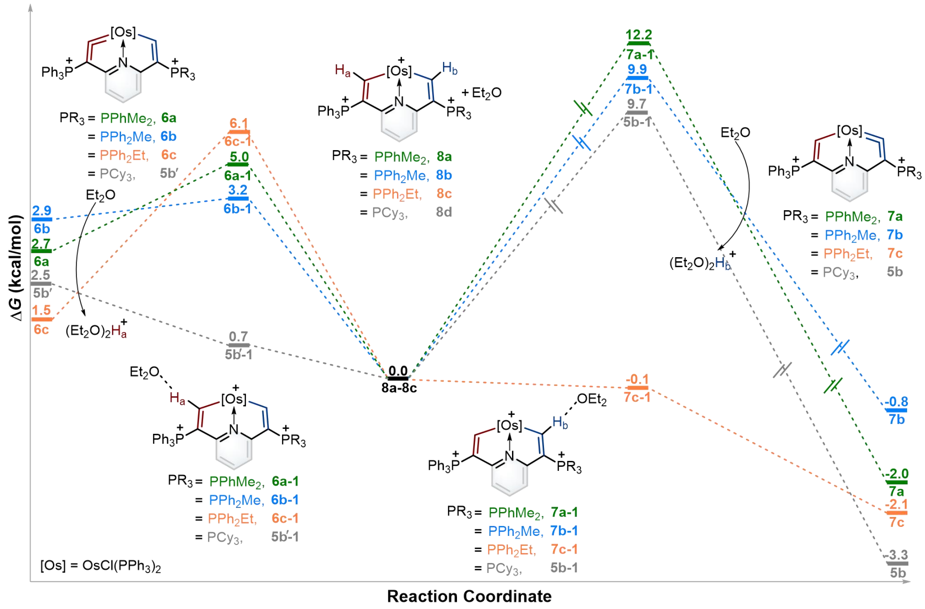

Figure S158. DFT-calculated the Gibbs energies for the formation of 7 and 5b at 298 K. Energies are given in kcal/mol.

7. Cartesian Coordinates

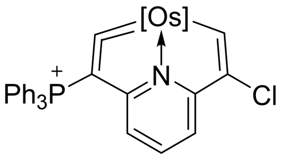

1

[Os] = $OsCl(PPh_3)_2$

E = -4521.992235 a.u.

Os	1.17270800	0.25081000	-0.49993200
Cl	3.76987100	1.23006100	3.29264300
Cl	1.99116900	0.41327300	-2.75685500
P	0.54816800	2.58462600	-0.54607700
P	1.99940800	-2.01308200	-0.35969100
P	-3.21376100	-0.79344400	0.12634900
N	0.44299500	0.09356200	1.49925400
C	-0.59355200	-0.28452000	-0.63266800
C	-1.49397500	-0.51477300	0.37721100
C	-0.84684400	-0.30797500	1.67846400
C	-1.35444000	-0.46043300	2.97031500
H	-2.36893400	-0.80503600	3.12129800
C	-0.53081900	-0.15944700	4.05539000
H	-0.91455100	-0.26546900	5.06556700
C	0.77740600	0.27102400	3.85519500
H	1.43329600	0.51022500	4.68169700
C	1.25039700	0.38298200	2.54719100
C	2.56688500	0.79277900	2.08744900
C	2.77352300	0.81193900	0.75995400
H	3.74580600	1.11971900	0.37995200
C	1.78284500	3.78933900	-1.16058500
C	1.44550000	5.15114500	-1.21977600
H	0.44985600	5.48016400	-0.94354700
C	2.38580800	6.09210300	-1.62992700
H	2.11368000	7.14247400	-1.67190700
C	3.67434200	5.68512300	-1.98601400
H	4.40687600	6.41978600	-2.30666900
C	4.01456800	4.33437700	-1.93333900
H	5.01089600	4.01027900	-2.21859600
C	3.07381100	3.38809100	-1.52356900
H	3.33589500	2.33995500	-1.51522600
C	0.19268000	3.17032100	1.14959700
C	1.20156300	3.76422000	1.91908600
H	2.16664600	3.98138700	1.47482200
C	0.97012200	4.07254300	3.26013000
H	1.76087500	4.52776900	3.84832500
C	-0.26702800	3.79351000	3.84259000
H	-0.44337700	4.03658800	4.88629400
C	-1.27622400	3.20074700	3.08033700
H	-2.24252100	2.97560300	3.52207200
C	-1.04456800	2.88486800	1.74431700
H	-1.82708600	2.41334500	1.16452300
C	-0.99204900	2.94297100	-1.47868300
C	-1.82371700	4.02289100	-1.14281100
H	-1.58496900	4.64948700	-0.2903600
C	-2.98059500	4.27767700	-1.87969100
H	-3.61417300	5.11928600	-1.61384600
C	-3.32251100	3.45209200	-2.95375100
H	-4.22796100	3.64486400	-3.52174900
C	-2.49479200	2.38196900	-3.29730900
H	-2.75556200	1.73486800	-4.12771700
C	-1.33273200	2.12840600	-2.56821400
H	-0.69070800	1.29850800	-2.83883700
C	3.66789300	-2.35399200	-1.02811600
C	4.55443800	-1.31673900	-1.33945300
H	4.23112100	-0.28869100	-1.26183000
C	5.84635500	-1.60711900	-1.77999800
H	6.52477000	-0.79545100	-2.02483300
C	6.25926000	-2.93201600	-1.91658500
H	7.26366200	-3.15614700	-2.26311300
C	5.37651100	-3.97178900	-1.61313800
H	5.69183800	-5.00507700	-1.72251100
C	4.08728000	-3.68592300	-1.17154700
H	3.40671800	-4.49907600	-0.94066000
C	2.12527800	-2.55781100	1.38357700
C	0.98591000	-2.94129000	2.10665300
H	0.02447300	-3.00618600	1.61333400
C	1.07365600	-3.22610200	3.46677900
H	0.18200400	-3.52278500	4.01100900
C	2.29806400	-3.11339700	4.12830100
H	2.36560300	-3.32848700	5.19052800
C	3.43349000	-2.71920200	3.42035900
H	4.38768100	-2.62023200	3.92833100
C	3.35027800	-2.44627500	2.05483800
H	4.23811800	-2.13865100	1.51371100
C	0.94120700	-3.25618700	-1.19608300
C	0.45482900	-2.93512900	-2.47359700
H	0.68580300	-1.96615100	-2.90609900
C	-0.30850800	-3.86104500	-3.18360600
H	-0.66814200	-3.61140300	-4.17769000
C	-0.60899000	-5.10585500	-2.62519200
H	-1.21132900	-5.82037900	-3.17806400
C	-0.11743200	-5.43371900	-1.36163800
H	-0.32801500	-6.40832900	-0.93050500
C	0.66437300	-4.51852800	-0.65265000
H	1.05666100	-4.79071500	0.32081600
C	-3.67439700	-2.43707900	0.72589700
C	-4.99778300	-2.77041600	1.05223100
H	-5.77869200	-2.01717900	1.02426300
C	-5.31005900	-4.08085200	1.40968400
H	-6.33152800	-4.33856800	1.67128000
C	-4.31408600	-5.06141800	1.42255200
H	-4.56430000	-6.08091300	1.69957500
C	-3.00376600	-4.73665100	1.06608300
H	-2.23168800	-5.49916600	1.05131000
C	-2.68118900	-3.42727800	0.71660400
H	-1.67083900	-3.17800600	0.41143400
C	-4.15184300	0.49342800	0.98555700
C	-4.14969700	1.77777800	0.41421400
H	-3.69341200	1.94963500	-0.55460300
C	-4.72402200	2.84291800	1.10200600
H	-4.71407000	3.83101600	0.65397300
C	-5.29438600	2.63954100	2.36158200
H	-5.73938800	3.47286600	2.89658700
C	-5.29122900	1.36633900	2.93413400
H	-5.73249700	1.20671100	3.91293400
C	-4.71886000	0.29240900	2.25153700
H	-4.71678300	-0.69279200	2.70467000
C	-3.60207500	-0.71574600	-1.63669200
C	-4.81953900	-0.17179900	-2.07005800
H	-5.51014800	0.26929100	-1.36009500
C	-5.13945400	-0.18580300	-3.42681200

H	-6.08017800	0.24068700	-3.76054200
C	-4.25367600	-0.74412900	-4.35032800
H	-4.50484900	-0.75124400	-5.40666000
C	-3.04900200	-1.30125400	-3.91509300
H	-2.36109000	-1.74296800	-4.62901000
C	-2.72334800	-1.29928500	-2.56084200
H	-1.79363900	-1.74540100	-2.23294200

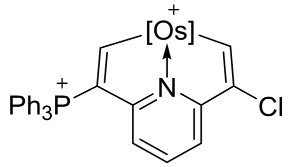

2

[Os] = $OsCl(PPh_3)_2$

E= -4522.439699 a.u.

Os	-1.28885000	0.03013400	-0.41500400
Cl	-3.79539200	-0.14480200	3.49980300
Cl	-2.13083800	0.01651000	-2.57255600
P	-1.33550500	-2.42260400	-0.46889900
P	-1.37016300	2.46453500	-0.31634400
P	3.36708400	0.05768300	0.04350400
N	-0.37169200	-0.14136600	1.55374500
C	0.68451100	0.19480200	-0.61656900
C	1.60251200	0.06146500	0.37081700
C	0.98065900	-0.17166700	1.67066700
C	1.58179900	-0.38686500	2.91178400
H	2.65983100	-0.45419200	2.98934200
C	0.77498500	-0.51800100	4.04198400
H	1.22803900	-0.68366500	5.01388300
C	-0.60763600	-0.42499400	3.92403000
H	-1.26336700	-0.49840700	4.78218100
C	-1.15606900	-0.24369000	2.65623900
C	-2.55127000	-0.11071600	2.29284900
C	-2.75864000	0.07767000	0.98344700
H	-3.72995000	0.30449600	0.54261200
C	-2.85316100	-3.07491400	-1.24200700
C	-2.78908400	-4.08571200	-2.21060900
H	-1.83281700	-4.49371400	-2.51625300
C	-3.96111000	-4.57036100	-2.79274500
H	-3.90183700	-5.35149300	-3.54405400
C	-5.20081500	-4.05622100	-2.41340000
H	-6.10988100	-4.43611900	-2.86868300
C	-5.27085000	-3.04851100	-1.44825200
H	-6.23316800	-2.64470900	-1.14926400
C	-4.10382000	-2.55364700	-0.87173900
H	-4.17073900	-1.76708800	-0.12846400
C	-1.23583300	-3.14556200	1.20651900
C	-2.39900600	-3.45714100	1.92315100
H	-3.37231600	-3.36017700	1.45684900
C	-2.31151300	-3.91290100	3.23964100
H	-3.21958300	-4.15431100	3.78284600
C	-1.06620800	-4.06984800	3.84834400
H	-1.00153300	-4.43536900	4.86850300
C	0.09691300	-3.76452100	3.13691800
H	1.07140800	-3.88854400	3.59985000
C	0.01388700	-3.29688100	1.82876700
H	0.92389700	-3.05736600	1.29315900
C	0.04685800	-3.17764000	-1.39900500
C	0.51064700	-4.46440800	-1.07699000
H	0.07412100	-5.00836700	-0.24689100
C	1.53203000	-5.05005500	-1.82546800
H	1.87803700	-6.04801300	-1.57355900
C	2.09434000	-4.36431800	-2.90513200
H	2.88588700	-4.82525200	-3.48797600
C	1.61812100	-3.09747000	-3.24724100
H	2.03294600	-2.56797100	-4.09812800
C	0.59855600	-2.50722200	-2.50048700
H	0.21211500	-1.53949800	-2.79778100
C	-2.94478400	3.26502400	-0.76858400
C	-4.07063400	2.54084600	-1.18035400
H	-4.02430900	1.46526200	-1.29315200
C	-5.25618300	3.20861900	-1.49241200
H	-6.12258000	2.64120100	-1.81743800
C	-5.32328200	4.59812900	-1.39675700
H	-6.24572900	5.11545900	-1.64092700
C	-4.20003100	5.32676900	-0.99391000
H	-4.24792200	6.40890300	-0.92462800
C	-3.01494000	4.66642000	-0.68364200
H	-2.14560900	5.23845200	-0.37361500
C	-1.07350400	2.96404900	1.41271700
C	0.21945000	2.93882900	1.95768500
H	1.07199000	2.74755600	1.31718800
C	0.41269300	3.16320000	3.31871500
H	1.41749900	3.16110000	3.73089600
C	-0.68137600	3.39605200	4.15558000
H	-0.52895500	3.57040400	5.21610900
C	-1.97002700	3.41522200	3.62148500
H	-2.82508200	3.59919400	4.26404800
C	-2.16752300	3.20331700	2.25652500
H	-3.17291800	3.22414300	1.85078000
C	-0.12518800	3.30603100	-1.36443100
C	-0.08689200	2.95799900	-2.72533100
H	-0.74798100	2.18807800	-3.10838300
C	0.77237200	3.62468400	-3.59634900
H	0.77149000	3.36568900	-4.65084800
C	1.61791900	4.62974500	-3.11850700
H	2.28394900	5.15236000	-3.79844400
C	1.58576100	4.97734300	-1.76725800
H	2.22582100	5.77035100	-1.39284000
C	0.70848700	4.33036400	-0.89352600
H	0.66704200	4.64131800	0.14337700
C	4.21024200	1.47483000	0.77230100
C	5.41524600	1.90329800	0.18896200
H	5.80254800	1.41119400	-0.69724600
C	6.10945700	2.97501400	0.74682100
H	7.03982300	3.30534200	0.29644900
C	5.60550400	3.62570300	1.87570900
H	6.14672700	4.46360700	2.30354000
C	4.40715600	3.20155100	2.45457300
H	4.01660800	3.70962100	3.33071100
C	3.70910500	2.12572200	1.91012000
H	2.78539000	1.79862000	2.36903300
C	4.01769000	-1.48098700	0.71457200
C	3.37782800	-2.68255700	0.36232100
H	2.53408000	-2.67895600	-0.32019400
C	3.84145500	-3.88503000	0.88799500
H	3.35078400	-4.81245000	0.61199800
C	4.93222200	-3.89251000	1.76394400
H	5.28913600	-4.83189300	2.17436200
C	5.56805900	-2.69824800	2.10944700
H	6.41726800	-2.70845900	2.78495000
C	5.11578900	-1.48633800	1.58543600
H	5.60899700	-0.55767700	1.85321100

C	3.61759500	0.13569900	-1.73811500
C	4.19588000	-0.93791800	-2.42876100
H	4.48378500	-1.84009000	-1.90211000
C	4.40752600	-0.83686200	-3.80397800
H	4.86741800	-1.66210900	-4.33825700
C	4.03807000	0.32290500	-4.48654600
H	4.20837200	0.39911000	-5.55590200
C	3.46236900	1.39393600	-3.79563200
H	3.18403900	2.29932800	-4.32232000
C	3.25548100	1.31209900	-2.42143600
H	2.83244800	2.15962800	-1.89000000
H	0.94511200	0.36986700	-1.66090600

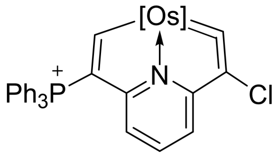

1′

[Os] = $OsCl(PPh_3)_2$

E = -4521.978145 a.u.

Os	1.29756800	0.03526100	0.18927500
Cl	2.25615200	0.01172400	2.39532700
P	1.33609100	2.44574600	0.35733700
P	1.48159800	-2.36697000	0.34478500
N	0.27297200	0.04847300	-1.67111900
C	2.63404700	0.11513200	-1.15170600
C	2.42894500	0.14657300	-2.48765800
C	1.01965000	0.10296900	-2.81414100
C	0.39958700	0.09845900	-4.06918200
H	1.00953600	0.13914800	-4.96341900
C	-0.98753500	0.03298800	-4.12295600
H	-1.49144000	0.01758100	-5.08462300
C	-1.74308500	-0.01552800	-2.94770200
H	-2.82292300	-0.08186800	-2.99038900
C	-1.08302600	0.00553300	-1.71596700
C	-1.63864400	-0.03549400	-0.36281300
C	-0.69862600	-0.05324400	0.63660400
H	-1.02856100	-0.14204000	1.66932500
C	0.78832500	3.18142400	1.94660600
C	1.11777300	4.51355200	2.23938500
H	1.74910800	5.07763100	1.56117000
C	0.64319700	5.11830400	3.40221900
H	0.90774200	6.14902600	3.61825500
C	-0.16031500	4.39930100	4.28954600
H	-0.52224200	4.86940200	5.19913200
C	-0.48028600	3.07023900	4.01096700
H	-1.07626000	2.49301000	4.71229800
C	-0.00713300	2.46313700	2.84759300
H	-0.22354700	1.42193500	2.66304500
C	0.27502500	3.29195200	-0.87445700
C	-0.84467200	4.04325800	-0.49844100
H	-1.10604900	4.14271800	0.54888600
C	-1.60959600	4.69617000	-1.46691000
H	-2.45993700	5.29604400	-1.15860500
C	-1.28435100	4.57885600	-2.81796000
H	-1.87764300	5.09154500	-3.57002100
C	-0.17769800	3.81546700	-3.20129600
H	0.08892300	3.72509700	-4.24990900
C	0.60533700	3.18788100	-2.23602300
H	1.49234800	2.64177500	-2.53417600
C	2.99216000	3.17390400	0.07094900
C	3.15588900	4.39013100	-0.60731200
H	2.29553600	4.91037400	-1.01385000
C	4.43029500	4.93345000	-0.77539800
H	4.54786300	5.87191400	-1.30906300
C	5.54645800	4.27227500	-0.26269400
H	6.53785700	4.69463800	-0.39685300
C	5.38599400	3.06609000	0.42266800
H	6.25158100	2.54891900	0.82582200
C	4.11730400	2.51455500	0.58847300
H	3.99577800	1.57999900	1.12319000
C	0.89894400	-2.99233100	1.96222400
C	-0.45593500	-2.87931900	2.31191500
H	-1.17053600	-2.47474300	1.60248700
C	-0.89692400	-3.29516900	3.56561200
H	-1.94724100	-3.20442600	3.82107000
C	0.01139300	-3.81730700	4.48982100
H	-0.33149000	-4.13583800	5.46981800
C	1.35880400	-3.92775900	4.14997100
H	2.07098500	-4.32903600	4.86456700
C	1.80274800	-3.51944100	2.89146100
H	2.85321600	-3.60060100	2.63819200
C	0.56578700	-3.35664600	-0.89949900
C	0.81755500	-3.08595100	-2.25378300
H	1.51919400	-2.30679300	-2.52317800
C	0.20373500	-3.83789800	-3.25184900
H	0.40412300	-3.61077500	-4.29445200
C	-0.64611500	-4.89312800	-2.91052600
H	-1.10918100	-5.49279900	-3.68882000
C	-0.87900200	-5.18736700	-1.56649400
H	-1.51546600	-6.02420200	-1.29229500
C	-0.28460400	-4.41827500	-0.56243400
H	-0.46465900	-4.66296100	0.47791800
C	3.18404100	-3.01672600	0.17802800
C	4.29051000	-2.19633400	0.43572900
H	4.14175300	-1.15861300	0.70339100
C	5.58072300	-2.71991600	0.34943100
H	6.43327900	-2.07714700	0.54705700
C	5.77607000	-4.05802200	0.00585200
H	6.78229400	-4.46017000	-0.06595300
C	4.67581000	-4.88015200	-0.24757500
H	4.82244800	-5.92190900	-0.51674300
C	3.38444200	-4.36415700	-0.16086700
H	2.53442900	-5.00792500	-0.36203100
P	-3.38439800	-0.10450900	-0.02018200
C	-4.15121200	-1.43432100	-0.97516600
C	-3.40006300	-2.58128800	-1.27721500
C	-5.48767500	-1.33293700	-1.39102000
C	-3.99690600	-3.62366900	-1.98347400
H	-2.35931900	-2.65767200	-0.97877100
C	-6.07320100	-2.38211200	-2.09657600
H	-6.06230700	-0.43895100	-1.17476700
C	-5.32888700	-3.52623800	-2.39144800
H	-3.41139800	-4.50328300	-2.22256800
H	-7.10620000	-2.30293800	-2.42031900
H	-5.78559100	-4.34026300	-2.94616900
C	-4.27606900	1.42448300	-0.39220000
C	-5.46437400	1.70462100	0.30564600
C	-3.85128500	2.27126000	-1.42480100
C	-6.22954100	2.81429700	-0.04502400
H	-5.78536600	1.06215500	1.11918500
C	-4.63168500	3.37298600	-1.77406700
H	-2.92138400	2.08559400	-1.94813800

C	-5.81814600	3.64337800	-1.09186300
H	-7.14441400	3.03114000	0.49736800
H	-4.30242000	4.02297500	-2.57735800
H	-6.41988700	4.50336200	-1.36950100
C	-3.61707600	-0.42515400	1.74164100
C	-4.11522100	-1.65698100	2.18325200
C	-3.29039100	0.58106400	2.66748900
C	-4.28610500	-1.88125800	3.55041100
H	-4.37343700	-2.42974300	1.46710000
C	-3.45078800	0.34132700	4.02932400
H	-2.91367300	1.54118200	2.32796800
C	-3.94971800	-0.88778200	4.47096500
H	-4.68400900	-2.83131000	3.89338400
H	-3.19563000	1.11616000	4.74510900
H	-4.08078700	-1.06731400	5.53352300
Cl	3.65335900	0.22934400	-3.73092300

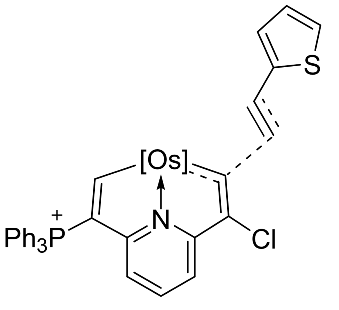

TS

[Os] = $OsCl(PPh_3)_2$

E= -5151.336821 a.u.

Os	0.99628700	0.16402000	0.14177500
Cl	2.31011900	0.25991200	4.58777700
Cl	1.60777300	0.16723900	-2.29052700
S	5.30739700	0.99536200	-2.68212700
P	0.62875300	2.58776400	0.05106500
P	1.25966000	-2.25414000	0.33620800
P	-3.57391700	-0.46697200	-0.68937100
N	-0.43100800	0.18970900	1.73777200
C	-0.83269100	-0.19435000	-0.67944700
H	-0.86754900	-0.41149100	-1.73960800
C	-1.98312800	-0.15659100	0.05437900
C	-1.76408500	0.12678400	1.47070700
C	-2.69502600	0.27013500	2.49947400
H	-3.75456900	0.29072800	2.27574500
C	-2.23707700	0.38545000	3.81665400
H	-2.95228400	0.48901400	4.62706900
C	-0.87911800	0.35604400	4.08922600
H	-0.49167800	0.41326100	5.09798100
C	0.02263300	0.27478200	3.01965300
C	1.45915500	0.24077800	3.03987100
C	2.06057200	0.12612000	1.84494000
C	3.31358600	0.91138300	0.42777400
H	3.35788800	1.86332200	0.92634100
C	3.99152900	0.20857200	-0.38324600
C	4.75556600	-0.19207800	-1.46158200
C	5.20227000	-1.45818000	-1.83398400
H	4.98067900	-2.33667300	-1.24740000
C	5.94294400	-1.47920600	-3.03439100
H	6.36197600	-2.37849000	-3.47089700
C	6.07051800	-0.23108600	-3.60741300
H	6.57724000	0.01181900	-4.53287300
C	2.00683600	3.70395100	-0.43091100
C	2.81640200	3.35104600	-1.52096100
H	2.68073700	2.39213000	-2.00633700
C	3.79401500	4.23391600	-1.97666700
H	4.41862700	3.94708900	-2.81649600
C	3.97523600	5.47025700	-1.35471900
H	4.74257900	6.15201500	-1.70933800
C	3.16438600	5.82942900	-0.27753300
H	3.29473700	6.79178600	0.20865300
C	2.17834300	4.95542900	0.17978100
H	1.55041800	5.25145600	1.01206100
C	-0.68864700	3.22016900	-1.07292400
C	-1.30304200	4.45969900	-0.82519800
H	-1.05292000	5.02278600	0.06676300
C	-2.23262900	4.98015500	-1.72413900
H	-2.70151300	5.93811100	-1.51771500
C	-2.54064100	4.28481100	-2.89663100
H	-3.25839800	4.69604400	-3.60046900
C	-1.89902700	3.07757800	-3.17079000
H	-2.10409900	2.54469400	-4.09288700
C	-0.97962100	2.54452900	-2.26497900
H	-0.45806800	1.62672400	-2.50426900
C	0.14302000	3.17027200	1.71852100
C	1.13894400	3.31468100	2.69784300
H	2.18141700	3.16145600	2.44222800
C	0.80384000	3.65959700	4.00588800
H	1.58820000	3.76193200	4.74874900
C	-0.53201600	3.85738700	4.35712900
H	-0.79288900	4.12861000	5.37572600
C	-1.53065500	3.69474700	3.39655900
H	-2.57457300	3.82967600	3.66425400
C	-1.19796100	3.34459200	2.08941400
H	-1.98840800	3.20180500	1.36317000
C	0.51079300	-2.82793600	1.91686800
C	1.31849600	-3.17813200	3.00663100
H	2.39522000	-3.20377300	2.90199100
C	0.74972500	-3.48426900	4.24335600
H	1.39555400	-3.74151400	5.07709300
C	-0.63354400	-3.45691500	4.40837700
H	-1.07526700	-3.69608700	5.37110100
C	-1.44696900	-3.11801300	3.32632300
H	-2.52507400	-3.09054800	3.44862100
C	-0.88086700	-2.79689700	2.09557900
H	-1.52182600	-2.51256000	1.27223500
C	2.94236800	-2.96804500	0.41284100
C	3.24799000	-4.18719400	-0.20650300
H	2.50801400	-4.69334800	-0.81563000
C	4.51103100	-4.75923000	-0.03955100
H	4.74144400	-5.70187200	-0.52694500
C	5.46780600	-4.12742100	0.75669700
H	6.44769400	-4.57663600	0.88882500
C	5.16431800	-2.91353500	1.37819700
H	5.90781500	-2.41089600	1.98918700
C	3.91211800	-2.32771800	1.20106300
H	3.66642400	-1.38619700	1.68549200
C	0.43312500	-3.21786100	-0.99519500
C	0.90477000	-3.04064900	-2.30763700
H	1.72014600	-2.35515500	-2.49975600
C	0.32697500	-3.74086800	-3.36576300
H	0.71249700	-3.59852300	-4.37115900
C	-0.73391200	-4.62038700	-3.13544100
H	-1.18294200	-5.16490700	-3.96126000

C	-1.20153700	-4.80885900	-1.83417300
H	-2.01565800	-5.50078300	-1.63893100
C	-0.61400000	-4.12327600	-0.76836200
H	-0.96570300	-4.31707400	0.23615200
C	-4.29763700	-2.05778300	-0.20362800
C	-4.18987400	-2.51882100	1.11811300
H	-3.68489000	-1.92082500	1.86603800
C	-4.72573300	-3.75523200	1.46979300
H	-4.62945700	-4.11180600	2.49045500
C	-5.37812400	-4.53602900	0.51296900
H	-5.79159400	-5.50075900	0.79028300
C	-5.50090900	-4.07474000	-0.79845900
H	-6.01193000	-4.67626500	-1.54362900
C	-4.96340400	-2.84012900	-1.16071200
H	-5.05216300	-2.49443100	-2.18405700
C	-3.38805600	-0.51367000	-2.48274000
C	-4.01917600	0.44060700	-3.28954300
H	-4.61992100	1.22469300	-2.84334200
C	-3.86339400	0.38263900	-4.67504600
H	-4.35741200	1.11870700	-5.30166000
C	-3.07207500	-0.61198600	-5.25054400
H	-2.94629400	-0.64761000	-6.32831500
C	-2.44573300	-1.56704500	-4.44417500
H	-1.83002700	-2.34347100	-4.88448700
C	-2.60757900	-1.53032900	-3.06272500
H	-2.13580100	-2.28928000	-2.44792200
C	-4.68663000	0.87412400	-0.22366600
C	-4.19011400	2.18723800	-0.28257900
H	-3.17202800	2.37429800	-0.60602100
C	-5.01753300	3.24923300	0.07184400
H	-4.63353200	4.26262100	0.01772400
C	-6.32842500	3.00550500	0.49254600
H	-6.96892800	3.83551600	0.77488700
C	-6.81941100	1.69933800	0.55106800
H	-7.83788000	1.51377100	0.87732000
C	-6.00195200	0.62768000	0.19031900
H	-6.37890700	-0.38891000	0.23756700

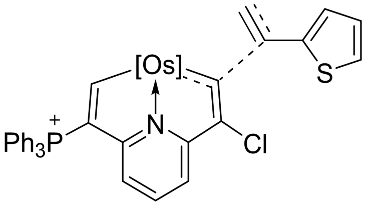

TS'

[Os] = $OsCl(PPh_3)_2$

E= -5151.311736 a.u.

Os	-0.88442800	0.33781300	-0.20175900
Cl	-3.06377400	0.61619400	3.87959400
Cl	-1.84098900	0.58468700	-2.43618500
P	-1.37148800	-2.05173900	-0.26243000
P	-0.37579600	2.68202800	-0.33531800
P	3.68665000	-0.65195900	-0.08486400
N	0.11336900	0.05140700	1.60779400
C	1.07671700	-0.01984700	-0.73580700
H	1.45118800	-0.05203000	-1.75607100
C	1.99562000	-0.22412500	0.26778200
C	1.44621800	-0.22421000	1.62314600
C	2.09323900	-0.48419900	2.83316800
H	3.14822900	-0.72744700	2.84828800
C	1.35778700	-0.44660800	4.01892300
H	1.84932000	-0.65464500	4.96427600
C	-0.00079600	-0.15885500	3.99424800
H	-0.59553100	-0.13666500	4.89766200
C	-0.61235600	0.09214300	2.76065700
C	-1.99000300	0.46223400	2.50165500
C	-2.28308800	0.62510900	1.17593000
C	-4.11953100	1.19958700	0.91759600
C	-4.10497900	2.35283500	1.37741500
C	-2.93390200	-2.82060800	-0.87190500
C	-3.45961200	-2.39155800	-2.09791300
H	-3.07446600	-1.49469400	-2.56463000
C	-4.46989100	-3.12323700	-2.72168600
H	-4.86298800	-2.77957300	-3.67417800
C	-4.97235200	-4.28263700	-2.13254700
H	-5.75516200	-4.85262400	-2.62465200
C	-4.46213900	-4.70692700	-0.90514100
H	-4.84574100	-5.60811100	-0.43513900
C	-3.44310600	-3.98851100	-0.28147200
H	-3.03917600	-4.35225500	0.65558500
C	-0.22264700	-3.02905200	-1.32738100
C	0.01099900	-4.39158400	-1.08520000
H	-0.43494000	-4.87283900	-0.22152600
C	0.81296400	-5.13461900	-1.95057200
H	0.98459100	-6.18926000	-1.75374900
C	1.38181500	-4.53015100	-3.07541200
H	2.00686800	-5.11091300	-3.74749700
C	1.11575800	-3.18771300	-3.34654600
H	1.52779200	-2.71619300	-4.23329100
C	0.30999400	-2.44296000	-2.48266300
H	0.06347300	-1.41552300	-2.72195700
C	-1.15507700	-2.74099900	1.41828600
C	-2.21537700	-2.66007300	2.33396100
H	-3.17484000	-2.27608500	2.01023400
C	-2.04348900	-3.07360600	3.65399100
H	-2.87488300	-3.00513400	4.34876300
C	-0.80651500	-3.55875300	4.08148900
H	-0.67362900	-3.88051700	5.11017200
C	0.25994700	-3.61952600	3.18344200
H	1.23184300	-3.98135100	3.50651700
C	0.08862100	-3.20762200	1.86350100
H	0.92866200	-3.24795100	1.18377100
C	0.76610500	3.23389500	0.99237900
C	0.35530600	3.07914300	2.32706700
H	-0.62034300	2.65976700	2.54181100
C	1.18045700	3.48055200	3.37458900
H	0.84752900	3.35044200	4.39974600
C	2.42268600	4.06128900	3.10358900
H	3.05668600	4.39548900	3.92003300
C	2.83711200	4.21994400	1.78147300
H	3.79963100	4.67080700	1.56020200
C	2.02171300	3.79460800	0.73041600
H	2.35849800	3.92141300	-0.29169000
C	-1.80045600	3.83399300	-0.18927500
C	-2.84443700	3.68917200	-1.11679600
H	-2.80340800	2.89290400	-1.85201600
C	-3.94102400	4.54684400	-1.07722900
H	-4.74607600	4.41941100	-1.79458500
C	-4.01439500	5.55373700	-0.11091000
H	-4.87445400	6.21571300	-0.07672000
C	-2.97602800	5.70724800	0.80860400
H	-3.01996500	6.49368800	1.55646500
C	-1.86838400	4.85509400	0.76756400

H	-1.06457100	4.99433600	1.48093500
C	0.40592400	3.33259100	-1.86938500
C	0.81853300	2.50741400	-2.92004000
H	0.64518100	1.44385500	-2.86978200
C	1.38933600	3.06017400	-4.06905200
H	1.67553700	2.40662900	-4.88751400
C	1.56413300	4.43867600	-4.17625100
H	2.00264400	4.86681000	-5.07295300
C	1.15097700	5.27176700	-3.13268000
H	1.27037100	6.34812300	-3.21247300
C	0.56613600	4.72427800	-1.99411800
H	0.22682300	5.38093800	-1.19928300
C	4.90966600	0.45643100	0.65349200
C	4.55525300	1.40538500	1.61913700
H	3.52384600	1.52696500	1.92302400
C	5.54490000	2.20814700	2.18867700
H	5.26732700	2.93913800	2.94034700
C	6.87504400	2.07574800	1.79196200
H	7.63995300	2.70294400	2.23964100
C	7.22640700	1.14329500	0.81051900
H	8.25919800	1.05004000	0.48984800
C	6.24828900	0.33510500	0.23913800
H	6.51948700	-0.38355900	-0.52873600
C	3.95223500	-0.56715800	-1.87045800
C	4.22144200	-1.70536500	-2.63924000
H	4.26857600	-2.68360300	-2.17629900
C	4.42788900	-1.57629200	-4.01348700
H	4.63886200	-2.45919400	-4.60875000
C	4.36685000	-0.32029700	-4.61756700
H	4.53070800	-0.22470400	-5.68660500
C	4.10181800	0.81716600	-3.84882600
H	4.05268300	1.79600200	-4.31318800
C	3.89641900	0.69916900	-2.47780400
H	3.68592400	1.58391600	-1.88502200
C	4.02222200	-2.32877300	0.51081200
C	3.18602800	-3.36392700	0.06230100
H	2.38987200	-3.15973800	-0.64505400
C	3.37349700	-4.65923200	0.53829200
H	2.72425800	-5.45279400	0.18470400
C	4.38150100	-4.92502000	1.46889500
H	4.52056700	-5.93457000	1.84346300
C	5.20984400	-3.89526300	1.91851000
H	5.99209300	-4.10087800	2.64249500
C	5.03617700	-2.59613700	1.44031200
H	5.68047300	-1.79837200	1.79280600
C	-4.89738700	0.14495900	0.33495500
C	-5.34317900	-1.00728200	0.94048600
S	-5.55875000	0.31335800	-1.27091200
C	-6.22663300	-1.75756500	0.11504200
H	-5.08218300	-1.26578900	1.95949200
C	-6.44080200	-1.16441400	-1.10055600
H	-6.67460600	-2.69908200	0.40748300
H	-7.04350000	-1.52957200	-1.92019500
H	-3.75078800	3.29086300	1.74787600

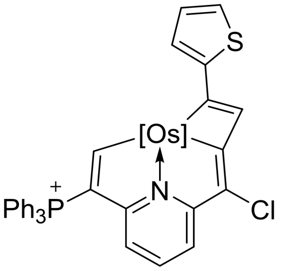

4a

[Os] = $OsCl(PPh_3)_2$

E= -5151.408381 a.u.

Os	0.97231600	-0.00623200	-0.06877100
Cl	2.55816200	0.24309300	4.37870500
Cl	1.25598600	-0.06462200	-2.55653700
S	5.82365100	0.67699300	-0.89464800
P	0.86141100	2.43289900	-0.10843100
P	1.17118000	-2.42421400	0.13587000
P	-3.73231900	-0.13132000	-0.47383500
N	-0.32921400	0.02840200	1.65881900
C	-0.97869000	-0.16030600	-0.75431200
H	-1.17235300	-0.25392700	-1.81589700
C	-2.04539100	-0.12161100	0.10070600
C	-1.68503300	0.01451400	1.50273200
C	-2.53657900	0.10665000	2.60108700
H	-3.60900000	0.13623500	2.45547800
C	-1.99082200	0.15873900	3.88806100
H	-2.64529000	0.21929600	4.75199300
C	-0.61983800	0.13714500	4.05270600
H	-0.15690300	0.18026600	5.02985500
C	0.20630100	0.09432600	2.91837500
C	1.62675300	0.15498500	2.89429100
C	2.17996800	0.21181600	1.66117500
C	3.46699900	0.32252800	1.10274700
H	4.42109500	0.42896300	1.62129300
C	3.21035100	0.09760200	-0.22575900
C	4.23962000	-0.05186700	-1.21471700
C	4.30296200	-0.80731300	-2.37172800
H	3.45756700	-1.36516700	-2.74092300
C	5.58294400	-0.80871700	-2.98511500
H	5.81198200	-1.35055800	-3.89562800
C	6.50048600	-0.04503800	-2.30904100
H	7.53112100	0.14811200	-2.57602500
C	2.33148300	3.36680500	-0.68441600
C	3.03347300	2.88319600	-1.79658100
H	2.75182200	1.94065800	-2.24734300
C	4.09585900	3.61437900	-2.32648100
H	4.64170100	3.21749200	-3.17687700
C	4.46153500	4.83510000	-1.75967200
H	5.29393900	5.39987000	-2.16904300
C	3.74923900	5.33321500	-0.66681200
H	4.02067600	6.28866000	-0.22739700
C	2.68356000	4.60909400	-0.13461100
H	2.13340600	5.01385700	0.70713300
C	-0.44883400	3.24447500	-1.12507200
C	-0.91489300	4.52713500	-0.78921700
H	-0.55812700	5.01680900	0.10976300
C	-1.83845700	5.18006800	-1.60376900
H	-2.18971800	6.17119400	-1.33061800
C	-2.29376900	4.57215100	-2.77705800
H	-3.00673500	5.08622300	-3.41517400
C	-1.80577500	3.31625800	-3.13554500
H	-2.12851200	2.84567700	-4.05816000
C	-0.89044700	2.65293500	-2.31513000
H	-0.49114400	1.69398100	-2.61901900
C	0.52015900	3.05057500	1.58324100
C	1.57040600	3.24103700	2.49291500
H	2.59676800	3.10220400	2.17230200

C	1.30494000	3.59446800	3.81534200
H	2.12983500	3.73521300	4.50633600
C	-0.01186400	3.74903000	4.25048000
H	-0.21610300	4.02232800	5.28144100
C	-1.06377400	3.54120500	3.35745500
H	-2.09343700	3.64152100	3.68810900
C	-0.79995200	3.19026100	2.03549700
H	-1.62818200	3.01957000	1.35984600
C	0.64376700	-2.95569500	1.81718900
C	1.52445200	-3.17697100	2.88025700
H	2.59397800	-3.14524300	2.72556600
C	1.03450400	-3.45154300	4.15910300
H	1.73383200	-3.61636300	4.97285600
C	-0.33828600	-3.51245600	4.39066000
H	-0.71561400	-3.72388700	5.38662900
C	-1.22499200	-3.30833500	3.33082100
H	-2.29702200	-3.35967400	3.50115400
C	-0.73749700	-3.03003400	2.05820500
H	-1.42640400	-2.88204900	1.23524600
C	2.83743000	-3.12728500	-0.17461600
C	3.05650200	-3.92800200	-1.30618400
H	2.22866400	-4.20657200	-1.94667900
C	4.34212100	-4.36228300	-1.62802100
H	4.49315400	-4.97576900	-2.51130800
C	5.42607800	-4.00825800	-0.82579900
H	6.42784800	-4.33829800	-1.08333300
C	5.21692700	-3.22390900	0.30886100
H	6.05412400	-2.93644400	0.93740800
C	3.93613600	-2.78214500	0.63006600
H	3.80668300	-2.13315500	1.48416400
C	0.10333700	-3.49291900	-0.91835000
C	-0.30541100	-3.07826800	-2.19145000
H	-0.02672900	-2.09666200	-2.55223800
C	-1.02712400	-3.94588900	-3.01559700
H	-1.31358700	-3.61903600	-4.01114000
C	-1.35945800	-5.22662900	-2.57573500
H	-1.92187800	-5.89787000	-3.21819900
C	-0.94695700	-5.64944100	-1.30974600
H	-1.18681400	-6.65029700	-0.96322900
C	-0.21176600	-4.79449100	-0.49165300
H	0.11947500	-5.14099200	0.48092000
C	-4.65791400	-1.60313300	0.03149100
C	-4.21906400	-2.40751100	1.09144900
H	-3.32328900	-2.14492700	1.63739700
C	-4.94345000	-3.54454500	1.44593800
H	-4.59365500	-4.17158700	2.26019600
C	-6.10802200	-3.87768700	0.75266100
H	-6.66940900	-4.76462400	1.02975600
C	-6.55083900	-3.07493600	-0.30205600
H	-7.45353900	-3.33654200	-0.84507500
C	-5.82732700	-1.94251500	-0.66939900
H	-6.16056900	-1.33411300	-1.50443100
C	-3.77017100	-0.11920900	-2.27785200
C	-4.23618600	0.99305100	-2.98836200
H	-4.56777100	1.88082400	-2.46328400
C	-4.26827400	0.95518800	-4.38294400
H	-4.63579100	1.81506000	-4.93428600
C	-3.82963800	-0.18014900	-5.06423800
H	-3.85306000	-0.20390400	-6.14935000
C	-3.36741500	-1.29147900	-4.35278500
H	-3.03384000	-2.17764700	-4.88283400
C	-3.34359400	-1.27036700	-2.96174100
H	-2.99321600	-2.13928400	-2.41463800
C	-4.56408100	1.34482200	0.15610600
C	-3.90718600	2.57785000	0.00974100
H	-2.94454900	2.63141200	-0.48695900
C	-4.50019800	3.73656900	0.50438800
H	-3.98933400	4.68599200	0.38420500
C	-5.73804000	3.66881200	1.15010600
H	-6.19487500	4.57291600	1.54092100
C	-6.39016500	2.44261700	1.29436200
H	-7.35155200	2.39193100	1.79579600
C	-5.80808600	1.27631200	0.79641600
H	-6.31239400	0.32286900	0.91329400

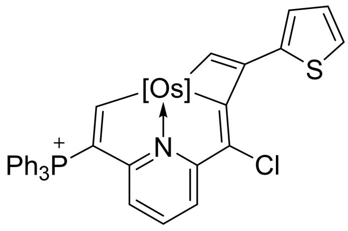

4a'

$[Os] = OsCl(PPh_3)_2$

E = -5151.408381

Os	-0.79763000	0.09154700	-0.50882200
Cl	-3.34090900	0.12188500	3.49433100
Cl	-0.62344800	0.18518900	-3.01977400
P	-0.85672100	-2.34372900	-0.46673000
P	-0.92491800	2.51819600	-0.42789500
P	3.86066000	-0.09343500	0.13936800
N	0.05479900	0.06426100	1.44237000
C	1.25680100	0.09632500	-0.77664700
H	1.69630600	0.11296400	-1.76742100
C	2.09240700	0.03959400	0.30859800
C	1.41247000	-0.00351700	1.59493700
C	1.98126400	-0.09056500	2.86323400
H	3.05449100	-0.18695300	2.97085400
C	1.15523500	-0.05090500	3.98938600
H	1.58976500	-0.10650600	4.98247200
C	-0.21494400	0.05715800	3.83414600
H	-0.88542300	0.09061500	4.68231000
C	-0.75699900	0.09276300	2.54191700
C	-2.15055600	0.10983100	2.20183200
C	-2.43365900	0.03086800	0.88526900
C	-3.53660100	-0.08554900	-0.03159800
C	-2.82233500	0.03364300	-1.18440100
C	-2.26696300	-3.17073000	-1.30435100
C	-2.64734100	-2.69877300	-2.56989600
H	-2.13548700	-1.84858900	-3.00611500
C	-3.67213500	-3.32917900	-3.27446700
H	-3.95947800	-2.95251700	-4.25179800
C	-4.32431600	-4.43659100	-2.72770400
H	-5.12770100	-4.92169300	-3.27416500
C	-3.92917700	-4.92705000	-1.48285600
H	-4.41944500	-5.79801800	-1.05817100
C	-2.89890900	-4.30485800	-0.77695400
H	-2.59539000	-4.70669500	0.18252400
C	0.55657700	-3.27018800	-1.19880700
C	0.87401600	-4.56624500	-0.75985500
H	0.32308000	-5.01212500	0.06092200
C	1.89929500	-5.28602900	-1.37159300
H	2.13591500	-6.28736500	-1.02286700

C	2.60616000	-4.73016000	-2.44180400
H	3.40013200	-5.29559500	-2.92102800
C	2.26787300	-3.45969300	-2.90734900
H	2.79059100	-3.02932900	-3.75506700
C	1.24916200	-2.73045700	-2.29026900
H	0.96808200	-1.75890700	-2.67816700
C	-0.90828900	-2.91338900	1.27328300
C	-2.12679000	-2.96380000	1.96753800
H	-3.05754100	-2.75150300	1.45339400
C	-2.15195600	-3.26878800	3.32797500
H	-3.10334100	-3.30164000	3.84886600
C	-0.96246400	-3.50884800	4.01708100
H	-0.98527400	-3.74295000	5.07725200
C	0.25560300	-3.43515400	3.34019700
H	1.18968400	-3.60181200	3.86878900
C	0.28256100	-3.13574100	1.97995600
H	1.23678400	-3.06848600	1.47374400
C	-0.72927800	3.10420800	1.30689200
C	-1.79105400	3.40613900	2.16515800
H	-2.80943200	3.41120800	1.80300800
C	-1.55110500	3.71742800	3.50553300
H	-2.38918100	3.94621000	4.15651100
C	-0.25013000	3.73349700	4.00436600
H	-0.06754100	3.97328100	5.04754900
C	0.81792600	3.44938400	3.15006400
H	1.83629700	3.46762100	3.52889600
C	0.57935500	3.13703000	1.81567500
H	1.41021800	2.92903100	1.15141500
C	-2.47383700	3.22200900	-1.10523300
C	-2.44352100	3.91801700	-2.32333400
H	-1.49936100	4.10820400	-2.81942600
C	-3.62743900	4.36502500	-2.91059900
H	-3.58582800	4.90168200	-3.85370900
C	-4.85520600	4.12453400	-2.29371300
H	-5.77484700	4.47275400	-2.75436300
C	-4.89663200	3.42813500	-1.08489200
H	-5.84402400	3.21777800	-0.59832700
C	-3.71759000	2.97336200	-0.50048000
H	-3.78183900	2.40427000	0.41515400
C	0.37179600	3.52266500	-1.26406900
C	1.11431800	3.02599100	-2.34090400
H	0.93614200	2.02042500	-2.69711600
C	2.04035100	3.84536100	-2.99237000
H	2.58985900	3.45532400	-3.84430900
C	2.24094700	5.15943700	-2.57231000
H	2.96140000	5.79309000	-3.08130400
C	1.49556700	5.66407300	-1.50363300
H	1.63399800	6.69056500	-1.17742500
C	0.56255100	4.85541400	-0.85940500
H	-0.01904700	5.26039300	-0.03822800
C	4.76236900	1.32091600	0.81989700
C	4.14082400	2.22675100	1.68897700
H	3.11099700	2.07708000	1.98291500
C	4.85451900	3.32005400	2.17807100
H	4.36632800	4.02644900	2.84233100
C	6.18697800	3.50865700	1.80864400
H	6.73987300	4.36181200	2.18944400
C	6.81040000	2.60537800	0.94313800
H	7.84468100	2.75578200	0.65022100
C	6.10159100	1.51592400	0.44227600
H	6.58058400	0.82914300	-0.24896700
C	4.30056200	-0.16337900	-1.60989600
C	4.81265400	-1.32973400	-2.18984300
H	4.94659800	-2.22603200	-1.59621100
C	5.14883100	-1.33524200	-3.54423400
H	5.55201700	-2.23785700	-3.99272300
C	4.96719100	-0.18835900	-4.31692800
H	5.22684100	-0.19805000	-5.37099400
C	4.45896000	0.97720000	-3.73535300
H	4.32555700	1.87153000	-4.33535000
C	4.13216400	0.99828400	-2.38303900
H	3.74465900	1.90753300	-1.93505200
C	4.41255100	-1.60573500	0.96309500
C	3.73462200	-2.79823200	0.65966700
H	2.92775100	-2.80167400	-0.06532800
C	4.10342400	-3.98132200	1.29470600
H	3.57827200	-4.89913300	1.05234500
C	5.13668500	-3.97795800	2.23614500
H	5.41759600	-4.90050800	2.73494600
C	5.80955200	-2.79228000	2.53727900
H	6.61184000	-2.79115200	3.26844900
C	5.45287200	-1.60263200	1.90101900
H	5.97326900	-0.68091000	2.13874200
H	-3.19056300	0.09294500	-2.20220800
C	-4.97248500	-0.28865200	0.11698800
C	-5.75551500	-1.03422800	-0.73823400
S	-5.96742700	0.51142700	1.31704500
C	-7.14572800	-0.96419600	-0.43737100
H	-5.33102700	-1.62224400	-1.54435100
C	-7.41437200	-0.16201900	0.63914900
H	-7.91163300	-1.49611700	-0.99043400
H	-8.37224100	0.05145600	1.09391800

Et_2O:

E = -233.802087 a.u.

O	0.00000000	0.00000000	0.83605600
C	0.00000000	1.19634600	0.06889800
H	-0.42817600	1.96681800	0.71934200
H	-0.66411600	1.10363200	-0.80410800
C	1.40445200	1.60361700	-0.37126900
H	1.85154500	0.85241600	-1.03180200
H	1.37735200	2.55537800	-0.91517200
H	2.05488600	1.71876700	0.50173800
C	0.00000000	-1.19634600	0.06889800
H	0.66411600	-1.10363200	-0.80410800
H	0.42817600	-1.96681800	0.71934200
C	-1.40445200	-1.60361700	-0.37126900
H	-1.37735200	-2.55537800	-0.91517200
H	-2.05488600	-1.71876700	0.50173800
H	-1.85154500	-0.85241600	-1.03180200

$(Et_2O)_2H^+$:

E = -468.054149

H	-0.21140500	0.05818000	-0.26919100
O	0.97909800	-0.28974300	-0.25595700
C	1.56552100	-0.54680500	1.06150300
H	2.13199900	0.33837700	1.36494800
H	0.70826500	-0.65509200	1.73088800
C	2.41383400	-1.80232800	1.03982900
H	3.26801700	-1.70570200	0.36311000
H	2.80644600	-1.98821200	2.04488800
H	1.82057000	-2.66898100	0.73429900
C	1.81985800	0.41537500	-1.22955700
H	2.82375900	-0.00348400	-1.13694400

H	1.41718700	0.12263300	-2.20154500
C	1.79427800	1.91893700	-1.03011700
H	2.43171700	2.39089600	-1.78481100
H	0.78181400	2.31686000	-1.14806800
H	2.17696700	2.20866000	-0.04645600
O	-1.34857600	0.30999800	-0.38448200
C	-2.04277400	-0.76917400	-1.10902700
H	-3.06044200	-0.40847100	-1.27399000
H	-1.52777900	-0.81096100	-2.07072400
C	-1.99710400	-2.09619500	-0.37672600
H	-2.53014000	-2.06221300	0.57778600
H	-2.48129100	-2.85759800	-0.99674400
H	-0.96432800	-2.41476000	-0.20233600
C	-1.99746000	0.80460300	0.83729300
H	-1.98630100	0.00777700	1.58668300
H	-3.03187400	1.01625100	0.55696300
C	-1.27464600	2.05009800	1.30478800
H	-1.78227300	2.44177200	2.19167900
H	-0.23674200	1.84102900	1.58260500
H	-1.28738100	2.82393700	0.53254800

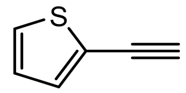

E = -629.348406

C	-1.95970300	-0.24649700	-0.00016000
C	-1.68476400	1.09543800	-0.00002300
C	-0.29006200	1.37394600	0.00001000
C	0.49050200	0.23661100	-0.00009500
S	-0.51645100	-1.20295100	0.00021300
H	-2.92748800	-0.72941600	-0.00027600
H	-2.45194200	1.86136500	-0.00005400
H	0.13709200	2.36960800	0.00000900
C	1.89706700	0.13663200	-0.00018600
C	3.10347300	0.03536700	-0.00012200
H	4.16648100	-0.04331500	0.00037100

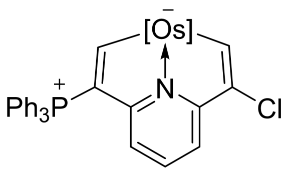

P2-1

[Os] = $OsCl(PPh_3)_2$

E = -4521.819890 a.u.

Os	-1.22411500	0.04829700	-0.39384500
Cl	-3.82945800	-0.12916400	3.50521800
Cl	-1.97927200	0.06666500	-2.81952800
P	-1.34823600	-2.30954800	-0.51801300
P	-1.39932300	2.39416500	-0.35358400
P	3.33752400	0.01280400	0.08632000
N	-0.41831800	-0.05287600	1.46401400
C	0.74029200	0.13942600	-0.84220700
C	1.59349400	0.06950200	0.27420500
C	0.94352000	-0.08143700	1.58065600
C	1.51983700	-0.23935300	2.84671500
H	2.59641500	-0.31038000	2.95519200
C	0.69060600	-0.31516800	3.96723500
H	1.13086700	-0.43264300	4.95332500
C	-0.69229600	-0.25202200	3.83082200
H	-1.35062800	-0.31228300	4.68727800
C	-1.24149100	-0.13238700	2.55007400
C	-2.64219500	-0.08420200	2.16101100
C	-2.98598100	-0.00541800	0.85256300
H	-4.06034600	0.03760000	0.65089500
C	-2.89075800	-3.06435600	-1.17168500
C	-2.99378400	-4.45208600	-1.34450900
H	-2.14174200	-5.09101900	-1.13634600
C	-4.18775400	-5.02115900	-1.78215400
H	-4.25759900	-6.09776700	-1.91202100
C	-5.29103000	-4.20763500	-2.05311000
H	-6.22227300	-4.65120000	-2.39515800
C	-5.19197800	-2.82629400	-1.88839700
H	-6.04316700	-2.18779000	-2.10760800
C	-3.99633000	-2.25379600	-1.45135200
H	-3.89986700	-1.18260900	-1.34188900
C	-1.21045800	-3.07754100	1.14513400
C	-2.36459200	-3.34339000	1.89317300
H	-3.34158600	-3.20917800	1.44241100
C	-2.26546800	-3.75979200	3.22085300
H	-3.17039800	-3.94844000	3.79082900
C	-1.01385500	-3.91703500	3.81715900
H	-0.93950300	-4.23740500	4.85277200
C	0.14113900	-3.64692000	3.08124500
H	1.12179300	-3.74651800	3.53830000
C	0.04185600	-3.22107100	1.75937500
H	0.94371400	-2.98599600	1.20949200
C	-0.01697100	-3.15507300	-1.48210100
C	0.48214000	-4.42429200	-1.15283200
H	0.08597300	-4.95544600	-0.29382000
C	1.51125800	-4.99818000	-1.90260800
H	1.88783300	-5.98268700	-1.63649300
C	2.05499200	-4.30958700	-2.98898000
H	2.86171400	-4.75328600	-3.56653400
C	1.54853500	-3.05476200	-3.33510400
H	1.95826000	-2.51473600	-4.18362500
C	0.51701700	-2.48113300	-2.59078800
H	0.10755300	-1.51429900	-2.86440600
C	-3.00458200	3.16375400	-0.80201900
C	-4.04585700	2.36919000	-1.29278700
H	-3.87333400	1.31034400	-1.43527000
C	-5.27324500	2.94994900	-1.61848700
H	-6.07565100	2.32715300	-2.00398800
C	-5.46719500	4.32140100	-1.45562800
H	-6.42404300	4.77070800	-1.70774400
C	-4.42708100	5.12006000	-0.97138300
H	-4.57279200	6.18954400	-0.84567500
C	-3.20035900	4.54429200	-0.64953900
H	-2.39508900	5.16768700	-0.27233100
C	-1.12479600	2.99458800	1.35793500
C	0.17682500	3.11388900	1.86638100
H	1.02129800	3.01191400	1.19406800
C	0.38946000	3.35319700	3.22219900
H	1.40285300	3.44623700	3.60438700
C	-0.69558500	3.45889500	4.09451400
H	-0.52938600	3.63223200	5.15403100
C	-1.99333300	3.33316300	3.59924200
H	-2.84407200	3.39661000	4.27100400
C	-2.20794700	3.10201600	2.24040300
H	-3.21941400	2.98448600	1.86874600
C	-0.19722200	3.35373800	-1.37402300
C	0.11871200	2.84697500	-2.64412300
H	-0.33562200	1.91652300	-2.97156700

C	0.99232300	3.54227700	-3.47980500
H	1.21380900	3.14463200	-4.46636600
C	1.58104700	4.73516100	-3.05343900
H	2.26718200	5.27196300	-3.70376500
C	1.27284600	5.24289800	-1.79001800
H	1.71976500	6.17393400	-1.45142600
C	0.37823900	4.56359700	-0.96052200
H	0.14007500	4.97323200	0.01526700
C	4.26857100	1.34352000	0.90797900
C	5.60006700	1.58787000	0.53501500
H	6.07045700	0.97912200	-0.23145100
C	6.31216800	2.62461200	1.13445400
H	7.34064200	2.81301500	0.84119300
C	5.69884100	3.42716100	2.10026200
H	6.25324700	4.23967700	2.56069700
C	4.37366700	3.19031000	2.46712200
H	3.89229000	3.81790900	3.21117000
C	3.65658800	2.15074400	1.87455800
H	2.63040700	1.96440100	2.16230800
C	3.98962200	-1.56716000	0.70458400
C	3.33826800	-2.73642700	0.27952700
H	2.50564900	-2.67676300	-0.41403800
C	3.75422200	-3.97559600	0.76016600
H	3.24302200	-4.87153000	0.42451100
C	4.80834900	-4.05388600	1.67397500
H	5.12335700	-5.02025500	2.05684900
C	5.45375600	-2.89192400	2.10052000
H	6.26968900	-2.95196800	2.81469700
C	5.04930900	-1.64715000	1.61612300
H	5.54665800	-0.74452400	1.95464700
C	3.76274600	0.17419000	-1.66632000
C	4.23397700	-0.91053800	-2.41361800
H	4.36330300	-1.88122800	-1.95002700
C	4.52462000	-0.74299100	-3.76826100
H	4.88695700	-1.58719700	-4.34718400
C	4.34707400	0.50069500	-4.37436800
H	4.57202800	0.62705200	-5.42937200
C	3.87724700	1.58438400	-3.62716600
H	3.72580200	2.55128500	-4.09456800
C	3.58471100	1.42723500	-2.27581800
H	3.20221700	2.26828900	-1.70644100
H	1.23679500	0.25872200	-1.81052400

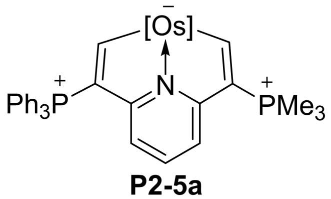

P2-5a

[Os] = $OsCl(PPh_3)_2$

E = -4522.606889 a.u.

Os	0.98271400	0.01308200	-0.58354100
Cl	1.39722700	-0.04951300	-3.06317100
P	0.95972900	2.37320600	-0.84168400
P	1.23236200	-2.34056700	-0.67044500
P	-3.52306200	-0.11319200	0.45307700
N	0.38589800	0.11160900	1.34662200
C	-1.04645700	-0.15177500	-0.80296500
C	-1.75244400	-0.09368900	0.39636800
C	-0.96114600	0.06793700	1.61610600
C	-1.39965700	0.14582100	2.94333400
H	-2.45720900	0.11119700	3.17383200
C	-0.46169700	0.27433400	3.96694000
H	-0.79418300	0.34466900	4.99796500
C	0.89879300	0.31827200	3.67152300
H	1.61774100	0.42730400	4.47466300
C	1.31854400	0.22808900	2.33877100
C	2.67749400	0.21581300	1.79489500
C	2.80993400	0.13482400	0.41022400
H	3.84741300	0.09985400	0.05441400
C	2.32938900	3.13153500	-1.79426900
C	2.27344700	4.48124600	-2.17034200
H	1.38771300	5.07010100	-1.95462800
C	3.35115100	5.07309600	-2.82680600
H	3.29824700	6.11843100	-3.11682100
C	4.49203100	4.32101700	-3.11682700
H	5.32873900	4.78138500	-3.63436000
C	4.54806600	2.97474500	-2.75395100
H	5.42339800	2.38036600	-3.00048000
C	3.47045100	2.37916900	-2.09770200
H	3.48445400	1.32672900	-1.84960100
C	1.08006900	3.23053100	0.78272000
C	2.26852400	3.83591100	1.20953600
H	3.11752600	3.88683900	0.53603200
C	2.35810900	4.39393900	2.48718800
H	3.27719400	4.88576100	2.79565200
C	1.26863200	4.34175700	3.35830600
H	1.33957300	4.78160300	4.34914200
C	0.08093200	3.73649800	2.94059600
H	-0.77663200	3.68866000	3.60536200
C	-0.01088000	3.18816400	1.66417800
H	-0.93825900	2.72495400	1.35274200
C	-0.55633300	3.11242500	-1.58085300
C	-1.09834400	4.33204900	-1.14881800
H	-0.62381600	4.88359400	-0.34415400
C	-2.26093800	4.83552100	-1.73736700
H	-2.67163600	5.78191500	-1.39614200
C	-2.88878600	4.12803500	-2.76408900
H	-3.79433900	4.51925600	-3.21936100
C	-2.33997800	2.92432000	-3.21294400
H	-2.81726500	2.37314800	-4.01698900
C	-1.18011300	2.41724600	-2.62813700
H	-0.74179600	1.49009100	-2.98335700
C	2.57794600	-3.00602300	-1.71788600
C	3.67651500	-2.20072400	-2.04113800
H	3.66766900	-1.15865600	-1.75197700
C	4.74060700	-2.72802600	-2.77224300
H	5.58198100	-2.09265100	-3.03383300
C	4.71201600	-4.05880300	-3.19259800
H	5.53718100	-4.46525100	-3.77023300
C	3.61388200	-4.86399700	-2.88203000
H	3.58317100	-5.89739900	-3.21476400
C	2.55004000	-4.34036300	-2.14826600
H	1.69757800	-4.96879100	-1.90953600
C	1.58471500	-3.12338200	0.96502300
C	0.70628500	-2.90263700	2.03735000
H	-0.17117300	-2.28766700	1.89137800
C	0.94884700	-3.45598100	3.29326400
H	0.25106500	-3.26801600	4.10438300
C	2.08475500	-4.24035300	3.50392700
H	2.27410100	-4.67996900	4.47905600
C	2.97505700	-4.45636800	2.45015700

H	3.85834100	-5.07116600	2.60084000
C	2.72954600	-3.90116400	1.19130700
H	3.42357100	-4.08844800	0.38021700
C	-0.24266100	-3.25035000	-1.29209400
C	-0.63359400	-2.98177600	-2.61588300
H	-0.06772400	-2.26565500	-3.20495900
C	-1.74079800	-3.62682200	-3.16126900
H	-2.02067200	-3.42674200	-4.19159200
C	-2.49013600	-4.52157400	-2.38975900
H	-3.35446500	-5.02224800	-2.81734700
C	-2.11984300	-4.77156900	-1.06855900
H	-2.69723600	-5.46190400	-0.46026400
C	-0.99154500	-4.14932700	-0.52520900
H	-0.69371000	-4.38031100	0.49151400
C	-4.18170600	-1.58042900	1.28743500
C	-5.56784000	-1.79931600	1.34695300
H	-6.25526900	-1.07173100	0.92514200
C	-6.06032900	-2.96456100	1.92952800
H	-7.13158100	-3.13384600	1.97578700
C	-5.17586900	-3.92000900	2.43999200
H	-5.56384500	-4.82990700	2.88778600
C	-3.79818700	-3.71320400	2.36265500
H	-3.10942800	-4.46085200	2.74428000
C	-3.29896800	-2.54593800	1.78548100
H	-2.23126400	-2.39276100	1.70021300
C	-4.08841800	1.38902500	1.29447700
C	-3.54078600	2.60266600	0.84552000
H	-2.84767100	2.61662800	0.01065200
C	-3.88210500	3.79396100	1.48026500
H	-3.45475000	4.72606700	1.12517200
C	-4.75964200	3.78050100	2.56789000
H	-5.02003500	4.70931900	3.06639500
C	-5.30034600	2.57433400	3.01733500
H	-5.97912000	2.56361900	3.86456700
C	-4.96842100	1.37605000	2.38323400
H	-5.38059500	0.43991400	2.74412200
C	-4.20288500	-0.15344200	-1.21874000
C	-4.81430900	0.96394700	-1.79883900
H	-4.90458700	1.89186900	-1.24712500
C	-5.30485700	0.88035000	-3.10213800
H	-5.78225700	1.74563100	-3.55149100
C	-5.17952700	-0.30681400	-3.82441800
H	-5.56011400	-0.36594600	-4.83964500
C	-4.56884200	-1.42162700	-3.24282900
H	-4.46725300	-2.34596400	-3.80164200
C	-4.08544500	-1.35276700	-1.93994600
H	-3.61457100	-2.22184400	-1.49330200
H	-1.64173700	-0.29519900	-1.70864300
P	4.07753400	0.19203100	2.86977400
C	4.06094900	-1.24022000	3.99850700
H	4.06602600	-2.15374300	3.39772100
H	4.93071400	-1.22099100	4.66222500
H	3.14581600	-1.24090400	4.59376600
C	4.23718200	1.69779900	3.88933100
H	5.07819900	1.60830300	4.58362300
H	4.39034600	2.55079800	3.22411200
H	3.31591700	1.87723500	4.44751900
C	5.61523100	0.06540500	1.90981400
H	5.61392500	-0.85411300	1.31970800
H	5.71382700	0.92148900	1.23822600

H
6.46208600

0.05021300 2.60257900

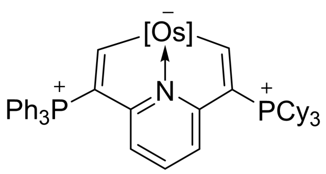

P2-5c
[Os] = $OsCl(PPh_3)_2$

E = -5108.857760 a.u.

Os	-0.20380200	-0.27511300	-1.02540300
Cl	-0.40821400	-0.64332500	-3.50918900
P	-0.16245000	2.01945100	-1.65593500
P	-0.18357300	-2.63987700	-0.88274000
P	-4.11694400	0.29727900	1.34853200
N	-0.13328700	0.04515600	0.96297900
C	-2.20906600	-0.23348100	-0.58751100
C	-2.47574400	-0.01493200	0.76265300
C	-1.32157700	0.15190800	1.64782600
C	-1.30521300	0.40375900	3.02396500
H	-2.23112500	0.48612400	3.57966600
C	-0.07849400	0.55840200	3.66895700
H	-0.05040600	0.76428400	4.73459500
C	1.11275000	0.45004400	2.95590000
H	2.05275000	0.57239200	3.47356200
C	1.08618000	0.17762800	1.58017500
C	2.19471400	0.02372800	0.63240000
C	1.83117900	-0.18654000	-0.69478700
H	2.64588000	-0.24449000	-1.41979100
C	1.03718200	2.34275300	-3.00044600
C	0.63090700	2.53517100	-4.32305400
H	-0.42534800	2.55649200	-4.56587300
C	1.58257200	2.68061600	-5.33478900
H	1.25614300	2.82279000	-6.36064200
C	2.94350700	2.63659700	-5.03481900
H	3.68001400	2.75027200	-5.82482100
C	3.35671600	2.44850000	-3.71239100
H	4.41639400	2.42302900	-3.46971900
C	2.40895400	2.29774300	-2.70398000
H	2.72988400	2.15727100	-1.67717400
C	0.30835100	3.29349500	-0.39969400
C	1.06948000	4.42740800	-0.72902300
H	1.49153300	4.52528300	-1.72244900
C	1.26355600	5.44902100	0.20245300
H	1.84296400	6.32491700	-0.07561800
C	0.70725400	5.35389700	1.48021500
H	0.85590100	6.15298100	2.20092700
C	-0.04623800	4.22933700	1.81937700
H	-0.49588900	4.14159000	2.80393600
C	-0.24781400	3.21463500	0.88456400
H	-0.86889400	2.37429800	1.15258200
C	-1.77496000	2.71873500	-2.22791400
C	-2.14770800	4.04605500	-1.96436900
H	-1.45704500	4.71949400	-1.46997100
C	-3.41623700	4.50864000	-2.32019200
H	-3.69315600	5.53708400	-2.10517400
C	-4.32275300	3.65523700	-2.95114100
H	-5.31133300	4.01498400	-3.22276300
C	-3.94859400	2.34168600	-3.24063900
H	-4.64507100	1.67204900	-3.73573200

C	-2.68516600	1.87297100	-2.88173800
H	-2.38965100	0.85557000	-3.11109300
C	0.75262100	-3.57410300	-2.14933200
C	1.78980600	-2.95815200	-2.85906900
H	1.97047400	-1.90060200	-2.72199100
C	2.54269300	-3.69426900	-3.77455700
H	3.33285200	-3.20507100	-4.33612700
C	2.26626500	-5.04527700	-3.98857700
H	2.84933700	-5.61328800	-4.70783500
C	1.22811700	-5.66295700	-3.28739300
H	1.00288900	-6.71196200	-3.45628300
C	0.47198700	-4.93003500	-2.37397400
H	-0.33730100	-5.41183500	-1.83402100
C	0.49753800	-3.32767100	0.68837200
C	-0.07718900	-2.93997200	1.90920600
H	-0.88833700	-2.22564600	1.91239400
C	0.37333700	-3.46870800	3.11666100
H	-0.09352400	-3.15674500	4.04673100
C	1.42031900	-4.39341100	3.12527600
H	1.76177800	-4.82360500	4.06213400
C	2.02607000	-4.75794100	1.92265200
H	2.84795200	-5.46842500	1.92065900
C	1.57025900	-4.22937400	0.71247400
H	2.03757800	-4.53794000	-0.21537600
C	-1.86072900	-3.39968600	-0.99060000
C	-2.58786800	-3.14652200	-2.16711300
H	-2.14453900	-2.53131400	-2.94507000
C	-3.86636300	-3.67492500	-2.32660900
H	-4.41099900	-3.48583400	-3.24727400
C	-4.44827700	-4.43972500	-1.30998200
H	-5.44580000	-4.85162500	-1.43691200
C	-3.73844400	-4.67660900	-0.13320900
H	-4.18083100	-5.27052700	0.66176500
C	-2.44395900	-4.16977300	0.02155700
H	-1.89176000	-4.38983400	0.92815000
C	-4.65939100	-0.89382600	2.60112300
C	-5.93351500	-0.79110200	3.18267700
H	-6.59099500	0.03219900	2.91885100
C	-6.36263600	-1.76083500	4.08572400
H	-7.34674800	-1.68048100	4.53683100
C	-5.53322800	-2.84224600	4.39862300
H	-5.87399700	-3.59893100	5.09880800
C	-4.27710700	-2.95901100	3.80223400
H	-3.63861200	-3.80699900	4.03070700
C	-3.83870900	-1.98806400	2.90231500
H	-2.87589400	-2.08787700	2.41736600
C	-4.16497600	1.98559400	2.00804400
C	-3.66648300	2.99493500	1.16639000
H	-3.31005300	2.75486000	0.16981100
C	-3.61432100	4.31081900	1.61734100
H	-3.22449500	5.08162600	0.96076400
C	-4.04203400	4.62449300	2.91005800
H	-3.99393300	5.65012200	3.26354700
C	-4.52524300	3.62056000	3.75152900
H	-4.85048800	3.86309800	4.75857100
C	-4.58962400	2.29998100	3.30459100
H	-4.95403700	1.52207500	3.96661800
C	-5.29783500	0.15573400	-0.01105400
C	-5.96691000	1.26842500	-0.53318100
H	-5.79020800	2.25781000	-0.12865200
C	-6.86101800	1.10041400	-1.59097000
H	-7.38016500	1.96371800	-1.99544200
C	-7.08156000	-0.16771800	-2.12833600
H	-7.77672000	-0.29357800	-2.95302200
C	-6.41158100	-1.27800300	-1.60583900
H	-6.57626800	-2.26546700	-2.02354800
C	-5.52480900	-1.12357300	-0.54507600
H	-5.00664700	-1.98949100	-0.14616000
H	-3.08509500	-0.35039600	-1.23061000
P	3.90970500	0.34154400	1.06560400
C	4.38317300	-0.41289100	2.68921500
C	3.74265400	-1.79477500	2.92206100
C	5.89714200	-0.46271000	2.97141300
H	3.95721500	0.27408900	3.42797100
C	3.98569400	-2.23056600	4.37328300
H	4.18339500	-2.52782200	2.23829200
H	2.67273000	-1.77543100	2.70664700
C	6.13332500	-0.87810400	4.43093900
H	6.36358700	-1.20487700	2.31564300
H	6.37758000	0.49972100	2.76176000
C	5.47770500	-2.23274600	4.73032500
H	3.55387200	-3.22399000	4.52982700
H	3.44696700	-1.54545900	5.04444800
H	7.20977700	-0.92049400	4.63233100
H	5.71721100	-0.10897000	5.09724800
H	5.61595900	-2.49446700	5.78552700
H	5.98564100	-3.01196500	4.14421100
C	4.92569600	-0.35978200	-0.32157100
C	6.45202300	-0.12359600	-0.31491600
C	4.65185900	-1.86986000	-0.52274100
H	4.52862600	0.17141100	-1.19588300
C	7.03417200	-0.55812300	-1.67045700
H	6.91846500	-0.71855300	0.47458300
H	6.70599000	0.91790000	-0.12098700
C	5.22897400	-2.32270800	-1.87007200
H	5.13840000	-2.42767000	0.28733200
H	3.58551800	-2.09740800	-0.47176700
C	6.73077100	-2.03196100	-1.96746100
H	8.11585100	-0.38146700	-1.67399500
H	6.60852300	0.07364700	-2.46340200
H	5.02717800	-3.38938500	-2.01476800
H	4.69736400	-1.79611400	-2.67441600
H	7.10800600	-2.30375400	-2.95983400
H	7.26811500	-2.66101600	-1.24339800
C	4.05059200	2.21450700	1.17944500
C	4.19331600	2.75083300	2.61805600
C	5.09565800	2.87093500	0.25846600
H	3.06000100	2.51648600	0.81492400
C	4.06482300	4.28170700	2.63813900
H	5.16700600	2.45389600	3.02922600
H	3.42126000	2.32674600	3.26742700
C	4.91616900	4.39480800	0.25786100
H	6.10398900	2.63767800	0.61989100
H	5.01700700	2.48386700	-0.76337500
C	5.04593200	4.96168200	1.67638900
H	4.21960100	4.64282500	3.66175700
H	3.03875900	4.54567100	2.35787200
H	5.65944600	4.84933000	-0.40723300
H	3.92763300	4.63798200	-0.15066400
H	4.87446000	6.04431300	1.66998900

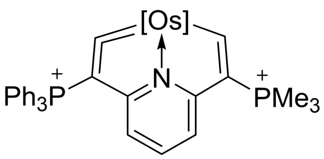

5a′

[Os] = $OsCl(PPh_3)_2$

E = -4522.849345 a.u.

Os	-0.97572600	-0.02235200	-0.65571500
Cl	-1.57081100	0.02168800	-2.96720000
P	-0.90886100	-2.44513400	-0.83759100
P	-1.12388000	2.40008000	-0.68007900
P	3.43073300	0.11730900	0.52231300
N	-0.49389100	-0.15345400	1.42009900
C	0.87698800	0.05680700	-0.53648700
C	1.66281100	0.00435200	0.58188500
C	0.82875600	-0.16354600	1.76971300
C	1.19608200	-0.31931300	3.11156800
H	2.24218700	-0.36508700	3.38818000
C	0.19569800	-0.43937400	4.07250300
H	0.46366700	-0.56797300	5.11645400
C	-1.14727000	-0.40226400	3.70068400
H	-1.91922000	-0.50243100	4.45315300
C	-1.47501100	-0.25744700	2.35040600
C	-2.79299500	-0.18588400	1.72141700
C	-2.77692400	-0.09583300	0.35695200
H	-3.72488500	-0.01996500	-0.17437000
C	-2.18543600	-3.21682500	-1.88947400
C	-1.92859400	-4.43885200	-2.52710800
H	-0.95030900	-4.90010900	-2.45124100
C	-2.92819300	-5.06339200	-3.27239700
H	-2.71820100	-6.00609100	-3.76789700
C	-4.18968900	-4.47753600	-3.38599200
H	-4.96403600	-4.96325800	-3.97152300
C	-4.45008700	-3.25897100	-2.75592400
H	-5.42440300	-2.79058000	-2.85891000
C	-3.45161100	-2.62834000	-2.01637200
H	-3.64656100	-1.66634500	-1.56075800
C	-1.12439700	-3.24912200	0.79560000
C	-2.35573400	-3.80464100	1.16684700
H	-3.17182300	-3.83392400	0.45224100
C	-2.52409900	-4.35349600	2.44031200
H	-3.47176200	-4.81337400	2.70669200
C	-1.47154700	-4.34368500	3.35702800
H	-1.60130400	-4.78295600	4.34163100
C	-0.24081700	-3.79283200	2.99070000
H	0.58950400	-3.79200800	3.69047500
C	-0.06770100	-3.25252000	1.71926100
H	0.89642700	-2.84462100	1.44135000
C	0.68618500	-3.08528700	-1.46122600
C	1.22194300	-4.30272200	-1.01222700
H	0.70470500	-4.88163200	-0.25493600
C	2.42745100	-4.77346700	-1.53608200
H	2.83618200	-5.71564600	-1.18307200
C	3.09695500	-4.04046000	-2.51809000
H	4.03452300	-4.40707200	-2.92488400
C	2.55250300	-2.84289100	-2.98562700
H	3.06419000	-2.27394900	-3.75328000
C	1.35460000	-2.36204600	-2.46065800
H	0.93773200	-1.42999500	-2.82472900
C	-2.48447800	3.15901800	-1.62908600
C	-3.64297500	2.44446800	-1.95908900
H	-3.70248100	1.38482500	-1.75292600
C	-4.69955400	3.08201500	-2.60978400
H	-5.58607200	2.51625200	-2.88016200
C	-4.60657800	4.43521300	-2.93749600
H	-5.42618400	4.92792400	-3.45127400
C	-3.45153000	5.15268900	-2.61636800
H	-3.37190500	6.20352800	-2.87658000
C	-2.39408200	4.51972000	-1.96649000
H	-1.49865100	5.08212700	-1.72162400
C	-1.33119600	3.10555400	1.00155000
C	-0.35878900	2.86131700	1.98330100
H	0.53211700	2.30612000	1.72606900
C	-0.52017500	3.33710300	3.28249300
H	0.24524100	3.13911800	4.02739200
C	-1.66015800	4.06892800	3.62292700
H	-1.78280100	4.45244700	4.63132500
C	-2.63314900	4.31989100	2.65340000
H	-3.51113600	4.90888200	2.90348700
C	-2.47514400	3.83780300	1.35116900
H	-3.23240100	4.05086400	0.60543400
C	0.37825900	3.16358300	-1.40020400
C	0.55468000	3.02130500	-2.78811000
H	-0.18957600	2.49586900	-3.37771500
C	1.68220500	3.55837600	-3.40572200
H	1.79692800	3.46802700	-4.48190700
C	2.65861600	4.21157000	-2.64667200
H	3.53899300	4.62299800	-3.13075200
C	2.49299900	4.34027000	-1.26822400
H	3.24326300	4.84703800	-0.67003500
C	1.35050900	3.83031200	-0.64571200
H	1.22397600	3.97381200	0.41983600
C	4.00904100	1.71765400	1.13692100
C	5.11392700	2.33068800	0.52793300
H	5.62747800	1.84140100	-0.29260100
C	5.54077400	3.58353600	0.96805000
H	6.39646300	4.05573500	0.49611000
C	4.86497200	4.23082800	2.00424000
H	5.19581700	5.20889500	2.33929200
C	3.76417200	3.62074800	2.61303900
H	3.23898500	4.12366500	3.41902700
C	3.33711000	2.36615700	2.18551300
H	2.48549000	1.89791900	2.66488100
C	4.10376300	-1.22752700	1.51416200
C	3.67867000	-2.53201800	1.20666500
H	3.00656500	-2.70980300	0.37260300
C	4.14167600	-3.60012400	1.96891500
H	3.82128100	-4.60910900	1.72882000
C	5.01995300	-3.37168000	3.03432000
H	5.37857500	-4.20711700	3.62753100
C	5.44453300	-2.07578600	3.33377600
H	6.13237400	-1.90385200	4.15543700
C	4.99019500	-0.99644000	2.57305400
H	5.31965800	0.01225700	2.80133900
C	3.98416200	-0.04693100	-1.18136900
C	5.05950100	-0.89145000	-1.49083300
H	5.51686400	-1.50458700	-0.72220800
C	5.54062100	-0.93966600	-2.79917400
H	6.37699700	-1.58775000	-3.04071400
C	4.94664800	-0.15836600	-3.79249100

H	5.32404000	-0.19839900	-4.80973100
C	3.87129700	0.67905900	-3.48065000
H	3.41013200	1.29033000	-4.24890700
C	3.39186900	0.74864600	-2.17533700
H	2.57300800	1.41614400	-1.93926900
P	-4.30131900	-0.12709900	2.66436200
C	-4.59861900	-1.66992900	3.58178500
H	-5.49072200	-1.57282900	4.20781200
H	-4.73444700	-2.48453300	2.86638600
H	-3.73632200	-1.91293300	4.20678000
C	-4.28449600	1.26103000	3.83909000
H	-4.10770200	2.18752300	3.28570300

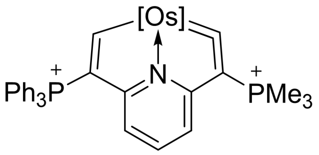

5a

[Os] = $OsCl(PPh_3)_2$

E = -4522.854279 a.u.

Os	-1.03468100	-0.01168700	-0.57999000
Cl	-1.43167600	0.03299800	-2.92904800
P	-1.18847100	2.40668400	-0.65027400
P	-1.01108300	-2.42964600	-0.75589400
P	3.55788300	0.09016100	0.42617100
P	-4.13118600	-0.08873800	2.74254400
N	-0.34086300	-0.08432900	1.43088300
C	1.01109600	0.09427100	-0.67100200
H	1.48767700	0.18941100	-1.64404400
C	1.76804100	0.05861000	0.46193200
C	0.99456000	-0.06613000	1.69594800
C	1.44605500	-0.13608300	3.01661800
H	2.50682400	-0.13757200	3.23227900
C	0.50995300	-0.20590100	4.05042600
H	0.84856500	-0.25906700	5.08036200
C	-0.85179000	-0.20736600	3.76691500
H	-1.58046100	-0.25933700	4.56721400
C	-1.26656000	-0.14978000	2.43199700
C	-2.60804300	-0.13753600	1.86565800
C	-2.56205200	-0.08941300	0.49525500
C	-2.55579300	3.02922200	-1.68175400
C	-2.47950500	4.30625900	-2.25756100
H	-1.58051700	4.90323200	-2.14518400
C	-3.56075500	4.81332500	-2.97767400
H	-3.49408100	5.80028500	-3.42467200
C	-4.72235200	4.05282800	-3.12557000
H	-5.56017100	4.44698100	-3.69235400
C	-4.80245300	2.78233200	-2.55132400
H	-5.69971600	2.18412600	-2.67936600
C	-3.72401600	2.26844700	-1.83322400
H	-3.77445500	1.27351500	-1.40620100
C	-1.48776200	3.18726500	0.98565600
C	-2.60495700	4.00934400	1.19837900
H	-3.29120900	4.21341100	0.38498600
C	-2.82996600	4.58561900	2.45103200
H	-3.68896900	5.23455900	2.59454700
C	-1.94860500	4.34690800	3.50740300
H	-2.12217500	4.80374200	4.47700600
C	-0.83667200	3.52657300	3.30548800
H	-0.14259600	3.33325400	4.11815600
C	-0.61085500	2.95246300	2.05577300
H	0.25415000	2.31995900	1.91237500
C	0.31167400	3.20752200	-1.33387700
C	0.57417100	3.03222000	-2.70425400
H	-0.10909100	2.45302000	-3.31626900
C	1.69923000	3.61924900	-3.27996500
H	1.87623400	3.50327900	-4.34508500
C	2.58840900	4.36051200	-2.49505800
H	3.46253900	4.81973300	-2.94690900
C	2.34499800	4.51332000	-1.12964300
H	3.03207400	5.08246400	-0.51117000
C	1.20434800	3.95019000	-0.55184300
H	1.00742100	4.11352700	0.50084100
C	0.52951300	-3.15651600	-1.42597900
C	1.13640500	-2.53665500	-2.52987400
H	0.68982100	-1.64767300	-2.96211300
C	2.29093700	-3.07936800	-3.09021400
H	2.75200100	-2.59128800	-3.94223200
C	2.85101000	-4.24365900	-2.55897200
H	3.74996600	-4.66566200	-2.99816800
C	2.24018600	-4.87665200	-1.47534800
H	2.65724100	-5.79580900	-1.07459300
C	1.08057800	-4.33984900	-0.91131900
H	0.60810600	-4.84455600	-0.07608800
C	-1.22979700	-3.23549200	0.87552800
C	-2.43069300	-3.87683800	1.20668700
H	-3.22431300	-3.95069200	0.47105300
C	-2.59840700	-4.44563600	2.47169400
H	-3.52202300	-4.96696600	2.70778500
C	-1.57517000	-4.37256900	3.41873900
H	-1.70481200	-4.82452800	4.39754100
C	-0.37416800	-3.73772300	3.09215500
H	0.43360200	-3.68622200	3.81606200
C	-0.20201500	-3.17551500	1.82954000
H	0.74147100	-2.70228800	1.58499400
C	-2.35174900	-3.11476200	-1.78440200
C	-3.57453700	-2.43538900	-1.89000600
H	-3.69148800	-1.46616200	-1.41893200
C	-4.62302300	-2.99677000	-2.61664100
H	-5.56443600	-2.46272700	-2.70612400
C	-4.45740500	-4.23099600	-3.24900000
H	-5.27225000	-4.66131600	-3.82293400
C	-3.23965900	-4.90634500	-3.15174100
H	-3.10510800	-5.86258400	-3.64757100
C	-2.18826200	-4.35288000	-2.42176500
H	-1.24376300	-4.88213600	-2.35455300
C	4.13647500	-1.33602900	1.36484500
C	5.12863100	-1.22842300	2.34758400
H	5.58805400	-0.26893000	2.56172900
C	5.51718000	-2.36634000	3.05642500
H	6.28479800	-2.28838500	3.81962200
C	4.92255900	-3.60021100	2.78399400
H	5.23031200	-4.48136100	3.33830000
C	3.93565600	-3.70583200	1.79858300
H	3.47659200	-4.66489000	1.58057900
C	3.53665000	-2.57694300	1.08884800
H	2.77404400	-2.66174500	0.32127600
C	4.12897100	-0.02078100	-1.27689800
C	3.88889000	1.07391200	-2.12531900
H	3.38740200	1.96116300	-1.75468500
C	4.31070900	1.01936700	-3.44996900
H	4.11945800	1.86113500	-4.10712100

C	4.98900300	-0.10838600	-3.92411500
H	5.32883000	-0.14123500	-4.95463000
C	5.24070000	-1.18642300	-3.07421100
H	5.77634100	-2.05638900	-3.44061100
C	4.80564700	-1.15240900	-1.74901000
H	4.99364800	-1.99574100	-1.09484100
C	4.21214600	1.62795400	1.10741700
C	3.39649600	2.49893800	1.84219600
H	2.36074300	2.24859800	2.02998700
C	3.91930700	3.69735800	2.32485200
H	3.28489800	4.37255200	2.89073600
C	5.25253300	4.02954300	2.07551100
H	5.65743400	4.96423800	2.45063500
C	6.06748100	3.16437500	1.33956500
H	7.10140500	3.42682500	1.14018900
C	5.55137000	1.96667000	0.84981300
H	6.17943700	1.30845100	0.25703000
C	-4.25122500	1.40494500	3.77288200

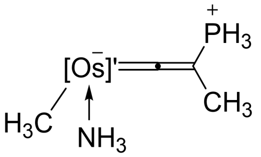

5a-1

[Os]' = $OsCl(PH_3)_2$

E = -1793.19369948 a.u.

P	-0.75365800	-2.35305000	0.04984700
P	-0.75326100	2.35304200	0.05495200
P	3.35237300	-0.00115800	0.83928400
C	2.56333500	0.00067100	-0.73725700
C	1.21672500	0.00059400	-0.61811500
H	4.20372700	1.09629100	1.08084300
H	4.20387200	-1.09906500	1.07823000
H	-1.47765100	-2.80433600	1.16967100
H	-1.38906000	-3.11507400	-0.96133600
H	0.42044000	-3.12571000	0.18908100
H	-1.47413500	2.80193000	1.17773700
H	0.42094300	3.12593000	0.19220300
H	-1.39190700	3.11667300	-0.95295900
H	2.35794300	-0.00239100	1.84256100
C	-2.72965400	-0.00079700	0.51523500
H	-2.77026500	-0.00903300	1.60882000
H	-3.29676800	0.88319700	0.18315500
H	-3.29919100	-0.87814900	0.16967300
H	-1.29682000	0.82125800	-2.70202600
H	-1.29628100	-0.81437000	-2.70415800
H	-2.54472000	0.00213400	-2.01018900
C	3.37054800	0.00223600	-2.02579400
H	2.69051200	0.00329100	-2.88224900
H	4.00878600	0.89027000	-2.10621600
H	4.00877200	-0.88560700	-2.10838700
Os	-0.59515300	0.00024900	-0.16468100
Cl	-0.01998400	-0.00254900	2.25065000
N	-1.53062200	0.00263300	-2.13934000

H	-3.42992700	1.42527500	4.49411600
H	-5.20461200	1.42690600	4.30947200
H	-4.15977800	2.28592300	3.13177000
C	-4.36344500	-1.53719800	3.81826100
H	-4.29990500	-2.44371600	3.21144800
H	-5.33442600	-1.48619900	4.32021500
H	-3.57172700	-1.57719800	4.57094200
C	-5.46515000	-0.05878300	1.51423000
H	-5.35202800	0.82141500	0.87590800
H	-5.40867600	-0.95634900	0.89282900
H	-6.43362300	-0.02340400	2.02133900
H	-5.23969700	1.31634200	4.36979100
H	-3.47565800	1.14100600	4.56280000
C	-5.70540200	0.12467200	1.54238000
H	-5.58227300	1.05930600	0.98864900
H	-5.78399200	-0.70816900	0.83879200
H	-6.62488100	0.17679900	2.13301500
H	6.07472100	4.80906800	2.03297300

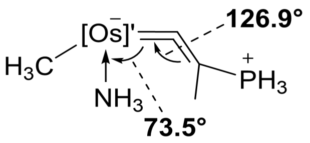

5a-2

[Os]' = $OsCl(PH_3)_2$

E = -1793.15810382 a.u.

P	0.09559400	2.25823000	-0.17482500
P	-1.72089100	-2.11307800	0.03192200
P	3.75869400	-0.78714000	-0.93474800
C	2.43914900	-0.14570000	0.07756500
C	1.24288900	-0.58230400	-0.43997400
H	4.66898800	-1.69912300	-0.35395900
H	4.64090300	0.18206200	-1.45983500
H	-0.83513500	3.02304500	-0.90358400
H	0.26234300	3.09456000	0.95480500
H	1.28377400	2.58467800	-0.86725000
H	-3.11136800	-2.05482500	0.25327900
H	-1.68873100	-2.79345300	-1.19894900
H	-1.39709800	-3.18011000	0.90682200
H	3.23596000	-1.46866100	-2.03831400
C	-1.85560700	0.86102300	1.59332800
H	-2.69606200	1.32422100	1.06305900
H	-2.26773800	0.08065300	2.24697600
H	-1.39352500	1.62106100	2.23403600
H	1.07768200	-1.51463900	1.77831400
H	0.98301500	-0.02520600	2.46114700
H	-0.26162400	-1.05456400	2.62192100
C	2.88966100	0.76934200	1.20907700
H	3.70990900	1.42231700	0.88585200
H	2.07949600	1.43186300	1.52621800
H	3.25335100	0.22250500	2.08926800
Os	-0.55543600	0.00432400	0.08440800
Cl	-1.92798300	0.46048800	-1.81289100
N	0.44959900	-0.73395600	1.96130800

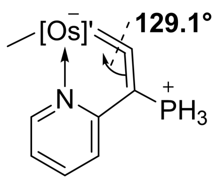

5a-3

[Os]' = $OsCl(PH_3)_2$

E= -1944.49807271 a.u.

Os	0.84686500	-0.03193400	0.00001200
Cl	3.05527000	0.85679200	-0.00015100
P	1.06588900	-0.09326000	2.37310000
P	1.06578200	-0.09351900	-2.37308000
P	-2.64062500	2.83620700	-0.00008000
N	-1.24779800	-0.93702600	0.00011700
C	-0.35257900	1.38163400	-0.00000200
C	-1.73513000	1.35268100	0.00003100
C	-2.25826000	-0.00902600	0.00001900
C	-3.60473300	-0.39205200	-0.00005700
H	-4.38728000	0.36209800	-0.00015000
C	-3.93530400	-1.74153200	-0.00001500
H	-4.97536200	-2.05266500	-0.00007200
C	-2.90604400	-2.68405500	0.00008800
H	-3.11082200	-3.74874300	0.00011600
C	-1.58883000	-2.24180200	0.00015100
C	1.43615700	-2.13392100	0.00004000
H	2.53113800	-2.05782300	-0.00003100
H	1.14238100	-2.70215000	0.88904900
H	1.14227000	-2.70222700	-0.88888500
H	-1.73222200	3.89977700	0.00096100
H	-3.49835800	3.06797700	-1.09630600
H	-3.49985400	3.06691300	1.09518200
H	1.91941900	-1.08795300	2.89130000
H	1.60216700	1.05076900	2.99374700
H	-0.07300800	-0.31043600	3.18079900
H	-0.07297500	-0.31177900	-3.18068700
H	1.60113600	1.05077800	-2.99402900
H	1.92007900	-1.08766200	-2.89105900
H	-0.77282500	-2.94913400	0.00022800

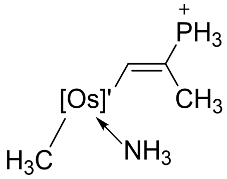

5a-4

[Os]' = $OsCl(PH_3)_2$

E = -1793.92170241 a.u.

Os	-0.62892200	0.07235700	-0.13493400
Cl	-0.78279200	-0.97515600	2.14246300
P	-1.32271800	-2.01240100	-0.87727300
P	-0.25001400	2.07701900	0.95183500
P	3.98725500	-0.83339100	0.17422300
C	2.58130500	0.07693700	-0.30352700
C	1.33415800	-0.53214700	-0.11124600
H	4.73759900	-0.47096800	1.32993200
H	5.06591300	-0.91920600	-0.74808100
H	-2.50995300	-2.54508600	-0.32156600
H	-1.63296900	-2.28910300	-2.24472000
H	-0.49159100	-3.14859200	-0.67693500
H	-1.20090400	2.48032700	1.92037000
H	0.91539000	2.31605200	1.72833400
H	-0.20974400	3.31428200	0.23283800
H	3.64766100	-2.16577300	0.44425600
C	-2.80585800	0.55968000	0.01318300
H	-3.03742600	1.47075900	0.58773000
H	-3.28654200	0.70474700	-0.97811400
H	-3.36173700	-0.24800600	0.50872100
H	-0.42660900	1.97133000	-2.12221600
H	-0.20540900	0.50453600	-2.82047400
H	-1.72121200	1.00379600	-2.39224900
C	2.83482700	1.49456400	-0.77921000
H	3.60059500	1.55050300	-1.56674600
H	1.90210800	1.88535600	-1.19634200
H	3.14512900	2.17779300	0.02424200
H	1.46124300	-1.57545900	0.22721600
N	-0.73863500	0.99980000	-2.10714700

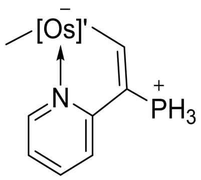

5a-5

[Os]' = $OsCl(PH_3)_2$

E = -1945.25244826 a.u.

Os	0.76843100	-0.04663800	-0.00112700
Cl	2.87626000	1.30529900	-0.02854000
P	1.18269500	-0.10172300	2.29630700
P	1.16098700	-0.31342900	-2.28396900
P	-2.91925500	2.63852400	-0.07192000
N	-1.13721400	-0.93570000	0.02918700
C	-0.38543000	1.57396600	-0.03778900
C	-1.78873100	1.32973000	-0.03009800
C	-2.20461200	-0.06278900	0.00905800
C	-3.52270700	-0.54590000	0.02383700
H	-4.35804200	0.15132200	0.00775000
C	-3.76844000	-1.91203700	0.05892200
H	-4.78730100	-2.28780800	0.07042400
C	-2.67897700	-2.78923700	0.07913200
H	-2.81727700	-3.86501200	0.10657400
C	-1.39498800	-2.26348200	0.06336000
C	1.94626900	-1.98803200	0.02558300
H	3.02116200	-1.76361200	0.06545500
H	1.71578700	-2.61711200	0.90207000
H	1.79032800	-2.64815700	-0.84554000
H	-2.19675900	3.83743900	-0.10861500
H	-3.82920700	2.76222800	-1.15665400
H	-3.83008400	2.82932300	1.00213300
H	2.33774000	-0.80621200	2.70304700
H	1.41314900	1.10205900	3.00363500
H	0.26617500	-0.68716800	3.21581800
H	0.52924100	-1.36231900	-3.00684700
H	0.92415800	0.71770200	-3.23055900
H	2.49868300	-0.59835300	-2.62832000
H	-0.07976600	2.63201500	-0.06985700
H	-0.51533100	-2.89588800	0.07771400

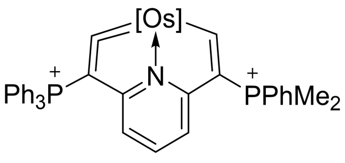

6a

[Os] = $OsCl(PPh_3)_2$

E =-4714.70543606 a.u.

Os	0.45185400	0.43257000	0.94776900
Cl	0.39509700	1.04042200	3.25566300
P	0.79848600	-1.83647400	1.74276000
P	0.09153300	2.79051000	0.46047500
P	-3.38354700	-0.81150300	-1.18861000
N	0.59547900	-0.16826400	-1.09360900
C	-1.27404000	0.01333900	0.39859500
C	-1.69240100	-0.43200800	-0.82597700
C	-0.55005100	-0.56957200	-1.72646500
C	-0.49436300	-1.02669600	-3.04810000
H	-1.39322000	-1.37374500	-3.54268800
C	0.73704700	-1.05022000	-3.70068000
H	0.79830500	-1.41451100	-4.72140200
C	1.89028500	-0.61330600	-3.05066500
H	2.85086100	-0.65029100	-3.54932500
C	1.79283300	-0.16400700	-1.73031900
C	2.84398400	0.35288100	-0.85551100
C	2.43695700	0.64752400	0.41642300
H	3.15946900	1.05600200	1.12217000
C	1.86281100	-1.94213700	3.21872000
C	1.44457300	-2.62630100	4.36560300
H	0.47025700	-3.10037300	4.39358000
C	2.27940500	-2.69243100	5.48273900
H	1.94482200	-3.21908800	6.37096900
C	3.53460500	-2.08617300	5.45980500
H	4.18054200	-2.13869400	6.33060500
C	3.95949100	-1.40743600	4.31403900
H	4.93642600	-0.93353600	4.29382900
C	3.12490100	-1.32869100	3.20297100
H	3.45388600	-0.79410900	2.31794100
C	1.55354500	-2.99039100	0.53262200
C	2.77487300	-3.63321300	0.77179100
H	3.32198400	-3.43995000	1.68721400
C	3.27728100	-4.55360600	-0.15273800
H	4.21337400	-5.06268000	0.05714300
C	2.57485300	-4.83040500	-1.32609500
H	2.96312800	-5.55290100	-2.03785400
C	1.35602800	-4.19092700	-1.57104400
H	0.79147000	-4.40935600	-2.47260600
C	0.84697300	-3.28623300	-0.64452300
H	-0.12025500	-2.83555300	-0.82181900
C	-0.76192700	-2.67457700	2.20062100
C	-0.94213200	-4.04965000	1.98601900
H	-0.15734900	-4.63860500	1.52482900
C	-2.13489600	-4.66763100	2.36617600
H	-2.26862900	-5.73130200	2.19298700
C	-3.14370200	-3.92288300	2.97960400
H	-4.07003400	-4.40435500	3.27796800
C	-2.95472900	-2.56082700	3.22092000
H	-3.73205400	-1.98013500	3.70371800
C	-1.77476400	-1.93291300	2.82823500
H	-1.63558400	-0.87407400	3.01288000
C	0.92155200	4.00754800	1.53782600

C	2.09873000	3.69051900	2.22788900
H	2.47066000	2.67533800	2.22705900
C	2.76394200	4.66694700	2.96894200
H	3.66475300	4.40692900	3.51662800
C	2.25842700	5.96631100	3.02989200
H	2.77245400	6.72322000	3.61415300
C	1.08239500	6.28877800	2.34875400
H	0.68141500	7.29629400	2.39870600
C	0.41540100	5.31608100	1.60619700
H	-0.49736000	5.57442500	1.07934300
C	0.60347000	3.30691700	-1.22829300
C	0.10685000	2.61926400	-2.34617000
H	-0.58830100	1.80458900	-2.20592800
C	0.48055900	2.98658300	-3.63738200
H	0.08249100	2.44047600	-4.48788300
C	1.35786000	4.05539300	-3.83398400
H	1.64174100	4.35249000	-4.83900300
C	1.85782600	4.74799500	-2.72971800
H	2.52876200	5.58990400	-2.87356900
C	1.48992400	4.37516200	-1.43422300
H	1.87913300	4.92949700	-0.58843400
C	-1.68425900	3.22344600	0.60527700
C	-2.22354700	3.30271000	1.90116500
H	-1.58721200	3.13540700	2.76416300
C	-3.57284200	3.60402300	2.07646200
H	-3.97488300	3.69269700	3.08148000
C	-4.40402500	3.79267700	0.96747600
H	-5.45614500	4.02025700	1.10859900
C	-3.87590200	3.69159700	-0.31921200
H	-4.51230200	3.83208400	-1.18712800
C	-2.51720000	3.42165600	-0.50187500
H	-2.11853300	3.38308100	-1.50780200
C	-4.11655000	0.42893200	-2.28261800
C	-5.48309000	0.72513500	-2.16768100
H	-6.09695500	0.20293700	-1.44130700
C	-6.04806200	1.71097200	-2.97612300
H	-7.10510800	1.93972800	-2.88569200
C	-5.25485500	2.40779500	-3.89042700
H	-5.69688600	3.17944300	-4.51295500
C	-3.89310300	2.11407000	-4.00540100
H	-3.27576200	2.65745100	-4.71394400
C	-3.32283400	1.12614700	-3.20624500
H	-2.26636900	0.90494800	-3.29839000
C	-3.42900400	-2.44262300	-1.95405700
C	-2.81339200	-3.50058600	-1.26216600
H	-2.37668700	-3.33759800	-0.28125800
C	-2.78126700	-4.76750200	-1.83720100
H	-2.31051600	-5.58715700	-1.30341800
C	-3.35706700	-4.98098400	-3.09464900
H	-3.32970600	-5.97041300	-3.54051000
C	-3.97391400	-3.93011300	-3.77616800
H	-4.42658500	-4.10158800	-4.74751400
C	-4.01420300	-2.65516000	-3.20824100
H	-4.49269000	-1.83551300	-3.73489700
C	-4.34284200	-0.82464000	0.33277600
C	-5.23862800	-1.87141200	0.59069300
H	-5.29743200	-2.72316700	-0.07795100
C	-6.05996500	-1.81039300	1.71657200
H	-6.75931400	-2.61606400	1.91581000
C	-5.98283500	-0.71646200	2.58068200
H	-6.62690200	-0.67070400	3.45360300
C	-5.08145300	0.32120200	2.32453300

H	-5.01925500	1.17281000	2.99352000
C	-4.26452400	0.27822000	1.19818200
H	-3.58322600	1.09458100	0.99641900
P	4.45509500	0.79112600	-1.47583800
C	4.27876100	2.08721700	-2.73935100
H	5.25370400	2.38479600	-3.13411600
H	3.65124100	1.72279400	-3.55544400
H	3.78402500	2.95064100	-2.28744900
C	5.44749500	1.49157800	-0.12341100
H	5.56949600	0.75186000	0.67178400
H	6.43519700	1.74879300	-0.51674100
H	4.97143300	2.39188000	0.27490700
C	5.31948200	-0.61983000	-2.18317300
C	4.89636100	-1.92147400	-1.87279100
C	6.43509600	-0.41509500	-3.01333900
C	5.59141000	-3.00964300	-2.39751400
H	4.03364500	-2.08649600	-1.23599900
C	7.12325300	-1.51066800	-3.52797800
H	6.77132900	0.58780600	-3.25983000
C	6.70018600	-2.80692800	-3.22102200
H	5.26113400	-4.01479700	-2.16204300
H	7.98527600	-1.35426300	-4.16823200
H	7.23709900	-3.65891200	-3.62616000

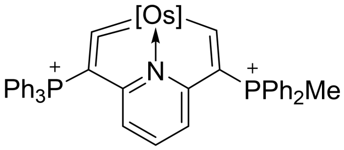

6b

[Os] = $OsCl(PPh_3)_2$

E = -4906.561111 a.u.

Os	0.12892100	0.05866800	1.12458100
Cl	-0.04829100	0.00976400	3.50760200
P	0.34929900	-2.36045900	1.28006000
P	-0.16689900	2.46693700	1.32853100
P	-3.51250200	-0.34032400	-1.58318400
N	0.45492400	0.08183700	-0.97937000
C	-1.54797200	-0.10248900	0.33611300
C	-1.85425100	-0.14027400	-0.99742500
C	-0.63488200	-0.04858200	-1.79919900
C	-0.46317700	-0.10081100	-3.18618800
H	-1.31813400	-0.23850200	-3.83672800
C	0.82708100	-0.00714300	-3.70696700
H	0.97815700	-0.05285300	-4.78096000
C	1.92469000	0.14072600	-2.86141100
H	2.92514600	0.22011100	-3.26564400
C	1.71404500	0.18584400	-1.47922000
C	2.69115700	0.31682300	-0.39888700
C	2.15716900	0.25848500	0.85869600
H	2.81217700	0.31614700	1.72700400
C	1.41036700	-2.83795700	2.68213900
C	0.87889100	-3.43985700	3.82642300
H	-0.17883800	-3.67223900	3.87760100
C	1.70954000	-3.73277600	4.90987800
H	1.28972700	-4.19641800	5.79701300
C	3.06993900	-3.43280400	4.85453000
H	3.71275300	-3.66396900	5.69817900
C	3.60726600	-2.83981800	3.70779900
H	4.66968100	-2.61961600	3.65581100
C	2.78135300	-2.53692100	2.62870500
H	3.20543100	-2.09282800	1.73238800
C	1.04898600	-3.27514200	-0.15051000
C	2.00476700	-4.28842500	0.00986000
H	2.42297800	-4.49477100	0.98749100
C	2.40314800	-5.05936000	-1.08472100
H	3.12676300	-5.85597200	-0.93938100
C	1.86873100	-4.82004300	-2.35165100
H	2.18123800	-5.42303900	-3.19903900
C	0.91158400	-3.81549400	-2.51802000
H	0.46945100	-3.63007200	-3.49239400
C	0.49774500	-3.06162800	-1.42291400
H	-0.28576300	-2.33170200	-1.55476200
C	-1.26741000	-3.18628900	1.52053300
C	-1.53967300	-4.42994300	0.92972100
H	-0.78803600	-4.92644600	0.32682500
C	-2.78219600	-5.03794400	1.11631100
H	-2.98553800	-5.99804700	0.65147200
C	-3.75103200	-4.42164100	1.90900400
H	-4.71509700	-4.89803800	2.05888600
C	-3.47525900	-3.19508500	2.51575500
H	-4.22307900	-2.71391000	3.13558500
C	-2.24727400	-2.56918900	2.31454300
H	-2.04605200	-1.60983800	2.77582800
C	0.63492100	3.29376300	2.74392600
C	1.81517700	2.78773700	3.30330700
H	2.20490300	1.83525800	2.97239000
C	2.46722300	3.48412100	4.32002900
H	3.37206500	3.07507600	4.75935300
C	1.94417800	4.68984000	4.79002300
H	2.44741500	5.22692900	5.58801000
C	0.76600100	5.19899100	4.23976800
H	0.35241600	6.13373100	4.60529600
C	0.11293000	4.50668200	3.22115700
H	-0.80088400	4.91001500	2.79778200
C	0.39791800	3.45797300	-0.11125300
C	-0.08098600	3.14532300	-1.39285100
H	-0.76315200	2.31810000	-1.51841300
C	0.27937300	3.91225200	-2.49766700
H	-0.10543000	3.65621800	-3.48070400
C	1.12455600	5.01256200	-2.33718400
H	1.38637000	5.62774700	-3.19283500
C	1.62213600	5.32267100	-1.07068600
H	2.27867000	6.17730500	-0.93692300
C	1.26704700	4.54879900	0.03746600
H	1.64753400	4.81248100	1.01716700
C	-1.94431000	2.88165600	1.52943300
C	-2.54858100	2.55733400	2.75655800
H	-1.96124500	2.09775300	3.54456500
C	-3.89870300	2.83376600	2.96381900
H	-4.34949300	2.60551300	3.92524000
C	-4.66765700	3.40412400	1.94424900
H	-5.72029100	3.61354700	2.10829100
C	-4.07588400	3.70633400	0.71818900
H	-4.66321900	4.14499700	-0.08225900
C	-2.71594200	3.45880000	0.51366800
H	-2.26881100	3.73191600	-0.43372300
C	-4.15116600	1.20210500	-2.28027800
C	-5.50616800	1.52676000	-2.11824900
H	-6.17397400	0.84551900	-1.60188400
C	-5.98982800	2.74119400	-2.60454100
H	-7.03832100	2.99136700	-2.47814000
C	-5.12693700	3.63524000	-3.24184400

H	-5.50557100	4.58307300	-3.61148100
C	-3.77688500	3.31191300	-3.40612900
H	-3.10551600	4.00743600	-3.89992700
C	-3.28792400	2.09792100	-2.93048700
H	-2.24058200	1.85309400	-3.06085200
C	-3.50551900	-1.64860500	-2.82305500
C	-2.93407400	-2.88291000	-2.46622200
H	-2.56116800	-3.04634400	-1.45905900
C	-2.86134000	-3.90232600	-3.41108200
H	-2.42192400	-4.85672900	-3.13854200
C	-3.35297000	-3.69504000	-4.70466800
H	-3.29371900	-4.49216900	-5.43924700
C	-3.92593600	-2.47090300	-5.05444300
H	-4.31294900	-2.31656000	-6.05655100
C	-4.00592000	-1.44167600	-4.11454500
H	-4.44904600	-0.48792600	-4.38300200
C	-4.58086500	-0.79604900	-0.20945700
C	-5.45262600	-1.88743200	-0.32170100
H	-5.43358400	-2.51929500	-1.20272000
C	-6.35438300	-2.15432400	0.70870500
H	-7.03511300	-2.99510400	0.62205400
C	-6.38458200	-1.33882100	1.84093400
H	-7.09397500	-1.54509300	2.63669700
C	-5.50641100	-0.25684000	1.95357600
H	-5.52611900	0.37842100	2.83254900
C	-4.60543300	0.02405100	0.93076100
H	-3.94176300	0.87470800	1.01636000
P	4.44348600	0.54584900	-0.64661600
C	5.12077000	1.32887000	0.85315400
H	6.16135400	1.60822300	0.67146800
H	4.54584800	2.23115800	1.07727200
H	5.07594500	0.64139800	1.70115500
C	4.75020500	1.65516500	-2.03498100
C	5.68411100	1.32451400	-3.02798700
C	4.04261900	2.86692100	-2.09655100
C	5.91688300	2.21369700	-4.07698100
H	6.22103600	0.38320900	-2.98569100
C	4.28976000	3.74790700	-3.14548300
H	3.30042800	3.11830100	-1.34456500
C	5.22418700	3.42407900	-4.13327200
H	6.63941500	1.96091900	-4.84625400
H	3.74849400	4.68569600	-3.19224700
H	5.41078200	4.11607600	-4.94860400
C	5.30525700	-1.01065300	-0.91935400
C	6.69339300	-1.08346900	-0.70780400
C	4.60285200	-2.12664000	-1.39964900
C	7.37386700	-2.26740000	-0.98025700
H	7.24535700	-0.22420400	-0.33967200
C	5.29819000	-3.30275800	-1.67836800
H	3.52918300	-2.08455500	-1.55204100
C	6.67631200	-3.37463200	-1.47026300
H	8.44449600	-2.32519200	-0.81280800
H	4.75638700	-4.16199300	-2.05432400
H	7.20953400	-4.29474000	-1.68798900

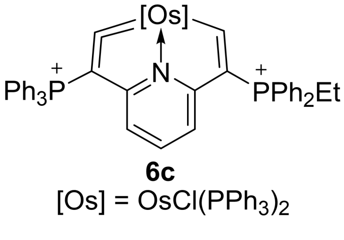

6c

$[Os] = OsCl(PPh_3)_2$

E = -4945.90489658 a.u.

Os	0.04943200	0.09521400	-1.18337500
Cl	0.00519800	0.23543300	-3.57440500
P	-0.11775000	2.52102300	-1.23699900
P	0.27077400	-2.31344200	-1.32565100
P	-3.66755300	-0.30076700	1.43525500
N	0.32803900	0.02433600	0.91822200
C	-1.64551700	-0.12177900	-0.43963900
C	-1.98104800	-0.23528700	0.88367800
C	-0.77524600	-0.14903400	1.70923000
C	-0.63155500	-0.16873500	3.09843100
H	-1.49490200	-0.30668900	3.73193300
C	0.63502400	-0.00092500	3.65154200
H	0.75525100	-0.00040200	4.73033000
C	1.74859300	0.16347400	2.83014300
H	2.73209400	0.31034500	3.25516800
C	1.57305000	0.15355400	1.44573100
C	2.58355400	0.26080700	0.39095000
C	2.07738400	0.25747000	-0.87618300
H	2.74120700	0.30211700	-1.73573900
C	0.77888000	3.25444900	-2.64466000
C	0.09738200	3.90314000	-3.67911000
H	-0.98083000	4.00844100	-3.63912600
C	0.80418400	4.40722200	-4.77272600
H	0.26751200	4.90493800	-5.57428100
C	2.19015300	4.27259800	-4.83703300
H	2.73648200	4.66636600	-5.68836000
C	2.87674700	3.63165900	-3.80131100
H	3.95755800	3.53316700	-3.84491000
C	2.17450800	3.11888800	-2.71460600
H	2.71051600	2.62690800	-1.90837500
C	0.50066900	3.43766700	0.23155900
C	1.36629800	4.53514100	0.11771600
H	1.74628400	4.83114200	-0.85260800
C	1.72137600	5.27357300	1.24971400
H	2.37933800	6.13083200	1.14239000
C	1.22087800	4.92524300	2.50501800
H	1.48835000	5.50877600	3.38107100
C	0.35961700	3.83180300	2.62673400
H	-0.04339300	3.55423300	3.59599100
C	-0.00041500	3.09999200	1.49856300
H	-0.69538500	2.28010100	1.60513100
C	-1.84455000	3.11315100	-1.37753300
C	-2.22265600	4.35569300	-0.84467900
H	-1.48954000	4.98170100	-0.34741500
C	-3.54592000	4.78573300	-0.94396400
H	-3.83267600	5.74636000	-0.52670700
C	-4.49681800	3.98570700	-1.58138000
H	-5.52725400	4.32056200	-1.65272400
C	-4.11829700	2.76282300	-2.13810200
H	-4.84983800	2.14153500	-2.64321500
C	-2.79821300	2.32533700	-2.03936300
H	-2.50899100	1.37479200	-2.46989600
C	1.87560800	-2.92556400	-1.97648400
C	2.55616500	-2.18075500	-2.95230300
H	2.12657100	-1.25677900	-3.32253100
C	3.76085100	-2.64822900	-3.48124600
H	4.27259500	-2.06832700	-4.24387000
C	4.28739900	-3.87176400	-3.05910500
H	5.21686100	-4.24097200	-3.48181500
C	3.59875300	-4.63053500	-2.11013900
H	3.98771000	-5.59515300	-1.79722400

C	2.40386900	-4.15894200	-1.56605300
H	1.89312700	-4.74887500	-0.81310700
C	0.15894700	-3.02194900	0.35583000
C	-1.07507700	-3.44237400	0.87099100
H	-1.95026900	-3.46938800	0.23127100
C	-1.18099400	-3.84596300	2.20220500
H	-2.13825600	-4.17949700	2.58544700
C	-0.06125600	-3.82430700	3.03373600
H	-0.14733700	-4.14221700	4.06854500
C	1.17116400	-3.40351200	2.52914100
H	2.05155000	-3.38781200	3.16369700
C	1.27963000	-3.00225200	1.19997300
H	2.23974400	-2.68061300	0.81803000
C	-0.99673700	-3.22364800	-2.28146200
C	-1.89459900	-2.55447300	-3.12274000
H	-1.86040900	-1.47620900	-3.20133100
C	-2.80764500	-3.28391900	-3.88831300
H	-3.47426300	-2.76038800	-4.56736200
C	-2.84653800	-4.67534500	-3.80482200
H	-3.55565400	-5.23837300	-4.40392800
C	-1.96091600	-5.34611300	-2.95660300
H	-1.98446300	-6.42938200	-2.88908400
C	-1.03522900	-4.62641300	-2.20603200
H	-0.34690900	-5.15805100	-1.55740800
C	-4.04823500	-1.76508600	2.43756400
C	-4.64020200	-2.87921100	1.82182100
H	-4.90972900	-2.84652700	0.77248200
C	-4.90407600	-4.02940000	2.56463700
H	-5.36990000	-4.88325600	2.08316300
C	-4.58502900	-4.07683700	3.92340100
H	-4.79995900	-4.97034400	4.50105200
C	-4.00160400	-2.96864800	4.54231000
H	-3.76536500	-2.99636000	5.60129400
C	-3.73347700	-1.81700600	3.80541200
H	-3.31443500	-0.95318600	4.30837100
C	-4.03709600	1.19148400	2.38260900
C	-3.48677500	2.39531600	1.91340500
H	-2.85084500	2.39995800	1.03612700
C	-3.78760400	3.59153300	2.55781000
H	-3.35841000	4.51727700	2.18932500
C	-4.64332700	3.59455100	3.66272100
H	-4.87653700	4.52787600	4.16580400
C	-5.21316400	2.40171800	4.11391100
H	-5.89112300	2.40766300	4.96138800
C	-4.91809100	1.19727300	3.47467200
H	-5.37468100	0.27667400	3.82060900
C	-4.71901800	-0.31568400	-0.03102700
C	-5.87452900	0.47838900	-0.04297500
H	-6.10388500	1.12586900	0.79584100
C	-6.72798200	0.44158600	-1.14496100
H	-7.62319000	1.05517400	-1.15152400
C	-6.42934900	-0.37803400	-2.23481700
H	-7.09623600	-0.40600800	-3.09103300
C	-5.27573200	-1.16676700	-2.22389900
H	-5.04182600	-1.81065100	-3.06423700
C	-4.41936500	-1.14380200	-1.12555800
H	-3.52877900	-1.76112300	-1.13212100
P	4.33988700	0.14089400	0.70141500
C	5.21260200	0.29840500	-0.90234800
H	4.82088100	-0.47438100	-1.57202300
H	4.91826200	1.26912500	-1.31627300
C	4.73315700	-1.47836500	1.40287200
C	4.84455500	-1.66441500	2.78944000
C	4.82320100	-2.58230600	0.53846600
C	5.05344200	-2.94329400	3.30422900
H	4.78771100	-0.81712800	3.46355700
C	5.03369200	-3.85547300	1.06251200
H	4.71544500	-2.46555400	-0.53485600
C	5.14845000	-4.03817600	2.44240800
H	5.14961900	-3.08225900	4.37641900
H	5.10502200	-4.70233400	0.38914800
H	5.31585500	-5.03199200	2.84553000
C	4.89957800	1.44008800	1.81476000
C	6.14930900	1.33746100	2.45239300
C	4.11377700	2.59204200	1.98001600
C	6.60471900	2.38228700	3.25262800
H	6.75703800	0.44662100	2.33238500
C	4.58151500	3.62998600	2.78439700
H	3.14799300	2.67871300	1.49278300
C	5.82088400	3.52723500	3.41854200
H	7.56809400	2.30291000	3.74585400
H	3.97176000	4.51645300	2.91358300
H	6.17812100	4.33940500	4.04398000
C	6.73682100	0.19380600	-0.77267900
H	7.18878200	0.27905400	-1.76465700
H	7.14289500	0.99445600	-0.14923500
H	7.03815900	-0.76765900	-0.34699300

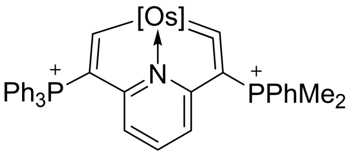

7a

[Os] = $OsCl(PPh_3)_2$

E = -4714.713362 a.u.

Os	-0.68217800	0.07068500	-0.57347700
Cl	-1.07116700	0.16264900	-2.92782400
P	-0.76957700	2.48778700	-0.59284400
P	-0.79418500	-2.33702400	-0.80988300
P	3.91223800	-0.05606100	0.41327500
P	-3.78268400	-0.08250600	2.73794900
N	0.01882600	-0.06423100	1.42874700
C	1.36737300	0.10428500	-0.68199000
H	1.84944700	0.19654000	-1.65293700
C	2.12581800	0.02151700	0.44845600
C	1.35561800	-0.09294000	1.68480400
C	1.81287700	-0.19956000	3.00103900
H	2.87436800	-0.23437000	3.21010100
C	0.88152300	-0.26052000	4.03981000
H	1.22495900	-0.34041800	5.06643900
C	-0.48141800	-0.21787300	3.76548400
H	-1.20689600	-0.25907800	4.56916500
C	-0.90297100	-0.12376500	2.43478700
C	-2.24602200	-0.06947100	1.87781400
C	-2.20220000	0.01795400	0.50926600
C	-2.08864700	3.17290200	-1.64862500
C	-1.96495100	4.46872100	-2.17294700
H	-1.06533500	5.04745600	-1.99080400
C	-2.99677600	5.01650000	-2.93391800
H	-2.89363900	6.01802000	-3.33967900
C	-4.15397300	4.27459000	-3.17995500
H	-4.95054500	4.69710300	-3.78467600

C	-4.28072300	2.98658100	-2.65599500
H	-5.16958900	2.39928300	-2.86497200
C	-3.25541300	2.43413300	-1.88964000
H	-3.34961300	1.42953300	-1.49611300
C	-1.09868400	3.22547600	1.05602500
C	-2.22973500	4.02820100	1.27010100
H	-2.89767400	4.25276900	0.44662100
C	-2.48971400	4.56094000	2.53493900
H	-3.35855300	5.19637600	2.68018700
C	-1.62855100	4.29763700	3.60220900
H	-1.82821000	4.72103400	4.58190900
C	-0.50302400	3.49664000	3.39880300
H	0.17528300	3.28545900	4.22017900
C	-0.24255700	2.96467800	2.13692000
H	0.63152900	2.34260400	1.99529800
C	0.76406800	3.27162400	-1.21750700
C	1.07035800	3.08842000	-2.57780100
H	0.40180400	2.51292700	-3.20983000
C	2.21927500	3.66237500	-3.11742200
H	2.43110700	3.54028800	-4.17545800
C	3.08914100	4.39754100	-2.30533300
H	3.98261700	4.84648900	-2.72883900
C	2.80011900	4.56025200	-0.95020200
H	3.47047500	5.12833200	-0.31274000
C	1.63475400	4.01091000	-0.40884500
H	1.40075900	4.18302800	0.63519100
C	0.69508200	-3.14109100	-1.50728600
C	1.36103200	-2.51780400	-2.57433900
H	0.98344400	-1.58229700	-2.97211700
C	2.48424800	-3.11530000	-3.14348900
H	2.99086200	-2.62426000	-3.96747500
C	2.95369400	-4.33777400	-2.65736600
H	3.82824300	-4.80226400	-3.10297200
C	2.28281900	-4.97317900	-1.61092800
H	2.62875900	-5.93583800	-1.24624200
C	1.15481600	-4.38152400	-1.03859000
H	0.63591300	-4.88671200	-0.23174200
C	-1.05212000	-3.19259400	0.79106000
C	-2.25492300	-3.85097300	1.07874900
H	-3.04317000	-3.88560800	0.33489500
C	-2.43158400	-4.48530600	2.31140900
H	-3.35619100	-5.01940600	2.51285700
C	-1.41651600	-4.45870100	3.26999200
H	-1.55378900	-4.95975600	4.22350500
C	-0.21505000	-3.80367300	2.98758700
H	0.58578900	-3.78662500	3.72075600
C	-0.03281300	-3.17892400	1.75610200
H	0.91339900	-2.69581900	1.54213800
C	-2.18636500	-2.88858200	-1.85190100
C	-3.40680500	-2.19648800	-1.80710400
H	-3.50007200	-1.30277800	-1.20055500
C	-4.49212700	-2.65223000	-2.55178000
H	-5.43089600	-2.10838000	-2.51508900
C	-4.36746800	-3.78832300	-3.35500600
H	-5.21132800	-4.13403200	-3.94425500
C	-3.15359800	-4.47469700	-3.40626600
H	-3.05064400	-5.35530500	-4.03262100
C	-2.06483000	-4.03009500	-2.65573000
H	-1.12455100	-4.56854900	-2.70251000
C	4.38918800	-1.56437000	1.28094400
C	5.37490700	-1.57737100	2.27589800
H	5.89702400	-0.66510600	2.54469500
C	5.67484700	-2.77382200	2.92904400
H	6.43723500	-2.78804800	3.70130600
C	4.99792500	-3.94736500	2.59063900
H	5.23665600	-4.87463000	3.10212400
C	4.01610900	-3.93279400	1.59496500
H	3.49093800	-4.84357000	1.32594300
C	3.70547100	-2.74453100	0.93999200
H	2.94345600	-2.73691400	0.16729200
C	4.48128500	-0.11643400	-1.29276000
C	4.33020000	1.04038000	-2.07650200
H	3.89585200	1.94066100	-1.65525200
C	4.75001100	1.02829500	-3.40290300
H	4.62845700	1.91839400	-4.01112000
C	5.33705400	-0.12105100	-3.94226900
H	5.67543500	-0.12189600	-4.97376800
C	5.49974200	-1.26286600	-3.15622000
H	5.96531500	-2.15027600	-3.57314500
C	5.06651600	-1.26976000	-1.83004700
H	5.18510300	-2.16109800	-1.22508100
C	4.65696800	1.40007100	1.17418800
C	3.86815000	2.34508800	1.84342600
H	2.79899300	2.20016300	1.92853000
C	4.46245000	3.48250500	2.38798800
H	3.85028700	4.21513400	2.90485300
C	5.83885100	3.68012800	2.26386900
H	6.29936300	4.56695900	2.68784800
C	6.62674300	2.74366700	1.58755600
H	7.69478600	2.90427600	1.48218500
C	6.04034400	1.60701400	1.03638000
H	6.65021400	0.89440900	0.48874000
C	-5.06692200	-0.09219100	1.48112600
C	-5.74726000	-1.27654600	1.16453500
H	-5.54814500	-2.19398600	1.70798800
C	-6.68724100	-1.27792500	0.13480000
H	-7.21742600	-2.19301500	-0.10788000
C	-6.94769900	-0.10447600	-0.57603600
H	-7.68706400	-0.10683700	-1.37093100
C	-6.26501700	1.07448900	-0.26441600
H	-6.46498800	1.98622700	-0.81660300
C	-5.32111100	1.08518400	0.75903400
H	-4.77973000	1.99973300	0.98009600
C	-3.95621200	1.38824300	3.79534600
H	-3.14593800	1.40388100	4.52971900
H	-4.92035300	1.37252000	4.31176300
H	-3.87119200	2.29026900	3.18541200
C	-3.95091400	-1.53628100	3.81687200
H	-3.73223700	-2.44397800	3.25041100
H	-4.95990700	-1.58005300	4.23685500
H	-3.22817700	-1.46597900	4.63397400

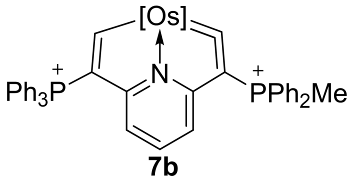

7b

[Os] = $OsCl(PPh_3)_2$

E = -4906.567456 a.u.

Os	0.20948500	-0.02158800	1.15268200
Cl	-0.21913600	0.17972800	3.49235100
P	-0.10744400	-2.40702900	1.44614700

P	0.43046900	2.39270500	1.15291100
P	-3.65277800	0.25632600	-1.51096300
N	0.32449200	-0.18808200	-0.96779200
C	-1.70818500	0.16417700	0.45550000
C	-1.98375400	0.09350100	-0.87764200
C	-0.80843700	-0.13251400	-1.72195600
C	-0.72873600	-0.27368200	-3.10964200
H	-1.62678700	-0.26133800	-3.71376400
C	0.52515000	-0.44262900	-3.70323100
H	0.59852700	-0.55352500	-4.78061600
C	1.67677500	-0.47122000	-2.92523500
H	2.65095900	-0.61217700	-3.37555900
C	1.55731500	-0.34602000	-1.53603000
C	2.58277200	-0.37223700	-0.50092200
C	2.01590700	-0.23733100	0.74061600
C	0.71362900	-3.08796900	2.92364300
C	0.18159100	-4.20950900	3.57502200
H	-0.75392000	-4.64367400	3.23825000
C	0.85331700	-4.76824900	4.66216300
H	0.43419500	-5.63318400	5.16678200
C	2.05690100	-4.21458200	5.10197800
H	2.57511800	-4.64833400	5.95163600
C	2.58867400	-3.09658800	4.45515000
H	3.51673700	-2.65598800	4.80734700
C	1.91919200	-2.52951200	3.37245900
H	2.31319300	-1.64517400	2.88427100
C	0.51521100	-3.44517300	0.06721600
C	1.50354700	-4.41699400	0.27239100
H	1.94793400	-4.53974000	1.25352500
C	1.89620000	-5.25327800	-0.77611000
H	2.65123600	-6.01334700	-0.60005200
C	1.31107800	-5.12500600	-2.03644000
H	1.60014800	-5.79258100	-2.84287100
C	0.34075700	-4.14263000	-2.25439600
H	-0.12100800	-4.03184900	-3.23110100
C	-0.05482000	-3.31216400	-1.20933400
H	-0.83539100	-2.58352300	-1.38139200
C	-1.86613500	-2.90846200	1.59860900
C	-2.38988100	-4.01869500	0.92097700
H	-1.75516800	-4.61722300	0.27786400
C	-3.73358200	-4.36889700	1.07836700
H	-4.12787400	-5.23334900	0.55228400
C	-4.55866000	-3.62310100	1.91952700
H	-5.60103500	-3.89989500	2.04448200
C	-4.03582400	-2.52924700	2.61362300
H	-4.67062800	-1.94959700	3.27567100
C	-2.69948500	-2.17118200	2.45667300
H	-2.29495600	-1.33201000	3.01196400
C	1.42370900	3.05428900	2.52997000
C	2.43797400	2.27581800	3.10475500
H	2.56617600	1.24722000	2.78765200
C	3.25505400	2.81683000	4.09623200
H	4.02896000	2.20549400	4.55125600
C	3.06343800	4.13240200	4.52368900
H	3.69442100	4.54822500	5.30322800
C	2.05229300	4.91025800	3.95581700
H	1.89765400	5.93186800	4.28875000
C	1.23392400	4.37563400	2.96138600
H	0.45062000	4.98520700	2.52273200
C	1.23679200	3.07980900	-0.34227700
C	0.70320000	2.80619900	-1.61057700
H	-0.19183400	2.20857600	-1.69332100

C	1.30320200	3.30704400	-2.76263100
H	0.87613600	3.07882200	-3.73511800
C	2.45091800	4.09573500	-2.66231300
H	2.92201000	4.49168600	-3.55659700
C	2.98641500	4.37741400	-1.40626100
H	3.87452100	4.99291600	-1.32067900
C	2.39183400	3.86840700	-0.25135700
H	2.82249800	4.09431800	0.71674400
C	-1.18934000	3.24725500	1.28842300
C	-1.87462500	3.16900500	2.51363700
H	-1.43177800	2.63670900	3.34812000
C	-3.11544500	3.78710500	2.66196500
H	-3.62278600	3.74271400	3.62117200
C	-3.69811500	4.46826000	1.58860300
H	-4.66346600	4.95098100	1.70833800
C	-3.03125300	4.52929000	0.36426600
H	-3.47604700	5.05010600	-0.47805700
C	-1.77746500	3.93149700	0.21665600
H	-1.25969400	4.02093300	-0.72980300
C	-3.89934400	1.85076500	-2.32750900
C	-5.17566600	2.43606000	-2.32083200
H	-6.00828600	1.93034400	-1.84348800
C	-5.36825800	3.68406700	-2.91185700
H	-6.35521300	4.13533600	-2.90429100
C	-4.29436000	4.35395000	-3.50287800
H	-4.44744100	5.32868000	-3.95549500
C	-3.02455800	3.77087000	-3.51417000
H	-2.18820800	4.29032100	-3.97139100
C	-2.82538100	2.52157700	-2.93170000
H	-1.83974000	2.07530900	-2.94728500
C	-3.94185800	-1.10629100	-2.65444800
C	-3.56995300	-2.39788100	-2.24284200
H	-3.15659100	-2.56206900	-1.25237300
C	-3.75094000	-3.47251000	-3.10895500
H	-3.46758600	-4.47150300	-2.79227600
C	-4.29528000	-3.26274500	-4.38040400
H	-4.43266100	-4.10214900	-5.05490900
C	-4.66736700	-1.97921500	-4.78555900
H	-5.09395100	-1.82118700	-5.77085700
C	-4.49355200	-0.89466600	-3.92430200
H	-4.77786400	0.10461500	-4.23829200
C	-4.82683700	0.17237000	-0.14795700
C	-5.74148700	-0.88303800	-0.04438600
H	-5.74473700	-1.68362400	-0.77494300
C	-6.65690500	-0.89630400	1.00843500
H	-7.37482700	-1.70662700	1.08386600
C	-6.64989400	0.12520700	1.95882100
H	-7.36451200	0.10898900	2.77597700
C	-5.73265600	1.17621800	1.85575000
H	-5.72775900	1.97616400	2.58885200
C	-4.82832400	1.21176200	0.79912900
H	-4.14473800	2.04764700	0.70494400
H	-2.50770000	0.32234600	1.17490600
P	4.34079000	-0.40421100	-0.67153600
C	5.07015200	1.16330900	-1.20260400
C	5.10323600	1.49180100	-2.56735900
C	5.61658500	2.04645900	-0.25892300
C	5.70671800	2.67638600	-2.98227500
H	4.68866400	0.81318400	-3.30555200
C	6.22433800	3.22720700	-0.68293100
H	5.58427700	1.81697900	0.80068500
C	6.27893000	3.53733500	-2.04290000

H	5.74451500	2.91949500	-4.03939600
H	6.66369700	3.89787900	0.04864300
H	6.76686600	4.44991000	-2.37121400
C	4.87288700	-1.67966100	-1.82844100
C	6.15249600	-1.61465600	-2.40615800
C	4.03688400	-2.77829700	-2.08008100
C	6.58663900	-2.64334900	-3.23867000
H	6.80075400	-0.76593100	-2.21419800
C	4.48125800	-3.79820700	-2.91949800
H	3.05263900	-2.83706800	-1.62919200
C	5.75006300	-3.73198000	-3.49834800
H	7.57431500	-2.59435300	-3.68563600
H	3.83267600	-4.64348300	-3.11906500
H	6.08962700	-4.52970600	-4.15166900
C	4.98649300	-0.80322900	0.98259900
H	4.61733300	-0.08700400	1.72018600
H	4.63373100	-1.80144500	1.25292000
H	6.07902100	-0.79351600	0.95954100

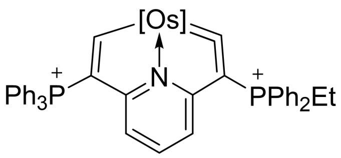

7c

[Os] = $OsCl(PPh_3)_2$

E = -4945.90533605 a.u.

Os	-0.12875600	0.08601600	-1.11953200
Cl	0.11663000	0.06470200	-3.49296000
P	0.00244800	2.51028100	-1.28194100
P	-0.31665000	-2.31974900	-1.33521700
N	-0.18055500	0.08653800	1.01089800
C	-1.92480200	0.21821300	-0.64096700
C	-2.45746300	0.25831100	0.61875400
C	-1.40040000	0.18178600	1.62325900
C	-1.48609500	0.21569900	3.01693000
H	-2.44982900	0.31321300	3.50024300
C	-0.30967100	0.14596000	3.76209000
H	-0.35544100	0.17072200	4.84636000
C	0.92672100	0.05062100	3.12565500
H	1.84047100	-0.00546000	3.70318200
C	0.97151500	0.02595200	1.72756800
C	2.13066200	-0.06448400	0.83992500
C	1.82132300	-0.06540900	-0.49172700
H	2.61492900	-0.17090600	-1.22951800
C	1.09623100	3.14552300	-2.59402700
C	0.71544200	4.24221400	-3.37749900
H	-0.26134200	4.69428900	-3.24426300
C	1.59264800	4.75513500	-4.33441100
H	1.28861500	5.60114300	-4.94282200
C	2.85255200	4.18249400	-4.51035700
H	3.53071300	4.58092400	-5.25856300
C	3.23698700	3.09060400	-3.72769200
H	4.21190400	2.63393600	-3.87149900
C	2.36217800	2.57109700	-2.77776500
H	2.65623700	1.71081100	-2.18975300
C	0.53346700	3.43336600	0.21309200
C	1.53786100	4.40916400	0.15142200
H	2.07273100	4.58065500	-0.77528300
C	1.83290900	5.18682000	1.27297500
H	2.60308800	5.94905600	1.20660700
C	1.13561500	4.99765500	2.46658200
H	1.35297900	5.61929900	3.33034900
C	0.14915200	4.01085200	2.54380000
H	-0.39860100	3.85130500	3.46800200
C	-0.15042700	3.23901400	1.42364000
H	-0.94390100	2.50857700	1.48431400
C	-1.65626600	3.20586600	-1.64908300
C	-2.20064100	4.27710800	-0.93028800
H	-1.62658500	4.75916100	-0.14741000
C	-3.49110200	4.73190800	-1.21628200
H	-3.90755800	5.55790000	-0.64772200
C	-4.23383000	4.13857100	-2.23633300
H	-5.23206900	4.49999900	-2.46418300
C	-3.68071200	3.08905100	-2.97818100
H	-4.24496400	2.63956100	-3.79024300
C	-2.40335200	2.61710400	-2.68344800
H	-1.97978200	1.79785900	-3.25477400
C	0.89153300	-3.03446100	-2.49810600
C	2.26464200	-2.92854400	-2.22360200
H	2.60642700	-2.46694800	-1.30270100
C	3.19959100	-3.42730400	-3.12644100
H	4.25725700	-3.34233700	-2.90247800
C	2.77621100	-4.02361600	-4.31716400
H	3.50656600	-4.40520800	-5.02398400
C	1.41429500	-4.12673800	-4.59596000
H	1.07966500	-4.58683900	-5.52040200
C	0.47245800	-3.63636600	-3.69027600
H	-0.58374900	-3.71524500	-3.91946800
C	-0.15278600	-3.28957900	0.21144400
C	-1.04917100	-3.00862100	1.25512300
H	-1.78970500	-2.23017600	1.13340800
C	-1.02954000	-3.75751900	2.42797600
H	-1.73576200	-3.52919500	3.21986200
C	-0.12517000	-4.81415700	2.56579600
H	-0.12089900	-5.41260300	3.47206200
C	0.75689000	-5.11231700	1.52609700
H	1.44515300	-5.94769900	1.61652400
C	0.75052300	-4.35105500	0.35366200
H	1.42791400	-4.60276300	-0.45424100
C	-1.95918000	-2.86232100	-1.94904000
C	-2.72929900	-2.02073600	-2.76560600
H	-2.35460900	-1.04087700	-3.03373700
C	-3.96732800	-2.45291700	-3.24541300
H	-4.55306700	-1.79888100	-3.88496800
C	-4.44380500	-3.72629800	-2.92229000
H	-5.40474300	-4.06103000	-3.30104300
C	-3.66984600	-4.57505300	-2.12760900
H	-4.02382300	-5.57423000	-1.89133400
C	-2.43404100	-4.14638800	-1.64193300
H	-1.84282200	-4.81167300	-1.02214800
P	3.81991700	-0.20160800	1.41604400
C	3.90985300	-1.45020800	2.71663000
C	3.09596600	-2.59182600	2.62611300
C	4.79530700	-1.29476000	3.79403200
C	3.18571300	-3.57548400	3.60806100
H	2.39389100	-2.70982100	1.80663000
C	4.87357000	-2.28552200	4.77144900
H	5.41158400	-0.40624100	3.87464400
C	4.07201900	-3.42472800	4.67757600
H	2.55457800	-4.45364100	3.53905600
H	5.55753100	-2.16546700	5.60543000
H	4.13439900	-4.19385800	5.44117200

C	4.47988000	1.35218600	2.04883300
C	5.87641500	1.51226600	2.10690500
C	3.63181200	2.34891900	2.55155800
C	6.41833200	2.65762900	2.68400400
H	6.53293800	0.75033400	1.69859700
C	4.18848900	3.48671700	3.13522400
H	2.55377200	2.24944700	2.48585200
C	5.57353800	3.64074100	3.20582300
H	7.49552900	2.78276500	2.72595600
H	3.53474400	4.25517800	3.52949700
H	5.99699500	4.53018100	3.66190000
C	4.85794700	-0.69423000	0.02431800
C	5.32824700	-2.00926700	-0.08612700
C	5.17843100	0.25908600	-0.95840100
C	6.11975400	-2.36765900	-1.17846800
H	5.09272600	-2.74123000	0.67903500
C	5.95502600	-0.11337600	-2.05237100
H	4.83559900	1.28484900	-0.85953800
C	6.42771400	-1.42485100	-2.16105800
H	6.50091600	-3.38105000	-1.25599400
H	6.20439800	0.62021400	-2.81239100
H	7.04483700	-1.70761100	-3.00811000
P	-4.19788500	0.15008100	0.88854000
C	-4.76267000	1.48528200	1.95819700
C	-5.95952700	1.38814600	2.68698900
C	-4.02866300	2.68227500	1.96362300
C	-6.40326100	2.47988600	3.43173800
H	-6.53773400	0.46988800	2.67636500
C	-4.48177900	3.76781400	2.70962200
H	-3.12371800	2.76721300	1.37254900
C	-5.66438900	3.66581700	3.44631500
H	-7.32600800	2.40564900	3.99808700
H	-3.91313700	4.69257200	2.71363700
H	-6.01472300	4.51228400	4.02866300
C	-4.58126900	-1.46033500	1.61273100
C	-4.64178400	-2.57285300	0.75619200
C	-4.65288400	-1.64167300	3.00254600
C	-4.78793900	-3.85004800	1.29086800
H	-4.56085900	-2.45535700	-0.31910400
C	-4.80036500	-2.92530800	3.52816500
H	-4.60953200	-0.78857100	3.67106900
C	-4.86857200	-4.02811400	2.67403500
H	-4.83560100	-4.70337600	0.62321000
H	-4.86864900	-3.06182100	4.60287200
H	-4.98635100	-5.02543300	3.08613000
C	-4.99820600	0.30732900	-0.74466600
H	-4.55854100	-0.45293400	-1.39763500
H	-4.68119700	1.28126900	-1.13369700
C	-6.52423100	0.19013400	-0.67851500
H	-6.95940600	0.96455100	-0.04009200
H	-6.94101700	0.31197200	-1.68219800
H	-6.83793700	-0.78736800	-0.30026900

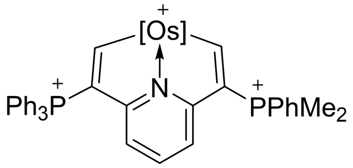

8a

[Os] = $OsCl(PPh_3)_2$

E =-4715.144817 a.u.

Os	0.51156500	0.49782200	0.97260000
Cl	0.36396200	1.18747600	3.16715800
P	0.86463000	-1.77547900	1.84970800
P	0.10856400	2.84848000	0.34075200
P	-3.43718700	-0.89547400	-1.16410600
N	0.55754500	-0.21702800	-1.09470100
C	-1.33910600	0.01937100	0.40994800
C	-1.72340500	-0.47347600	-0.78661100
C	-0.59303000	-0.63759900	-1.69296000
C	-0.57708100	-1.13745100	-2.99632500
H	-1.49188300	-1.49253200	-3.45301700
C	0.63340300	-1.18906500	-3.68722000
H	0.66402600	-1.58411600	-4.69740500
C	1.80008500	-0.73398800	-3.08050700
H	2.75018700	-0.77959800	-3.59805000
C	1.73358700	-0.24211900	-1.77518000
C	2.80650100	0.29783900	-0.95275700
C	2.37533000	0.63727900	0.28519000
H	3.01648100	1.06242500	1.05976500
C	1.94074600	-1.74057100	3.31537900
C	1.51417000	-2.26351900	4.54202200
H	0.53391000	-2.71654100	4.63436100
C	2.35487600	-2.20132300	5.65561300
H	2.01791500	-2.60711500	6.60402100
C	3.62055800	-1.62590700	5.55109700
H	4.27041300	-1.58004800	6.41900900
C	4.05543100	-1.11187500	4.32490600
H	5.04539400	-0.67407200	4.24062200
C	3.21770800	-1.16224700	3.21470700
H	3.56588900	-0.77508500	2.26100800
C	1.62576800	-2.96776000	0.69055100
C	2.80998100	-3.64687800	1.01163900
H	3.32582200	-3.43830200	1.94144200
C	3.31131000	-4.62698600	0.15029400
H	4.21565000	-5.16285900	0.42209300
C	2.64268700	-4.93332100	-1.03571200
H	3.02603300	-5.70683600	-1.69408800
C	1.46187800	-4.25904300	-1.36121000
H	0.92330900	-4.50354900	-2.27182000
C	0.95520000	-3.28905600	-0.50159400
H	0.01209600	-2.81637700	-0.73910800
C	-0.69917000	-2.56777200	2.36840600
C	-0.95472300	-3.91930700	2.09164800
H	-0.23321600	-4.51329800	1.54303000
C	-2.13623200	-4.51445300	2.54013600
H	-2.32184300	-5.56303200	2.32886300
C	-3.06064900	-3.77327600	3.27611000
H	-3.97177700	-4.24233000	3.63361600
C	-2.80183500	-2.43209400	3.57049200
H	-3.51077600	-1.85495400	4.15407500
C	-1.63145700	-1.82923800	3.11785300
H	-1.42761500	-0.79704800	3.38100500
C	0.89903200	4.08715500	1.41613000
C	2.11111800	3.82104000	2.06973000
H	2.55054100	2.83153700	2.03172300
C	2.74621700	4.81646400	2.81004800
H	3.67523700	4.59696000	3.32687200
C	2.17668900	6.08809100	2.90421700
H	2.66727100	6.86086000	3.48730700
C	0.97153000	6.36252400	2.25409900
H	0.52545400	7.34923200	2.32619800

C	0.33315900	5.36974800	1.51209200
H	-0.60078300	5.59480900	1.00844200
C	0.66165300	3.29428200	-1.34914000
C	0.21020300	2.57004300	-2.46401600
H	-0.47134400	1.74242200	-2.32844600
C	0.60445800	2.92595400	-3.75260700
H	0.23639500	2.36036400	-4.60366200
C	1.45706300	4.01541800	-3.94818300
H	1.75235500	4.30478800	-4.95195800
C	1.91390800	4.74117900	-2.84632500
H	2.56295000	5.59954300	-2.99078100
C	1.52429200	4.38368800	-1.55297000
H	1.87402500	4.96805700	-0.71018300
C	-1.68057600	3.22844400	0.43855000
C	-2.25504100	3.34246600	1.71769300
H	-1.64133000	3.22281200	2.60469100
C	-3.60656400	3.65708800	1.84967400
H	-4.03325500	3.78092200	2.84040500
C	-4.40336900	3.83190300	0.71301500
H	-5.45403400	4.08348300	0.81892000
C	-3.84181200	3.69125400	-0.55678000
H	-4.45272800	3.82166800	-1.44401400
C	-2.48239800	3.40113400	-0.69676600
H	-2.05548000	3.34025400	-1.68983500
C	-4.15079400	0.33105600	-2.27435500
C	-5.53408900	0.56917200	-2.21458100
H	-6.15514800	0.03255000	-1.50502600
C	-6.10940800	1.51188300	-3.06579900
H	-7.17826600	1.69378000	-3.02175400
C	-5.31289100	2.22065900	-3.96864400
H	-5.76505100	2.95468700	-4.62812800
C	-3.93587100	1.98473600	-4.02765600
H	-3.31882200	2.53617000	-4.73000400
C	-3.35280600	1.04129200	-3.18557100
H	-2.28570000	0.86382300	-3.23997000
C	-3.41233700	-2.53263900	-1.90474400
C	-2.70835800	-3.55438100	-1.24139700
H	-2.22556200	-3.36377900	-0.28727300
C	-2.65677100	-4.82573400	-1.80546600
H	-2.12112100	-5.62023700	-1.29538300
C	-3.30065700	-5.07946400	-3.02222500
H	-3.26014700	-6.07286500	-3.45774400
C	-4.00324300	-4.06446000	-3.67537000
H	-4.50852200	-4.26824200	-4.61377000
C	-4.06340500	-2.78494600	-3.12044300
H	-4.60908900	-1.99497800	-3.62643300
C	-4.39155300	-0.89935200	0.35863400
C	-5.03212700	-2.06409700	0.80234200
H	-4.92436600	-2.99568900	0.25883000
C	-5.82764100	-2.01288600	1.94777800
H	-6.34127700	-2.90779900	2.28369200
C	-5.96997000	-0.81666400	2.65280200
H	-6.59552400	-0.78270300	3.53938800
C	-5.32479200	0.34356400	2.21035100
H	-5.44723400	1.27798900	2.74810800
C	-4.54685700	0.31243500	1.05687200
H	-4.09996300	1.22739400	0.68533800
H	-2.01765000	0.20603500	1.24383100
P	4.44656200	0.67680600	-1.56479300
C	5.44794400	1.28058600	-0.17342800
H	6.45426000	1.49639000	-0.54508400
H	5.02058400	2.19605100	0.24627400
H	5.52278600	0.50852300	0.59712000
C	4.30803600	2.02179200	-2.77678100
H	5.29704900	2.32926000	-3.12737900
H	3.70438600	1.69764700	-3.62748000
H	3.80979900	2.87142600	-2.30258900
C	5.19641500	-0.77010700	-2.30941000
C	6.15214700	-0.61798600	-3.32989900
C	4.86711300	-2.04786100	-1.82433900
C	6.77842200	-1.74451700	-3.85837100
H	6.41309100	0.36333800	-3.71319800
C	5.50261700	-3.16386300	-2.36237300
H	4.12311400	-2.17354600	-1.04349500
C	6.45523000	-3.01456900	-3.37392800
H	7.51693800	-1.63084600	-4.64500800
H	5.25243100	-4.15090000	-1.99126900
H	6.94737300	-3.88943300	-3.78674400

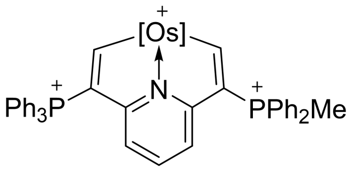

8b

[Os] = $OsCl(PPh_3)_2$

E =-4907.001437 a.u.

Os	0.15107800	0.08730500	1.21589300
Cl	-0.11687700	0.06981700	3.50773500
P	0.38771000	-2.35760500	1.32652800
P	-0.19366600	2.52311200	1.30589100
P	-3.57411700	-0.40956300	-1.59986200
N	0.41656200	-0.00387900	-0.95417400
C	-1.63027600	-0.11646800	0.35443800
C	-1.89128100	-0.22514500	-0.96603000
C	-0.66681600	-0.18091200	-1.76232600
C	-0.51244700	-0.29372700	-3.14440500
H	-1.37583700	-0.45706200	-3.77653200
C	0.76835000	-0.21985400	-3.69015500
H	0.90581400	-0.31077800	-4.76261700
C	1.86904700	-0.02909000	-2.86083100
H	2.86683600	0.05088500	-3.27103700
C	1.66735700	0.07757600	-1.48431800
C	2.65232800	0.30060400	-0.43336600
C	2.07642200	0.33713300	0.79089700
H	2.62312300	0.49214600	1.72111100
C	1.41458000	-2.89495400	2.72947300
C	0.94380900	-3.85528700	3.63400100
H	-0.03881100	-4.29420000	3.50486400
C	1.74037700	-4.24928500	4.71118900
H	1.36721600	-4.99143300	5.40950400
C	3.00688000	-3.69438500	4.89094700
H	3.62116800	-4.00168100	5.73113500
C	3.48428400	-2.73943600	3.98779400
H	4.47109800	-2.30770600	4.12719300
C	2.69024800	-2.33674700	2.91786200
H	3.06839700	-1.59909200	2.21724200
C	1.13415200	-3.05799500	-0.18432700
C	2.45103200	-3.53272000	-0.21197000
H	3.05123700	-3.54224300	0.68982400
C	2.98885200	-4.02912200	-1.40091200
H	4.00378900	-4.40676800	-1.40654300

C	2.22697800	-4.05226800	-2.56809200
H	2.64959100	-4.44856100	-3.48618500
C	0.90930900	-3.58654300	-2.54481100
H	0.29656500	-3.61776400	-3.44081600
C	0.36919300	-3.09453100	-1.36172100
H	-0.65838000	-2.75931800	-1.34680600
C	-1.22326100	-3.20084800	1.50896600
C	-1.47592600	-4.42424100	0.86825800
H	-0.72056100	-4.87898300	0.23789400
C	-2.69960500	-5.07142900	1.05174500
H	-2.88318500	-6.02086800	0.55823100
C	-3.67151500	-4.51377300	1.88378700
H	-4.61786300	-5.02434000	2.03161000
C	-3.41477000	-3.31024800	2.54426300
H	-4.15947100	-2.88100100	3.20560000
C	-2.20011200	-2.65476600	2.35858200
H	-2.00206800	-1.73847600	2.90326400
C	0.55676900	3.38205000	2.72533700
C	1.72817600	2.91003500	3.33417800
H	2.16179900	1.96519800	3.03135700
C	2.32974300	3.63413700	4.36202600
H	3.22765400	3.25330200	4.83854100
C	1.76664300	4.83752900	4.79128600
H	2.23084600	5.39743000	5.59681100
C	0.60118300	5.31637600	4.18872600
H	0.15977200	6.25046900	4.52102000
C	-0.00361000	4.59513700	3.16065200
H	-0.90766500	4.97686800	2.69845600
C	0.40136600	3.44027800	-0.16148500
C	-0.05710600	3.08413100	-1.43965800
H	-0.73184300	2.24865300	-1.55158600
C	0.30687200	3.82901900	-2.55877400
H	-0.06804200	3.55063900	-3.53951100
C	1.13969900	4.94217500	-2.41430600
H	1.39864400	5.54424400	-3.27999500
C	1.62700100	5.28460400	-1.15214000
H	2.27805700	6.14510100	-1.03455700
C	1.26234600	4.53970700	-0.02814800
H	1.62704100	4.83303200	0.94919000
C	-1.98434400	2.89635800	1.44915200
C	-2.61525400	2.60568300	2.67233000
H	-2.04589600	2.18169700	3.49306900
C	-3.96479300	2.90617000	2.85122400
H	-4.43377700	2.71145900	3.81098100
C	-4.70375900	3.47654200	1.80938900
H	-5.75192800	3.71869500	1.95488900
C	-4.08625700	3.74371000	0.58666800
H	-4.65077000	4.18566600	-0.22786600
C	-2.72935700	3.46345800	0.40664000
H	-2.26057400	3.71567600	-0.53584600
C	-4.13527000	1.14946500	-2.30966800
C	-5.49195900	1.49682500	-2.20336800
H	-6.19195700	0.83659500	-1.70302100
C	-5.93836000	2.70618400	-2.73530800
H	-6.98738600	2.97248200	-2.65660200
C	-5.03946800	3.57127700	-3.36360800
H	-5.39099900	4.51336000	-3.77231200
C	-3.68960800	3.22337500	-3.47478000
H	-2.99332500	3.89445700	-3.96750900
C	-3.23587600	2.01406000	-2.95498400
H	-2.19109500	1.74687800	-3.05669700
C	-3.54604900	-1.72350200	-2.82634200
C	-3.05924800	-2.98876600	-2.44944500
H	-2.76010000	-3.18356000	-1.42341600
C	-2.99103800	-4.00642000	-3.39696700
H	-2.62421500	-4.98672700	-3.10961200
C	-3.40559700	-3.76808000	-4.71256100
H	-3.35418600	-4.56518000	-5.44748000
C	-3.89780100	-2.51449700	-5.08180000
H	-4.23102700	-2.33794300	-6.09927300
C	-3.97178000	-1.48580900	-4.14103300
H	-4.35662000	-0.51200100	-4.42616400
C	-4.66786500	-0.83288500	-0.23517100
C	-5.38842100	-2.03532600	-0.23422000
H	-5.27416100	-2.75020000	-1.04037700
C	-6.27645700	-2.30237800	0.80845000
H	-6.84775800	-3.22490900	0.80154000
C	-6.43834100	-1.38473300	1.84739800
H	-7.13623900	-1.59549600	2.65177700
C	-5.71933300	-0.18471100	1.84461800
H	-5.85631600	0.53915800	2.64088600
C	-4.84406100	0.10302600	0.80146100
H	-4.33819600	1.06123700	0.77560500
H	-2.38934000	-0.14727300	1.13805800
P	4.43502500	0.43976200	-0.64260400
C	5.09572600	1.05175800	0.94086200
H	4.89953000	0.36627500	1.76845000
H	6.17704900	1.17064400	0.82998200
H	4.65202900	2.02759100	1.15593500
C	5.22560000	-1.13623300	-1.02005700
C	5.41697600	-1.53121200	-2.35470800
C	5.67904900	-1.95830600	0.02571300
C	6.08495800	-2.72124700	-2.63601600
H	5.08519000	-0.89820300	-3.17057400
C	6.34581700	-3.14674500	-0.26494600
H	5.53608200	-1.67393600	1.06285100
C	6.55899700	-3.52274000	-1.59420200
H	6.25103600	-3.01373800	-3.66774700
H	6.71421200	-3.76914700	0.54405000
H	7.09824900	-4.43801400	-1.81732100
C	4.83617500	1.63987900	-1.91623800
C	6.13274400	1.64005200	-2.46439500
C	3.91196900	2.63538100	-2.27391800
C	6.49263500	2.62445000	-3.38086600
H	6.85308200	0.87824400	-2.18441800
C	4.28620400	3.60978000	-3.19689400
H	2.91640500	2.65464800	-1.84334300
C	5.56835500	3.60508200	-3.75062200
H	7.49108900	2.62563600	-3.80550100
H	3.57402900	4.37512100	-3.48124100
H	5.85060900	4.36877800	-4.46837600

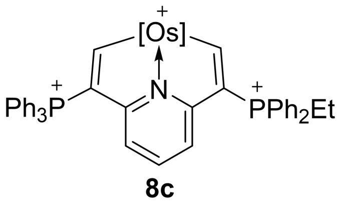

8c

[Os] = $OsCl(PPh_3)_2$

E = -4946.33836810 a.u.

Os	0.12269000	-0.02470400	1.17787800
Cl	-0.10463900	-0.19035100	3.47044100
P	0.29469100	-2.47486800	1.04193800

P	-0.06934600	2.41594400	1.43894600
P	-3.84621700	-0.17829700	-1.32189600
N	0.19662200	0.04979200	-1.00794200
C	-1.73889400	-0.09668500	0.47517300
C	-2.11329000	-0.09352600	-0.82027000
C	-0.96389400	-0.02978000	-1.71818700
C	-0.95181200	-0.03211300	-3.11238300
H	-1.87902000	-0.11382700	-3.66484400
C	0.27266500	0.04372000	-3.77138900
H	0.30709900	0.02755400	-4.85584500
C	1.45386800	0.13784000	-3.04387100
H	2.40359300	0.19478200	-3.55101200
C	1.39633800	0.15151300	-1.64826400
C	2.47731200	0.28825200	-0.67305300
C	2.01140500	0.22852300	0.59539000
H	2.64503200	0.33381300	1.47922700
C	1.40476900	-3.24365500	2.26834600
C	1.34393600	-4.63476200	2.45261800
H	0.60015000	-5.22414500	1.92658800
C	2.23575700	-5.26606300	3.31709200
H	2.17865400	-6.34065100	3.45768900
C	3.19332200	-4.51823600	4.00781600
H	3.87928100	-5.01196400	4.68885000
C	3.25459500	-3.13418600	3.83493600
H	3.98225400	-2.54682400	4.38654700
C	2.36259200	-2.49888800	2.96992600
H	2.39967700	-1.42141400	2.87229200
C	0.96219100	-3.00803600	-0.57419100
C	2.30737600	-3.37998000	-0.69392200
H	2.92949000	-3.45840400	0.18792300
C	2.84755900	-3.68328600	-1.94517500
H	3.89015300	-3.97733400	-2.01901400
C	2.04456900	-3.63969600	-3.08525500
H	2.45973700	-3.89511100	-4.05530000
C	0.69475500	-3.29455400	-2.96930500
H	0.05511200	-3.27963800	-3.84658100
C	0.15931200	-2.97433700	-1.72540700
H	-0.88743900	-2.71152800	-1.65116300
C	-1.31630200	-3.32137700	1.22200900
C	-1.64046400	-4.45417600	0.45851900
H	-0.94781800	-4.83144300	-0.28518900
C	-2.85675300	-5.11055900	0.66027200
H	-3.09537600	-5.99102900	0.07144800
C	-3.75113600	-4.65030200	1.62757100
H	-4.69159900	-5.16812200	1.78770700
C	-3.42333800	-3.53664600	2.40444800
H	-4.10733600	-3.18419100	3.16884900
C	-2.21445400	-2.87435800	2.20519600
H	-1.95873400	-2.03083100	2.83686200
C	0.78163500	3.11625900	2.88940100
C	1.92063800	2.50917300	3.43781000
H	2.26114800	1.54788600	3.07335700
C	2.60951900	3.11774200	4.48555600
H	3.48096800	2.63255300	4.91402400
C	2.16653200	4.33947900	4.99642900
H	2.69808400	4.80946500	5.81765900
C	1.03416500	4.95224500	4.45520500
H	0.68581200	5.90080600	4.85102900
C	0.34319900	4.34712900	3.40650200
H	-0.53350700	4.83302200	2.99173800
C	0.60382600	3.36191200	0.01973500
C	0.13050600	3.13073000	-1.28179100
H	-0.66032800	2.41315800	-1.44742700
C	0.65471600	3.83074100	-2.36594900
H	0.27259300	3.64147500	-3.36457800
C	1.66209500	4.77838000	-2.16590700
H	2.05957200	5.33851800	-3.00690000
C	2.13722000	5.01984500	-0.87543600
H	2.90360000	5.77074000	-0.70812500
C	1.61407900	4.31637300	0.21316500
H	1.98477500	4.52403200	1.20995700
C	-1.81661100	2.91814400	1.65223900
C	-2.43068600	2.62919900	2.88449600
H	-1.86976300	2.13644700	3.67213500
C	-3.74947000	3.01771200	3.11411300
H	-4.20372100	2.82535800	4.08137200
C	-4.47674100	3.66842600	2.11164200
H	-5.50177800	3.97483100	2.29548400
C	-3.87803000	3.93131900	0.87877700
H	-4.43526100	4.43318300	0.09463100
C	-2.54835900	3.56845500	0.65070300
H	-2.08719200	3.82134600	-0.29559000
C	-4.40257500	1.43069400	-1.91282900
C	-5.75508900	1.77551600	-1.75421400
H	-6.44529500	1.09016400	-1.27363700
C	-6.20955000	3.01333300	-2.20754000
H	-7.25489200	3.27852000	-2.08739100
C	-5.32269800	3.90820000	-2.81129400
H	-5.68085200	4.87110300	-3.16163600
C	-3.97637300	3.56530500	-2.96950100
H	-3.28920700	4.26127500	-3.44020500
C	-3.51395000	2.32933500	-2.52457100
H	-2.47038400	2.07019600	-2.65547900
C	-3.97100400	-1.43077500	-2.60495300
C	-3.46764300	-2.71682200	-2.33715600
H	-3.05539500	-2.95727500	-1.36152400
C	-3.52795900	-3.69509400	-3.32552900
H	-3.14892200	-4.69149200	-3.12117700
C	-4.08562000	-3.39630500	-4.57410800
H	-4.13363900	-4.16252600	-5.34139000
C	-4.59149300	-2.12096500	-4.83465100
H	-5.03364700	-1.89696900	-5.79994400
C	-4.53810500	-1.13121500	-3.85162800
H	-4.93326400	-0.14055900	-4.05201600
C	-4.83777500	-0.62894600	0.10892100
C	-5.57549900	-1.82019500	0.13448000
H	-5.54557500	-2.50814900	-0.70217900
C	-6.36726000	-2.11155600	1.24600900
H	-6.95281100	-3.02502200	1.26151300
C	-6.41321200	-1.23094800	2.32793200
H	-7.03507300	-1.46168600	3.18728100
C	-5.67633700	-0.04183800	2.29992000
H	-5.72445900	0.65292600	3.13182500
C	-4.89839100	0.27106100	1.18921900
H	-4.37494000	1.21930900	1.14988200
H	-2.42975700	-0.15772300	1.31708400
P	4.22146100	0.66803000	-0.97875600
C	4.52115600	1.00781100	-2.71561400
C	4.93828700	-0.02224900	-3.57644000
C	4.25078300	2.29009200	-3.22271900
C	5.09182000	0.23855500	-4.93687800
H	5.13838100	-1.01523000	-3.18827900
C	4.40857100	2.53842800	-4.58439300
H	3.90884000	3.08635700	-2.57033200

C	4.82960900	1.51637200	-5.43984900
H	5.42433000	-0.55116200	-5.60276400
H	4.21221500	3.53060300	-4.97776300
H	4.95995600	1.71704200	-6.49855900
C	5.29800000	-0.66140400	-0.41302900
C	6.62942600	-0.70234400	-0.86796700
C	4.86356300	-1.57349800	0.55943000
C	7.50202300	-1.66338900	-0.36059400
H	6.98229100	0.00042200	-1.61529800
C	5.74380900	-2.52844000	1.06307600
H	3.84333500	-1.55466500	0.91697900
C	7.06125000	-2.57657700	0.60130400
H	8.52657100	-1.69789100	-0.71631100
H	5.39524700	-3.23141000	1.81320500
H	7.74641100	-3.32264700	0.99103000
C	4.57579900	2.17590500	0.00153700
H	3.84893400	2.93472500	-0.30553800
H	4.33822500	1.92165900	1.04131800
C	6.02012900	2.67182900	-0.12634500
H	6.14732000	3.57086800	0.48299500
H	6.73461300	1.92426100	0.22800200
H	6.26934000	2.92851200	-1.15973400

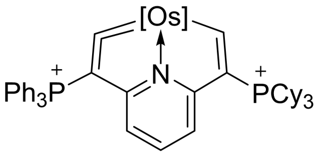

5b′

[Os] = OsCl(PPh_3)$_2$

E = -5109.29918213 a.u.

Os	0.26162000	0.23365400	-1.04896600
Cl	0.46614000	0.68003200	-3.39761500
P	0.33495500	2.64360200	-0.71741700
P	0.18526800	-2.13421500	-1.57589600
P	4.15519200	-0.32741600	1.24012500
P	-3.99700800	-0.07076100	1.00367200
N	0.11516400	-0.22558500	1.03376900
C	2.00826000	0.07489700	-0.44095800
C	2.43762800	-0.21364500	0.82193900
C	1.29243400	-0.42489100	1.70552700
C	1.29057500	-0.77173400	3.05820900
H	2.22150000	-0.96603800	3.57599500
C	0.06644400	-0.87140100	3.70676700
H	0.02854700	-1.13158700	4.76011300
C	-1.12003200	-0.64608900	3.01590300
H	-2.04354300	-0.72895500	3.55433900
C	-1.09609500	-0.33382600	1.65081900
C	-2.19574100	-0.07834600	0.69847700
C	-1.75069500	0.19502000	-0.56852200
C	-0.60274400	3.67901400	-1.89477800
C	-0.33712600	5.05822300	-1.94205700
H	0.44081600	5.48285700	-1.31520500
C	-1.06712400	5.88548500	-2.79251600
H	-0.85474400	6.94958400	-2.82237500
C	-2.06140200	5.34413800	-3.61210600
H	-2.62174000	5.98795700	-4.28304500
C	-2.32395700	3.97411800	-3.57530800
H	-3.08451600	3.54707900	-4.22207700
C	-1.59889200	3.14427700	-2.71959900
H	-1.78937300	2.08041000	-2.71807000
C	-0.28787500	3.16679900	0.92867800
C	-1.38438000	4.02978600	1.06060000
H	-1.82822600	4.48237400	0.18299300
C	-1.90725100	4.32137500	2.32237200
H	-2.75272100	4.99789600	2.40721000
C	-1.33723100	3.76600700	3.46851200
H	-1.74241000	3.99956800	4.44847300
C	-0.22782700	2.92572700	3.34809500
H	0.23266300	2.49588200	4.23289100
C	0.28840700	2.62885700	2.08943400
H	1.14545800	1.97708000	2.00668500
C	2.04207000	3.29805900	-0.86288500
C	2.59491100	3.37179200	-2.15349600
H	2.00640100	3.06965400	-3.01333900
C	3.89720000	3.83607900	-2.32823400
H	4.30715300	3.91438100	-3.33091100
C	4.67098800	4.20108500	-1.22202500
H	5.68744000	4.55587200	-1.36220100
C	4.12960400	4.11543800	0.06014000
H	4.72009100	4.39734200	0.92589500
C	2.81507400	3.67775400	0.24052900
H	2.40152900	3.65359500	1.24058200
C	1.83354400	-2.86597000	-1.89569600
C	2.78686100	-2.10042500	-2.58410800
H	2.54935600	-1.08846500	-2.89135400
C	4.03801600	-2.64167100	-2.87357900
H	4.76933000	-2.03955500	-3.40043700
C	4.35393000	-3.94214100	-2.47647900
H	5.33299500	-4.35612100	-2.69781300
C	3.40492000	-4.71350100	-1.80290500
H	3.63901200	-5.73128900	-1.50482500
C	2.14655300	-4.18103000	-1.51694700
H	1.41812500	-4.78726100	-0.98954300
C	-0.48113900	-3.12116900	-0.18203900
C	-1.79911100	-3.59282000	-0.20391200
H	-2.40600600	-3.44810300	-1.08979400
C	-2.32381600	-4.27513100	0.89568000
H	-3.34020200	-4.65745200	0.85647000
C	-1.54008000	-4.48681700	2.03135900
H	-1.94655200	-5.02521300	2.88227300
C	-0.22280700	-4.02188600	2.05804600
H	0.40092800	-4.19003900	2.93074700
C	0.30306000	-3.34717600	0.95933400
H	1.32991700	-3.00557700	0.98751600
C	-0.84773700	-2.65028400	-2.99582800
C	-1.73885000	-1.76703500	-3.61700500
H	-1.78333500	-0.73351700	-3.30353000
C	-2.53536200	-2.20174300	-4.67745500
H	-3.20744600	-1.50223800	-5.16562600
C	-2.45382700	-3.52148000	-5.12100300
H	-3.06894500	-3.85676200	-5.95037900
C	-1.56797100	-4.40896600	-4.50429900
H	-1.49439100	-5.43516800	-4.85057400
C	-0.76538600	-3.97615000	-3.45163700
H	-0.06979800	-4.66942100	-2.99081500
C	4.41481900	-1.87252400	2.13188800
C	4.95946900	-1.90216400	3.42130300
H	5.27827100	-0.98275300	3.90209000
C	5.08684400	-3.12489100	4.08368200
H	5.50915300	-3.15448600	5.08301000
C	4.67784100	-4.30521300	3.46020800
H	4.78141600	-5.25315000	3.97905000
C	4.14404900	-4.27339200	2.16700400

H	3.83770400	-5.19227200	1.67690800
C	4.00908700	-3.06019500	1.49884100
H	3.60700700	-3.03693700	0.49060300
C	5.13621300	-0.33061600	-0.26873100
C	4.90698100	0.65929900	-1.23772000
H	4.11353400	1.38478700	-1.11442400
C	5.71598700	0.70703900	-2.36985600
H	5.53443300	1.46946300	-3.11975100
C	6.76005100	-0.20890000	-2.52877300
H	7.39512800	-0.15867000	-3.40799700
C	6.99219600	-1.18546400	-1.55815200
H	7.80397600	-1.89542700	-1.68020500
C	6.17948300	-1.25370900	-0.42664900
H	6.35700700	-2.01753700	0.32224000
C	4.69716800	1.07708900	2.24389400
C	3.81417800	1.70446600	3.13631000
H	2.80298200	1.33541300	3.25800300
C	4.23666300	2.81177200	3.86794000
H	3.54940800	3.30016500	4.55169100
C	5.54018900	3.29388800	3.71840200
H	5.86696800	4.15752100	4.28914000
C	6.42291900	2.66659400	2.83660100
H	7.43490600	3.04086600	2.71951200
C	6.00484700	1.56248400	2.09433300
H	6.68570100	1.09255900	1.39257800
C	-4.76011600	-1.46563200	0.03681100
C	-6.24372100	-1.74786200	0.37064200
C	-4.56612600	-1.37419100	-1.48931800
H	-4.16521700	-2.31608400	0.39537000
C	-6.70015100	-3.05009900	-0.30360000
H	-6.86909200	-0.92888700	0.00686100
H	-6.40454300	-1.81662700	1.44759000
C	-5.07791700	-2.64566600	-2.18620900
H	-5.10405000	-0.49913000	-1.87775400
H	-3.51221400	-1.24852300	-1.73648700
C	-6.52866800	-2.96998900	-1.82278100
H	-7.74586900	-3.24283400	-0.04093800
H	-6.11453400	-3.89216600	0.09327100
H	-4.95974200	-2.52922900	-3.26760100
H	-4.43600400	-3.49050500	-1.89992200
H	-6.83468600	-3.91050400	-2.29299000
H	-7.19118300	-2.18862600	-2.22062300
C	-4.34582900	-0.36274000	2.80239900
C	-5.68184700	0.18112900	3.35696800
C	-4.16114600	-1.84964500	3.18727800
H	-3.57067000	0.24029800	3.29080100
C	-5.71372400	0.00056200	4.88416900
H	-6.53057300	-0.34827000	2.91245700
H	-5.79917800	1.24160800	3.11714200
C	-4.20210500	-2.02092600	4.71223000
H	-4.97658200	-2.43512200	2.75025100
H	-3.23514200	-2.26215400	2.77600000
C	-5.50472300	-1.46119100	5.29591700
H	-6.66870600	0.37412300	5.26868700
H	-4.92840500	0.62499900	5.33372000
H	-4.09101900	-3.08207100	4.96118200
H	-3.34523500	-1.50133300	5.16649400
H	-5.49992300	-1.54823900	6.38744600
H	-6.34953100	-2.06490700	4.93650700
C	-4.68927000	1.61523900	0.60878500
C	-3.98060200	2.39837200	-0.50782400
C	-6.21197600	1.60138500	0.34337200

H	-4.49905900	2.15816600	1.54722800
C	-4.52488200	3.83224900	-0.57704800
H	-4.14398200	1.90241800	-1.47333100
H	-2.90691300	2.42448700	-0.33027900
C	-6.75848400	3.03548900	0.27745500
H	-6.39370000	1.10634800	-0.61814900
H	-6.75364800	1.03503700	1.10245300
C	-6.04029400	3.85914000	-0.79577000
H	-4.00535700	4.37120800	-1.37572500
H	-4.28498000	4.34560500	0.36448600
H	-7.83572600	3.00018300	0.08240600
H	-6.63202600	3.51430800	1.25883000
H	-6.40717300	4.89097400	-0.79227600
H	-6.27272700	3.44553800	-1.78719200
H	-2.47777400	0.41551700	-1.34568300

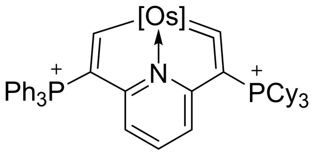

5b

[Os] = $OsCl(PPh_3)_2$

E = -5109.30776183

Os	0.10233300	0.22646500	-0.99058400
Cl	0.48856700	0.67961800	-3.31490600
P	0.29369100	2.63549000	-0.70963100
P	-0.00982800	-2.12763300	-1.57188400
P	4.21786000	-0.36469400	1.18577500
P	-3.92238200	-0.04851400	1.18554500
N	0.17488200	-0.22296700	1.07745300
C	2.09433100	0.06367200	-0.53814700
H	2.83046700	0.22906100	-1.31990400
C	2.48842400	-0.23438500	0.73060200
C	1.38758900	-0.42384200	1.67436900
C	1.46203400	-0.73516300	3.03228000
H	2.41836400	-0.92482500	3.50134700
C	0.28044800	-0.78465100	3.76971900
H	0.31418300	-1.00554700	4.83207300
C	-0.94328400	-0.54819500	3.15923100
H	-1.83581900	-0.57619900	3.75861900
C	-1.00385100	-0.28567800	1.78089500
C	-2.14264800	-0.04506100	0.88410400
C	-1.66746400	0.19532700	-0.38269900
C	-0.66704500	3.59945200	-1.92459000
C	-0.33849200	4.94177000	-2.17284600
H	0.52240200	5.39049800	-1.68755900
C	-1.11753500	5.70397900	-3.04202400
H	-0.85761300	6.74109900	-3.22962700
C	-2.22502200	5.13223500	-3.67307600
H	-2.82714400	5.72555200	-4.35433400
C	-2.54945300	3.79518500	-3.43540200
H	-3.40145600	3.34468100	-3.93593100
C	-1.77283600	3.02876900	-2.56741600
H	-2.01549700	1.98858000	-2.39127900
C	-0.25407800	3.28766600	0.91968900
C	-1.15680700	4.35592500	1.01734300
H	-1.54068700	4.82748500	0.12152900
C	-1.57179200	4.81931800	2.26727100
H	-2.26769700	5.65091100	2.32361200
C	-1.09082800	4.22834500	3.43599500
H	-1.41422900	4.59371600	4.40594200

C	-0.18093700	3.17251300	3.34925300
H	0.20657900	2.70556700	4.25022600
C	0.23080600	2.70838600	2.10199500
H	0.93879900	1.89587300	2.04731500
C	2.01186800	3.25655700	-0.90556800
C	2.56377500	3.30885200	-2.19718800
H	1.97317200	2.99738800	-3.05101500
C	3.86378900	3.77770000	-2.38338300
H	4.27034100	3.83859700	-3.38858800
C	4.63413700	4.17884200	-1.28757600
H	5.64438400	4.54794500	-1.43706400
C	4.09787800	4.10606700	-0.00113900
H	4.68841100	4.40685600	0.85852000
C	2.78964100	3.65600600	0.18894900
H	2.37792600	3.64040900	1.19010500
C	1.62539200	-2.92001500	-1.82411500
C	2.54802700	-2.26197600	-2.65424700
H	2.28482500	-1.31066700	-3.10500400
C	3.78660900	-2.83943000	-2.91978700
H	4.49302100	-2.32004600	-3.55853000
C	4.12023800	-4.07757100	-2.36423800
H	5.08677700	-4.52585400	-2.57359300
C	3.20109500	-4.74494500	-1.55530700
H	3.44568600	-5.71772400	-1.13884000
C	1.95418000	-4.17241400	-1.28789500
H	1.24247000	-4.70752500	-0.66959000
C	-0.82240300	-3.16399500	-0.29447400
C	-2.04498500	-3.79792700	-0.55313000
H	-2.50937900	-3.69517000	-1.52703400
C	-2.65517100	-4.58542800	0.42599800
H	-3.59616900	-5.08120000	0.20568700
C	-2.04884500	-4.75293700	1.67151700
H	-2.51790900	-5.37514800	2.42773800
C	-0.82998700	-4.12342000	1.93776800
H	-0.34790200	-4.24940200	2.90264500
C	-0.22274500	-3.33299400	0.96405100
H	0.73193000	-2.86869200	1.18047500
C	-0.91004200	-2.51629100	-3.11197400
C	-1.81358500	-1.60384800	-3.66975200
H	-1.96086000	-0.63908500	-3.20324200
C	-2.49678900	-1.92575100	-4.84231400
H	-3.18002700	-1.20498800	-5.28092200
C	-2.29184100	-3.16042800	-5.45839600
H	-2.82077600	-3.40601000	-6.37414100
C	-1.39656800	-4.07753000	-4.90187800
H	-1.23029500	-5.03747500	-5.38072900
C	-0.70248800	-3.75638800	-3.73753200
H	0.00455500	-4.46626700	-3.32064300
C	4.43910500	-1.89005800	2.11939600
C	5.21421300	-1.93215000	3.28543000
H	5.69670800	-1.03331500	3.65579400
C	5.35530800	-3.13961800	3.97081300
H	5.95354600	-3.17842200	4.87545400
C	4.73100800	-4.29396300	3.49308500
H	4.84564500	-5.23045400	4.03001400
C	3.96165600	-4.24948000	2.32589400
H	3.48121900	-5.14826500	1.95187000
C	3.80872200	-3.04949200	1.63733100
H	3.21718900	-3.01579300	0.72808600
C	5.23691700	-0.41783200	-0.29792300
C	5.29229500	0.72307100	-1.11715900
H	4.74184200	1.61892400	-0.85560100
C	6.08014100	0.70728700	-2.26383500
H	6.11575500	1.58672700	-2.89840300
C	6.83121500	-0.42878100	-2.58301500
H	7.45642600	-0.43216400	-3.47056300
C	6.78860100	-1.55352900	-1.75793700
H	7.37896800	-2.43116400	-2.00128800
C	5.98552400	-1.55775800	-0.61703800
H	5.94563700	-2.43738900	0.01483400
C	4.76265700	1.06105600	2.15585500
C	3.85493000	1.78722200	2.94088700
H	2.81476400	1.49278300	2.98973800
C	4.28996700	2.89963100	3.65732400
H	3.58172500	3.46409200	4.25582400
C	5.63039600	3.28998400	3.59867700
H	5.96728100	4.15802000	4.15673500
C	6.53835700	2.56531500	2.82280600
H	7.57943600	2.86822900	2.77586200
C	6.10933100	1.45423200	2.09795000
H	6.81358000	0.90768800	1.47949900
C	-4.66548700	-1.24794100	-0.01538600
C	-6.09140800	-1.73137700	0.32869300
C	-4.59537000	-0.74490700	-1.47284500
H	-3.98521600	-2.10487400	0.07684900
C	-6.52595700	-2.83092100	-0.65252400
H	-6.79751400	-0.89709300	0.27056500
H	-6.13875400	-2.11975300	1.34778700
C	-5.06010800	-1.83645900	-2.44727100
H	-5.24042100	0.13367100	-1.58799900
H	-3.57574800	-0.43075400	-1.71395400
C	-6.46260100	-2.34636500	-2.10353400
H	-7.53898400	-3.16068200	-0.39796400
H	-5.86878100	-3.70355500	-0.52646800
H	-5.03217800	-1.44260400	-3.46813100
H	-4.34707800	-2.67010300	-2.42252200
H	-6.74997600	-3.15279300	-2.78673100
H	-7.19208500	-1.53704600	-2.24749400
C	-4.22438800	-0.61097400	2.91870900
C	-5.61351800	-0.30490300	3.51975100
C	-3.84361500	-2.09800000	3.08064600
H	-3.52429700	0.00640500	3.49475200
C	-5.64959900	-0.74760000	4.99244800
H	-6.40105000	-0.82287400	2.96423800
H	-5.82541500	0.76653500	3.46079700
C	-3.90131500	-2.51940000	4.55410400
H	-4.54578800	-2.71464500	2.50956400
H	-2.85471100	-2.29908500	2.66179600
C	-5.27808400	-2.22533600	5.16068300
H	-6.64748900	-0.55329700	5.39962700
H	-4.94927900	-0.12697700	5.56962900
H	-3.65992400	-3.58524700	4.63556700
H	-3.12872400	-1.97928400	5.12133100
H	-5.29288200	-2.49862900	6.22099200
H	-6.03329000	-2.85053500	4.66420000
C	-4.60771000	1.66880300	1.01541100
C	-3.89216000	2.50500700	-0.06114800
C	-6.13720400	1.70901200	0.79221500
H	-4.38336700	2.12149100	1.99391800
C	-4.41183600	3.94834500	-0.06446200
H	-4.05808400	2.05596200	-1.04623900
H	-2.81693300	2.49933900	0.11405100
C	-6.64121400	3.15991900	0.81518800
H	-6.36592400	1.27381600	-0.18674700

H	-6.67220600	1.11534900	1.53626100
C	-5.93188000	4.01121600	-0.24303400
H	-3.90499000	4.50432300	-0.86089200
H	-4.13601200	4.42541400	0.88566200
H	-7.72470500	3.16655900	0.65432800
H	-6.46790200	3.58792600	1.81263600
H	-6.27828100	5.04891400	-0.19024100
H	-6.19755100	3.64108500	-1.24342500

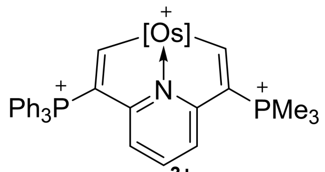

8e^{3+}

[Os] = $OsCl(PPh_3)_2$

E = -4523.60465968 a.u.

Os	-0.98271400	-0.01308200	-0.58354100
Cl	-1.39722700	0.04951300	-3.06317100
P	-0.95972900	-2.37320600	-0.84168400
P	-1.23236200	2.34056700	-0.67044500
P	3.52306200	0.11319200	0.45307700
N	-0.38589800	-0.11160900	1.34662200
C	1.04645700	0.15177500	-0.80296500
C	1.75244400	0.09368900	0.39636800
C	0.96114600	-0.06793700	1.61610600
C	1.39965700	-0.14582100	2.94333400
H	2.45720900	-0.11119700	3.17383200
C	0.46169700	-0.27433400	3.96694000
H	0.79418300	-0.34466900	4.99796500
C	-0.89879300	-0.31827200	3.67152300
H	-1.61774100	-0.42730400	4.47466300
C	-1.31854400	-0.22808900	2.33877100
C	-2.67749400	-0.21581300	1.79489500
C	-2.80993400	-0.13482400	0.41022400
H	-3.84741300	-0.09985400	0.05441400
C	-2.32938900	-3.13153500	-1.79426900
C	-2.27344600	-4.48124600	-2.17034200
H	-1.38771200	-5.07010100	-1.95462800
C	-3.35115000	-5.07309600	-2.82680600
H	-3.29824600	-6.11843100	-3.11682100
C	-4.49203000	-4.32101800	-3.11682700
H	-5.32873800	-4.78138600	-3.63436000
C	-4.54806600	-2.97474600	-2.75395100
H	-5.42339800	-2.38036700	-3.00048000
C	-3.47045100	-2.37916900	-2.09770200
H	-3.48445400	-1.32672900	-1.84960100
C	-1.08006900	-3.23053100	0.78272000
C	-2.26852400	-3.83591100	1.20953600
H	-3.11752600	-3.88683900	0.53603200
C	-2.35810800	-4.39393900	2.48718800
H	-3.27719300	-4.88576100	2.79565200
C	-1.26863100	-4.34175700	3.35830600
H	-1.33957200	-4.78160300	4.34914200
C	-0.08093200	-3.73649800	2.94059600
H	0.77663200	-3.68866000	3.60536200
C	0.01088000	-3.18816400	1.66417800
H	0.93825900	-2.72495400	1.35274200
C	0.55633300	-3.11242500	-1.58085300
C	1.09834500	-4.33204900	-1.14881800
H	0.62381700	-4.88359400	-0.34415400
C	2.26093900	-4.83552100	-1.73736700
H	2.67163700	-5.78191500	-1.39614200
C	2.88878700	-4.12803500	-2.76408900
H	3.79434000	-4.51925600	-3.21936100
C	2.33997800	-2.92432000	-3.21294400
H	2.81726500	-2.37314800	-4.01698900
C	1.18011300	-2.41724600	-2.62813700
H	0.74179600	-1.49009100	-2.98335700
C	-2.57794600	3.00602300	-1.71788600
C	-3.67651500	2.20072400	-2.04113800
H	-3.66766900	1.15865600	-1.75197700
C	-4.74060700	2.72802500	-2.77224300
H	-5.58198100	2.09265000	-3.03383300
C	-4.71201600	4.05880200	-3.19259800
H	-5.53718100	4.46525000	-3.77023300
C	-3.61388300	4.86399700	-2.88203000
H	-3.58317200	5.89739900	-3.21476400
C	-2.55004000	4.34036300	-2.14826600
H	-1.69757900	4.96879100	-1.90953600
C	-1.58471500	3.12338200	0.96502300
C	-0.70628500	2.90263700	2.03735000
H	0.17117300	2.28766700	1.89137800
C	-0.94884700	3.45598100	3.29326400
H	-0.25106500	3.26801600	4.10438300
C	-2.08475500	4.24035300	3.50392700
H	-2.27410200	4.67996900	4.47905600
C	-2.97505700	4.45636800	2.45015700
H	-3.85834200	5.07116600	2.60084000
C	-2.72954600	3.90116400	1.19130700
H	-3.42357100	4.08844800	0.38021700
C	0.24266100	3.25035000	-1.29209400
C	0.63359400	2.98177600	-2.61588300
H	0.06772400	2.26565500	-3.20495900
C	1.74079800	3.62682200	-3.16126900
H	2.02067200	3.42674200	-4.19159200
C	2.49013600	4.52157400	-2.38975900
H	3.35446400	5.02224800	-2.81734700
C	2.11984200	4.77156900	-1.06855900
H	2.69723500	5.46190400	-0.46026400
C	0.99154500	4.14932700	-0.52520900
H	0.69371000	4.38031100	0.49151400
C	4.18170600	1.58042900	1.28743500
C	5.56784000	1.79931700	1.34695300
H	6.25526900	1.07173200	0.92514200
C	6.06032900	2.96456200	1.92952800
H	7.13158100	3.13384700	1.97578700
C	5.17586900	3.92001000	2.43999200
H	5.56384400	4.82990800	2.88778600
C	3.79818700	3.71320400	2.36265500
H	3.10942800	4.46085200	2.74428000
C	3.29896800	2.54593800	1.78548100
H	2.23126400	2.39276100	1.70021300
C	4.08841800	-1.38902500	1.29447700
C	3.54078600	-2.60266600	0.84552000
H	2.84767100	-2.61662800	0.01065200
C	3.88210500	-3.79396100	1.48026500
H	3.45475100	-4.72606700	1.12517200
C	4.75964200	-3.78050000	2.56789000
H	5.02003600	-4.70931800	3.06639500
C	5.30034600	-2.57433300	3.01733500
H	5.97912000	-2.56361800	3.86456700
C	4.96842100	-1.37604900	2.38323400
H	5.38059500	-0.43991300	2.74412200

C	4.20288500	0.15344200	-1.21874000
C	4.81430900	-0.96394600	-1.79883900
H	4.90458700	-1.89186800	-1.24712500
C	5.30485700	-0.88034900	-3.10213800
H	5.78225700	-1.74563000	-3.55149100
C	5.17952700	0.30681500	-3.82441800
H	5.56011400	0.36594700	-4.83964500
C	4.56884200	1.42162800	-3.24282900
H	4.46725300	2.34596400	-3.80164200
C	4.08544500	1.35276700	-1.93994600
H	3.61457100	2.22184400	-1.49330200
H	1.64173700	0.29519900	-1.70864300
P	-4.07753400	-0.19203200	2.86977400
C	-4.06094900	1.24021900	3.99850700
H	-4.06602600	2.15374200	3.39772100
H	-4.93071400	1.22099000	4.66222500
H	-3.14581600	1.24090400	4.59376600
C	-4.23718200	-1.69780000	3.88933100
H	-5.07819900	-1.60830400	4.58362300
H	-4.39034600	-2.55079900	3.22411200
H	-3.31591700	-1.87723500	4.44751900
C	-5.61523100	-0.06540600	1.90981400
H	-5.61392500	0.85411200	1.31970800
H	-5.71382700	-0.92149000	1.23822600
H	-6.46208600	-0.05021400	2.60257900

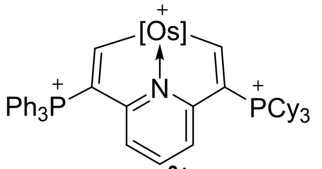

8d^{3+}

[Os] = $OsCl(PPh_3)_2$

E = -5109.74175217

Os	-0.17200200	0.26123700	1.09205900
Cl	-0.30023600	0.66950800	3.36529500
P	-0.11225500	-2.13732900	1.65924500
P	-0.28622600	2.69960900	0.77266400
P	-4.12043400	-0.35528000	-1.35823600
N	-0.08194900	-0.13841600	-1.05092300
C	-2.02514400	0.07624600	0.40390700
C	-2.39441100	-0.16170200	-0.87329200
C	-1.24016800	-0.30431900	-1.75282400
C	-1.21434200	-0.59884100	-3.11669300
H	-2.13809600	-0.75856700	-3.65767200
C	0.01865300	-0.70452500	-3.75757800
H	0.06183700	-0.92934600	-4.81827400
C	1.19435800	-0.52518100	-3.03657300
H	2.15143900	-0.59786300	-3.53100700
C	1.12776700	-0.24686100	-1.66848000
C	2.21546000	-0.04151500	-0.71645600
C	1.73030200	0.19610600	0.52255200
H	2.33570000	0.35545600	1.41646500
C	1.05936200	-2.36777500	3.03388300
C	0.62618000	-2.64214600	4.33474800
H	-0.42807300	-2.78991100	4.54072200
C	1.55605500	-2.72728600	5.37436100
H	1.21484700	-2.94302300	6.38170100
C	2.91486400	-2.54200100	5.12172000
H	3.63268300	-2.61225800	5.93262100
C	3.35378900	-2.27995400	3.81894800
H	4.41449900	-2.15964800	3.61663800
C	2.43131600	-2.18852800	2.78077900
H	2.77752700	-2.01677200	1.76549000
C	0.41771900	-3.34471500	0.38837800
C	1.24173900	-4.42872600	0.73406500
H	1.67842600	-4.49021900	1.72370300
C	1.47469200	-5.45510900	-0.18349800
H	2.09843700	-6.29618700	0.10252500
C	0.89628100	-5.41275300	-1.45359500
H	1.07358300	-6.21848900	-2.15916500
C	0.07483200	-4.33830600	-1.80398900
H	-0.39623600	-4.30184800	-2.78161900
C	-0.16813700	-3.31859200	-0.88640900
H	-0.85522500	-2.53055400	-1.15185300
C	-1.75188900	-2.76652800	2.17897800
C	-2.20257300	-4.02466800	1.74756300
H	-1.58119000	-4.64296100	1.11104800
C	-3.45161300	-4.49918800	2.15230500
H	-3.78538000	-5.47758700	1.82077700
C	-4.25628600	-3.73205600	2.99439300
H	-5.22088600	-4.11012700	3.31773500
C	-3.81100900	-2.48283200	3.43233000
H	-4.42894200	-1.88383200	4.09282000
C	-2.57079900	-1.99758300	3.02469000
H	-2.23196300	-1.03496500	3.38858700
C	0.56280700	3.71412300	2.02414700
C	1.68209200	3.23996800	2.72042100
H	2.02943900	2.22493000	2.57516900
C	2.34401400	4.06359100	3.63025400
H	3.19758400	3.68203500	4.18061600
C	1.89958300	5.36925900	3.84488700
H	2.41101400	6.00656200	4.55919300
C	0.79265200	5.85357200	3.14383900
H	0.44565800	6.86900700	3.30590000
C	0.12387300	5.03216800	2.23860600
H	-0.73712500	5.41609600	1.70203500
C	0.37128800	3.29597700	-0.82736700
C	-0.17615800	2.84132300	-2.03801700
H	-0.97828100	2.11751900	-2.02739000
C	0.27476600	3.34440100	-3.25586200
H	-0.17025700	2.99453700	-4.18277900
C	1.28653400	4.30784900	-3.28194800
H	1.62188000	4.71934900	-4.22854300
C	1.85705800	4.74471800	-2.08596000
H	2.64299800	5.49343600	-2.09932400
C	1.40589800	4.24324100	-0.86325200
H	1.84143300	4.61044500	0.05852100
C	-2.03268000	3.25298500	0.88031900
C	-2.63857800	3.22877600	2.15001300
H	-2.07516600	2.89918400	3.01696000
C	-3.94952300	3.67463600	2.30790600
H	-4.39522900	3.68733700	3.29794100
C	-4.67935800	4.12229000	1.20138900
H	-5.69798400	4.47565200	1.32791300
C	-4.08977700	4.12302500	-0.06322800
H	-4.64783300	4.46677900	-0.92812400
C	-2.76729100	3.70125200	-0.22429400
H	-2.31370200	3.75538600	-1.20541100
C	-4.67959700	1.08120700	-2.29067300
C	-6.03402900	1.44737400	-2.21944700
H	-6.72683800	0.87871300	-1.60816700
C	-6.48659500	2.55874100	-2.92955900
H	-7.53322400	2.84021500	-2.87646300

C	-5.59620300	3.30783200	-3.70277700
H	-5.95286300	4.17351200	-4.25191400
C	-4.24820200	2.94336800	-3.77460900
H	-3.55794900	3.52586300	-4.37656500
C	-3.78782700	1.83180600	-3.07357000
H	-2.74272300	1.55520700	-3.13800000
C	-4.21865700	-1.86410200	-2.33047900
C	-3.63360700	-3.03383900	-1.81179000
H	-3.16070100	-3.02888500	-0.83387200
C	-3.68354600	-4.21003900	-2.55431100
H	-3.23846700	-5.11670500	-2.15677900
C	-4.30987500	-4.22290100	-3.80599700
H	-4.34886800	-5.14274100	-4.38094300
C	-4.89295200	-3.06118300	-4.31663400
H	-5.38435200	-3.07775800	-5.28393500
C	-4.85097200	-1.87522100	-3.58174700
H	-5.30385900	-0.97148200	-3.97670000
C	-5.12190400	-0.48805900	0.12870900
C	-5.84672300	-1.65388800	0.41036700
H	-5.79094600	-2.51287000	-0.24835900
C	-6.66019200	-1.69561900	1.54325500
H	-7.23819200	-2.58926100	1.75447000
C	-6.73977400	-0.59179200	2.39377600
H	-7.38118900	-0.62875700	3.26885500
C	-6.01023000	0.56853500	2.11329200
H	-6.08167800	1.43271700	2.76549700
C	-5.20994100	0.63143600	0.97655500
H	-4.68970900	1.55102600	0.73459800
H	-2.72264300	0.18976900	1.23641300
P	3.99163700	-0.30636600	-1.02295100
C	4.48414600	0.49909100	-2.60765300
C	3.81430600	1.86638800	-2.85050800
C	6.01475900	0.59118500	-2.79535700
H	4.11877700	-0.19442300	-3.37364700
C	4.13833300	2.34018200	-4.27591200
H	4.18875600	2.59684700	-2.12650000
H	2.73151000	1.81126900	-2.70702300
C	6.32389000	1.05148500	-4.22814700
H	6.42070500	1.32509400	-2.09170100
H	6.50327300	-0.36670900	-2.58381500
C	5.64955500	2.39453900	-4.53352600
H	3.68810100	3.32437900	-4.43725300
H	3.66460000	1.65656000	-4.99546400
H	7.40826300	1.12885200	-4.35616700
H	5.97665800	0.28727500	-4.93734100
H	5.84333900	2.68532300	-5.57078800
H	6.09599500	3.17441100	-3.90124500
C	4.84211200	0.42092100	0.45717300
C	6.37009800	0.19482000	0.57214200
C	4.54788400	1.93317500	0.61467400
H	4.37732500	-0.11744400	1.29654500
C	6.84893600	0.66103100	1.95703800
H	6.88474400	0.77603600	-0.19713800
H	6.64247900	-0.84776000	0.42212400
C	5.02698800	2.41266800	1.99302200
H	5.09433900	2.47749500	-0.16380200
H	3.48868800	2.16725700	0.47511800
C	6.51978300	2.13701500	2.20157200
H	7.92728300	0.49022700	2.03634900
H	6.37498800	0.03956700	2.73094800
H	4.80963100	3.48046600	2.09626800
H	4.44698100	1.89606000	2.77226200
H	6.82090400	2.43084500	3.21216700
H	7.10229100	2.75821300	1.50802900
C	4.13925800	-2.17617200	-1.14170500
C	4.30715600	-2.67482800	-2.59374000
C	5.20690800	-2.81088200	-0.22766200
H	3.15569600	-2.50793900	-0.78198100
C	4.24337300	-4.20943300	-2.64794400
H	5.27085000	-2.33193800	-2.98989700
H	3.52443500	-2.26688200	-3.24120500
C	5.10149600	-4.34196000	-0.27425000
H	6.20481500	-2.51804600	-0.57212600
H	5.09797000	-2.45994400	0.80457000
C	5.25884600	-4.86428400	-1.70601800
H	4.41111700	-4.53402800	-3.68043000
H	3.22946400	-4.52513500	-2.37442200
H	5.86747300	-4.77215500	0.37936100
H	4.12784000	-4.64591400	0.13036000
H	5.14213000	-5.95279900	-1.72735000
H	6.27719200	-4.65393900	-2.05950100

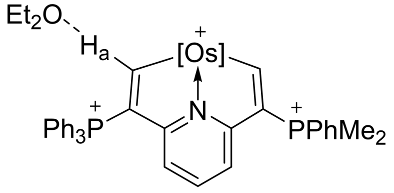

6a-1

[Os] = $OsCl(PPh_3)_2$

E = -4948.96398229

Os	0.69273400	0.13831300	0.35443800
Cl	1.05261000	1.31436800	2.29177900
P	0.66214200	-1.89457500	1.73953500
P	0.89942800	2.24342000	-0.90283400
P	-3.76435200	-0.58759200	-0.96205200
N	0.21901400	-1.02006700	-1.44073700
C	-1.29092800	0.13411100	0.09831000
C	-1.96773300	-0.49485200	-0.88708100
C	-1.07980900	-1.18727400	-1.81149100
C	-1.40979500	-1.93298400	-2.94457600
H	-2.44583300	-2.05716100	-3.23131500
C	-0.38711700	-2.51545800	-3.69088900
H	-0.62502100	-3.10300400	-4.57158100
C	0.93919900	-2.33868000	-3.30628000
H	1.73993600	-2.78489600	-3.88260600
C	1.22146500	-1.57666300	-2.17219200
C	2.51212100	-1.23375300	-1.59581500
C	2.38582400	-0.47018800	-0.48519600
H	3.23654500	-0.09257800	0.08452200
C	1.96337800	-1.82214300	3.01272700
C	1.64093000	-1.92123500	4.37205300
H	0.61418300	-2.07803000	4.68175300
C	2.64434400	-1.81594700	5.33776400
H	2.38572200	-1.89423000	6.38884400
C	3.97046700	-1.61428100	4.95689600
H	4.74623900	-1.53200700	5.71137000
C	4.29884600	-1.52254800	3.60079100
H	5.32989000	-1.37153500	3.29607100
C	3.30224800	-1.61961500	2.63453600
H	3.57699800	-1.55813000	1.58677000
C	0.94556300	-3.42750900	0.78372700
C	2.09083500	-4.21635000	0.96067400
H	2.83494800	-3.94091700	1.69906100

C	2.25734500	-5.38701500	0.21489900
H	3.13292100	-6.00808300	0.38066800
C	1.28844800	-5.77857700	-0.71086600
H	1.41590500	-6.69598100	-1.27721100
C	0.13733000	-5.00333100	-0.87868900
H	-0.63693900	-5.31346800	-1.57387300
C	-0.03354400	-3.83876100	-0.13531900
H	-0.94687800	-3.26876700	-0.24957800
C	-0.90646900	-2.18239800	2.62577400
C	-1.37459700	-3.48638600	2.85411600
H	-0.82447500	-4.33991100	2.47430000
C	-2.55018400	-3.68874600	3.57902000
H	-2.90351100	-4.69939000	3.75897600
C	-3.25829600	-2.59798200	4.08859100
H	-4.16737600	-2.76093200	4.65916000
C	-2.78235200	-1.30079300	3.88324800
H	-3.31802300	-0.45129900	4.29277600
C	-1.61253600	-1.09115000	3.15548000
H	-1.23357800	-0.08343000	3.03293400
C	2.06684400	3.43491100	-0.16751600
C	3.24163600	2.99569800	0.46197800
H	3.44501600	1.93802100	0.57576600
C	4.15457800	3.91778500	0.96852500
H	5.05156800	3.56793500	1.47030300
C	3.90406100	5.28749000	0.85199100
H	4.61043100	6.00566400	1.25604800
C	2.74146900	5.73116600	0.21970500
H	2.54353900	6.79405100	0.12596800
C	1.82454100	4.81169900	-0.28968900
H	0.92196400	5.16725100	-0.77371600
C	1.50077800	2.00862200	-2.61700900
C	0.75131100	1.26271100	-3.54090200
H	-0.19171600	0.82021700	-3.24396000
C	1.19504300	1.10406000	-4.85275000
H	0.59511300	0.53779700	-5.55878900
C	2.39667500	1.68835900	-5.26202400
H	2.73363000	1.58130300	-6.28849600
C	3.15186500	2.42876600	-4.34948100
H	4.07399600	2.90667800	-4.66671700
C	2.70849000	2.58960200	-3.03393200
H	3.28709300	3.19311800	-2.34386000
C	-0.67732000	3.15082800	-1.01438300
C	-1.27433100	3.51284200	0.20390100
H	-0.85034200	3.21579900	1.15687300
C	-2.41637600	4.30928700	0.20858200
H	-2.83662600	4.60376900	1.16457300
C	-2.99346600	4.71896700	-0.99767700
H	-3.88246200	5.34236100	-0.99511500
C	-2.41390800	4.33829900	-2.20996200
H	-2.85389800	4.66102900	-3.14822300
C	-1.24729200	3.56898200	-2.22381100
H	-0.77866600	3.32348500	-3.17016800
C	-4.36032500	0.26682000	-2.42614400
C	-5.74835200	0.34380800	-2.64413500
H	-6.44359400	-0.11972300	-1.95009800
C	-6.23263300	1.03492800	-3.75137500
H	-7.30215700	1.09329700	-3.92461300
C	-5.34301200	1.66126800	-4.63071300
H	-5.72645300	2.20165000	-5.49035100
C	-3.96716900	1.60445800	-4.40051300
H	-3.28080100	2.10482000	-5.07628800
C	-3.47087000	0.90928600	-3.29896100

H	-2.40495500	0.88849700	-3.10840500
C	-4.15727400	-2.34565800	-0.99246600
C	-3.60695600	-3.15315100	0.01967800
H	-2.99724300	-2.71794300	0.80513500
C	-3.85613300	-4.52262500	0.01899300
H	-3.43646700	-5.14333500	0.80398500
C	-4.64688500	-5.09029700	-0.98568400
H	-4.84263200	-6.15792000	-0.98237300
C	-5.19088300	-4.28779000	-1.99086000
H	-5.80706400	-4.72979600	-2.76707100
C	-4.94893500	-2.91313800	-2.00068500
H	-5.36948300	-2.29494100	-2.78644300
C	-4.47910400	0.20855400	0.48386900
C	-5.11809400	-0.52898600	1.49086000
H	-5.15939300	-1.61059900	1.44443500
C	-5.72278300	0.14449700	2.55213600
H	-6.23472500	-0.42127700	3.32390400
C	-5.68711600	1.53884900	2.61190100
H	-6.16905900	2.05770400	3.43413600
C	-5.04535600	2.27138100	1.60808700
H	-5.02548000	3.35493800	1.65136000
C	-4.44858200	1.61366600	0.53687900
H	-3.98314900	2.18741800	-0.25689400
H	-1.77035800	0.71590300	0.88743100
O	-1.43546600	3.35586300	3.26609600
C	-0.54368500	4.12965700	4.07486300
H	-1.11348000	4.78590500	4.74467300
H	0.05602900	3.45110800	4.70135300
C	0.34978700	4.95300200	3.16409000
H	-0.24979000	5.60340000	2.51887800
H	1.01112700	5.58598600	3.76414000
H	0.96906500	4.31046300	2.53174900
C	-2.24054000	2.43697200	3.99232400
H	-2.69515100	1.79473600	3.22894300
C	-3.33563200	3.09493600	4.82511300
H	-3.94836600	3.75245000	4.19948200
H	-3.98593300	2.33016300	5.26511500
H	-2.92725100	3.68912200	5.64708100
H	-1.60040600	1.80563500	4.63045700
P	4.12520600	-1.70379200	-2.22001700
C	4.34429400	-1.11742600	-3.92446800
H	5.35966300	-1.35284400	-4.25754700
H	3.62434400	-1.60170500	-4.58872400
H	4.18018300	-0.03813700	-3.96839600
C	4.30172700	-3.50997000	-2.19973100
H	3.58976400	-3.95314000	-2.90088500
H	5.31656800	-3.79039000	-2.49692000
H	4.07560000	-3.89565500	-1.20309500
C	5.33506000	-0.93102100	-1.14275900
C	6.04651800	-1.68704700	-0.19782700
C	5.50870900	0.46399200	-1.21315300
C	6.93426600	-1.04693200	0.66761900
H	5.92500000	-2.76338700	-0.13739700
C	6.39282000	1.09195200	-0.33956000
H	4.95474200	1.05633900	-1.93574400
C	7.10547700	0.33782100	0.59852700
H	7.50135000	-1.63187400	1.38456100
H	6.53069000	2.16663900	-0.39651300
H	7.80357500	0.82914200	1.26884100

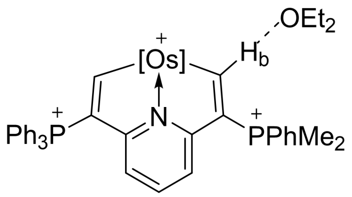

7a-1

[Os] = $OsCl(PPh_3)_2$

E = -4948.95608122

Os	0.46011900	0.47379300	0.69542800
Cl	0.72770500	1.65554100	2.65977100
P	0.37345800	-1.55393600	2.08914700
P	0.53387200	2.62172600	-0.51271700
P	-4.01258800	-0.21711600	-0.61661300
N	-0.02020400	-0.69282000	-1.09076200
C	-1.52822100	0.43619200	0.46431100
C	-2.21017200	-0.19247100	-0.51578300
C	-1.32310600	-0.87895600	-1.44597000
C	-1.65847000	-1.64123400	-2.56538100
H	-2.69668400	-1.80410200	-2.82236700
C	-0.63692800	-2.19137100	-3.33767300
H	-0.87816100	-2.78040800	-4.21651100
C	0.69030400	-1.98310700	-2.98285500
H	1.48948700	-2.40101200	-3.57747000
C	0.97942100	-1.23355400	-1.83832800
C	2.27615700	-0.88889700	-1.27267300
C	2.16310600	-0.10187200	-0.17309100
H	3.01465200	0.33713400	0.36758300
C	1.56001800	-1.51125100	3.47069800
C	1.17571900	-1.95042300	4.74538300
H	0.15956600	-2.28005800	4.92815300
C	2.10235200	-1.96530700	5.78883800
H	1.79561600	-2.30238800	6.77372400
C	3.41531000	-1.55110300	5.56696600
H	4.13430700	-1.56334800	6.37981700
C	3.80403900	-1.11704600	4.29657500
H	4.82519100	-0.79253000	4.12769400
C	2.88166100	-1.08919300	3.25362200
H	3.19224200	-0.73944800	2.27461300
C	0.69996200	-3.13518100	1.22516300
C	1.74644900	-3.97584100	1.63225700
H	2.42108300	-3.66323800	2.42107100
C	1.89254100	-5.24311500	1.06013600
H	2.68879700	-5.89656800	1.40326000
C	1.00064700	-5.67932200	0.07903800
H	1.09622400	-6.67669800	-0.33906800
C	-0.02980900	-4.83663600	-0.34792600
H	-0.73097300	-5.16898500	-1.10746300
C	-0.18062000	-3.57560800	0.22271000
H	-1.01703700	-2.95866300	-0.07907900
C	-1.26190600	-1.79666300	2.87570600
C	-1.85272100	-3.06599900	2.96409200
H	-1.36706500	-3.92972400	2.52565000
C	-3.06587700	-3.22900000	3.63788300
H	-3.51184800	-4.21639300	3.70743000
C	-3.68894100	-2.13455600	4.23705900
H	-4.62589100	-2.26650500	4.76882300
C	-3.09303500	-0.87238000	4.17064600
H	-3.56534900	-0.02031500	4.64720300
C	-1.88844000	-0.70134500	3.49450500
H	-1.41805000	0.27561600	3.48257800
C	1.59465200	3.93186100	0.18366600
C	2.67868800	3.65020300	1.02363900
H	2.95343600	2.63233300	1.26325900
C	3.42052300	4.69652900	1.57416400
H	4.22903900	4.47189900	2.26109100
C	3.11447900	6.02116500	1.26254100
H	3.69087500	6.83055900	1.69937200
C	2.06288600	6.30508700	0.38700800
H	1.82764600	7.33286300	0.12987000
C	1.29874100	5.26901200	-0.14265200
H	0.46842900	5.50167100	-0.80072000
C	1.07036400	2.44530200	-2.25410400
C	0.36473900	1.62964000	-3.15316900
H	-0.53305800	1.11706700	-2.83277700
C	0.79877300	1.48506700	-4.47004600
H	0.23359300	0.86222400	-5.15695500
C	1.94706600	2.15065400	-4.90753400
H	2.27446200	2.05373400	-5.93822500
C	2.65802700	2.95978900	-4.01897200
H	3.54058600	3.49466200	-4.35658100
C	2.22606600	3.10685900	-2.69938900
H	2.77344600	3.75519600	-2.02468400
C	-1.11620100	3.42177700	-0.50148800
C	-1.53223400	3.99410800	0.71535700
H	-0.89513000	3.93412300	1.59232400
C	-2.73763400	4.68877200	0.78442700
H	-3.03175200	5.16123200	1.71683300
C	-3.55657100	4.79045100	-0.34585100
H	-4.49340400	5.33606400	-0.29178300
C	-3.16071500	4.19729700	-1.54416600
H	-3.79151700	4.26860400	-2.42370800
C	-1.93804600	3.52476400	-1.62849800
H	-1.62385000	3.11595600	-2.58084400
C	-4.58942200	0.83454700	-1.96160300
C	-5.85683700	1.43036400	-1.85116700
H	-6.45686600	1.28622100	-0.95882200
C	-6.34192300	2.22019600	-2.89291900
H	-7.32174100	2.67869400	-2.80798000
C	-5.57008800	2.41943100	-4.04003700
H	-5.95283500	3.03270300	-4.84972900
C	-4.30660700	1.83044400	-4.14842000
H	-3.70737400	1.98917000	-5.03943900
C	-3.81302800	1.04021100	-3.11344700
H	-2.83033800	0.59363700	-3.20484200
C	-4.48708600	-1.93130000	-0.88207900
C	-3.92082800	-2.91426700	-0.05122100
H	-3.23515900	-2.63677200	0.74322600
C	-4.26583900	-4.24954800	-0.23805900
H	-3.83704700	-5.01159800	0.40530200
C	-5.16786900	-4.60555200	-1.24727100
H	-5.43645000	-5.64761500	-1.38925900
C	-5.73164300	-3.62643400	-2.06831900
H	-6.43663100	-3.90598000	-2.84439600
C	-5.39494400	-2.28343500	-1.89045400
H	-5.83225500	-1.52174100	-2.52785000
C	-4.71119600	0.38958700	0.92308900
C	-5.53738300	-0.42686100	1.70735200
H	-5.72924900	-1.45493800	1.42278800
C	-6.12510100	0.10014000	2.85798800
H	-6.78054000	-0.52252600	3.45824800
C	-5.87813000	1.42211700	3.23226700
H	-6.34282900	1.82698900	4.12591400

C	-5.04902000	2.23315900	2.44936500
H	-4.86964400	3.26583800	2.73051700
C	-4.47553800	1.72715900	1.28664800
H	-3.88237400	2.37580800	0.65344600
H	-2.00084000	0.99975500	1.26812300
O	4.98378300	1.35959900	0.79578200
C	5.85156000	0.99467200	1.88970200
H	5.61754800	-0.04958200	2.12939700
H	6.89628800	1.02287100	1.56071600
C	5.62510900	1.88085300	3.10326600
H	4.57367200	1.85490100	3.40866200
H	6.23401500	1.53279100	3.94480300
H	5.90422400	2.91697600	2.89446300
C	5.61258200	2.04471900	-0.30060900
H	6.29340700	1.34750900	-0.81438000
H	4.78354900	2.27604300	-0.97913400
C	6.35815200	3.32376200	0.05120700
H	5.68926800	4.06160700	0.49733900
H	7.19294100	3.14085600	0.73355500
H	6.77760800	3.75250800	-0.86538700
P	3.86347200	-1.36745200	-1.97412800
C	3.75803600	-2.95991900	-2.79281300
C	3.29123300	-4.06005700	-2.05362100
C	4.15279100	-3.11673400	-4.13111300
C	3.22986700	-5.31194400	-2.65721500
H	2.96953700	-3.94314000	-1.02326300
C	4.08491600	-4.37741700	-4.72424500
H	4.51404100	-2.27460900	-4.71150100
C	3.62654500	-5.47219800	-3.98871800
H	2.87578800	-6.16436500	-2.08826500
H	4.39648100	-4.50400800	-5.75583000
H	3.58181300	-6.45258600	-4.45222600
C	4.40154800	-0.09814800	-3.15292400
H	3.66458200	0.00066800	-3.95384900
H	4.46280400	0.85683800	-2.62862500
H	5.38061900	-0.34898300	-3.57073300
C	5.05172500	-1.49366100	-0.61479300
H	6.03678000	-1.72921600	-1.02830500
H	5.08722900	-0.55024900	-0.06334600
H	4.74124100	-2.30477400	0.04963500

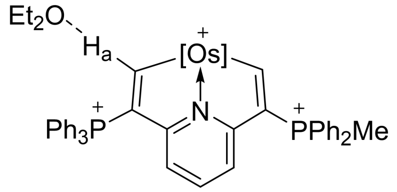

6b-1

[Os] = $OsCl(PPh_3)_2$

E = -5140.82161067

Os	0.31041900	0.56726100	0.37411100
Cl	0.05977000	2.27447300	1.89365400
P	0.58433200	-0.91988600	2.31148500
P	0.09519600	2.24598900	-1.41703600
P	-3.51397300	-1.81611800	-0.95821300
N	0.45475500	-1.08714400	-1.04936000
C	-1.52620900	-0.12603400	0.00632600
C	-1.84743300	-1.15295000	-0.81071100
C	-0.67383200	-1.73742600	-1.44673400
C	-0.60394400	-2.80692100	-2.34066700
H	-1.50727600	-3.31619000	-2.65069800
C	0.64443800	-3.21371200	-2.81176000
H	0.71810100	-4.05280200	-3.49586300
C	1.79452200	-2.54385300	-2.40293000
H	2.77348300	-2.85956200	-2.74253700
C	1.67116500	-1.46622100	-1.52399200
C	2.71299600	-0.60664100	-0.98481900
C	2.21692400	0.31325700	-0.12520500
H	2.83244800	1.05684000	0.38517900
C	1.67349600	-0.11754500	3.52961700
C	1.19359500	0.26527800	4.78741100
H	0.17396200	0.03756000	5.07702400
C	2.03018400	0.94629800	5.67460400
H	1.65295400	1.23842200	6.64938900
C	3.34320900	1.24714800	5.31380400
H	3.98885400	1.77710300	6.00678700
C	3.82958600	0.85724200	4.06170300
H	4.85371800	1.07902300	3.77823400
C	2.99976400	0.18192400	3.17171300
H	3.39599500	-0.13373400	2.21113700
C	1.31879700	-2.56174100	1.98240900
C	2.41177100	-3.04047600	2.71959100
H	2.89893200	-2.40644500	3.45076800
C	2.85556400	-4.35338700	2.54074600
H	3.68895500	-4.71966600	3.13236800
C	2.21972600	-5.19524400	1.62662100
H	2.55802700	-6.21946700	1.50214800
C	1.12991900	-4.72313000	0.88919400
H	0.61420300	-5.37544100	0.19118200
C	0.67990800	-3.41880800	1.07184900
H	-0.19964300	-3.08652300	0.53911800
C	-0.98246500	-1.31423000	3.16850300
C	-1.18386100	-2.58214700	3.73763500
H	-0.41675100	-3.34421400	3.66322300
C	-2.37338300	-2.86660200	4.41054000
H	-2.51839600	-3.84711900	4.85345500
C	-3.36379200	-1.89018700	4.53013800
H	-4.28395300	-2.11269900	5.06144100
C	-3.15940000	-0.62162100	3.98268700
H	-3.91955800	0.14515700	4.08490600
C	-1.97756200	-0.33247400	3.30366900
H	-1.82014600	0.66468100	2.91035100
C	0.77995700	3.88179500	-0.99182300
C	1.93147300	3.99681200	-0.19881900
H	2.39848800	3.11795900	0.22772700
C	2.47614900	5.25030800	0.07160500
H	3.35373100	5.33053900	0.70565700
C	1.87984900	6.40083800	-0.45030900
H	2.29910400	7.37773900	-0.23148900
C	0.74045300	6.29265400	-1.24948600
H	0.27462000	7.18342100	-1.65845200
C	0.18971700	5.04027300	-1.52050300
H	-0.69946200	4.96832400	-2.13665700
C	0.91144500	1.80989200	-2.99888700
C	0.55954900	0.63758400	-3.68750700
H	-0.17811900	-0.03591200	-3.27102300
C	1.12719100	0.34233100	-4.92612200
H	0.83250100	-0.56178500	-5.45050300
C	2.05581500	1.21562100	-5.49798800
H	2.48541400	0.99554000	-6.47031500
C	2.41806100	2.38072300	-4.81875000
H	3.12706100	3.07244000	-5.26347600
C	1.85293100	2.67769300	-3.57570500
H	2.12229100	3.59932800	-3.07294800

C	-1.65598400	2.57550000	-1.81415400
C	-2.43955500	3.11525700	-0.78105200
H	-2.03835400	3.27762600	0.21291500
C	-3.75602500	3.49318500	-1.03154000
H	-4.32558100	3.94281400	-0.22492100
C	-4.31495300	3.29943400	-2.29891300
H	-5.34042700	3.59679200	-2.49630700
C	-3.54590300	2.73307100	-3.31775500
H	-3.97386500	2.58348100	-4.30391600
C	-2.21221900	2.38529900	-3.08531600
H	-1.61109400	1.99729700	-3.89970500
C	-4.12294000	-1.59082700	-2.63383700
C	-5.44543200	-1.97263400	-2.92476800
H	-6.07705900	-2.40698800	-2.15509900
C	-5.95106700	-1.77673800	-4.20708400
H	-6.96964300	-2.07256700	-4.43573600
C	-5.15106400	-1.19080000	-5.19368000
H	-5.55134600	-1.03760000	-6.19084200
C	-3.84545100	-0.79352500	-4.89856300
H	-3.23197400	-0.32771600	-5.66340600
C	-3.32718900	-0.99002500	-3.61957100
H	-2.32190800	-0.66080800	-3.38776100
C	-3.39513600	-3.55650500	-0.50959000
C	-2.80284100	-3.87270900	0.72657000
H	-2.46307700	-3.08822200	1.39543300
C	-2.66180700	-5.20605800	1.10032000
H	-2.20920200	-5.44942500	2.05619600
C	-3.10435100	-6.22304800	0.24783000
H	-2.99683800	-7.26203000	0.54306300
C	-3.69042500	-5.90758000	-0.98001500
H	-4.03676100	-6.69831600	-1.63752500
C	-3.83835600	-4.57446300	-1.36571800
H	-4.29138200	-4.33348700	-2.32140900
C	-4.60261400	-0.93358200	0.16879800
C	-4.90422600	0.40647800	-0.13341200
H	-4.48177700	0.88604400	-1.00970500
C	-5.77458100	1.11386400	0.69013200
H	-6.00864500	2.14744200	0.46036200
C	-6.35947600	0.48793400	1.79588900
H	-7.05254700	1.03920200	2.42329600
C	-6.06717600	-0.84591800	2.08545800
H	-6.53319900	-1.33436200	2.93508100
C	-5.18502300	-1.56349300	1.27736900
H	-4.96565000	-2.60000300	1.50459700
H	-2.25739700	0.44826800	0.57831100
O	-2.92308400	3.83781400	2.14453300
C	-2.39576600	5.02051200	2.75172100
H	-3.21231100	5.65704100	3.11595100
H	-1.77942200	4.73783500	3.61931600
C	-1.56577800	5.76278800	1.71907500
H	-2.17388800	6.01539400	0.84432300
H	-1.18163800	6.69421100	2.14679600
H	-0.71599700	5.15836400	1.38828900
C	-3.59747300	2.96204500	3.03644800
H	-3.72543000	2.03009600	2.47375600
C	-4.95894000	3.47281400	3.49627800
H	-5.59188500	3.70754800	2.63361800
H	-5.46468500	2.71020400	4.09987700
H	-4.87465700	4.37309800	4.11114600
H	-2.95358400	2.74769100	3.90531400
P	4.43335300	-0.58837900	-1.49335900
C	4.50602100	-0.15398100	-3.25565100
H	5.54700700	-0.07121000	-3.57846200
H	4.00032300	-0.92192500	-3.84515900
H	3.99769200	0.79831100	-3.41913000
C	5.24346800	0.69077700	-0.52248400
C	6.05604000	0.33088900	0.56357300
C	5.01052800	2.04564600	-0.82087100
C	6.63575700	1.32911800	1.34727900
H	6.24486200	-0.71474600	0.78394400
C	5.59280100	3.03251900	-0.02904300
H	4.38102800	2.33166300	-1.65850100
C	6.40411500	2.67511400	1.05285700
H	7.28140700	1.05549600	2.17570500
H	5.42306500	4.07802700	-0.26422800
H	6.86655300	3.44775300	1.65890700
C	5.19773500	-2.18802600	-1.23330200
C	6.39498300	-2.49054600	-1.90750300
C	4.63936000	-3.10388600	-0.32627800
C	7.02620200	-3.70955000	-1.67581000
H	6.83992200	-1.78379100	-2.60098500
C	5.28200700	-4.32083900	-0.10639800
H	3.71914500	-2.87390400	0.20075700
C	6.46888300	-4.62392300	-0.77737800
H	7.94970400	-3.94559400	-2.19407200
H	4.85317400	-5.03157000	0.59004200
H	6.96312500	-5.57385000	-0.60005700

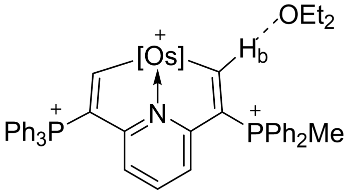

7b-1

[Os] = $OsCl(PPh_3)_2$

E = -5140.81433647

Os	-0.10379900	0.07718900	-0.90728200
Cl	-0.51098800	0.25388700	-3.17141300
P	-0.07880200	-2.36751000	-1.21230000
P	-0.04705900	2.54435300	-0.82743000
P	4.28549900	-0.11727100	0.76422000
N	0.25589600	-0.11601900	1.24395400
C	1.86105400	0.03378800	-0.58270500
C	2.48661000	-0.08465400	0.60786100
C	1.53468700	-0.19935100	1.70969400
C	1.79071600	-0.38211400	3.06790600
H	2.80879900	-0.47318900	3.42346100
C	0.71314900	-0.48056900	3.94704500
H	0.89047500	-0.64343700	5.00517000
C	-0.58878100	-0.37112600	3.47231700
H	-1.42873600	-0.44583100	4.14616100
C	-0.79899200	-0.16828900	2.10497200
C	-2.05137000	-0.00531400	1.37621600
C	-1.85164200	0.06869900	0.03955200
H	-2.65685800	0.16752100	-0.69408600
C	-1.36728100	-2.95032500	-2.35585900
C	-1.04660600	-3.66619200	-3.51618700
H	-0.01151600	-3.85572000	-3.77585600
C	-2.06602600	-4.14680600	-4.34053000
H	-1.81224700	-4.69716600	-5.24075800
C	-3.40306900	-3.93650200	-4.00253100
H	-4.19150900	-4.32617300	-4.63857800
C	-3.72555100	-3.22673200	-2.84193900

H	-4.76380700	-3.07014300	-2.56648500
C	-2.71351400	-2.71847100	-2.03262700
H	-2.97392500	-2.17435900	-1.13080500
C	-0.30787600	-3.34332000	0.32015400
C	-1.36774500	-4.24640400	0.48402500
H	-2.08532200	-4.39677600	-0.31298900
C	-1.49247700	-4.97524500	1.66982400
H	-2.31029200	-5.68085800	1.78105300
C	-0.56111000	-4.82026900	2.69703600
H	-0.65931900	-5.39497100	3.61264300
C	0.51531600	-3.94498000	2.52711500
H	1.26670200	-3.83828700	3.30371000
C	0.64033000	-3.21768900	1.34749500
H	1.49881500	-2.57644600	1.21254300
C	1.51709400	-2.94127200	-1.89735800
C	2.12985700	-4.11148300	-1.42449600
H	1.67188200	-4.68600800	-0.62769000
C	3.32620700	-4.55463800	-1.99276900
H	3.78838300	-5.46620800	-1.62650000
C	3.91111500	-3.84436000	-3.04129500
H	4.83436000	-4.19808300	-3.48880300
C	3.29581800	-2.68847900	-3.52792300
H	3.73986400	-2.13971500	-4.35152600
C	2.10790500	-2.23542500	-2.95972900
H	1.62463400	-1.35382700	-3.36699600
C	-1.20154400	3.40271900	-1.93978200
C	-2.49948300	2.90795600	-2.12849500
H	-2.81141500	1.97037400	-1.68878000
C	-3.42096800	3.62397500	-2.88773000
H	-4.41578500	3.21762600	-3.02128500
C	-3.05438600	4.83846600	-3.47097700
H	-3.76973800	5.39266300	-4.07047700
C	-1.76562000	5.34125000	-3.28170900
H	-1.47774900	6.28757400	-3.72835500
C	-0.84103500	4.63201300	-2.51564800
H	0.15281400	5.03836800	-2.36703000
C	-0.40939700	3.24771100	0.82587400
C	0.36420600	2.89162400	1.94147500
H	1.20643800	2.22636200	1.82006400
C	0.07590300	3.40626900	3.20326600
H	0.68868500	3.12264900	4.05381000
C	-0.99311100	4.29072200	3.37016100
H	-1.21040000	4.70643400	4.34942200
C	-1.76481000	4.65803100	2.26586000
H	-2.58148000	5.36405900	2.38254500
C	-1.47727100	4.14178900	0.99927600
H	-2.07504100	4.44821300	0.14891400
C	1.60872500	3.14356000	-1.32993500
C	1.97071500	2.98533100	-2.68064000
H	1.28225000	2.51961400	-3.37893100
C	3.19290900	3.47484300	-3.13780500
H	3.44629500	3.38525200	-4.18984200
C	4.08000100	4.09324700	-2.24990100
H	5.02965800	4.47731000	-2.60911300
C	3.73651700	4.22313000	-0.90379800
H	4.41941900	4.69867900	-0.20762500
C	2.49963300	3.76267100	-0.44479000
H	2.23135700	3.91528900	0.59295100
C	4.88084100	1.44879000	1.43070600
C	6.13902000	1.92345400	1.02514700
H	6.73748600	1.36107900	0.31663000
C	6.61622500	3.13276300	1.52959100
H	7.58983300	3.49724000	1.21848500
C	5.84430700	3.87173600	2.42920000
H	6.21931800	4.81332400	2.81775500
C	4.59279300	3.39853800	2.83542400
H	3.99534100	3.97185400	3.53718100
C	4.11050500	2.18894000	2.34224500
H	3.14412800	1.82597400	2.66979100
C	4.70347500	-1.49190100	1.84510800
C	4.20572300	-2.76962700	1.53101700
H	3.62360700	-2.92930500	0.62805800
C	4.48804900	-3.84047900	2.37451100
H	4.11155400	-4.82976700	2.13418400
C	5.26231500	-3.64236200	3.52358500
H	5.48265700	-4.48041400	4.17723600
C	5.76287200	-2.37472900	3.82816400
H	6.37260900	-2.22790600	4.71367100
C	5.48676400	-1.29258400	2.99079900
H	5.87544200	-0.30706200	3.22599400
C	5.01345800	-0.34534400	-0.86390100
C	5.81792100	-1.45762300	-1.14629000
H	5.97895200	-2.22778000	-0.40106600
C	6.43078700	-1.55971100	-2.39578900
H	7.06927200	-2.41035900	-2.61072600
C	6.23423400	-0.56937100	-3.35928900
H	6.72039900	-0.65157500	-4.32644400
C	5.42882800	0.53883600	-3.07582500
H	5.28833700	1.31868900	-3.81696900
C	4.82746400	0.66313900	-1.82709800
H	4.25460200	1.55266100	-1.59347600
H	2.37435900	0.10821000	-1.54331300
O	-5.34844500	1.19356500	-1.58735900
C	-5.25959400	-0.04554300	-2.30263700
H	-4.68541300	-0.73039200	-1.66702400
H	-6.25997700	-0.48217800	-2.41401600
C	-4.58142300	0.12156900	-3.65513300
H	-3.57317200	0.53191800	-3.54026300
H	-4.49279700	-0.84673200	-4.15726600
H	-5.15122600	0.79392200	-4.30200900
C	-6.66848900	1.75067800	-1.49659900
H	-7.33730100	1.01289800	-1.02826400
H	-6.56841400	2.59561000	-0.80763000
C	-7.25215500	2.21783400	-2.82290200
H	-6.61390400	2.97061800	-3.29595300
H	-7.38839100	1.38896700	-3.52419100
H	-8.23517200	2.66885600	-2.65093600
P	-3.72161800	0.24383100	2.02135900
C	-3.73772800	0.32377600	3.81445100
C	-4.16915400	-0.77789600	4.56894000
C	-3.28632800	1.49457800	4.44966700
C	-4.14366800	-0.70635900	5.96169000
H	-4.52982900	-1.67465000	4.07588900
C	-3.26626600	1.55242600	5.84023200
H	-2.94236100	2.34580000	3.87040500
C	-3.69318400	0.45444200	6.59497600
H	-4.48654000	-1.55064100	6.55081100
H	-2.92712100	2.45524000	6.33804500
H	-3.68353000	0.50842600	7.67901000
C	-4.75666400	-1.09213100	1.41149500
C	-6.10202300	-0.84928100	1.09243200
C	-4.21502100	-2.38003400	1.27275400
C	-6.89650300	-1.89403700	0.62403600
H	-6.52906200	0.14155100	1.19908600

C	-5.02007200	-3.41771000	0.80726000
H	-3.17463500	-2.57165800	1.51438400
C	-6.35693600	-3.17526800	0.47961500
H	-7.93662200	-1.70967500	0.37556000
H	-4.60458900	-4.41414200	0.69803000
H	-6.98121900	-3.98606700	0.11753600
C	-4.32153500	1.82178200	1.36085600
H	-3.57056400	2.59438600	1.54024200
H	-4.50468400	1.71093700	0.28561600
H	-5.25136500	2.09701100	1.86647900

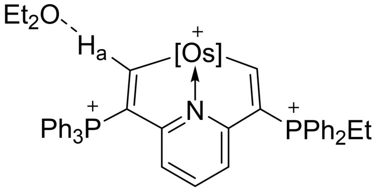

6c-1

[Os] = $OsCl(PPh_3)_2$

E = -5180.15247621

Os	0.03391100	-0.09833100	-0.91551300
Cl	0.44948700	-0.33557400	-3.17395600
P	0.02354600	2.33948200	-1.26770300
P	-0.00399600	-2.55918900	-0.75517800
P	-4.34528000	0.10666400	0.77011500
N	-0.31224800	0.15361300	1.23085800
C	-1.92601500	-0.05880000	-0.57898300
C	-2.54652400	0.08175300	0.61239400
C	-1.58803000	0.23584600	1.70422800
C	-1.83493300	0.46326200	3.05758800
H	-2.85119100	0.55392000	3.41877700
C	-0.75147300	0.61399400	3.92175800
H	-0.92221900	0.81485700	4.97442100
C	0.54829500	0.51088900	3.43865700
H	1.39370200	0.62588300	4.10036600
C	0.74963300	0.25679800	2.07890000
C	1.99789700	0.08758100	1.34468600
C	1.78760200	-0.03971200	0.01509300
H	2.58476200	-0.14260900	-0.72633800
C	1.35423200	2.87847400	-2.38538400
C	1.06683800	3.45412900	-3.62945000
H	0.03933800	3.57986800	-3.95091000
C	2.10739500	3.87656700	-4.45892300
H	1.87815200	4.31961800	-5.42270400
C	3.43336400	3.74517200	-4.04605400
H	4.23843700	4.08960500	-4.68743600
C	3.72319000	3.17500700	-2.80306700
H	4.75173200	3.07960900	-2.47079900
C	2.69155400	2.72602300	-1.98212500
H	2.92943100	2.29215700	-1.01553700
C	0.20096700	3.35442000	0.24657200
C	1.23082900	4.28898800	0.41887000
H	1.97809600	4.43123100	-0.35112200
C	1.28610700	5.06431600	1.58049000
H	2.07441200	5.80245300	1.69340000
C	0.32269500	4.91837400	2.57845200
H	0.36951100	5.52857800	3.47501500
C	-0.72111400	4.00556900	2.40104300
H	-1.49793300	3.90276600	3.15262100
C	-0.78205500	3.23886200	1.24229900
H	-1.61958700	2.57386200	1.09461600
C	-1.55457200	2.89933300	-2.00595500
C	-2.16171000	4.09382100	-1.58837600
H	-1.70329900	4.70070100	-0.81633600
C	-3.35458100	4.51728700	-2.17795900
H	-3.81186700	5.44718100	-1.85408500
C	-3.94313500	3.76291500	-3.19338500
H	-4.86458800	4.10056000	-3.65688000
C	-3.33350600	2.58310000	-3.62601600
H	-3.77969200	1.99924700	-4.42398600
C	-2.14869300	2.14963200	-3.03585400
H	-1.67209700	1.24653100	-3.40073600
C	1.15993300	-3.45878700	-1.82450800
C	2.45247200	-2.96242900	-2.04355200
H	2.75403400	-1.99718900	-1.65922200
C	3.38029800	-3.71197300	-2.76240200
H	4.37037400	-3.30291300	-2.92011300
C	3.02446700	-4.96068700	-3.27560800
H	3.74459700	-5.54117000	-3.84372900
C	1.74024400	-5.46360800	-3.05788100
H	1.46047900	-6.43564200	-3.45130500
C	0.81013600	-4.72127400	-2.33122400
H	-0.18015600	-5.12721500	-2.15920700
C	0.36136300	-3.18150900	0.92895800
C	-0.45681200	-2.83083000	2.01380500
H	-1.33233700	-2.21978200	1.84950200
C	-0.17009700	-3.28432000	3.29909900
H	-0.81922000	-3.00961000	4.12531800
C	0.94420400	-4.09800700	3.52101500
H	1.16070700	-4.46738100	4.51886500
C	1.76602900	-4.45209700	2.44907000
H	2.62201300	-5.10041900	2.61010000
C	1.47865500	-3.99919900	1.15859000
H	2.11226900	-4.30034800	0.33245400
C	-1.65221500	-3.19301200	-1.24099500
C	-2.01157100	-3.06730000	-2.59591600
H	-1.32736200	-2.60520100	-3.30085300
C	-3.22468500	-3.58331000	-3.04777700
H	-3.47520500	-3.51811200	-4.10228700
C	-4.10622600	-4.19683600	-2.15105000
H	-5.04907700	-4.60127600	-2.50580700
C	-3.76500300	-4.29678000	-0.80184600
H	-4.44280900	-4.76997000	-0.09907000
C	-2.53643000	-3.80983200	-0.34752600
H	-2.26961000	-3.94131100	0.69330900
C	-4.92864700	-1.44424200	1.48107700
C	-6.17227600	-1.95391700	1.07355000
H	-6.76993000	-1.42549700	0.33881400
C	-6.63598900	-3.15454000	1.61046700
H	-7.59857900	-3.54589000	1.29771100
C	-5.86509000	-3.85018200	2.54470100
H	-6.22949100	-4.78523200	2.95832100
C	-4.62873400	-3.34125200	2.95425500
H	-4.03282700	-3.88006100	3.68409500
C	-4.16031300	-2.13981600	2.42895400
H	-3.20700900	-1.74729200	2.76095500
C	-4.77604700	1.50937100	1.80926600
C	-4.30318200	2.78490100	1.45030900
H	-3.73019100	2.92635400	0.53840900
C	-4.59785100	3.87736100	2.26133900
H	-4.24074500	4.86462500	1.98587100
C	-5.35928700	3.70312300	3.42277500
H	-5.58937100	4.55800400	4.05077000
C	-5.83491700	2.43750000	3.77220900

H	-6.43507000	2.30873500	4.66702000
C	-5.54677000	1.33402700	2.96743800
H	-5.91716500	0.35025800	3.23693400
C	-5.06847500	0.28158100	-0.86720200
C	-5.86414000	1.38829700	-1.19326400
H	-6.03081500	2.18160200	-0.47414100
C	-6.46152600	1.45517300	-2.45253400
H	-7.09253700	2.30222400	-2.70103500
C	-6.25975300	0.43428800	-3.38247500
H	-6.73414900	0.48906200	-4.35739000
C	-5.46450000	-0.66909300	-3.05518700
H	-5.32017200	-1.47251200	-3.76987500
C	-4.87724300	-0.75737900	-1.79665300
H	-4.30993200	-1.64120700	-1.52979100
H	-2.44299400	-0.15985800	-1.53554700
O	5.31077700	-1.20124200	-1.80752800
C	5.07924500	-0.01101800	-2.57147200
H	4.44657700	0.63000500	-1.94829500
H	6.02646700	0.52523200	-2.71924800
C	4.38821400	-0.27920400	-3.90111900
H	3.44309700	-0.80854600	-3.74791400
H	4.16608000	0.67013100	-4.39979300
H	5.01246300	-0.88033700	-4.56694400
C	6.66425100	-1.68172500	-1.80616300
H	7.32525500	-0.89378800	-1.41178600
H	6.66326700	-2.50585200	-1.08621700
C	7.17444200	-2.16049500	-3.15820800
H	6.53271200	-2.94559600	-3.57060800
H	7.23506800	-1.34512200	-3.88515500
H	8.18201700	-2.57341400	-3.04172600
P	3.69615300	-0.03320800	1.95568800
C	3.73903500	-0.14988900	3.74743600
C	4.33427600	0.84585800	4.53411700
C	3.18565700	-1.29756600	4.34542800
C	4.36375700	0.69710500	5.92183300
H	4.77904300	1.72084800	4.07314800
C	3.22256800	-1.43392700	5.72919100
H	2.72229600	-2.07106800	3.74019500
C	3.80962100	-0.43717900	6.51741900
H	4.82925100	1.46226900	6.53437900
H	2.80138200	-2.31887500	6.19557500
H	3.84223000	-0.55112100	7.59631700
C	4.58106000	1.41416900	1.35603000
C	5.74217000	1.27970500	0.58057800
C	4.06636500	2.69111400	1.64457300
C	6.39347200	2.42034900	0.10987600
H	6.12918000	0.30125900	0.32654500
C	4.72907400	3.82149000	1.17502300
H	3.15177400	2.80394700	2.21913500
C	5.89292800	3.68810400	0.41088400
H	7.29522400	2.31640500	-0.48495800
H	4.34008700	4.80758100	1.40441200
H	6.40793100	4.57336800	0.05148000
C	4.39705800	-1.57087800	1.25889900
H	3.60893600	-2.32332100	1.37207500
H	4.54596800	-1.41238600	0.18246400
C	5.69330400	-2.03065500	1.94179500
H	5.53402900	-2.25619000	2.99891700
H	6.04778200	-2.94102300	1.45022300
H	6.48906500	-1.28412500	1.87364700

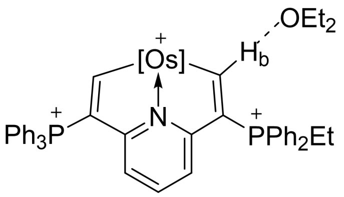

7c-1

[Os] = $OsCl(PPh_3)_2$

E = -5180.15937856

Os	-0.23740300	0.52938500	-0.45439000
Cl	0.03795400	2.18190500	-2.02796000
P	-0.40191100	-1.04327500	-2.33399700
P	-0.13128300	2.27369200	1.28344200
P	3.57907200	-1.70644900	1.13442300
N	-0.40741200	-1.07980000	1.01714500
C	1.59666500	-0.10492000	0.01700700
C	1.90469000	-1.09625400	0.88192200
C	0.71758400	-1.68826800	1.48483600
C	0.63514500	-2.72733600	2.41294100
H	1.53605000	-3.20263300	2.77885600
C	-0.62205700	-3.14768200	2.84546000
H	-0.70633200	-3.96272600	3.55685700
C	-1.76828000	-2.52251700	2.36245700
H	-2.75167500	-2.85310400	2.67318500
C	-1.63540700	-1.47460600	1.44874400
C	-2.67339100	-0.66243800	0.83141800
C	-2.15789000	0.24608000	-0.02843800
H	-2.76443500	0.96715900	-0.57993100
C	-1.43753000	-0.32447800	-3.64749700
C	-0.90653200	-0.04576500	-4.91245100
H	0.12136200	-0.30109800	-5.14339900
C	-1.70220400	0.56655700	-5.88329400
H	-1.28467500	0.77830100	-6.86240700
C	-3.02576900	0.90174100	-5.59988000
H	-3.63974000	1.37807400	-6.35753400
C	-3.56278900	0.61651000	-4.34046900
H	-4.59504500	0.86729400	-4.11664900
C	-2.77370400	0.01094900	-3.36720800
H	-3.20836700	-0.22102100	-2.39986700
C	-1.12749600	-2.67460700	-1.94010700
C	-2.23900100	-3.17857500	-2.62952100
H	-2.73851700	-2.57559500	-3.37834600
C	-2.68653700	-4.47849300	-2.38053900
H	-3.53774300	-4.86428400	-2.93258600
C	-2.03343200	-5.28315000	-1.44593500
H	-2.37157100	-6.30014300	-1.27100600
C	-0.92561500	-4.78480000	-0.75320400
H	-0.39708900	-5.40841200	-0.03859500
C	-0.47435900	-3.49212500	-1.00316200
H	0.41686200	-3.13999500	-0.50268000
C	1.20816800	-1.45388700	-3.09638100
C	1.45087000	-2.73997300	-3.60511300
H	0.69061100	-3.50930100	-3.53438500
C	2.67314400	-3.03281200	-4.21269200
H	2.85080500	-4.02752900	-4.60970400
C	3.65506900	-2.04701200	-4.32710000
H	4.60108000	-2.27668400	-4.80754000
C	3.40920300	-0.76053600	-3.84188800
H	4.16233700	0.01342800	-3.94216800
C	2.19405500	-0.46280700	-3.22861500
H	2.00387500	0.54728400	-2.88597500

C	-0.82649500	3.88044400	0.77266100
C	-1.94875500	3.94416000	-0.06625500
H	-2.38896400	3.04090900	-0.46947800
C	-2.50176500	5.17635100	-0.40821900
H	-3.35795000	5.21494200	-1.07444000
C	-1.94243600	6.35681300	0.08676900
H	-2.36715200	7.31705300	-0.18821400
C	-0.83318700	6.30007700	0.93253600
H	-0.39688900	7.21431200	1.32183200
C	-0.27468600	5.06915900	1.27563300
H	0.59102700	5.03724900	1.92739300
C	-1.00613800	1.87461600	2.84441000
C	-0.65995600	0.73558100	3.58937300
H	0.10810300	0.06415500	3.22813200
C	-1.27426600	0.47134700	4.81261700
H	-0.98379700	-0.40689800	5.38136100
C	-2.24670400	1.34180700	5.31153200
H	-2.71355000	1.14591100	6.27181600
C	-2.60522300	2.47264800	4.57468100
H	-3.34938800	3.16190400	4.96207400
C	-1.99109700	2.73959400	3.34804500
H	-2.25784000	3.63601800	2.80032600
C	1.59399300	2.65536200	1.74321600
C	2.41044600	3.17395500	0.72473400
H	2.05073600	3.28789300	-0.29168000
C	3.70503100	3.59451100	1.01758700
H	4.29970900	4.02642600	0.21943900
C	4.21088400	3.46427600	2.31504600
H	5.21915200	3.79497200	2.54530400
C	3.41062200	2.91797900	3.32066600
H	3.79749600	2.81670100	4.32967300
C	2.09714900	2.52813400	3.04388500
H	1.46948100	2.15633300	3.84572700
C	4.10208500	-1.39521500	2.82548400
C	5.42294200	-1.71542100	3.18915500
H	6.10451200	-2.15304900	2.46533400
C	5.86195100	-1.45372000	4.48421800
H	6.87919000	-1.70175100	4.76882800
C	4.99678700	-0.86346300	5.41146600
H	5.34527200	-0.65879500	6.41879300
C	3.69215200	-0.52817400	5.04408800
H	3.02745100	-0.05912000	5.76276000
C	3.24060200	-0.79072700	3.75176300
H	2.23536000	-0.50980600	3.46368900
C	3.52883500	-3.46702500	0.75745100
C	3.00770700	-3.85357300	-0.49070800
H	2.68182900	-3.10898900	-1.21013900
C	2.92052100	-5.20546000	-0.81059200
H	2.52313000	-5.50297500	-1.77566700
C	3.34681600	-6.17129700	0.10723700
H	3.28147200	-7.22479600	-0.14587300
C	3.86293300	-5.78596400	1.34649500
H	4.19728800	-6.53708000	2.05471100
C	3.95623600	-4.43359200	1.67865600
H	4.35528600	-4.13816400	2.64313400
C	4.70013400	-0.84324100	0.02430800
C	4.96191600	0.51297800	0.28876800
H	4.49019000	1.01559000	1.12600000
C	5.85667300	1.20712900	-0.51978000
H	6.06007400	2.25314600	-0.31887100
C	6.50497800	0.55275700	-1.57240200
H	7.21638600	1.09442600	-2.18749600

C	6.25195600	-0.79675200	-1.82410600
H	6.76707400	-1.30688000	-2.63161900
C	5.34637200	-1.50179900	-1.03107900
H	5.15872200	-2.55058800	-1.22803900
H	2.33972600	0.46946200	-0.53916600
O	3.00637100	3.77833200	-2.20858800
C	2.47893600	4.92027600	-2.88927200
H	3.29594000	5.55508000	-3.25548700
H	1.89861000	4.58464400	-3.76281400
C	1.59979000	5.69379500	-1.92234900
H	2.17193800	5.99903300	-1.04013700
H	1.21448400	6.59643900	-2.40695000
H	0.74971700	5.08915000	-1.59282400
C	3.72916000	2.87634700	-3.03402100
H	3.85401000	1.97409800	-2.42417800
C	5.09671300	3.39324300	-3.46820500
H	5.69219600	3.68178200	-2.59529700
H	5.63926900	2.61356900	-4.01539400
H	5.01807100	4.26136500	-4.12839900
H	3.12168200	2.60911900	-3.91434200
P	-4.41694700	-0.63082100	1.26995700
C	-4.49796600	-0.25614700	3.05899500
H	-3.96190500	-1.06060200	3.57224700
H	-3.89558500	0.64677100	3.19720500
C	-5.15550600	0.71893000	0.33517000
C	-5.83233700	0.45005500	-0.86573400
C	-4.98676300	2.04442600	0.77269400
C	-6.34228100	1.50681000	-1.61974300
H	-5.97261500	-0.57318600	-1.19824700
C	-5.50253500	3.09150400	0.01192700
H	-4.45535800	2.26234900	1.69387300
C	-6.17922300	2.82374800	-1.18192200
H	-6.88271600	1.30100300	-2.53823700
H	-5.38081700	4.11365500	0.35437800
H	-6.58977200	3.64160800	-1.76565100
C	-5.23723200	-2.17671000	0.87235600
C	-6.62917100	-2.27058700	1.06802100
C	-4.51489500	-3.27742200	0.38476800
C	-7.28371500	-3.46935100	0.80030000
H	-7.20039000	-1.41219200	1.40549300
C	-5.18384800	-4.47336800	0.12697700
H	-3.44965400	-3.20744400	0.19621500
C	-6.55978600	-4.57309700	0.33911900
H	-8.35606800	-3.54232200	0.94873300
H	-4.62697100	-5.32555800	-0.24510700
H	-7.07292600	-5.50784300	0.13674200
C	-5.91006900	-0.07893500	3.62762200
H	-6.47468300	-1.01397600	3.60756500
H	-5.83416100	0.24060500	4.67072800
H	-6.47413800	0.68499000	3.08439800

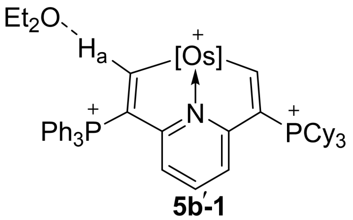

5b′-1

[Os] = OsCl$(PPh_3)_2$

E = -5343.56454343

Os	-0.08187700	-0.37796700	-0.67190900
Cl	-0.53720200	-1.66760200	-2.52436600

P	-0.08538700	1.56939000	-2.17213300
P	-0.30381700	-2.49020400	0.60597300
P	-3.56979900	1.47071700	1.86824700
N	0.35311400	0.87103600	1.06749900
C	-1.80475700	0.22891700	0.12199400
C	-1.95639000	1.00896700	1.21386100
C	-0.67607700	1.40434700	1.78678700
C	-0.42858200	2.19213200	2.91209300
H	-1.25206500	2.61493500	3.47245400
C	0.89168700	2.42319600	3.29498600
H	1.10420300	3.03086700	4.16852000
C	1.93649800	1.87324500	2.55816800
H	2.96317500	2.04757400	2.85058900
C	1.64652800	1.09569400	1.43355500
C	2.56620500	0.40958700	0.53522200
C	1.88948300	-0.26934100	-0.42074900
H	2.35358300	-0.87184800	-1.20207300
C	0.58420600	1.26242400	-3.83900100
C	0.07016600	1.98081700	-4.93005200
H	-0.75223800	2.67371800	-4.79052900
C	0.61500000	1.80617300	-6.20178100
H	0.21000400	2.36352800	-7.04034300
C	1.67148700	0.91482100	-6.39662800
H	2.08757400	0.77420000	-7.38913300
C	2.18723200	0.19792700	-5.31457700
H	2.99883700	-0.50603700	-5.46523500
C	1.64709400	0.37046300	-4.04173700
H	2.04349800	-0.20663100	-3.21585400
C	0.81063800	3.04116800	-1.56287000
C	1.84594900	3.61488300	-2.31406900
H	2.17871400	3.14373900	-3.23198700
C	2.42174900	4.82029500	-1.90637500
H	3.20648800	5.26914300	-2.50742600
C	1.97552900	5.45880700	-0.74868400
H	2.40730500	6.40875000	-0.44989300
C	0.96032500	4.87934700	0.01811400
H	0.60225200	5.37167400	0.91741100
C	0.38084200	3.68021200	-0.38774800
H	-0.44299100	3.27323400	0.18308900
C	-1.79180300	2.16582700	-2.47361400
C	-2.12789900	3.52553000	-2.41024400
H	-1.38303700	4.26341200	-2.13694600
C	-3.42262500	3.94334100	-2.72999300
H	-3.67039700	4.99964300	-2.68927600
C	-4.38251800	3.01258800	-3.12453100
H	-5.38311300	3.34117300	-3.38634500
C	-4.04796600	1.65805900	-3.20140100
H	-4.78929500	0.93322000	-3.51891900
C	-2.76242000	1.23360600	-2.87837800
H	-2.50569900	0.18388900	-2.97261700
C	0.46848800	-3.87957700	-0.29354800
C	1.83099000	-3.77978100	-0.61934600
H	2.39315500	-2.89656600	-0.33695100
C	2.47785800	-4.81936900	-1.28019800
H	3.53332800	-4.73520700	-1.52237200
C	1.76853300	-5.97277400	-1.62955000
H	2.26926900	-6.78271200	-2.15018600
C	0.41858800	-6.08254000	-1.29915600
H	-0.13496400	-6.97888400	-1.55983200
C	-0.23276700	-5.04336800	-0.62966000
H	-1.28046200	-5.14701300	-0.37591500
C	0.36192400	-2.65296900	2.30675300
C	0.42671000	-1.57028400	3.19352600
H	0.20819800	-0.57416500	2.84717000
C	0.74689000	-1.76965000	4.53759100
H	0.79161300	-0.91843700	5.21099800
C	0.99935400	-3.05783900	5.01308300
H	1.23817900	-3.21731300	6.05998700
C	0.95112100	-4.14195800	4.13308700
H	1.15249100	-5.14560000	4.49400700
C	0.63999800	-3.94421600	2.78837500
H	0.59443900	-4.79623700	2.11914900
C	-2.07435700	-2.91187100	0.81654800
C	-2.86310900	-3.18187000	-0.31409600
H	-2.46486300	-3.11630300	-1.31880600
C	-4.19144900	-3.57713400	-0.15976600
H	-4.76071600	-3.81918200	-1.05083600
C	-4.75949900	-3.66345000	1.11436800
H	-5.79289300	-3.97495900	1.23298200
C	-3.99219000	-3.34589500	2.23742700
H	-4.42748800	-3.39327500	3.23063800
C	-2.65070800	-2.98641500	2.09398900
H	-2.05965200	-2.78398400	2.97790700
C	-3.91161000	0.50986700	3.35123500
C	-5.24081900	0.40433000	3.79602300
H	-6.04462200	0.89240900	3.25326200
C	-5.52756300	-0.34959600	4.93227000
H	-6.55264400	-0.43237200	5.27837000
C	-4.49946500	-1.00366300	5.61830400
H	-4.72985300	-1.59277200	6.50040700
C	-3.17967900	-0.90433000	5.17067200
H	-2.38179700	-1.42060800	5.69545400
C	-2.88183900	-0.14852100	4.03911800
H	-1.85921200	-0.09331000	3.68926900
C	-3.51162300	3.23594300	2.20676400
C	-3.03415000	4.09166600	1.19742200
H	-2.74561500	3.69428800	0.22876500
C	-2.95597400	5.46002000	1.44051200
H	-2.59461300	6.12609100	0.66316900
C	-3.34879400	5.97495700	2.68119300
H	-3.28814500	7.04268800	2.86669900
C	-3.82549000	5.12350300	3.68018100
H	-4.13541800	5.52756400	4.63837300
C	-3.90924500	3.74940300	3.44908100
H	-4.27664700	3.08688500	4.22605400
C	-4.83766300	1.09822100	0.64907300
C	-5.60276800	2.11635400	0.06421600
H	-5.41954300	3.15610800	0.30946600
C	-6.61931400	1.77929700	-0.83010000
H	-7.22740700	2.56308000	-1.26984100
C	-6.85996100	0.44217000	-1.14981300
H	-7.65616700	0.18583600	-1.84179200
C	-6.09236300	-0.57126700	-0.56663700
H	-6.28723700	-1.61138100	-0.80289700
C	-5.08922200	-0.25148900	0.34354700
H	-4.53374100	-1.04358200	0.83249700
H	-2.63224600	-0.17097000	-0.46741500
O	-3.57640500	-3.07135400	-3.26172700
C	-3.20730600	-3.91404800	-4.35532900
H	-4.09795100	-4.37814200	-4.79834500
H	-2.72313500	-3.30604400	-5.13505400
C	-2.26018800	-4.97833500	-3.83060200
H	-2.74576600	-5.56916700	-3.04648800
H	-1.96904300	-5.65679700	-4.63856400

H	-1.35475900	-4.52520300	-3.41431900
C	-4.37492300	-1.95085600	-3.61017100
H	-4.34661400	-1.30080000	-2.72813100
C	-5.82229500	-2.29911200	-3.94343200
H	-6.28497000	-2.85569400	-3.12069600
H	-6.40281100	-1.38407700	-4.11077000
H	-5.90204400	-2.90637400	-4.84931600
H	-3.90826200	-1.40539200	-4.44677800
P	4.36424400	0.28192000	0.76497400
C	4.98925900	-0.54052500	-0.76172200
C	4.85274700	0.36594000	-2.00657000
C	6.41288900	-1.13467900	-0.66851600
H	4.30745300	-1.39465100	-0.87895100
C	5.16082500	-0.44167200	-3.27527000
H	5.56600400	1.19367300	-1.92671600
H	3.85650300	0.81582600	-2.06905800
C	6.71519900	-1.93486900	-1.94620000
H	7.14793400	-0.33255500	-0.55713900
H	6.50946400	-1.78499500	0.20433500
C	6.55362400	-1.07932800	-3.20789700
H	5.07564000	0.21356100	-4.14826400
H	4.40094100	-1.22915300	-3.39390000
H	7.73089300	-2.33816100	-1.88390300
H	6.03689500	-2.79940800	-1.99594300
H	6.73434200	-1.68688600	-4.10027200
H	7.31371200	-0.28647000	-3.21006100
C	5.11047300	1.94441700	1.07191700
C	6.63481400	1.98237200	0.80475600
C	4.40373300	3.09740100	0.33109000
H	4.96860800	2.09019700	2.15290800
C	7.21162000	3.32545200	1.27658000
H	6.81219700	1.87504800	-0.27092200
H	7.15018300	1.15721300	1.30153500
C	4.99163000	4.43947200	0.79372100
H	4.54943100	2.98106500	-0.74834500
H	3.32421600	3.08168900	0.50314900
C	6.50983100	4.50333300	0.59330500
H	8.28674500	3.34491400	1.07113800
H	7.09886900	3.40401800	2.36682900
H	4.50044400	5.25112600	0.25018600
H	4.75221900	4.58496900	1.85711200
H	6.90099800	5.45051100	0.97792000
H	6.73454200	4.48460100	-0.48221500
C	4.57912900	-0.77309200	2.27030900
C	4.14677100	-2.22705900	1.98738800
C	5.97847600	-0.68681700	2.91788500
H	3.85450100	-0.34558000	2.98002200
C	4.25316000	-3.08250800	3.25784000
H	4.78608200	-2.66159800	1.20930600
H	3.11726800	-2.24529300	1.61811700
C	6.04244700	-1.56645200	4.17639900
H	6.74328900	-1.02139600	2.20858400
H	6.21445800	0.34921500	3.18097200
C	5.65535400	-3.01652600	3.86978700
H	3.97725100	-4.11418000	3.01564800
H	3.51744500	-2.72349500	3.98670100
H	7.05231500	-1.51325800	4.59556200
H	5.36301300	-1.15745600	4.93736900
H	5.69644300	-3.61818400	4.78341800
H	6.38538900	-3.45326700	3.17420700

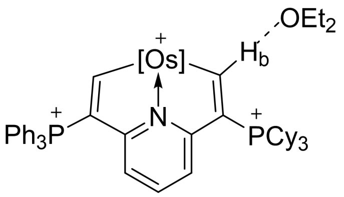

5b-1

[Os] = $OsCl(PPh_3)_2$

E = -5343.55359231

Os	-0.22993200	0.67739500	-0.59663500
Cl	-0.12398600	2.26533300	-2.27427500
P	-0.41547400	2.50656800	1.05259600
P	-0.16079500	-1.07872400	-2.32224800
P	-4.55260500	-0.74936500	0.55348400
N	-0.51048400	-0.78344200	1.00045000
C	-2.18381900	0.33185700	-0.40645500
C	-2.76290900	-0.50137300	0.48183900
C	-1.77977200	-1.14640400	1.34607000
C	-2.01978700	-2.02141200	2.40350200
H	-3.03314700	-2.27364000	2.68638700
C	-0.93010200	-2.52873300	3.10100000
H	-1.08218400	-3.19975400	3.94013400
C	0.36013900	-2.17778600	2.72639900
H	1.18384200	-2.58514000	3.28096700
C	0.57134800	-1.30712400	1.65179500
C	1.82318800	-0.80758600	1.05867600
C	1.54313100	0.09029000	0.08045500
H	2.28416100	0.64665000	-0.50005300
C	0.65535100	3.95861900	0.79691900
C	0.32062000	5.15694000	1.45220400
H	-0.59971000	5.23102600	2.02141300
C	1.16899300	6.25826900	1.37432500
H	0.90172800	7.18044500	1.88024400
C	2.35730700	6.17548600	0.64377100
H	3.01752400	7.03486700	0.58218300
C	2.68629400	4.99103000	-0.01514600
H	3.59941300	4.92640400	-0.59594500
C	1.83911800	3.88378800	0.05368100
H	2.11029000	2.99041200	-0.49408000
C	-0.02928900	1.95748500	2.75497600
C	1.15950300	2.35934200	3.38002200
H	1.81418000	3.07251800	2.89463800
C	1.48810400	1.87335500	4.64743500
H	2.40486500	2.20391700	5.12659300
C	0.62616500	0.99957700	5.31160900
H	0.87511400	0.63752500	6.30436900
C	-0.57704600	0.62288300	4.70813700
H	-1.27015300	-0.03098900	5.22875000
C	-0.90056200	1.09585800	3.43950600
H	-1.84354900	0.80889400	2.99359800
C	-2.10779500	3.20218600	1.12812600
C	-2.72404300	3.52224100	2.34693700
H	-2.22671700	3.30749400	3.28598800
C	-3.97666000	4.14163500	2.35792000
H	-4.44189600	4.39422200	3.30591200
C	-4.61565000	4.45426700	1.15822900
H	-5.58361400	4.94517500	1.17005600
C	-3.99666600	4.15510300	-0.05819400
H	-4.48227600	4.40955800	-0.99408100
C	-2.75107700	3.53350200	-0.07623200
H	-2.26531900	3.33851400	-1.02650200

C	0.98515400	-0.85462400	-3.71788400
C	2.04289100	0.06049400	-3.69306500
H	2.18067000	0.75494800	-2.87458500
C	2.92909300	0.12281100	-4.77014800
H	3.73081900	0.85001300	-4.74738500
C	2.76883200	-0.71949600	-5.86883600
H	3.45890400	-0.66168000	-6.70478100
C	1.70848200	-1.63051800	-5.90049700
H	1.57252900	-2.28231700	-6.75758900
C	0.81759700	-1.69706300	-4.83364500
H	-0.00613300	-2.40319800	-4.86584300
C	0.29094000	-2.71436400	-1.63131800
C	-0.53058400	-3.34518000	-0.68483800
H	-1.46520200	-2.88760600	-0.39580500
C	-0.16691900	-4.56824800	-0.12629100
H	-0.81973100	-5.04511400	0.59894600
C	1.03032200	-5.18190200	-0.50362000
H	1.31078900	-6.14034100	-0.07796600
C	1.85398000	-4.56413900	-1.44600500
H	2.77647800	-5.04308900	-1.76073100
C	1.48927400	-3.33837700	-2.00749300
H	2.12868700	-2.87985800	-2.75165500
C	-1.78447700	-1.25847700	-3.15748100
C	-2.16894900	-0.21475700	-4.01907800
H	-1.52600700	0.64989100	-4.14842300
C	-3.35568500	-0.30699600	-4.74376400
H	-3.62701200	0.48860300	-5.43108000
C	-4.18093800	-1.42786200	-4.60307900
H	-5.10101900	-1.50133700	-5.17443100
C	-3.80964400	-2.45745300	-3.73799700
H	-4.44011100	-3.33314000	-3.62602100
C	-2.61119400	-2.38069200	-3.02327300
H	-2.32156500	-3.21197200	-2.39403700
C	-5.00246100	-2.35126500	-0.14379400
C	-6.20124100	-2.46483900	-0.86637900
H	-6.83739900	-1.59976900	-1.01684700
C	-6.57197300	-3.69844500	-1.40135700
H	-7.50115600	-3.78536700	-1.95513100
C	-5.75160600	-4.81492600	-1.22655000
H	-6.04298600	-5.77238500	-1.64669700
C	-4.55997000	-4.70422200	-0.50307300
H	-3.92630300	-5.57399400	-0.36157200
C	-4.18653300	-3.47930200	0.04338500
H	-3.27046600	-3.40849800	0.61679000
C	-5.04327400	-0.63905700	2.27894000
C	-4.61494000	0.47195500	3.02691000
H	-4.03289800	1.26000200	2.55943800
C	-4.96792500	0.57048500	4.36957200
H	-4.64640600	1.42963300	4.94996700
C	-5.74186900	-0.43168700	4.96593700
H	-6.01659200	-0.35094800	6.01291000
C	-6.17101500	-1.53100800	4.21908400
H	-6.77925600	-2.30074200	4.68277100
C	-5.82515700	-1.64099300	2.87115900
H	-6.15875700	-2.49427200	2.28970900
C	-5.35361400	0.53218000	-0.41971500
C	-6.24656400	1.43258500	0.17644100
H	-6.43885600	1.39640000	1.24239900
C	-6.90120300	2.37469400	-0.61799900
H	-7.60629100	3.06223400	-0.16229300
C	-6.65725300	2.42870200	-1.99107400
H	-7.17385900	3.16089200	-2.60383100
C	-5.76364000	1.52977300	-2.58380000
H	-5.58503900	1.55830600	-3.65360200
C	-5.11994900	0.57130100	-1.80694500
H	-4.47476600	-0.15880300	-2.28126800
H	-2.73150200	0.93252000	-1.13183000
O	3.55784600	2.73789100	-2.49833700
C	3.02921500	3.87579000	-3.21172700
H	2.12231400	4.17551500	-2.67894700
H	3.74203700	4.70724300	-3.14019300
C	2.69673700	3.57773800	-4.66555600
H	1.95907600	2.77396300	-4.74093700
H	2.27162100	4.47399800	-5.13096500
H	3.58264300	3.29000500	-5.23871600
C	4.96238100	2.79341700	-2.24264100
H	5.21224700	3.75231300	-1.76359000
H	5.14990300	2.00540700	-1.50582800
C	5.84450100	2.57880800	-3.46580000
H	5.63586500	1.61229900	-3.93631000
H	5.70224200	3.36554800	-4.21211800
H	6.89933100	2.59168900	-3.17006900
P	3.57231900	-1.23983800	1.43140700
C	4.31731900	-1.95454700	-0.11522500
C	4.49526800	-0.92828900	-1.25346000
C	5.64211700	-2.71791700	0.13088000
H	3.55490400	-2.68196900	-0.42379400
C	5.02324600	-1.59590100	-2.53332400
H	5.20128000	-0.15269100	-0.93757700
H	3.55212700	-0.43363900	-1.48891900
C	6.11397400	-3.39955100	-1.16247700
H	6.41687600	-2.02381200	0.46863700
H	5.52361300	-3.46993000	0.91129500
C	6.31240400	-2.38151300	-2.28698100
H	5.17490600	-0.82372200	-3.29332700
H	4.25278500	-2.26892300	-2.93146500
H	7.04402300	-3.94014000	-0.95913800
H	5.37249800	-4.15189400	-1.46773300
H	6.62950900	-2.88402500	-3.20637300
H	7.11988300	-1.68869600	-2.01283100
C	4.44594100	0.26897600	2.07285600
C	5.98693600	0.16964000	1.96548000
C	3.96142800	1.60353400	1.48187700
H	4.16708300	0.26239400	3.13774400
C	6.64746300	1.34778100	2.69802400
H	6.27172800	0.20377700	0.90785700
H	6.36160100	-0.77327500	2.36735600
C	4.62760100	2.77777700	2.21353500
H	4.20892100	1.65296500	0.41488900
H	2.87875700	1.68488200	1.57009100
C	6.15561700	2.69210000	2.15359600
H	7.73457000	1.26410600	2.59877300
H	6.42390900	1.27754100	3.77153000
H	4.27080800	3.71697200	1.78018600
H	4.30316900	2.76974500	3.26406500
H	6.60403500	3.51652400	2.71689500
H	6.48551000	2.80655800	1.11122200
C	3.59217100	-2.50688500	2.77801000
C	3.08758300	-3.87812500	2.27190500
C	4.91233000	-2.64795300	3.57216400
H	2.87932100	-2.09349400	3.50182800
C	2.89846400	-4.84883100	3.44616000
H	3.82752700	-4.30069400	1.58497500
H	2.16226100	-3.77985000	1.69585500

C	4.70319000	-3.63307000	4.73556400
H	5.71870000	-3.00998600	2.92809900
H	5.22370600	-1.67930200	3.97301000
C	4.19257800	-4.99593800	4.25506800
H	2.56837300	-5.82019800	3.06226800
H	2.09563700	-4.48262300	4.10383600
H	5.64765600	-3.74632200	5.27727400
H	3.98479600	-3.20008200	5.44620700
H	4.03033300	-5.65901200	5.11058400
H	4.95810900	-5.47413500	3.62897500

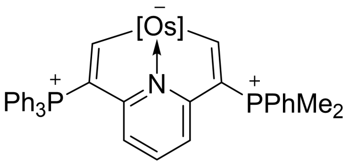

P2-6a

[Os] = $OsCl(PPh_3)_2$

E = -4715.46277896

Os	-0.60736500	-0.10650400	-0.64724900
Cl	-0.96689700	-0.06565600	-3.13622800
P	-0.39892800	-2.45681200	-0.89101300
P	-1.03161300	2.22011300	-0.75165700
P	3.83665700	0.33334000	0.55243500
N	-0.07603600	-0.17498700	1.30335400
C	1.41136700	0.20915600	-0.79166200
C	2.07589600	0.18274300	0.43260800
C	1.25442000	-0.05013000	1.62180500
C	1.64871300	-0.11947700	2.96336300
H	2.69237100	-0.01723600	3.23391400
C	0.68583800	-0.33713400	3.94811400
H	0.98640400	-0.41537100	4.98836000
C	-0.65757900	-0.46518900	3.60233500
H	-1.39398700	-0.66195900	4.37226300
C	-1.03694000	-0.36113700	2.25902300
C	-2.37273200	-0.41646900	1.66491100
C	-2.45285000	-0.36812600	0.27492200
H	-3.47364600	-0.41707500	-0.11584400
C	-1.69426900	-3.32731800	-1.85196500
C	-1.54584100	-4.68292100	-2.17933200
H	-0.63242300	-5.20875500	-1.92063100
C	-2.56675500	-5.36182300	-2.84169400
H	-2.44229800	-6.41100400	-3.09356700
C	-3.74350200	-4.69185700	-3.18579200
H	-4.53638500	-5.22059500	-3.70712000
C	-3.89158500	-3.34071800	-2.87116100
H	-4.79554800	-2.81006800	-3.15645600
C	-2.87048500	-2.65761700	-2.20947600
H	-2.95822500	-1.60130700	-1.99679400
C	-0.48190400	-3.29824700	0.74288600
C	-1.66049400	-3.90966100	1.18777800
H	-2.51175800	-3.98407300	0.51908700
C	-1.73763500	-4.43910700	2.47806900
H	-2.64897100	-4.93484800	2.80239100
C	-0.64556500	-4.35283700	3.34307000
H	-0.70661100	-4.77110300	4.34386500
C	0.53156600	-3.73973300	2.90765200
H	1.39021500	-3.66349800	3.56837700
C	0.61045900	-3.21789300	1.61957400
H	1.52748200	-2.74347500	1.29556600
C	1.18214000	-3.08050500	-1.59969500
C	1.79676000	-4.26560300	-1.16850200
H	1.34069100	-4.86069900	-0.38443700
C	3.00891800	-4.67629800	-1.72808200
H	3.47550200	-5.59676100	-1.38766700
C	3.61576400	-3.90912100	-2.72434300
H	4.56074200	-4.22721500	-3.15583200
C	2.99593800	-2.74063900	-3.17355300
H	3.45673100	-2.14391700	-3.95446700
C	1.78509100	-2.32728900	-2.61860800
H	1.29181100	-1.42889500	-2.97550800
C	-2.40679300	2.78146900	-1.82406500
C	-3.45094000	1.90317700	-2.13592500
H	-3.37978900	0.86891800	-1.83063900
C	-4.54998400	2.35197000	-2.86823200
H	-5.34902300	1.65876400	-3.11494900
C	-4.61036000	3.67709900	-3.30312200
H	-5.46189200	4.02295500	-3.88214500
C	-3.56439400	4.55475400	-3.00745400
H	-3.60127400	5.58384700	-3.35267200
C	-2.46633800	4.10925700	-2.27229200
H	-1.65583900	4.79438100	-2.04364100
C	-1.47239700	2.99496400	0.86847900
C	-0.62573400	2.80410200	1.97163300
H	0.27451300	2.21673400	1.85733600
C	-0.92888000	3.35146000	3.21687000
H	-0.25458000	3.18557200	4.05223600
C	-2.09670100	4.09828600	3.38732800
H	-2.33568500	4.52919200	4.35532500
C	-2.95439300	4.28789400	2.30194600
H	-3.86219800	4.87300000	2.42071000
C	-2.64470500	3.74274200	1.05257300
H	-3.31065400	3.91641800	0.21511300
C	0.38498800	3.23540700	-1.34551700
C	0.81696700	3.00723500	-2.66398200
H	0.31036500	2.26179900	-3.27024000
C	1.89144500	3.72715100	-3.18079500
H	2.20338800	3.55645100	-4.20719900
C	2.56734700	4.65913200	-2.38603800
H	3.40564200	5.21880300	-2.79178100
C	2.15831300	4.86892300	-1.06913600
H	2.67950000	5.58682300	-0.44219600
C	1.06248900	4.16959100	-0.55421800
H	0.73354500	4.36752400	0.45985800
C	4.35220900	1.83359400	1.42862700
C	5.71469600	2.15386800	1.54510400
H	6.47028500	1.48394000	1.14489500
C	6.09615500	3.34538100	2.15721000
H	7.14925800	3.59281900	2.24748800
C	5.12399000	4.22659400	2.64089300
H	5.42551700	5.15730200	3.11189300
C	3.76953700	3.91893200	2.50780500
H	3.01267100	4.60837200	2.86960300
C	3.38137400	2.72483100	1.90089900
H	2.33196500	2.49325900	1.77445000
C	4.49100700	-1.13300900	1.39283500
C	4.07665200	-2.37982900	0.89456000
H	3.43198700	-2.43653000	0.02341800
C	4.48760500	-3.55037300	1.52675700
H	4.16290900	-4.50812600	1.13331400
C	5.30187500	-3.48420800	2.66066900
H	5.61617900	-4.39745400	3.15685900
C	5.71017400	-2.24558700	3.15888700
H	6.33992700	-2.19408700	4.04173500

C	5.30819000	-1.06737700	2.52770600
H	5.61764700	-0.10692100	2.92557100
C	4.56704600	0.45204500	-1.09473900
C	5.29622600	-0.59914900	-1.66239900
H	5.45310600	-1.52048900	-1.11472400
C	5.81931700	-0.45856100	-2.94807800
H	6.38723300	-1.27280300	-3.38744600
C	5.61122300	0.71990100	-3.66531600
H	6.01763900	0.82347500	-4.66688900
C	4.88427600	1.76926400	-3.09593300
H	4.71775500	2.68652600	-3.65058000
C	4.36596400	1.64344700	-1.81085900
H	3.80339900	2.46201800	-1.37476000
H	2.02806300	0.41015300	-1.67177300
P	-3.83548700	-0.41383200	2.66180100
C	-3.82373400	0.91668700	3.91276400
H	-4.76815200	0.90861200	4.46446400
H	-2.99095600	0.79177100	4.60659600
H	-3.70099100	1.87651100	3.40552900
C	-4.09559200	-1.97576100	3.56732800
H	-4.98017700	-1.92151500	4.20822500
H	-4.19596800	-2.79465100	2.85204800
H	-3.21045900	-2.17988300	4.17516700
C	-5.26299700	-0.10176100	1.60431200
C	-6.25935900	-1.06457900	1.40322100
C	-5.33649400	1.13535400	0.94143000
C	-7.32285400	-0.79124000	0.54159100
H	-6.21110100	-2.02610700	1.90298900
C	-6.39714100	1.39853200	0.08036300
H	-4.55286200	1.87459200	1.07284100
C	-7.39042100	0.43538100	-0.11990200
H	-8.09348300	-1.53937900	0.38489800
H	-6.43539000	2.34519200	-0.44823900
H	-8.21587800	0.64107000	-0.79456500

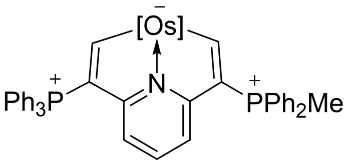

P2-6b

[Os] = $OsCl(PPh_3)_2$

E = -4907.31989416

Os	0.28387900	0.26810300	0.84584400
Cl	0.26317500	0.92943500	3.27324300
P	0.29702100	-1.94657100	1.70602900
P	0.35823500	2.60347000	0.44887600
P	-3.92151600	-0.28941400	-1.00774500
N	0.09176200	-0.30568200	-1.08459900
C	-1.75826600	0.27583200	0.64117500
C	-2.20164700	-0.11756400	-0.61915200
C	-1.17217500	-0.46157600	-1.60130300
C	-1.32456700	-0.88015800	-2.92863600
H	-2.31268800	-1.00636000	-3.35345200
C	-0.19065300	-1.15318400	-3.69511600
H	-0.30247700	-1.50501600	-4.71608300
C	1.08214200	-0.98225200	-3.15518200
H	1.96143700	-1.22776000	-3.73957500
C	1.21246300	-0.52544200	-1.83741000
C	2.42805900	-0.21976200	-1.08575700
C	2.27203900	0.13230200	0.25441400
H	3.21352100	0.36815300	0.76049700
C	1.43945900	-2.29124300	3.09633200
C	1.23479000	-3.39473900	3.93604300
H	0.35677700	-4.01952100	3.80866100
C	2.15460600	-3.69315700	4.94093900
H	1.98557800	-4.54769900	5.58971500
C	3.28482500	-2.89215700	5.11538700
H	3.99813300	-3.12274300	5.90147700
C	3.48842600	-1.78766800	4.28644700
H	4.35537300	-1.14939400	4.43171900
C	2.56767600	-1.48369400	3.28471600
H	2.69654400	-0.60785900	2.66409100
C	0.75869500	-3.24226600	0.47853300
C	1.88215900	-4.06332900	0.63625400
H	2.51928500	-3.94631400	1.50550700
C	2.17280200	-5.05214200	-0.30797600
H	3.03736400	-5.69342500	-0.16044700
C	1.35628100	-5.22135000	-1.42698500
H	1.57988600	-5.99535000	-2.15595400
C	0.24270500	-4.39479200	-1.60040100
H	-0.40224700	-4.51207600	-2.46643900
C	-0.05354300	-3.41891000	-0.65300600
H	-0.93300300	-2.80349000	-0.78662400
C	-1.32503700	-2.57695100	2.31766900
C	-1.76714200	-3.88829800	2.09073000
H	-1.14315700	-4.58563200	1.54237600
C	-3.01682100	-4.30353700	2.55901900
H	-3.34956700	-5.32163700	2.37591700
C	-3.83020800	-3.41573000	3.26387400
H	-4.80281900	-3.73800300	3.62529300
C	-3.38288100	-2.11599600	3.51471200
H	-4.00758700	-1.42193200	4.06812900
C	-2.13882000	-1.69625400	3.04757900
H	-1.78288900	-0.69130000	3.25058300
C	1.44876200	3.60837900	1.52539800
C	2.54940100	3.01516600	2.15424600
H	2.68664700	1.94577200	2.08288100
C	3.43228700	3.79263400	2.90361500
H	4.27526000	3.31899200	3.39837000
C	3.21878800	5.16540500	3.03897500
H	3.90122200	5.76790700	3.63165900
C	2.11433700	5.76122800	2.42525000
H	1.93691200	6.82696300	2.53623200
C	1.23173800	4.98643800	1.67329200
H	0.37397200	5.45312800	1.19907400
C	0.91132500	3.11678400	-1.24095000
C	0.29799600	2.54031700	-2.36446900
H	-0.46577900	1.78864200	-2.22737300
C	0.65437300	2.92175800	-3.65695200
H	0.15973400	2.45872700	-4.50623200
C	1.64525500	3.88580200	-3.85374600
H	1.92401000	4.18888700	-4.85875200
C	2.27916400	4.45325100	-2.74678200
H	3.05144100	5.20440900	-2.88726500
C	1.91630700	4.07287100	-1.45137900
H	2.40318700	4.54001000	-0.60310400
C	-1.26924100	3.45125100	0.62452100
C	-1.85600600	3.44225000	1.90237400
H	-1.34029700	2.95181600	2.72267800
C	-3.09230300	4.05013500	2.10797100
H	-3.52614700	4.05297000	3.10390000
C	-3.77283700	4.64960600	1.04284600
H	-4.73700500	5.12319800	1.20626800

C	-3.20645600	4.63723300	-0.23169700
H	-3.72914700	5.09371800	-1.06744200
C	-1.95360100	4.05179300	-0.43808600
H	-1.51134200	4.07925200	-1.42746600
C	-4.48107000	0.90590300	-2.24950500
C	-5.84201900	0.99375500	-2.58476600
H	-6.56270600	0.32310000	-2.12568800
C	-6.27078000	1.96030600	-3.49145700
H	-7.32278000	2.02859300	-3.75040300
C	-5.35007700	2.84948000	-4.05466000
H	-5.68960400	3.60565100	-4.75603200
C	-4.00052300	2.77546900	-3.70770200
H	-3.28580100	3.47398900	-4.13241400
C	-3.56430700	1.80653300	-2.80461200
H	-2.52285600	1.76079800	-2.51524900
C	-4.21059600	-1.97939600	-1.59381400
C	-3.66374600	-3.01223500	-0.81437400
H	-3.13627400	-2.78437200	0.10588500
C	-3.79309800	-4.33535300	-1.22782600
H	-3.36620100	-5.12684200	-0.62008200
C	-4.45824500	-4.63294700	-2.42039500
H	-4.55284800	-5.66448100	-2.74608600
C	-4.99955500	-3.60625900	-3.19674600
H	-5.51342100	-3.83802600	-4.12458700
C	-4.87898100	-2.27743200	-2.78723100
H	-5.28840900	-1.48146200	-3.40005900
C	-4.93321600	0.01230800	0.45594000
C	-5.61384300	-1.02071400	1.10989300
H	-5.54888100	-2.03949300	0.74683800
C	-6.37373000	-0.73340500	2.24413400
H	-6.90419700	-1.53382400	2.75048900
C	-6.44523000	0.57295500	2.72833600
H	-7.03470200	0.79089800	3.61387700
C	-5.76255800	1.60309000	2.07450600
H	-5.81217400	2.61991500	2.44953400
C	-5.01269500	1.33026200	0.93483300
H	-4.48982500	2.13445800	0.42849200
H	-2.53423600	0.55178400	1.35978300
P	3.98446300	-0.02808900	-1.90917200
C	3.84733500	1.13160500	-3.31178800
H	3.53740100	2.10897100	-2.93777800
H	4.80814000	1.22180500	-3.82486800
H	3.08824900	0.77715500	-4.01072800
C	5.17700300	0.68882100	-0.75863600
C	6.22081700	-0.08083300	-0.23045000
C	5.00223300	2.02037100	-0.34616100
C	7.08891300	0.48309900	0.70532200
H	6.35091800	-1.11145100	-0.54284700
C	5.87124700	2.57404000	0.58931100
H	4.17546400	2.61132900	-0.72724600
C	6.91477100	1.80611400	1.11422000
H	7.89775700	-0.11307600	1.11605700
H	5.72042900	3.59600200	0.92071400
H	7.59041000	2.23969000	1.84537500
C	4.64896500	-1.57605800	-2.56538000
C	5.79386400	-1.57195300	-3.37961500
C	4.01311700	-2.78395600	-2.24923200
C	6.29673500	-2.77207700	-3.87442200
H	6.29902600	-0.63983700	-3.61602700
C	4.52044800	-3.98075300	-2.75608300
H	3.13298100	-2.78898900	-1.61531800
C	5.65676100	-3.97625600	-3.56517000
H	7.18375800	-2.76986500	-4.50024600
H	4.02222800	-4.91267300	-2.51414600
H	6.04828900	-4.91078200	-3.95573000

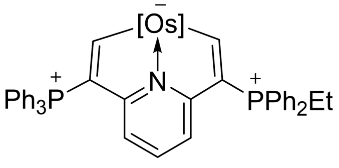

P2-6c

$[Os] = OsCl(PPh_3)_2$

Os	0.09340600	-0.15178200	-1.09720600
Cl	-0.26077100	-0.22089200	-3.58813500
P	0.36416500	2.20127000	-1.32827700
P	-0.06352600	-2.51353700	-1.18318000
P	-3.86245500	0.40614400	1.21997200
N	0.12870600	0.01127500	0.92168200
C	-1.90912700	0.00975800	-0.70456900
C	-2.20672900	0.14827100	0.64677800
C	-1.07058200	0.16755600	1.57338700
C	-1.08742000	0.29458500	2.96501500
H	-2.02492800	0.40369700	3.49609800
C	0.12219700	0.29907900	3.65714600
H	0.12660400	0.42104100	4.73608400
C	1.32918700	0.16458100	2.97840500
H	2.25421200	0.19371200	3.52929800
C	1.32713800	-0.00320000	1.58723900
C	2.43809400	-0.25356900	0.65909600
C	2.11801000	-0.37529700	-0.69126900
H	2.96315500	-0.65622400	-1.33660600
C	1.60182500	2.79050300	-2.55072900
C	1.82798900	4.16330300	-2.73007400
H	1.23574600	4.88810700	-2.18063500
C	2.81613000	4.60606600	-3.60704100
H	2.98483700	5.67099800	-3.73780700
C	3.58188400	3.68057400	-4.32244600
H	4.34484600	4.02637900	-5.01427700
C	3.35132900	2.31377300	-4.15885700
H	3.92926100	1.59101200	-4.72773600
C	2.36438200	1.86794400	-3.27683200
H	2.15260000	0.81232800	-3.16799800
C	0.96563900	2.95867800	0.23849400
C	2.31858500	3.25516200	0.43857800
H	3.01565500	3.17860900	-0.38454000
C	2.78034900	3.66592500	1.69074800
H	3.83501700	3.89405000	1.82141500
C	1.89184000	3.79936700	2.75784600
H	2.24945100	4.12328400	3.73116200
C	0.53607400	3.52419400	2.56329000
H	-0.17002400	3.62479600	3.38227600
C	0.08115300	3.10112700	1.31819100
H	-0.96780600	2.87442000	1.18692700
C	-1.15142600	3.18422900	-1.69321500
C	-1.35308200	4.47724000	-1.18681200
H	-0.59763900	4.94035900	-0.56096500
C	-2.53788000	5.16500600	-1.45680100
H	-2.68216200	6.16656800	-1.06058300
C	-3.53223200	4.56838000	-2.23509500
H	-4.45625400	5.10203400	-2.43947300
C	-3.32763200	3.29033100	-2.75967800
H	-4.09200400	2.82260500	-3.37246300
C	-2.14379600	2.60100000	-2.49541700

H	-1.97463200	1.61465600	-2.91459100
C	0.78372100	-3.36724900	-2.56468000
C	1.84513300	-2.74024700	-3.22819400
H	2.08190000	-1.71279000	-2.98873100
C	2.54943600	-3.41871200	-4.22251000
H	3.36200900	-2.91972400	-4.74286400
C	2.19397600	-4.72346900	-4.56822300
H	2.73766200	-5.24743400	-5.34902300
C	1.12793400	-5.34993000	-3.91875000
H	0.84118600	-6.36148200	-4.19112200
C	0.42463600	-4.67513700	-2.92145800
H	-0.40321500	-5.16564300	-2.41881800
C	0.64663200	-3.37069000	0.29419000
C	0.22016200	-2.99649000	1.57856500
H	-0.54728800	-2.24342600	1.68972500
C	0.78409500	-3.56366500	2.71942000
H	0.44266700	-3.24365500	3.69949600
C	1.78902800	-4.52637400	2.59814000
H	2.22485700	-4.97870400	3.48483600
C	2.21853400	-4.91356300	1.32729800
H	2.99083600	-5.67045900	1.21990700
C	1.65437000	-4.33934400	0.18457600
H	2.00185700	-4.64952700	-0.79376600
C	-1.77508200	-3.17720200	-1.30474900
C	-2.49327700	-2.85412100	-2.46945000
H	-2.02776700	-2.23156600	-3.22855800
C	-3.79081800	-3.32920000	-2.64262300
H	-4.32790700	-3.09326000	-3.55680000
C	-4.40101100	-4.10046000	-1.64746000
H	-5.41396800	-4.46870900	-1.78482500
C	-3.70216900	-4.39678800	-0.47729500
H	-4.16980500	-4.98982200	0.30350100
C	-2.38778500	-3.94894800	-0.31197700
H	-1.84074600	-4.21811300	0.58502700
C	-4.44980900	-0.93959500	2.28235200
C	-5.76537600	-0.93289000	2.77388100
H	-6.42617400	-0.09870200	2.55624000
C	-6.22922500	-2.00935400	3.52606900
H	-7.24606700	-2.00419200	3.90611000
C	-5.39075700	-3.09968200	3.77865200
H	-5.75804800	-3.93959000	4.36061000
C	-4.08979900	-3.11613900	3.27466100
H	-3.44184300	-3.96790500	3.45819600
C	-3.61770100	-2.03907600	2.52489700
H	-2.61711500	-2.05773300	2.11286500
C	-3.94406200	1.97996100	2.11657600
C	-3.45966800	3.11635800	1.44674300
H	-3.09859400	3.04118300	0.42594100
C	-3.42886800	4.34611200	2.09922000
H	-3.05220400	5.21659600	1.57217600
C	-3.86863200	4.44764700	3.42177100
H	-3.83767800	5.40578000	3.93170300
C	-4.34509600	3.31826700	4.09000800
H	-4.68338600	3.39592000	5.11870700
C	-4.38571600	2.08324200	3.44120100
H	-4.74649100	1.20650500	3.96813700
C	-5.00402400	0.45851000	-0.17942700
C	-5.60204500	1.64748300	-0.61340400
H	-5.40537000	2.58272300	-0.10405000
C	-6.45669800	1.62661500	-1.71576200
H	-6.92138300	2.54881800	-2.05069300
C	-6.71181900	0.42840500	-2.38340500
H	-7.37619500	0.41728700	-3.24219800
C	-6.11770700	-0.75851000	-1.94589400
H	-6.31197800	-1.69252100	-2.46207200
C	-5.26844100	-0.75013400	-0.84374200
H	-4.80986400	-1.67475400	-0.51042800
H	-2.75953100	-0.01312100	-1.39111900
P	4.12987900	-0.56033100	1.10786900
C	4.42690100	-0.58697400	2.89091500
C	4.76650300	0.59246300	3.57125800
C	4.18290800	-1.76531200	3.61201900
C	4.86827900	0.58719300	4.96107800
H	4.92984900	1.51101500	3.01815700
C	4.28756900	-1.76245400	5.00169000
H	3.88208400	-2.67316800	3.10031400
C	4.63033400	-0.58888600	5.67656500
H	5.13020000	1.50096600	5.48539400
H	4.09801500	-2.67560400	5.55740500
H	4.71027600	-0.58981800	6.75936700
C	5.27282600	0.65426500	0.38319100
C	6.57474600	0.79002400	0.89480500
C	4.88984900	1.40291600	-0.73742900
C	7.46827300	1.67879200	0.29887600
H	6.88719500	0.21647800	1.76113700
C	5.78840300	2.28780700	-1.33167900
H	3.89344500	1.30564400	-1.14633600
C	7.07585800	2.42978000	-0.81189700
H	8.47017400	1.78456500	0.70349100
H	5.46990200	2.86106700	-2.19667000
H	7.77468800	3.12252400	-1.27106400
C	4.60866700	-2.20669300	0.44924300
H	3.89561700	-2.92400300	0.86687300
H	4.39853000	-2.16454600	-0.62430900
C	6.05900000	-2.61639200	0.71402400
H	6.23895700	-3.61277800	0.29879300
H	6.76306100	-1.92460000	0.24327600
H	6.27956200	-2.65400700	1.78539000

8. References

[1] P. R. Hoffmann and K. G. Caulton, *J. Am. Chem. Soc.*, 1975, **97**, 4221-4228.

[2] S. Y. Wu, X.Q. Guo, L. P. Zhou and Q. F. Sun, *Inorg. Chem.*, 2019, **58**, 7091-7098.

[3] G. M. Sheldrick, *Acta Cryst.*, 2015, **A71**, 3-8.

[4] G. M. Sheldrick, *Acta Cryst.*, 2015, **C71**, 3-8.

[5] M. J. Frisch, G. W. Trucks, H. B. Schlegel, G. E. Scuseria, M. A. Robb, J. R. Cheeseman, G. Scalmani, V. Barone, G. A. Petersson, H. Nakatsuji, X. Li, M. Caricato, A. V. Marenich, J. Bloino, B. G. Janesko, R. Gomperts, B. Mennucci, H. P. Hratchian, J. V. Ortiz, A. F. Izmaylov, J. L. Sonnenberg, D. Williams-Young, F. Ding, F. Lipparini, F. Egidi, J. Goings, B. Peng, A. Petrone, T. Henderson, D. Ranasinghe, V. G. Zakrzewski, J. Gao, N. Rega, G. Zheng, W. Liang, M. Hada, M. Ehara, K. Toyota, R. Fukuda, J. Hasegawa, M. Ishida, T. Nakajima, Y. Honda, O. Kitao, H. Nakai, T. Vreven, K. Throssell, J. A. Montgomery Jr., J. E. Peralta, F. Ogliaro, M. J. Bearpark, J. J. Heyd, E. N. Brothers, K. N. Kudin, V. N. Staroverov, T. A. Keith, R. Kobayashi, J. Normand, K. Raghavachari, A. P. Rendell, J. C. Burant, S. S. Iyengar, J. Tomasi, M. Cossi, J. M. Millam, M. Klene, C. Adamo, R. Cammi, J. W. Ochterski, R. L. Martin, K. Morokuma, O. Farkas, J. B. Foresman and D. J. Fox, *Gaussian 16 Rev.*, **A.03** (Gaussian Inc., 2016)

[6] C. Lee, W. Yang and R. G. Parr, *Phys. Rev.,* 1988, **B37**, 785-789.

[7] P. J. Stephens, F. J. Devlin, C. F. Chabalowski and M. J. Frisch, *J. Phys. Chem.,* 1994, **98**, 11623-11627.

[8] S. Grimme, J. Antony, S. Ehrlich and H. Krieg, *J. Chem. Phys.,* 2010, **132**, 154104.

[9] S. Grimme, *J. Comput. Chem.*, 2006, **27**, 1787-1799.

[10] S. Grimme, *J. Comput. Chem.*, 2004, **25**, 1463-1473.

[11] C. Peng, P. Y. Ayala, H. B. Schlegel and M. J. Frisch, *J. Comput. Chem.,* 1996, **17**, 49–56.

[12] J. Tao, J. P. Perdew, V. N. Staroverov and G. E. Scuseria, *Phys. Rev. Lett.,* 2003, **91**, 146401.

[13] A. V. Marenich, J. C. Cramer and D. G. Truhlar, *J. Phys. Chem., B* 2009, **113**, 6378-6396.

[14] E. D. Glendening, J. K. Badenhoop, A. E. Reed, J. E. Carpenter, J. A. Bohmann, K. P. C. M. Morales, C. R. Landis and F. Weinhold, **NBO 7.0** (Univ. of Wisconsin, 2018).
